# Complete diastereodivergence in asymmetric 1,6-addition reactions enabled by minimal modification of a chiral catalyst

**DOI:** 10.1038/ncomms14793

**Published:** 2017-03-20

**Authors:** Daisuke Uraguchi, Ken Yoshioka, Takashi Ooi

**Affiliations:** 1 Institute of Transformative Bio-Molecules (WPI-ITbM) and Department of Applied Chemistry, Graduate School of Engineering, Nagoya University, Nagoya 464-8601, Japan; 2 CREST, Japan Science and Technology Agency (JST), Nagoya University, Nagoya 464-8601, Japan

## Abstract

Catalytic systems that allow selective generation of any diastereomer of a reaction product bearing multiple stereocentres through minimal modification of a single catalyst scaffold remain elusive, particularly for carbon–carbon bond formations requiring simultaneous control of multiple selectivity factors. Here, we report a catalyst-directed pinpoint inversion of diastereochemical preference in the 1,6-addition of azlactones to δ-aryl dienyl carbonyl compounds with full control over other selectivities preserved. This rigorous diastereodivergence is enabled by the slight structural adjustment of a chiral iminophosphorane catalyst, providing access to all the stereoisomers with high regio-, distereo- and enantioselectivity. The utility of this method is demonstrated in the facile stereodivergent preparation of densely functionalized proline derivatives. The experimental and computational elucidation of the origin of the diastereodivergence is also reported.

## Abstract (web summary)

For reactions producing numerous chiral centres, accessing all potential stereoisomers is synthetically appealing but difficult. Here, the authors report a stereodivergent 1,6-addition of azlactones where minimal changes to the catalyst structure switches the product selectivity.

In the last few decades, numerous advances in the field of asymmetric synthesis have led to the establishment of reliable stereoselective methods for assembling enantiomerically enriched organic compounds^1^. Nevertheless, in reactions involving the creation of multiple stereogenic centres in a single bond-forming event, arbitrary access to all the possible product diastereomers is incredibly difficult because diastereochemical preference is largely governed by the inherent structural and stereoelectronic nature of substrates, while absolute stereochemistry can be dictated by the choice of enantiomeric chiral catalyst. This is particularly true in diastereo- and enantioselective bond formation between two prochiral intermediates. Precise stereochemical control becomes even more complicated when additional selectivity factors, such as chemo- and regioselectivity, are associated with this type of bond construction, despite offering an unparalleled synthetic tool for rapidly increasing molecular complexity in a highly predictable and controlled manner. As a powerful strategy for addressing this intrinsic problem, catalyst-directed diastereodivergence would be much sought after. Impressive progress has been made in the development of diastereodivergent asymmetric catalysis for carbon–carbon and carbon-heteroatom bond-forming reactions^2^,^3^,^4^,^5^,^6^,^7^,^8^,^9^,^10^,^11^,^12^,^13^,^14^,^15^,^16^,^17^,^18^,^19^,^20^,^21^,^22^,^23^,^24^,^25^,^26^,^27^,^28^,^29^,^30^,^31^,^32^. However, most of the processes currently available rely on using each elaborated catalyst, or the appropriate combination of two different catalysts, to access complementary diastereomers selectively. This situation demonstrates the formidable challenge faced in achieving rigorous diastereodivergence by overriding substrate bias through minimal modification of a single catalyst scaffold.

In conjunction with our study on the development of chiral *P*-spiro iminophosphorane-catalysed regio-, diastereo- and enantioselective conjugate addition reactions, we herein report a complete inversion of diastereoselectivity in the 1,6-addition^33^,^34^,^35^,^36^,^37^,^38^,^39^,^40^,^41^,^42^,^43^,^44^ of azlactones (oxazol-5(4*H*)-ones) to δ-aryl dienyl carbonyl compounds enabled by the slight structural alteration of iminophosphorane catalyst **1** (Fig. 1)^37^,^45^,^46^,^47^,^48^,^49^. Since the minimal change in catalyst structure allows the preservation of other selectivity values in this bond connection between two prochiral reactants with two reaction sites, the present diastereodivergent catalysis provides access to two distinct isomers out of 2^4^ possibly generated isomers, even without considering the newly generated olefin geometry, with almost perfect fidelity. The synthetic utility of this protocol is also demonstrated by the facile derivatization of diastereomeric 1,6-adducts to various α-tetrasubstituted α-amino acids, such as multiply functionalized proline derivatives. In addition, the origin of the unique diastereodivergence is discussed based on the structural elucidation of aminophosphonium ions, conjugate acids of the iminophosphorane catalysts, and computational analysis of the transition-state structures.

## Results

### Initial finding and optimization of the reaction conditions

Our study originated from observing that the regio- and stereoselectivity of the L-leucine-derived iminophosphorane-catalysed conjugate addition of azlactones **2** to δ-aryl dienyl *N*-acylpyrroles **3** were sensitive to water content in the reaction medium. For instance, the treatment of azlactone **2a** with **3a** in the presence of iminophosphorane **1aa** (5 mol%) in dehydrated toluene under an argon atmosphere at 0 °C afforded a mixture of addition products, including desired 1,6-adduct **4aa**, with variable selectivities (a representative result is shown in Table 1, entry 1). On the other hand, attempting the reaction in the presence of powdered molecular sieves 4Å (MS4A) under otherwise identical conditions led to a substantial decrease in regio- and enantioselectivity (entry 2). These results prompted us to employ wet toluene (w-toluene; saturated with water) as a solvent to exploit the potentially positive effect of water on the selectivity profile without experimental inconsistency. As expected, a notable enhancement in both regio- and enantioselectivity was attained in a reproducible manner (entry 3). Moreover, lowering the reaction temperature to −30 °C delivered a significant improvement in diastereoselectivity with complete regio- and enantiocontrol, suggesting the intervention of a structurally defined chiral aminophosphonium/azlactone enolate ion pair incorporating water molecules in the transition state (entry 4). We then evaluated the impact of catalyst structure on diastereocontrol. Interestingly, while using L-valine-derived iminophosphorane **1ba** diminished all selectivities, the totally opposite diastereochemical preference was observed with comparable degree of other selectivity values, when L-phenylalanine-derived **1ca** was applied (entries 5 and 6). In addition, L-isoleucine-derived **1da** showed a similar tendency, affording major diastereomer *RS*-**4aa** ((4*R*,1′*S*)-oxazol-5(4*H*)-one) with a higher enantiomeric excess (entry 7). Noteworthy is that D-*allo*-isoleucine-derived iminophosphorane *ent*-**1ea**, which is a diastereomer of **1da**, behaved almost as an enantiomer of the valine-derived **1ba**, revealing the critical importance of central chirality at the branching carbon in the aliphatic side chain of **1da** for inducing the inversion of diastereoselectivity (entry 5 versus 8). This surprising finding clearly indicated the possibility of establishing an efficient diastereodivergent catalysis in this conjugate addition reaction that entails multiple selectivity control. Since **1aa**, bearing the isobutyl groups, turned out to be the most selective catalyst for producing *RR*-**4aa** ((4*R*,1′*R*)-oxazol-5(4*H*)-one), we first undertook modification of its geminal aromatic substituents and found that introducing 4-fluorophenyl groups further enhanced the diastereoselectivity (entries 9 and 10). The subsequent solvent screening revealed that chlorinated solvents were beneficial, and stereochemically pure *RR*-**4aa** was isolated in 88% yield by performing the reaction in wet 1,2-dichloroethane (w-DCE) under similar conditions (entries 11 and 12). To achieve complete diastereodivergence with the present single catalyst scaffold, we next adopted L-isoleucine-derived iminophosphorane **1da** as a lead catalyst because of its high enantiofacial discriminating capability. Importantly, exclusion of water was crucial in shaping each selectivity value in this case (entry 13), and slight structural manipulation of the aromatic substituents allowed us to identify **1dc**, possessing 4-tolyl groups, as the optimal catalyst for obtaining *RS*-**4aa** with immaculate stereochemical integrity (entries 14 and 15).

### Investigation of substrate scope

Having established the strictly diastereodivergent asymmetric 1,6-addition of azlactones **2** to δ-aryl dienyl *N*-acylpyrroles **3** through minimal adjustment of the structure of chiral iminophosphorane **1** and the reaction solvent, the scope of each optimal catalytic system was surveyed independently (Tables 2 and 3). Under catalysis of **1ab** in w-DCE, the reaction of azlactone **2a** tolerated a wide variety of **3**, containing terminal aromatic substituents of different electronic properties, as an acceptor, giving 1,6-adduct *RR*-**4** in high chemical yield with excellent regio-, diastereo- and enantioselectivity (Table 2, entries 1–10), whereas the *ortho*-substituted aryl appendage appeared to cause an erosion in regio- and diastereoselectivity (entry 11). Heteroaryl-substituted dienyl *N*-acylpyrroles were also amenable to this conjugate addition protocol (entries 12 and 13). Not only δ-aromatic but also δ-alkynyl dienyl acylpyrrole **3n** were well accommodated to this system to afford desired 1,6-adduct *RR*-**4an** in 92% yield with high stereoselectivity (entry 14). An array of azlactones **2**, bearing different α-side chains of amino acid origin, were employed as enolate precursors, although a certain decrease in diastereoselectivity was detected for those derived from aliphatic amino acids (entries 15–18). On the other hand, subjecting the same series of substrate combinations to catalysis of **1dc** with MS4A in toluene revealed the efficiency and robustness of the diastereodivergent asymmetric catalysis, providing *RS*-**4** with high stereochemical purity (Table 3, entries 1–18). It should be noted that the permutations of either dienes with δ-heteroaryl or alkynyl substituents (**3l**–**3n**) as an acceptor, or leucine-derived azlactone **2b** as an enolate precursor, caused a slight loss in diastereoselectivity (entries 12–15). The absolute configurations of both diastereomers were unambiguously determined as *RR* and *RS* by single crystal X-ray diffraction analysis (Supplementary Figs 101 and 102), and the stereochemistry of other examples was assumed by analogy.

**Figure 2:**
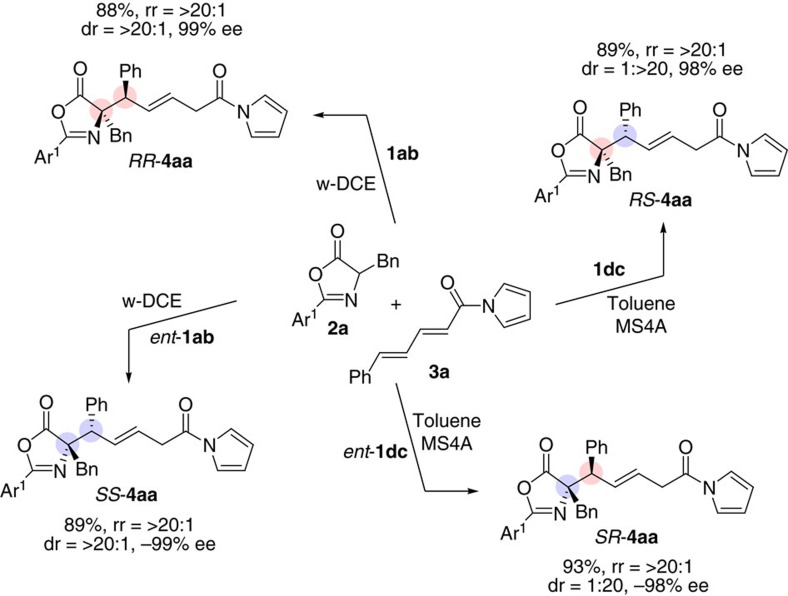
Stereodivergent synthesis of all stereoisomers of 4aa (Ar^1^=2,6-(MeO)_2_C_6_H_3_). All reactions were performed with 5 mol % of **1** in the indicated solvent at −30 °C for 16 h.

We then prepared enantiomeric catalysts, *ent*-**1ab** and *ent*-**1dc**, from the parent D-amino acids to demonstrate the accessibility of the full array of stereoisomers of 1,6-adduct **4aa**. Using each iminophosphorane catalyst system, all four stereoisomers of **4aa** were obtained from the same set of starting materials, **2a** and **3a**, in high yields with virtually complete regio-, enantio- and diastereoselectivity as illustrated in Fig. 2.

**Figure 3:**
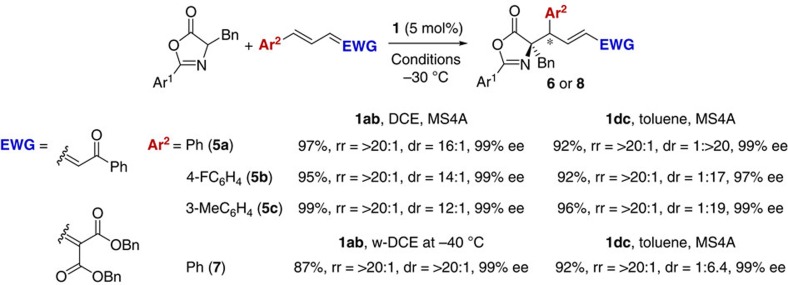
Diastereodivergence in 1,6-selective conjugate addition to δ-aryl dienyl carbonyl compounds (Ar^1^=2,6-(MeO)_2_C_6_H_3_). Unless otherwise noted, all reactions were conducted under the optimized reaction conditions for 1,6-addition to δ-aryl dienyl acylpyrroles **3**. Absolute stereochemistry of *RR*-**6ac** was unambiguously determined by X-ray diffraction analysis (Supplementary Fig. 105).

The efficient diastereodivergent catalysis of iminophosphoranes **1ab** and **1dc** could further be extended to the reactions with other δ-aryl dienyl carbonyl compounds (Fig. 3). For example, in the conjugate addition of **2a** to vinylogous chalcone derivative **5a**, near complete inversion of diastereoselectivity was accomplished with full preservation of other selectivity values by the proper use of **1ab** or **1dc** as a catalyst, giving rise to 1,6-adducts *RR*-**6aa** and *RS*-**6aa** of high stereochemical purity, respectively. It was of interest that dehydrated condition was beneficial in both catalytic systems for this substrate combination, which clearly demonstrates that the slight structural difference between iminophosphoranes **1ab** and **1dc** plays a pivotal role in inducing the rigorous diastereodivergence. Similar reactivity and selectivity were attained in the reactions of **2a** with **5**, bearing different aromatic substituents at the δ-position, implying general applicability of this unique stereodivergent protocol. Moreover, vinylidene malonate **7** also appeared to be a good candidate as an acceptor for the addition of **2a** under the catalysis of either **1ab** or **1dc**.

### Transformation of 4 into α-amino acid derivatives

**Figure 4:**
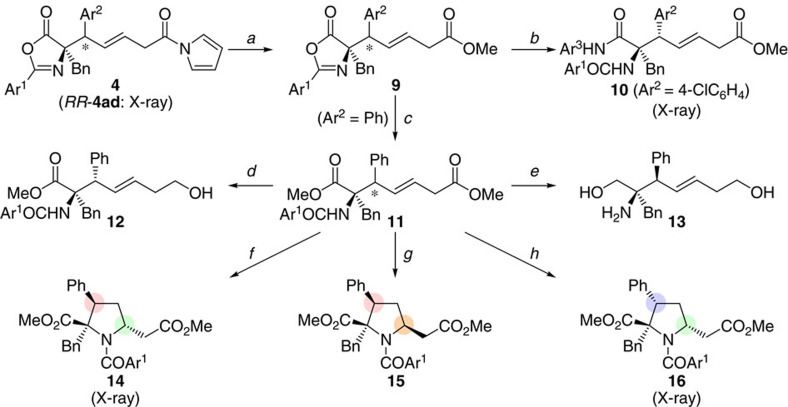
Conversion to various α-tetrasubstituted α-amino acid derivatives. Conditions (Ar^1^=2,6-(MeO)_2_C_6_H_3_): (a) NaOMe, MeOH/CH_2_Cl_2_, 0 °C, 81% (from *RS*-**4ac**); 91% (from *RR*-**4aa**); 83% (from *RS*-**4aa**), (b) BF_3_·OEt_2_, 4-ClC_6_H_4_NH_2_ (Ar^3^NH_2_), toluene, 100 °C, 56% (from *RS*-**9ac**), (c) SOCl_2_, MeOH, 5 °C, 83% (from *RR*-**9aa**); 69% (from *RS*-**9aa**), (d) LiBH_4_, THF, rt, 95% (from *RS*-**11**), (e) (i) LiAlH_4_, Et_2_O, reflux, (ii) KOH, aqueous MeOH, 150 °C (MW), 78% (2 steps from *RR*-**11**), (f) PtCl_2_, Cs_2_CO_3_, DMF, 140 °C, 76% (pure diastereomer, see Supplementary Fig. 103) (from *RR*-**11**), (g) DBU, MeOH, 40 °C, 54% (pure diastereomer) (from *RR*-**11**), (h) DBU, MeOH, 60 °C, 88% (diastereomeric mixture, dr=14:1, see Supplementary Fig. 104 for the absolute stereochemistry of major isomer) (from *RS*-**11**).

Conjugate adduct **4** can be converted into a series of functionalized α-amino acid derivatives (Fig. 4)^50^,^51^. Harnessing the structural feature of **4**, possessing two differentiated functionalities of carboxylic acid oxidation state, the acyl pyrrole unit was selectively transformed into methyl ester by treatment with sodium methoxide in methanol^52^. The azlactone ring of resultant ester **9** was cleaved by reaction with either 4-chloroaniline in the presence of Lewis acid, to give amide **10**, or with thionyl chloride in methanol, to furnish diester **11**. Selective reduction of the sterically more accessible ester group in **11** was feasible, and thus protected α-allylic α-amino acid **12** was isolated in 95% yield. The complete reduction of the two terminal ester moieties and subsequent removal of the nitrogen protecting group by methanolic potassium hydroxide under microwave (MW) irradiation afforded corresponding amino diol **13** in 78% yield (two steps). Moreover, the olefin component of diester **11** was utilized as a functional handle to increase the molecular complexity in a stereodivergent manner by taking advantage of the availability of two diastereomers, *RR*- and *RS*-**11**, in an essentially pure form. For example, aza-platination of *RR*-**11**, followed by protodeplatination, resulted in the diastereoselective production of functionalized proline derivative **14** via 5-*endo* cyclization. In contrast, diastereomeric **15** was preferably formed by treating *RR*-**11** with 1,8-diazabicyclo[5.4.0]undec-7-ene (DBU) in methanol, presumably through an olefin isomerization-aza-conjugate addition sequence^36^. Upon exposure of *RS*-**11** to similar basic conditions, another diastereomer **16** was obtained in 88% yield with a diastereomeric ratio of 14:1.

## Discussion

**Figure 5:**
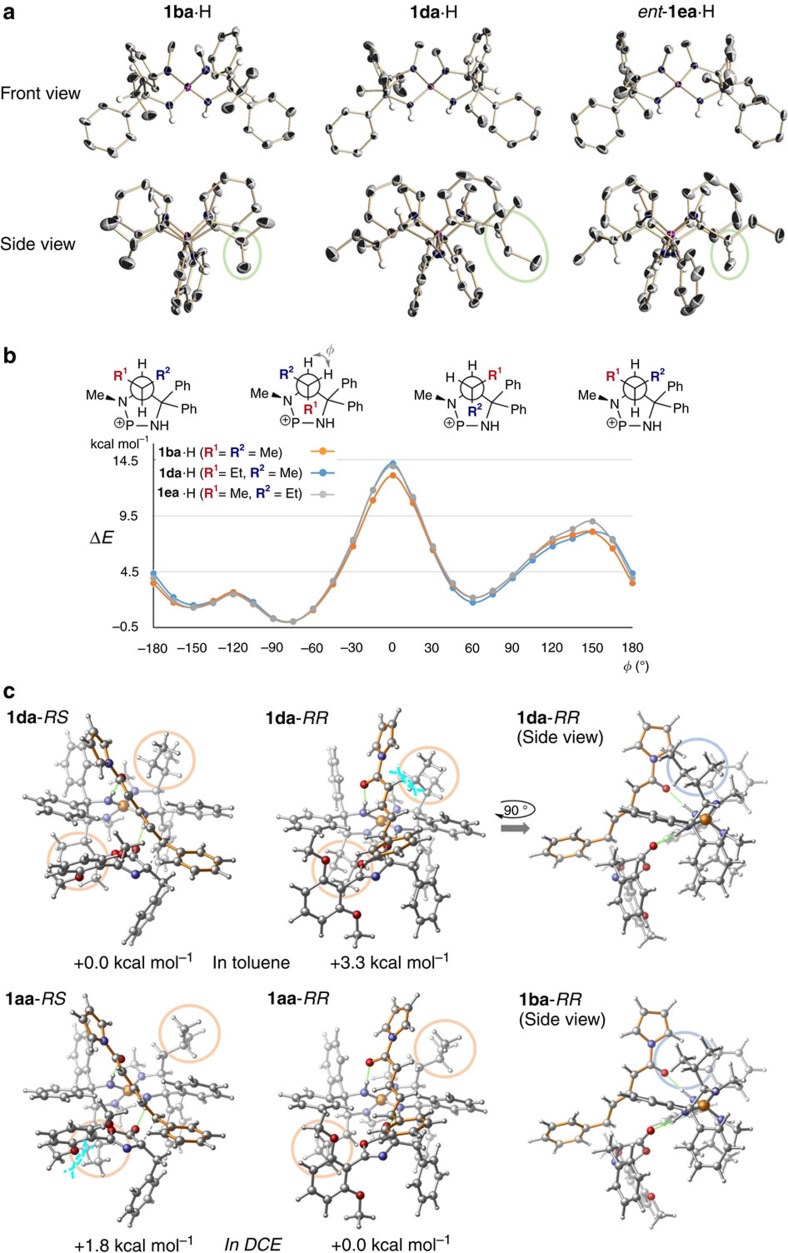
Structural elucidation of aminophosphonium ions 1·H and computational analysis of transition-state structures of the diastereodivergent 1,6-addition reactions. (**a**) ORTEP diagrams of aminophosphonium ions **1**·H. (**b**) Energy profile along with rotation around C–C axis of the stereogenic carbon of **1**·H and the branching carbon of alkyl side chain. (**c**) Transition-state structure models for the C–C bond-forming step from DFT calculation and relative Gibbs free energies at PCM-B3LYP-D3/6-311++G**//B3LYP/6-31G* level.

The observed complete inversion of diastereoselectivity most likely stems from the unique structural feature of L-isoleucine(Ile)-derived iminophosphorane catalysts such as **1da**, compared with L-valine(Val)- and D-*allo*-isoleucine(Allo)-derived **1ba** and *ent*-**1ea**. Therefore, to clarify the difference in the structures of the conjugate acids of these iminophosphoranes that possess α-branched aliphatic side chains, namely, aminophosphonium ions **1ba**·H, **1da**·H and *ent*-**1ea**·H, their molecular architectures were visualized using a single crystal X-ray diffraction analysis (Fig. 5a; Supplementary Figs 106–109). While three-dimensional structures created by [5.5]-spirocyclic core and aromatic substituents are nearly identical, α-amino acid-derived aliphatic substituents cause notable structural difference in each aminophosphonium ion. Dihedral angles (*φ*) between C-H bond on the stereogenic centre of the diazaphosphacycles and C-H bond on the branching carbons of the aliphatic substituents were determined to be −59.3, −82.2° for **1ba**·H, −73.6, −156° for **1da**·H and 67.9, 147.2° for *ent*-**1ea**·H, respectively. Because it was uncertain whether the unsymmetrical disposition of two aliphatic substituents of **1da** and *ent*-**1ea** reflected the most stable structures of these aminophosphonium ions or it was induced by different effects, such as a crystal packing, energy profile associated with the rotation around the axis of the C–C bond was analysed by the density functional theory (DFT) calculation at B3LYP/6-31G* level (signs of dihedral angles for *ent*-**1ea**·H were inverted for comparison with the others)^53^. As shown in Fig. 5b, the most preferable *φ* is around −75° and the second stable conformer is formed around −150° in all three cations as a consequence of minimizing steric repulsion between α-branched aliphatic substituent and anchimeric phenyl group. These results indicate that the aminophosphonium ions adopt one of the possible energetically favourable conformers in the solid state. In solution state, *φ* of the H–C–C–H component is expected to totter between *ca.* −75 and −150° and one of the two alkyl groups attached on the branching carbon of the aliphatic substituent would be directed toward the chiral cavity constructed by the aromatic substituents. While methyl groups of Val- and Allo-derived aminophosphonium ions **1ba**·H and *ent*-**1ea**·H overhang the cavity, the bulkier ethyl group is dangling in the case of Ile-derived cation **1da**·H due to the (*S*)-configuration at the branching carbon of *sec*-butyl substituent, which may induce different diastereochemical outcome. To gain insight into the transition-state (TS) structures, further analysis was pursued *in silico*. Experimentally observed linear relationship between enantiomeric purity of the iminophosphorane catalysts and 1,6-addition products (Supplementary Figs 110 and 111), and the quantum mechanistic study on the related iminophosphorane catalysis suggested the participation of a single chiral aminophosphonium ion to the stereodetermining C–C bond-forming step of the conjugate addition reaction^54^,^55^. Most likely, the aminophosphonium ion featuring two N–H protons accommodates the two nucleophilic and electrophilic substrates in a way that one N–H proton makes hydrogen bond with the azlactone enolate and the other with the carbonyl of the *N*-acylpyrrole to organize a macrocyclic transition-state structure, thereby enabling rigorous control of multiple selectivity factors. On the basis of this presupposition, we designed and generated the macrocyclic transition-state models of the reaction between **2a** and **3a** with Ile-derived **1da** as a catalyst for DFT calculation. Initially, relatively stable TSs that afforded each diastereomer were selected from 32 possible TSs (B3LYP/6-31G*) and the energy of the TS was estimated by calculation at B3LYP-D3/6-311++G** level with solvent and temperature parameters (PCM, toluene at −30 °C) (Fig. 5c and see also Supplementary Data 1)^56^. The resulting energy profile qualitatively reproduced experimental selectivities in the **1da**-catalysed reaction (Supplementary Table 1, entry 7). From structural viewpoint, entire form of aminophosphonium residues in all TSs is very similar, but positions of ethyl groups are variable depending on the orientations of the two substrates captured via hydrogen-bonding interactions, while *φ* of the H–C–C–H component fall within −80.7 and −152°. In the most stable TS giving the major *RS*-isomer, the steric demand imposed by one ethyl group regulates the placement of **3a** that lies down into the chiral cavity and expose the *si* face to the enolate of **2a**. The other ethyl group is situated as to avoid inducing steric repulsion with the substituent of **2a** and is directed toward phenyl group across the aminophosphonium core of **1da**·H. The steric effect caused by the position of the ethyl group appears crucial for attaining high diastereoselectivity because it contributes to destabilize the TS leading to the minor *RR*-isomer, which is 3.3 kcal mol^−1^ higher in energy. Importantly, the steric constraint between the ethyl group and the *N*-acylpyrrole moiety of **3a** is relieved in the reaction with Val-derived catalyst **1ba** to an extent enough for the TS giving *RR*-isomer to be stabilized to constitute a major stereochemical pathway, as evident from the side view of the TS structure in comparison to that of the TS with Ile-derived catalyst **1da** (90° clockwise rotation from **1da**-*RR* around the vertical axis). These results are consistent with our analysis based on the solid state structures of the aminophosphonium ions obtained from the X-ray diffraction measurements and account for the reversal of diastereochemical preference between the reactions with **1ba** and **1da** as catalysts, respectively. With Leu-derived catalyst **1aa**, lack of α-branching in the isobutyl substituents allows for the accommodation of **3a** without obvious steric congestion. Instead, steric collision of one of the isobutyl groups with the substituent of **2a** contributes to make the TS affording *RS*-isomer 1.8 kcal mol^−1^ higher in energy than the most stable, stereodetermining TS, in which **3a** is tilted toward the isobutyl group, having the *re* face available for the attack of the enolate of **2a**. We assume that this TS structure leading to *RR*-isomer could be slightly modified by the intervention of water molecules, which would be critical to form even more stable, well-defined macrocyclic TS. The overall elucidation reveals that the ability of the aminophosphonium ion with two hydrogen-bonding donor sites embedded in the rigid structural core to simultaneously accommodate the two reacting substrates is essential for organizing the macrocyclic TS, wherein small difference in the catalyst substituents, aliphatic substituents, in particular, plays a key role in altering the orientation of the substrates, especially the dienyl carbonyl electrophile, for achieving the strict diastereodivergence without affecting the prominent regio- and enantioselectivity.

In summary, we achieved near perfect diastereodivergence in asymmetric 1,6-addition reactions of azlactones to δ-aryl dienyl carbonyl compounds with consistently excellent levels of regio- and enantiocontrol, primarily through minimal modification of chiral iminophosphorane catalysts. Using each optimal iminophosphorane catalyst and its enantiomer under appropriate conditions enabled access to the full complement of stereoisomers of the structurally diverse 1,6-adduct. The synthetic utility of the method was highlighted by the facile preparation of various α-tetrasubstituted α-amino acids, including densely functionalized proline derivatives. Furthermore, the origin of the diastereodivergence was elucidated by the experimental and computational analysis of the catalyst behaviour and transition-state structures. We anticipate that the reported salient feature of the iminophosphorane catalysis in multiple selectivity control will find fruitful applications, and further studies in this line are currently underway.

## Methods

### Representative procedure for 1,6-addition with **1ab** as a catalyst

To a solution of azlactone **2a** (155.67 mg, 0.50 mmol) and dienyl *N*-acylpyrrole **3a** (55.82 mg, 0.25 mmol) in 1,2-dichloroethane saturated with water (w-DCE, 1.05 ml) was added a solution of chiral iminophosphorane **1ab** (8.50 mg, 12.5 μmol) in DCE (0.20 ml) at −30 °C under Ar atmosphere. The resulting mixture was stirred for 16 h and the reaction was then quenched by the addition of a solution of trifluoroacetic acid in toluene (0.5 M, 125 μl). All volatiles were removed by evaporation to give crude residue, which was analysed by ^1^H NMR (800 MHz) to determine the regioisomeric and diastereomeric ratios of adducts. The subsequent purification by column chromatography on silica gel (hexane (H)/ethyl acetate (EA)=1:1 as eluent) gave adducts in 88% yield as a mixture of isomers (117.6 mg, 0.22 mmol). The enantiomeric excess of 1,6-adduct **4aa** was determined by HPLC analysis. *RR*-**4aa** (4 *R*,1′*R*): HPLC OZ3, H/EtOH=10:1, flow rate=1 ml min^−1^, *λ*=210 nm, 16.5 min (minor diastereomer), 18.9 min (minor diastereomer), 20.5 min (minor isomer of major diastereomer), 30.1 min (major isomer of major diastereomer); ^1^H NMR (400 MHz, CDCl_3_) *δ* 7.38 (2H, dd, *J*=8.2, 1.4 Hz), 7.32–7.14 (11H, m), 6.42 (2H, d, *J*=8.4 Hz), 6.39 (1H, ddt, *J*=15.5, 9.8, 1.3 Hz), 6.27 (2H, t, *J*=2.3 Hz), 6.02 (1H, dt, *J*=15.5, 7 Hz), 3.98 (1H, d, *J*=9.8 Hz), 3.72 (1H, ddd, *J*=17.4, 7, 1.3 Hz), 3.65 (1H, ddd, *J*=17.4, 7, 1.3 Hz), 3.58 (6H, s), 3.44 (1H, d, *J*=13.7 Hz), 3.17 (1H, d, *J*=13.7 Hz); ^13^C NMR (101 MHz, CDCl_3_) *δ* 179, 168.4, 159.4, 158.1, 138, 134.6, 132.9, 132.8, 130.9, 129.6, 128.3, 128, 127.4, 126.9, 125.8, 119.2, 113.4, 105.1, 103.5, 78.2, 56, 55.9, 42.9, 38.4; IR (film): 3,030, 2,936, 2,839, 1,805, 1,717, 1,663, 1,595, 1,476, 1,331, 1,300, 1,256, 1,115, 964, 918 cm^–1^; HRMS (ESI) Calcd for C_33_H_31_N_2_O_5_ ([M+H]^+^) 535.2227. Found 535.2226.

### Representative procedure for 1,6-addition with **1dc** as a catalyst

A test tube was charged with a magnetic stirrer bar and molecular sieves 4 Å (MS4A, 100 mg) under argon atmosphere. MS4A was then dried with a heat gun under reduced pressure for 5 min and the test tube was refilled with Ar. Azlactone **2a** (155.67 mg, 0.50 mmol) and dienyl *N*-acylpyrrole **3a** (55.82 mg, 0.25 mmol) were added and dissolved in toluene (1.05 ml). A solution of chiral iminophosphorane **1dc** (8.31 mg, 12.5 μmol) in toluene (0.20 ml) was added dropwise at −30 °C. After being stirred for 16 h, a solution of trifluoroacetic acid in toluene (0.5 M, 125 μl) was introduced to the reaction mixture to quench the reaction. The whole mixture was passed through a pad of Celite with the aid of toluene to remove MS4A and the filtrate was concentrated. The regioisomeric and diastereomeric ratios of adducts were determined by ^1^H NMR analysis (800 MHz) of the crude residue. The subsequent purification by column chromatography on silica gel (H/EA=1:1 as eluent) gave the adducts in 89% yield as a mixture of isomers (119 mg, 0.22 mmol). The enantiomeric excess of 1,6-adduct *RS-***4aa** was determined by HPLC analysis. *RS-***4aa** (4 *R*,1′*S*): HPLC OZ3, H/EtOH=10:1, flow rate=1 ml min^−1^, *λ*=210 nm, 17 min (minor isomer of major diastereomer), 19.3 min (major isomer of major diastereomer), 21.1 min (minor diastereomer), 31.3 min (minor diastereomer); ^1^H NMR (400 MHz, CDCl_3_) *δ* 7.49 (2H, d, *J*=7.4 Hz), 7.36 (2H, t, *J*=7.4 Hz), 7.32 (1H, t, *J*=8.6 Hz), 7.28 (1H, t, *J*=7.4 Hz), 7.22 (2H, brs), 7.21–7.15 (3H, m), 7.14–7.07 (2H, m), 6.48 (2H, d, *J*=8.6 Hz), 6.20 (1H, ddt, *J*=15.3, 9.9, 1.4 Hz), 6.17 (2H, t, *J*=2.3 Hz), 5.90 (1H, dt, *J*=15.3, 6.8 Hz), 3.88 (1H, d, *J*=9.9 Hz), 3.63 (6H, s), 3.61 (1H, ddd, *J*=16.8, 6.8, 1.4 Hz), 3.50 (1H, ddd, *J*=16.8, 6.8, 1.4 Hz), 3.12 (1H, d, *J*=13.7 Hz), 2.89 (1H, d, *J*=13.7 Hz); ^13^C NMR (101 MHz, CDCl_3_) *δ* 180, 168.2, 159.4, 157.5, 138.5, 134.5, 132.9, 132.8, 130.8, 129.4, 128.8, 128, 127.5, 126.8, 125.4, 119.2, 113.3, 105.6, 103.7, 78.5, 56.6, 56, 41.6, 38.8; IR (film): 3,030, 2,938, 2,839, 1,805, 1,717, 1,670, 1,595, 1,476, 1,333, 1,302, 1,256, 1,113, 964, 910 cm^–1^; HRMS (ESI) Calcd for C_33_H_31_N_2_O_5_ ([M+H]^+^) 535.2227. Found 535.2228.

For full characterization data of other new compounds and experimental details, see Supplementary Methods.

For NMR spectra of new compounds in this article, see Supplementary Figs 1–77.

For HPLC traces of the reaction products, see Supplementary Figs 78–100.

### Data availability

X-ray crystallographic data have been deposited in Cambridge Crystallographic Data Centre (http://www.ccdc.cam.ac.uk/) with the accession codes CCDC 1494061, 1494060, 1494058, 1494059, 1520998, 1520999, 1520995, 1520997 and 1520996. All other relevant data are available on request from the authors.

## Additional information

**How to cite this article:** Uraguchi, D. *et al*. Complete diastereodivergence in asymmetric 1,6-addition reactions enabled by minimal modification of a chiral catalyst. *Nat. Commun.* **8,** 14793 doi: 10.1038/ncomms14793 (2017).

**Publisher's note**: Springer Nature remains neutral with regard to jurisdictional claims in published maps and institutional affiliations.

## Supplementary Material

Supplementary Information Supplementary figures, supplementary table, supplementary discussion, supplementary methods and supplementary references.

Supplementary Data 1 Cartesian coordinates of DFT calculations for transition state models.

## Figures and Tables

**Figure 1:**
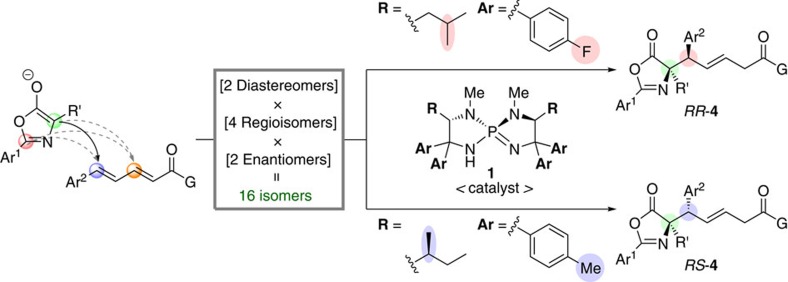
Research overview. Small change in the catalyst structure enables diastereodivergent 1,6-addition of azlactones to δ-aryl dienyl carbonyl compounds.

**Table 1:**
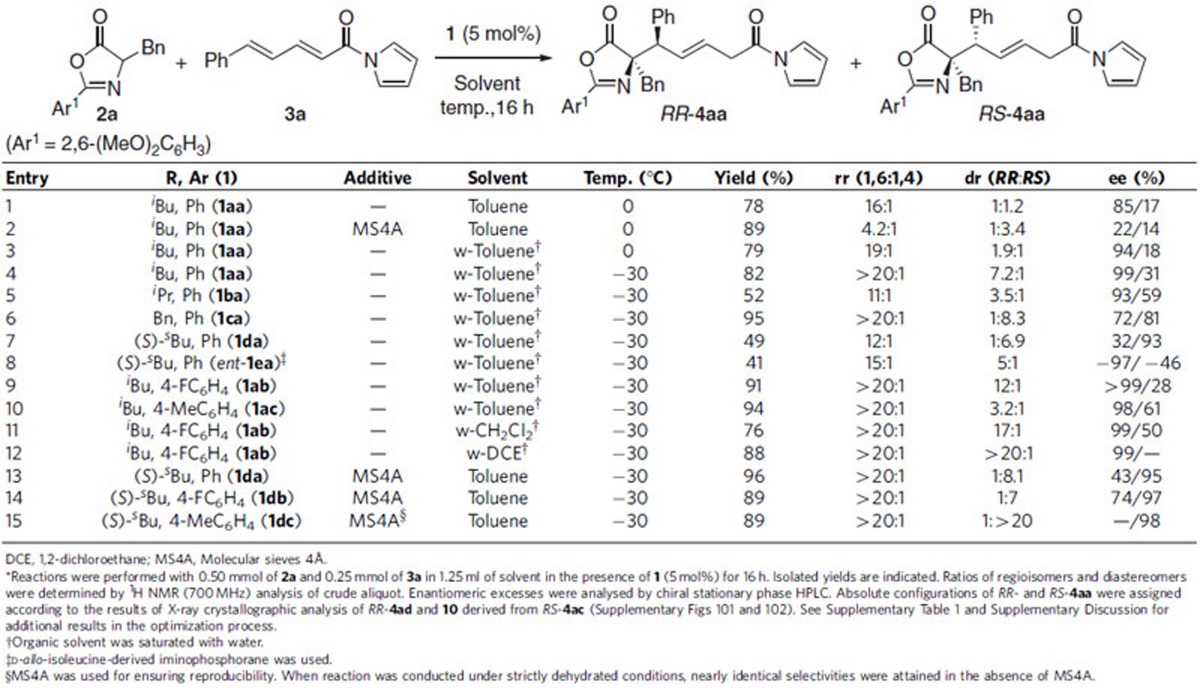
Optimization of reaction parameters*.

**Table 2:**
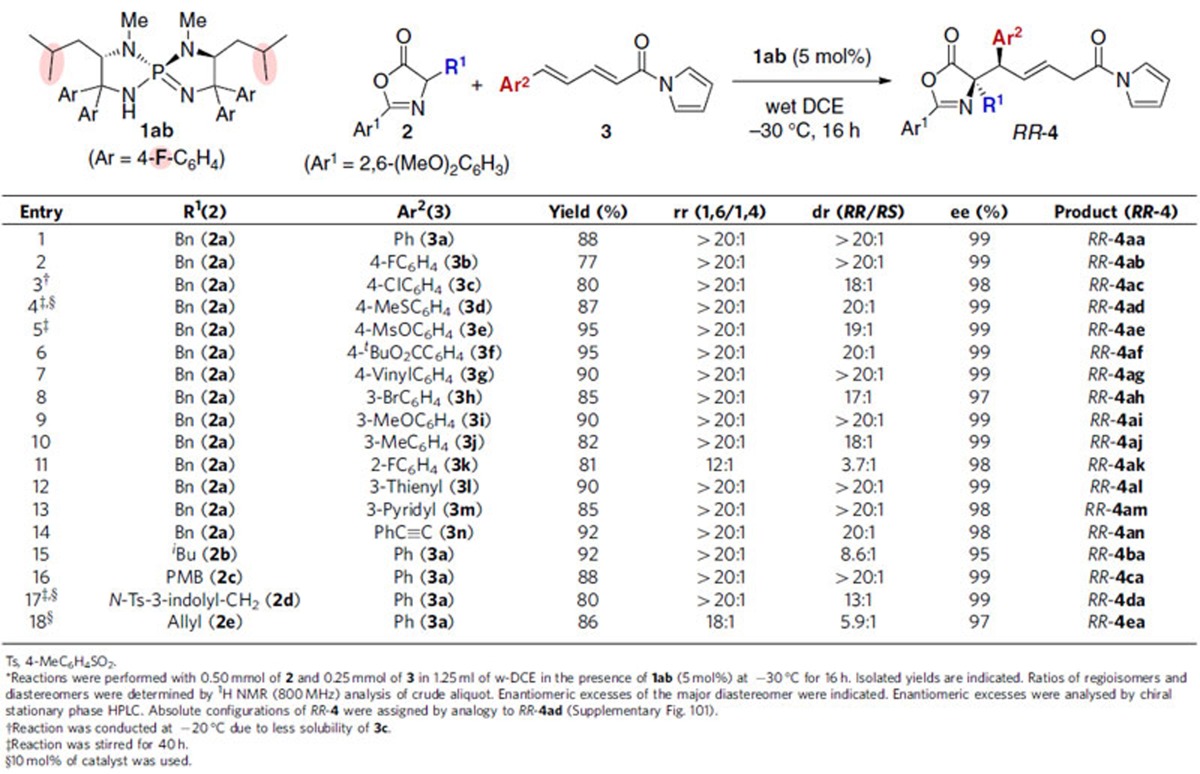
Substrate scope of **1ab**-catalyzed 1,6-addition to δ-aryl dienyl *N*-acylpyrroles 3*.

**Table 3:**
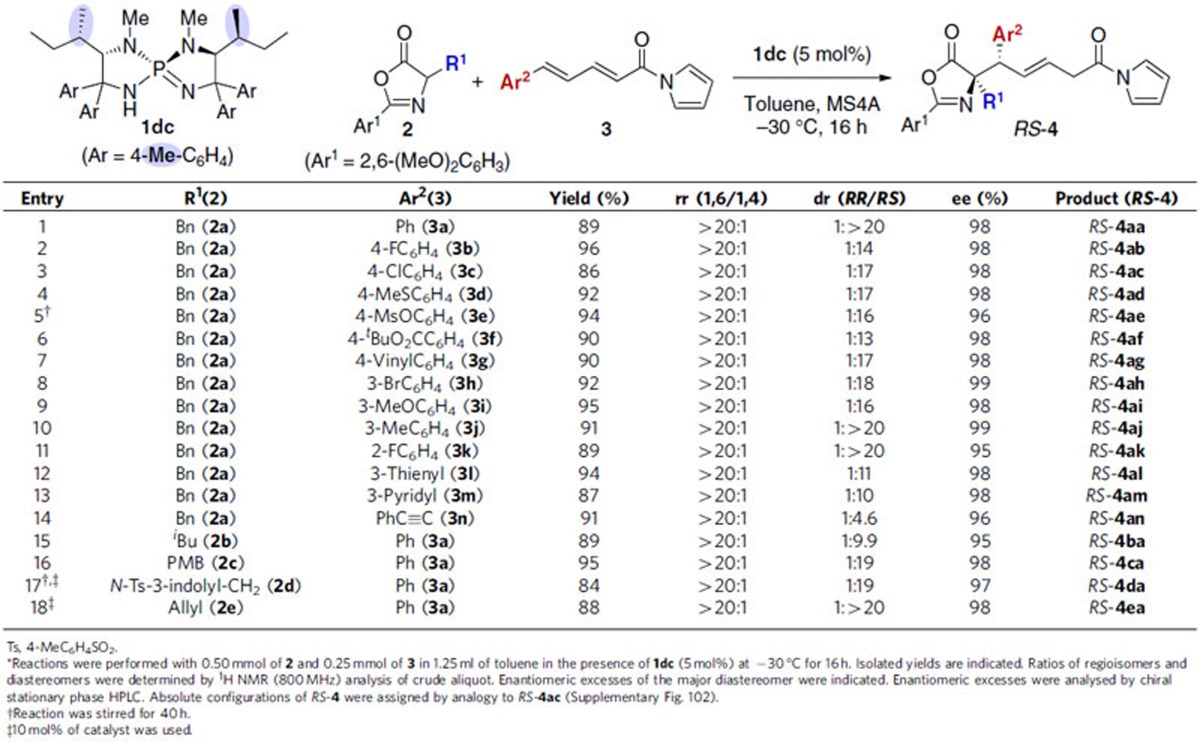
Substrate scope for **1dc**-catalysed 1,6-addition to δ-aryl dienyl *N*-acylpyrroles 3*.
