# Supplementary material for: Complete diastereodivergence in asymmetric 1,6-addition reactions enabled by minimal modification of a chiral catalyst
Source: Nat Commun. 2017 Mar 20;8:14793. doi: 10.1038/ncomms14793 (PMC5364390; doi:10.1038/ncomms14793)
Supplement: Supplementary Information — Supplementary figures, supplementary table, supplementary discussion, supplementary methods and supplementary references. [file ncomms14793-s1.pdf]

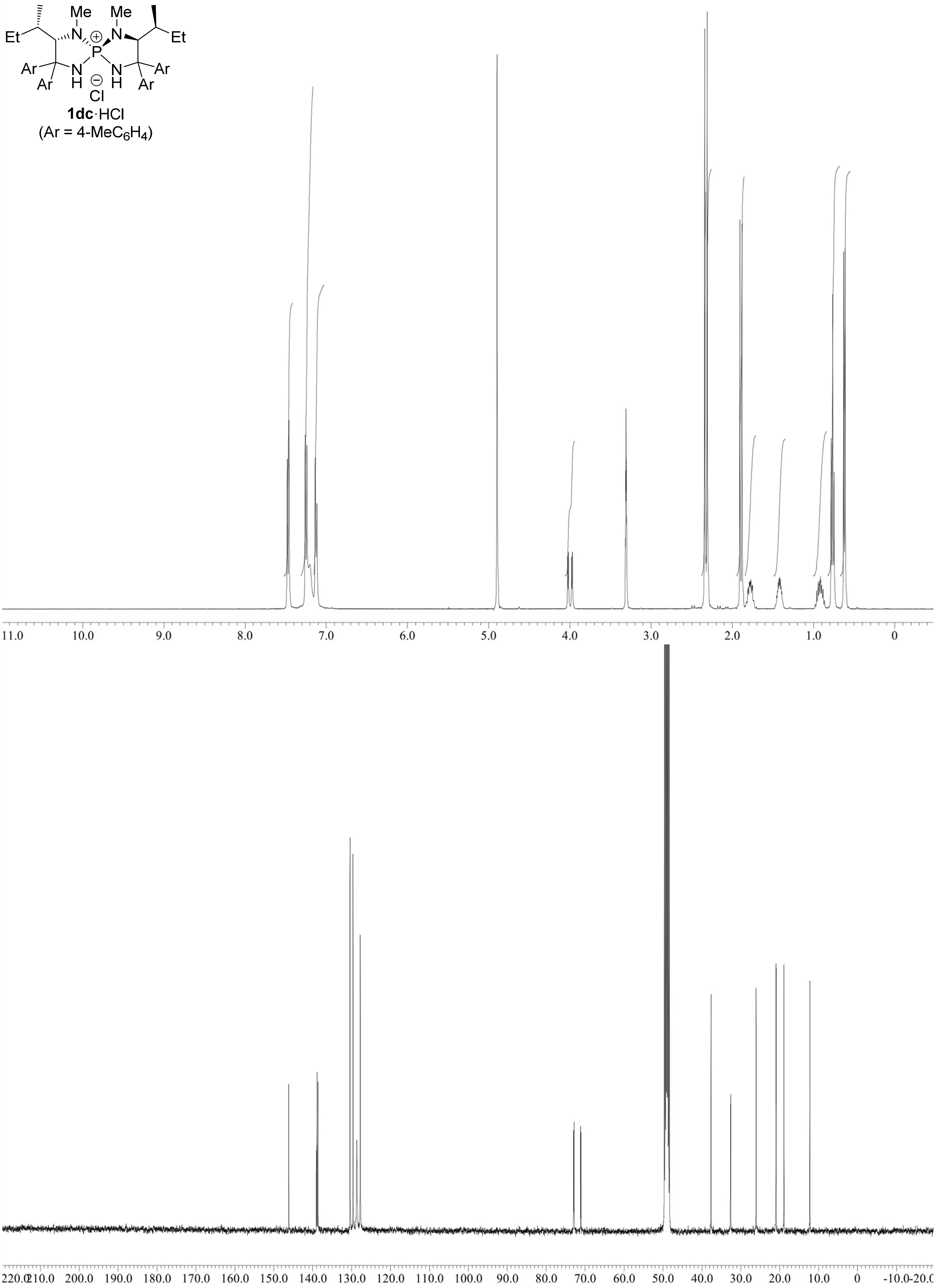

Supplementary Figure 1. ^{1}H and ^{13}C NMR spectra of **1dc**·HCl

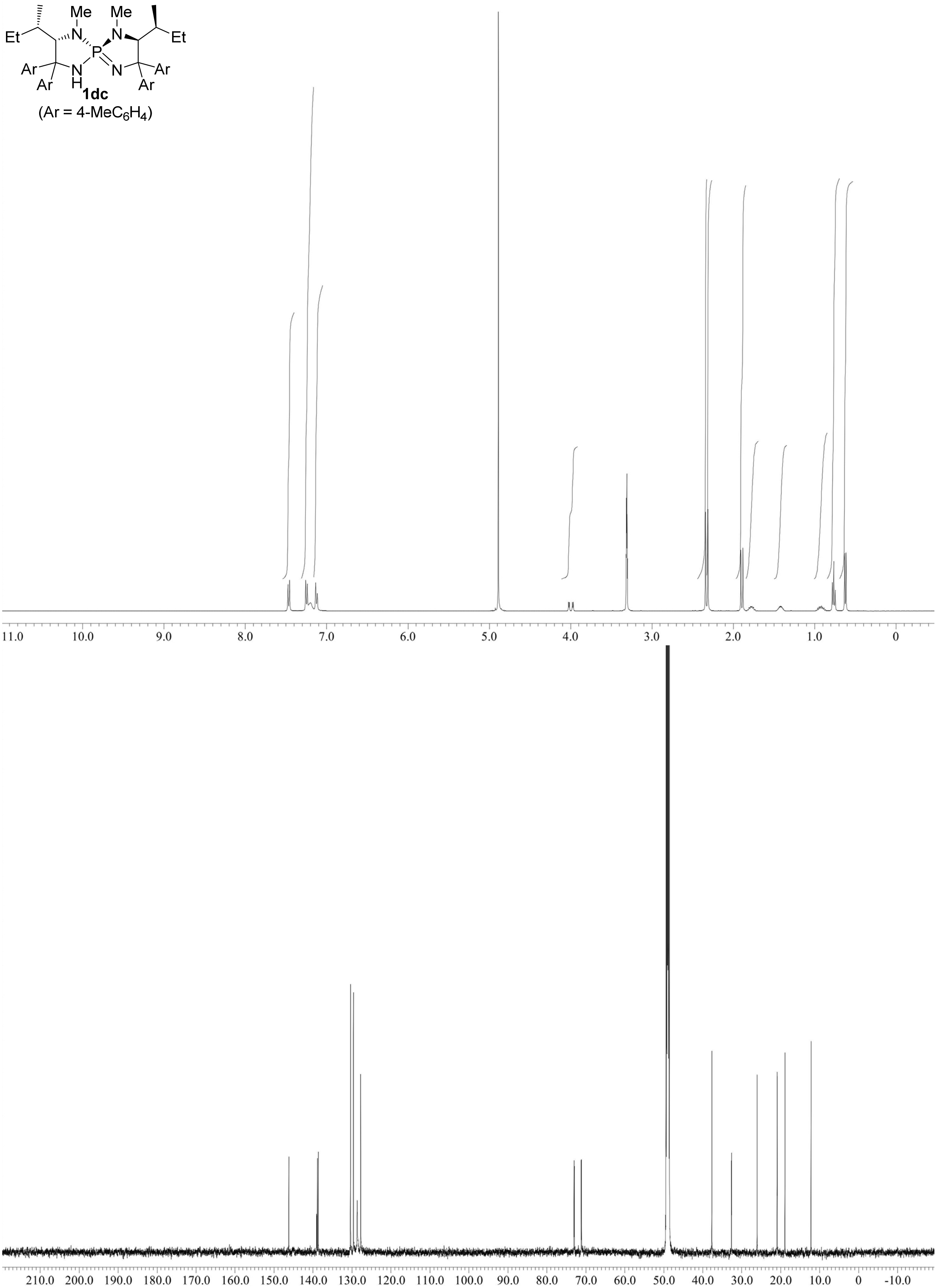

Supplementary Figure 2. ^{1}H and ^{13}C NMR spectra of **1dc**

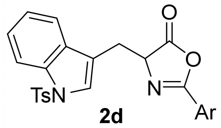

2d

Ar = 2,6-$(MeO)_2C_6H_3$

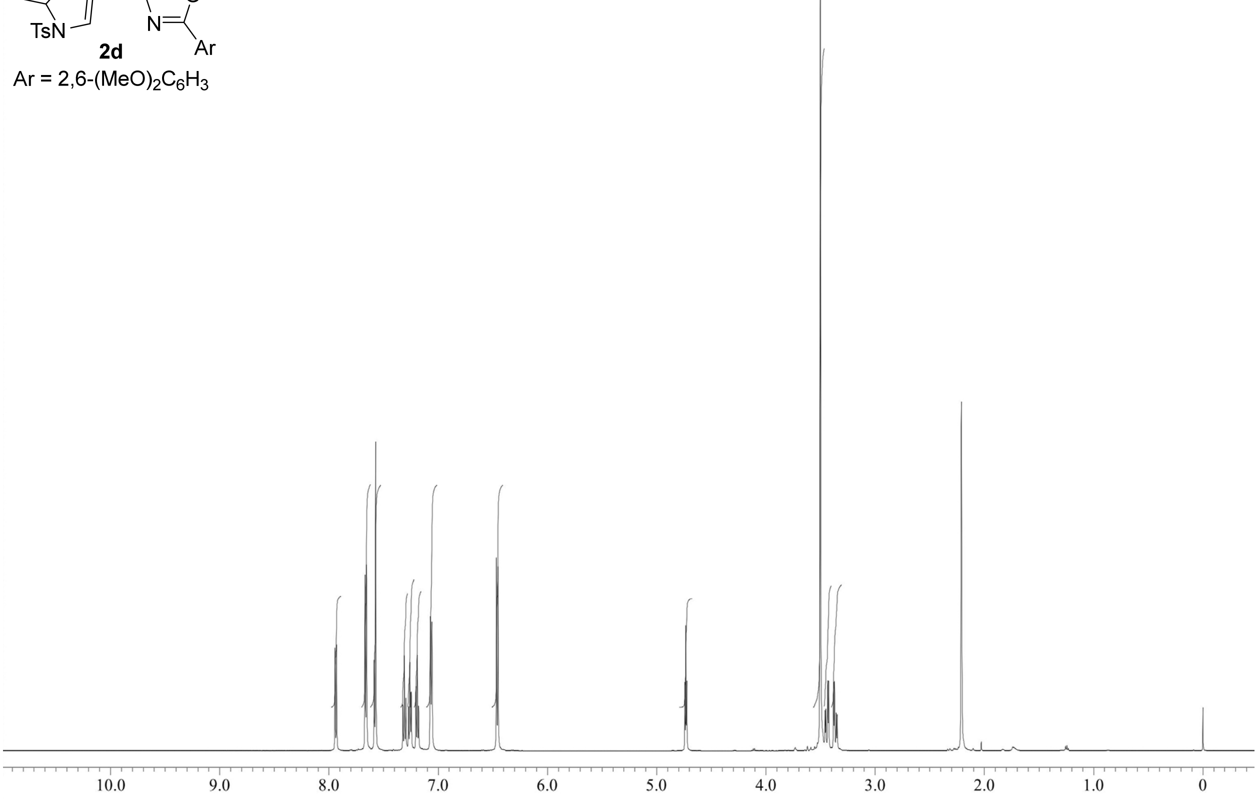

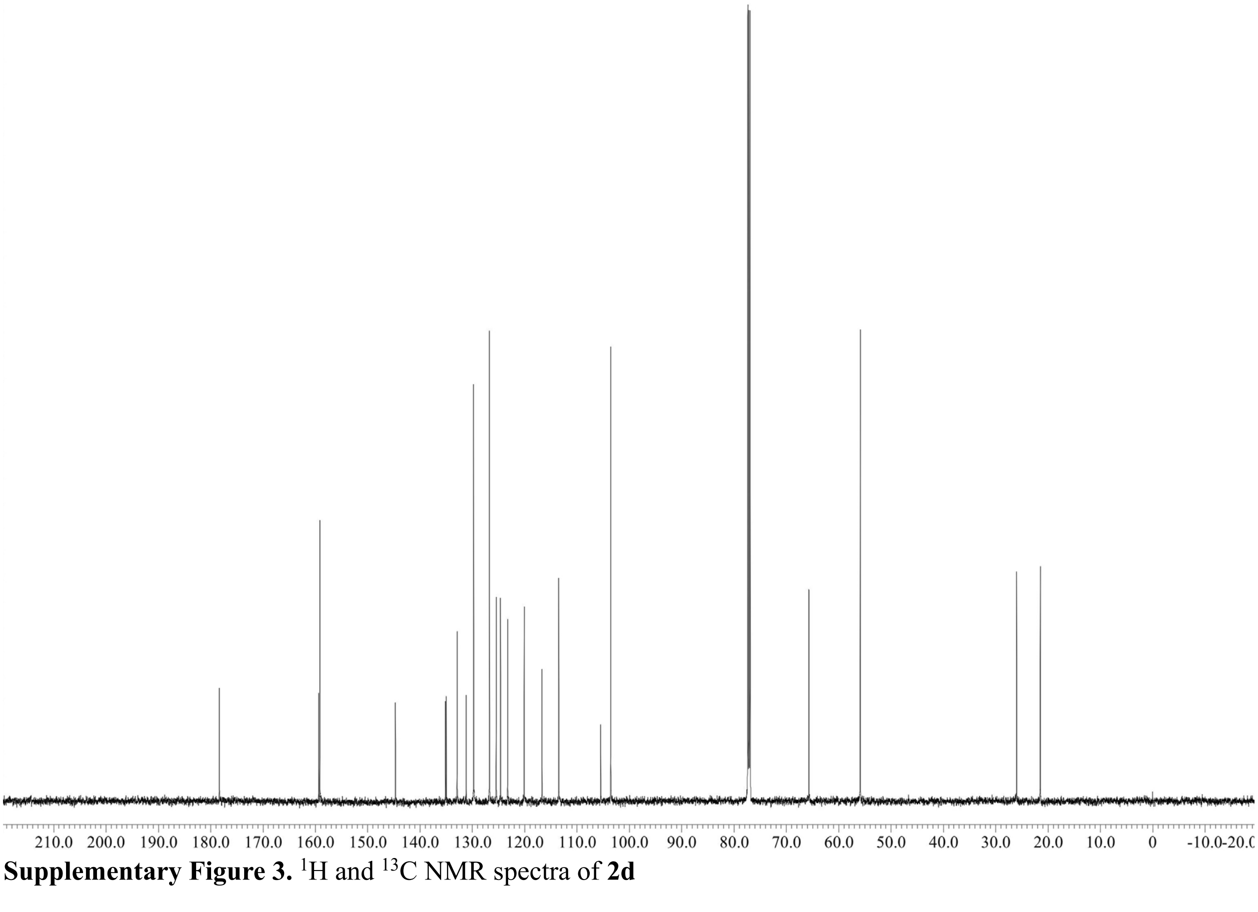

Supplementary Figure 3. ^{1}H and ^{13}C NMR spectra of **2d**

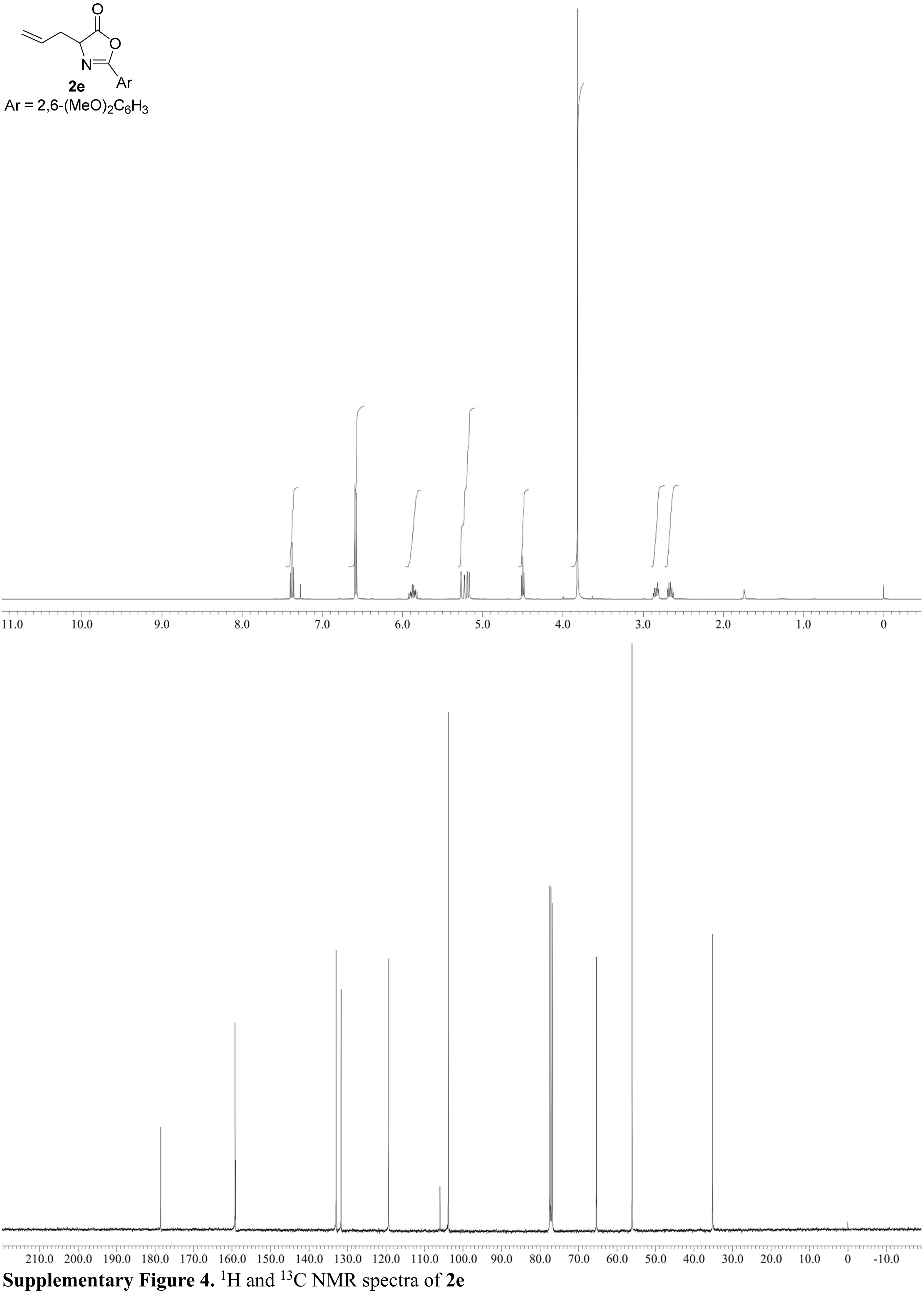

Supplementary Figure 4. ^{1}H and ^{13}C NMR spectra of **2e**

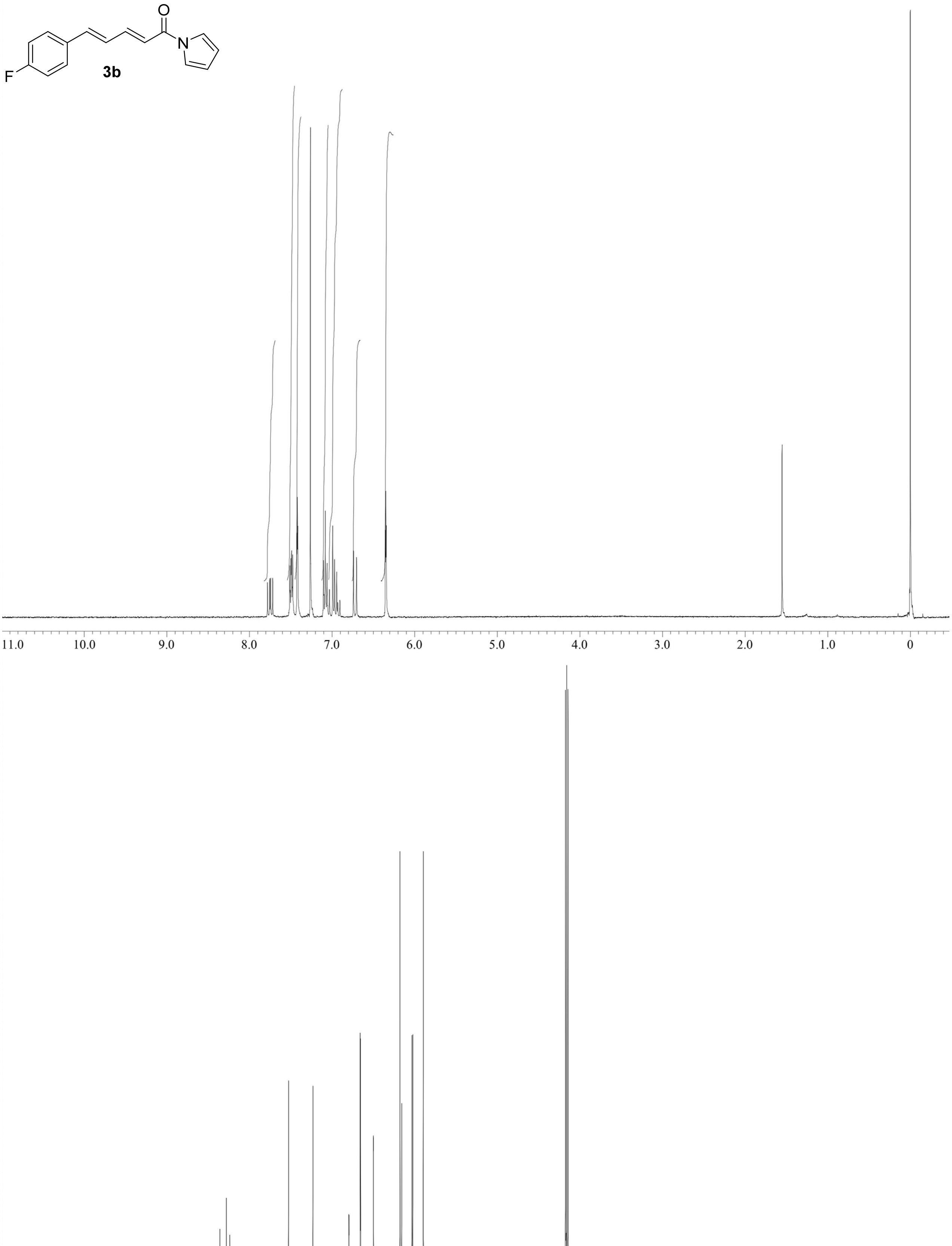

Supplementary Figure 5. ^{1}H and ^{13}C NMR spectra of **3b**

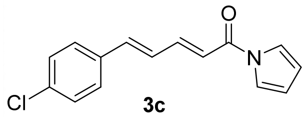

3c

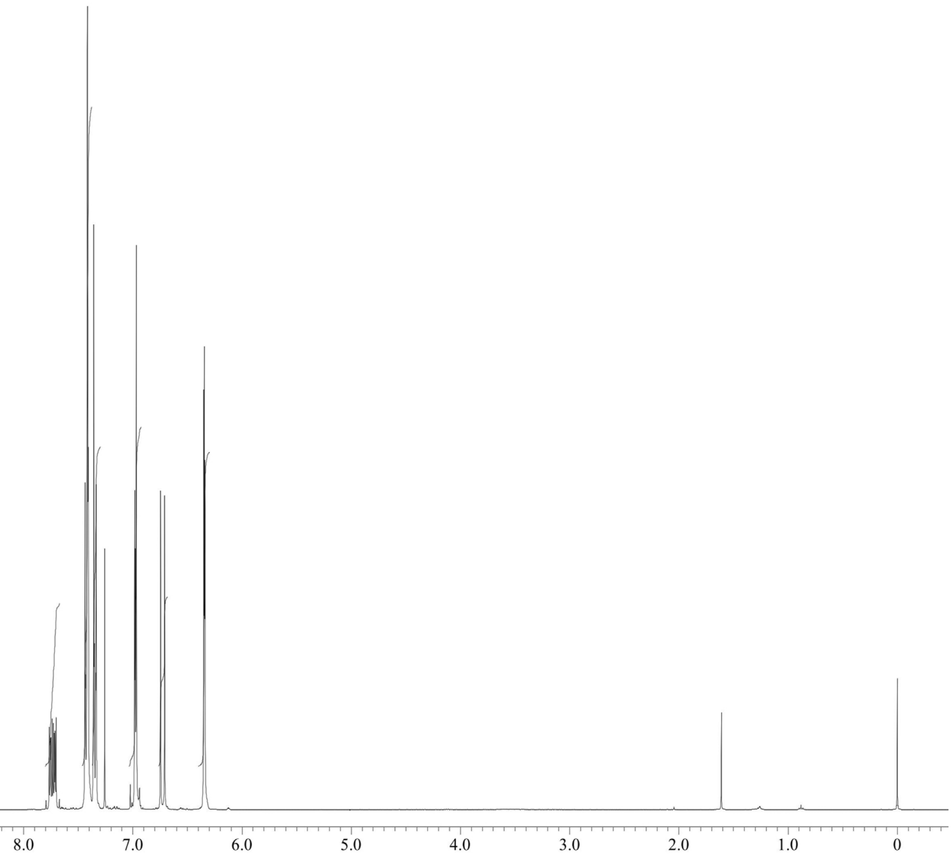

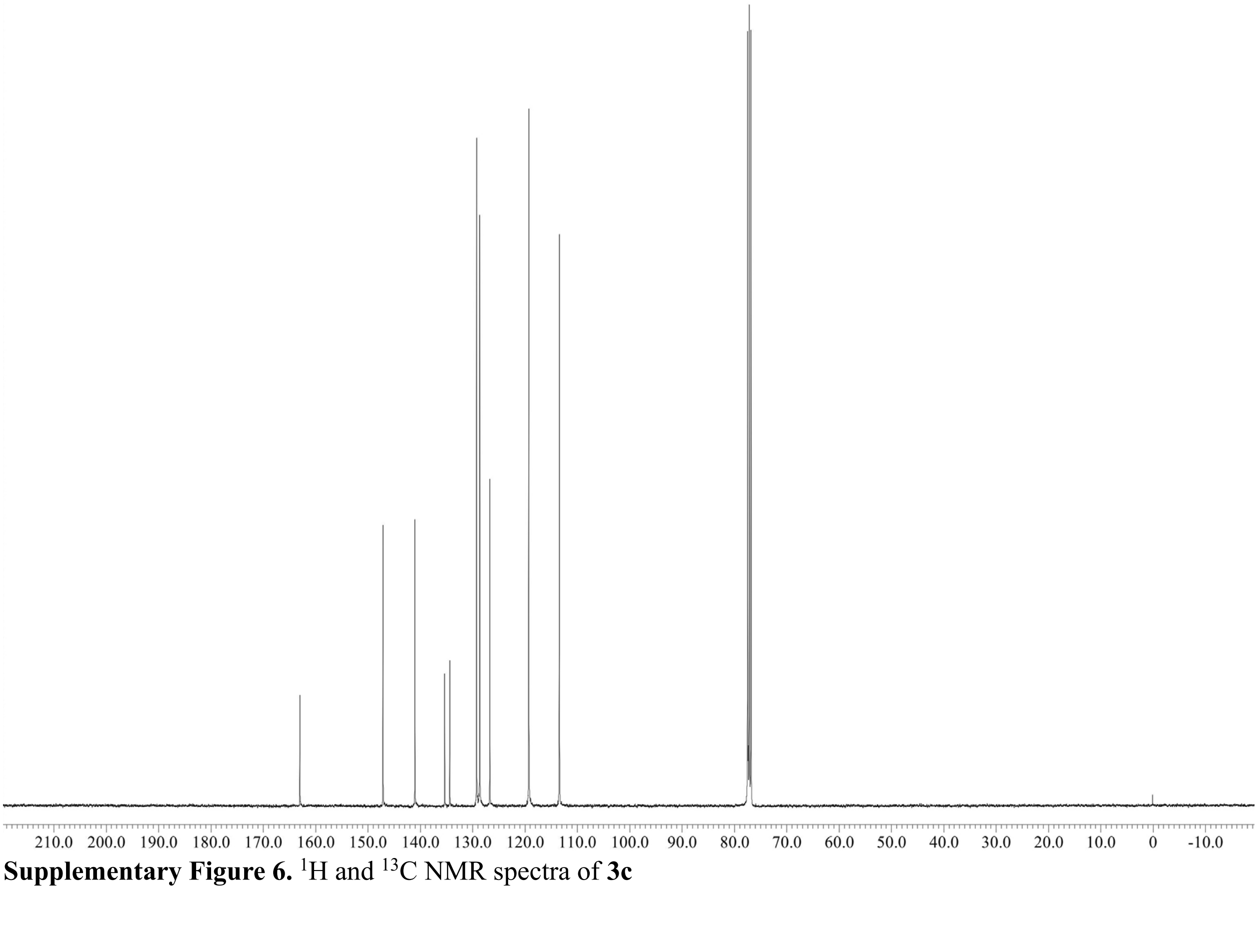

Supplementary Figure 6. ^{1}H and ^{13}C NMR spectra of **3c**

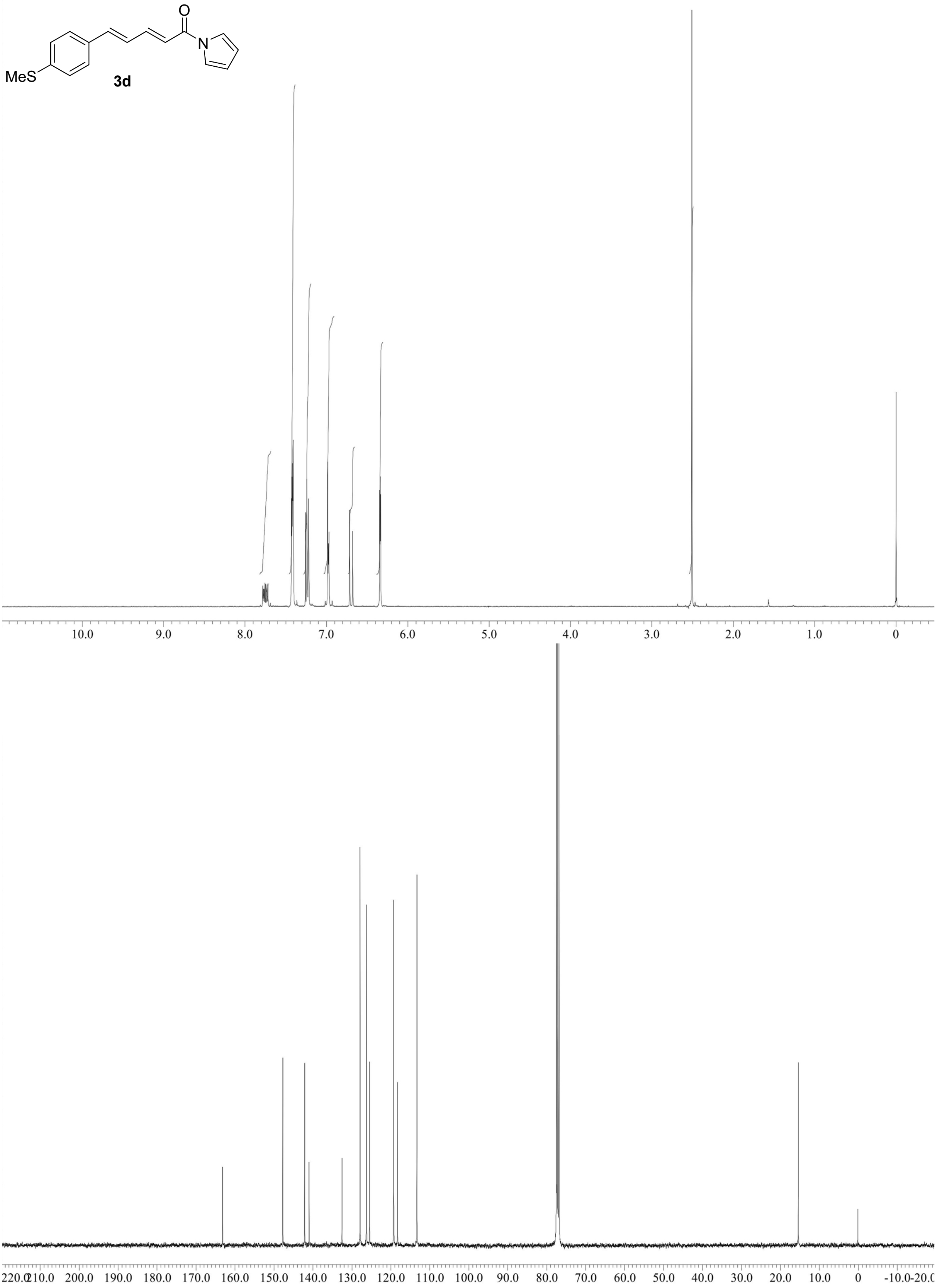

Supplementary Figure 7. ^{1}H and ^{13}C NMR spectra of **3d**

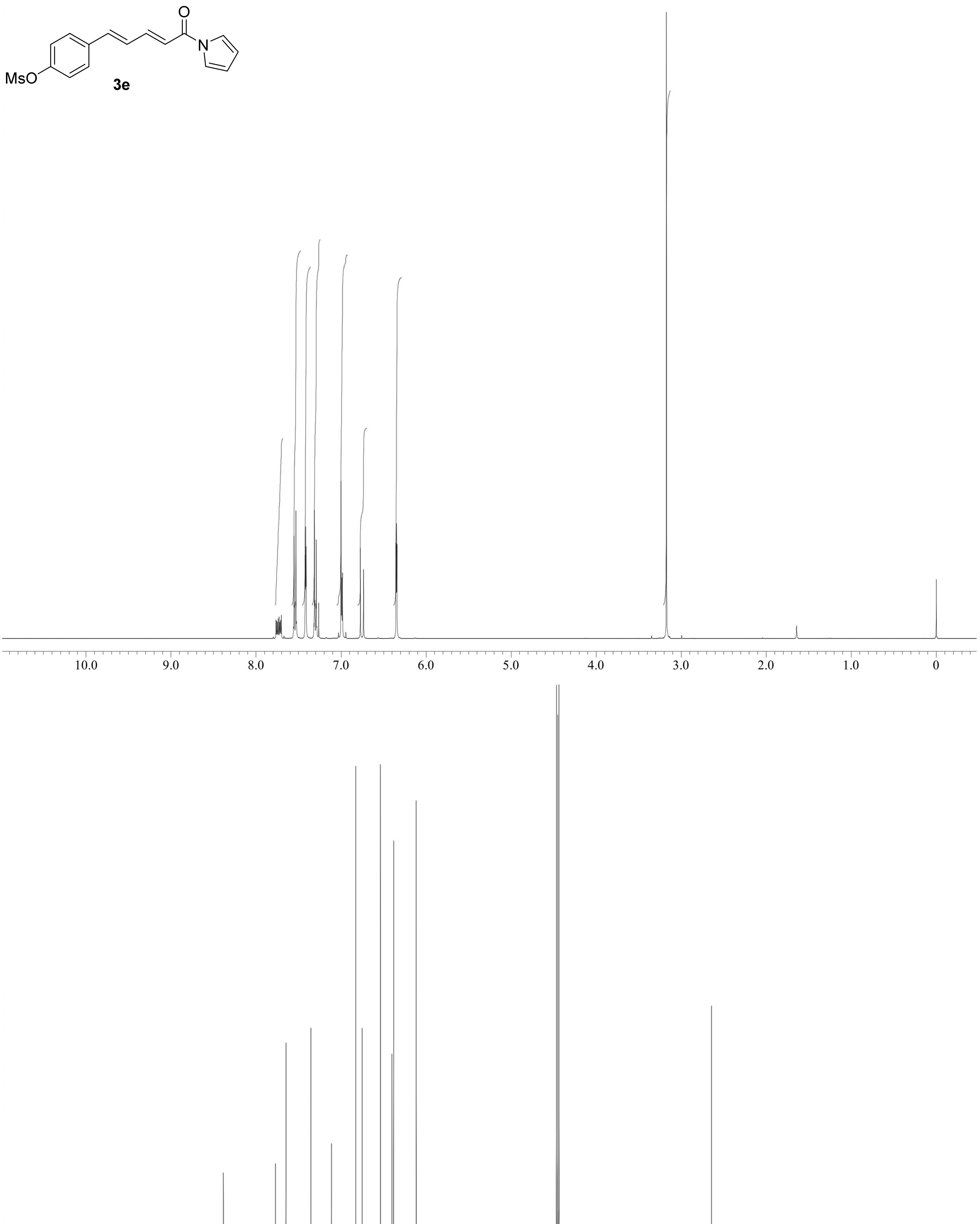

Supplementary Figure 8. ^{1}H and ^{13}C NMR spectra of **3e**

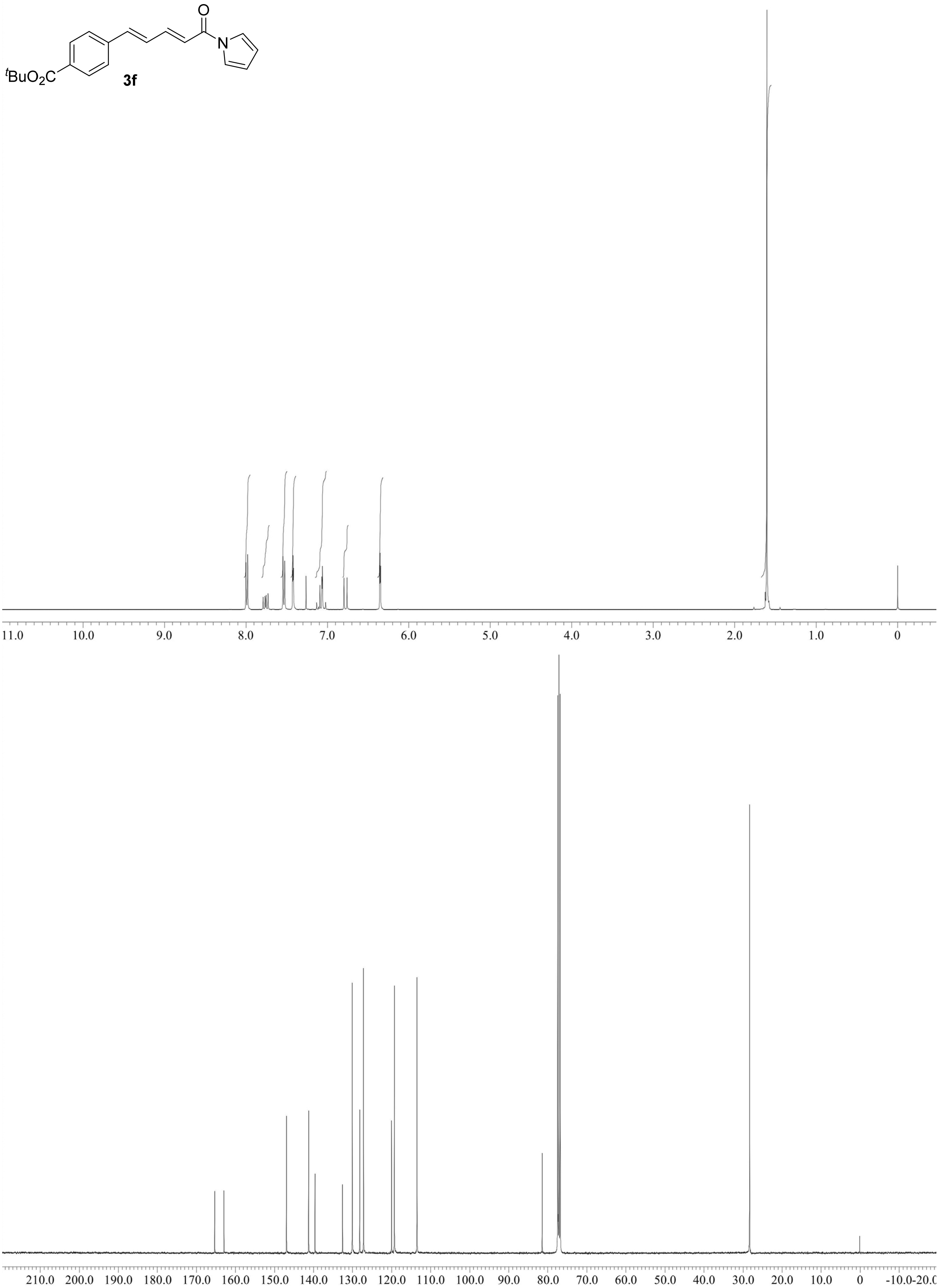

Supplementary Figure 9. ^{1}H and ^{13}C NMR spectra of **3f**

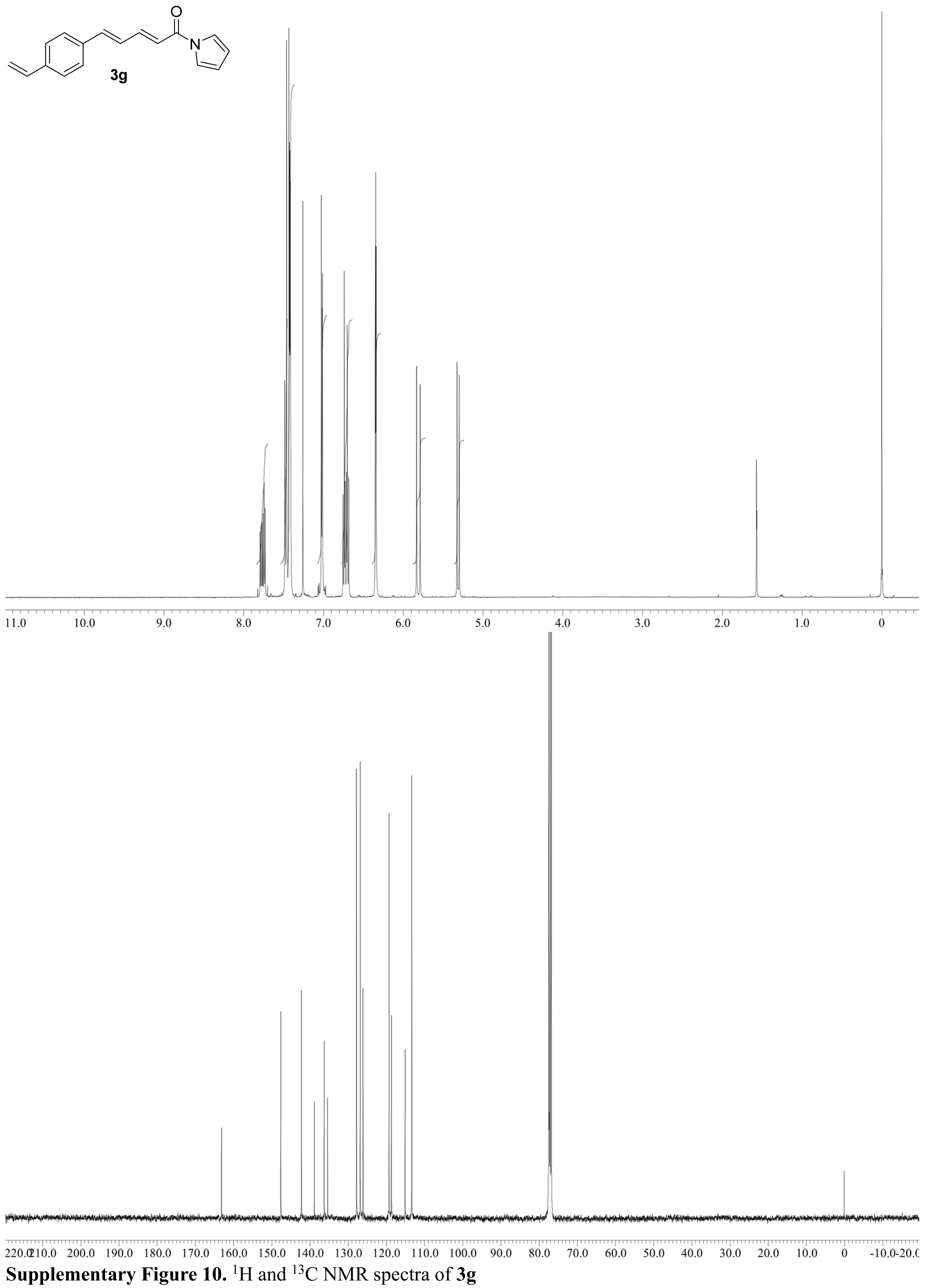

Supplementary Figure 10. ^{1}H and ^{13}C NMR spectra of **3g**

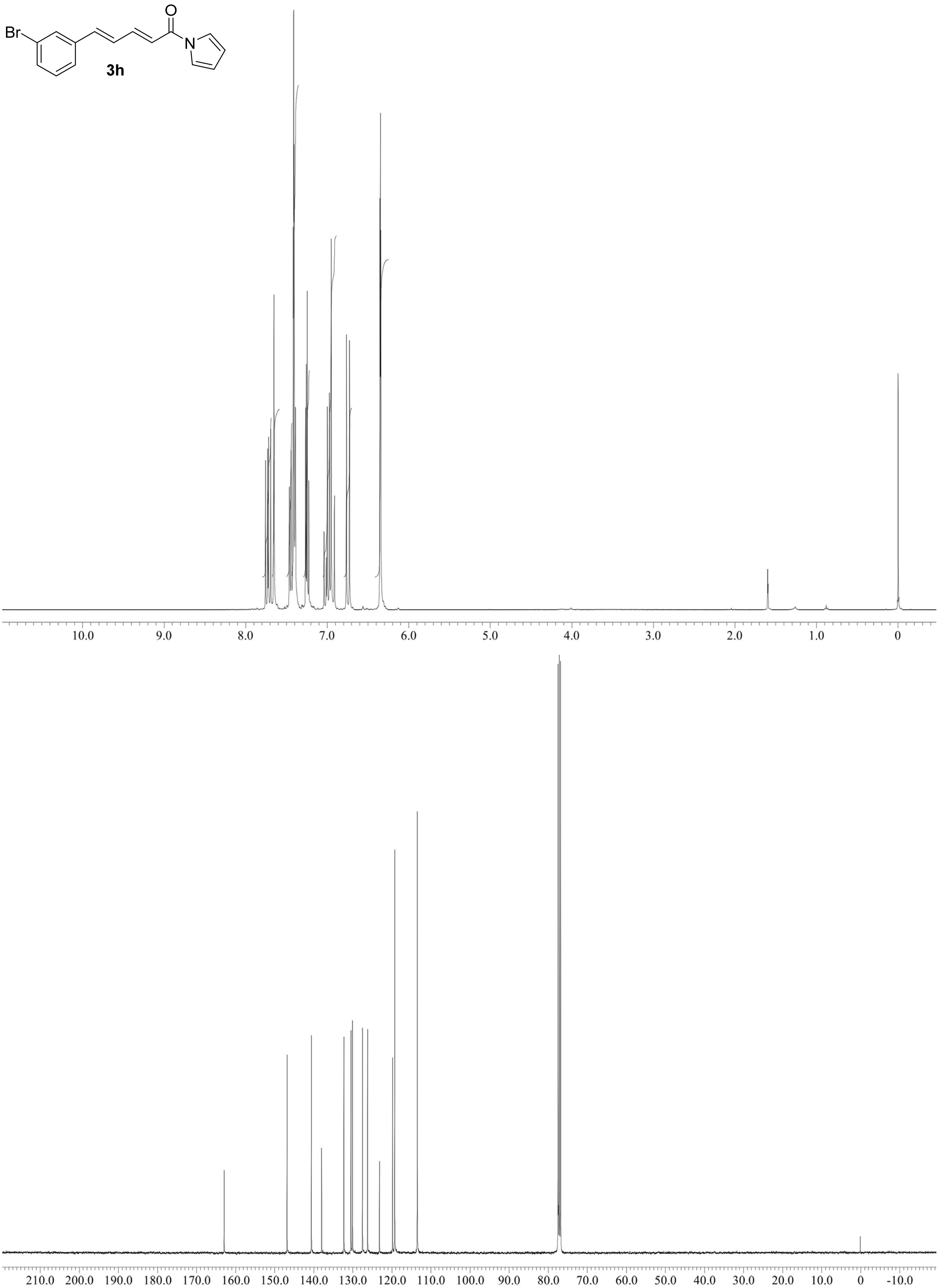

Supplementary Figure 11. ^{1}H and ^{13}C NMR spectra of **3h**

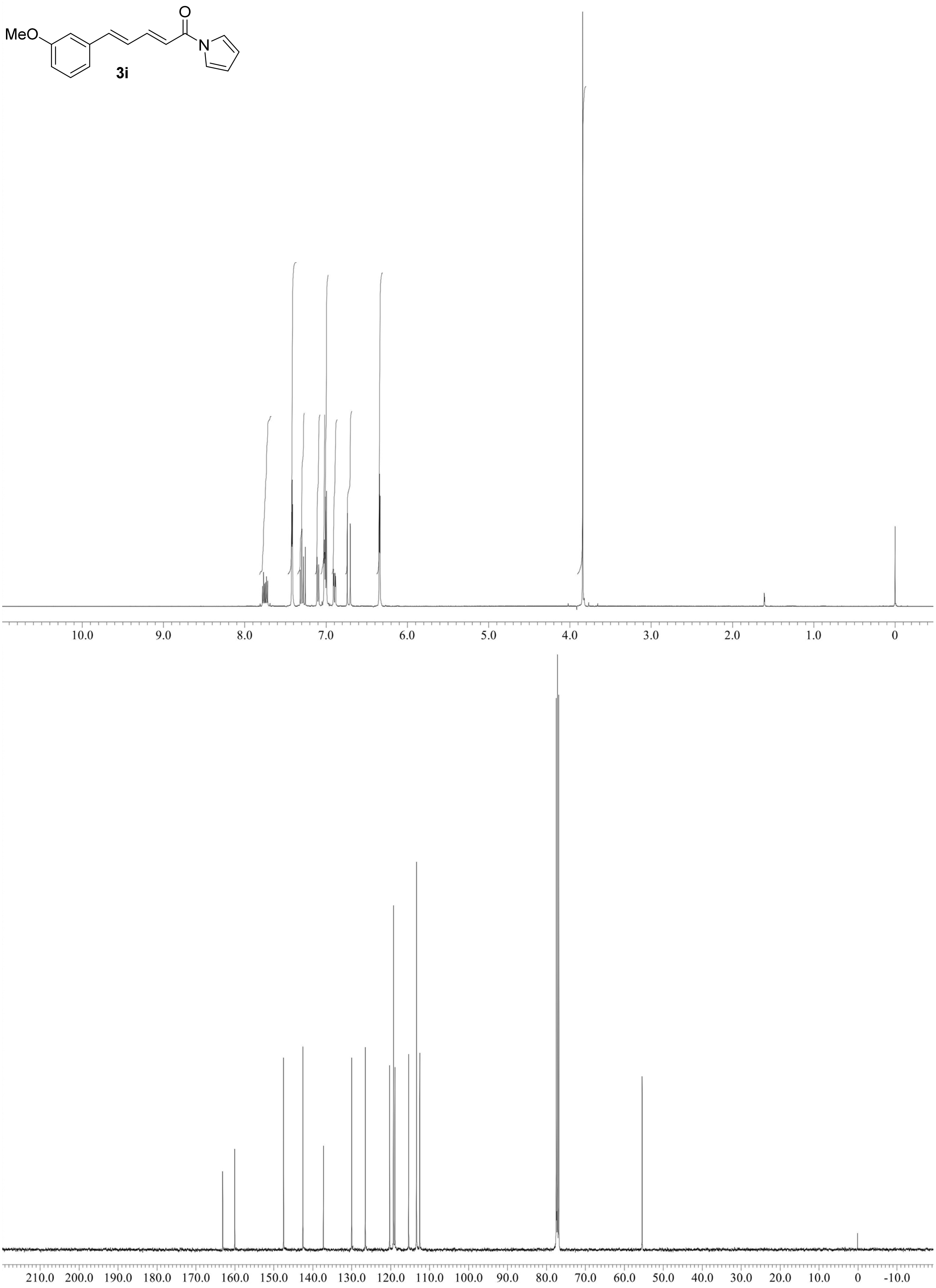

Supplementary Figure 12. ^{1}H and ^{13}C NMR spectra of **3i**

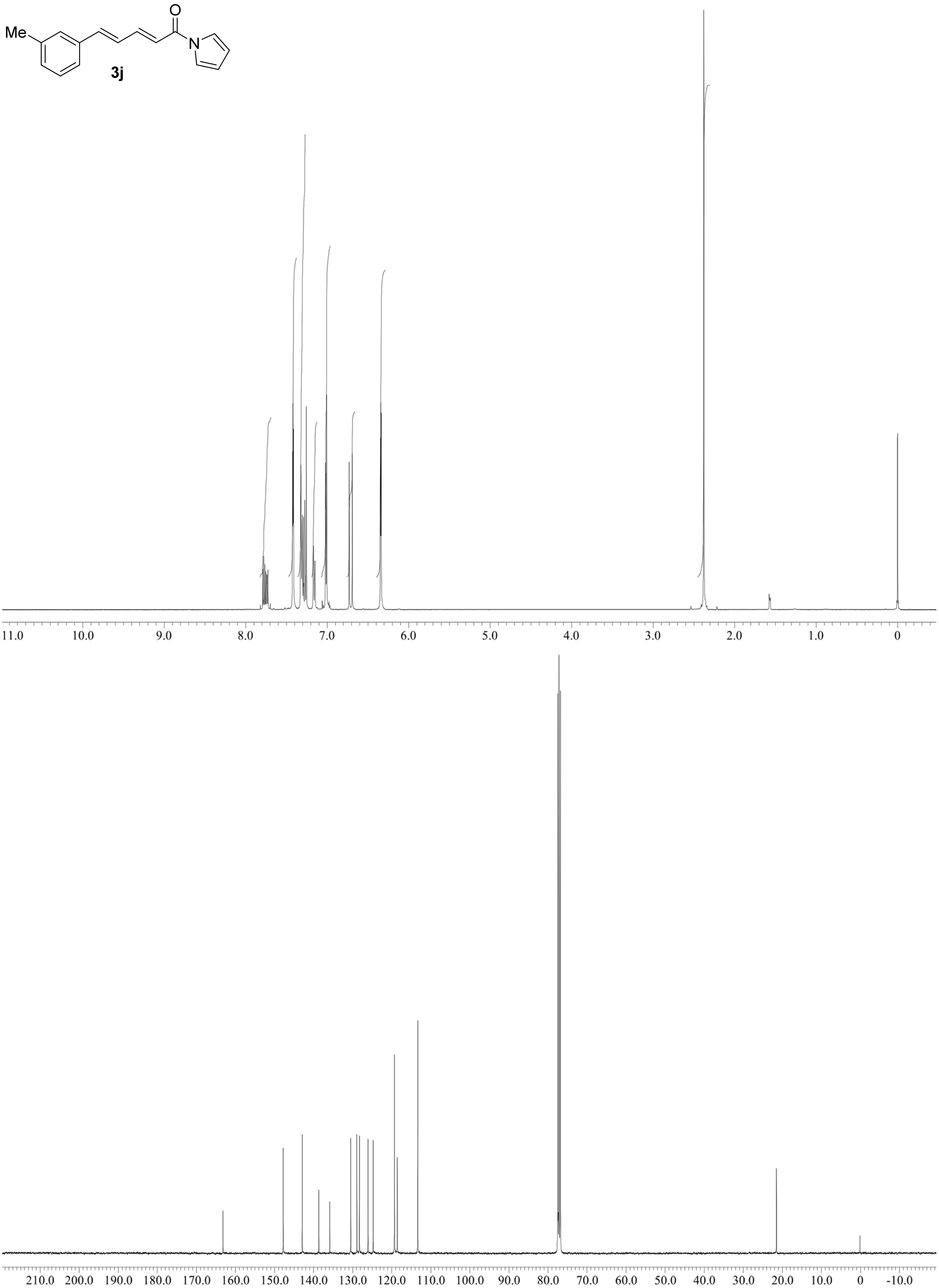

Supplementary Figure 13. ^{1}H and ^{13}C NMR spectra of **3j**

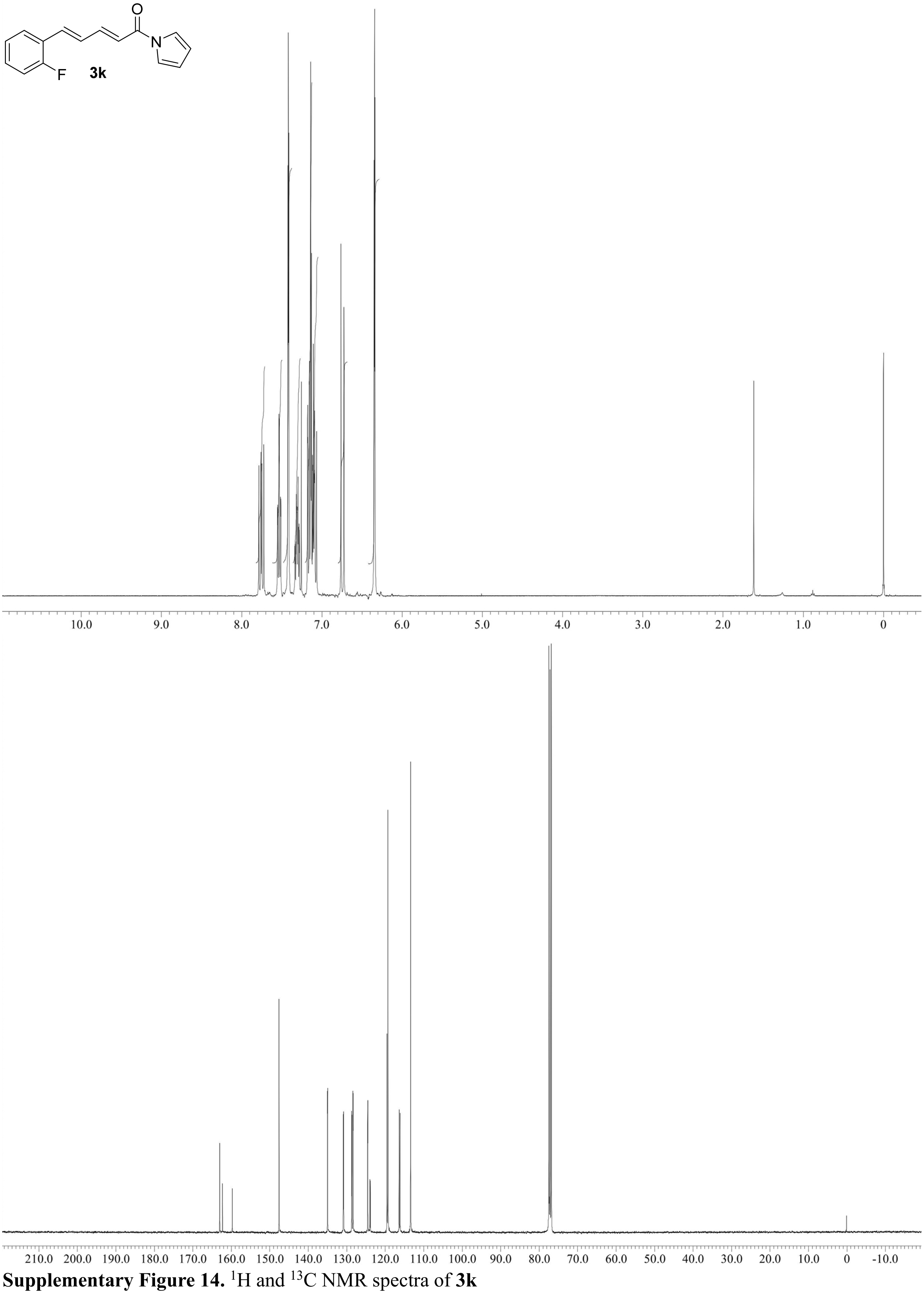

Supplementary Figure 14. ^{1}H and ^{13}C NMR spectra of **3k**

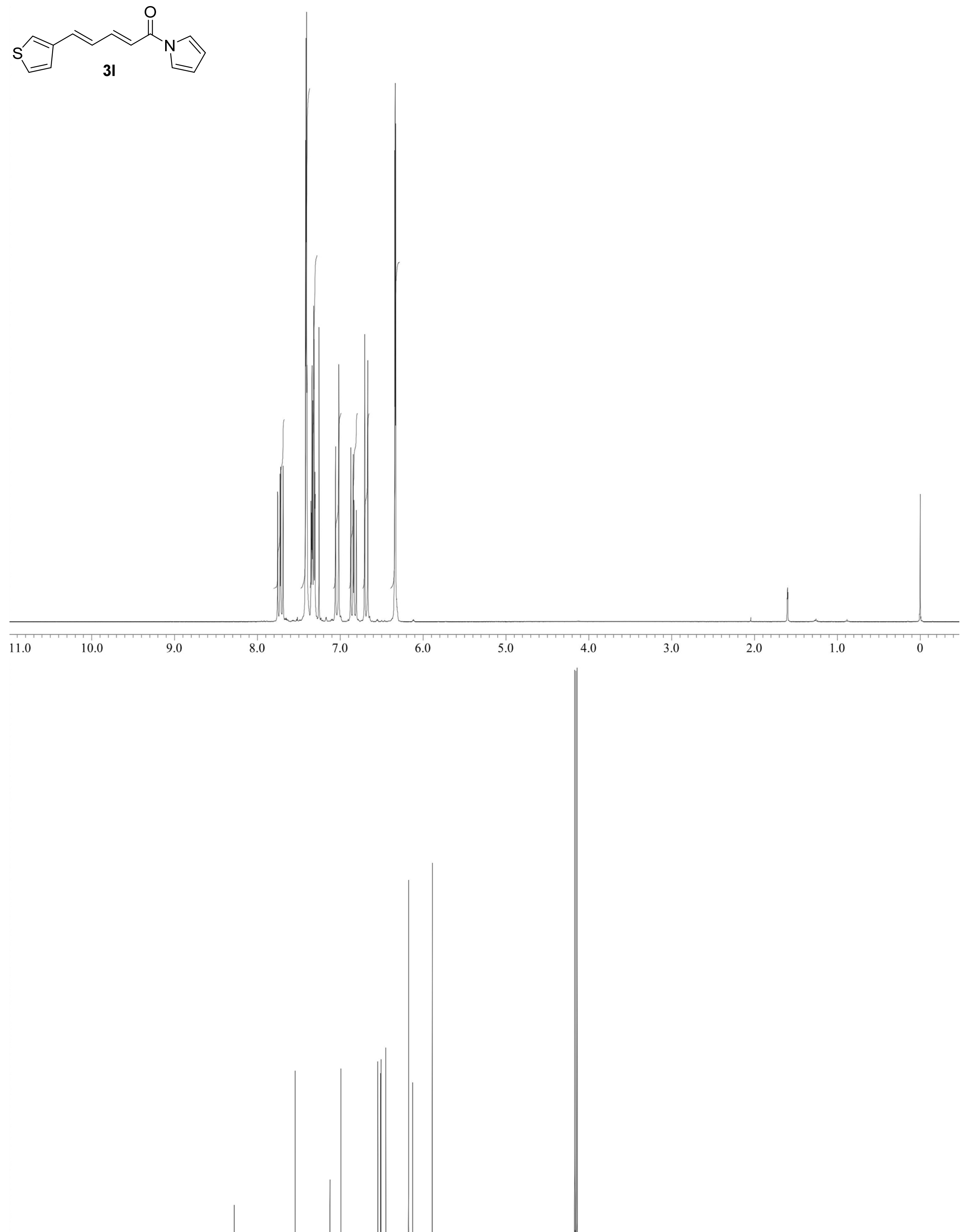

Supplementary Figure 15. ^{1}H and ^{13}C NMR spectra of **3l**

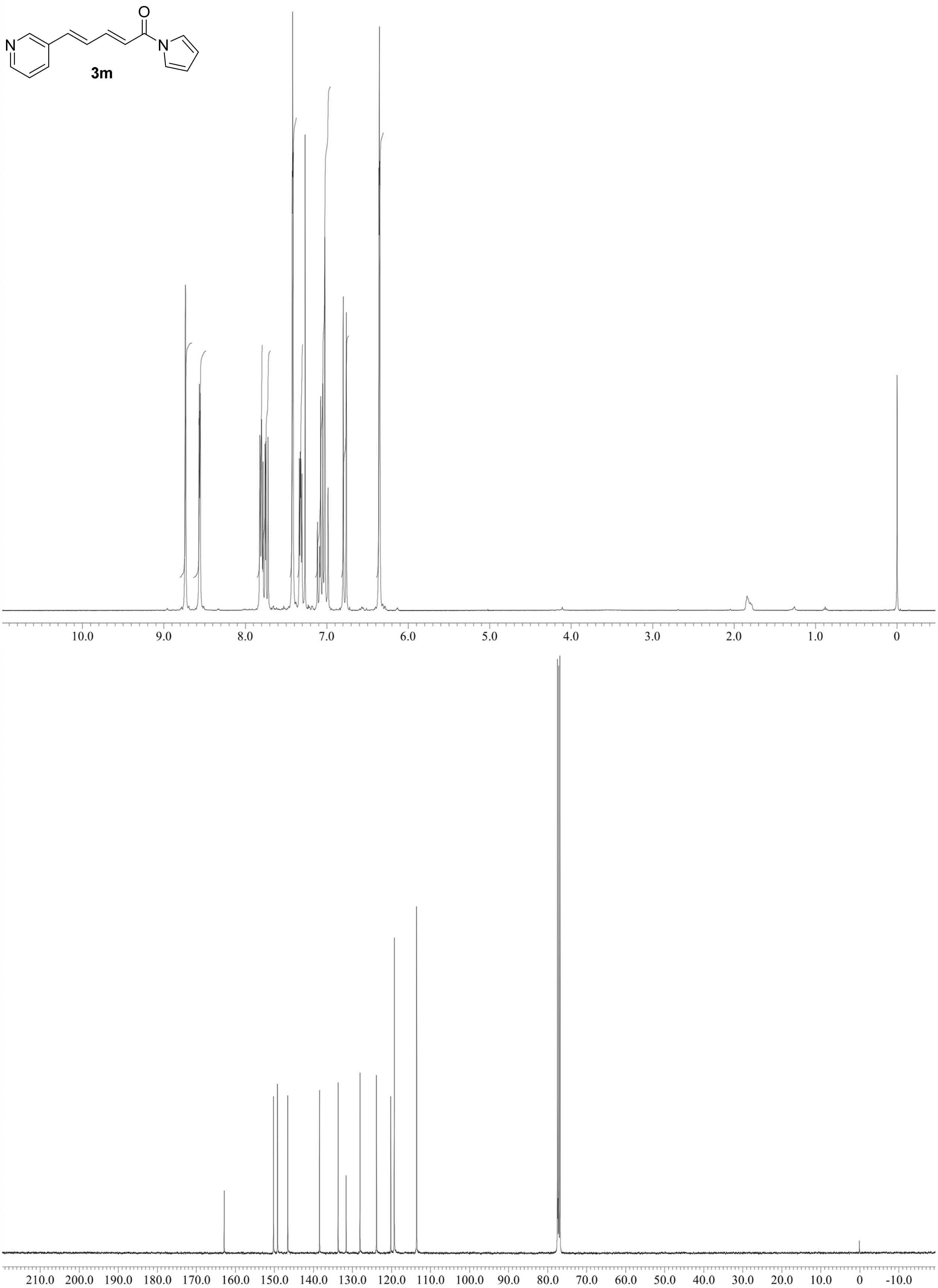

Supplementary Figure 16. ^{1}H and ^{13}C NMR spectra of **3m**

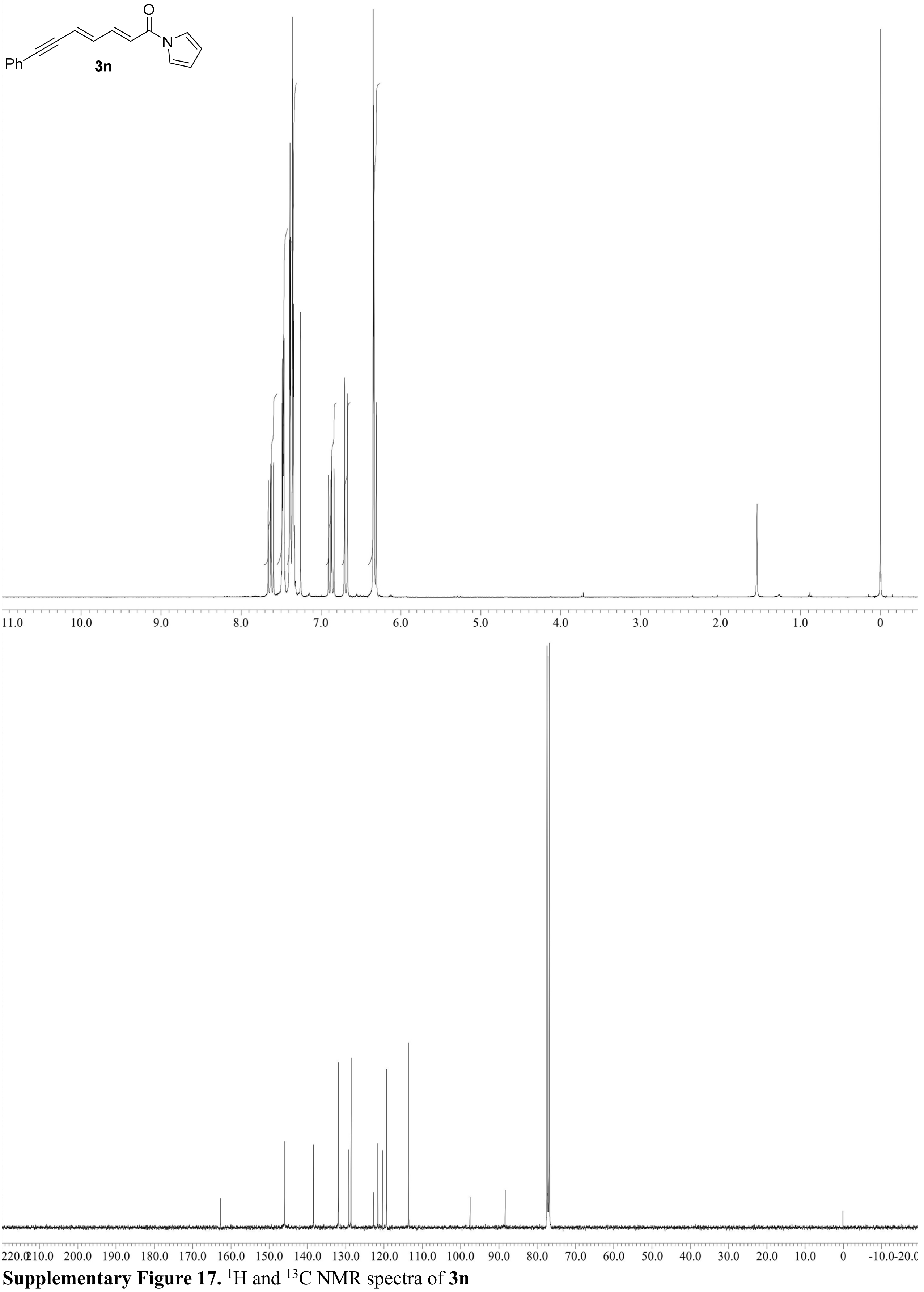

Supplementary Figure 17. ^{1}H and ^{13}C NMR spectra of **3n**

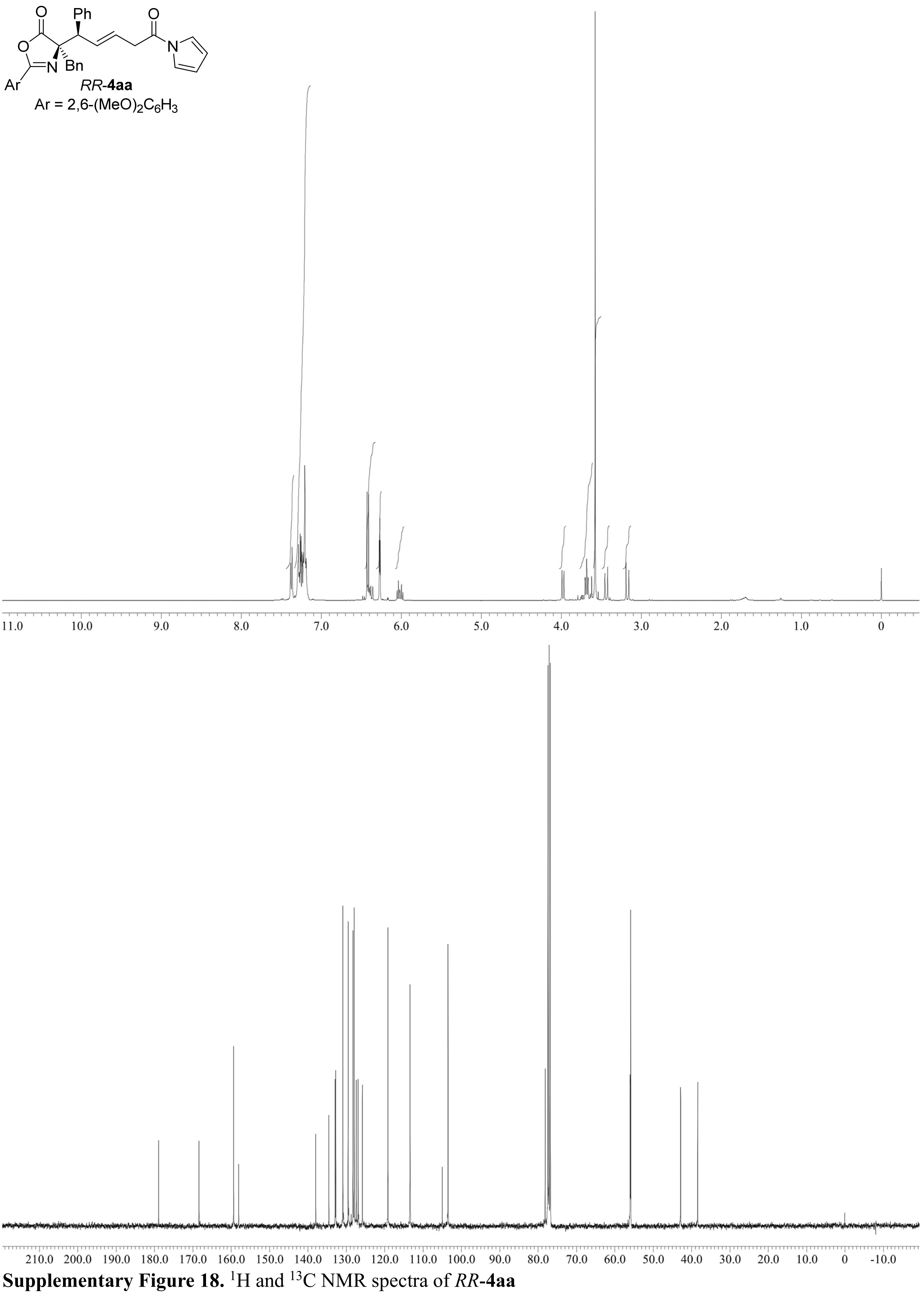

Supplementary Figure 18. ^{1}H and ^{13}C NMR spectra of *RR*-**4aa**

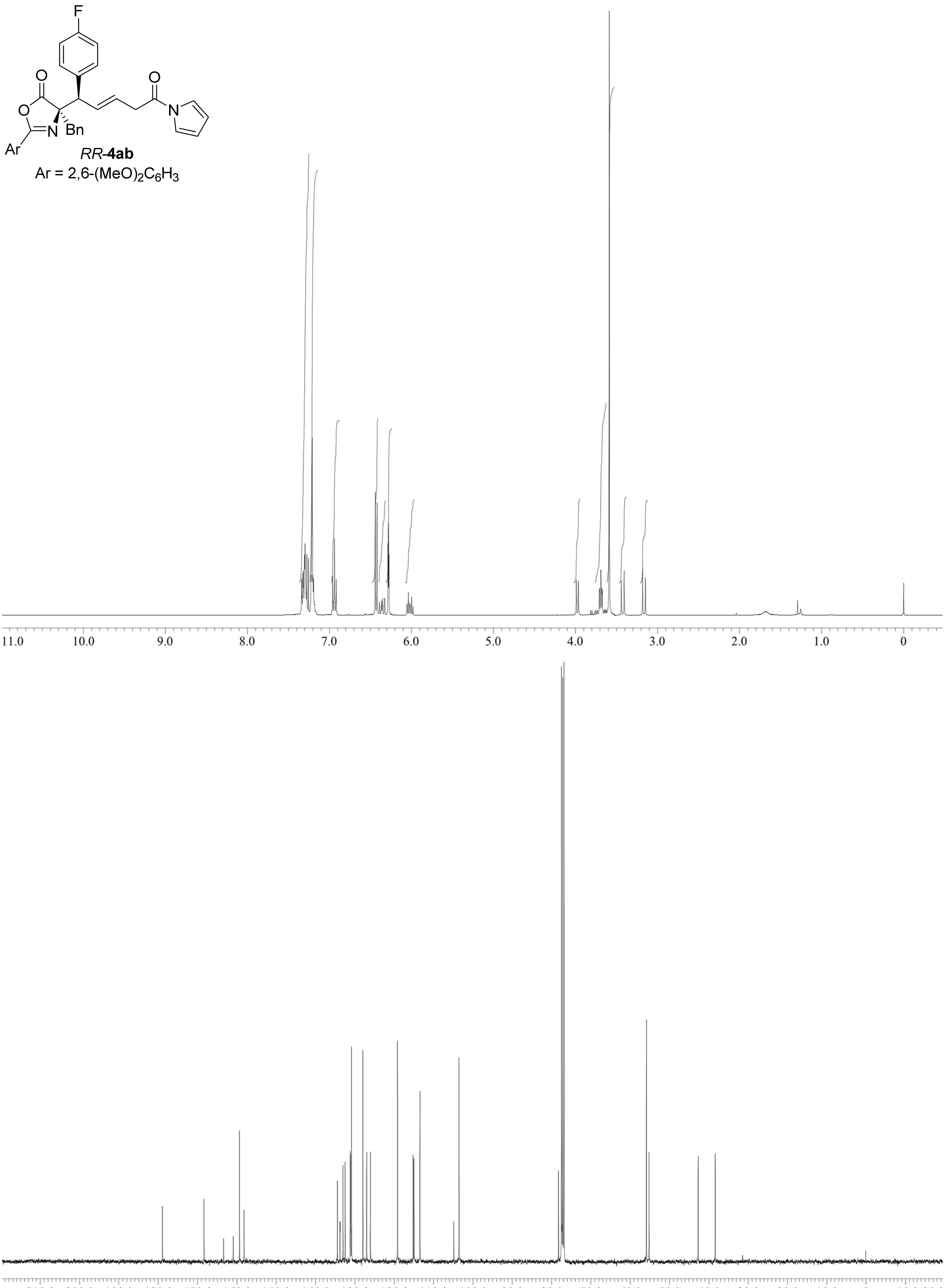

Supplementary Figure 19. ^{1}H and ^{13}C NMR spectra of *RR*-**4ab**

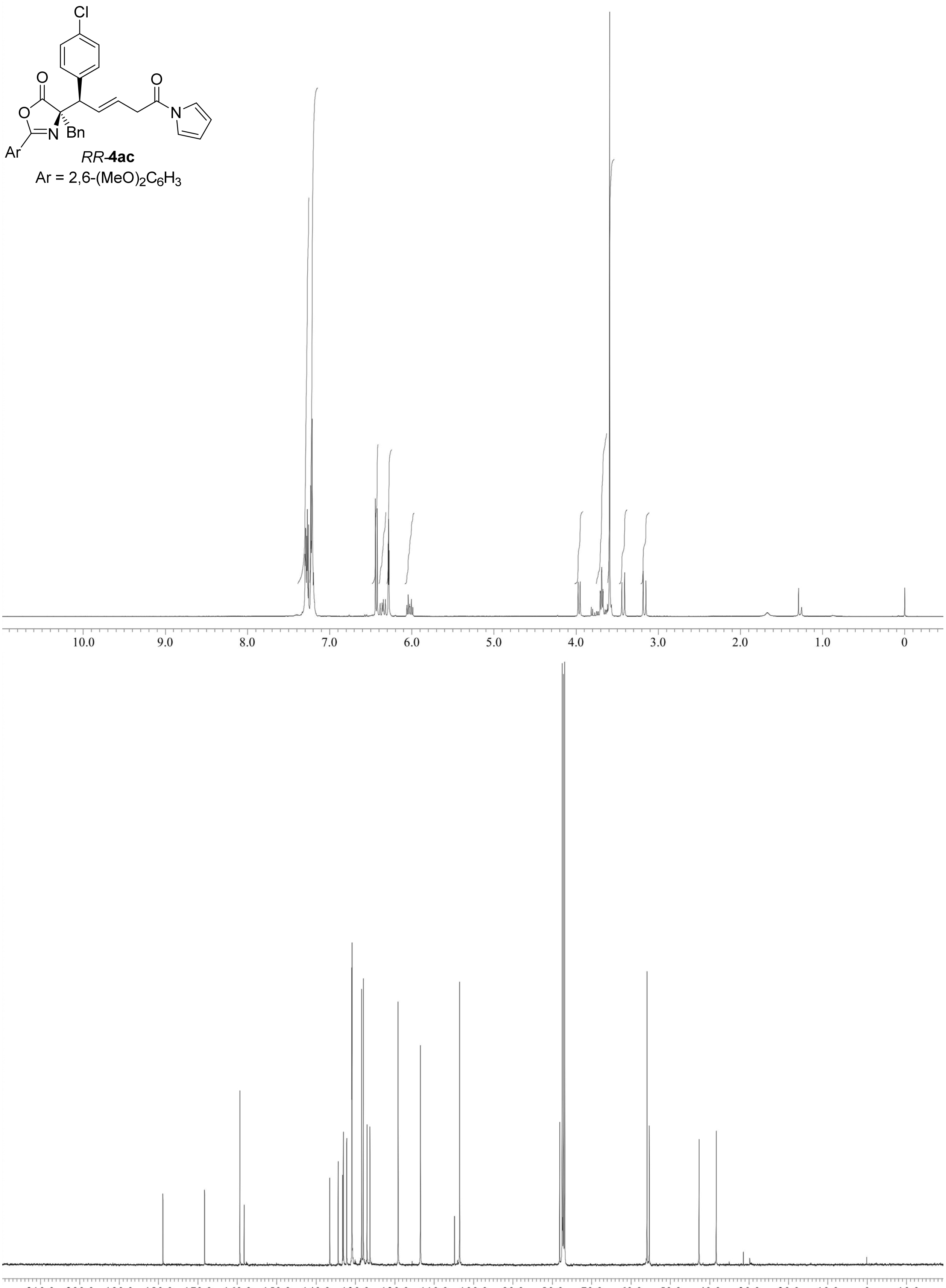

Supplementary Figure 20. ^{1}H and ^{13}C NMR spectra of *RR*-**4ac**

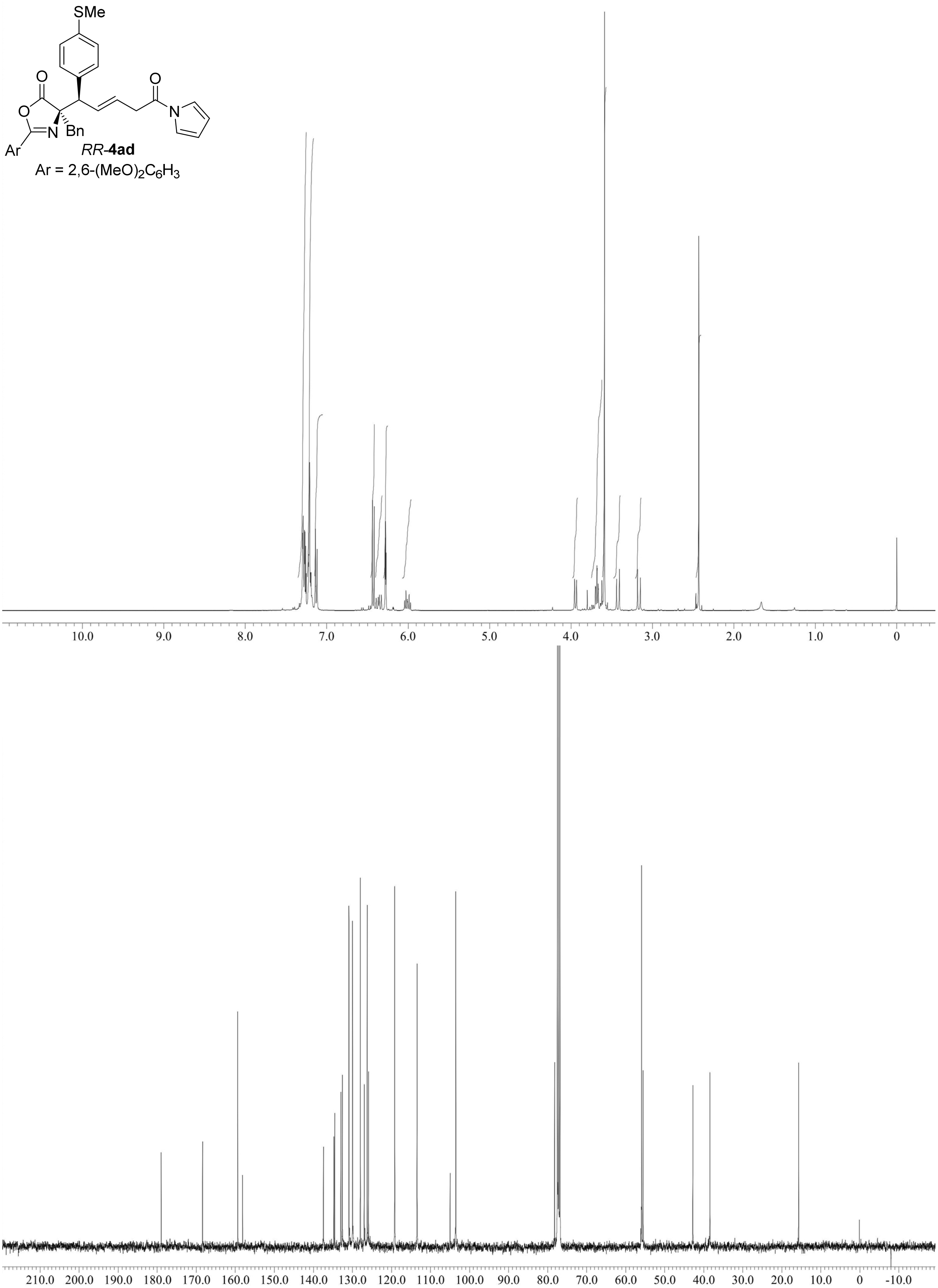

Supplementary Figure 21. ^{1}H and ^{13}C NMR spectra of *RR*-**4ad**

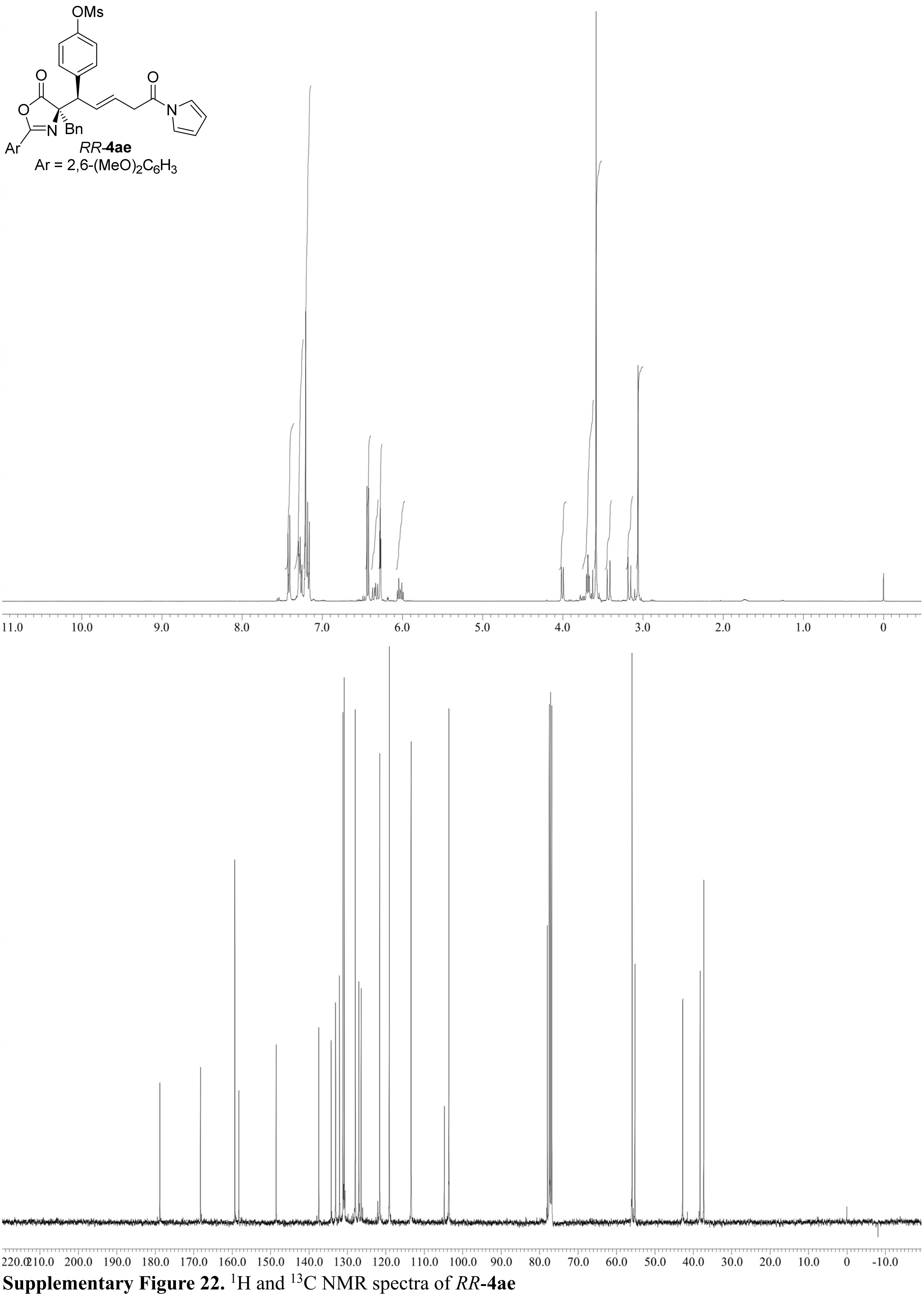

Supplementary Figure 22. ^{1}H and ^{13}C NMR spectra of *RR*-**4ae**

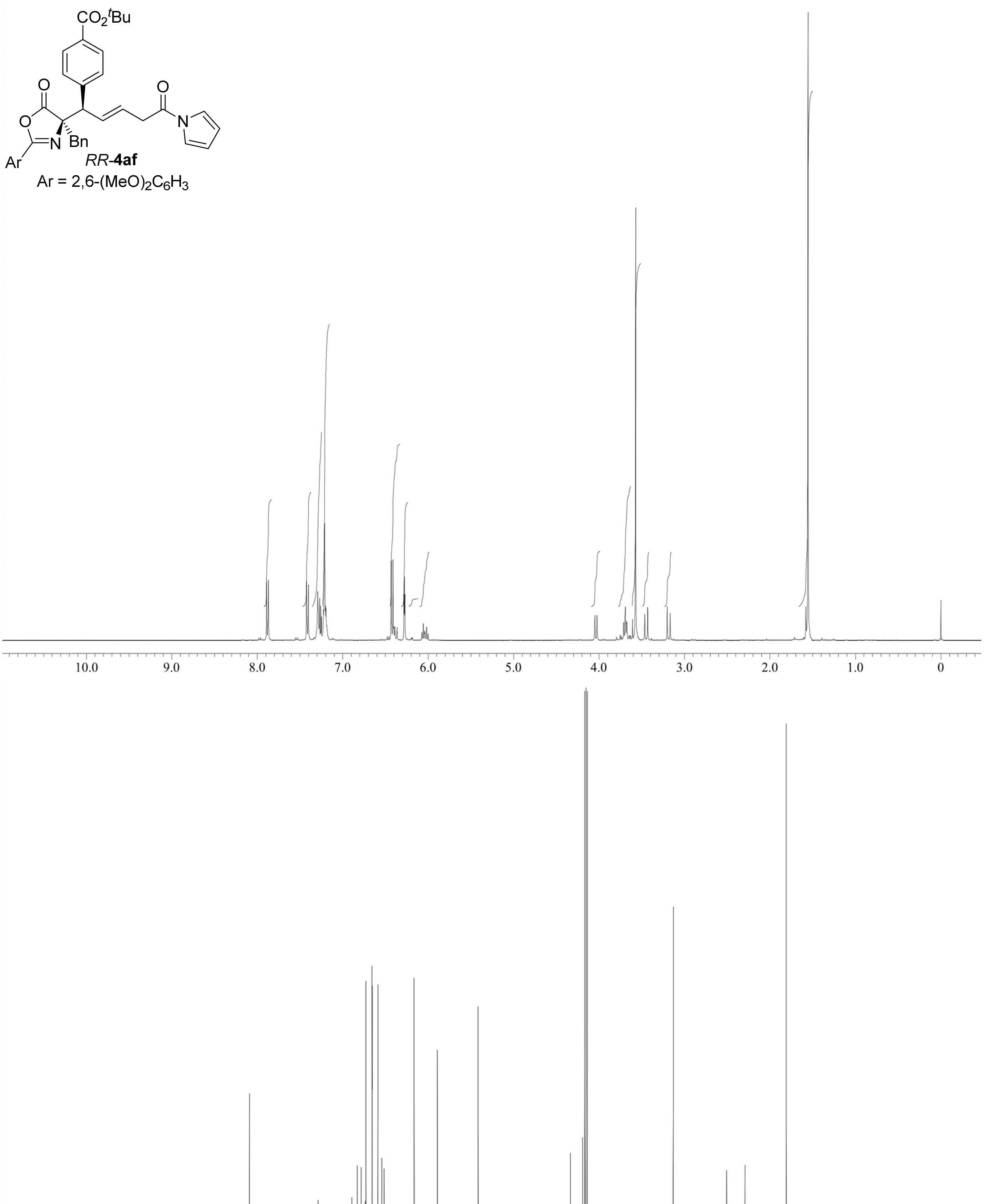

Supplementary Figure 23. ^{1}H and ^{13}C NMR spectra of *RR*-**4af**

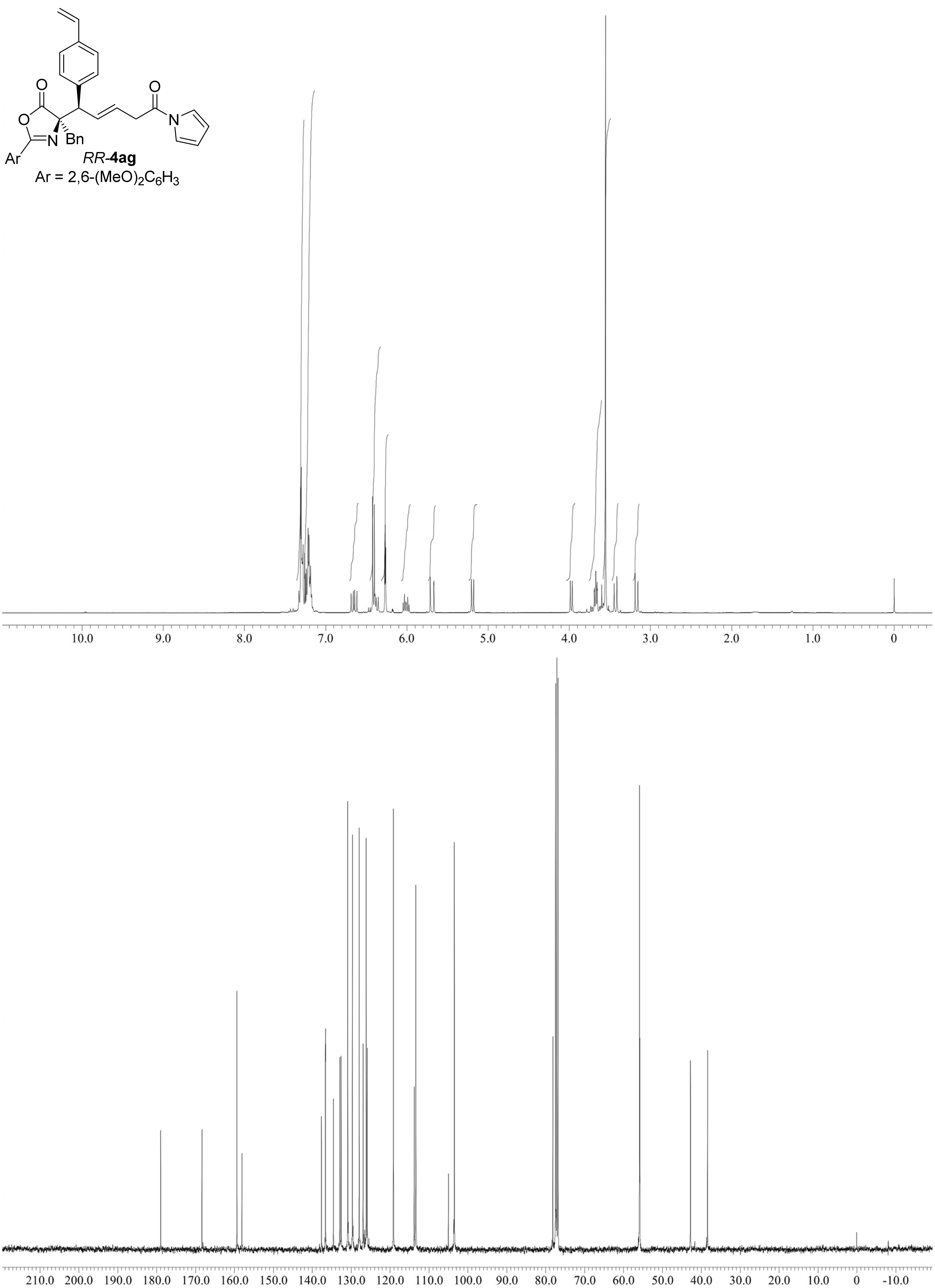

Supplementary Figure 24. ^{1}H and ^{13}C NMR spectra of *RR*-**4ag**

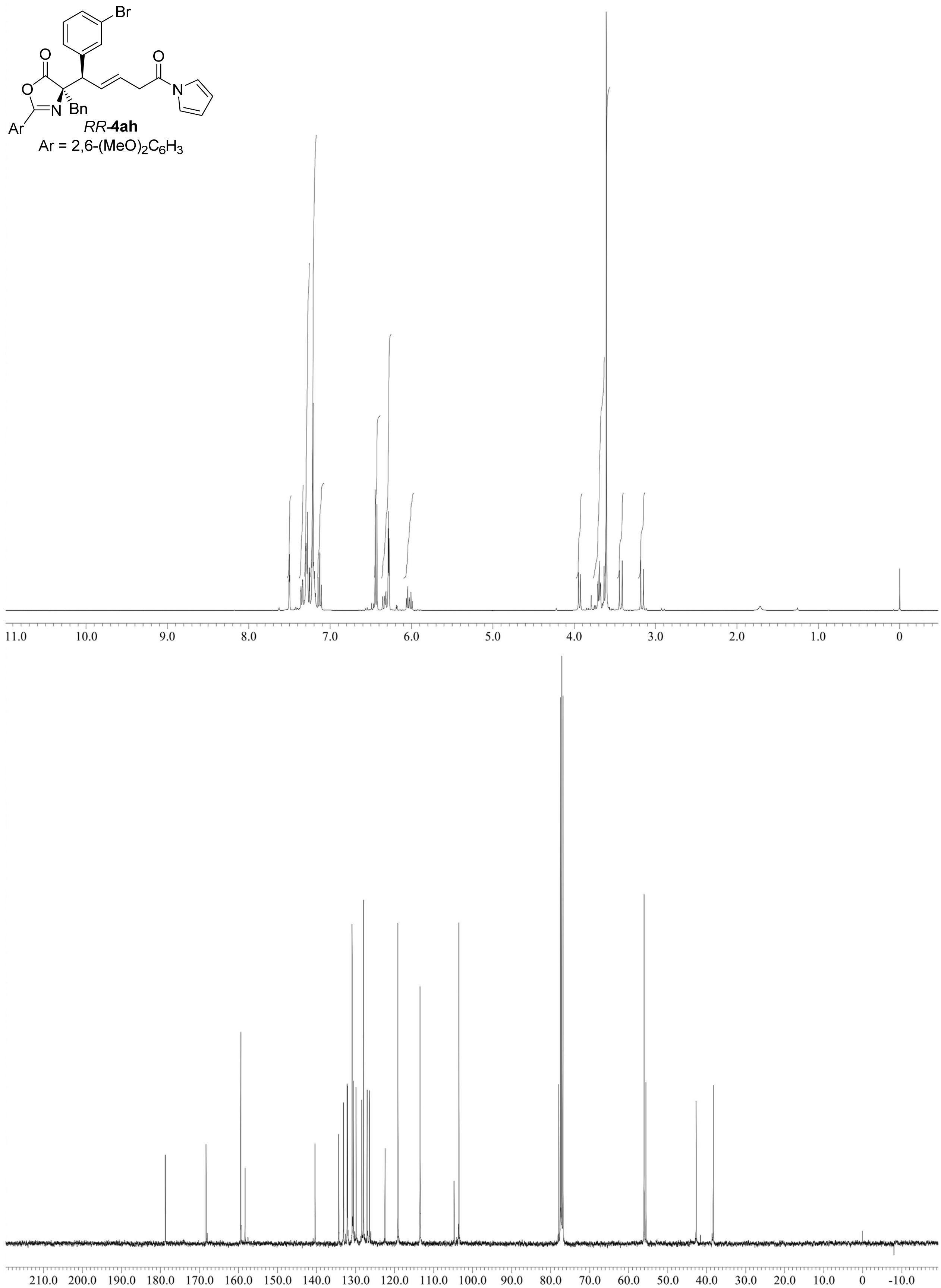

Supplementary Figure 25. ^{1}H and ^{13}C NMR spectra of *RR*-**4ah**

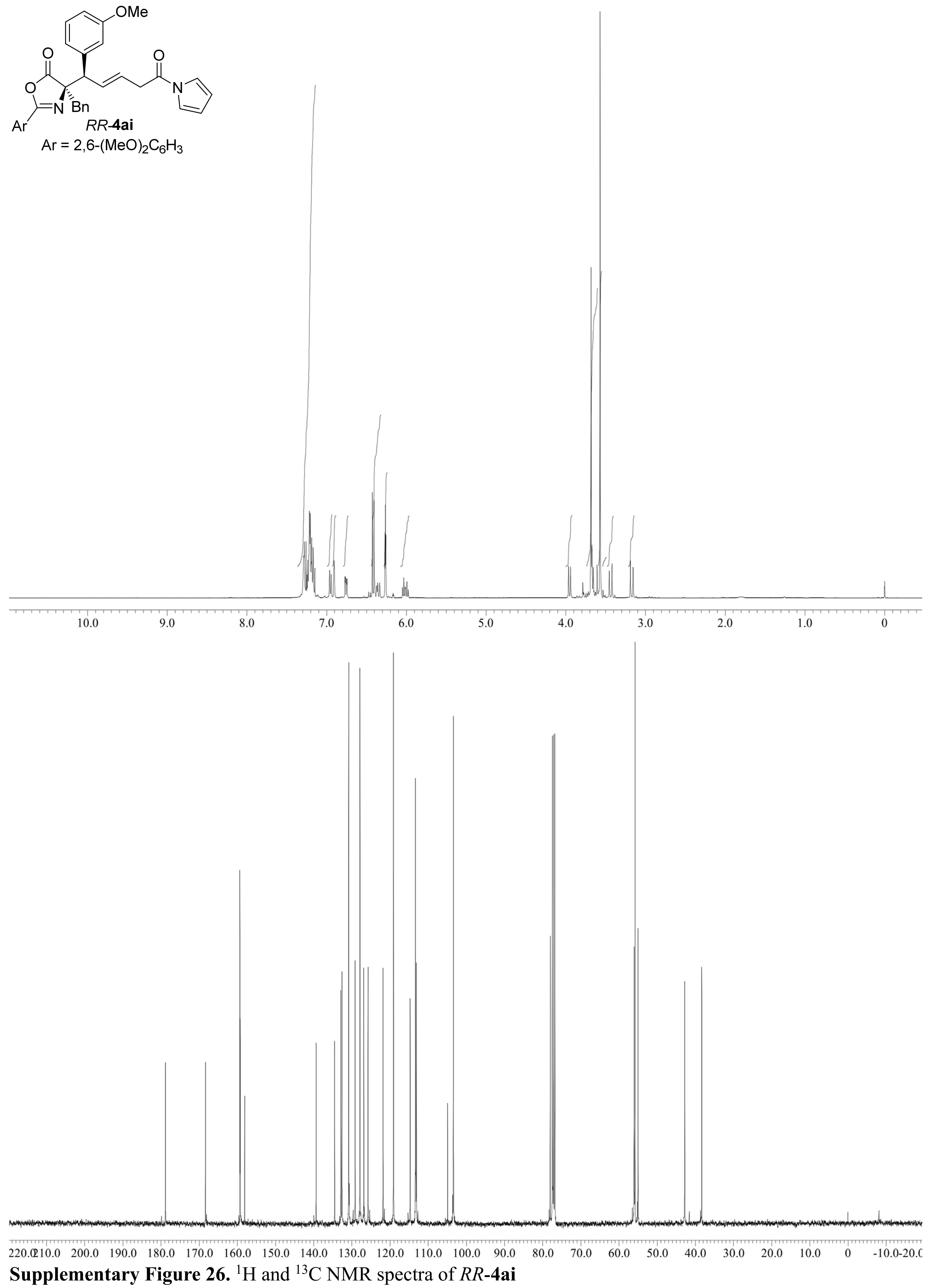

Supplementary Figure 26. ^{1}H and ^{13}C NMR spectra of *RR*-**4ai**

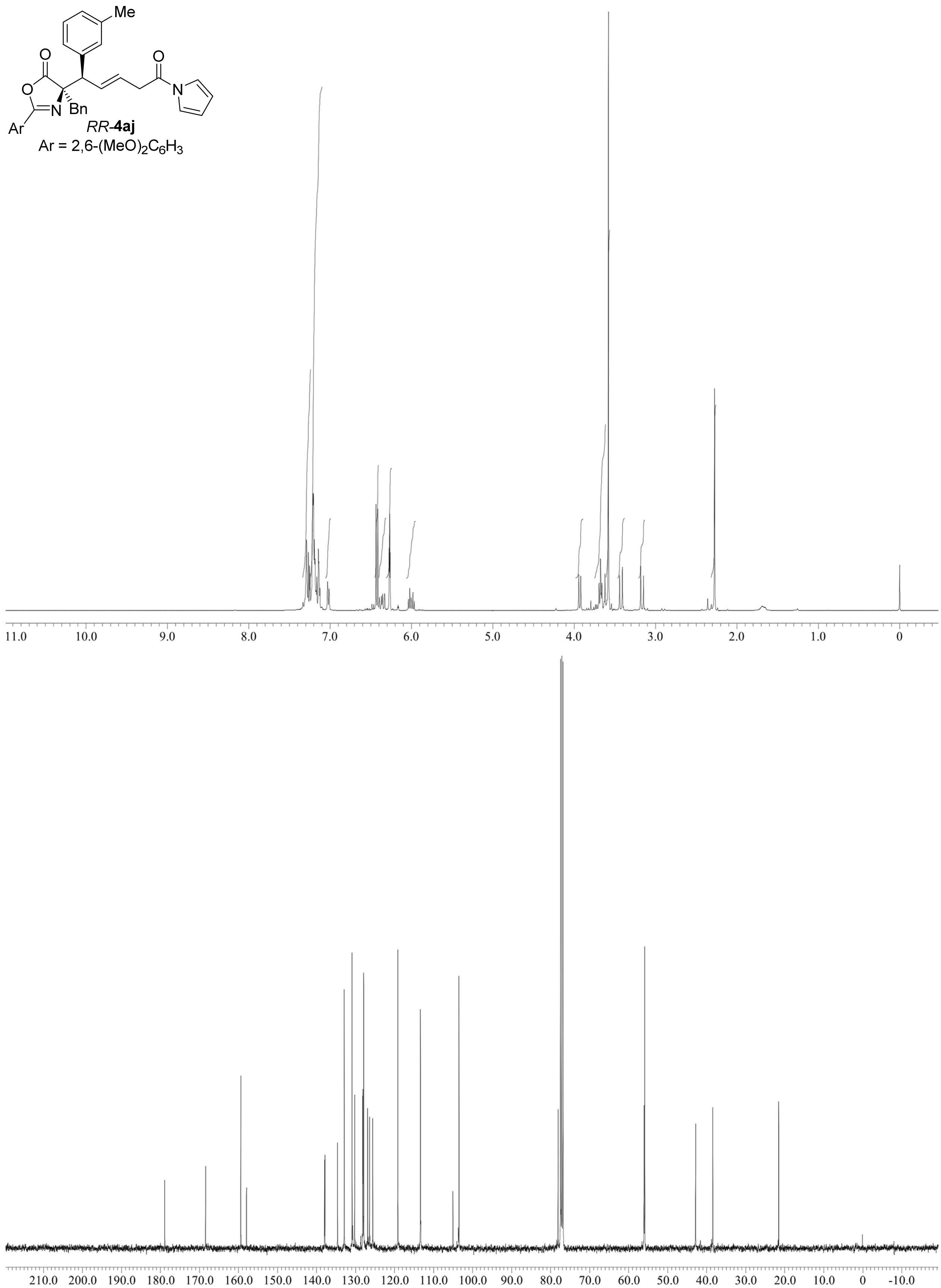

Supplementary Figure 27. ^{1}H and ^{13}C NMR spectra of *RR*-**4aj**

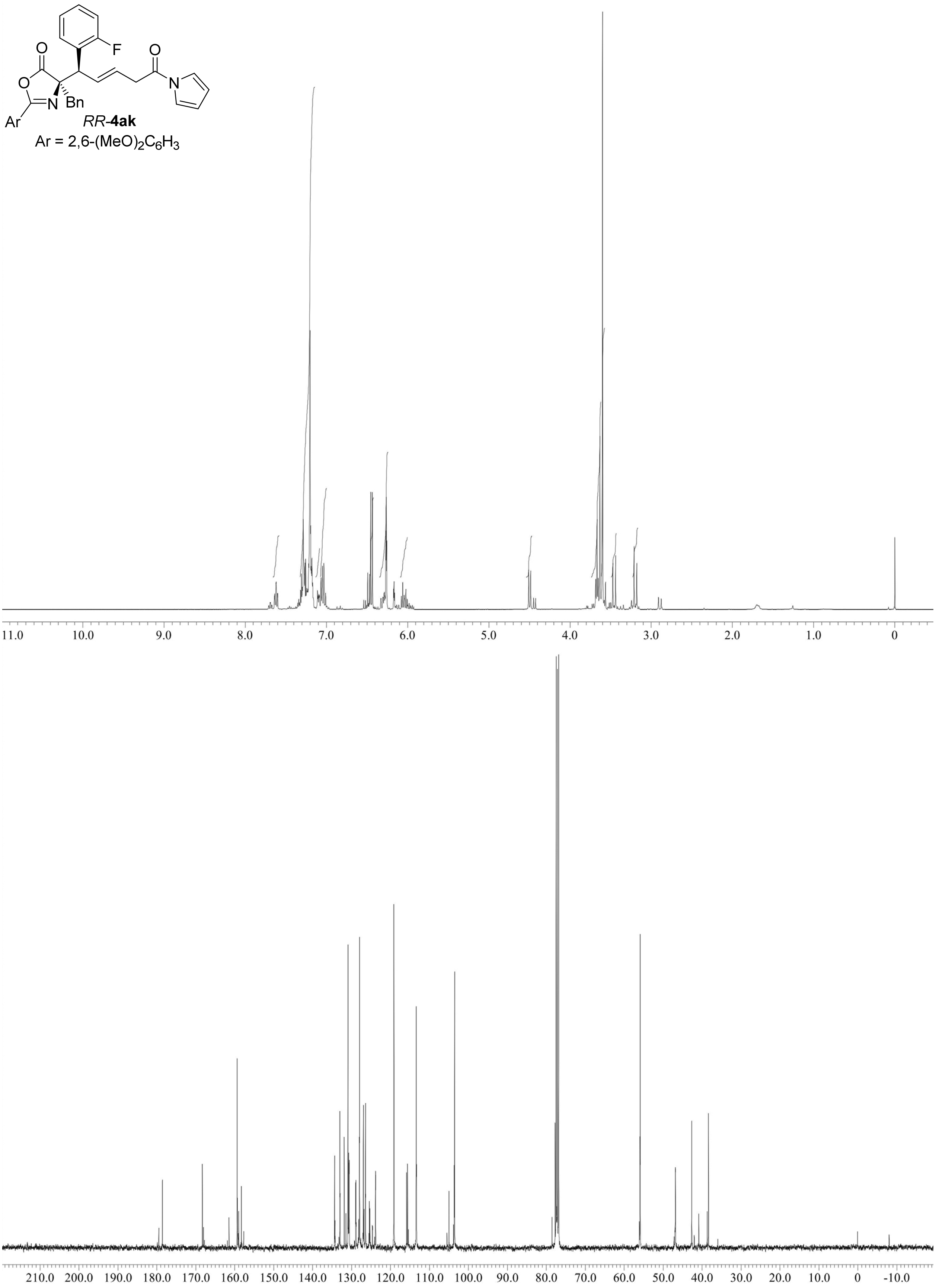

Supplementary Figure 28. ^{1}H and ^{13}C NMR spectra of *RR*-**4ak**

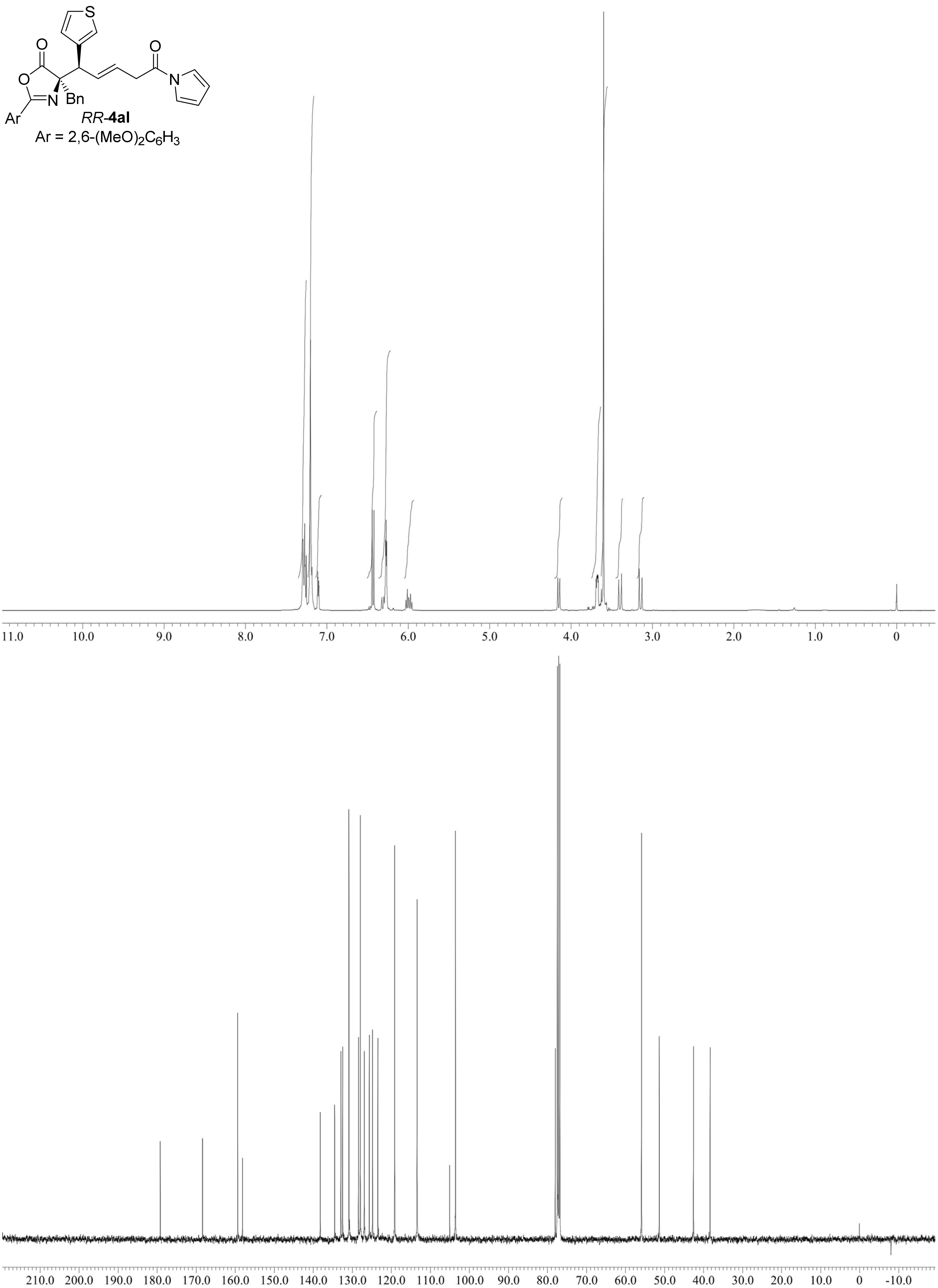

Supplementary Figure 29. ^{1}H and ^{13}C NMR spectra of *RR*-**4al**

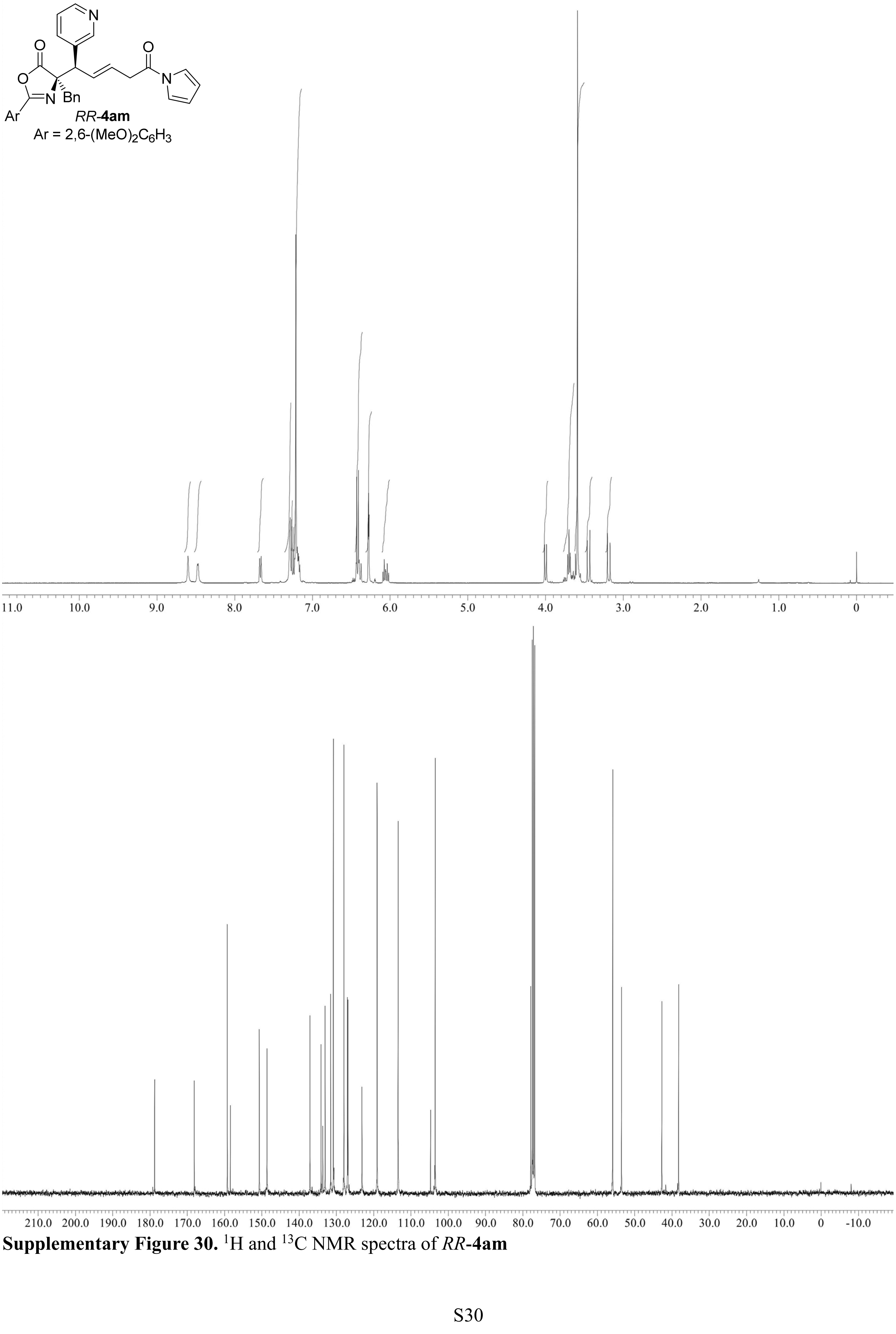

Supplementary Figure 30. ^{1}H and ^{13}C NMR spectra of *RR*-**4am**

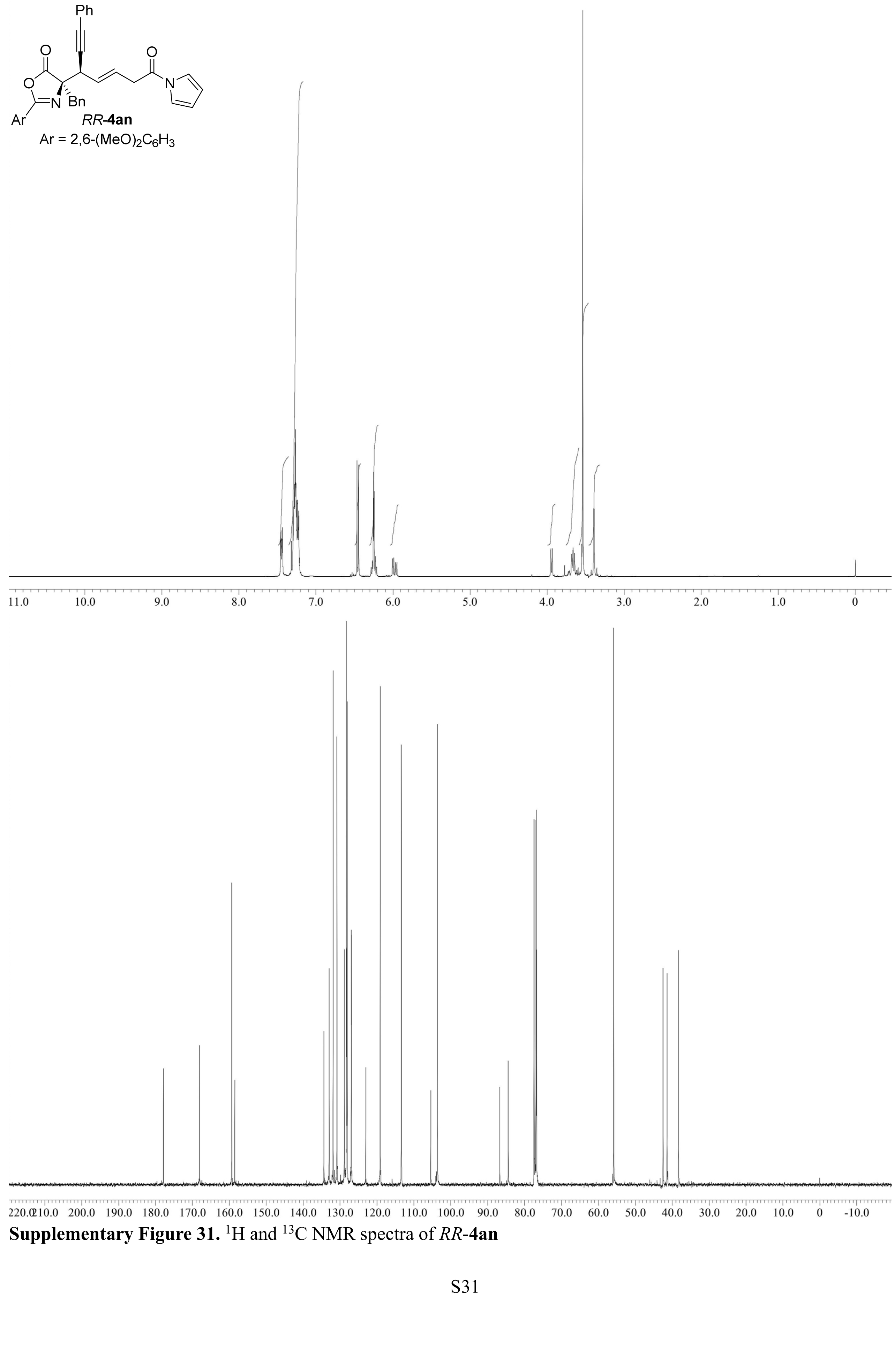

Supplementary Figure 31. ^{1}H and ^{13}C NMR spectra of *RR*-**4an**

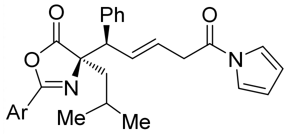

RR-**4ba**
Ar = 2,6-(MeO)$_2$C$_6$H$_3$

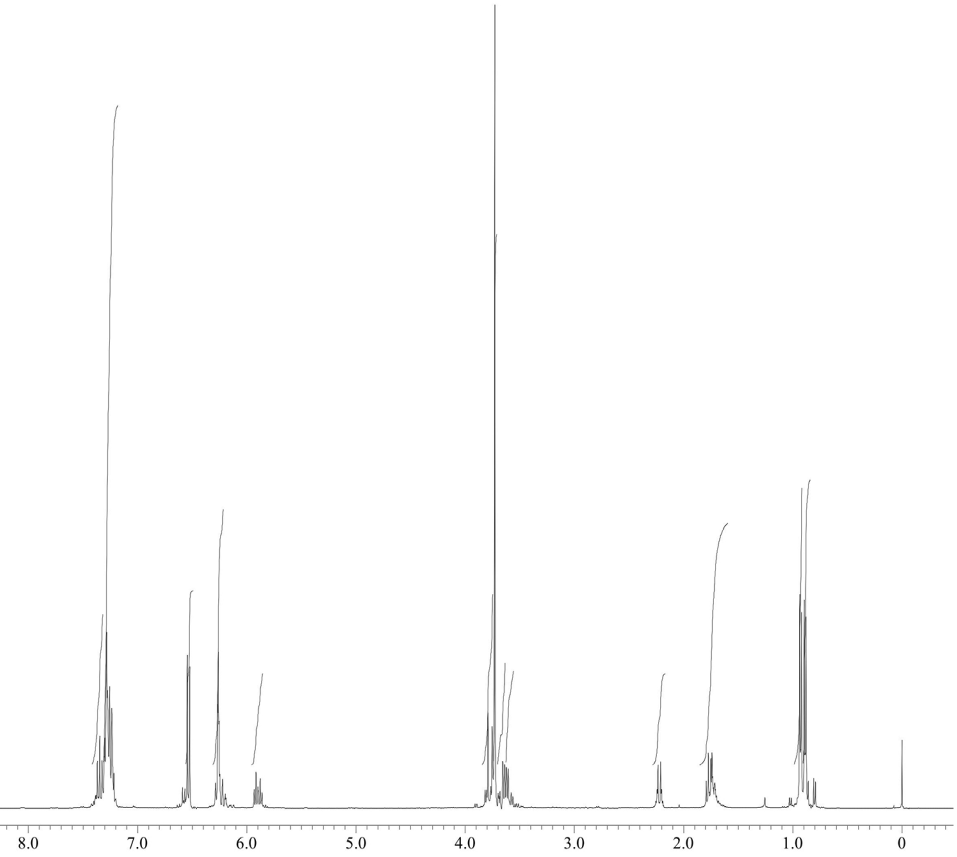

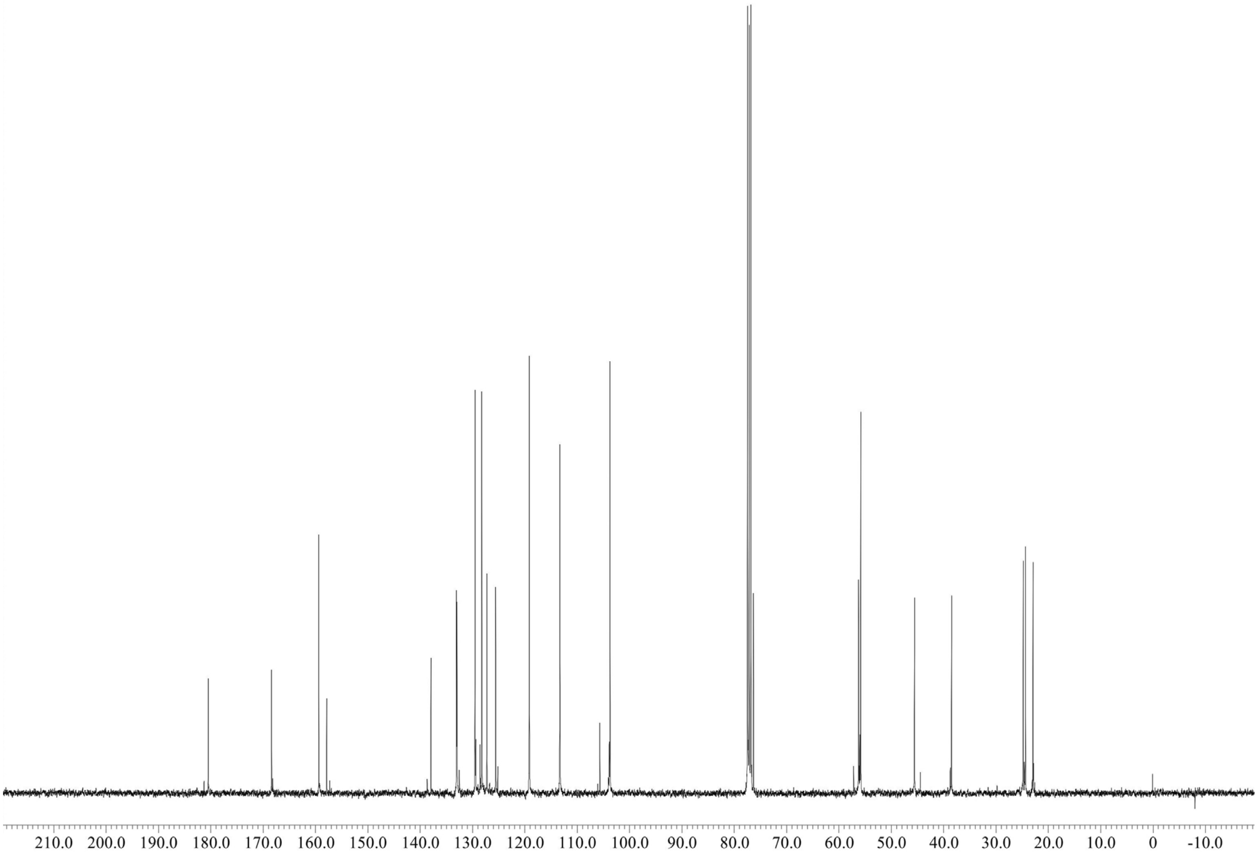

Supplementary Figure 32. ^{1}H and ^{13}C NMR spectra of *RR*-**4ba**

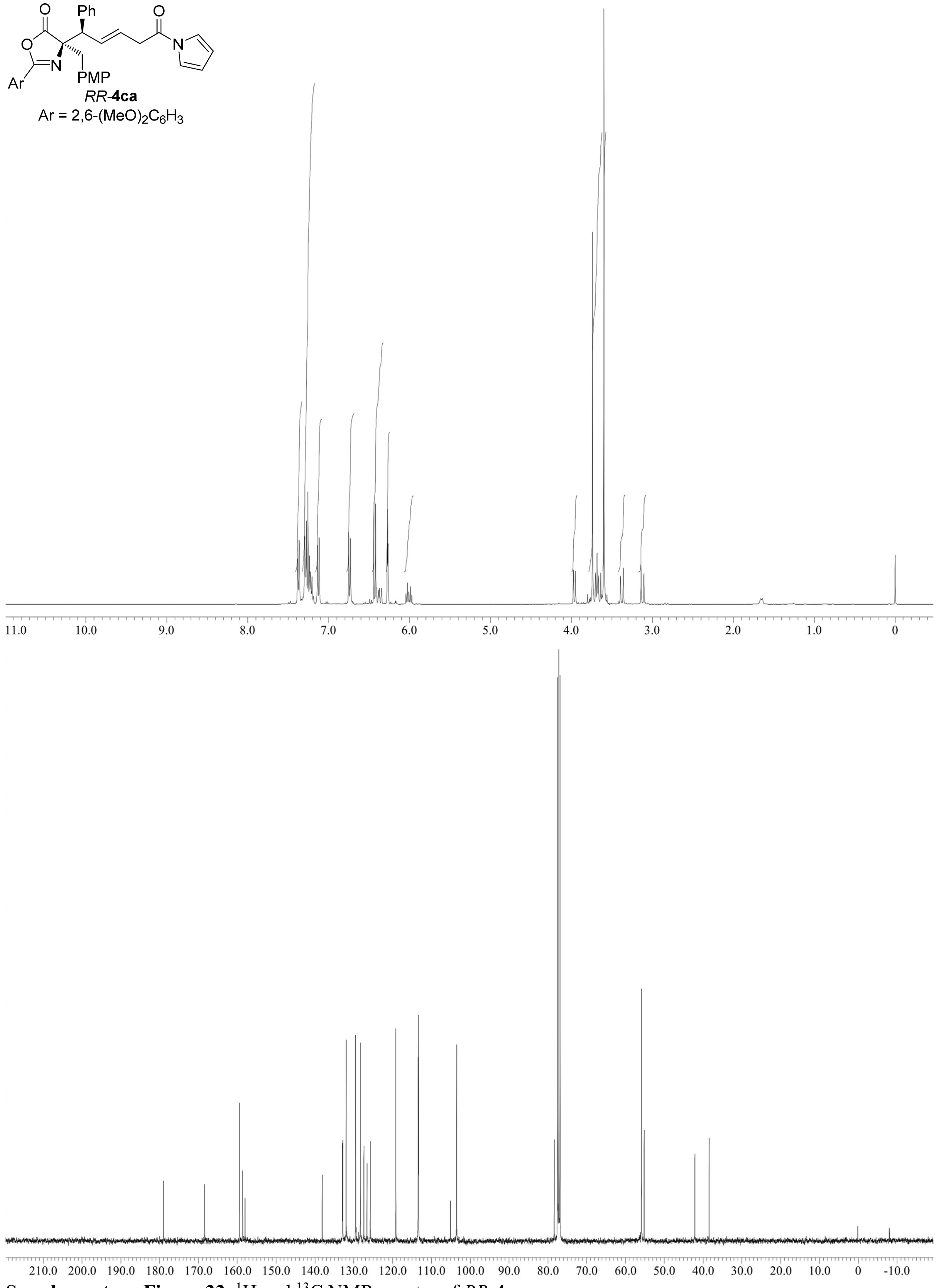

Supplementary Figure 33. ^{1}H and ^{13}C NMR spectra of *RR*-**4ca**

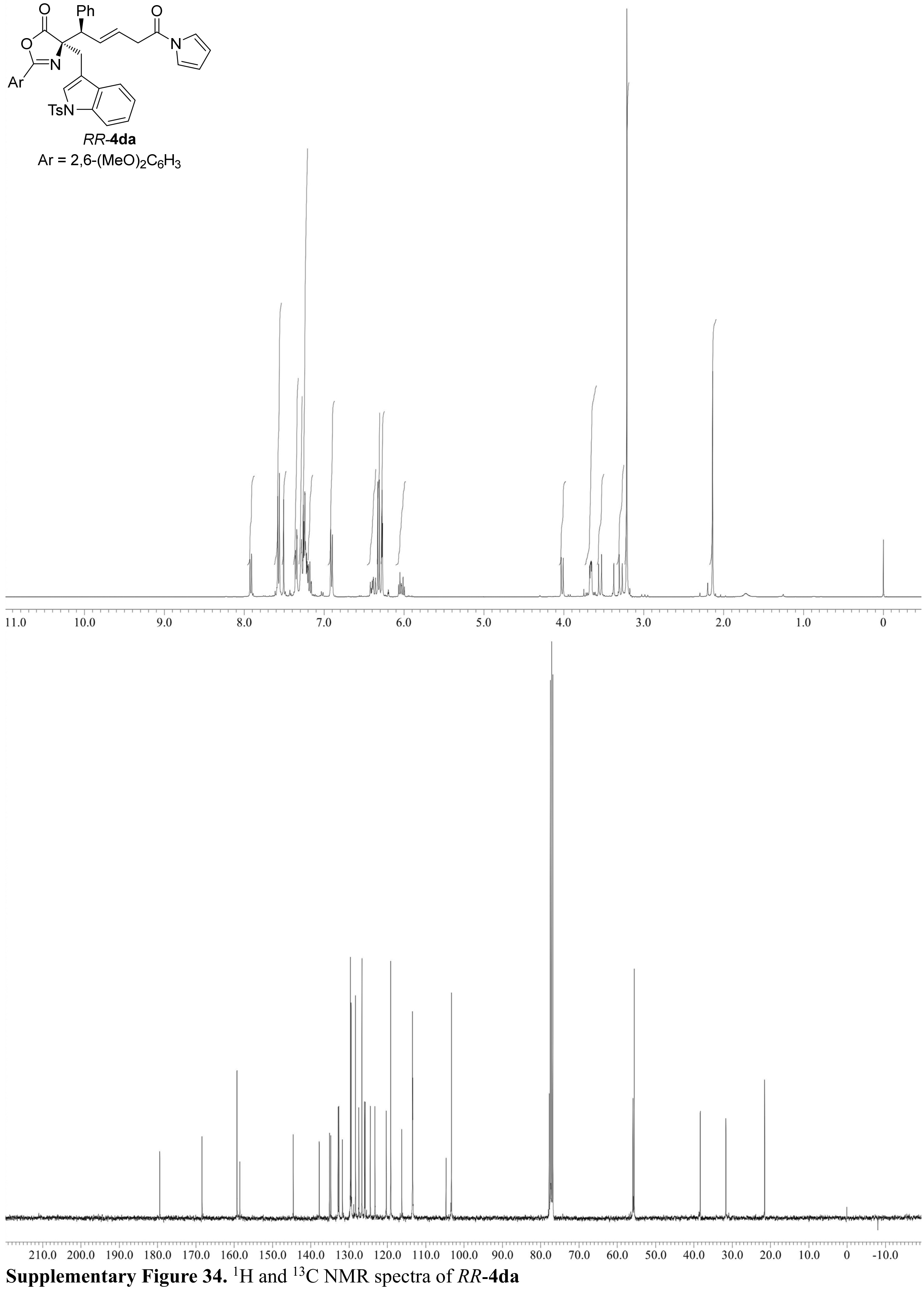

Supplementary Figure 34. ^{1}H and ^{13}C NMR spectra of *RR*-**4da**

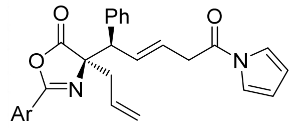

RR-**4ea**
Ar = 2,6-$(MeO)_2C_6H_3$

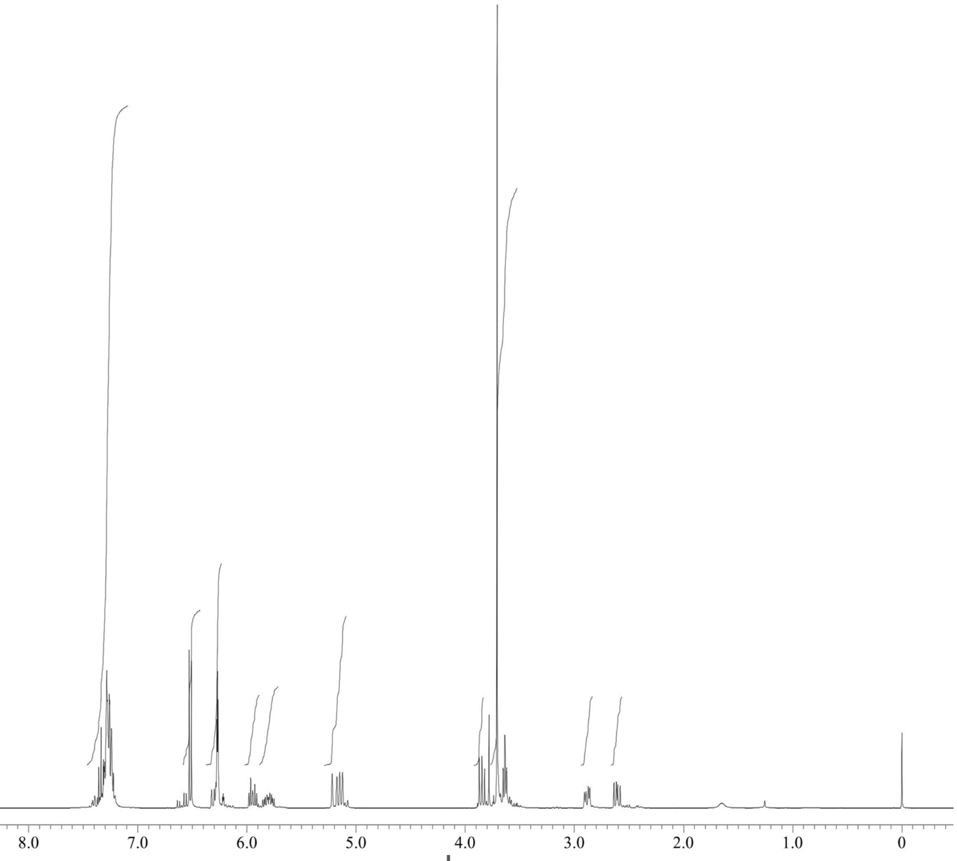

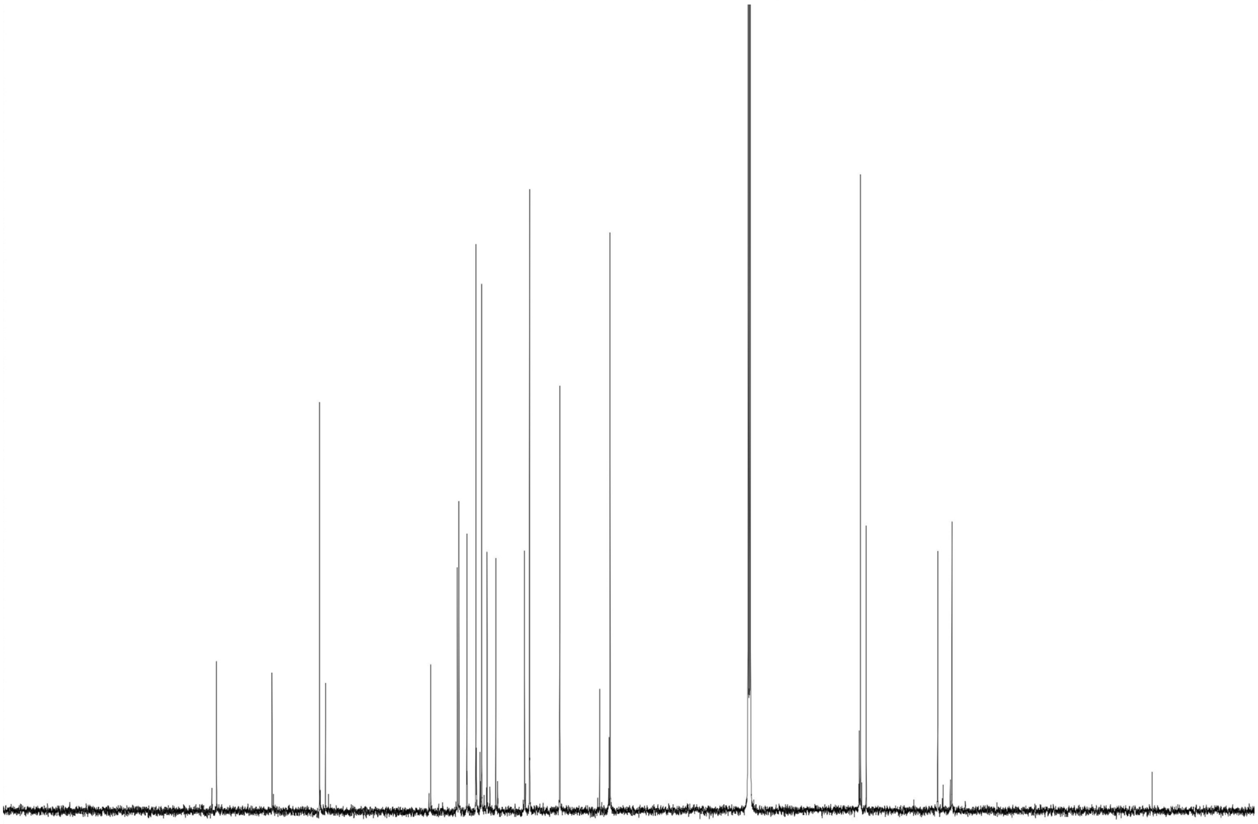

Supplementary Figure 35. ^{1}H and ^{13}C NMR spectra of *RR*-**4ea**

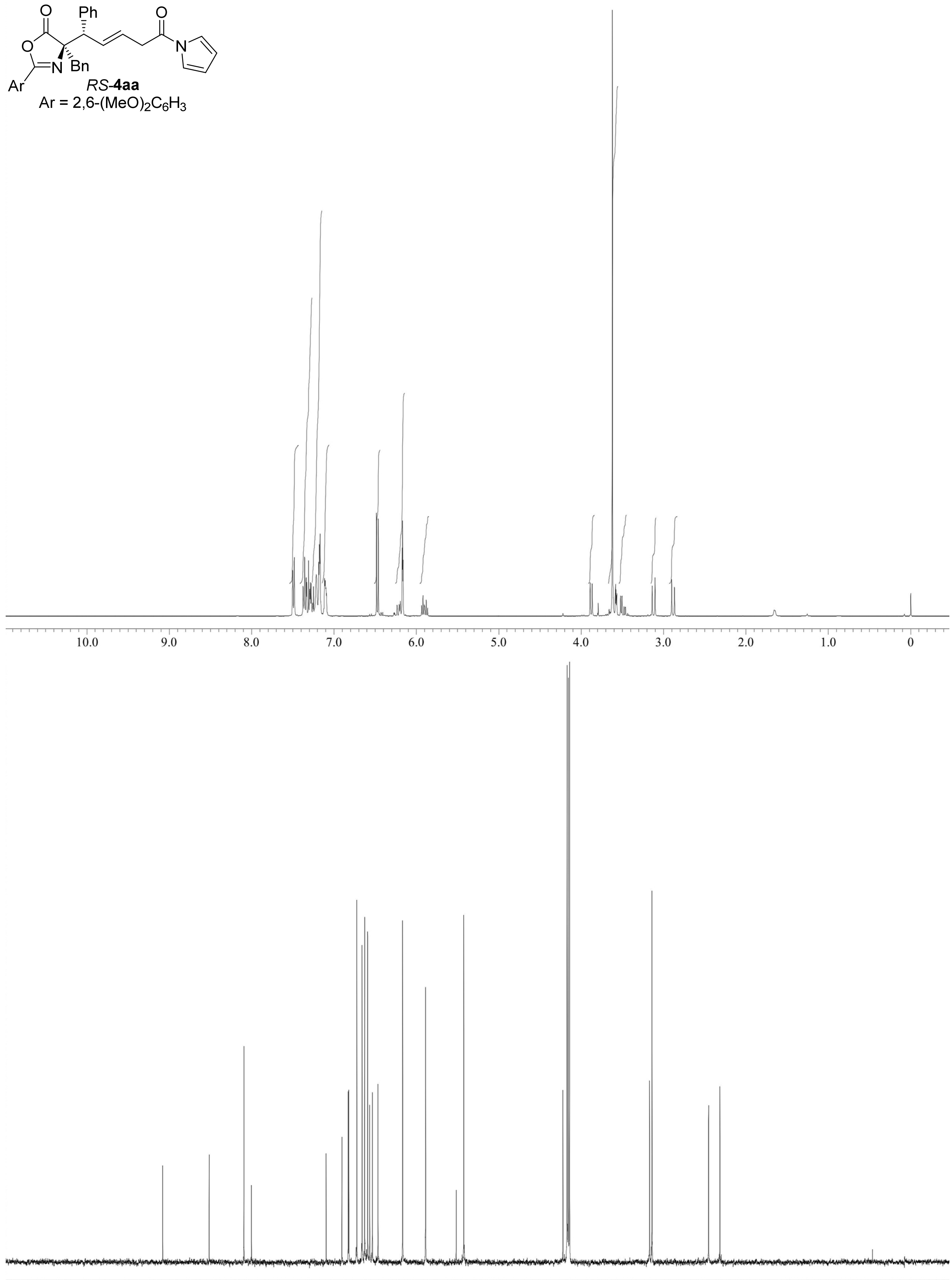

Supplementary Figure 36. ^{1}H and ^{13}C NMR spectra of *RS*-**4aa**

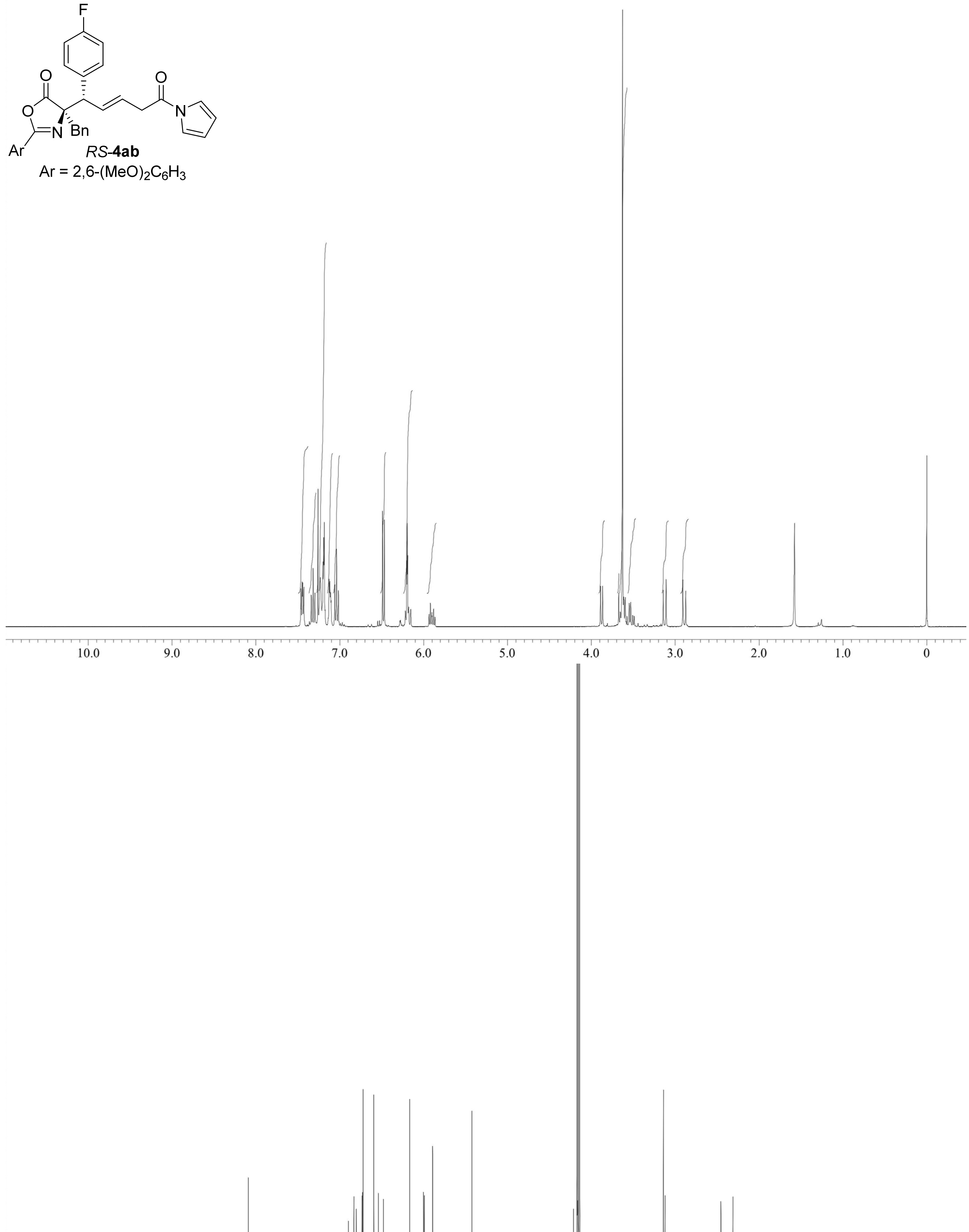

Supplementary Figure 37. ^{1}H and ^{13}C NMR spectra of *RS*-**4ab**

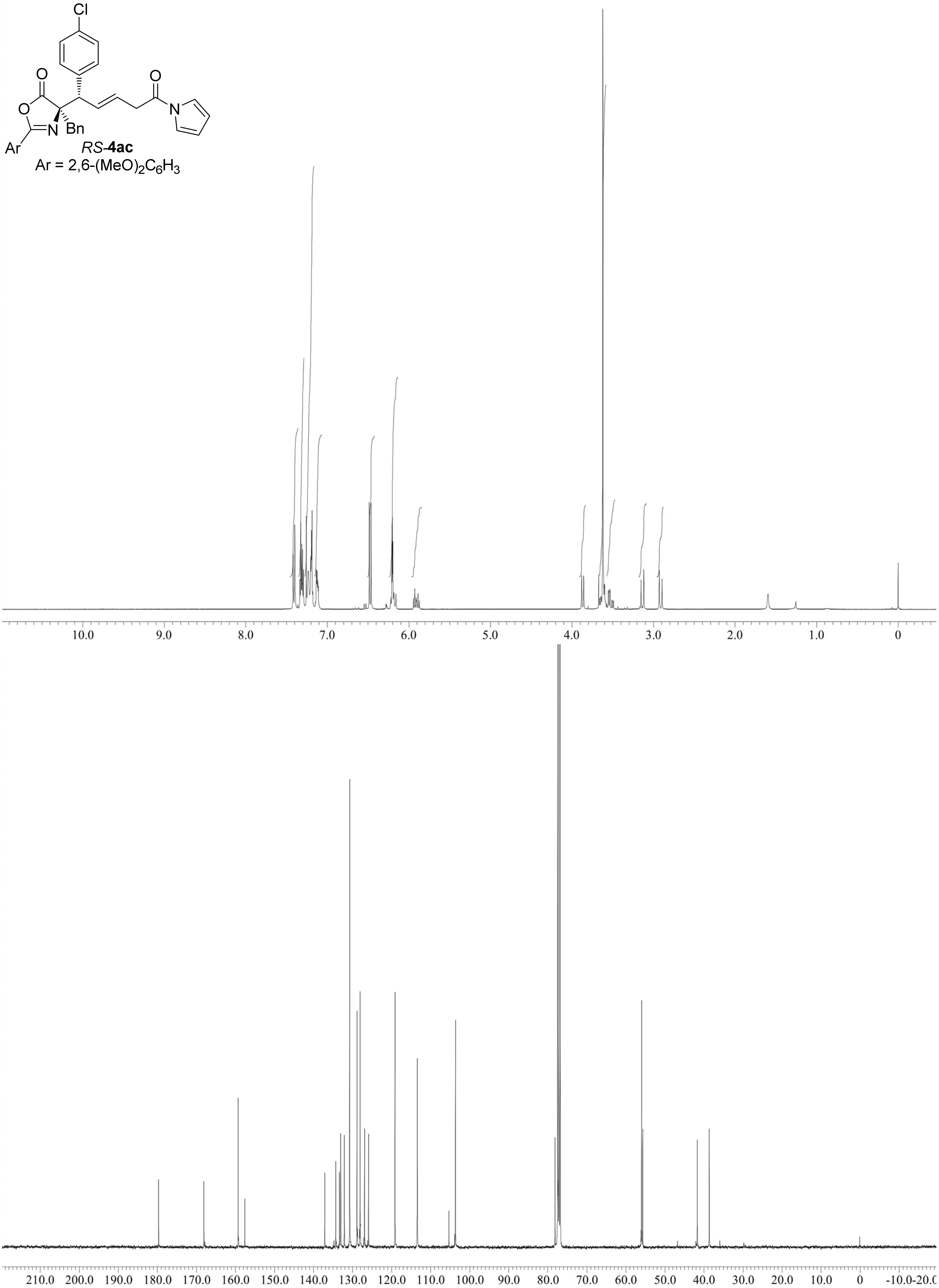

Supplementary Figure 38. ^{1}H and ^{13}C NMR spectra of *RS*-**4ac**

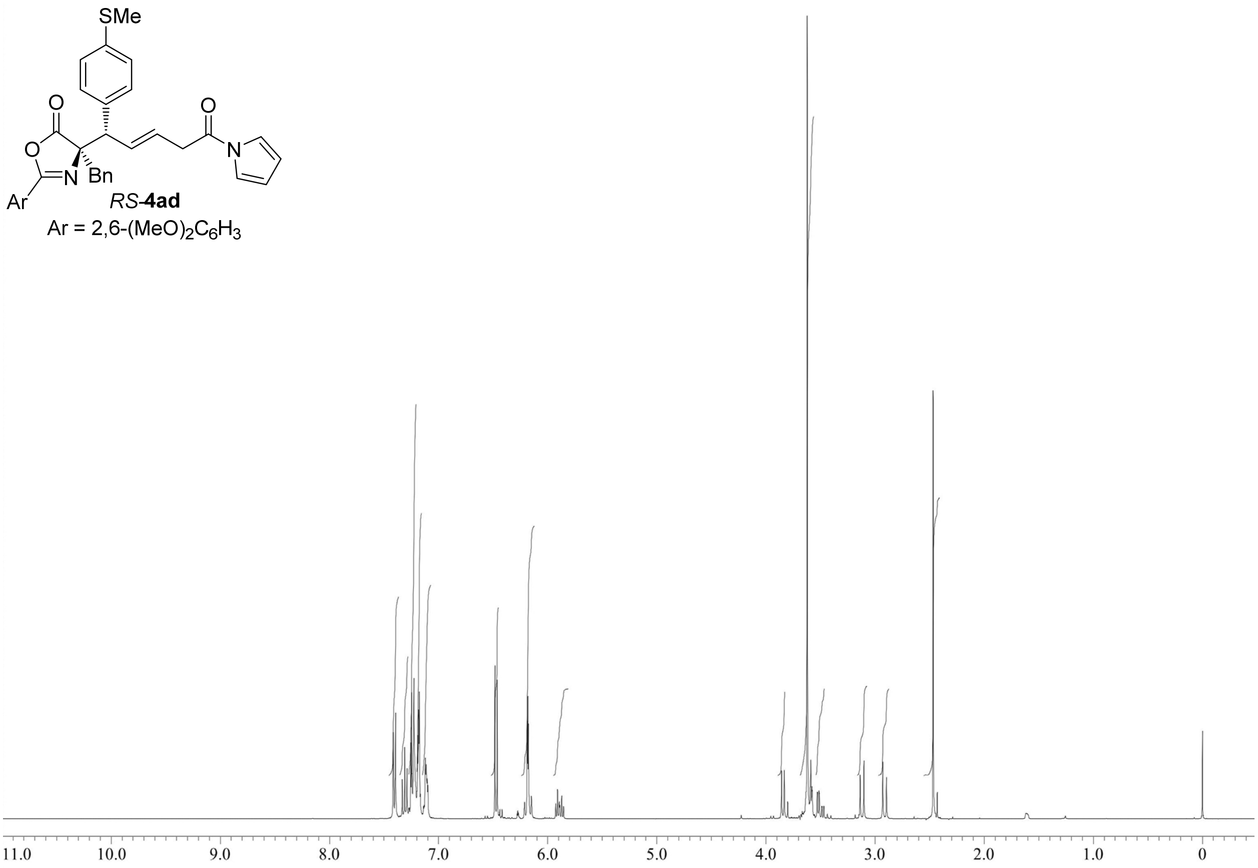

RS-**4ad**

Ar = 2,6-$(MeO)_2C_6H_3$

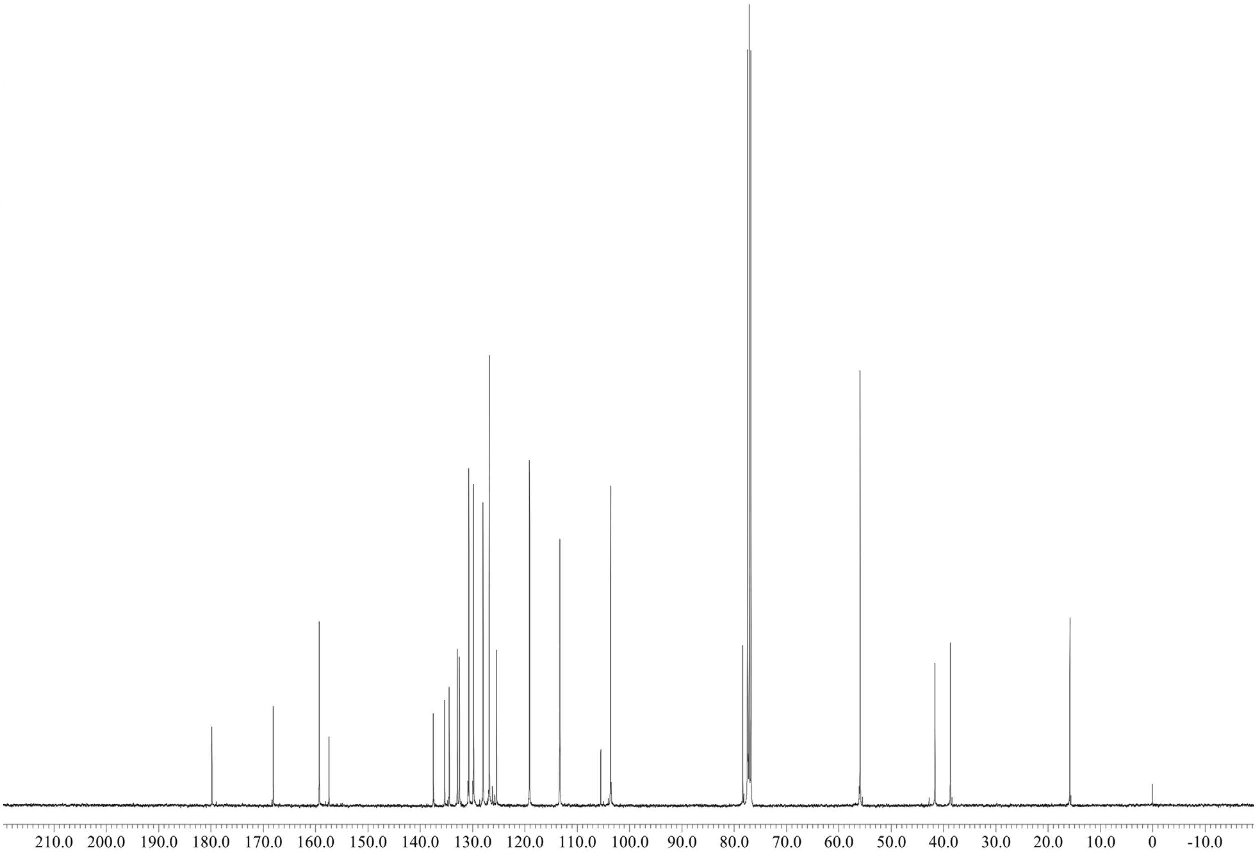

Supplementary Figure 39. ^{1}H and ^{13}C NMR spectra of *RS*-**4ad**

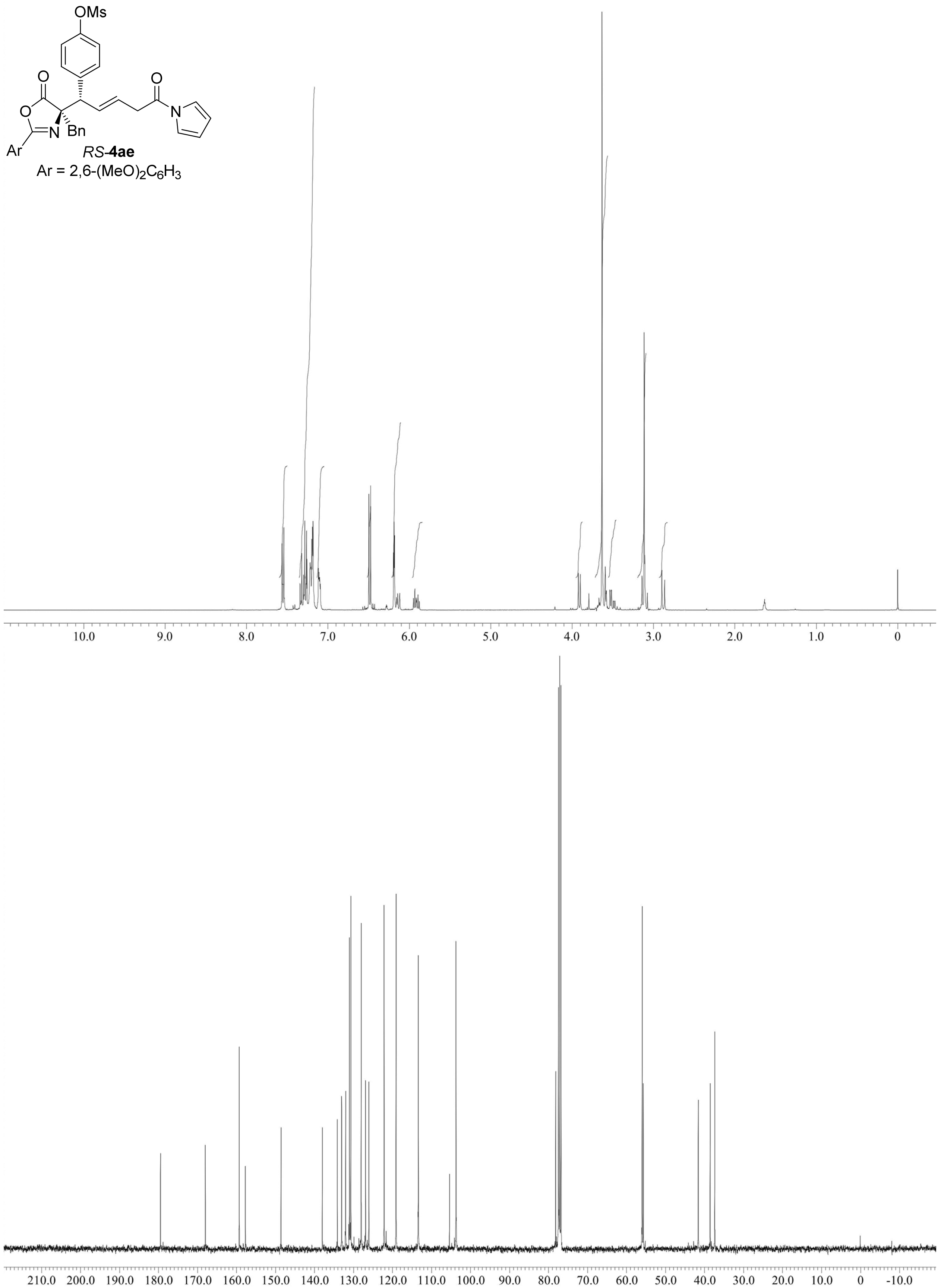

Supplementary Figure 40. ^{1}H and ^{13}C NMR spectra of *RS*-**4ae**

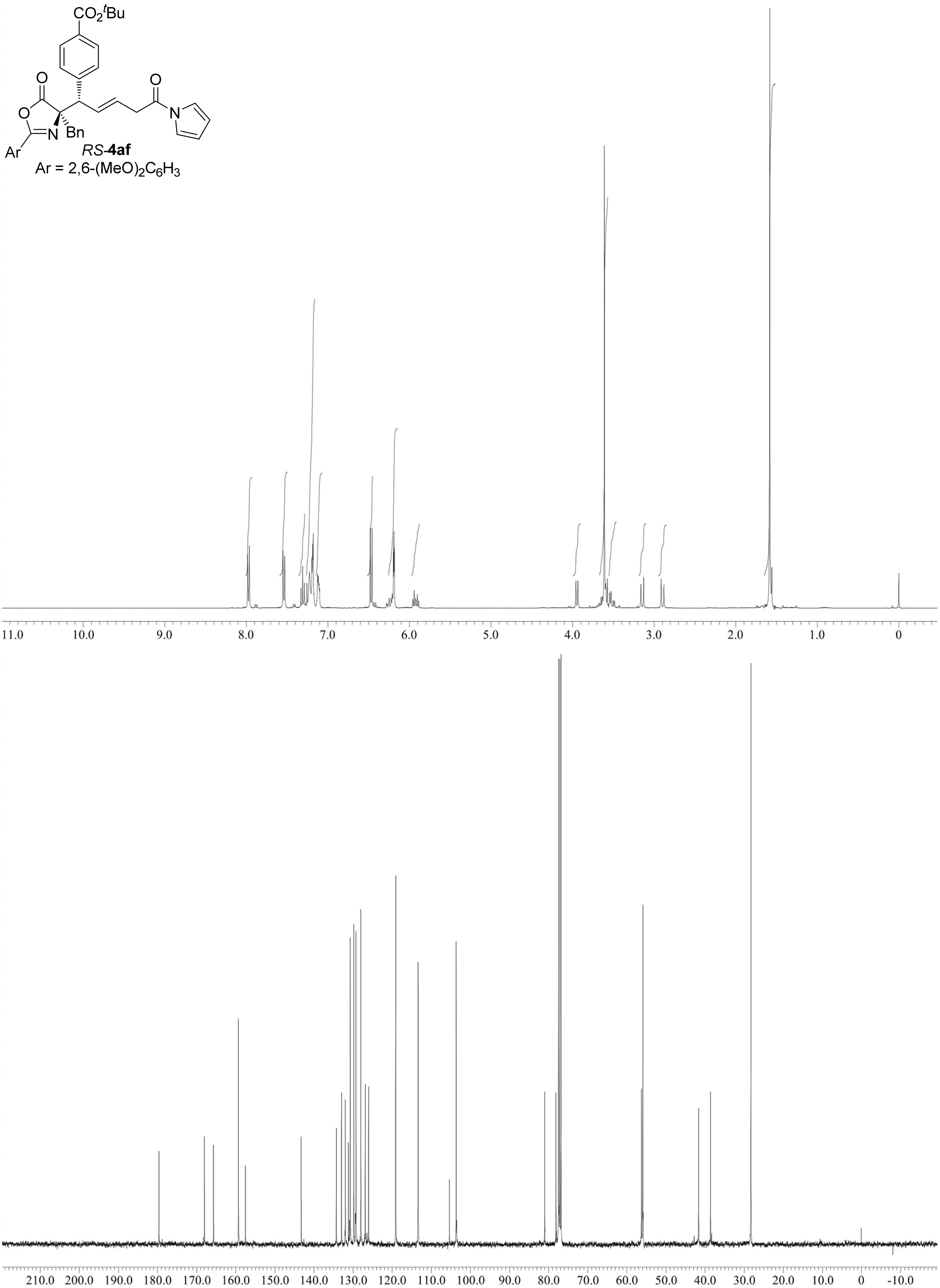

Supplementary Figure 41. ^{1}H and ^{13}C NMR spectra of *RS*-**4af**

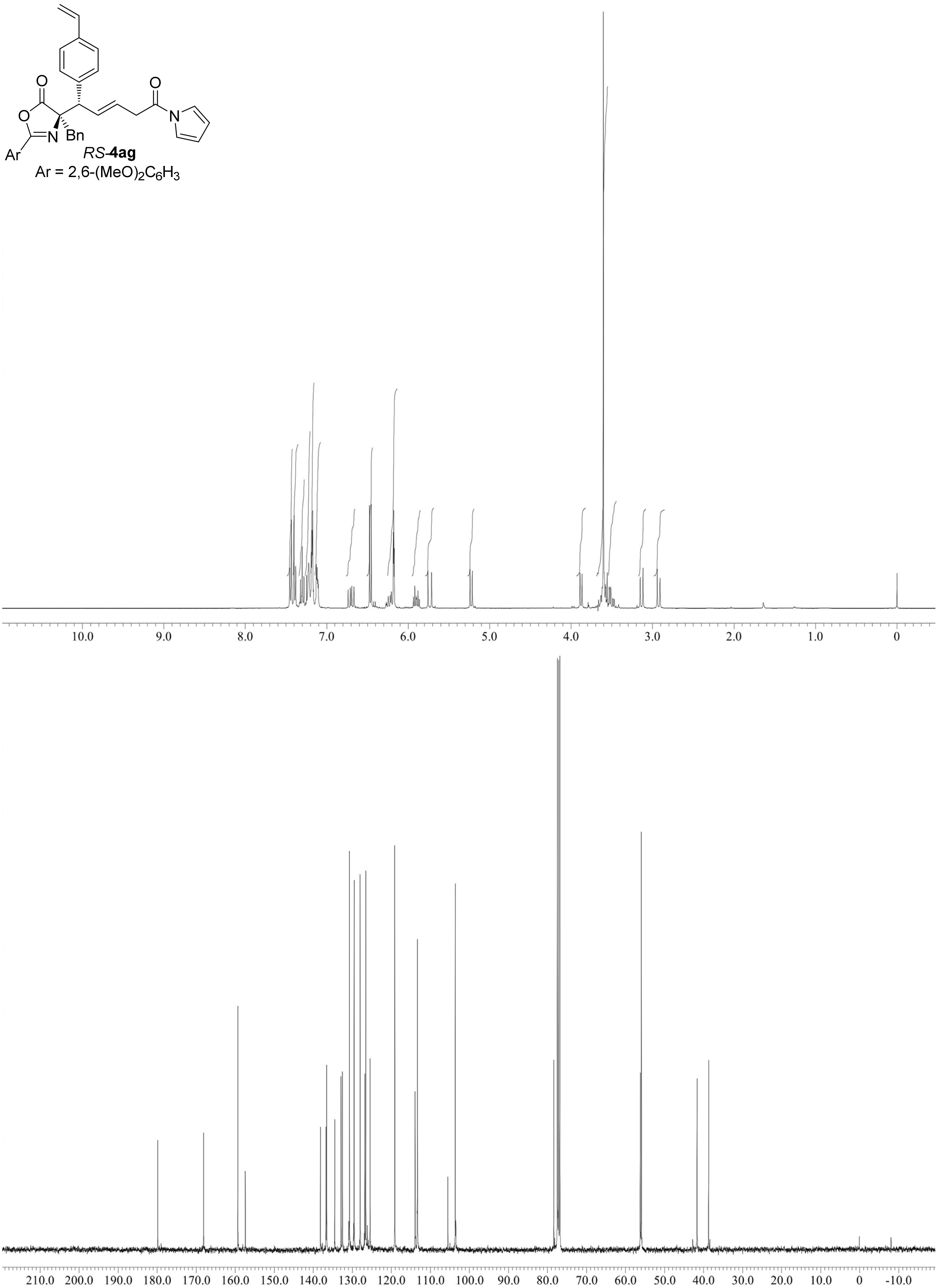

Supplementary Figure 42. ^{1}H and ^{13}C NMR spectra of *RS*-**4ag**

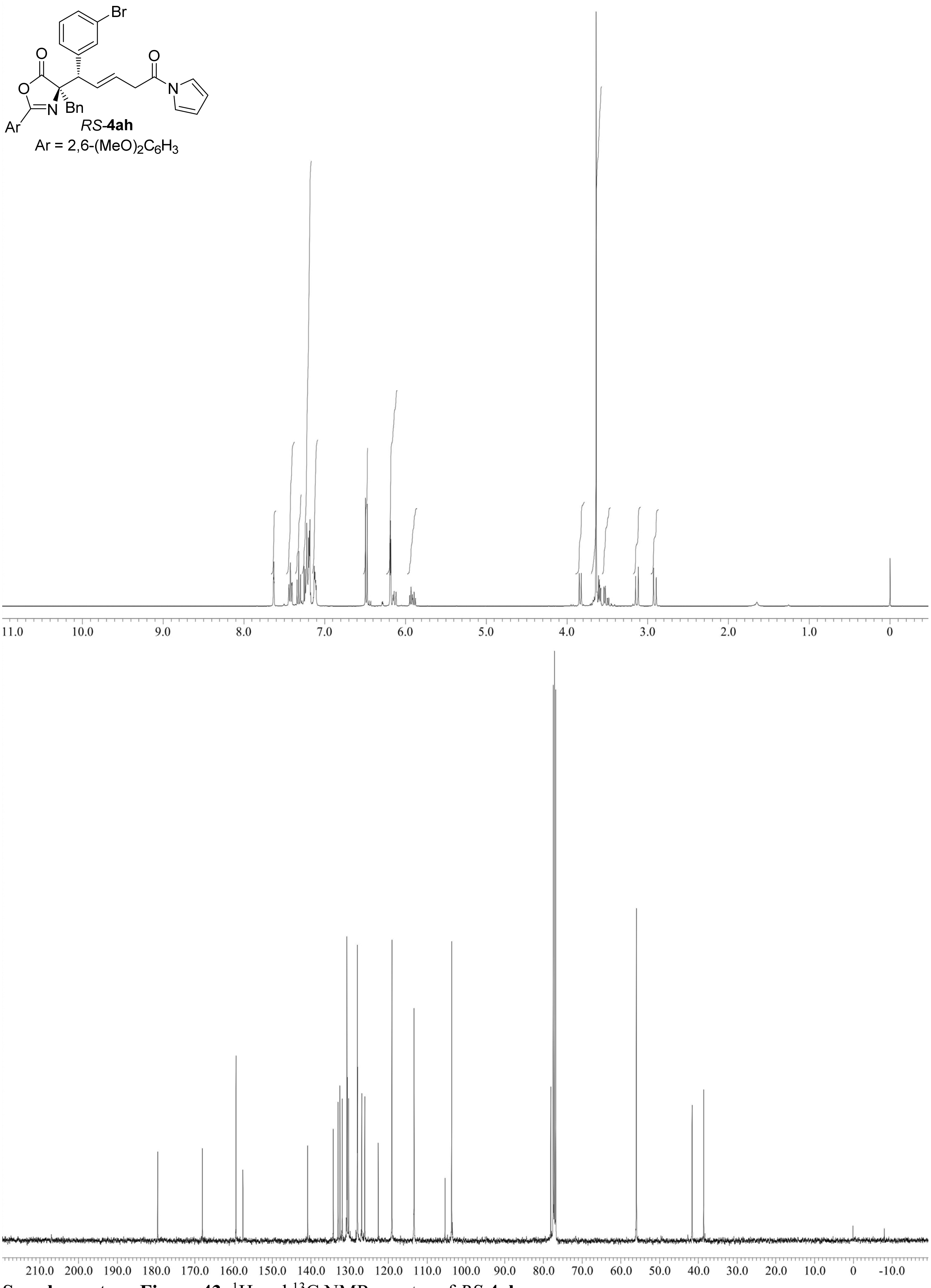

Supplementary Figure 43. ^{1}H and ^{13}C NMR spectra of *RS*-**4ah**

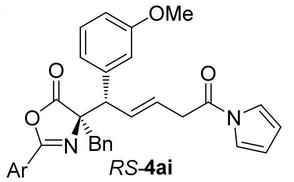

RS-**4ai**

Ar = 2,6-$(MeO)_2C_6H_3$

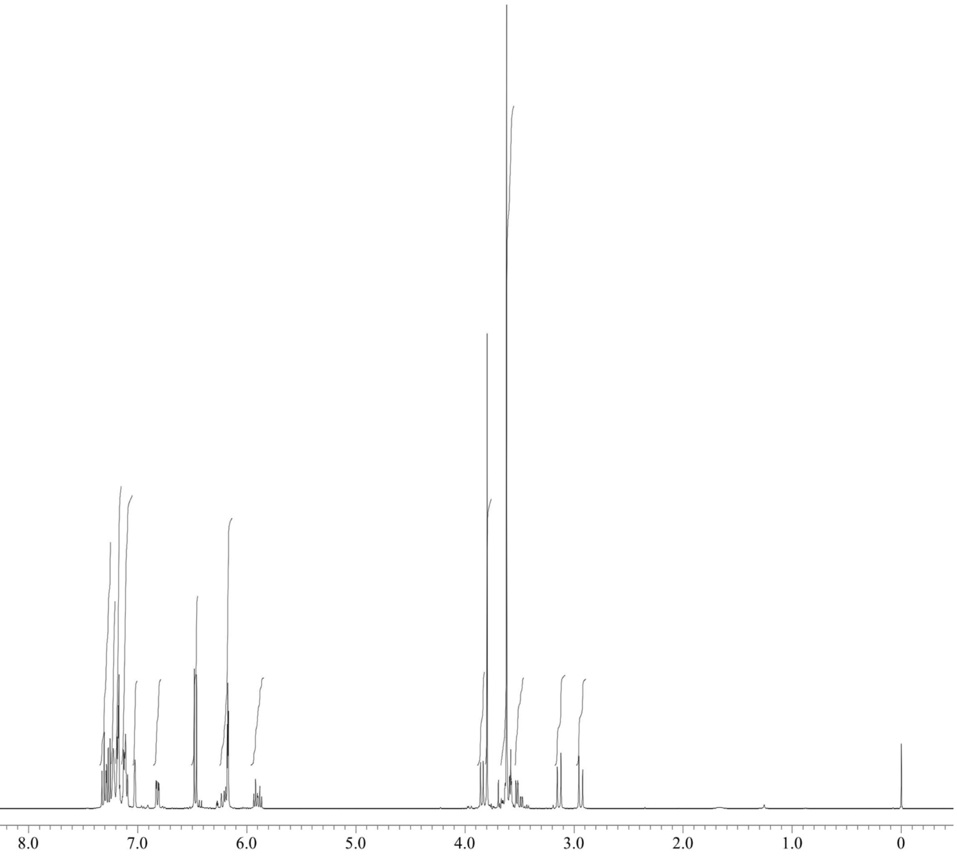

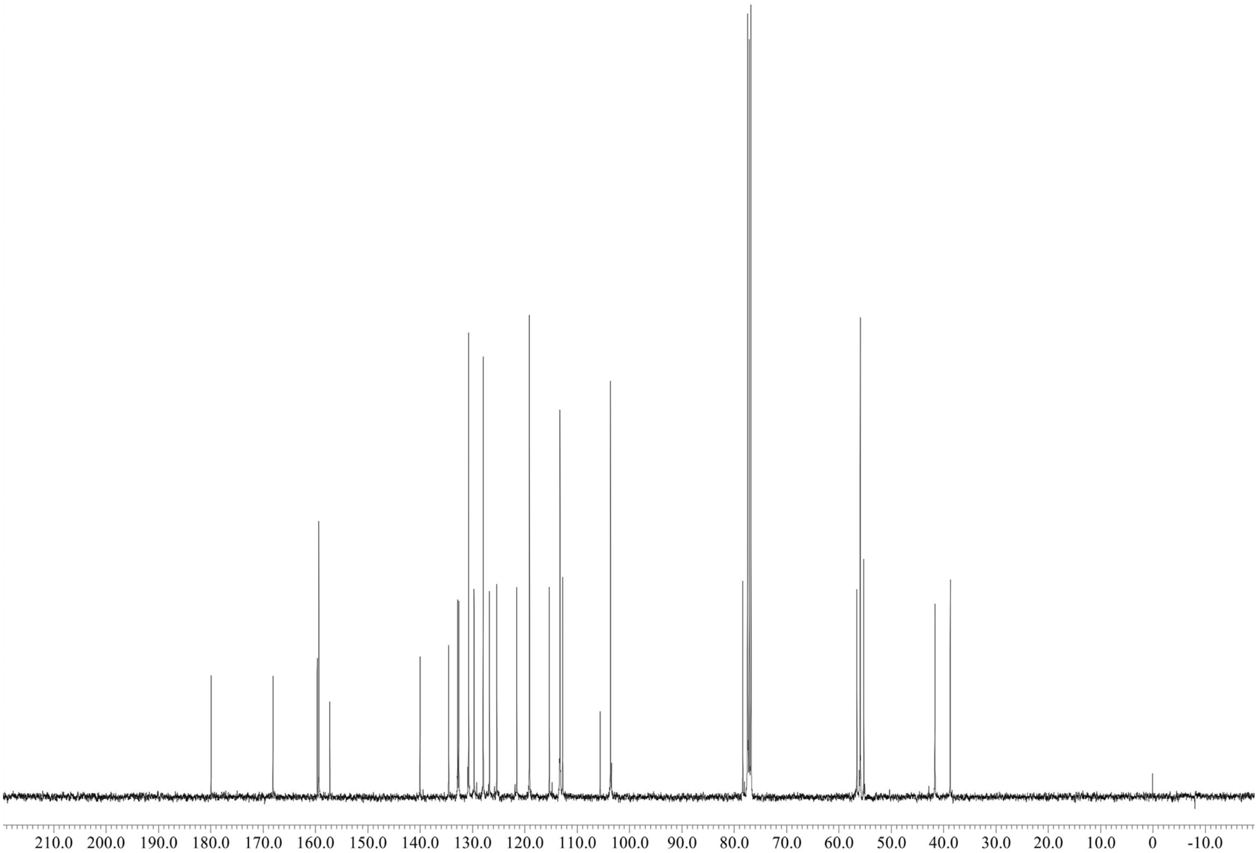

Supplementary Figure 44. ^{1}H and ^{13}C NMR spectra of *RS*-**4ai**

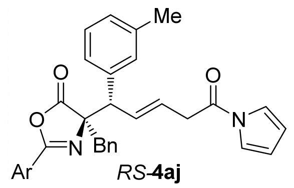

RS-**4aj**
Ar = 2,6-$(MeO)_2C_6H_3$

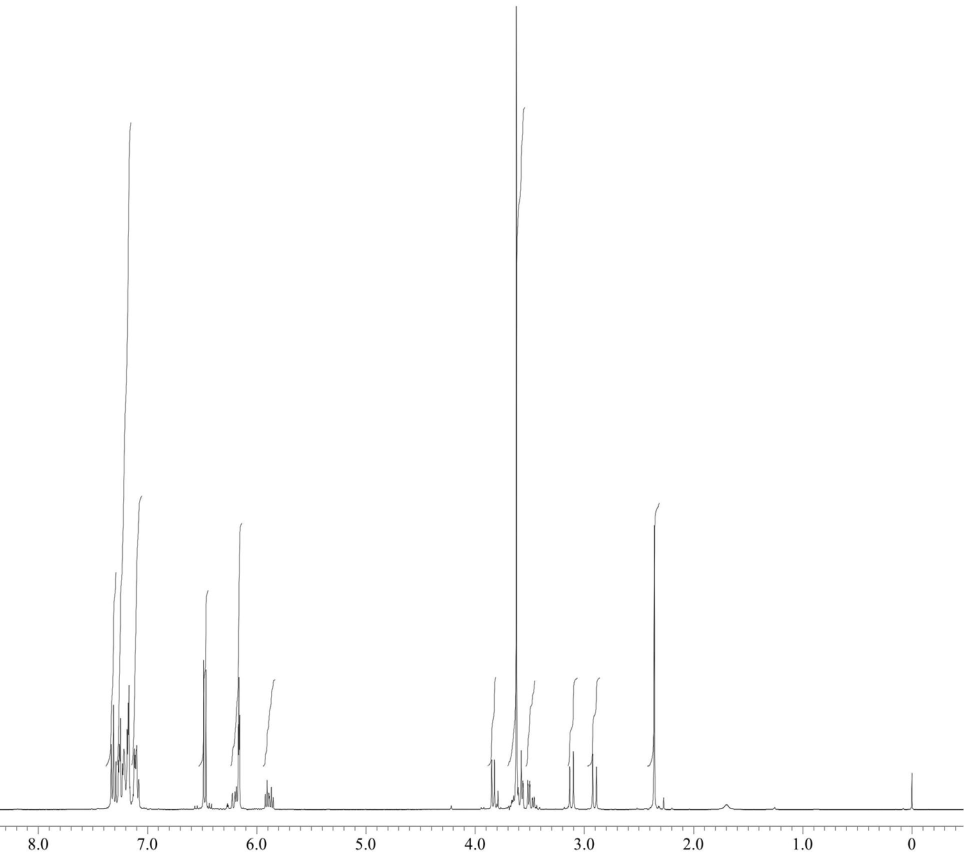

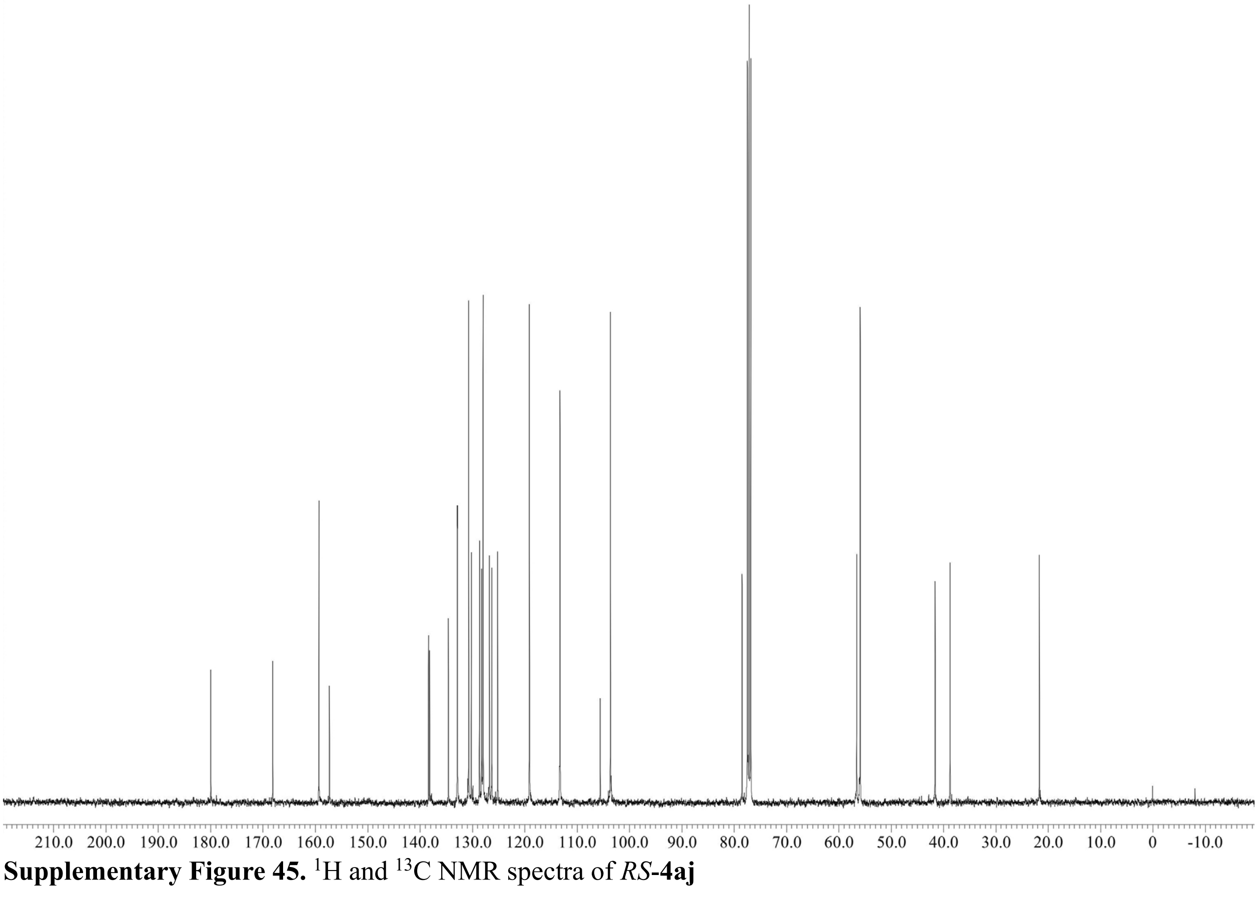

Supplementary Figure 45. ^{1}H and ^{13}C NMR spectra of *RS*-**4aj**

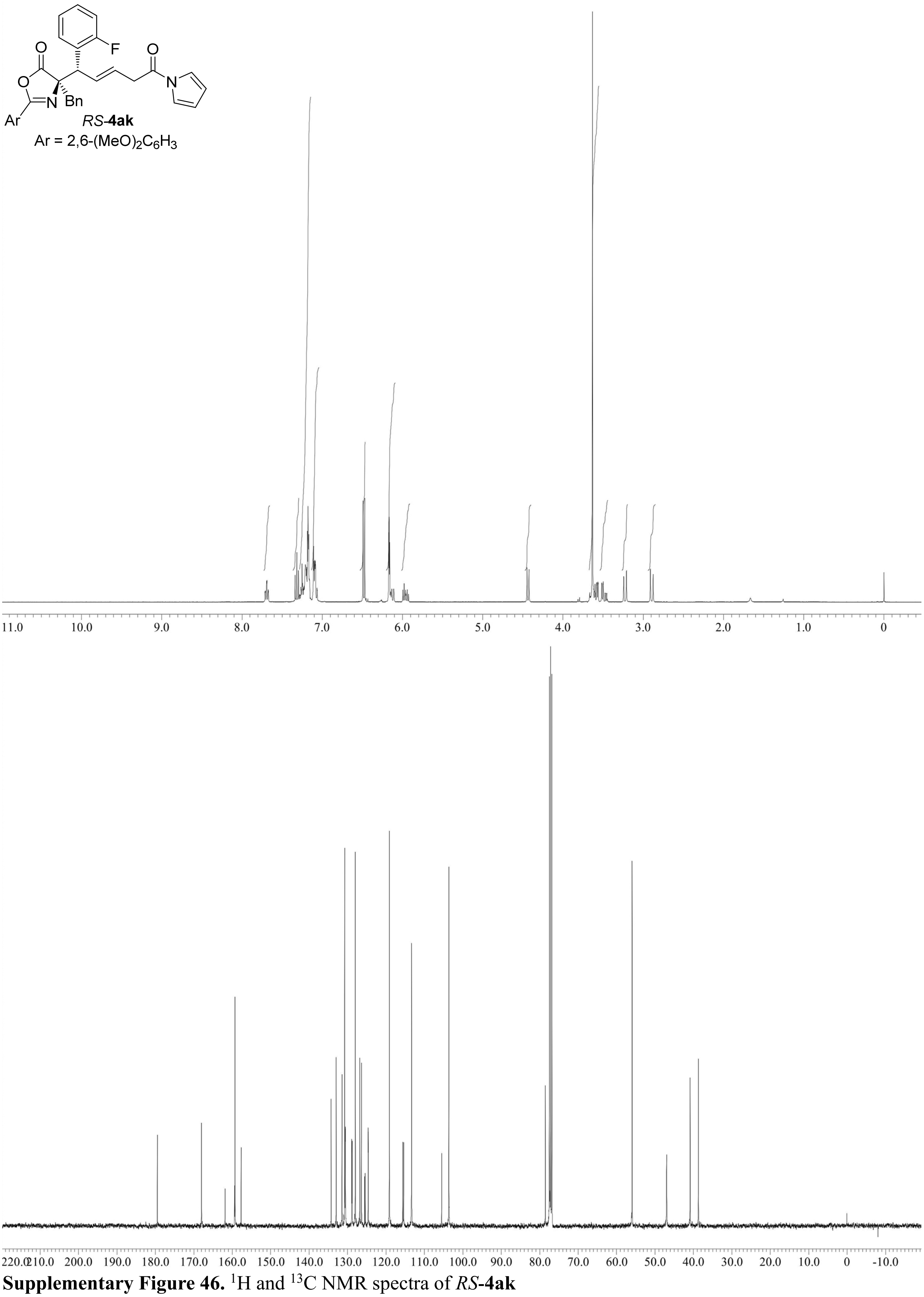

Supplementary Figure 46. ^{1}H and ^{13}C NMR spectra of *RS*-**4ak**

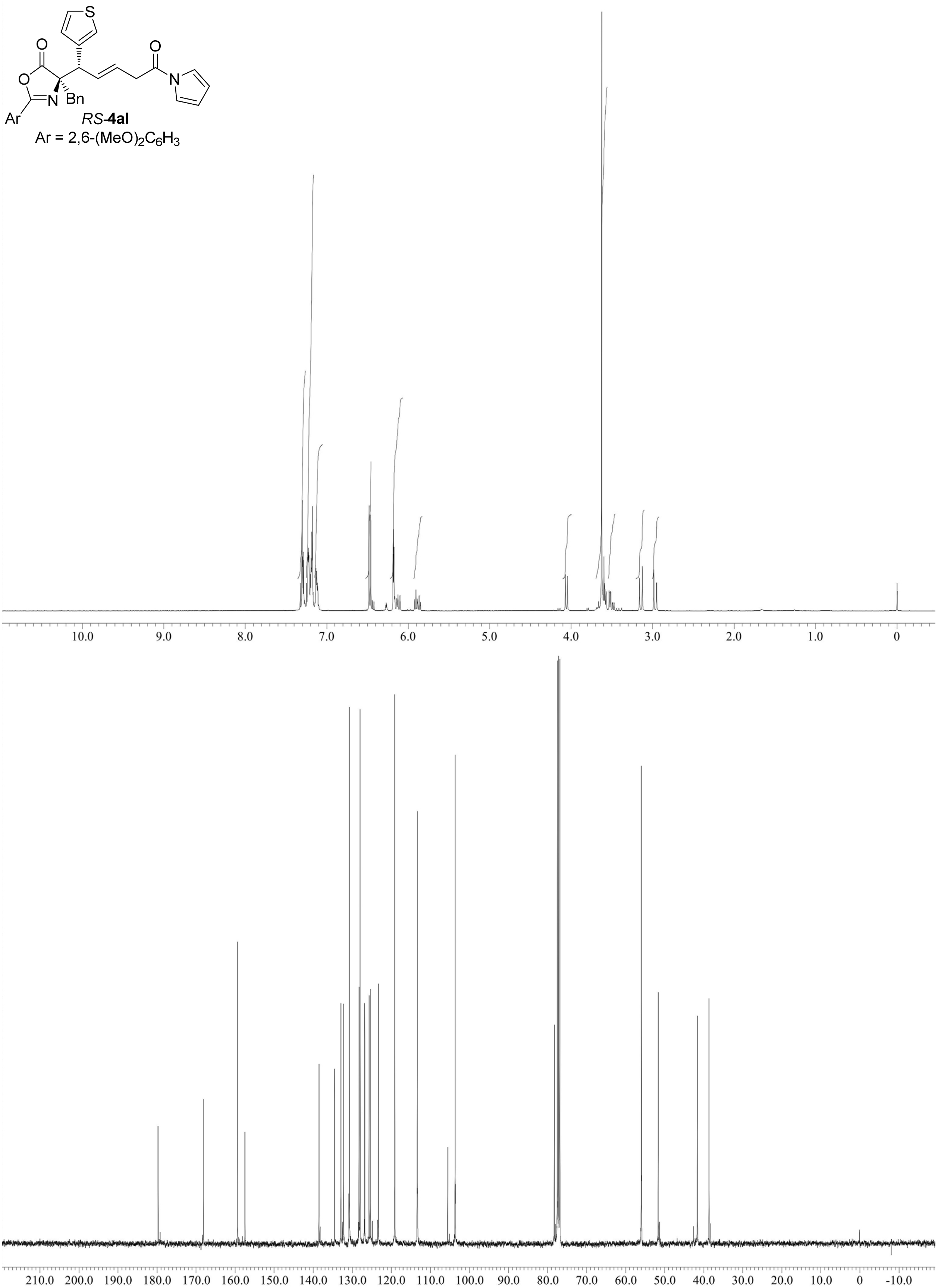

Supplementary Figure 47. ^{1}H and ^{13}C NMR spectra of *RS*-**4al**

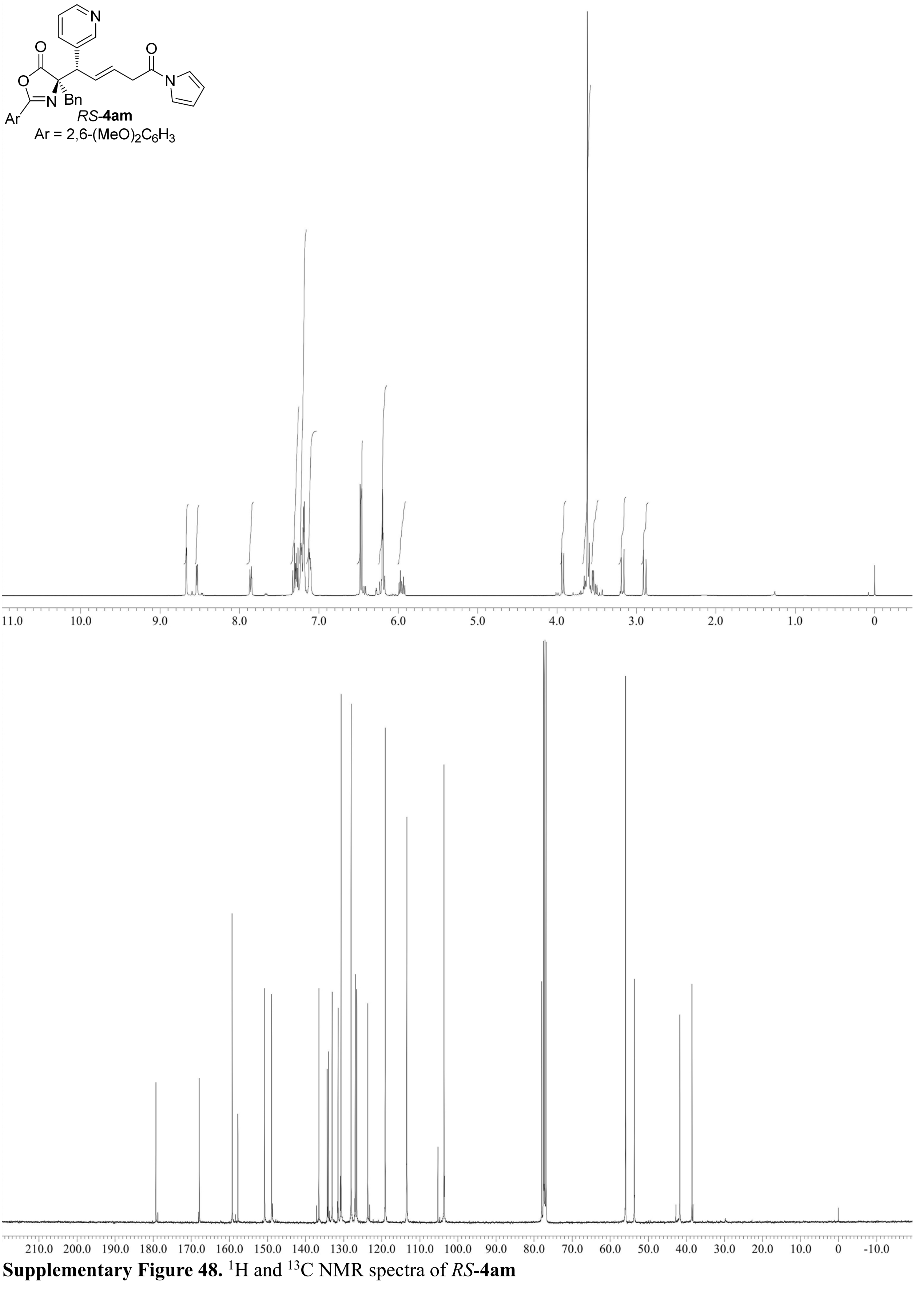

Supplementary Figure 48. ^{1}H and ^{13}C NMR spectra of *RS*-**4am**

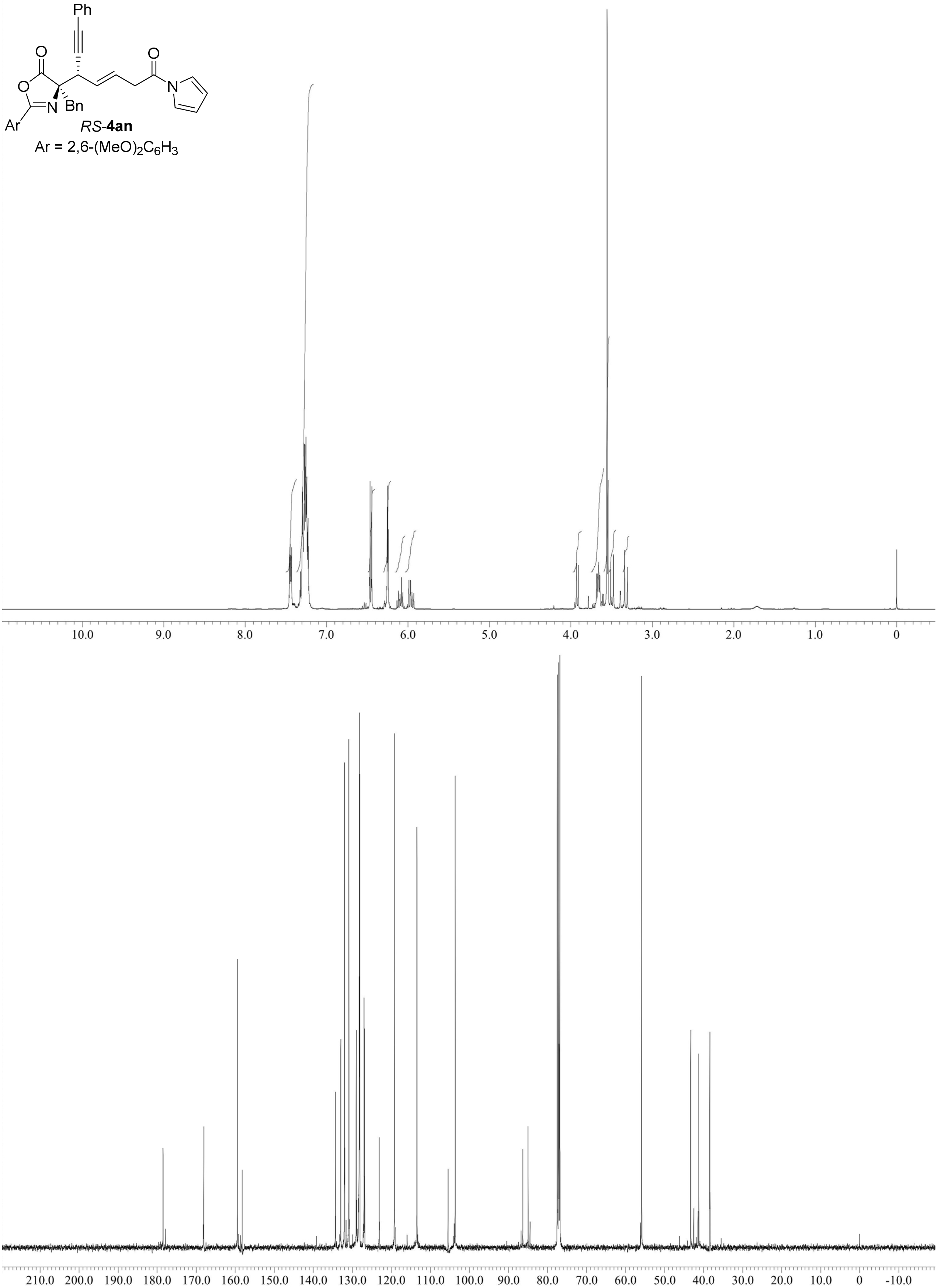

Supplementary Figure 49. ^{1}H and ^{13}C NMR spectra of *RS*-**4an**

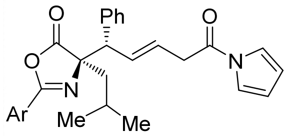

RS-**4ba**
Ar = 2,6-$(MeO)_2C_6H_3$

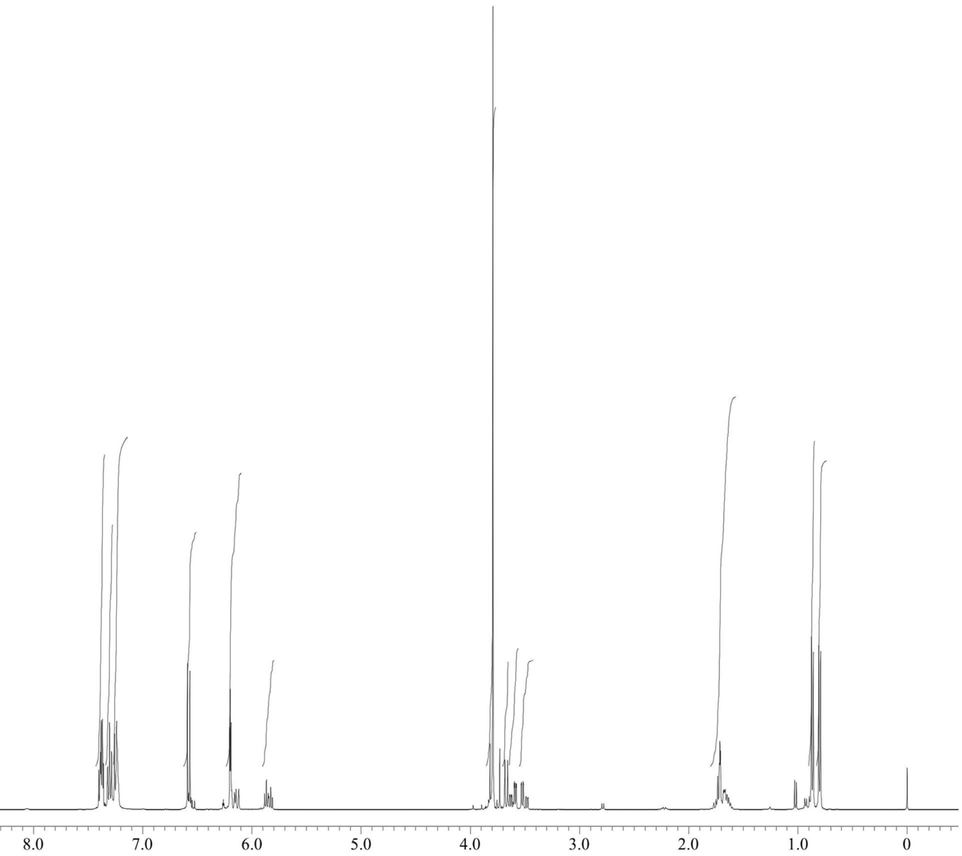

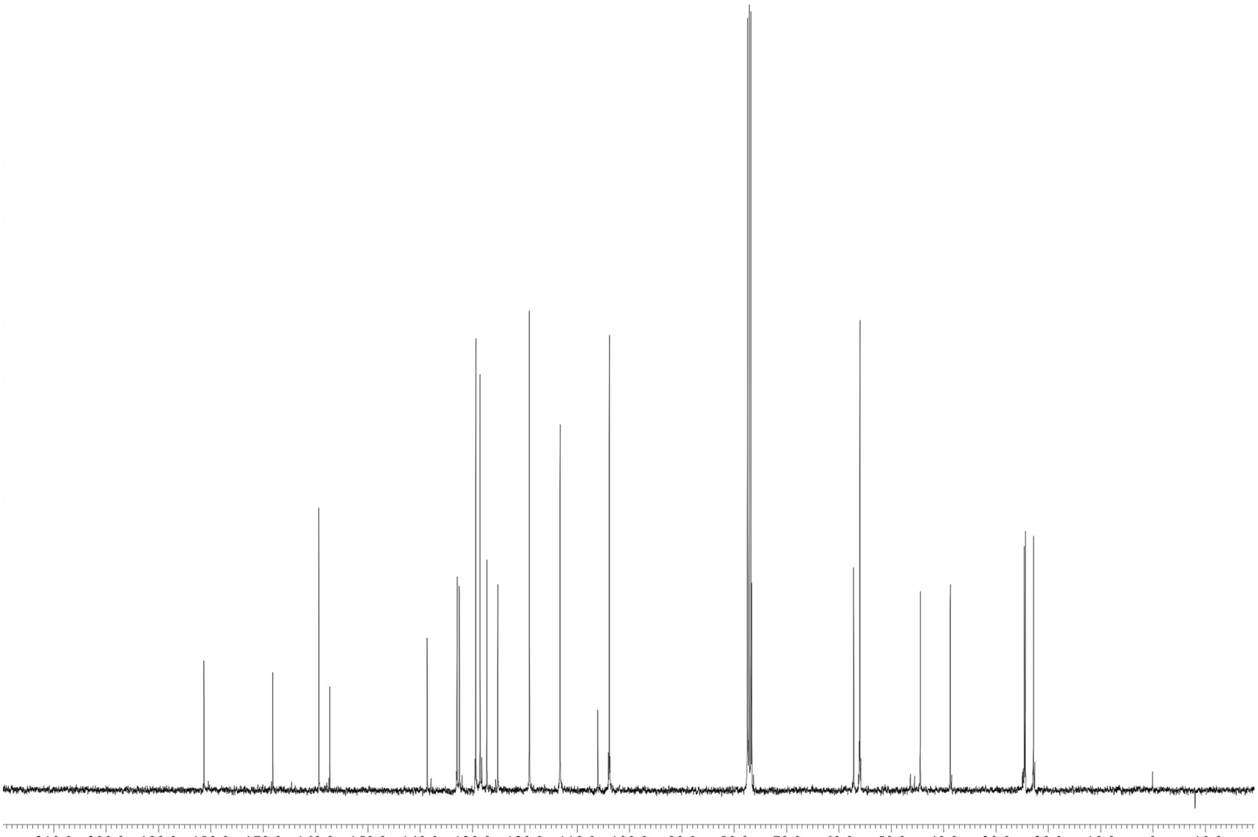

Supplementary Figure 50. ^{1}H and ^{13}C NMR spectra of *RS*-**4ba**

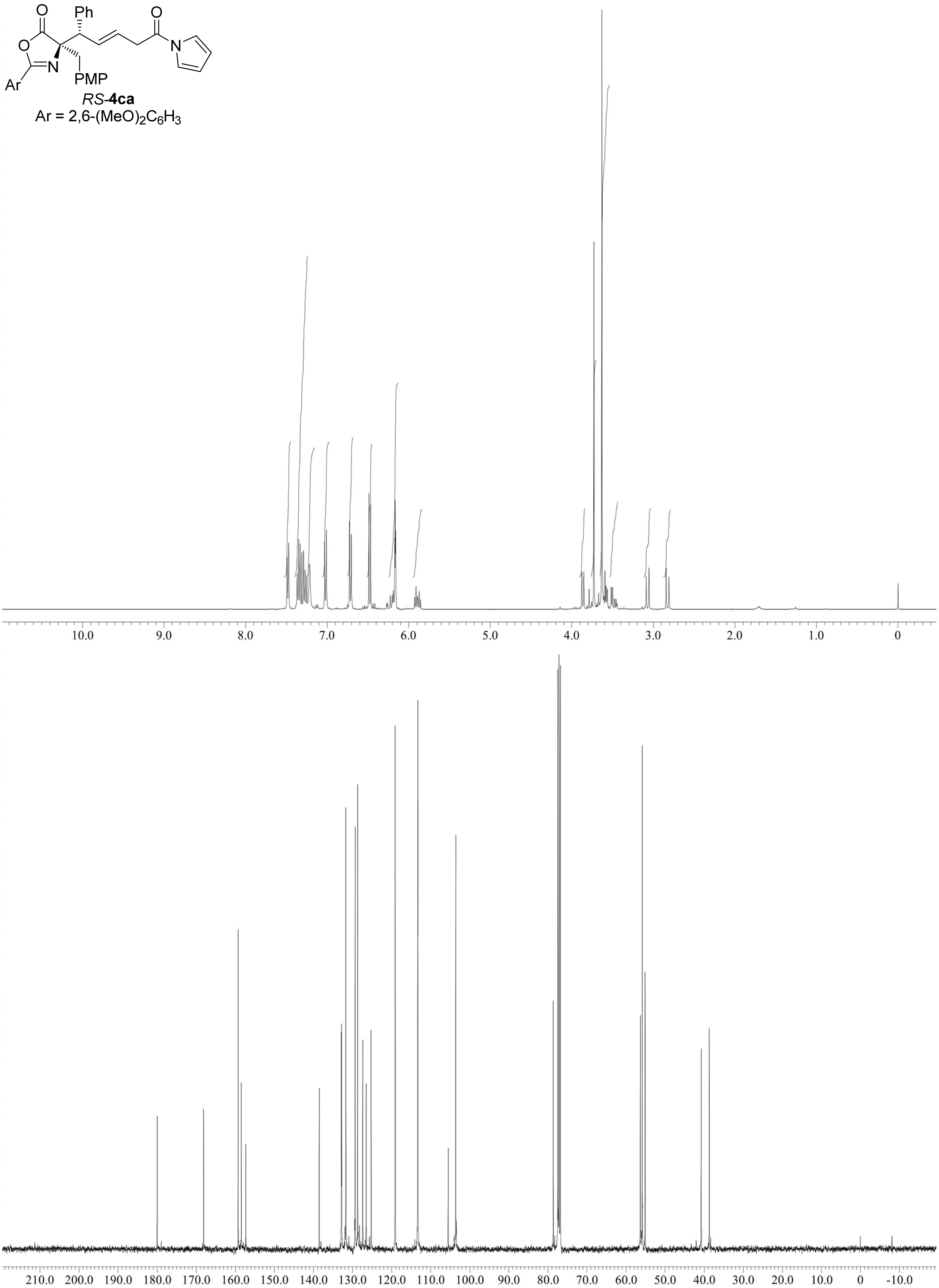

Supplementary Figure 51. ^{1}H and ^{13}C NMR spectra of *RS*-**4ca**

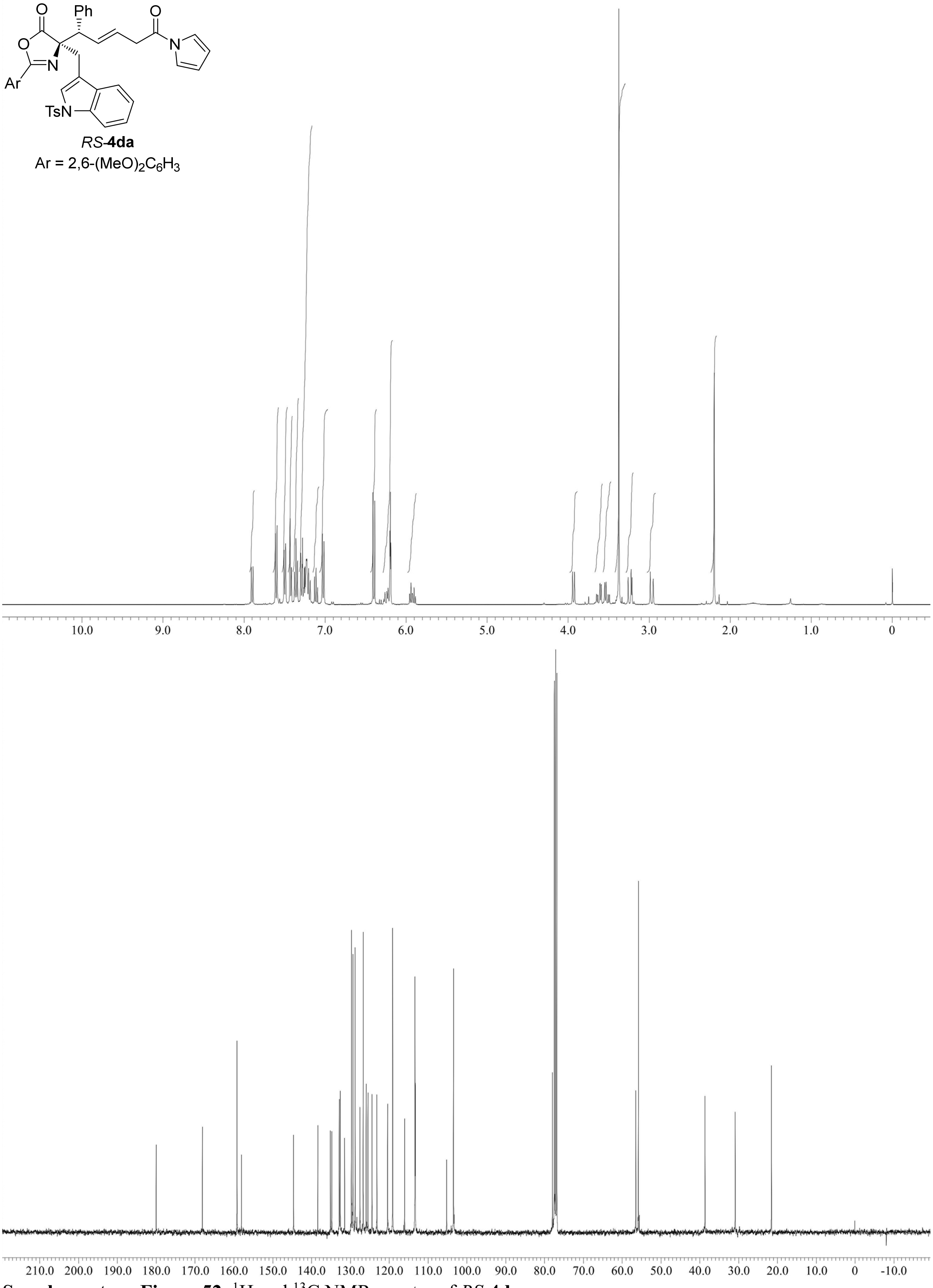

Supplementary Figure 52. ^{1}H and ^{13}C NMR spectra of *RS*-**4da**

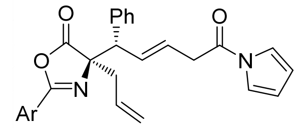

RS-**4ea**
Ar = 2,6-$(MeO)_2C_6H_3$

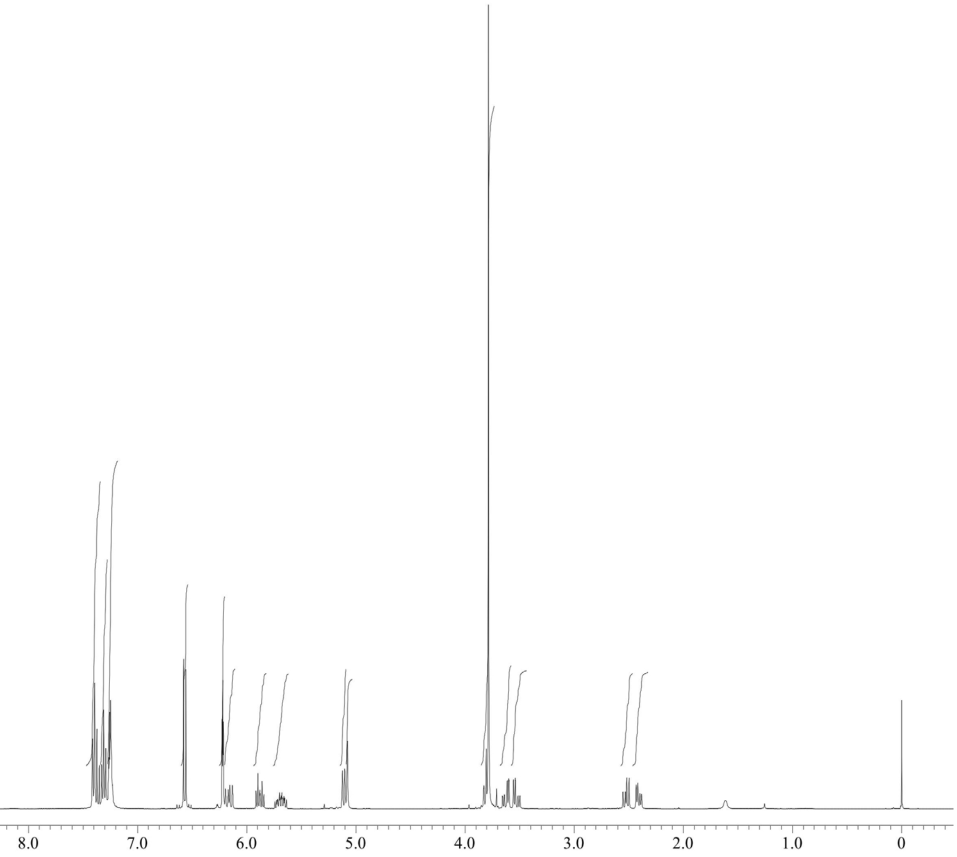

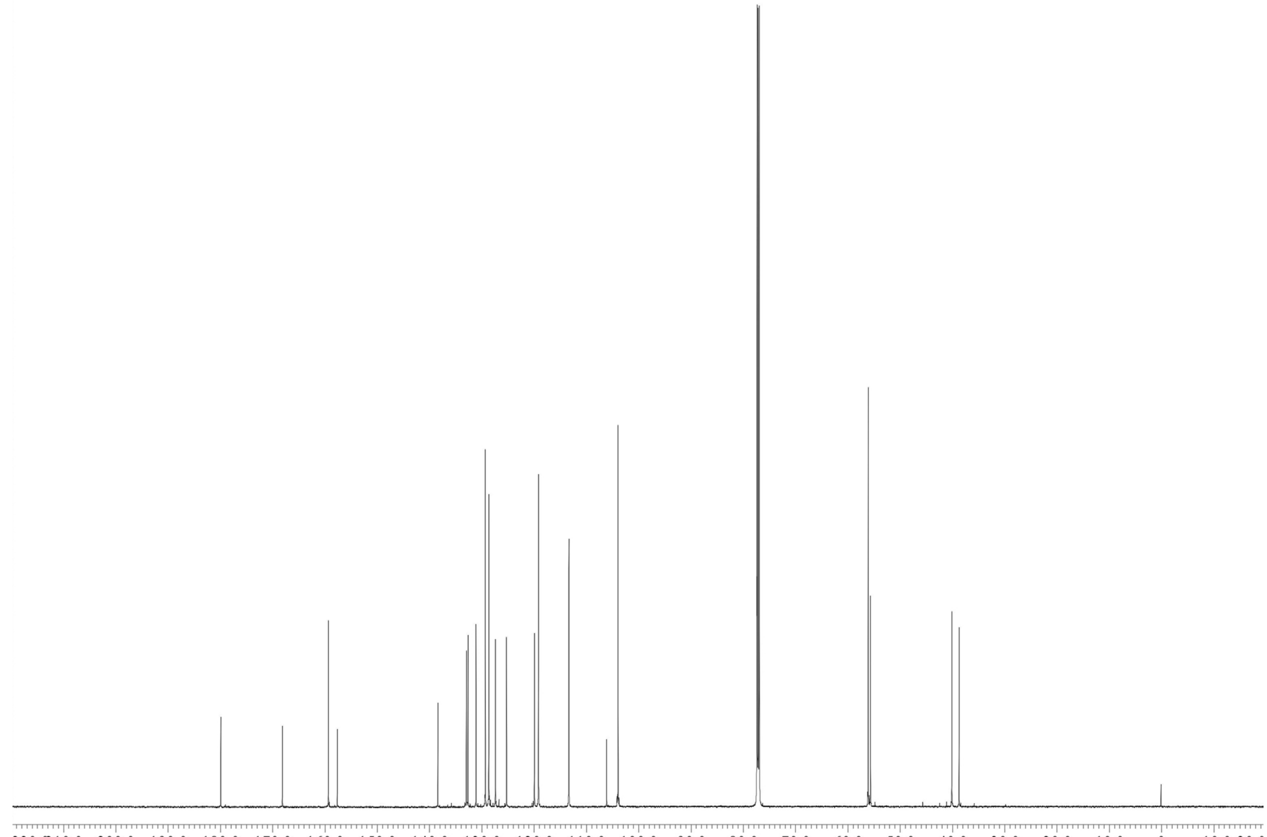

Supplementary Figure 53. ^{1}H and ^{13}C NMR spectra of *RS*-**4ea**

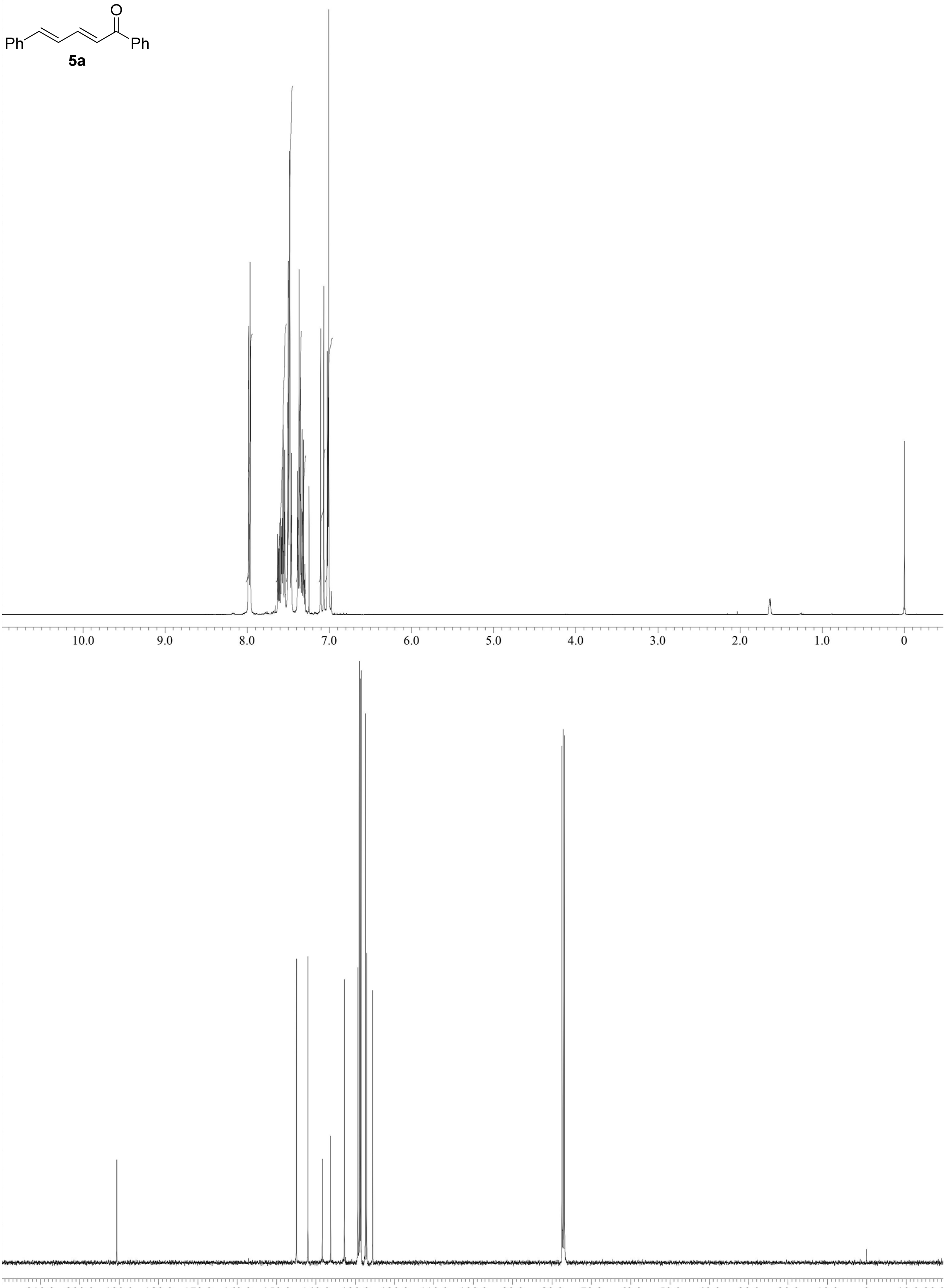

Supplementary Figure 54. ^{1}H and ^{13}C NMR spectra of **5a**

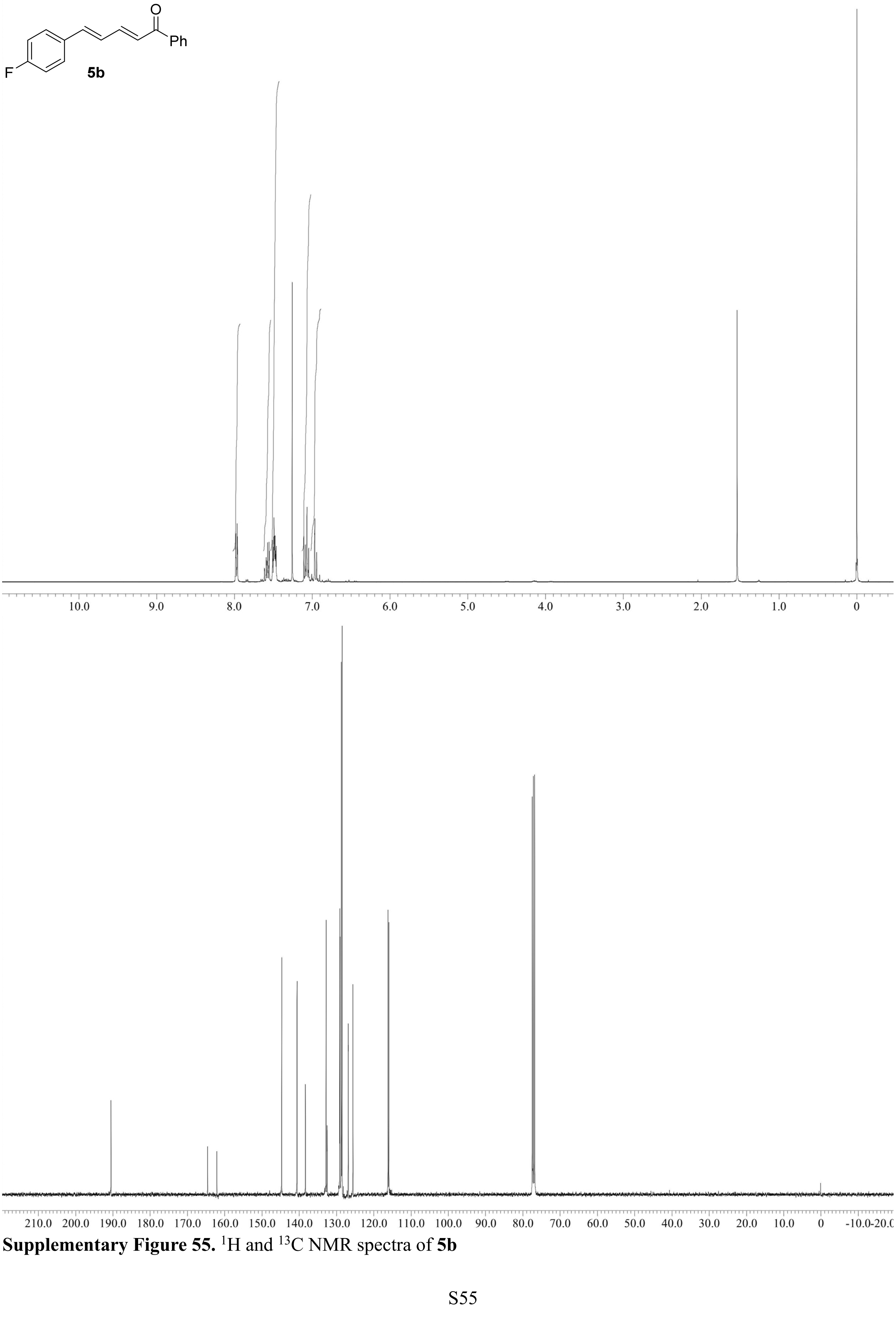

Supplementary Figure 55. ^{1}H and ^{13}C NMR spectra of **5b**

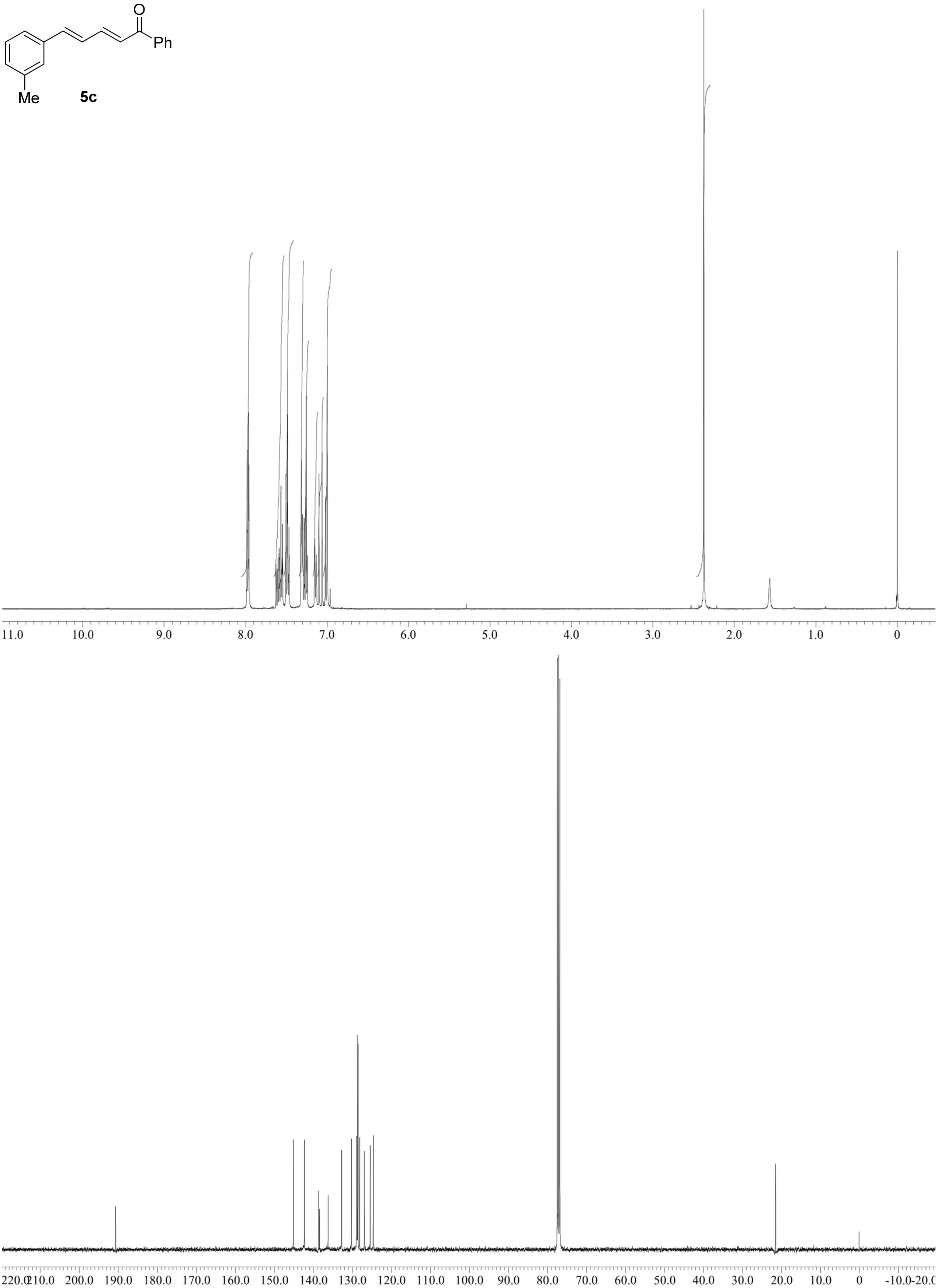

Supplementary Figure 56. ^{1}H and ^{13}C NMR spectra of **5c**

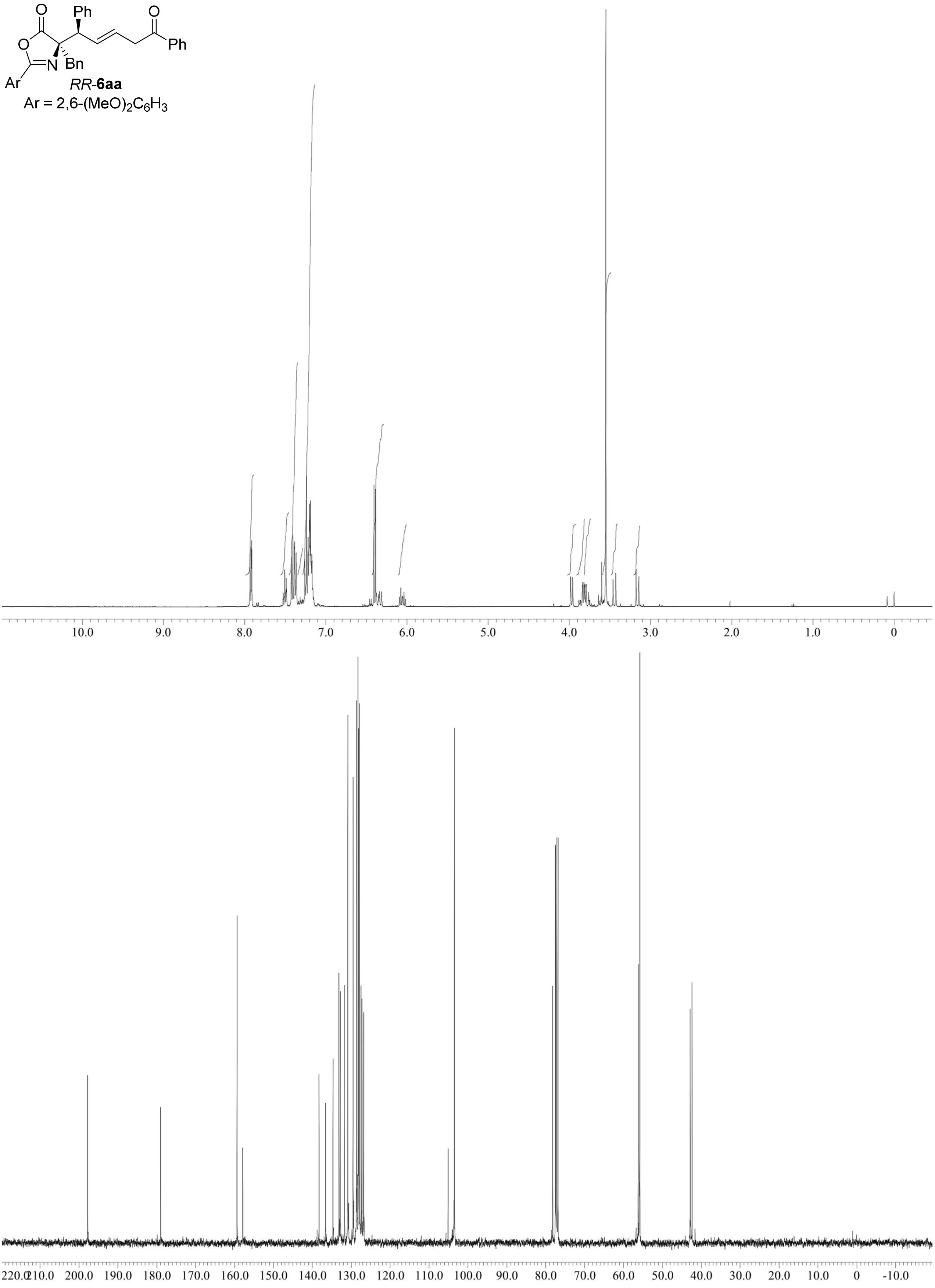

Supplementary Figure 57. ^{1}H and ^{13}C NMR spectra of *RR*-**6aa**

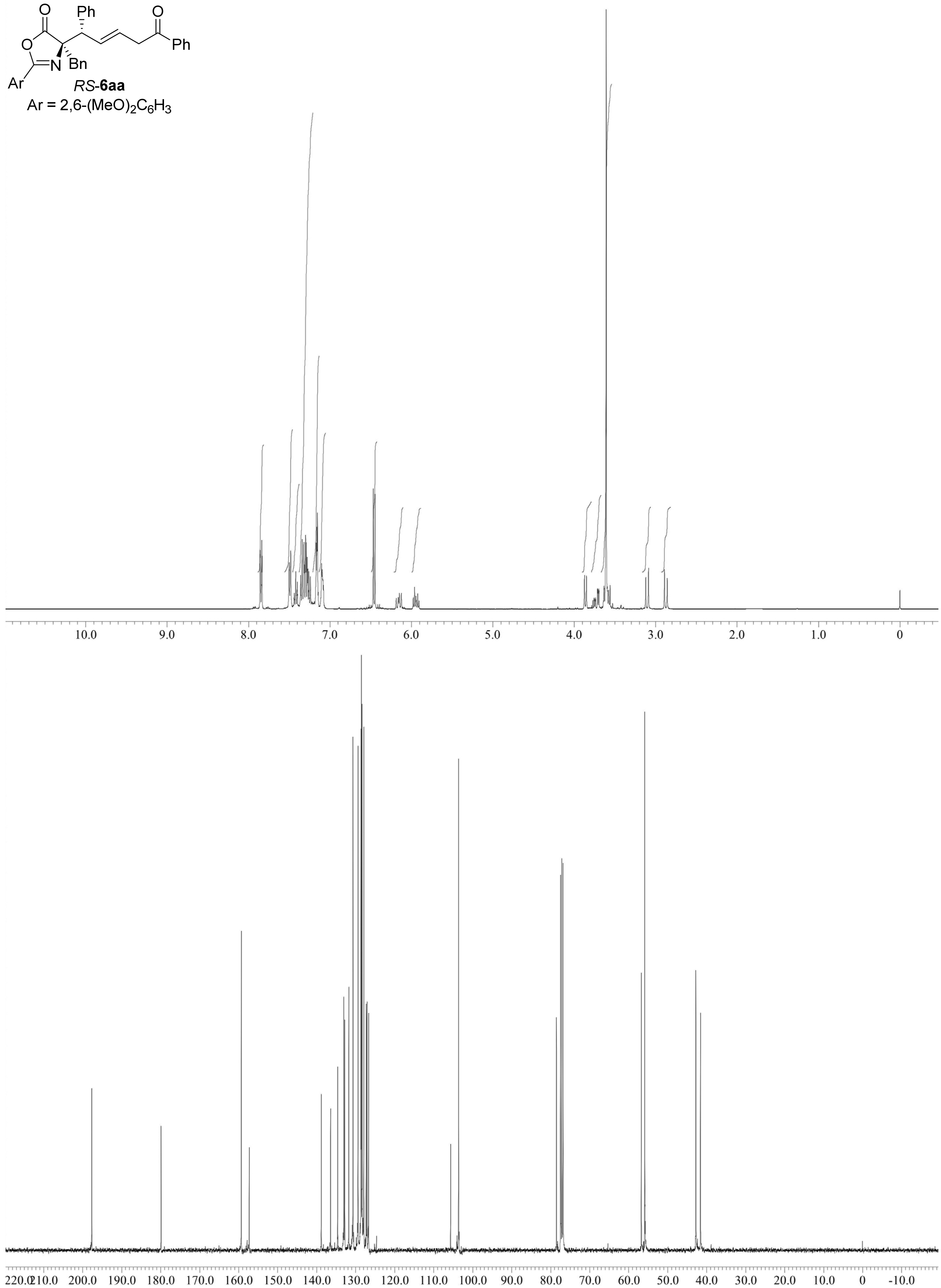

Supplementary Figure 58. ^{1}H and ^{13}C NMR spectra of *RS*-**6aa**

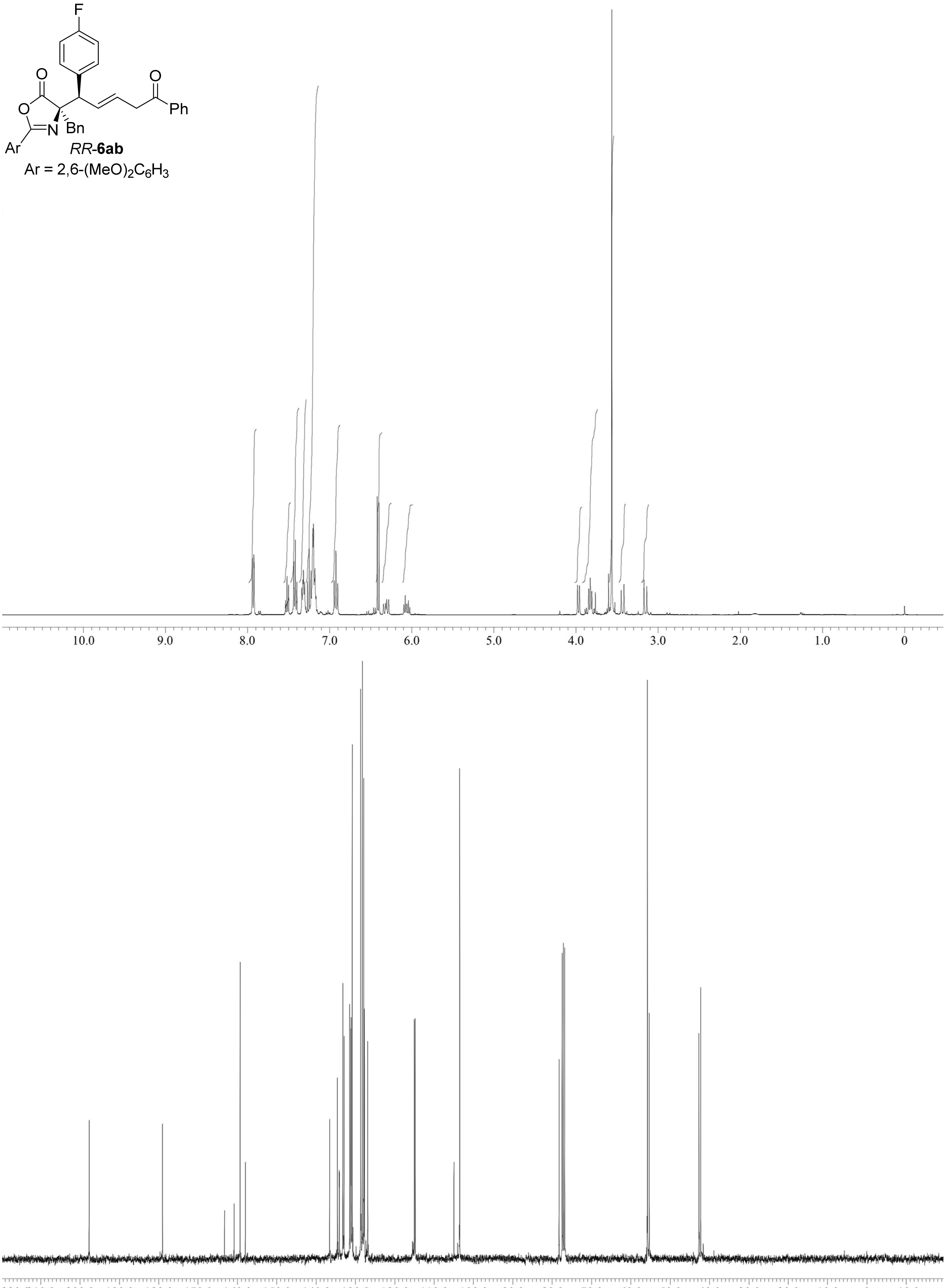

Supplementary Figure 59. ^{1}H and ^{13}C NMR spectra of *RR*-**6ab**

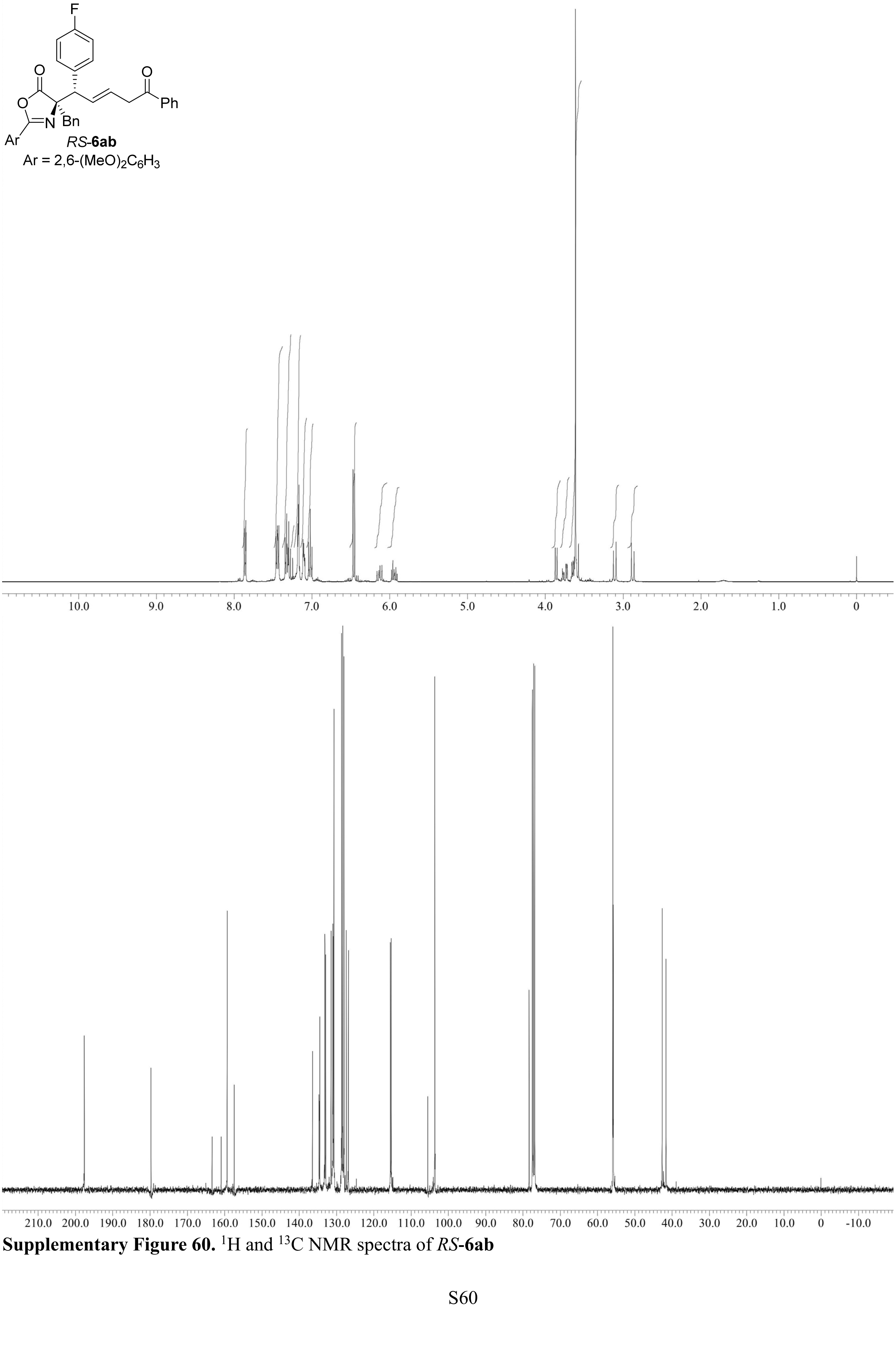

Supplementary Figure 60. ^{1}H and ^{13}C NMR spectra of *RS*-**6ab**

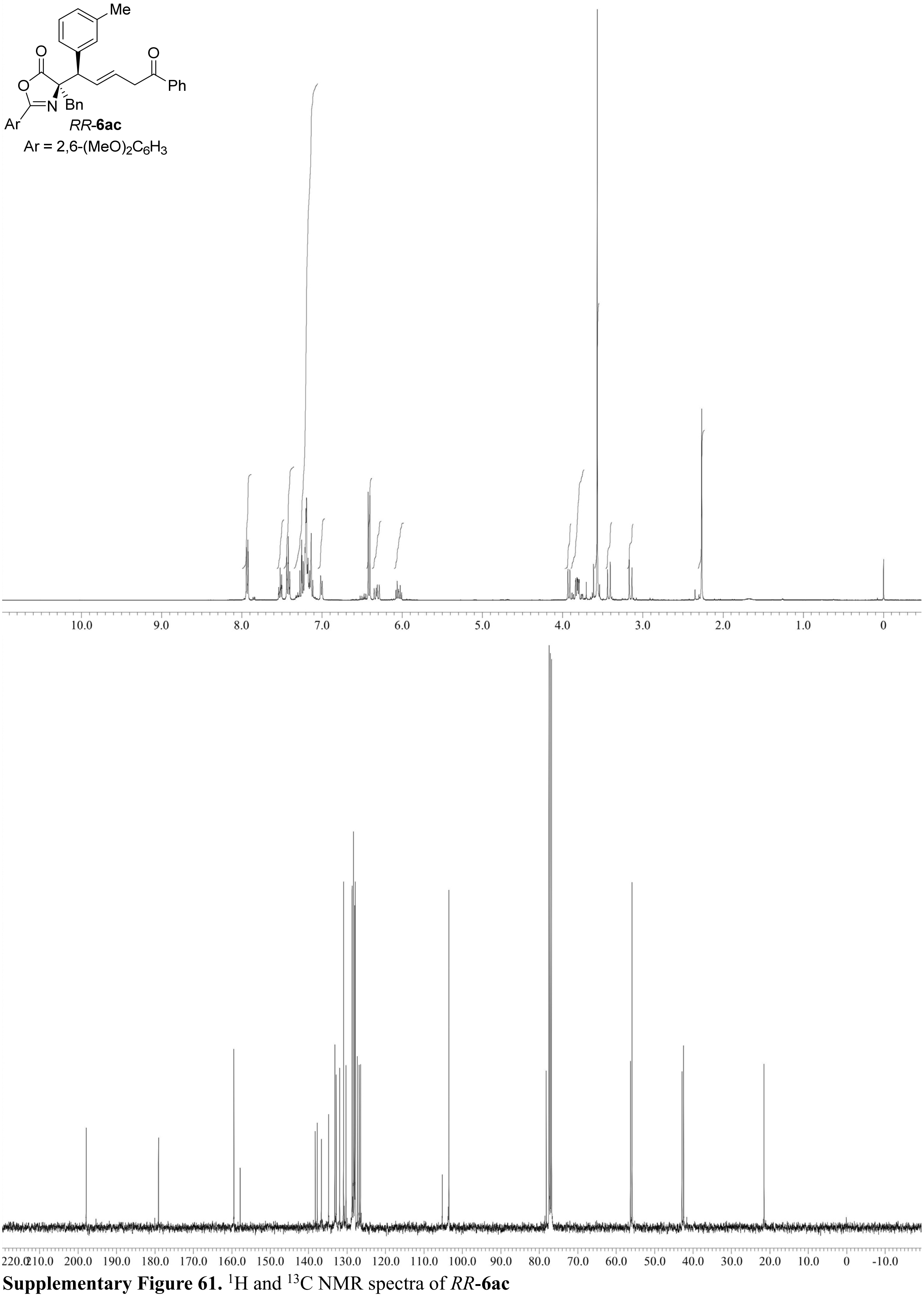

Supplementary Figure 61. ^{1}H and ^{13}C NMR spectra of *RR*-**6ac**

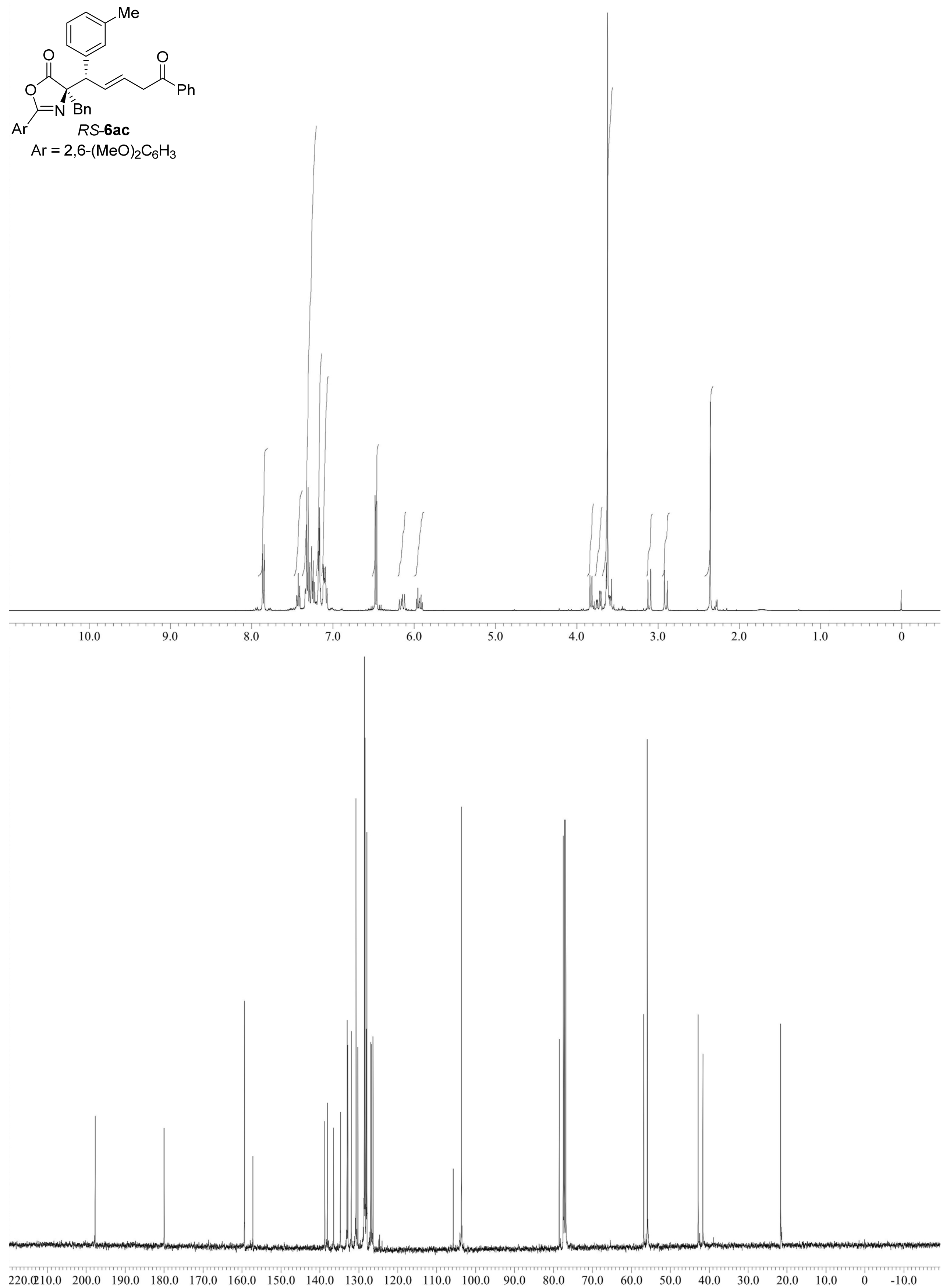

Supplementary Figure 62. ^{1}H and ^{13}C NMR spectra of *RS*-**6ac**

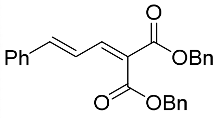

7

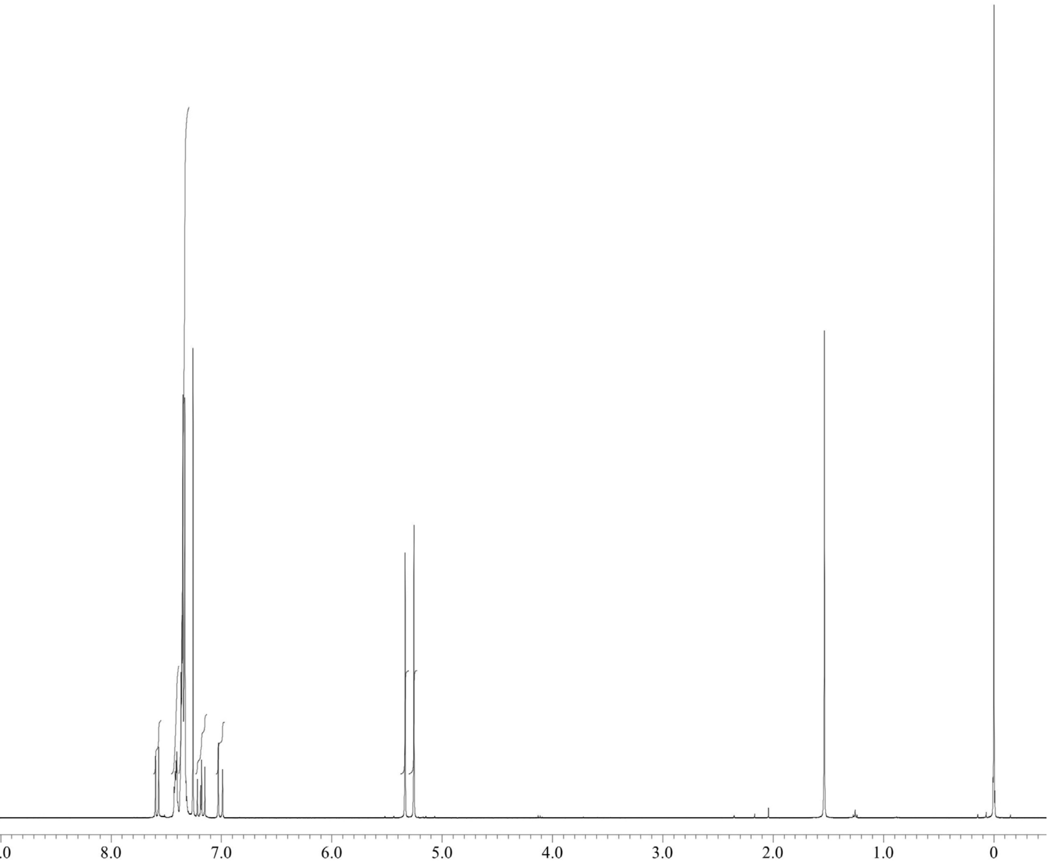

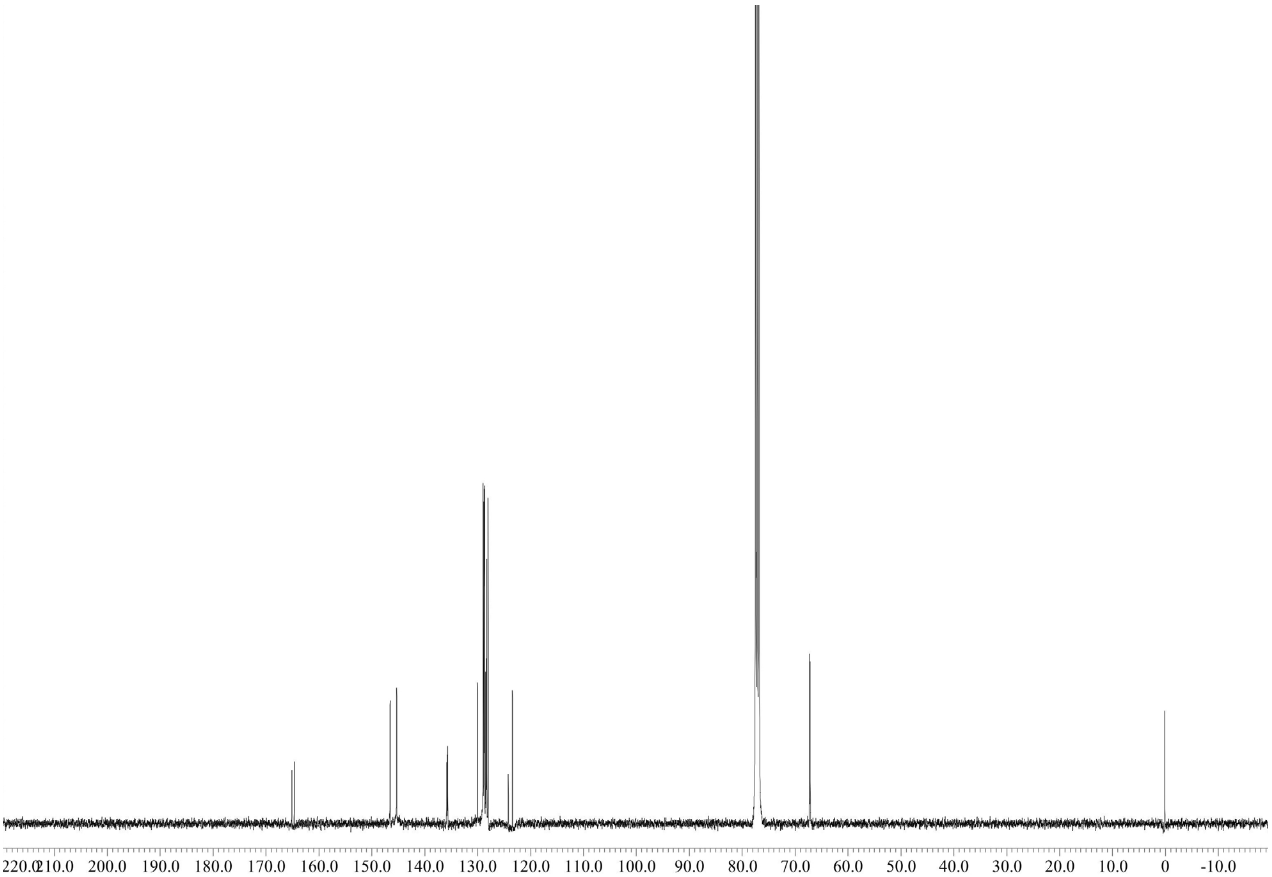

Supplementary Figure 63. ^{1}H and ^{13}C NMR spectra of **7**

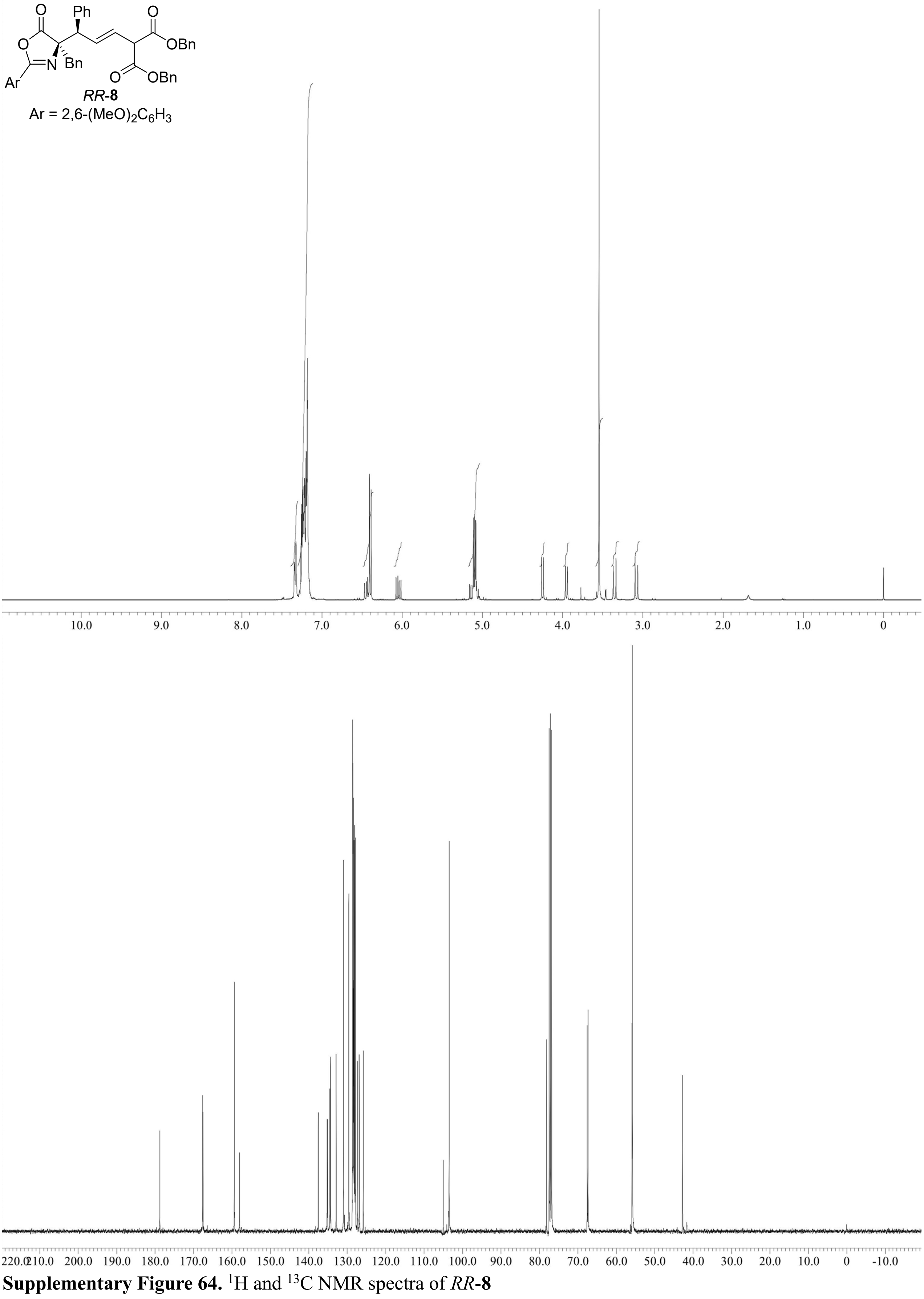

Supplementary Figure 64. ^{1}H and ^{13}C NMR spectra of *RR*-**8**

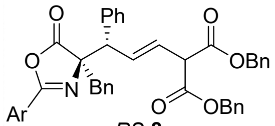

RS-**8**

Ar = 2,6-$(MeO)_2C_6H_3$

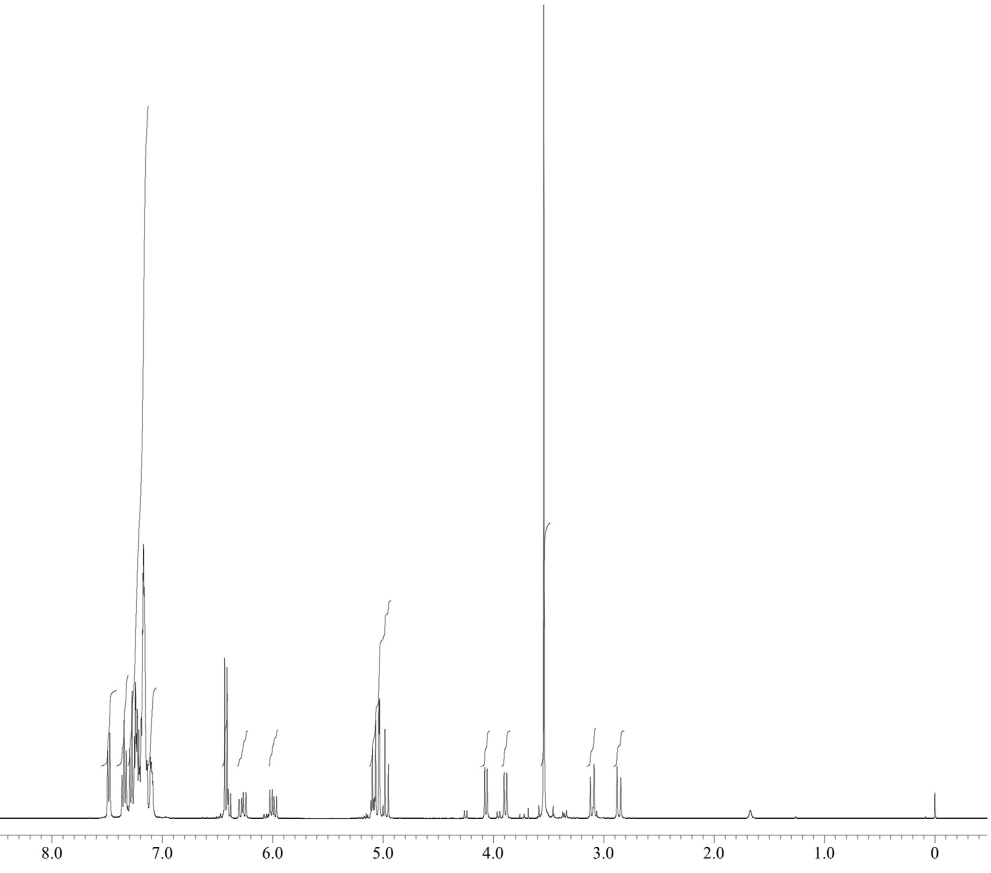

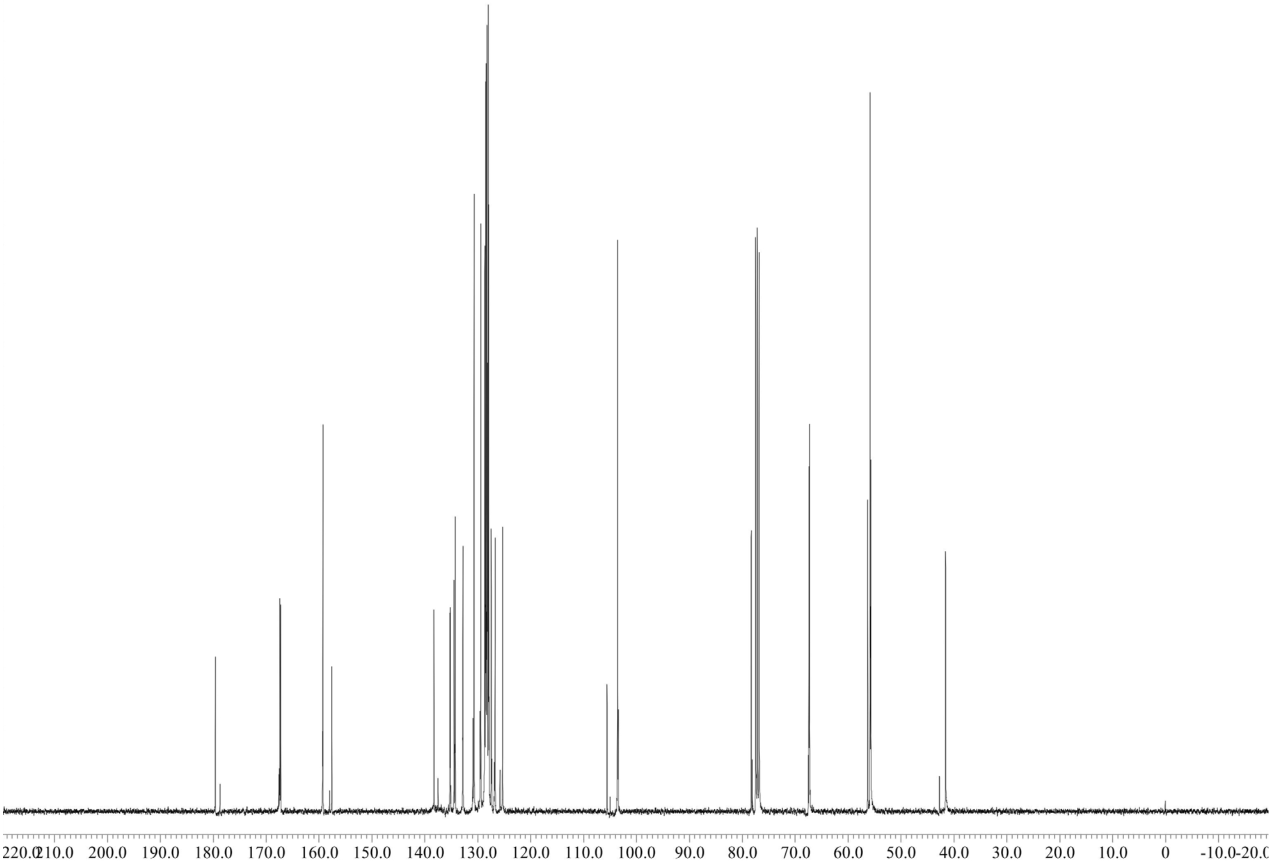

Supplementary Figure 65. ^{1}H and ^{13}C NMR spectra of *RS*-**8**

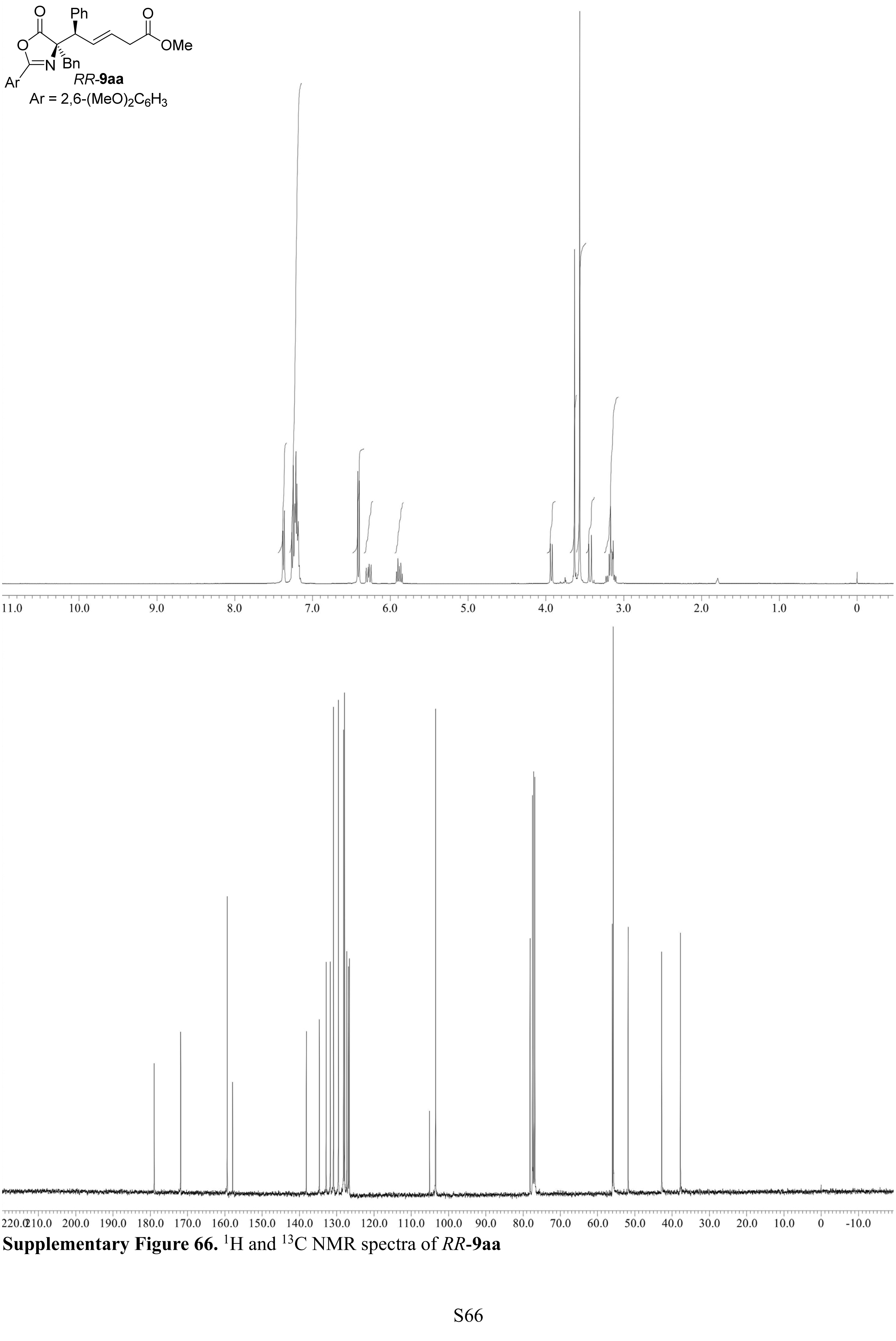

Supplementary Figure 66. ^{1}H and ^{13}C NMR spectra of *RR*-**9aa**

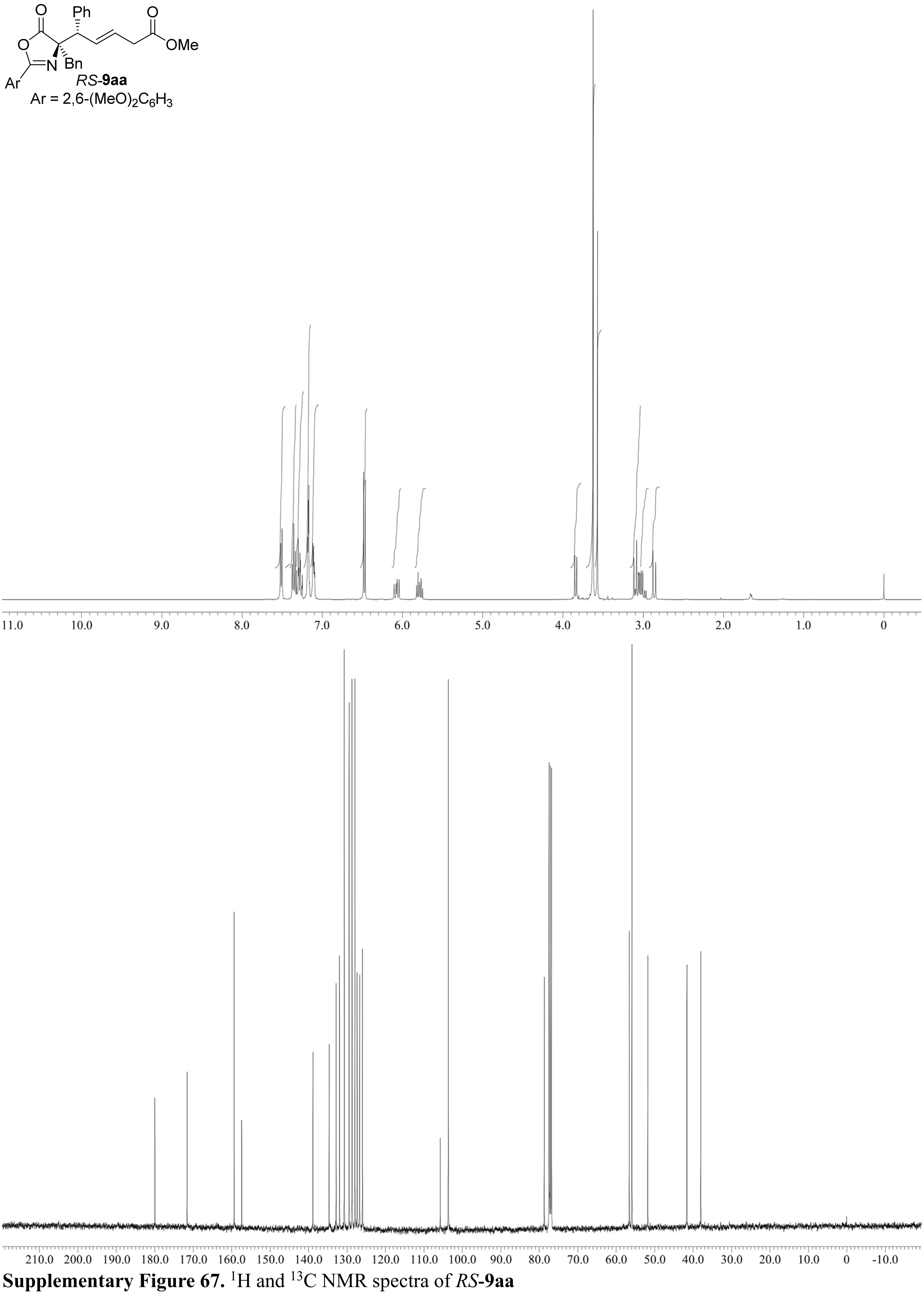

Supplementary Figure 67. ^{1}H and ^{13}C NMR spectra of *RS*-**9aa**

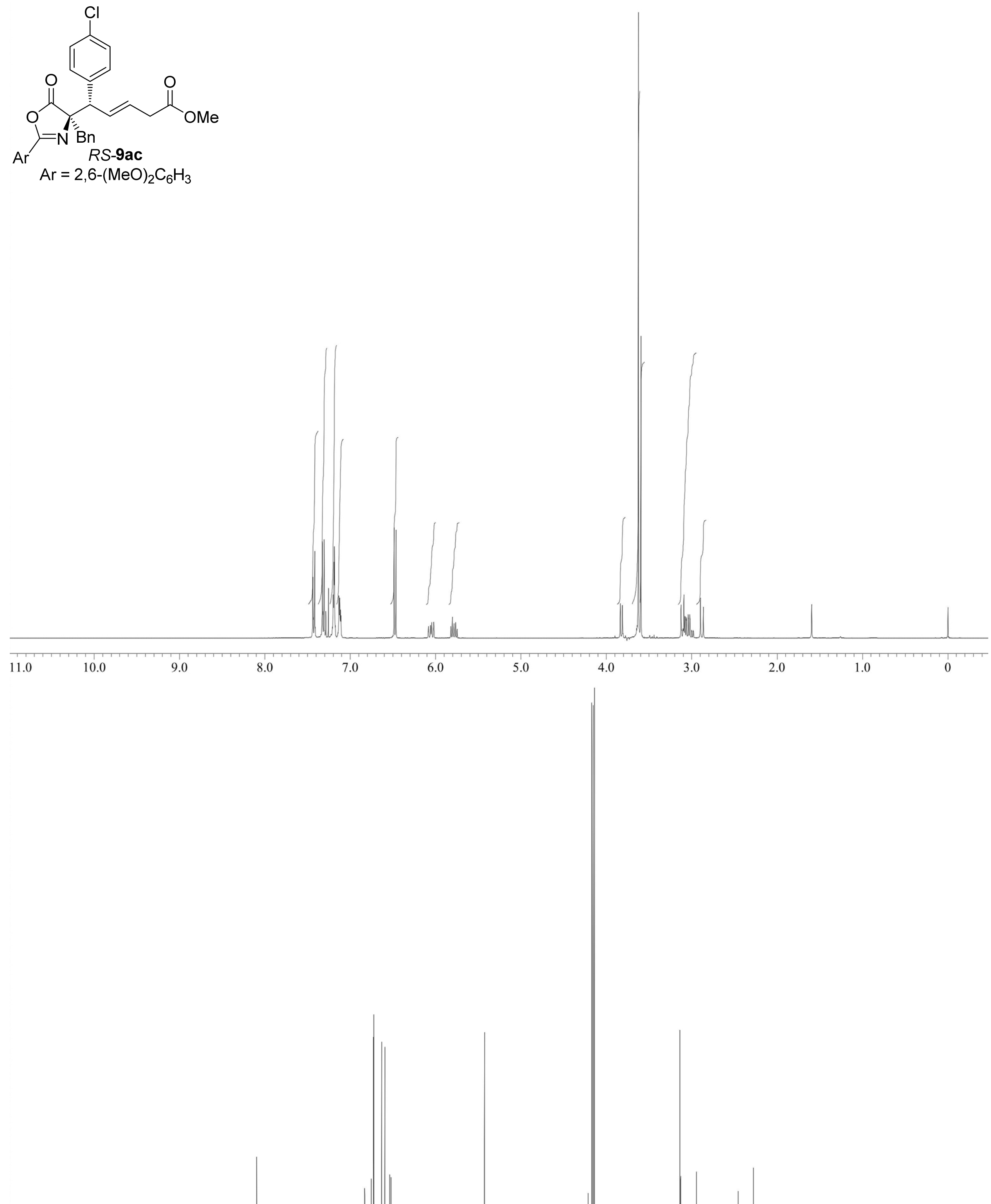

Supplementary Figure 68. ^{1}H and ^{13}C NMR spectra of *RS*-**9ac**

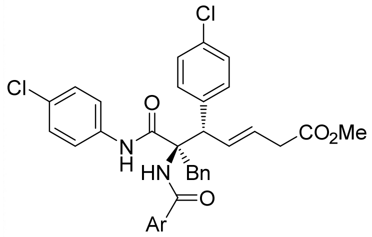

10
Ar = 2,6-$(MeO)_2C_6H_3$

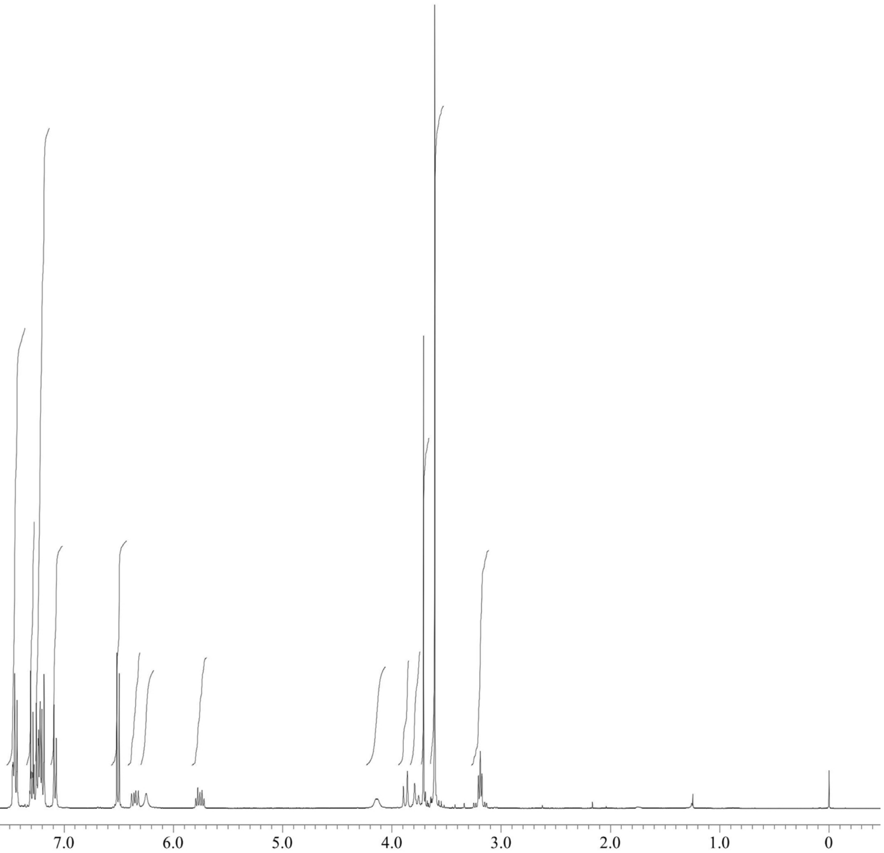

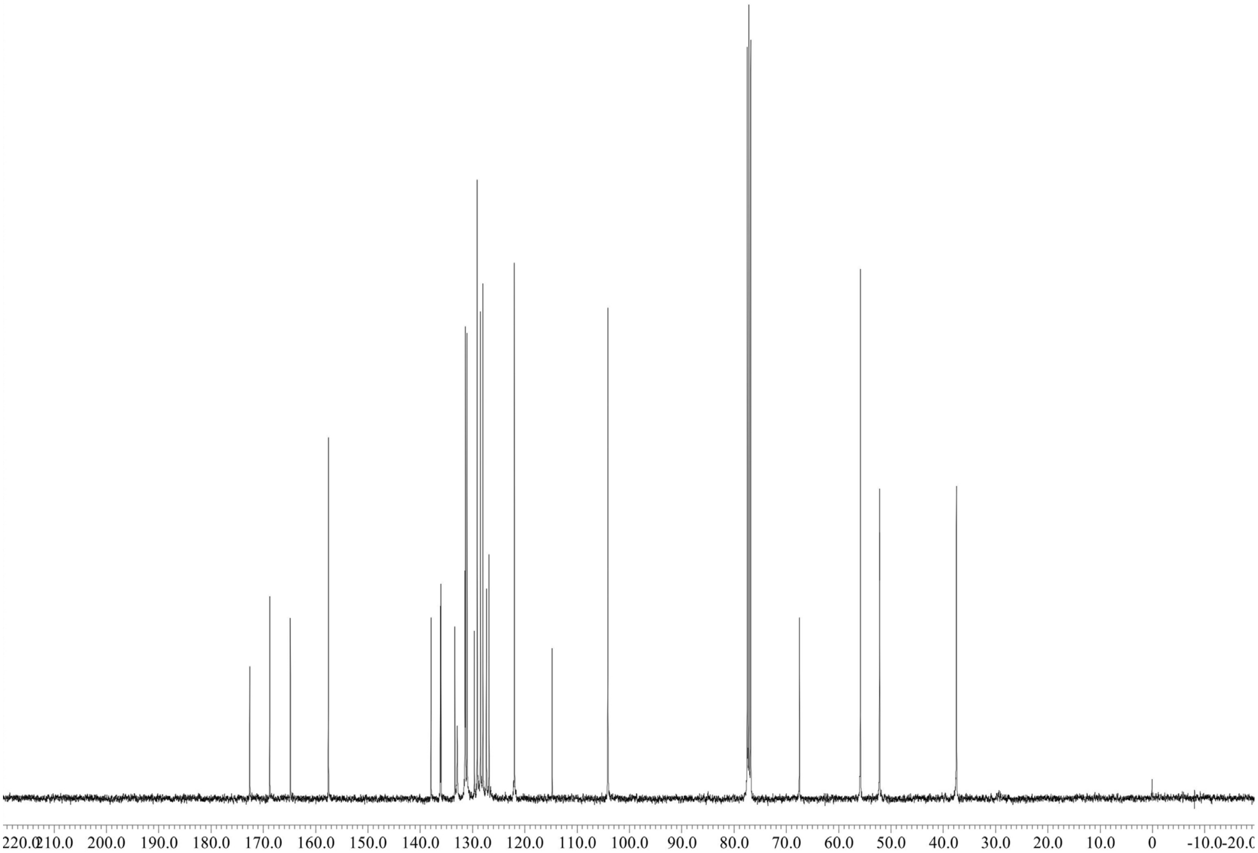

Supplementary Figure 69. ^{1}H and ^{13}C NMR spectra of **10**

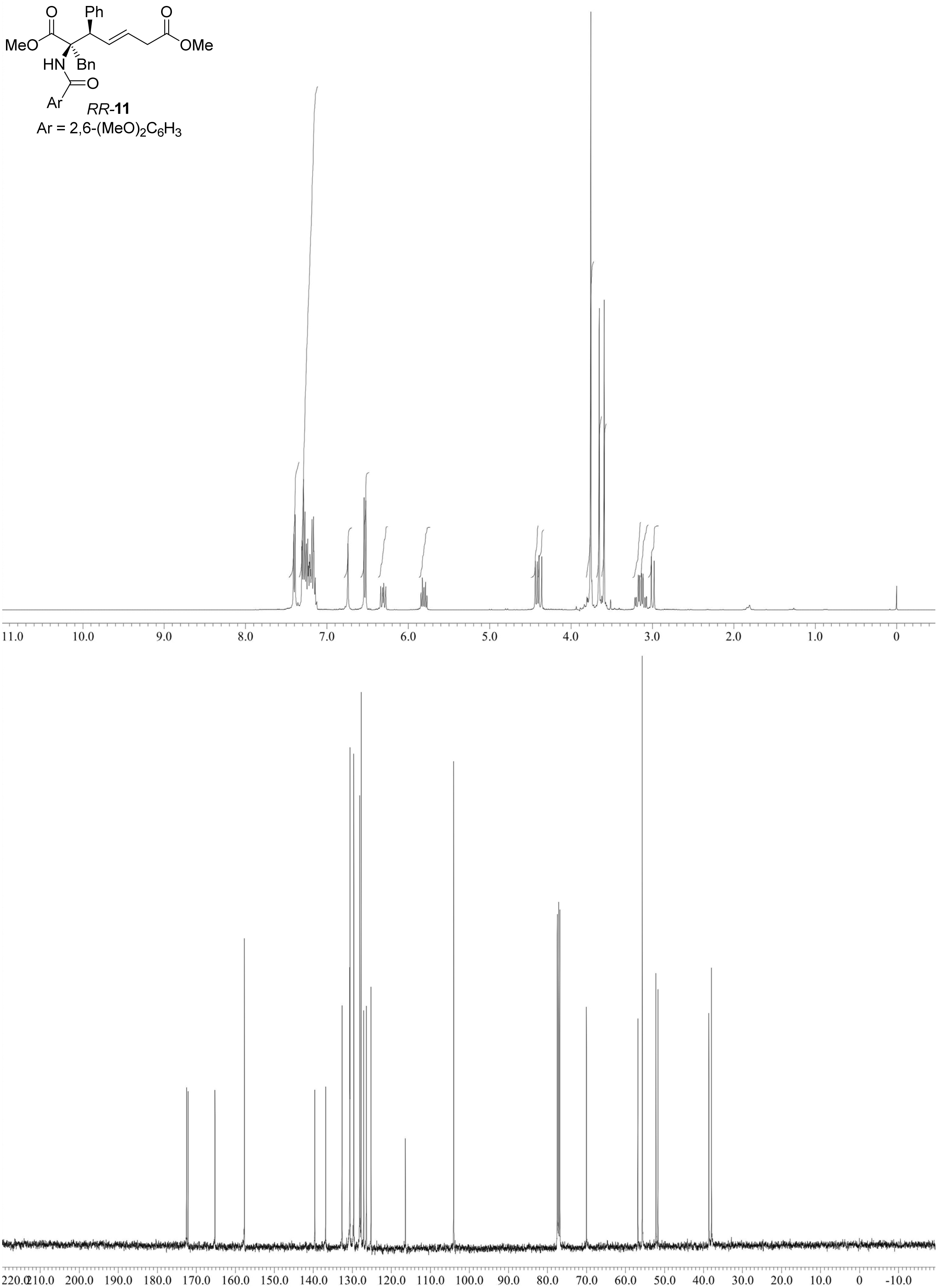

Supplementary Figure 70. ^{1}H and ^{13}C NMR spectra of *RR*-**11**

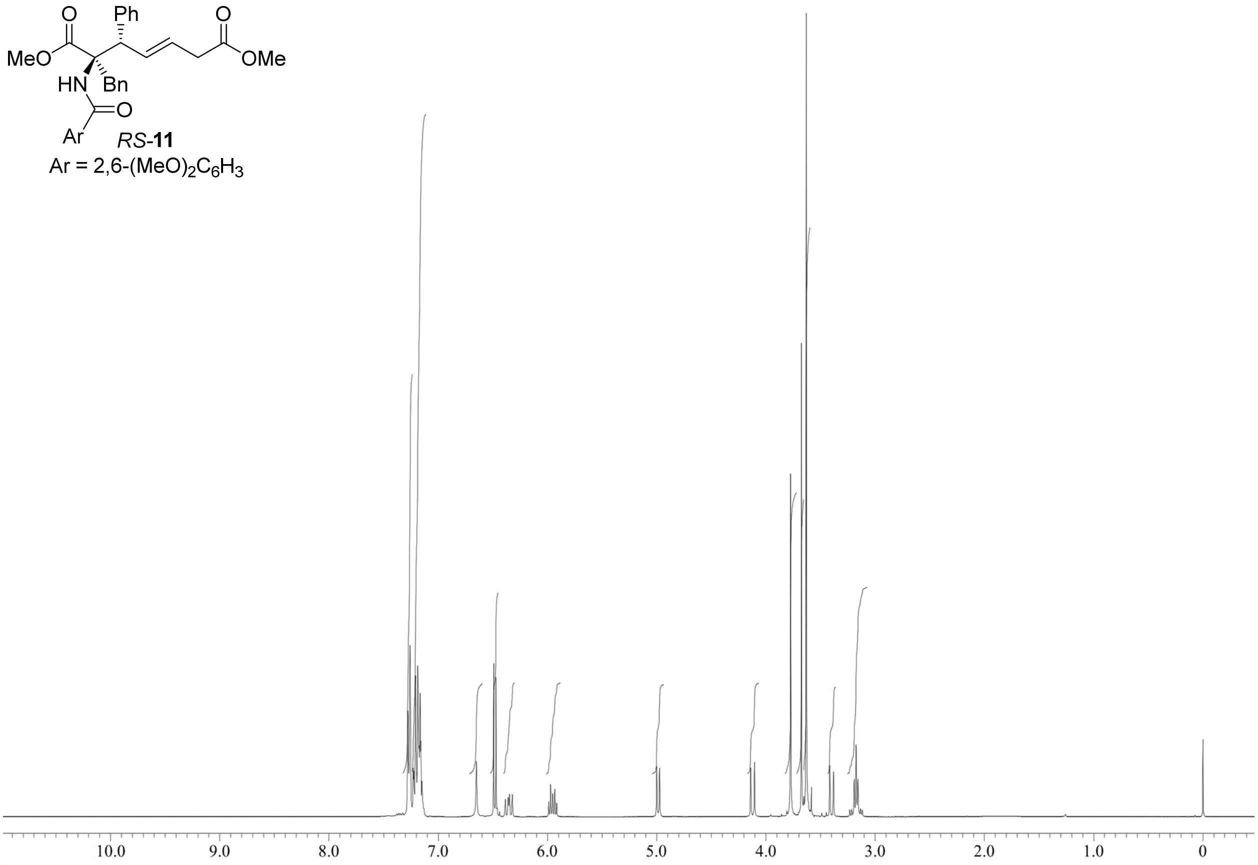

RS-**11**

Ar = 2,6-$(MeO)_2C_6H_3$

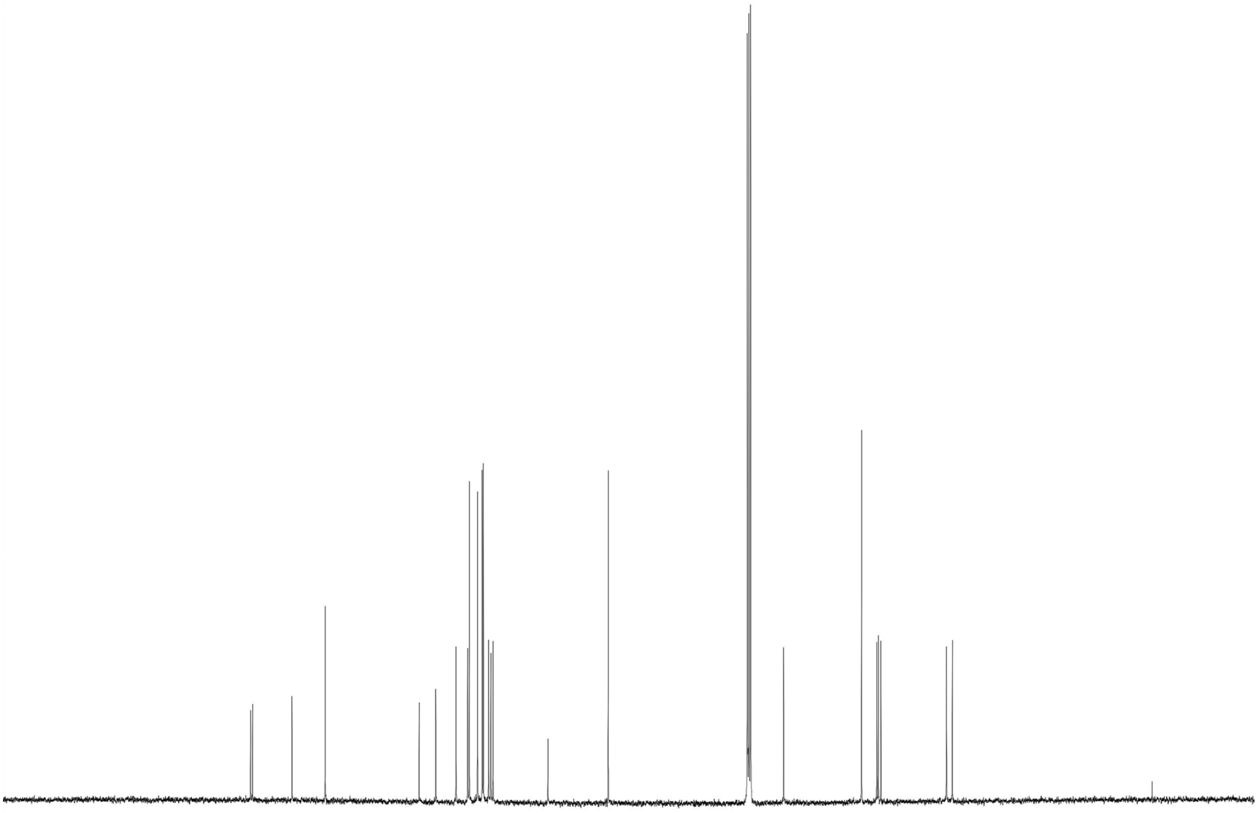

Supplementary Figure 71. ^{1}H and ^{13}C NMR spectra of *RS*-**11**

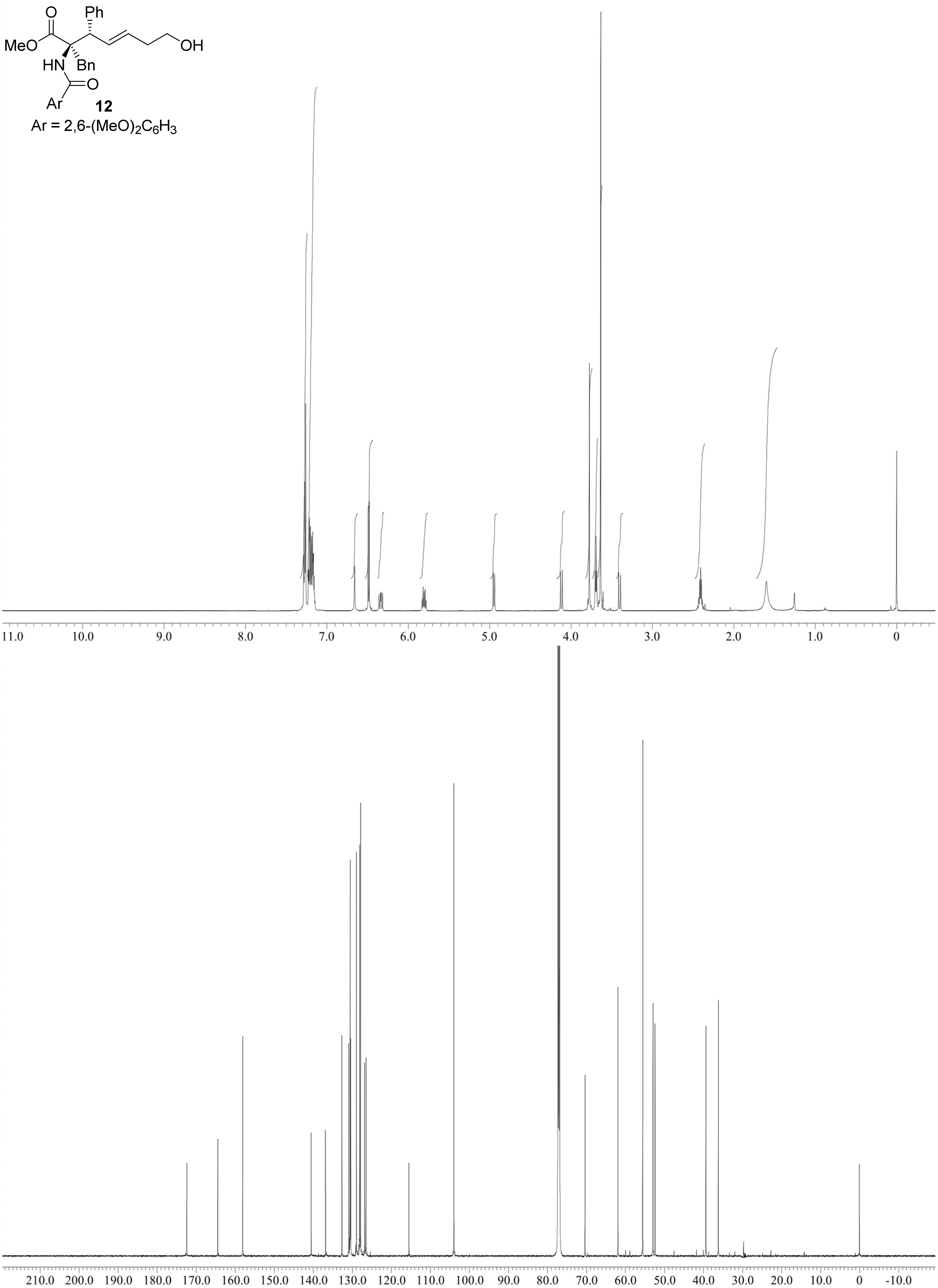

Supplementary Figure 72. ^{1}H and ^{13}C NMR spectra of **12**

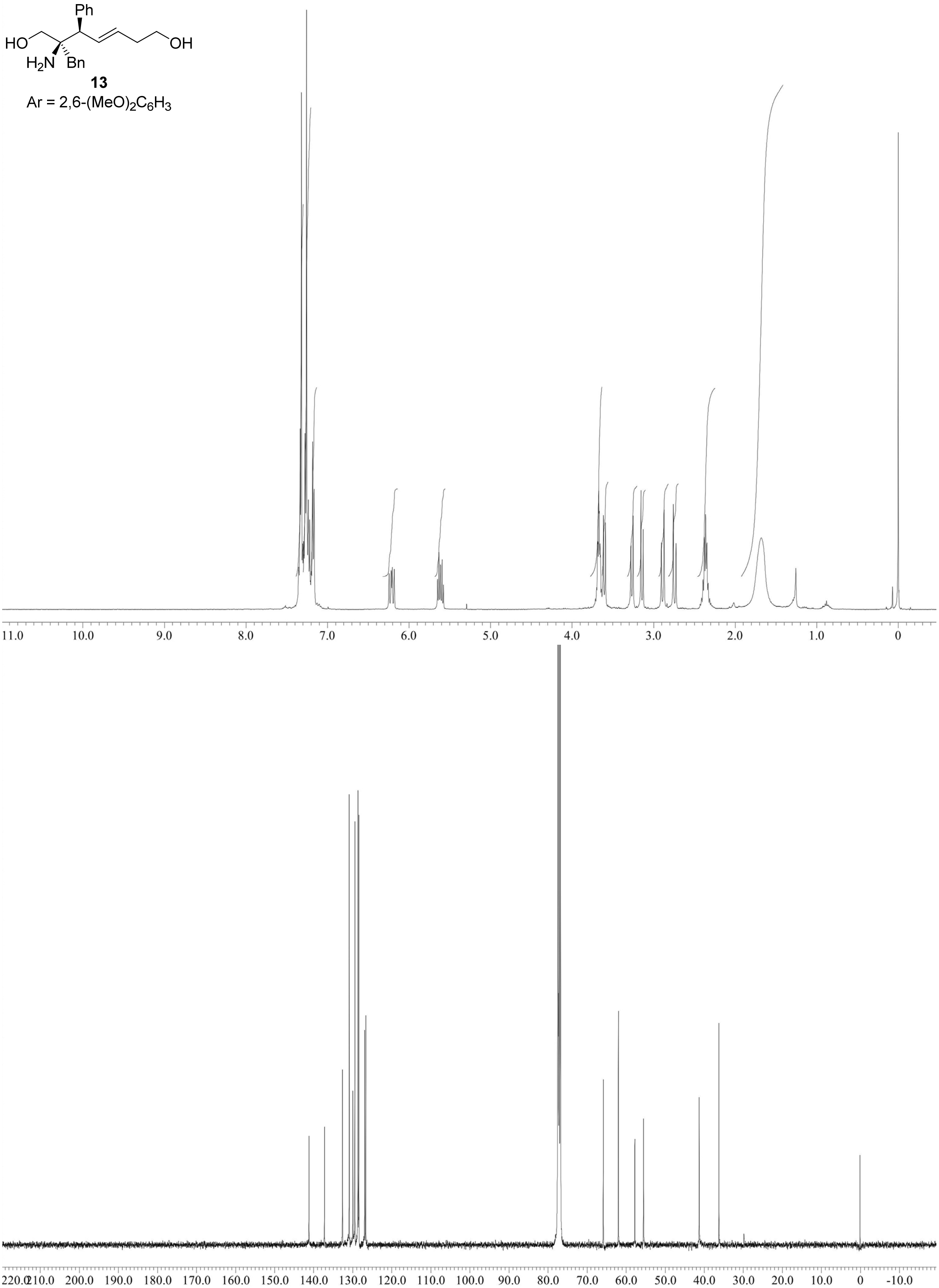

Supplementary Figure 73. ^{1}H and ^{13}C NMR spectra of **13**

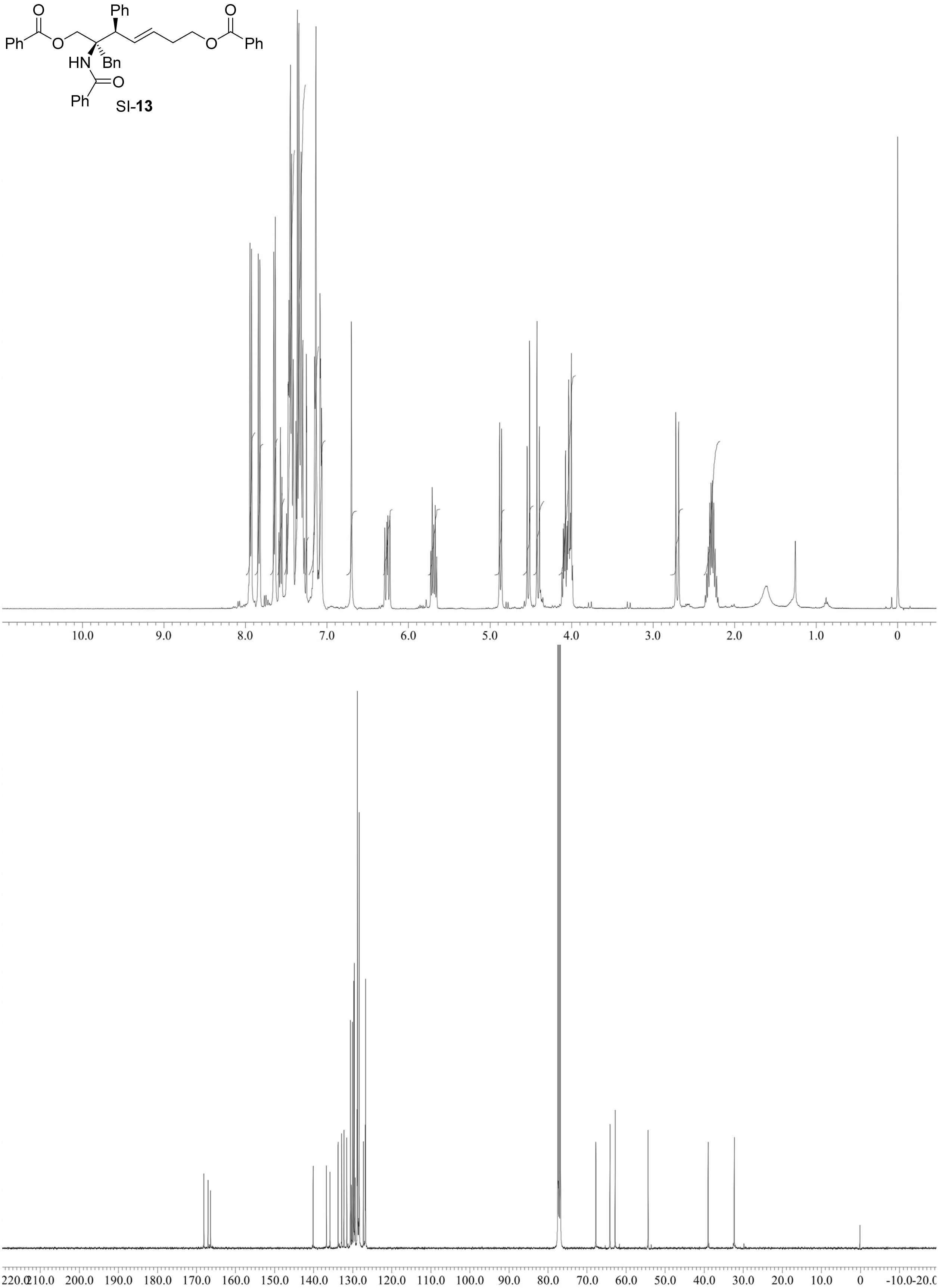

Supplementary Figure 74. ^{1}H and ^{13}C NMR spectra of SI-**13**

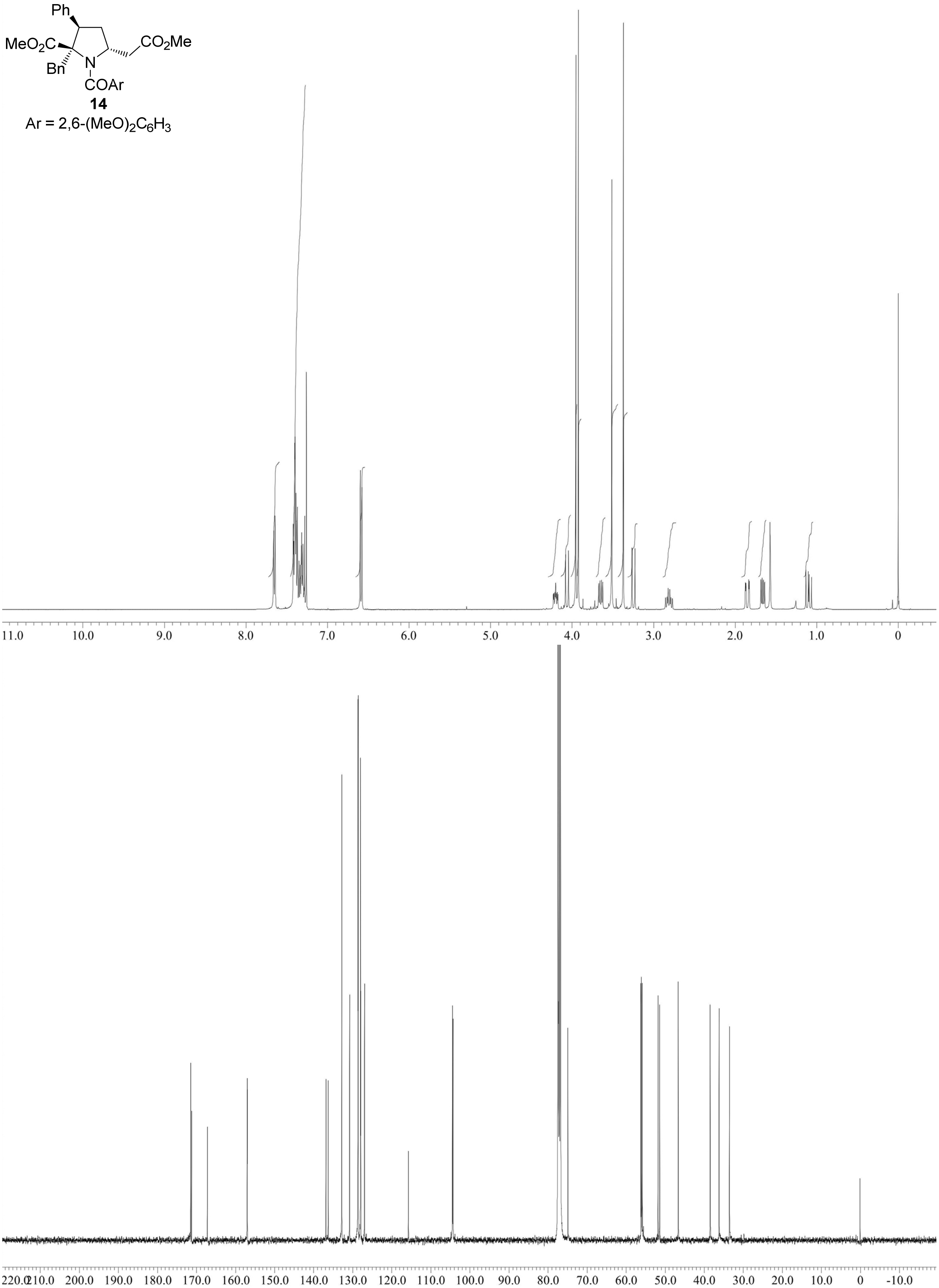

Supplementary Figure 75. ^{1}H and ^{13}C NMR spectra of **14**

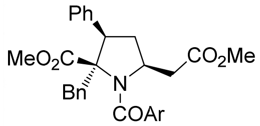

15

Ar = 2,6-$(MeO)_2C_6H_3$

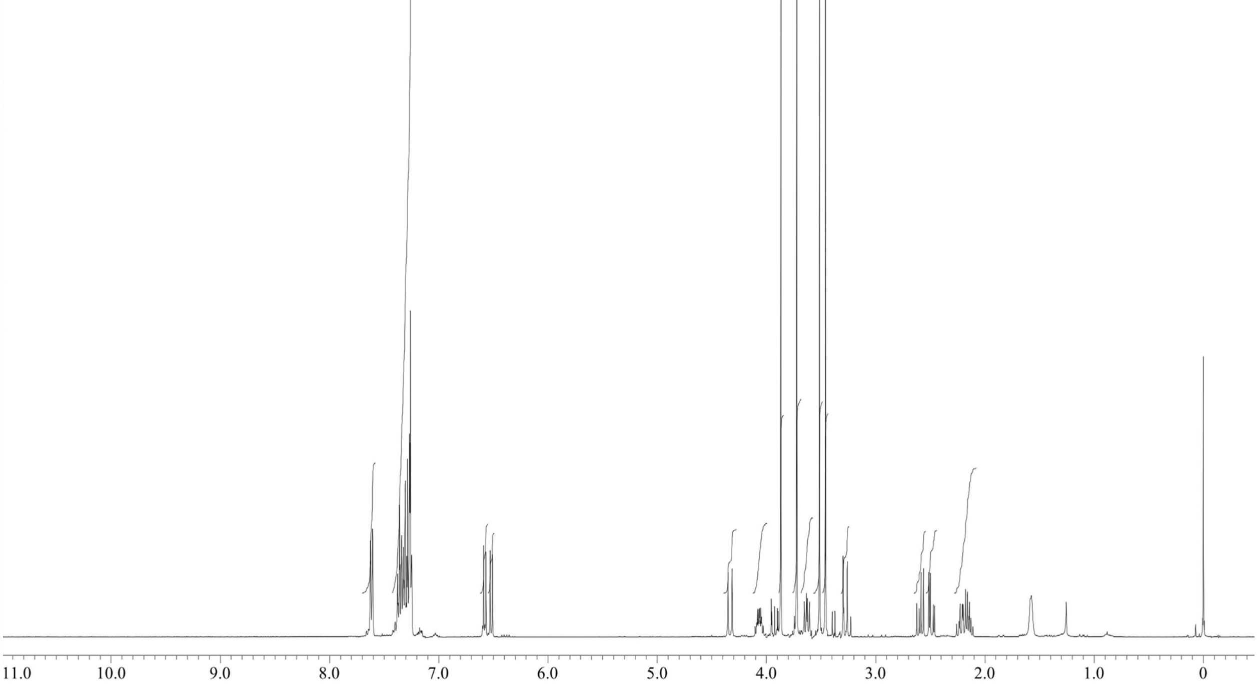

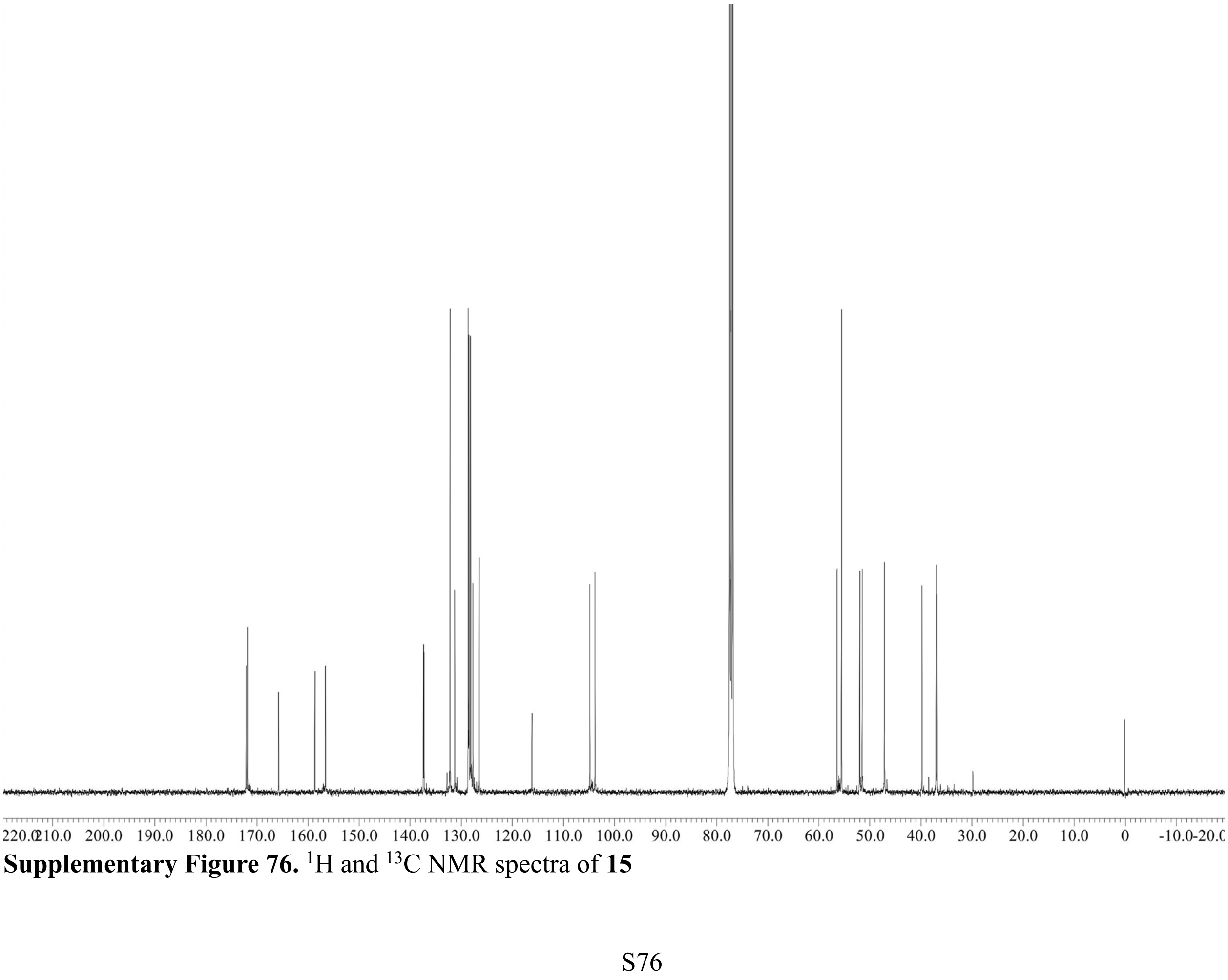

Supplementary Figure 76. ^{1}H and ^{13}C NMR spectra of **15**

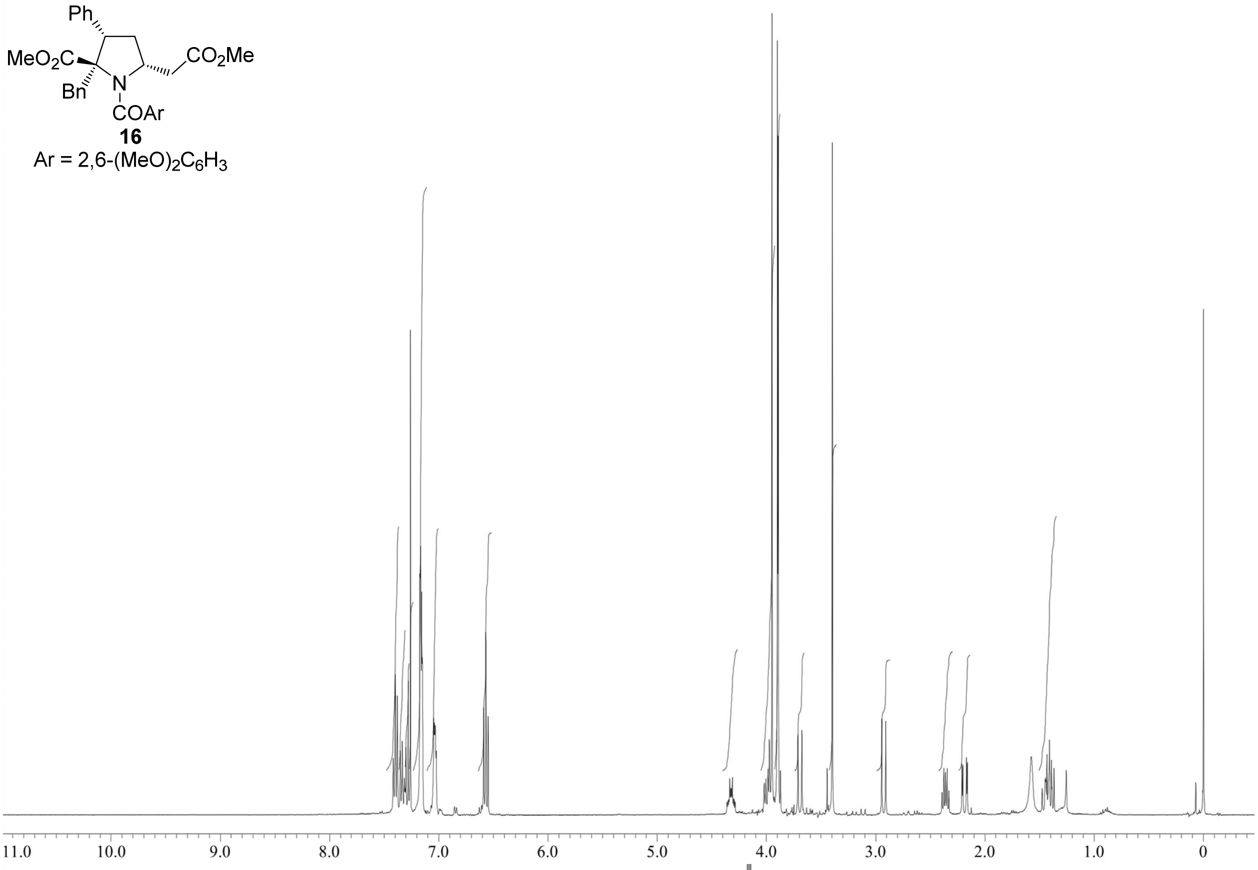

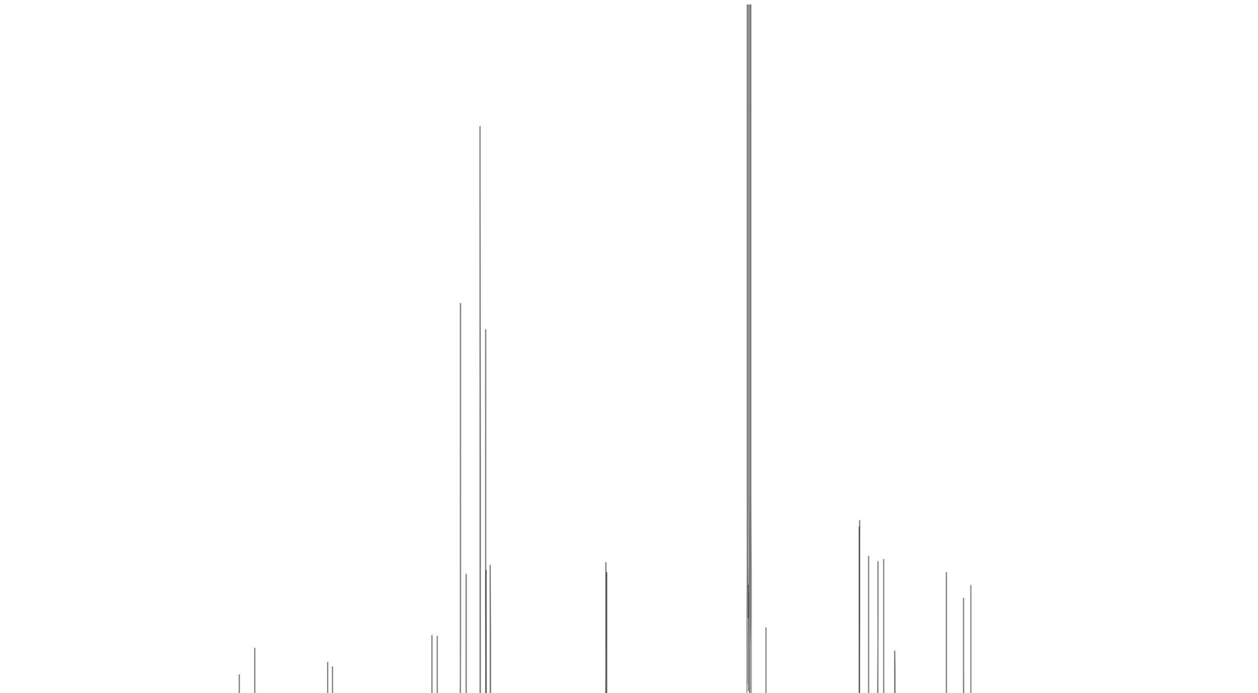

Supplementary Figure 77. ^{1}H and ^{13}C NMR spectra of **16**

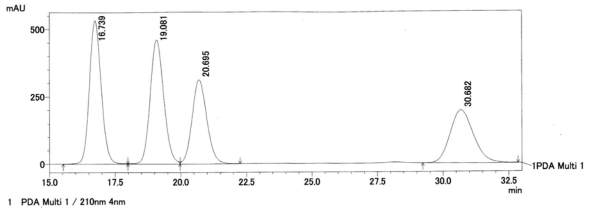

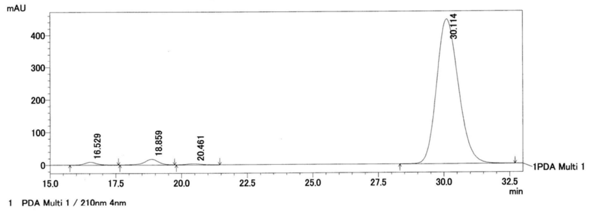

PDA Ch1 210nm 4nm

ピーク#	保持時間	面積%
1	16.529	0.969
2	18.859	2.401
3	20.461	0.436
4	30.114	96.194
合計		100.000

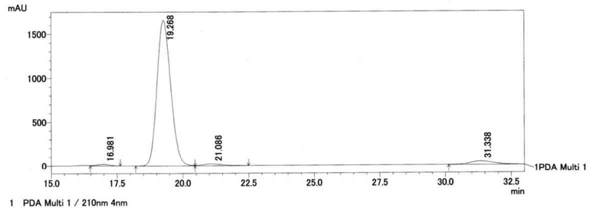

PDA Ch1 210nm 4nm

ピーク#	保持時間	面積%
1	16.981	0.898
2	19.268	94.066
3	21.086	1.469
4	31.338	3.567
合計		100.000

Supplementary Figure 78. HPLC trace of **4aa**

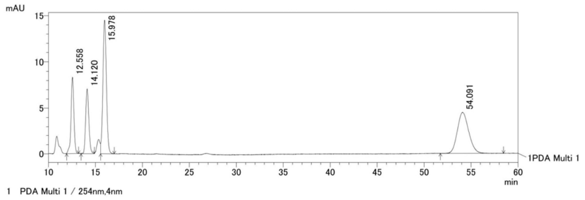

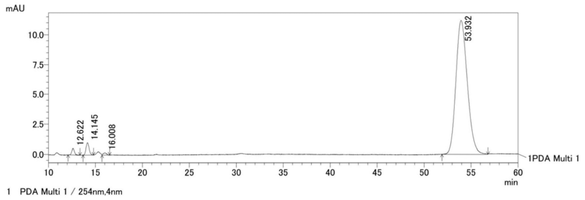

PDA Ch1 254nm

ピーク#	保持時間	面積%
1	12.622	1.067
2	14.145	2.366
3	16.008	0.444
4	53.932	96.124
合計		100.000

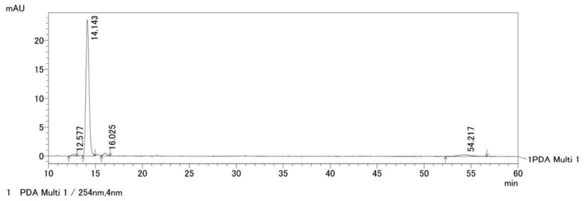

PDA Ch1 254nm

ピーク#	保持時間	面積%
1	12.577	0.870
2	14.143	92.634
3	16.025	2.034
4	54.217	4.462
合計		100.000

Supplementary Figure 79. HPLC trace of **4ab**

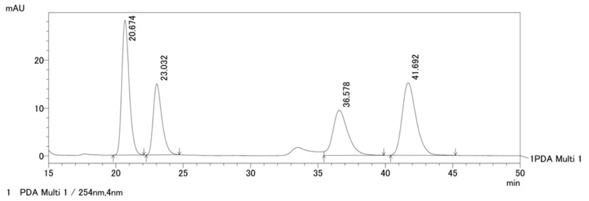

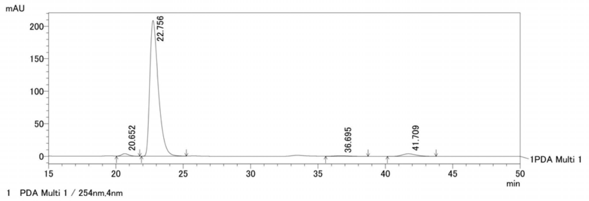

PDA Ch1 254nm

ピーク#	保持時間	面積%
1	20.652	1.552
2	22.756	94.705
3	36.695	0.809
4	41.709	2.934
合計		100.000

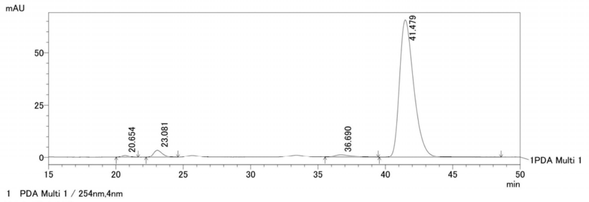

PDA Ch1 254nm

ピーク#	保持時間	面積%
1	20.654	0.713
2	23.081	3.113
3	36.690	2.193
4	41.479	93.981
合計		100.000

Supplementary Figure 80. HPLC trace of **4ac**

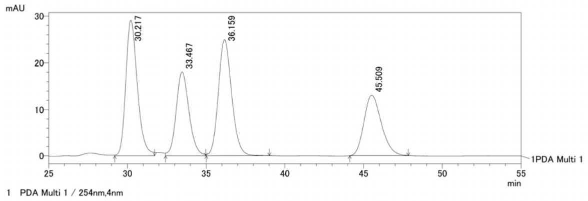

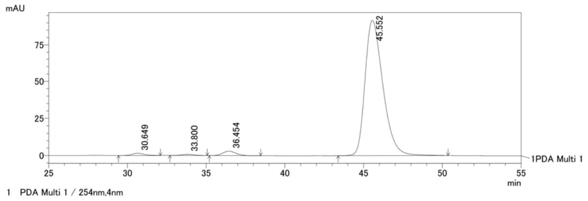

PDA Ch1 254nm

ピーク#	保持時間	面積%
1	30.649	1.136
2	33.800	0.601
3	36.454	2.368
4	45.552	95.895
合計		100.000

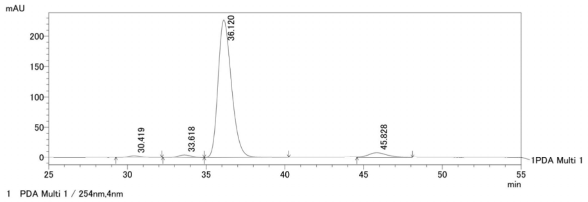

PDA Ch1 254nm

ピーク#	保持時間	面積%
1	30.419	0.966
2	33.618	1.709
3	36.120	92.297
4	45.828	5.027
合計		100.000

Supplementary Figure 81. HPLC trace of **4ad**

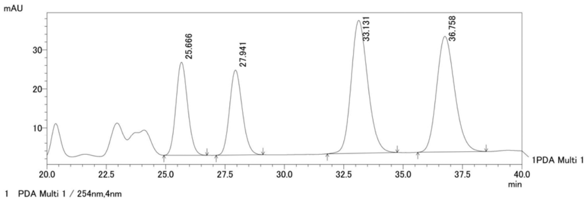

1 PDA Multi 1 / 254nm,4nm

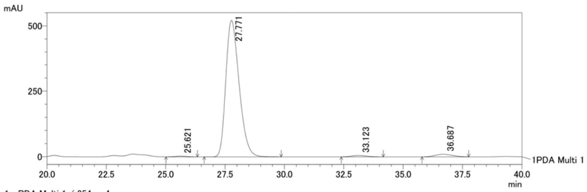

1 PDA Multi 1 / 254nm,4nm

PDA Ch1 254nm

ピーク#	保持時間	面積%
1	25.621	0.419
2	27.771	96.115
3	33.123	1.113
4	36.687	2.354
合計		100.000

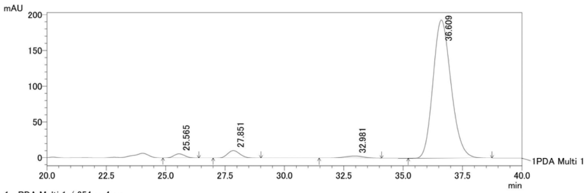

1 PDA Multi 1 / 254nm,4nm

PDA Ch1 254nm

ピーク#	保持時間	面積%
1	25.565	1.846
2	27.851	3.606
3	32.981	1.745
4	36.609	92.803
合計		100.000

Supplementary Figure 82. HPLC trace of **4ae**

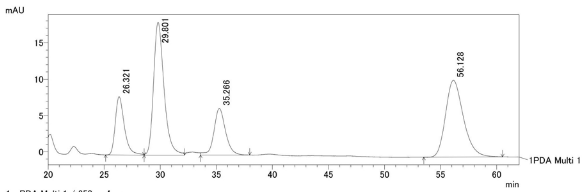

1 PDA Multi 1 / 250nm,4nm

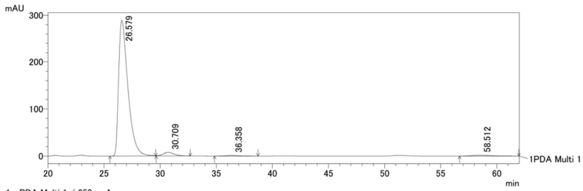

1 PDA Multi 1 / 250nm,4nm

PDA Ch1 250nm

ピーク#	保持時間	面積%
1	26.579	95.245
2	30.709	2.920
3	36.358	0.571
4	58.512	1.265
合計		100.000

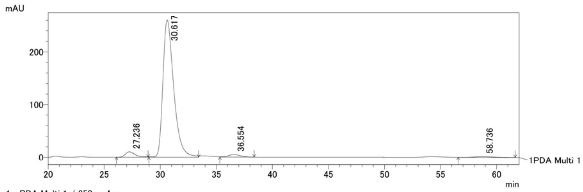

1 PDA Multi 1 / 250nm,4nm

PDA Ch1 250nm

ピーク#	保持時間	面積%
1	27.236	3.336
2	30.617	93.720
3	36.554	1.990
4	58.736	0.954
合計		100.000

Supplementary Figure 83. HPLC trace of **4af**

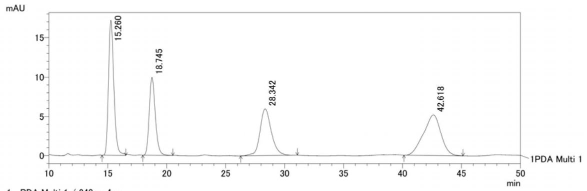

1 PDA Multi 1 / 240nm,4nm

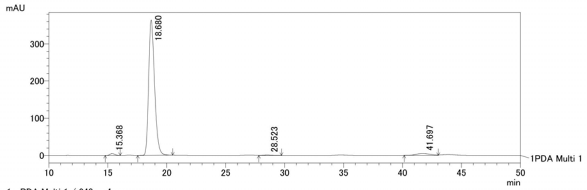

1 PDA Multi 1 / 240nm,4nm

PDA Ch1 240nm

ピーク#	保持時間	面積%
1	15.368	1.104
2	18.680	95.226
3	28.523	0.431
4	41.697	3.239
合計		100.000

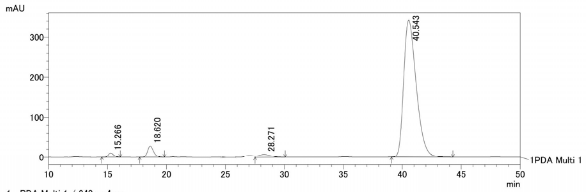

1 PDA Multi 1 / 240nm,4nm

PDA Ch1 240nm

ピーク#	保持時間	面積%
1	15.266	1.146
2	18.620	3.626
3	28.271	1.705
4	40.543	93.523
合計		100.000

Supplementary Figure 84. HPLC trace of **4ag**

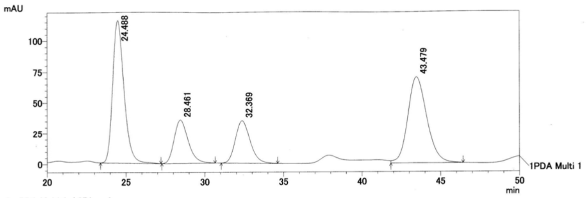

1 PDA Multi 1 / 254nm 4nm

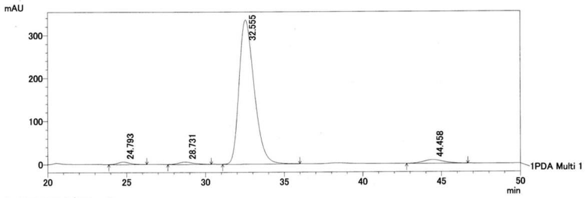

1 PDA Multi 1 / 254nm 4nm

PDA Ch1 254nm 4nm

ピーク#	保持時間	面積%
1	24.793	1.207
2	28.731	1.385
3	32.555	94.334
4	44.458	3.074
合計		100.000

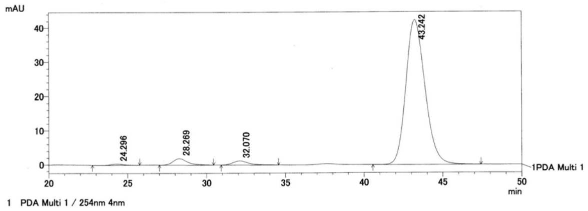

1 PDA Multi 1 / 254nm 4nm

PDA Ch1 254nm 4nm

ピーク#	保持時間	面積%
1	24.296	0.362
2	28.269	3.104
3	32.070	2.098
4	43.242	94.436
合計		100.000

Supplementary Figure 85. HPLC trace of **4ah**

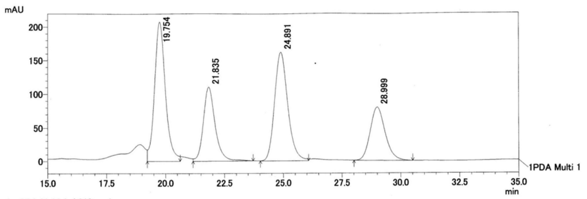

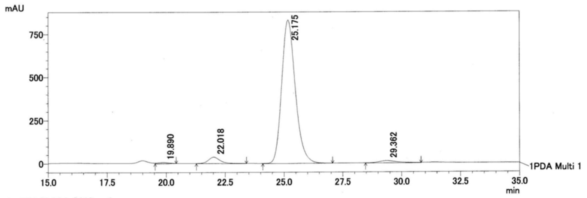

PDA Ch1 210nm 4nm

ピーク#	保持時間	面積%
1	19.890	0.494
2	22.018	3.479
3	25.175	94.252
4	29.362	1.775
合計		100.000

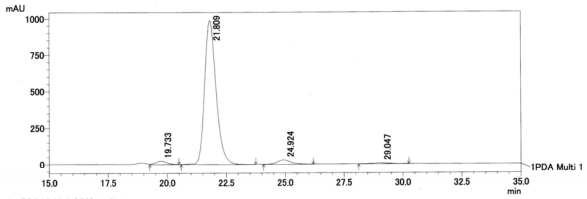

PDA Ch1 210nm 4nm

ピーク#	保持時間	面積%
1	19.733	2.148
2	21.809	93.747
3	24.924	3.263
4	29.047	0.842
合計		100.000

Supplementary Figure 86. HPLC trace of **4ai**

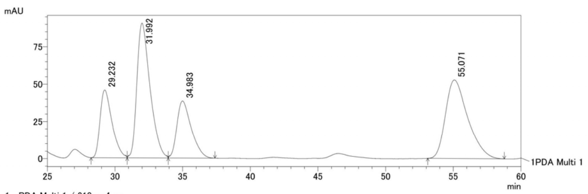

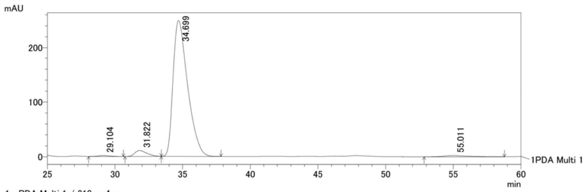

PDA Ch1 210nm

ピーク#	保持時間	面積%
1	29.104	0.565
2	31.822	3.568
3	34.699	94.237
4	55.011	1.631
合計		100.000

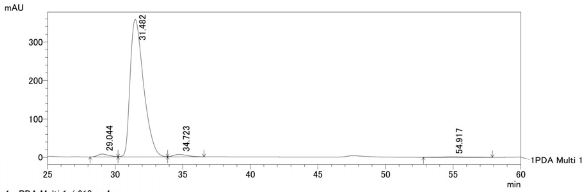

PDA Ch1 210nm

ピーク#	保持時間	面積%
1	29.044	1.684
2	31.482	95.934
3	34.723	1.798
4	54.917	0.584
合計		100.000

Supplementary Figure 87. HPLC trace of **4aj**

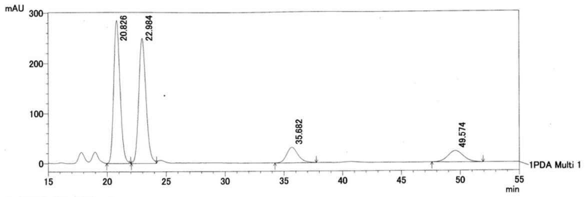

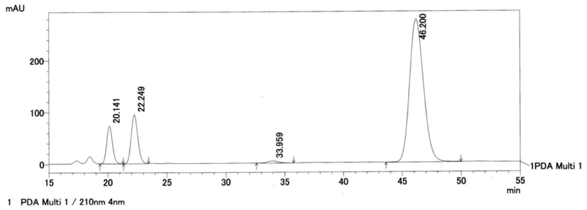

PDA Ch1 210nm 4nm

ピーク#	保持時間	面積%
1	20.141	8.534
2	22.249	12.684
3	33.959	0.785
4	46.200	77.997
合計		100.000

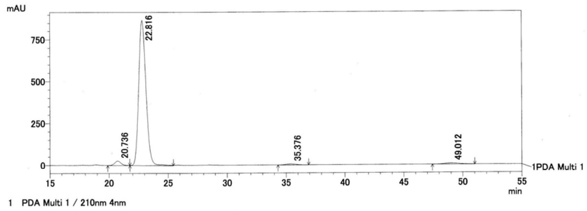

PDA Ch1 210nm 4nm

ピーク#	保持時間	面積%
1	20.736	2.432
2	22.816	95.359
3	35.376	0.840
4	49.012	1.369
合計		100.000

Supplementary Figure 88. HPLC trace of **4ak**

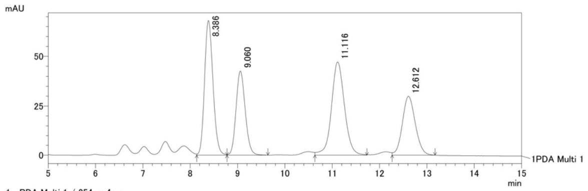

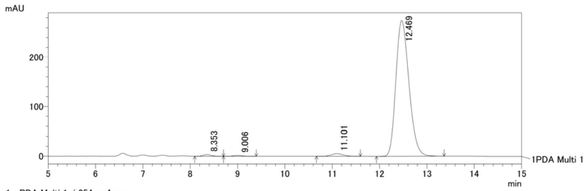

PDA Ch1 254nm

ピーク#	保持時間	面積%
1	8.353	0.802
2	9.006	0.468
3	11.101	1.806
4	12.469	96.923
合計		100.000

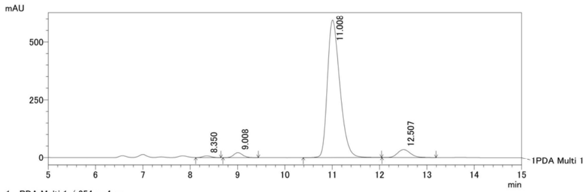

PDA Ch1 254nm

ピーク#	保持時間	面積%
1	8.350	0.749
2	9.008	2.437
3	11.008	91.254
4	12.507	5.561
合計		100.000

Supplementary Figure 89. HPLC trace of **4al**

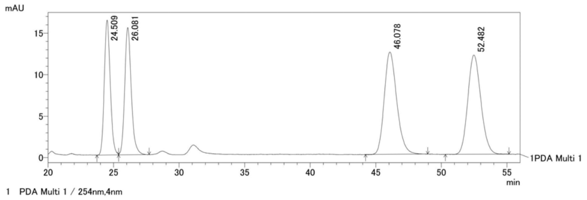

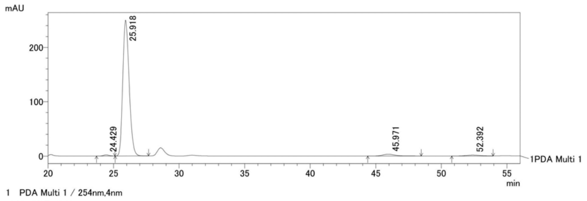

PDA Ch1 254nm

ピーク#	保持時間	面積%
1	24.429	0.744
2	25.918	95.241
3	45.971	2.520
4	52.392	1.494
合計		100.000

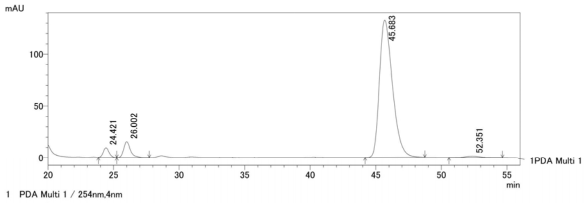

PDA Ch1 254nm

ピーク#	保持時間	面積%
1	24.421	2.837
2	26.002	5.378
3	45.683	90.862
4	52.351	0.923
合計		100.000

Supplementary Figure 90. HPLC trace of **4am**

4ba

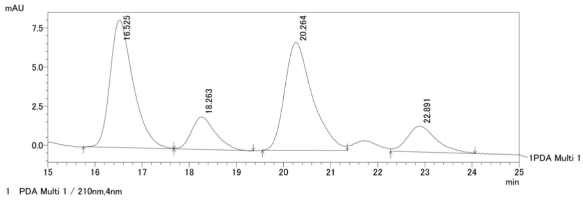

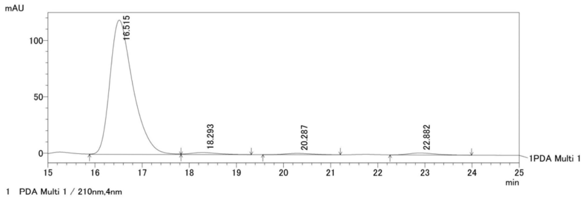

PDA Ch1 210nm

ピーク#	保持時間	面積%
1	16.515	95.663
2	18.293	1.591
3	20.287	1.162
4	22.882	1.585
合計		100.000

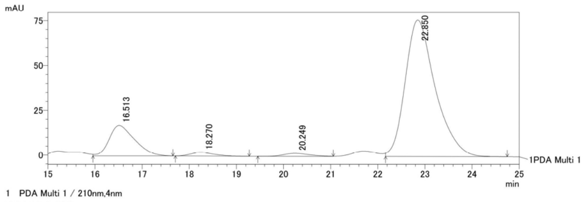

PDA Ch1 210nm

ピーク#	保持時間	面積%
1	16.513	15.438
2	18.270	1.746
3	20.249	1.598
4	22.850	81.218
合計		100.000

Supplementary Figure 91. HPLC trace of **4an**

4ca

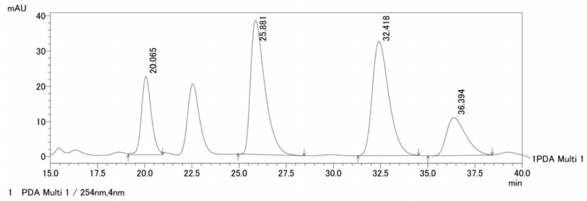

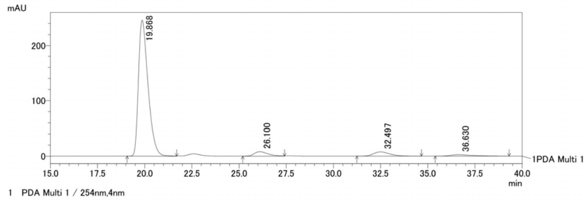

PDA Ch1 254nm

ピーク#	保持時間	面積%
1	19.868	88.893
2	26.100	4.272
3	32.497	4.708
4	36.630	2.127
合計		100.000

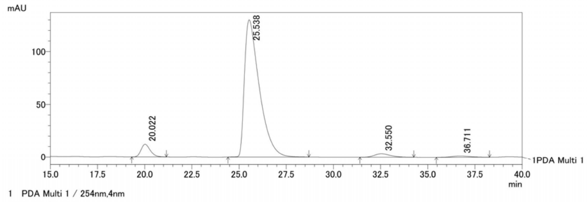

PDA Ch1 254nm

ピーク#	保持時間	面積%
1	20.022	5.226
2	25.538	91.245
3	32.550	2.509
4	36.711	1.020
合計		100.000

Supplementary Figure 92. HPLC trace of **4ba**

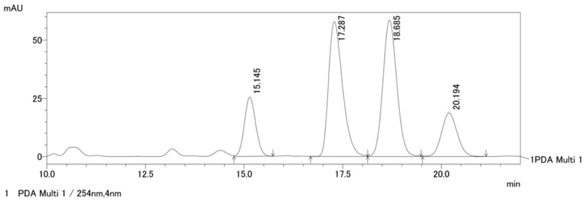

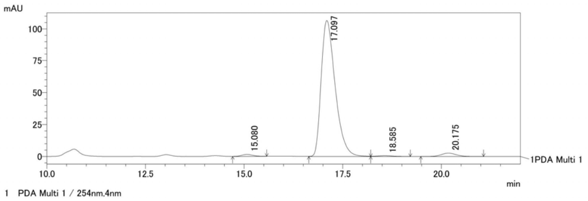

PDA Ch1 254nm

ピーク#	保持時間	面積%
1	15.080	1.174
2	17.097	95.613
3	18.585	0.665
4	20.175	2.548
合計		100.000

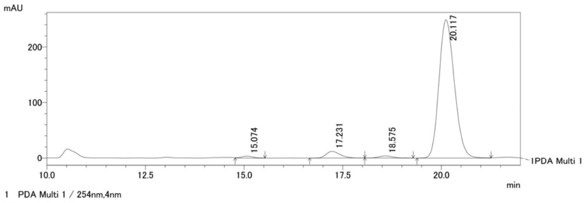

PDA Ch1 254nm

ピーク#	保持時間	面積%
1	15.074	0.929
2	17.231	4.031
3	18.575	1.237
4	20.117	93.803
合計		100.000

Supplementary Figure 93. HPLC trace of **4ca**

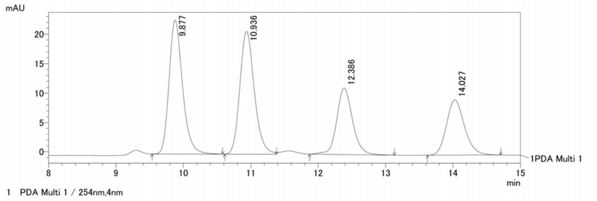

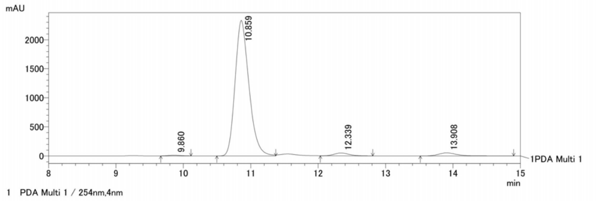

PDA Ch1 254nm

ピーク#	保持時間	面積%
1	9.860	0.405
2	10.859	94.632
3	12.339	2.111
4	13.908	2.853
合計		100.000

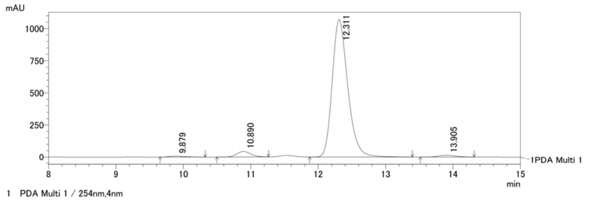

PDA Ch1 254nm

ピーク#	保持時間	面積%
1	9.879	0.662
2	10.890	3.499
3	12.311	94.546
4	13.905	1.293
合計		100.000

Supplementary Figure 94. HPLC trace of **4da**

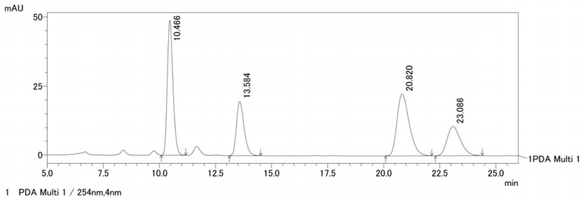

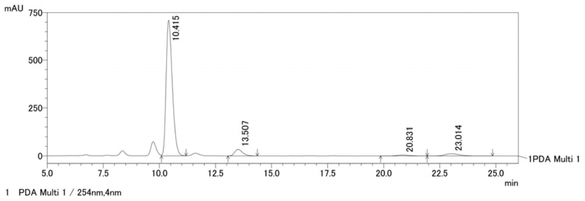

PDA Ch1 254nm

ピーク#	保持時間	面積%
1	10.415	89.855
2	13.507	5.586
3	20.831	1.189
4	23.014	3.369
合計		100.000

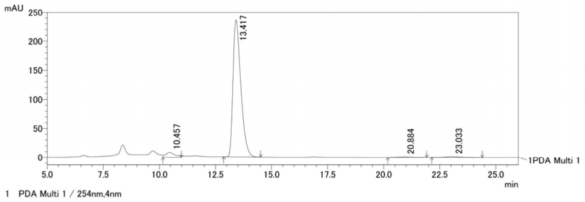

PDA Ch1 254nm

ピーク#	保持時間	面積%
1	10.457	4.196
2	13.417	94.440
3	20.884	0.479
4	23.033	0.885
合計		100.000

Supplementary Figure 95. HPLC trace of **4ea**

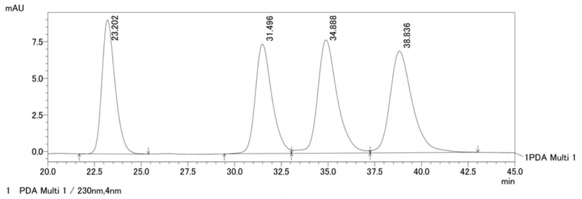

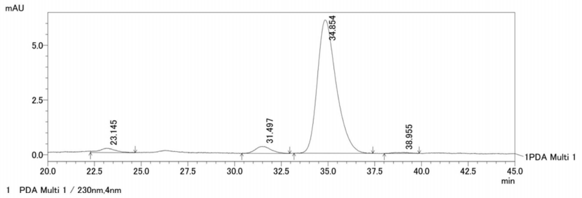

PDA Ch1 230nm

ピーク#	保持時間	面積%
1	23.145	2.568
2	31.497	4.043
3	34.854	92.862
4	38.955	0.527
合計		100.000

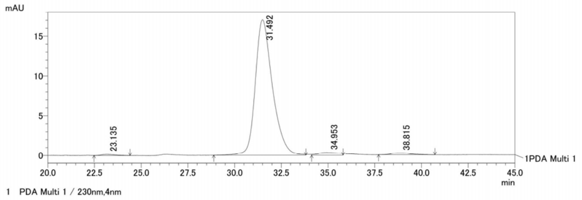

PDA Ch1 230nm

ピーク#	保持時間	面積%
1	23.135	0.667
2	31.492	95.855
3	34.953	2.093
4	38.815	1.385
合計		100.000

Supplementary Figure 96. HPLC trace of **6aa**

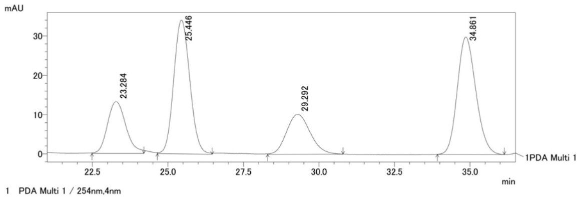

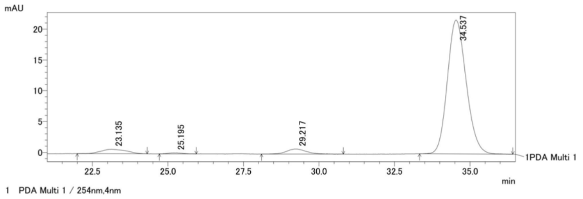

PDA Ch1 254nm

ピーク#	保持時間	面積%
1	23.135	4.091
2	25.195	0.495
3	29.217	3.627
4	34.537	91.787
合計		100.000

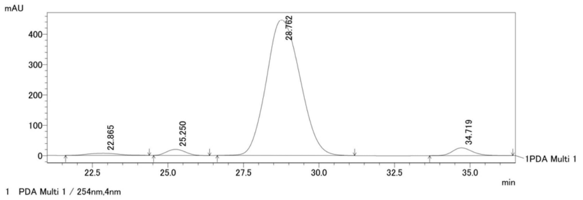

PDA Ch1 254nm

ピーク#	保持時間	面積%
1	22.865	1.403
2	25.250	2.300
3	28.762	93.167
4	34.719	3.129
合計		100.000

Supplementary Figure 97. HPLC trace of **6ab**

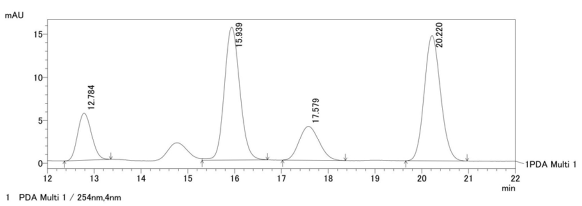

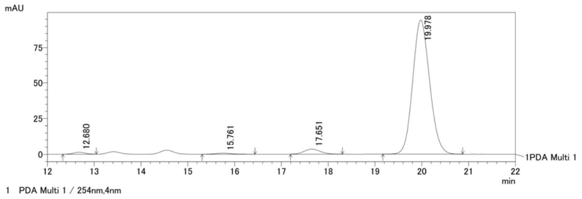

PDA Ch1 254nm

ピーク#	保持時間	面積%
1	12.680	0.939
2	15.761	0.710
3	17.651	3.470
4	19.978	94.881
合計		100.000

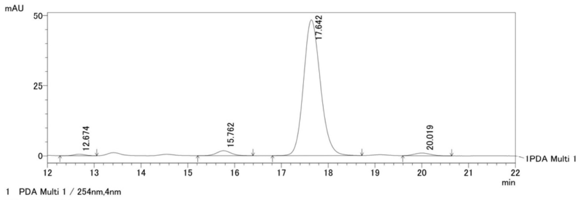

PDA Ch1 254nm

ピーク#	保持時間	面積%
1	12.674	0.706
2	15.762	3.016
3	17.642	94.485
4	20.019	1.792
合計		100.000

Supplementary Figure 98. HPLC trace of **6ac**

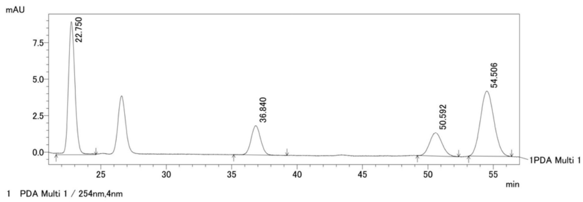

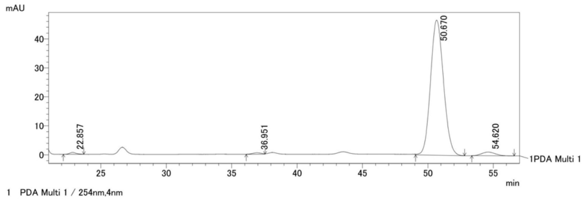

PDA Ch1 254nm

ピーク#	保持時間	面積%
1	22.857	0.689
2	36.951	0.684
3	50.670	95.926
4	54.620	2.701
合計		100.000

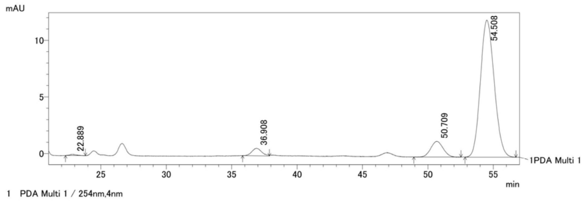

PDA Ch1 254nm

ピーク#	保持時間	面積%
1	22.889	0.377
2	36.908	3.416
3	50.709	8.830
4	54.508	87.377
合計		100.000

Supplementary Figure 99. HPLC trace of **8**

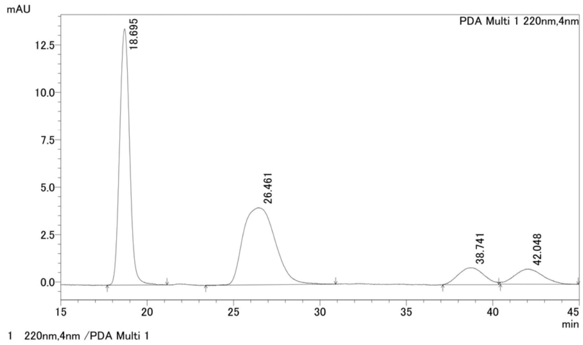

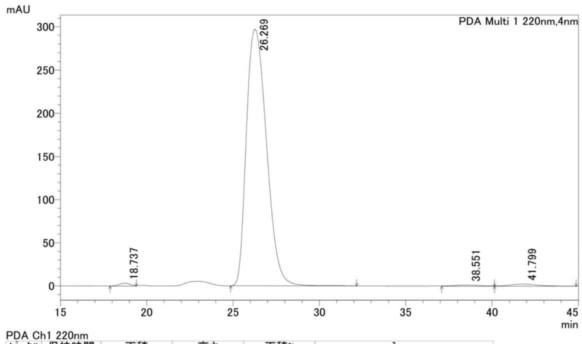

PDA Ch1 220nm

ピーク#	保持時間	面積	高さ	面積%	λ max
1	18.737	143683	3300	0.582	222
2	26.269	24141762	297007	97.829	204/221
3	38.551	129565	1219	0.525	221
4	41.799	262507	2428	1.064	204
合計		24677516	303954	100.000	

Supplementary Figure 100. HPLC trace of SI-**13**

Empirical formula	$C_{34}H_{32}N_2O_5S_1 \cdot C_1H_1Cl_3$	
Formula weight	700.04	
Temperature	93(2) K	
Wavelength	0.71073 Å	
Crystal system	Orthorhombic	
Space group	P2(1)2(1)2(1)	
Unit cell dimensions	a = 11.2529(5) Å	α = 90°.
	b = 14.0909(6) Å	β = 90°.
	c = 21.3929(11) Å	γ = 90°.
Volume	3392.1(3) $Å^3$	
Z	4	
Density (calculated)	1.371 Mg/m^3	
Absorption coefficient	0.376 mm^{-1}	
F(000)	1456	
Crystal size	0.30 x 0.30 x 0.30 mm^3	
Theta range for data collection	2.316 to 25.499°.	
Index ranges	–13<=h<=12, –16<=k<=17, –25<=l<=24	
Reflections collected	24918	
Independent reflections	6278 [R(int) = 0.0329]	
Completeness to theta = 25.242°	99.9 %	
Absorption correction	Semi-empirical from equivalents	
Max. and min. transmission	0.90 and 0.83	
Refinement method	Full-matrix least-squares on F^2	
Data / restraints / parameters	6278 / 0 / 418	
Goodness-of-fit on F^2	1.044	
Final R indices [I>2sigma(I)]	R_1 = 0.0235, wR_2 = 0.0592	
R indices (all data)	R_1 = 0.0247, wR_2 = 0.0601	
Absolute structure parameter	0.009(12)	
Extinction coefficient	0	
Largest diff. peak and hole	0.210 and –0.164 e.$Å^{-3}$	

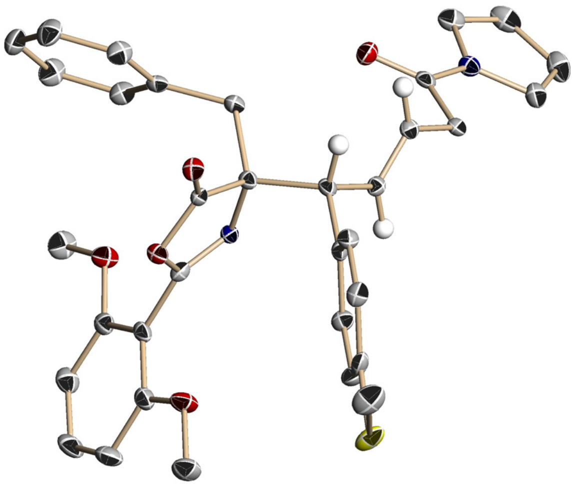

Supplementary Figure 101. Crystal data, structure refinement, and molecular structure of *RR*-**4ad** (CCDC 1494061). The thermal ellipsoids of non-hydrogen atoms are shown at the 50% probability level. Calculated hydrogen atoms except them attached to olefinic and stereogenic carbons are omitted for clarity. Blue = nitrogen, red = oxygen, gray = carbon, yellow = sulfur.

Empirical formula	$C_{36}H_{34}Cl_2N_2O_6$	
Formula weight	661.55	
Temperature	123.0(2) K	
Wavelength	0.71075 Å	
Crystal system	Orthorhombic	
Space group	P2(1)2(1)2(1)	
Unit cell dimensions	a = 8.2557(9) Å	α = 90°.
	b = 11.4883(15) Å	β = 90°.
	c = 34.369(4) Å	γ = 90°.
Volume	3259.7(7) $Å^3$	
Z	4	
Density (calculated)	1.348 Mg/m^3	
Absorption coefficient	0.249 mm^{-1}	
F(000)	1384	
Crystal size	0.010 x 0.010 x 0.020 mm^3	
Theta range for data collection	3.039 to 25.495°.	
Index ranges	–9<=h<=9, –13<=k<=13, –41<=l<=41	
Reflections collected	18848	
Independent reflections	5813 [R(int) = 0.0264]	
Completeness to theta = 25.242°	98.3 %	
Absorption correction	Semi-empirical from equivalents	
Max. and min. transmission	1.000 and 0.913	
Refinement method	Full-matrix least-squares on F^2	
Data / restraints / parameters	5813 / 0 / 426	
Goodness-of-fit on F^2	1.038	
Final R indices [I>2sigma(I)]	R_1 = 0.0281, wR_2 = 0.0605	
R indices (all data)	R_1 = 0.0374, wR_2 = 0.0646	
Absolute structure parameter	0.020(17)	
Extinction coefficient	0	
Largest diff. peak and hole	0.193 and –0.228 e.$Å^{-3}$	

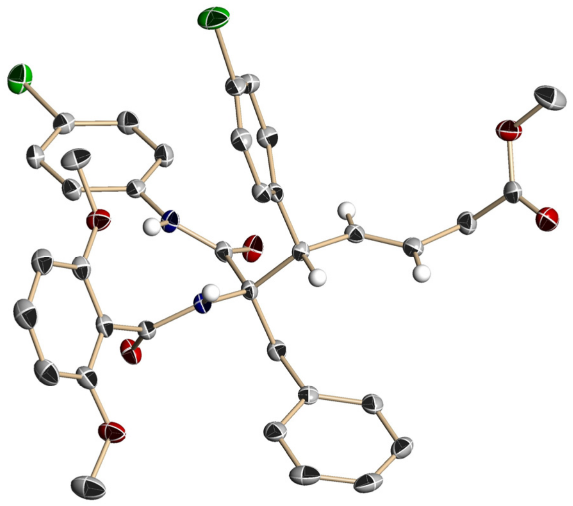

Supplementary Figure 102. Crystal data, structure refinement, and molecular structure of **10** (CCDC 1494060). The thermal ellipsoids of non-hydrogen atoms are shown at the 50% probability level. Calculated hydrogen atoms except them attached to olefinic and stereogenic carbons are omitted for clarity. Blue = nitrogen, red = oxygen, gray = carbon, green = chlorine.

Empirical formula	$C_{31}H_{33}N_1O_7$	
Formula weight	531.58	
Temperature	123.0(2) K	
Wavelength	0.71075 Å	
Crystal system	Triclinic	
Space group	P1	
Unit cell dimensions	a = 9.8460(15) Å	α = 73.576(8)°.
	b = 10.1027(13) Å	β = 81.882(9)°.
	c = 15.545(2) Å	γ = 67.752(6)°.
Volume	1371.8(3) $Å^3$	
Z	2	
Density (calculated)	1.287 Mg/m^3	
Absorption coefficient	0.091 mm^{-1}	
F(000)	564	
Crystal size	0.010 x 0.020 x 0.050 mm^3	
Theta range for data collection	3.076 to 25.499°.	
Index ranges	–11<=h<=11, –12<=k<=12, –18<=l<=15	
Reflections collected	9504	
Independent reflections	6980 [R(int) = 0.0164]	
Completeness to theta = 25.242°	95.9 %	
Absorption correction	Semi-empirical from equivalents	
Max. and min. transmission	1.000 and 0.933	
Refinement method	Full-matrix least-squares on F^2	
Data / restraints / parameters	6980 / 3 / 711	
Goodness-of-fit on F^2	0.975	
Final R indices [I>2sigma(I)]	R_1 = 0.0286, wR_2 = 0.0596	
R indices (all data)	R_1 = 0.0378, wR_2 = 0.0625	
Absolute structure parameter	0.3(4)	
Extinction coefficient	0	
Largest diff. peak and hole	0.155 and –0.185 e.$Å^{-3}$	

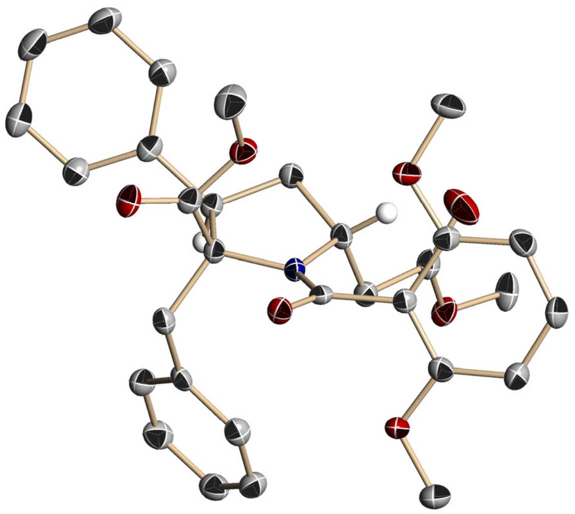

Supplementary Figure 103. Crystal data, structure refinement, and molecular structure of **14** (CCDC 1494058)
The thermal ellipsoids of non-hydrogen atoms are shown at the 50% probability level. Calculated hydrogen atoms except them attached to stereogenic carbons are omitted for clarity. Blue = nitrogen, red = oxygen, gray = carbon.

Empirical formula	$C_{31}H_{33}N_1O_7$
Formula weight	531.58
Temperature	123.0(2) K
Wavelength	0.71075 Å
Crystal system	Trigonal
Space group	P3(2)
Unit cell dimensions	a = 10.7136(11) Å α = 90°.
	b = 10.7136(11) Å β = 90°.
	c = 21.153(2) Å γ = 120°.
Volume	2102.7(5) $Å^3$
Z	3
Density (calculated)	1.259 Mg/m^3
Absorption coefficient	0.089 mm^{-1}
F(000)	846
Crystal size	0.60 x 0.60 x 0.60 mm^3
Theta range for data collection	3.629 to 25.500°.
Index ranges	–12<=h<=12, –12<=k<=11, –25<=l<=25
Reflections collected	14477
Independent reflections	5108 [R(int) = 0.0204]
Completeness to theta = 25.242°	99.6 %
Absorption correction	Semi-empirical from equivalents
Max. and min. transmission	1.000 and 0.917
Refinement method	Full-matrix least-squares on F^2
Data / restraints / parameters	5108 / 1 / 356
Goodness-of-fit on F^2	1.054
Final R indices [I>2sigma(I)]	R_1 = 0.0320, wR_2 = 0.0850
R indices (all data)	R_1 = 0.0340, wR_2 = 0.0860
Absolute structure parameter	0.1(2)
Extinction coefficient	0
Largest diff. peak and hole	0.245 and –0.191 e.$Å^{-3}$

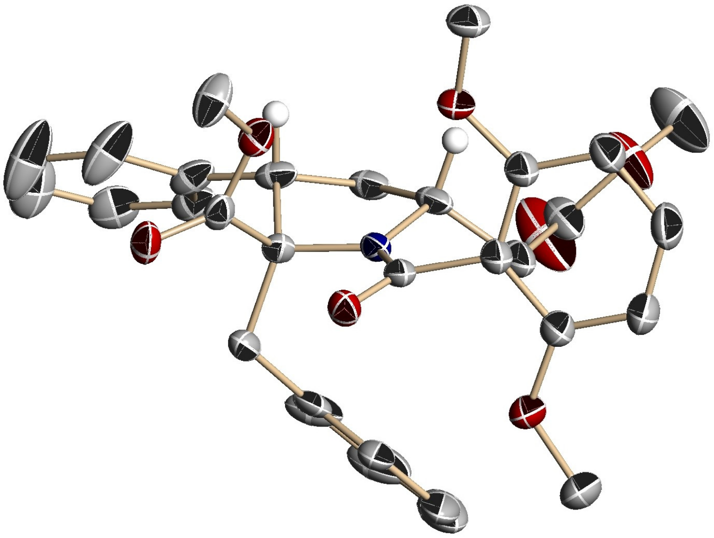

Supplementary Figure 104. Crystal data, structure refinement, and molecular structure of **16** (CCDC 1494059)
The thermal ellipsoids of non-hydrogen atoms are shown at the 50% probability level. Calculated hydrogen atoms except them attached to stereogenic carbons are omitted for clarity. Blue = nitrogen, red = oxygen, gray = carbon.

Empirical formula	$C_{36}H_{33}NO_5 \cdot CHCl_3$
Formula weight	679.00
Temperature	123.0(2) K
Wavelength	1.54187 Å
Crystal system	Orthothombic
Space group	P2(1)2(1)2(1)
Unit cell dimensions	a = 10.4072(2) Å α = 90°.
	b = 14.4661(3) Å β = 90°.
	c = 21.9250(4) Å γ = 90°.
Volume	3300.84(11) $Å^3$
Z	4
Density (calculated)	1.366 Mg/m^3
Absorption coefficient	2.879 mm^{-1}
F(000)	1416
Crystal size	0.250 x 0.250 x 0.250 mm^3
Theta range for data collection	3.661 to 68.249°.
Index ranges	–12<=h<=12, –17<=k<=17, –26<=l<=26
Reflections collected	60946
Independent reflections	6057 [R(int) = 0.0342]
Completeness to theta = 67.678°	100.0 %
Absorption correction	Semi-empirical from equivalents
Max. and min. transmission	1.000 and 0.916
Refinement method	Full-matrix least-squares on F^2
Data / restraints / parameters	6057 / 0 / 419
Goodness-of-fit on F^2	1.064
Final R indices [I>2sigma(I)]	R_1 = 0.0276, wR_2 = 0.0703
R indices (all data)	R_1 = 0.0284, wR_2 = 0.0707
Absolute structure parameter	–0.002(2)
Extinction coefficient	0.00232(16)
Largest diff. peak and hole	0.272 and –0.295 e.$Å^{-3}$

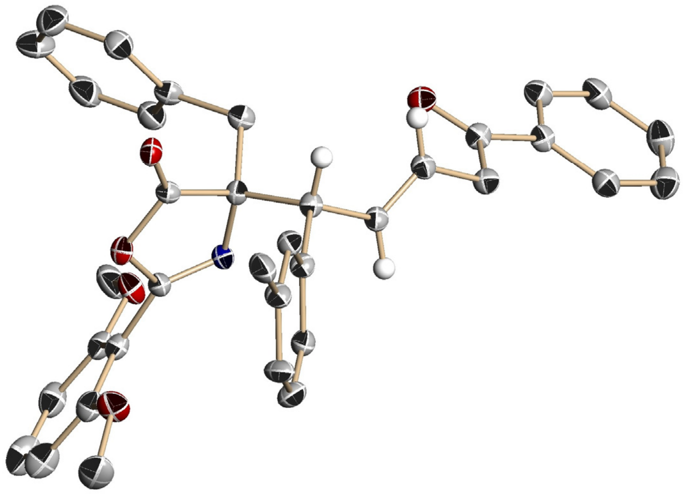

Supplementary Figure 105. Crystal data, structure refinement, and molecular structure of *RR*-**6ac** (CCDC 1520998) The thermal ellipsoids of non-hydrogen atoms are shown at the 50% probability level. Calculated hydrogen atoms except them attached to olefinic and stereogenic carbons are omitted for clarity. Blue = nitrogen, red = oxygen, gray = carbon.

Empirical formula	$2(C_{38}H_{48}N_4P)\cdot 2(Cl)\cdot H_2O$	
Formula weight	1272.46	
Temperature	123.0(2) K	
Wavelength	0.71075 Å	
Crystal system	Monoclinic	
Space group	P2(1)	
Unit cell dimensions	a = 13.167(2) Å	α = 90°.
	b = 18.308(3) Å	β = 98.277(3) °.
	c = 18.269(3) Å	γ = 90°.
Volume	4358.1(12) $Å^3$	
Z	2	
Density (calculated)	0.970 Mg/m^3	
Absorption coefficient	0.151 mm^{-1}	
F(000)	1364	
Crystal size	0.200 x 0.200 x 0.200 mm^3	
Theta range for data collection	3.029 to 25.500°.	
Index ranges	–15<=h<=15, –21<=k<=19, –15<=l<=22	
Reflections collected	30253	
Independent reflections	15180 [R(int) = 0.0432]	
Completeness to theta = 25.242°	99.3 %	
Absorption correction	Semi-empirical from equivalents	
Max. and min. transmission	1.000 and 0.829	
Refinement method	Full-matrix least-squares on F^2	
Data / restraints / parameters	15180 / 4 / 838	
Goodness-of-fit on F^2	1.016	
Final R indices [I>2sigma(I)]	R_1 = 0.0425, wR_2 = 0.1012	
R indices (all data)	R_1 = 0.0536, wR_2 = 0.1063	
Absolute structure parameter	0.03(3)	
Extinction coefficient	0	
Largest diff. peak and hole	0.363 and –0.254 e.$Å^{-3}$	

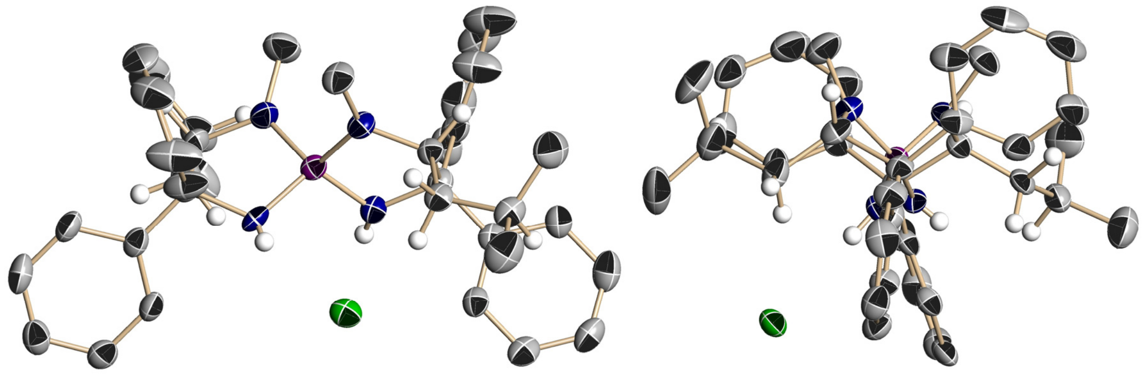

(a) Front view (b) Side view

Supplementary Figure 106. Crystal data, structure refinement, and molecular structure of **1aa**·HCl (CCDC 1520999) The thermal ellipsoids of non-hydrogen atoms are shown at the 50% probability level. Calculated hydrogen atoms except them attached, α-position, and β-position to stereogenic carbons are omitted for clarity. Blue = nitrogen, gray = carbon, green = chlorine, purple = phosphorus.

Empirical formula	$C_{36}H_{44}N_4P·C_3H_6O·Cl·H_2O$	
Formula weight	675.27	
Temperature	93.0(2) K	
Wavelength	0.71073 Å	
Crystal system	Orthorhombic	
Space group	P2(1)2(1)2(1)	
Unit cell dimensions	a = 12.3872(5) Å	α = 90°.
	b = 16.9120(7) Å	β = 90°.
	c = 17.8208(7) Å	γ = 90°.
Volume	3733.3(3) $Å^3$	
Z	4	
Density (calculated)	1.201 Mg/m^3	
Absorption coefficient	0.183 mm^{-1}	
F(000)	1448	
Crystal size	0.2 x 0.1 x 0.1 mm^3	
Theta range for data collection	2.286 to 25.495°.	
Index ranges	–15<=h<=14, –19<=k<=20, –19<=l<=21	
Reflections collected	26371	
Independent reflections	6900 [R(int) = 0.0572]	
Completeness to theta = 25.242°	99.7 %	
Absorption correction	Semi-empirical from equivalents	
Max. and min. transmission	0.982 and 0.978	
Refinement method	Full-matrix least-squares on F^2	
Data / restraints / parameters	6900 / 5 / 449	
Goodness-of-fit on F^2	1.165	
Final R indices [I>2sigma(I)]	R_1 = 0.0732, wR_2 = 0.1721	
R indices (all data)	R_1 = 0.0816, wR_2 = 0.1763	
Absolute structure parameter	0.11(3)	
Extinction coefficient	0	
Largest diff. peak and hole	1.281 and –0.476 e.$Å^{-3}$	

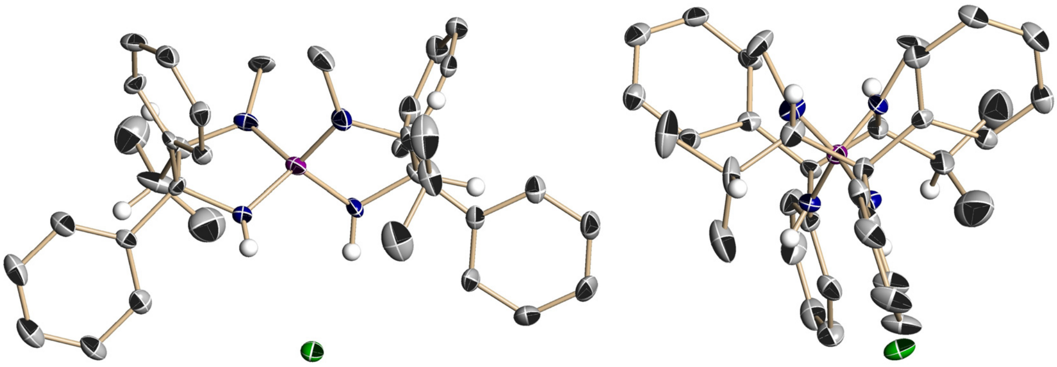

(a) Front view (b) Side view

Supplementary Figure 107. Crystal data, structure refinement, and molecular structure of **1ba**·HCl (CCDC 1520995) The thermal ellipsoids of non-hydrogen atoms are shown at the 50% probability level. Calculated hydrogen atoms except them attached and α-position to stereogenic carbons are omitted for clarity. Blue = nitrogen, gray = carbon, green = chlorine, purple = phosphorus.

Empirical formula	$2(C_{38}H_{48}N_4P)\cdot C_3H_6O\cdot 2(Cl)\cdot H_2O\cdot 2(O)$	
Formula weight	1362.54	
Temperature	123.0(2) K	
Wavelength	0.71075 Å	
Crystal system	Orthorhombic	
Space group	P2(1)2(1)2(1)	
Unit cell dimensions	a = 17.8512(18) Å	α = 90°.
	b = 17.9844(17) Å	β = 90°.
	c = 23.459(2) Å	γ = 90°.
Volume	7531.3(12) $Å^3$	
Z	4	
Density (calculated)	1.202 Mg/m^3	
Absorption coefficient	0.183 mm^{-1}	
F(000)	2920	
Crystal size	0.50 x 0.30 x 0.20 mm^3	
Theta range for data collection	3.061 to 25.497°.	
Index ranges	–21<=h<=21, –19<=k<=21, –28<=l<=28	
Reflections collected	49497	
Independent reflections	13884 [R(int) = 0.1359]	
Completeness to theta = 25.242°	99.6 %	
Absorption correction	Semi-empirical from equivalents	
Max. and min. transmission	1.000 and 0.690	
Refinement method	Full-matrix least-squares on F^2	
Data / restraints / parameters	13884 / 3 / 896	
Goodness-of-fit on F^2	0.994	
Final R indices [I>2sigma(I)]	R_1 = 0.0599, wR_2 = 0.1486	
R indices (all data)	R_1 = 0.0690, wR_2 = 0.1522	
Absolute structure parameter	0.08(5)	
Extinction coefficient	0	
Largest diff. peak and hole	1.127 and –0.792 e.$Å^{-3}$	

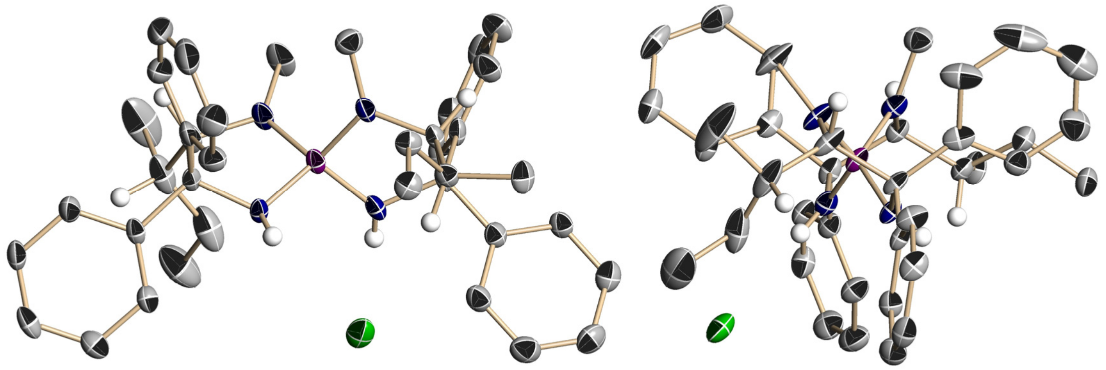

(a) Front view (b) Side view

Supplementary Figure 108. Crystal data, structure refinement, and molecular structure of **1da**·HCl (CCDC 1520997) The thermal ellipsoids of non-hydrogen atoms are shown at the 50% probability level. Calculated hydrogen atoms except them attached to stereogenic carbons are omitted for clarity. Blue = nitrogen, gray = carbon, green = chlorine, purple = phosphorus.

Empirical formula	$C_{38}H_{48}N_4P\cdot Cl\cdot H_2O$	
Formula weight	645.24	
Temperature	123.0(2) K	
Wavelength	0.71075 Å	
Crystal system	Tetragonal	
Space group	P4(3)2(1)2	
Unit cell dimensions	a = 22.8786(18) Å	α = 90°.
	b = 22.8786(18) Å	β = 90°.
	c = 13.6070(11) Å	γ = 90°.
Volume	7122.3(13) $Å^3$	
Z	8	
Density (calculated)	1.203 Mg/m^3	
Absorption coefficient	0.187 mm^{-1}	
F(000)	2768	
Crystal size	0.100 x 0.100 x 0.100 mm^3	
Theta range for data collection	3.062 to 25.499°.	
Index ranges	–27<=h<=22, –27<=k<=27, –16<=l<=16	
Reflections collected	47836	
Independent reflections	6635 [R(int) = 0.0307]	
Completeness to theta = 25.242°	99.6 %	
Absorption correction	Semi-empirical from equivalents	
Max. and min. transmission	1.000 and 0.845	
Refinement method	Full-matrix least-squares on F^2	
Data / restraints / parameters	6635 / 2 / 440	
Goodness-of-fit on F^2	1.050	
Final R indices [I>2sigma(I)]	R_1 = 0.0269, wR_2 = 0.0672	
R indices (all data)	R_1 = 0.0294, wR_2 = 0.0688	
Absolute structure parameter	–0.003(11)	
Extinction coefficient	0	
Largest diff. peak and hole	0.291 and –0.261 e.$Å^{-3}$	

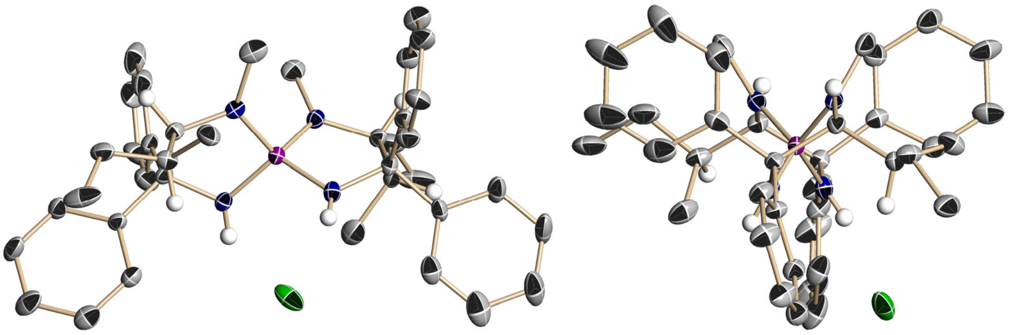

(a) Front view (b) Side view

Supplementary Figure 109. Crystal data, structure refinement, and molecular structure of *ent*-**1ea**·HCl (CCDC 1520996)

The thermal ellipsoids of non-hydrogen atoms are shown at the 50% probability level. Calculated hydrogen atoms except them attached to stereogenic carbons are omitted for clarity. Blue = nitrogen, gray = carbon, green = chlorine, purple = phosphorus.

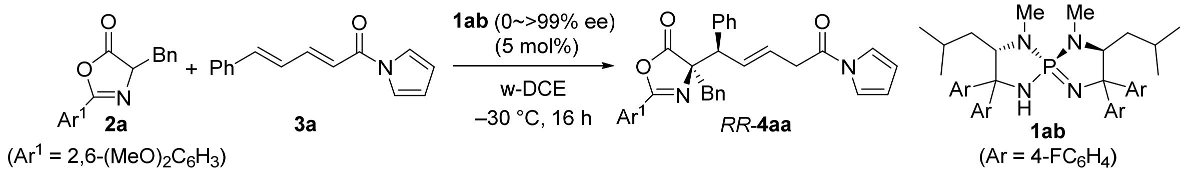

%ee of **1ab**	%yield of **4aa**	dr of **4aa**	%ee of *RR*-**4aa**
0	75	>20:1	0
20	83	>20:1	21
40	81	>20:1	38
59	95	>20:1	56
79	89	>20:1	77
100	88	>20:1	99

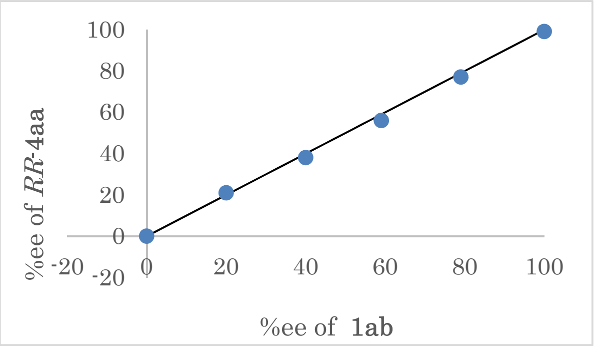

Supplementary Figure 110. Correlation between % ee of **1ab** and *RR*-**4aa**

This result shows a linear relationship between the enantiomeric excess (ee) of **1ab** and that of *RR*-**4aa**, which suggests that a single catalyst molecule is involved in the stereo-determining step.

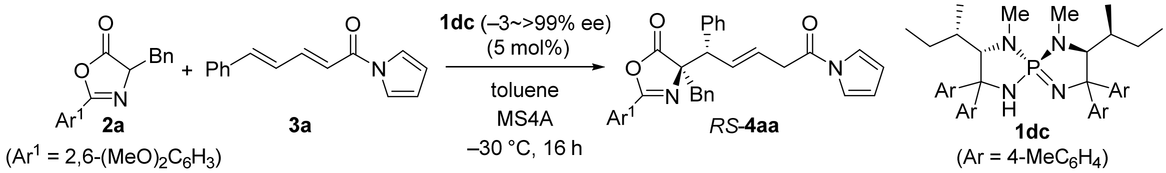

%ee of **1dc**	%yield of **4aa**	dr of **4aa**	%ee of *RS*-**4aa**
–3	96	1:20	–2
18	94	1:>20	14
38	94	1:20	31
66	96	1:20	62
85	90	1:20	81
100	89	1:>20	98

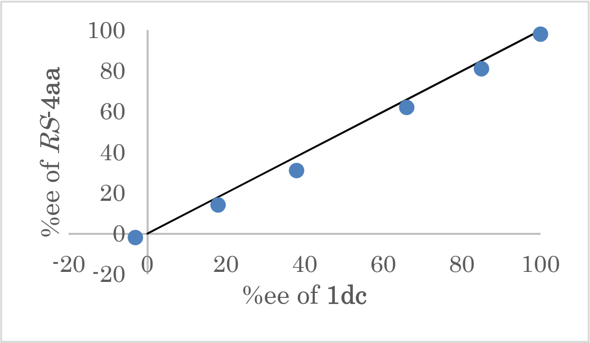

Supplementary Figure 111. Correlation between % ee of **1dc** and *RS*-**4aa** (MS4A = molecular sieves 4 Å)

This result shows a linear relationship between the enantiomeric excess (ee) of **1dc** and that of *RS*-**4aa**, which suggests that a single catalyst molecule is involved in the stereo-determining step.

Supplementary Table 1. Optimization of Reaction Parameters*

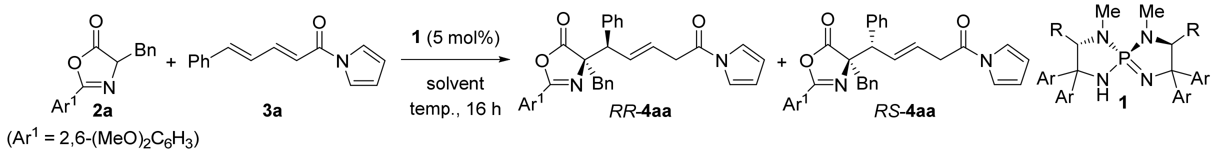

entry	R, Ar (**1**)	additive	solvent	temp (°C)	yield (%)†	rr (1,6:1,4)‡	dr (*RR*:*RS*)‡	ee (%)¶
1	^{i}Bu, Ph (**1aa**)	-	toluene	0	78	16:1	1:1.2	85/17
2	^{i}Bu, Ph (**1aa**)	MS4A	toluene	0	89	4.2:1	1:3.4	22/14
3	^{i}Bu, Ph (**1aa**)	-	w-toluene§	0	79	19:1	1.9:1	94/18
4	^{i}Bu, Ph (**1aa**)	MS4A, 3,5-$Cl_2C_6H_3OH$	toluene	0	94	12:1	1:2.3	69/14
5	^{i}Bu, Ph (**1aa**)	-	w-toluene§	–30	82	>20:1	7.2:1	99/31
6	^{i}Bu, Ph (**1aa**)	MS4A, 3,5-$Cl_2C_6H_3OH$	toluene	–30	96	>20:1	7.2:1	99/33
7	^{i}Bu, Ph (**1aa**)	MS4A	toluene	–30	95	>20:1	2.7:1	97/34
8	Me, Ph (**1fa**)	-	w-toluene§	–30	97	>20:1	4.7:1	94/61
9	^{i}Pr, Ph (**1ba**)	-	w-toluene§	–30	52	11:1	3.5:1	93/59
10	Bn, Ph (**1ca**)	-	w-toluene§	–30	95	>20:1	1:8.3	72/81
11	(*S*)-^{s}Bu, Ph (**1da**)	-	w-toluene§	–30	49	12:1	1:6.9	32/93
12	(*S*)-^{s}Bu, Ph (*ent*-**1ea**)‖	-	w-toluene§	–30	41	15:1	5.0:1	–97/–46
13	^{i}Pr, Ph (**1ba**)	MS4A	toluene	–30	93	14:1	3.4:1	95/64
14	(*S*)-^{s}Bu, Ph (*ent*-**1ea**)‖	MS4A	toluene	–30	97	>20:1	4.5:1	–98/–50
15	^{i}Bu, 4-FC_6H_4 (**1ab**)	-	w-toluene§	–30	91	>20:1	12:1	>99/28
16	^{i}Bu, 4-MeC_6H_4 (**1ac**)	-	w-toluene§	–30	94	>20:1	3.2:1	98/61
17	^{i}Bu, 3-FC_6H_4 (**1ad**)	-	w-toluene§	–30	65	>20:1	7.7:1	99/7
18	^{i}Bu, 3-MeC_6H_4 (**1ae**)	-	w-toluene§	–30	32	12:1	1:1.2	95/–31
19	^{i}Bu, 4-FC_6H_4 (**1ab**)	-	w-Et_2O§	–30	9	-	7.1:1	97/27
20	^{i}Bu, 4-FC_6H_4 (**1ab**)	-	w-CH_2Cl_2§	–30	76	>20:1	17:1	99/50
21	^{i}Bu, 4-FC_6H_4 (**1ab**)	-	w-DCE§	–30	88	>20:1	>20:1	99/-
22	^{i}Bu, 4-FC_6H_4 (**1ab**)	MS4A	DCE	–30	95	>20:1	18:1	99/41
23	^{i}Bu, Ph (**1aa**)	-	w-DCE§	–30	76	>20:1	11:1	96/53
24	^{i}Bu, Ph (**1aa**)	MS4A	DCE	–30	93	>20:1	10:1	98/61
25	(*S*)-^{s}Bu, Ph (**1da**)	MS4A	toluene	–30	96	>20:1	1:8.1	43/95
26	(*S*)-^{s}Bu, 4-FC_6H_4 (**1db**)	MS4A	toluene	–30	89	>20:1	1:7.0	74/97
27	(*S*)-^{s}Bu, 4-MeC_6H_4 (**1dc**)	MS4A	toluene	–30	89	>20:1	1:>20	-/98
28	(*S*)-^{s}Bu, 3-FC_6H_4 (**1dd**)	MS4A	toluene	–30	85	>20:1	1:5.8	56/94
29	(*S*)-^{s}Bu, 3-MeC_6H_4 (**1de**)	MS4A	toluene	–30	96	>20:1	1:6.0	10/84

MS4A = molecular sieves 4 Å, DCE = 1,2-dichloroethane

* Reactions were performed with 0.50 mmol of **2a** and 0.25 mmol of **3a** in 1.25 mL of solvent in the presence of **1** (5 mol%) for 16 h.

† Isolated yield is indicated.

‡ Ratios of regioisomers and diastereomers were determined by ^{1}H NMR (700 MHz) analysis of crude aliquot.

¶ Enantiomeric excess was analyzed by chiral stationary phase HPLC. Absolute configurations of *RR*- and *RS*-**4aa** were assigned according to the results of X-ray crystallographic analysis of *RR*-**4ad** and *RS*-**4ac**-derived **10** (See Supplymentary Figures 101 and 102).

§ Organic solvent was saturated with water.[1]

‖ D-Alloisoleucine-derived iminophosphorane was used.

Supplementary Discussion: Discussion about Supplementary Table 1

Additional results in the optimization process are shown in Supplementary Table 1. As described in the main text of the manuscript, the L-leucine-derived catalyst system was found to be sensitive to the moisture content (entries 1–3), and the use of w-toluene as solvent was beneficial for the establishment of the reliable protocol to obtain *RR*-**4aa** exclusively. Our interest in gaining an insight into the role of the water prompted us to examine the 1,6-addition with other protic additives in a defined molar ratio to L-leucine-derived iminophosphorane **1aa**. Specifically, we attempted the reactions in the presence of 3,5-dichlorophenol (1 equivalents to **1aa**) in dehydrated toluene with molecular sieves 4 Å (MS4A) for figuring out the selectivity profile induced by the *in-situ* generated, supramolecularly assembled aminophosphonium catalyst[2,3] in comparison with that observed with **1aa** as a sole catalyst in w-toluene. Although the selectivity was only slightly improved upon addition of the phenol at 0 °C (entry 4), all the selectivities were markedly enhanced to the identical level with those obtained with **1aa** in w-toluene at –30 °C (entries 5 and 6). Since structural perturbation of the dynamic ion-pair assembly would decrease at lower temperature, these observations imply that water molecules participate in the formation of a structurally defined ion-pair assembly relevant to the transition state. The impact of alkyl substituents (R) of **1** on the selectivities was evaluated in w-toluene at –30 °C with a series of α-amino acid-derived iminophosphoranes **1ba**–**fa** as catalysts (entries 8–12). While **1aa** turned out to be the most selective catalyst in providing *RR*-**4aa** (entry 5), diastereomeric *RS*-**4aa** was predominantly formed without detrimental effect on regio- and enantioselectivity by employing L-phenylalanine- and L-isoleucine-derived catalysts **1ca** and **1da** (entries 10 and 11). Noteworthy was that D-*allo*-isoleucine-derived iminophosphorane *ent*-**1ea** gave almost the same selectivity to that exerted by valine-derived **1ba** (entry 9 vs 12), which revealed the importance of the catalyst conformation regulated by the chirality of the pendant ^{s}Bu group. Finally, aromatic substituents (Ar) of **1aa** and **1da** as well as solvent system were properly adjusted to establish optimal protocols for the rigorous diastereodivergent catalysis for subsequent evaluation of substrate generality (entries 15–21, 25–29). It should be added that we examined the reactions with **1ba** and *ent*-**1ea** as catalysts in dehydrated toluene with MS4A because we conducted DFT calculations for elucidating the transition-state structures of the reactions with these catalysts under dry conditions (entries 13 and 14). Likewise, we checked the profile of the reactions with **1aa** in w-DCE and dehydrated DCE with MS4A and, to our surprise, a comparable yet high level of stereoselectivity was attained regardless of the presence and absence of water (entries 23 vs. 24), unlike the cases with toluene as solvent, where a considerable difference in diastereoselectivity was observed (entry 5 vs. 7). Furthermore, similar tendency was observed in the reactions with the optimal catalyst **1ab**, confirming that the effect of water was marginal when the reaction was performed with catalysts bearing α-branched aliphatic substituents or in DCE at –30 °C, while slightly improving diastereoselectivity (entry 21 vs. 22).

Supplementary Methods:

General Information: Infrared spectra were recorded on a Shimadzu IRAffinity-1 spectrometer. ^{1}H NMR spectra were recorded on a JEOL JNM-ECS400 (400 MHz), JEOL JNM-ECZ400S (400 MHz), JEOL JNM-ECA600II (600 MHz), JEOL JNM-ECA800 (800 MHz), and Varian INOVA-700 (700 MHz) spectrometers. Chemical shifts are reported in ppm from the solvent resonance (CD_3OD; 3.31 ppm) or tetramethylsilane (0.0 ppm) resonance as the internal standard [$(CD_3)_2CO$, $CDCl_3$]. Data are reported as follows: chemical shift, integration, multiplicity (s = singlet, d = doublet, t = triplet, q = quartet, m = multiplet, br = broad) and coupling constants (Hz). ^{13}C NMR spectra were recorded on a JEOL JNM-ECS400 (101 MHz), JEOL JNM-ECZ400S (101 MHz), and JEOL JNM-ECA600II (151 MHz) spectrometers with complete proton decoupling. Chemical shifts are reported in ppm from the solvent resonance as the internal standard [$(CD_3)_2CO$; 29.84 ppm, CD_3OD; 49.0 ppm, $CDCl_3$; 77.16 ppm]. ^{19}F NMR spectra were recorded on a JEOL JNM-ECS400 (376 MHz) spectrometer. Chemical shifts are reported in ppm from benzotrifluoride (–64.0 ppm) resonance as the external standard. ^{31}P NMR spectra were recorded on a JEOL JNM-ECS400 (162 MHz) spectrometer with complete proton decoupling. Chemical shifts are reported in ppm from H_3PO_4 (0.0 ppm) resonance as the external standard. Optical rotations were measured on a HORIBA SEPA-500 polarimeter. The high resolution mass spectra were conducted on Thermo Fisher Scientific Exactive. Microwave experiment was performed by using Anton-Paar Monowave 300. Analytical thin layer chromatography (TLC) was performed on Merck precoated TLC plates (silica gel 60 GF_{254}, 0.25 mm) and Chromatorex® TLC plates NH (0.2 mm; Fuji Silysia Chemical ltd.). TLC Plate-*WAKO* (silica gel 70 GF_{254}, 0.25 mm) was used for the preparative thin layer chromatography (PLC). Flash column chromatography was conducted on silica gel 60 (spherical, 40–50 μm; Kanto Chemical Co., Inc.), silica gel 60N (spherical, 40–50 μm; Kanto Chemical Co., Inc.), PSQ60AB (spherical, av. 55 μm; Fuji Silysia Chemical ltd.), Silica gel 60 (Merck 1.09385.9929, 230–400 mesh), and Chromatorex® NH DM2035 (spherical, 45–75 μm; Fuji Silysia Chemical ltd.). Enantiomeric excesses were determined by HPLC analysis using chiral columns [ϕ 4.6 mm x 250 mm, DAICEL CHIRALPAK AD-3 (AD3), CHIRALCEL OD-3 (OD3), CHIRALCEL OZ-3 (OZ3), CHIRALCEL OX-3 (OX3), CHIRALPAK IA-3 (IA3), CHIRALPAK IC-3 (IC3)] with hexane (H), 2-propanol (IPA), and ethanol (EtOH) as eluent.

Toluene, dichloromethane (CH_2Cl_2), diethyl ether (Et_2O), and tetrahydrofuran (THF) were supplied from Kanto Chemical Co., Inc. as "Dehydrated" and further purified by passing through neutral alumina under nitrogen atmosphere. Chiral tetraaminophosphonium salts **1**·HCl,[4] chiral triaminoiminophosphorane **1**,[4] azlactones **2**,[5] dienyl *N*-acylpyrroles **3**,[6] dienyl ketones **5**,[7] and dienylidene malonate **7**[8,9] were prepared by following the literature procedure. Powdered molecular sieves 4 Å (MS4A) was supplied from Sigma-Aldrich. Other simple chemicals were purchased and used as such.

Procedure for Preparation of Iminophosphorane 1ab (0~>99% ee)

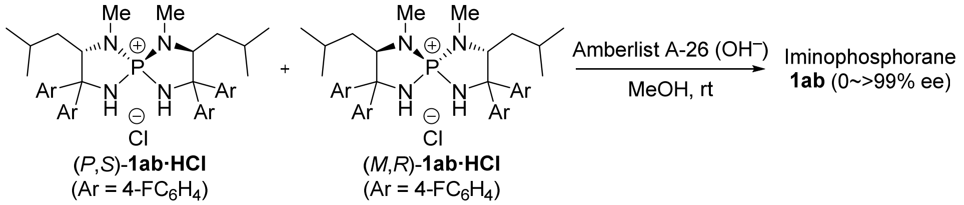

A mixture of chiral tetraaminophosphonium chlorides (*P*,*S*)-**1ab**·HCl and (*M*,*R*)-**1ab**·HCl in an appropriate ratio was placed in a sample tube, and the mixture was dissolved into methanol. The methanolic solution was passed through a column of ion exchange resin (Amberlyst A-26 OH form) to afford a solution of iminophosphorane **1ab** (0~>99% ee). The resulting solution was concentrated by rotary evaporation and residual solid was washed with deionized water on a funnel. The solid thus obtained was dried under reduced pressure to afford the iminophosphorane **1ab** as a white solid. Enantiomeric excess of iminophosphorane **1ab** was determined as shown below.

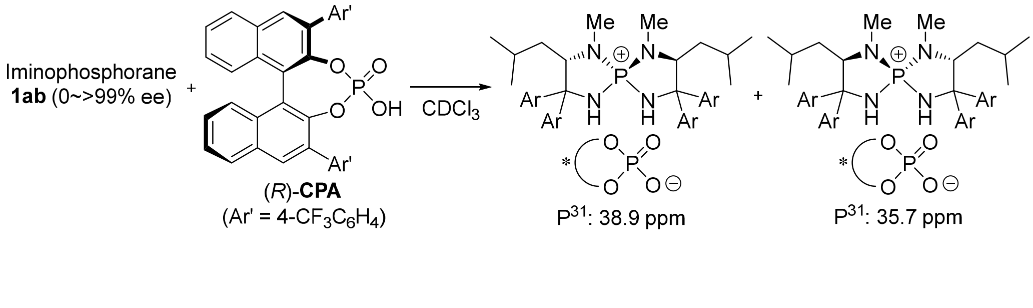

A mixture of iminophosphorane **1ab** (0~>99% ee) and chiral phosphoric acid (*R*)-**CPA** was dissolved into $CDCl_3$. Enantiomeric excess of **1ab** was determined by the ratio of the integration of diastereomeric phosphonium salts in ^{31}P NMR.

Experiments for investigating correlation between the enantiomeric excess of catalyst **1ab** or **1dc** and that of the 1,6-adduct *RR*-**4aa** or *RS*-**4aa** followed the representative procedure (see Methods in the main manuscript).

Characterization of Tetraaminophosphonium Salts 1·HCl:

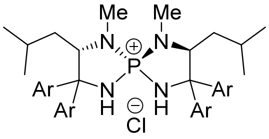

1ab·HCl
(Ar = 4-FC_6H_4)

1ab·HCl[4]**:** ^{1}H NMR (400 MHz, CD_3OD) δ 7.57 (4H, dd, $J_{H\text{-}H}$ = 8.7 Hz, $J_{F\text{-}H}$ = 5.0 Hz), 7.33 (4H, dd, $J_{H\text{-}H}$ = 8.7 Hz, $J_{F\text{-}H}$ = 5.5 Hz), 7.20 (4H, t, $J_{H\text{-}H}$ = $J_{F\text{-}H}$ = 8.7 Hz), 7.09 (4H, t, $J_{H\text{-}H}$ = $J_{F\text{-}H}$ = 8.7 Hz), 4.10 (2H, ddd, $J_{P\text{-}H}$ = 22.4 Hz, $J_{H\text{-}H}$ = 7.3, 4.6 Hz), 1.99 (6H, d, $J_{P\text{-}H}$ = 10.5 Hz), 1.42 (2H, ddd, *J* = 14.1, 7.3, 4.6 Hz), 1.26 (2H, ddd, *J* = 14.1, 7.3, 5.0 Hz), 0.81 (6H, d, *J* = 6.2 Hz), 0.64 (2H, m), 0.62 (6H, brs), N-H protons were not found due to deuterium exchange.

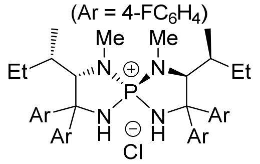

1dc·HCl
(Ar = 4-MeC_6H_4)

1dc·HCl: ^{1}H NMR (400 MHz, CD_3OD) δ 7.47 (4H, d, *J* = 8.2 Hz), 7.25 (4H, d, *J* = 8.2 Hz), 7.20 (4H, br), 7.13 (4H, d, *J* = 8.2 Hz), 4.00 (2H, dd, $J_{P\text{-}H}$ = 19.5 Hz, $J_{H\text{-}H}$ = 4.2 Hz), 2.34 (6H, s), 2.31 (6H, s), 1.90 (6H, d, $J_{P\text{-}H}$ = 10.1 Hz), 1.78 (2H, dqd, *J* = 13.3, 7.3, 2.6 Hz), 1.42 (2H, dqdd, *J* = 11.3, 6.9, 4.2, 2.6 Hz), 0.92 (2H, ddq, *J* = 13.3, 11.3, 7.3 Hz), 0.77 (6H, t, *J* = 7.3 Hz), 0.62 (6H, d, *J* = 6.9 Hz), N-H protons were not found due to deuterium exchange.; ^{13}C NMR (101 MHz, CD_3OD) δ 146.2, 139.0 (d, $J_{P\text{-}C}$ = 13.5 Hz), 138.9, 138.7, 130.4, 129.6, 128.7, 127.8, 72.9 (d, $J_{P\text{-}C}$ = 10.6 Hz), 71.1 (d, $J_{P\text{-}C}$ = 10.6 Hz), 37.7, 32.6 (d, $J_{P\text{-}C}$ = 6.8 Hz), 26.1, 21.0, 20.9, 18.9, 12.3; ^{31}P NMR (162 MHz, CD_3OD) δ 35.9; IR (film): 2972, 1508, 1456, 1362, 1348, 1175, 1022, 1011, 808 cm^{-1}; HRMS (ESI) Calcd for $C_{42}H_{56}N_4P^+$ ($[M–Cl]^+$) 647.4237. Found 647.4219.; $[\alpha]^{27}_D$ –223.6 (*c* = 1.00, CH_3OH).

Characterization of Triaminoiminophosphoranes 1:

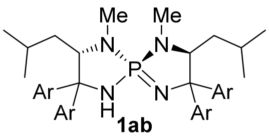

1ab
(Ar = 4-FC_6H_4)

1ab[4]**:** ^{1}H NMR (400 MHz, CD_3OD) δ 7.55 (4H, dd, $J_{H\text{-}H}$ = 8.8 Hz, $J_{F\text{-}H}$ = 5.0 Hz), 7.34 (4H, dd, $J_{H\text{-}H}$ = 8.8 Hz, $J_{F\text{-}H}$ = 5.5 Hz), 7.16 (4H, t, $J_{H\text{-}H}$ = $J_{F\text{-}H}$ = 8.8 Hz), 7.06 (4H, t, $J_{H\text{-}H}$ = $J_{F\text{-}H}$ = 8.8 Hz), 4.04 (2H, ddd, $J_{P\text{-}H}$= 22.4 Hz, $J_{H\text{-}H}$ = 7.4, 4.9 Hz), 1.96 (6H, d, $J_{P\text{-}H}$ = 10.6 Hz), 1.39 (2H, ddd, *J* = 14.2, 7.4, 4.9 Hz), 1.24 (2H, ddd, *J* = 14.2, 7.4, 4.9 Hz), 0.80 (6H, d, *J* = 6.0 Hz), 0.63 (2H, m), 0.61 (6H, brs), N-H proton was not found due to deuterium exchange.

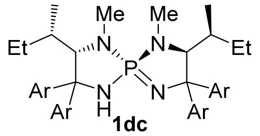

1dc
(Ar = 4-MeC_6H_4)

1dc: ^{1}H NMR (400 MHz, CD_3OD) δ 7.47 (4H, d, *J* = 8.3 Hz), 7.25 (4H, d, *J* = 8.3 Hz), 7.20 (4H, br), 7.13 (4H, d, *J* = 8.3 Hz), 4.00 (2H, dd, $J_{P\text{-}H}$ = 19.6 Hz, $J_{H\text{-}H}$ = 4.0 Hz), 2.34 (6H, s), 2.31 (6H, s), 1.89 (6H, d, $J_{P\text{-}H}$ = 10.4 Hz), 1.78 (2H, dqd, *J* = 13.2, 7.1, 2.7 Hz), 1.42 (2H, dqdd, *J* = 10.8, 6.9, 4.0, 2.7 Hz), 0.92 (2H, ddq, *J* = 13.2, 10.8, 7.1 Hz), 0.76 (6H, t, *J* = 7.1 Hz), 0.62 (6H, d, *J* = 6.9 Hz), N-H proton was not found due to deuterium exchange.; ^{13}C NMR (151 MHz, CD_3OD) δ 146.2, 139.1 (d, $J_{P\text{-}C}$ = 13.1 Hz), 138.9, 138.7, 130.4, 129.6, 128.7, 127.8, 73.0 (d, $J_{P\text{-}C}$ = 11.6 Hz), 71.2 (d, $J_{P\text{-}C}$ = 10.2 Hz), 37.7, 32.6 (d, $J_{P\text{-}C}$ = 7.2 Hz), 26.1, 21.0, 20.9, 18.9, 12.2; ^{31}P NMR (162 MHz, CD_3OD) δ 35.9; IR (film): 2918, 1609, 1508, 1456, 1375, 1327, 1190, 1099, 1018, cm^{-1}; HRMS (ESI) Calcd for $C_{42}H_{56}N_4P^+$ ($[M+H]^+$) 647.4237. Found 647.4229; $[\alpha]^{27}_D$ –228.3 (*c* = 1.00, CH_3OH).

Characterization of Azlactones 2 (Ar = 2,6-$(MeO)_2C_6H_3$):

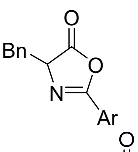

2a[4]**:** ^{1}H NMR (400 MHz, $CDCl_3$) δ 7.34 (1H, t, *J* = 8.5 Hz), 7.32-7.21 (5H, m), 6.52 (2H, d, *J* = 8.5 Hz), 4.70 (1H, dd, *J* = 6.4, 5.1 Hz), 3.72 (6H, s), 3.39 (1H, dd, *J* = 14.2, 5.1 Hz), 3.24 (1H, dd, *J* = 14.2, 6.4 Hz).

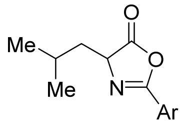

2b[4]**:** ^{1}H NMR (400 MHz, $CDCl_3$) δ 7.38 (1H, t, *J* = 8.4 Hz), 6.58 (2H, d, *J* = 8.4 Hz), 4.43 (1H, dd, *J* = 8.1, 6.0 Hz), 3.82 (6H, s), 2.06 (1H, nonet, *J* = 7.0 Hz), 1.89 (1H, ddd, *J* = 13.9, 7.0, 6.0 Hz), 1.74 (1H, ddd, *J* = 13.9, 8.1, 7.0 Hz), 1.03 (3H, d, *J* = 7.0 Hz), 1.00 (3H, d, *J* = 7.0 Hz).

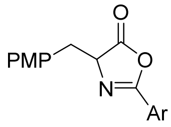

2c[4]**:** ^{1}H NMR (400 MHz, $CDCl_3$) δ 7.34 (1H, t, *J* = 8.7 Hz), 7.21 (2H, d, *J* = 8.9 Hz), 6.82 (2H, d, *J* = 8.9 Hz), 6.52 (2H, d, *J* = 8.7 Hz), 4.67 (1H, dd, *J* = 6.0, 5.0 Hz), 3.78 (3H, s), 3.72 (6H, s), 3.33 (1H, dd, *J* = 14.2, 5.0 Hz), 3.21 (1H, dd, *J* = 14.2, 6.0 Hz).

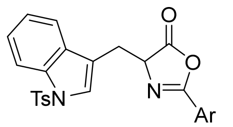

2d: ^{1}H NMR (600 MHz, CDCl$_3$) δ 7.94 (1H, d, J = 7.7 Hz), 7.66 (2H, d, J = 8.2 Hz), 7.58 (1H, d, J = 7.7 Hz), 7.57 (1H, s), 7.31 (1H, t, J = 8.5 Hz), 7.26 (1H, t, J = 7.7 Hz), 7.19 (1H, t, J = 7.7 Hz), 7.06 (2H, d, J = 8.2 Hz), 6.46 (2H, d, J = 8.5 Hz), 4.73 (1H, t, J = 5.1 Hz), 3.50 (6H, s), 3.44 (1H, dd, J = 14.8, 5.1 Hz), 3.37 (1H, dd, J = 14.8, 5.1 Hz); ^{13}C NMR (151 MHz, CDCl$_3$) δ 178.4, 159.4, 159.2, 144.7, 135.2, 135.0, 132.9, 131.2, 129.8, 126.7, 125.5, 124.6, 123.3, 120.1, 116.7, 113.5, 105.5, 103.6, 65.7, 55.9, 26.0, 21.5; IR (film): 2938, 2841, 1809, 1674, 1595, 1476, 1447, 1433, 1364, 1304, 1258, 1171, 1111, 1020, 907 cm^{-1}; HRMS (ESI) Calcd for $C_{27}H_{25}N_2O_6S$ ([M+H]$^+$) 505.1428. Found 505.1418.

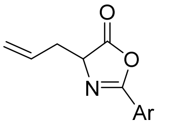

2e: ^{1}H NMR (400 MHz, CDCl$_3$) δ 7.38 (1H, t, J = 8.6 Hz), 6.58 (2H, d, J = 8.6 Hz), 5.87 (1H, ddt, J = 16.9, 10.2, 6.6 Hz), 5.25 (1H, dq, J = 16.9, 1.2 Hz), 5.18 (1H, d, J = 10.2 Hz), 4.50 (1H, t, J = 6.6 Hz), 3.82 (6H, s), 2.84 (1H, dt, J = 14.2, 6.6 Hz), 2.66 (1H, dt, J = 14.2, 6.6 Hz); ^{13}C NMR (101 MHz, CDCl$_3$) δ 178.5, 159.2, 159.1, 133.0, 131.7, 119.3, 106.0, 103.8, 65.4, 56.1, 35.2; IR (film): 2940, 2841, 1813, 1674, 1593, 1476, 1433, 1304, 1256, 1109, 1015, 883 cm^{-1}; HRMS (ESI) Calcd for $C_{14}H_{15}NO_4Na$ ([M+Na]$^+$) 284.0893. Found 284.0887.

Characterization of δ-Aryl Dienyl *N*-Acylpyrroles 3:

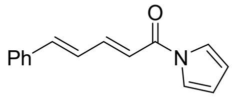

3a[10]: ^{1}H NMR (400 MHz, CDCl$_3$) δ 7.77 (1H, ddd, J = 15.0, 8.0, 2.6 Hz), 7.51 (2H, d, J = 7.8, Hz), 7.42 (2H, t, J = 2.3 Hz), 7.39 (2H, t, J = 7.8 Hz), 7.34 (1H, t, J = 7.8 Hz), 7.09-6.96 (2H, m), 6.72 (1H, d, J = 15.0 Hz), 6.34 (2H, t, J = 2.3 Hz).

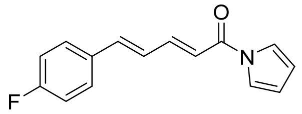

3b: ^{1}H NMR (400 MHz, CDCl$_3$) δ 7.74 (1H, dd, J = 14.9, 10.3 Hz), 7.48 (2H, dd, $J_{\text{H-H}}$ = 8.9 Hz, $J_{\text{F-H}}$ = 5.5 Hz), 7.42 (2H, t, J = 2.3 Hz), 7.07 (2H, t, $J_{\text{H-H}}$ = $J_{\text{F-H}}$ = 8.9 Hz), 7.00 (1H, d, J = 15.7 Hz), 6.93 (1H, dd, J = 15.7, 10.3 Hz), 6.71 (1H, d, J = 14.9 Hz), 6.34 (2H, t, J = 2.3 Hz); ^{13}C NMR (101 MHz, CDCl$_3$) δ 163.5 (d, $J_{\text{F-C}}$ = 254.5 Hz), 163.1, 147.4, 141.3, 132.2 (d, $J_{\text{F-C}}$ = 2.9 Hz), 129.3 (d, $J_{\text{F-C}}$ = 8.7 Hz), 126.0 (d, $J_{\text{F-C}}$ = 2.9 Hz), 119.3, 118.8, 116.2 (d, $J_{\text{F-C}}$ = 22.3 Hz), 113.4; ^{19}F NMR (376 MHz, CDCl$_3$) δ –110.6; IR (film): 3142, 1672, 1591, 1506, 1464, 1402, 1354, 1290, 1227, 1123, 991, 935 cm^{-1}; HRMS (ESI) Calcd for $C_{15}H_{13}NOF$ ([M+H]$^+$) 242.0976. Found 242.0976.

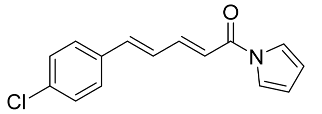

3c: ^{1}H NMR (400 MHz, CDCl$_3$) δ 7.73 (1H, ddd, J = 14.8, 6.3, 4.2 Hz), 7.46-7.38 (4H, m), 7.35 (2H, d, J = 8.7 Hz), 7.04-6.92 (2H, m), 6.73 (1H, d, J = 14.8 Hz), 6.35 (2H, t, J = 2.3 Hz); ^{13}C NMR (101 MHz, CDCl$_3$) δ 163.0, 147.2, 141.1, 135.4, 134.4, 129.3, 128.7, 126.7, 119.3, 113.5, one carbon atom was not found propably due to overlapping.; IR (film): 3146, 1672, 1601, 1470, 1402, 1356, 1292, 1125, 1092, 1065, 991, 939 cm^{-1}; HRMS (ESI) Calcd for $C_{15}H_{13}NOCl$ ([M+H]$^+$) 258.0680. Found 258.0682.

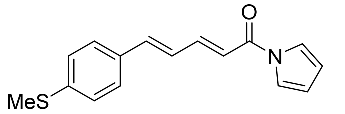

3d: ^{1}H NMR (400 MHz, CDCl$_3$) δ 7.75 (1H, ddd, J = 14.7, 6.5, 3.9 Hz), 7.42_0 (2H, d, J = 8.2 Hz), 7.41_6 (2H, t, J = 2.4 Hz), 7.23 (2H, d, J = 8.2 Hz), 7.00 (1H, dd, J = 15.6, 3.9 Hz), 6.96 (1H, dd, J = 15.6, 6.5 Hz), 6.70 (1H, d, J = 14.7 Hz), 6.34 (2H, t, J = 2.4 Hz), 2.51 (3H, s); ^{13}C NMR (101 MHz, CDCl$_3$) δ 163.2, 147.7, 142.1, 141.0, 132.5, 127.9, 126.3, 125.4, 119.3, 118.3, 113.3, 15.4; IR (film): 3140, 2918, 1692, 1582, 1466, 1406, 1356, 1287, 1115, 1059, 989, 935, 829 cm^{-1}; HRMS (ESI) Calcd for $C_{16}H_{16}NOS$ ([M+H]$^+$) 270.0947. Found 270.0948.

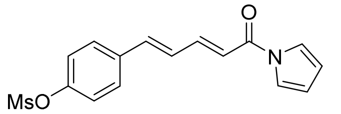

3e: ^{1}H NMR (400 MHz, CDCl$_3$) δ 7.73 (1H, ddd, J = 14.9, 6.9, 3.7 Hz), 7.54 (2H, d, J = 8.7 Hz), 7.42 (2H, t, J = 2.4 Hz), 7.30 (2H, d, J = 8.7 Hz), 7.05-6.93 (2H, m), 6.75 (1H, d, J = 14.9 Hz), 6.35 (2H, t, J = 2.4 Hz), 3.17 (3H, s); ^{13}C NMR (101 MHz, CDCl$_3$) δ 163.0, 149.6, 146.9, 140.5, 135.2, 129.0, 127.3, 122.6, 119.7, 119.3, 113.5, 37.7; IR (film): 3146, 3022, 2941, 1674, 1591, 1470, 1348, 1296, 1157, 1125, 991, 941, 839 cm^{-1}; HRMS (ESI) Calcd for $C_{16}H_{16}NO_4S$ ([M+H]$^+$) 318.0795. Found 318.0794.

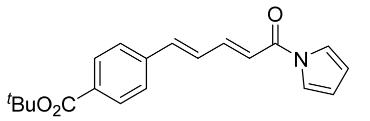

3f: ^{1}H NMR (400 MHz, CDCl$_3$) δ 7.99 (2H, d, J = 8.5 Hz), 7.76 (1H, dd, J = 14.9, 9.6 Hz), 7.53 (2H, d, J = 8.5 Hz), 7.42 (2H, t, J = 2.3 Hz), 7.10 (1H, dd, J = 15.9, 9.6 Hz), 7.04 (1H, d, J = 15.9 Hz), 6.78 (1H, d, J = 14.9 Hz), 6.35 (2H, t, J = 2.3 Hz), 1.61 (9H, s); ^{13}C NMR (101 MHz, CDCl$_3$) δ 165.3, 163.0, 146.9, 141.3, 139.6, 132.6, 130.1, 128.2, 127.2, 120.0, 119.3, 113.5, 81.5, 28.3; IR (film): 3142, 2992, 1703, 1674, 1597, 1472, 1414, 1360, 1292, 1163, 1128, 988, 937 cm^{-1}; HRMS (ESI) Calcd for $C_{20}H_{22}NO_3$ ([M+H]$^+$) 324.1594. Found 324.1594.

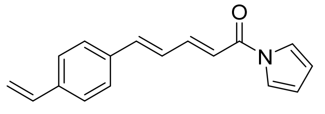

3g: ^{1}H NMR (400 MHz, CDCl$_3$) δ 7.76 (1H, ddd, J = 14.8, 5.6, 5.0 Hz), 7.47 (2H, d, J = 8.7 Hz), 7.42_3 (2H, d, J = 8.7 Hz), 7.42_2 (2H, t, J = 2.3 Hz), 7.04 (1H, dd, J = 15.8, 5.0 Hz), 7.00 (1H, dd, J = 15.8, 5.6 Hz), 6.72_1 (1H, d, J = 14.8 Hz), 6.72_0 (1H, dd, J = 17.7, 11.1 Hz), 6.35 (2H, t, J = 2.3 Hz), 5.81 (1H, d, J = 17.7 Hz), 5.31 (1H, d, J = 11.1 Hz); ^{13}C NMR (101 MHz, CDCl$_3$) δ 163.2, 147.7, 142.2, 138.9, 136.3, 135.4, 127.8, 126.9, 126.1, 119.3, 118.7, 115.1, 113.4;

IR (film): 3140, 1674, 1593, 1468, 1400, 1360, 1283, 1121, 1069, 995, 939, 843 cm^{-1}; HRMS (ESI) Calcd for $C_{17}H_{16}NO$ ($[M+H]^+$) 250.1226. Found 250.1227.

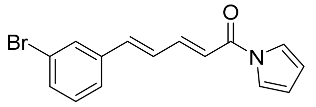

3h: ^1H NMR (400 MHz, $CDCl_3$) δ 7.73 (1H, dd, J = 15.0, 10.4 Hz), 7.65 (1H, s), 7.45 (1H, d, J = 7.8 Hz), 7.41 (2H, t, J = 2.4 Hz), 7.40 (1H, d, J = 7.8 Hz), 7.25 (1H, t, J = 7.8 Hz), 7.01 (1H, dd, J = 15.9, 10.4 Hz), 6.93 (1H, d, J = 15.9 Hz), 6.75 (1H, d, J = 15.0 Hz), 6.35 (2H, t, J = 2.4 Hz); ^{13}C NMR (101 MHz, $CDCl_3$) δ 162.9, 146.8, 140.6, 138.0, 132.3, 130.5, 130.1, 127.5, 126.2, 123.2, 119.8, 119.3, 113.5; IR (film): 3146, 1682, 1601, 1464, 1354, 1287, 1121, 1070, 995, 868 cm^{-1}; HRMS (ESI) Calcd for $C_{15}H_{13}NO^{79}Br$ ($[M+H]^+$) 302.0175. Found 302.0175.

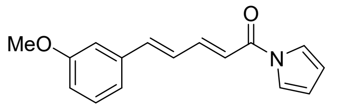

3i: ^1H NMR (400 MHz, $CDCl_3$) δ 7.75 (1H, dt, J = 14.9, 5.4 Hz), 7.42 (2H, t, J = 2.4 Hz), 7.30 (1H, t, J = 8.0 Hz), 7.10 (1H, d, J = 8.0 Hz), 7.02_{26} (1H, dd, J = 16.3, 5.4 Hz), 7.02_{21} (1H, t, J = 1.8 Hz), 6.98 (1H, dd, J = 16.3, 5.4 Hz), 6.90 (1H, dd, J = 8.0, 1.8 Hz), 6.72 (1H, d, J = 14.9 Hz), 6.34 (2H, t, J = 2.4 Hz), 3.84 (3H, s); ^{13}C NMR (101 MHz, $CDCl_3$) δ 163.1, 160.0, 147.5, 142.5, 137.3, 130.0, 126.5, 120.3, 119.3, 118.9, 115.4, 113.4, 112.5, 55.4; IR (film): 3142, 2963, 1672, 1605, 1576, 1470, 1433, 1360, 1267, 1125, 1042, 993, 941 cm^{-1}; HRMS (ESI) Calcd for $C_{16}H_{16}NO_2$ ($[M+H]^+$) 254.1176. Found 254.1176.

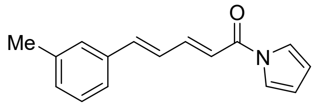

3j: ^1H NMR (400 MHz, $CDCl_3$) δ 7.76 (1H, ddd, J = 15.0, 5.9, 4.9 Hz), 7.42 (2H, t, J = 2.3 Hz), 7.32_3 (1H, s), 7.31_6 (1H, d, J = 7.3 Hz), 7.27 (1H, t, J = 7.3 Hz), 7.16 (1H, d, J = 7.3 Hz), 7.03 (1H, dd, J = 15.9, 5.9 Hz), 6.99 (1H, dd, J = 15.9, 4.9 Hz), 6.71 (1H, d, J = 15.0 Hz), 6.34 (2H, t, J = 2.3 Hz), 2.38 (3H, s); ^{13}C NMR (101 MHz, $CDCl_3$) δ 163.2, 147.8, 142.9, 138.7, 135.9, 130.5, 128.9, 128.3, 126.1, 124.8, 119.3, 118.6, 113.3, 21.5; IR (film): 3146, 2916, 1674, 1593, 1464, 1404, 1350, 1285, 1128, 1070, 991, 939 cm^{-1}; HRMS (ESI) Calcd for $C_{16}H_{16}NO$ ($[M+H]^+$) 238.1226. Found 238.1228.

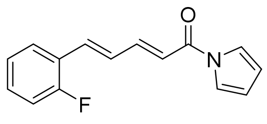

3k: ^1H NMR (400 MHz, $CDCl_3$) δ 7.76 (1H, dd, J = 14.9, 9.8 Hz), 7.53 (1H, td, J = 7.8, 1.4 Hz), 7.42 (2H, t, J = 2.3 Hz), 7.31 (1H, tdd, $J_{H\text{-}H}$ = 7.8, 1.6 Hz, $J_{F\text{-}H}$ = 5.8 Hz), 7.16_0 (1H, d, J = 15.6 Hz), 7.15_8 (1H, td, $J_{H\text{-}H}$ = 7.8, 1.6 Hz, $J_{F\text{-}H}$ = 7.8 Hz), 7.09_9 (1H, dd, J = 15.6, 9.8 Hz), 7.09_2 (1H, ddd, $J_{H\text{-}H}$ = 7.8, 1.4 Hz, $J_{F\text{-}H}$ = 11.0 Hz), 6.74 (1H, d, J = 14.9 Hz), 6.34 (2H, t, J = 2.3 Hz); ^{13}C NMR (101 MHz, $CDCl_3$) δ 163.0, 161.0 (d, $J_{F\text{-}C}$ = 256.4 Hz), 147.6, 135.0 (d, $J_{F\text{-}C}$ = 1.9 Hz), 130.9 (d, $J_{F\text{-}C}$ = 8.7 Hz), 128.7 (d, $J_{F\text{-}C}$ = 6.8 Hz), 128.4 (d, $J_{F\text{-}C}$ = 2.9 Hz), 124.5 (d, $J_{F\text{-}C}$ = 2.9 Hz), 123.9 (d, $J_{F\text{-}C}$ = 1.5 Hz), 119.4 (d, $J_{F\text{-}C}$ = 25.2 Hz), 119.3, 116.3 (d, $J_{F\text{-}C}$ = 22.3 Hz), 113.4; ^{19}F NMR (376 MHz, $CDCl_3$) δ –115.3; IR (film): 3142, 1670, 1595, 1456, 1406, 1358, 1281, 1229, 1123, 993, 937 cm^{-1}; HRMS (ESI) Calcd for $C_{15}H_{13}NOF$ ($[M+H]^+$) 242.0976. Found 242.0975.

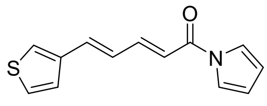

3l: ^1H NMR (400 MHz, $CDCl_3$) δ 7.72 (1H, dd, J = 15.0, 11.4 Hz), 7.41 (2H, t, J = 2.3 Hz), 7.40 (1H, dd, J = 3.0, 1.1 Hz), 7.34 (1H, dd, J = 5.3, 3.0 Hz), 7.31 (1H, dd, J = 5.3, 1.1 Hz), 7.04 (1H, d, J = 15.5 Hz), 6.84 (1H, dd, J = 15.5, 11.4 Hz), 6.69 (1H, d, J = 15.0 Hz), 6.34 (2H, t, J = 2.3 Hz); ^{13}C NMR (101 MHz, $CDCl_3$) δ 163.2, 147.8, 139.1, 136.3, 127.0, 126.3, 126.2, 125.0, 119.3, 118.3, 113.3; IR (film): 3140, 1672, 1597, 1510, 1466, 1418, 1354, 1281, 1123, 993, 932 cm^{-1}; HRMS (ESI) Calcd for $C_{13}H_{12}NOS$ ($[M+H]^+$) 230.0634. Found 230.0635.

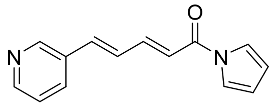

3m: ^1H NMR (400 MHz, $CDCl_3$) δ 8.74 (1H, d, J = 1.1 Hz), 8.56 (1H, dd, J = 4.8, 1.1 Hz), 7.81 (1H, d, J = 8.1 Hz), 7.75 (1H, dd, J = 15.0, 10.3 Hz), 7.42 (2H, t, J = 2.2 Hz), 7.32 (1H, dd, J = 8.1, 4.8 Hz), 7.08 (1H, dd, J = 15.9, 10.3 Hz), 7.01 (1H, d, J = 15.9 Hz), 6.78 (1H, d, J = 15.0 Hz), 6.36 (2H, t, J = 2.2 Hz); ^{13}C NMR (101 MHz, $CDCl_3$) δ 162.9, 150.2, 149.2, 146.6, 138.4, 133.7, 131.6, 128.1, 123.8, 120.2, 119.3, 113.6; IR (film): 3134, 1682, 1603, 1470, 1427, 1362, 1281, 1128, 1067, 993, 937 cm^{-1}; HRMS (ESI) Calcd for $C_{14}H_{13}N_2O$ ($[M+H]^+$) 225.1022. Found 225.1021.

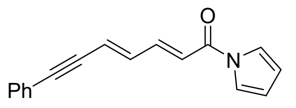

3n: ^1H NMR (400 MHz, $CDCl_3$) δ 7.63 (1H, dd, J = 14.8, 11.5 Hz), 7.51-7.44 (2H, m), 7.39 (2H, t, J = 2.3 Hz), 7.37-7.33 (3H, m), 6.87 (1H, dd, J = 15.2, 11.5 Hz), 6.69 (1H, d, J = 14.8 Hz), 6.34 (2H, t, J = 2.3 Hz), 6.33 (1H, d, J = 15.2 Hz); ^{13}C NMR (101 MHz, $CDCl_3$) δ 162.8, 146.0, 138.4, 131.9, 129.2, 128.6, 122.7, 121.6, 120.4, 119.3, 113.6, 97.6, 88.4; IR (film): 3146, 3067, 3032, 2191, 1678, 1599, 1470, 1358, 1288, 1125, 989 cm^{-1}; HRMS (ESI) Calcd for $C_{17}H_{13}NONa$ ($[M+Na]^+$) 270.0889. Found 270.0891.

Characterization of 1,6-Adduct 4 (see Methods in the main manuscript)**:**

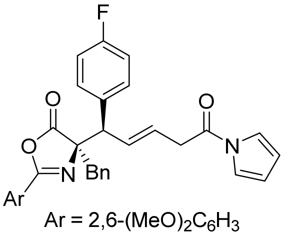

Ar = 2,6-(MeO)$_2$C$_6$H$_3$

RR-**4ab:** HPLC OZ3, H/IPA = 10:1, flow rate = 1.0 mL/min, λ = 254 nm, 12.6 min (minor diastereomer), 14.1 min (minor diastereomer), 16.0 min (minor isomer of major diastereomer), 53.9 min (major isomer of major diastereomer); ^{1}H NMR (400 MHz, CDCl$_3$) δ 7.32 (2H, dd, $J_{\text{H-H}}$ = 8.9 Hz, $J_{\text{F-H}}$ = 5.5 Hz), 7.32-7.27 (2H, brs), 7.28 (1H, t, J = 8.7 Hz), 7.24-7.15 (5H, m), 6.94 (2H, t, $J_{\text{H-H}}$ = $J_{\text{F-H}}$ = 8.9 Hz), 6.43 (2H, d, J = 8.7 Hz), 6.36 (1H, dd, J = 15.5, 10.1 Hz), 6.28 (2H, t, J = 2.5 Hz), 6.02 (1H, dt, J = 15.5, 6.7 Hz), 3.98 (1H, d, J = 10.1 Hz), 3.72 (1H, ddd, J = 17.2, 6.7, 1.3 Hz), 3.66 (1H, ddd, J = 17.2, 6.7, 1.3 Hz), 3.59 (6H, s), 3.42 (1H, d, J = 13.7 Hz), 3.16 (1H, d, J = 13.7 Hz); ^{13}C NMR (101 MHz, CDCl$_3$) δ 179.0, 168.4, 162.2 (d, $J_{\text{F-C}}$ = 248.7 Hz), 159.4, 158.2, 134.5, 133.8 (d, $J_{\text{F-C}}$ = 2.9 Hz), 133.0, 132.5, 131.2 (d, $J_{\text{F-C}}$ = 7.7 Hz), 130.9, 128.0, 127.0, 126.1, 119.2, 115.1 (d, $J_{\text{F-C}}$ = 22.3 Hz), 113.5, 104.9, 103.5, 78.2, 55.9, 55.2, 42.7, 38.4; ^{19}F NMR (376 MHz, CDCl$_3$) δ –115.4; IR (film): 3148, 2936, 2839, 1805, 1717, 1661, 1595, 1508, 1476, 1433, 1333, 1300, 1256, 1223, 1113, 964, 908 cm^{-1}; HRMS (ESI) Calcd for $C_{33}H_{30}N_2O_5F$ ([M+H]$^+$) 553.2133. Found 553.2131.

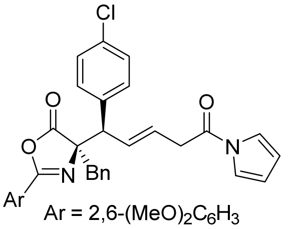

Ar = 2,6-(MeO)$_2$C$_6$H$_3$

RR-**4ac:** HPLC OD3, H/IPA = 10:1, flow rate = 1.0 mL/min, 40 °C, λ = 254 nm, 20.7 min (minor diastereomer), 22.8 min (major isomer of major diastereomer), 36.7 min (minor isomer of major diastereomer), 41.7 min (minor diastereomer); ^{1}H NMR (400 MHz, CDCl$_3$) δ 7.33-7.26 (5H, m), 7.24-7.16 (7H, m), 6.43 (2H, d, J = 8.5 Hz), 6.35 (1H, ddt, J = 15.4, 10.1, 1.1 Hz), 6.28 (2H, t, J = 2.3 Hz), 6.02 (1H, dt, J = 15.4, 6.8 Hz), 3.96 (1H, d, J = 10.1 Hz), 3.72 (1H, ddd, J = 17.2, 6.8, 1.1 Hz), 3.66 (1H, ddd, J = 17.2, 6.8, 1.1 Hz), 3.59 (6H, s), 3.43 (1H, d, J = 13.8 Hz), 3.17 (1H, d, J = 13.8 Hz); ^{13}C NMR (101 MHz, CDCl$_3$) δ 178.9, 168.3, 159.4, 158.3, 136.5, 134.4, 133.3, 133.1, 132.2, 130.9$_1$, 130.8$_7$, 128.4, 128.0, 127.1, 126.3, 119.2, 113.5, 104.8, 103.5, 78.1, 55.9, 55.3, 42.7, 38.4; IR (film): 3030, 2936, 2839, 1805, 1715, 1659, 1595, 1476, 1332, 1302, 1256, 1113, 964, 908 cm^{-1}; HRMS (ESI) Calcd for $C_{33}H_{30}N_2O_5Cl$ ([M+H]$^+$) 569.1838. Found 569.1835.

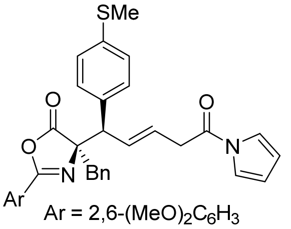

Ar = 2,6-(MeO)$_2$C$_6$H$_3$

RR-**4ad:** The absolute configuration of *RR*-**4ad** was determined by X-ray crystallographic analysis (see Supplymentary Figure 101). HPLC OX3, H/IPA/EtOH = 18:1:1, flow rate = 1.0 mL/min, λ = 254 nm, 30.6 min (minor diastereomer), 33.8 min (*SS*-isomer), 36.5 min (minor diastereomer), 45.6 min (*RR*-isomer); ^{1}H NMR (400 MHz, CDCl$_3$) δ 7.29 (2H, brs), 7.28 (2H, d, J = 8.2 Hz), 7.27 (1H, t, J = 8.1 Hz), 7.24-7.16 (5H, m), 7.13 (2H, d, J = 8.2 Hz), 6.43 (2H, d, J = 8.1 Hz), 6.36 (1H, ddt, J = 15.5, 10.1, 1.3 Hz), 6.27 (2H, t, J = 2.3 Hz), 6.00 (1H, dt, J = 15.5, 6.9 Hz), 3.94 (1H, d, J = 10.1 Hz), 3.71 (1H, ddd, J = 17.2, 6.9, 1.3 Hz), 3.65 (1H, ddd, J = 17.2, 6.9, 1.3 Hz), 3.59 (6H, s), 3.42 (1H, d, J = 13.7 Hz), 3.17 (1H, d, J = 13.7 Hz), 2.43 (3H, s); ^{13}C NMR (101 MHz, CDCl$_3$) δ 179.0, 168.4, 159.4, 158.1, 137.4, 134.7, 134.5, 133.0, 132.6, 130.9, 129.9, 127.9, 126.9, 126.2, 125.9, 119.2, 113.4, 104.9, 103.5, 78.2, 55.9, 55.5, 42.8, 38.4, 15.6; IR (film): 3030, 2924, 2837, 1805, 1715, 1661, 1595, 1476, 1333, 1300, 1254, 1113, 964, 908 cm^{-1}; HRMS (ESI) Calcd for $C_{34}H_{33}N_2O_5S$ ([M+H]$^+$) 581.2105. Found 581.2101.

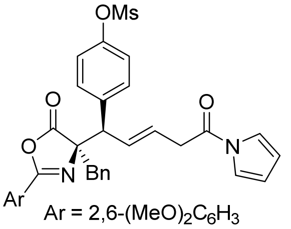

Ar = 2,6-(MeO)$_2$C$_6$H$_3$

RR-**4ae:** HPLC OX3, H/EtOH = 4:1, flow rate = 1.0 mL/min, 40 °C, λ = 254 nm, 25.6 min (minor isomer of major diastereomer), 27.8 min (major isomer of major diastereomer), 33.1 min (minor diastereomer), 36.7 min (minor diastereomer); ^{1}H NMR (400 MHz, CDCl$_3$) δ 7.42 (2H, d, J = 8.7 Hz), 7.30 (2H, brs), 7.29 (1H, t, J = 8.5 Hz), 7.24-7.18 (5H, m), 7.18 (2H, d, J = 8.7 Hz), 6.44 (2H, d, J = 8.5 Hz), 6.35 (1H, dd, J = 15.3, 10.1 Hz), 6.29 (2H, t, J = 2.1 Hz), 6.03 (1H, dt, J = 15.3, 6.8 Hz), 4.01 (1H, d, J = 10.1 Hz), 3.73 (1H, dd, J = 17.3, 6.8 Hz), 3.67 (1H, dd, J = 17.3, 6.8 Hz), 3.60 (6H, s), 3.43 (1H, d, J = 13.5 Hz), 3.17 (1H, d, J = 13.5 Hz), 3.09 (3H, s); ^{13}C NMR (101 MHz, CDCl$_3$) δ 178.9, 168.3, 159.4, 158.4, 148.6, 137.5, 134.3, 133.1, 132.1, 131.2, 130.9, 128.0, 127.1, 126.5, 121.7, 119.2, 113.5, 104.8, 103.6, 78.0, 56.0, 55.2, 42.8, 38.3, 37.3; IR (film): 3030, 2936, 2841, 1805, 1717, 1661, 1595, 1476, 1366, 1256, 1113, 964, 910 cm^{-1}; HRMS (ESI) Calcd for $C_{34}H_{33}N_2O_8S$ ([M+H]$^+$) 629.1952. Found 629.1949.

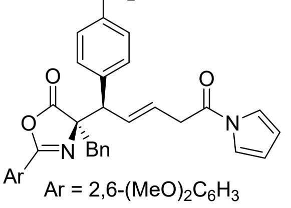

Ar = 2,6-(MeO)$_2$C$_6$H$_3$

RR-**4af:** HPLC IC3, H/EtOH = 19:1, flow rate = 1.0 mL/min, λ = 250 nm, 26.6 min (major isomer of major diastereomer), 30.7 min (minor diastereomer), 36.4 min (minor isomer of minor diastereomer), 58.5 min (minor diastereomer); ^{1}H NMR (400 MHz, CDCl$_3$) δ 7.88 (2H, d, J = 8.5 Hz), 7.41 (2H, d, J = 8.5 Hz), 7.30 (2H, brs), 7.28 (1H, t, J = 8.7 Hz), 7.24-7.16 (5H, m), 6.43 (2H, d, J = 8.7 Hz), 6.40 (1H, ddt, J = 15.5, 10.1, 1.4 Hz), 6.28 (2H, t, J = 2.5 Hz), 6.04 (1H, dt, J = 15.5, 6.8 Hz), 4.04 (1H, d, J = 10.1 Hz), 3.73 (1H, ddd, J = 17.2, 6.8, 1.4 Hz), 3.67 (1H, ddd, J = 17.2, 6.8, 1.4 Hz), 3.58 (6H, s), 3.45 (1H, d, J = 13.7 Hz), 3.19 (1H, d, J = 13.7 Hz), 1.56 (9H, s); ^{13}C NMR (101 MHz, CDCl$_3$) δ 178.8, 168.3, 165.8, 159.4, 158.3, 142.6, 134.4, 133.1, 132.1, 131.1, 130.9, 129.4$_4$, 129.4$_0$, 128.0, 127.1, 126.5, 119.2, 113.5, 104.8, 103.5, 81.0, 78.1, 55.9, 42.8, 38.4, 28.3, one carbon atom was not found probably due to overlapping; IR (film): 2974, 2839,

1807, 1707, 1659, 1595, 1476, 1294, 1256, 1165, 1113, 964, 908 cm^{-1}; HRMS (ESI) Calcd for $C_{38}H_{39}N_2O_7$ ([M+H]$^+$) 635.2752. Found 635.2749.

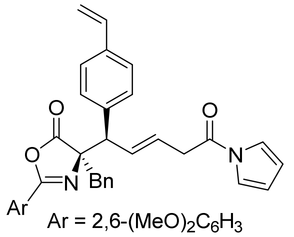

RR-**4ag:** HPLC OD3, H/EtOH = 10:1, flow rate = 1.0 mL/min, λ = 240 nm, 15.4 min (minor diastereomer), 18.7 min (major isomer of major diastereomer), 28.5 min (minor isomer of major diastereomer), 41.7 min (minor diastereomer); ^{1}H NMR (400 MHz, $CDCl_3$) *δ* 7.35-7.27 (6H, m), 7.27 (1H, t, *J* = 8.7 Hz), 7.24-7.15 (5H, m), 6.66 (1H, dd, *J* = 17.7, 11.3 Hz), 6.42 (2H, d, *J* = 8.7 Hz), 6.39 (1H, dd, *J* = 15.6, 10.1 Hz), 6.28 (2H, t, *J* = 2.5 Hz), 6.02 (1H, dt, *J* = 15.6, 6.6 Hz), 5.70 (1H, d, *J* = 17.7 Hz), 5.20 (1H, d, *J* = 11.3 Hz), 3.98 (1H, d, *J* = 10.1 Hz), 3.72 (1H, dd, *J* = 16.9, 6.6 Hz), 3.66 (1H, dd, *J* = 16.9, 6.6 Hz), 3.57 (6H, s), 3.43 (1H, d, *J* = 17.7 Hz), 3.18 (1H, d, *J* = 17.7 Hz); ^{13}C NMR (101 MHz, $CDCl_3$) *δ* 179.0, 168.4, 159.4, 158.1, 137.6, 136.6, 134.6, 133.0, 132.6, 130.9, 129.7, 128.0, 127.0, 126.2, 125.9, 119.2, 113.9, 113.4, 105.0, 103.5, 78.2, 55.9, 55.8, 42.8, 38.4, one carbon atom was not found probably due to overlapping.; IR (film): 3030, 2930, 2839, 1805, 1717, 1663, 1595, 1476, 1339, 1300, 1256, 1115, 964, 916 cm^{-1}; HRMS (ESI) Calcd for $C_{35}H_{33}N_2O_5$ ([M+H]$^+$) 561.2384. Found 561.2383.

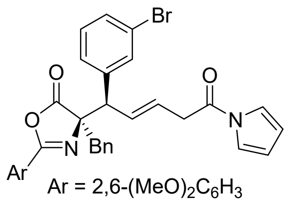

RR-**4ah:** HPLC OD3, H/IPA/EtOH = 15:1:4, flow rate = 0.5 mL/min, λ = 254 nm, 24.8 min (minor diastereomer), 28.7 min (minor isomer of major diastereomer), 32.6 min (major isomer of major diastereomer), 44.5 min (minor diastereomer); ^{1}H NMR (400 MHz, $CDCl_3$) *δ* 7.50 (1H, t, *J* = 1.7 Hz), 7.35 (1H, dt, *J* = 7.9, 1.7 Hz), 7.33-7.26 (3H, m), 7.29 (1H, t, *J* = 8.5 Hz), 7.24-7.17 (5H, m), 7.13 (1H, t, *J* = 7.9 Hz), 6.44 (2H, d, *J* = 8.5 Hz), 6.33 (1H, ddt, *J* = 15.4, 10.1, 1.4 Hz), 6.29 (2H, t, *J* = 2.5 Hz), 6.03 (1H, dt, *J* = 15.4, 6.5 Hz), 3.94 (1H, d, *J* = 10.1 Hz), 3.73 (1H, ddd, *J* = 17.2, 6.5, 1.4 Hz), 3.67 (1H, ddd, *J* = 17.2, 6.5, 1.4 Hz), 3.61 (6H, s), 3.43 (1H, d, *J* = 13.7 Hz), 3.17 (1H, d, *J* = 13.7 Hz); ^{13}C NMR (101 MHz, $CDCl_3$) *δ* 178.8, 168.3, 159.5, 158.4, 140.4, 134.3, 133.1, 132.2, 132.1, 130.9, 130.6, 129.9, 128.4, 128.0, 127.1, 126.5, 122.5, 119.2, 113.5, 104.8, 103.5, 77.9, 56.1, 55.6, 42.7, 38.4; IR (film): 2936, 2839, 1805, 1715, 1659, 1593, 1474, 1333, 1300, 1254, 1111, 964, 908 cm^{-1}; HRMS (ESI) Calcd for $C_{33}H_{30}N_2O_5{}^{79}Br$ ([M+H]$^+$) 613.1333. Found 613.1330.

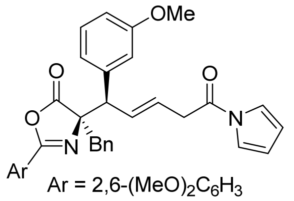

RR-**4ai:** HPLC AD3, H/IPA/EtOH = 85.9:9.1:5, flow rate = 1.0 mL/min, λ = 210 nm, 19.9 min (minor isomer of major diastereomer), 22.0 min (minor diastereomer), 25.2 min (major isomer of major diastereomer), 29.4 min (minor diastereomer); ^{1}H NMR (400 MHz, $CDCl_3$) *δ* 7.30 (2H, brs), 7.27 (1H, t, *J* = 8.6 Hz), 7.25-7.18 (5H, m), 7.18 (1H, t, *J* = 8.1 Hz), 6.96 (1H, d, *J* = 8.1 Hz), 6.91 (1H, t, *J* = 2.3 Hz), 6.77 (1H, dd, *J* = 8.1, 2.3 Hz), 6.43 (2H, d, *J* = 8.6 Hz), 6.37 (1H, ddt, *J* = 15.3, 10.1, 1.2 Hz), 6.28 (2H, t, *J* = 2.3 Hz), 6.02 (1H, dt, *J* = 15.3, 6.9 Hz), 3.96 (1H, d, *J* = 10.1 Hz), 3.72 (1H, ddd, *J* = 16.9, 6.9, 1.2 Hz), 3.70 (3H, s), 3.66 (1H, ddd, *J* = 16.9, 6.9, 1.2 Hz), 3.59 (6H, s), 3.44 (1H, d, *J* = 13.7 Hz), 3.18 (1H, d, *J* = 13.7 Hz); ^{13}C NMR (101 MHz, $CDCl_3$) *δ* 179.0, 168.5, 159.4, 159.3, 158.2, 139.5, 134.6, 133.0, 132.7, 130.9, 129.3, 128.0, 127.0, 125.9, 121.9, 119.2, 114.8, 113.4, 113.2, 105.0, 103.5, 78.1, 56.1, 55.9, 55.1, 42.9, 38.4; IR (film): 3030, 2936, 2837, 1805, 1717, 1661, 1595, 1476, 1335, 1300, 1258, 1113, 966, 918 cm^{-1}; HRMS (ESI) Calcd for $C_{34}H_{33}N_2O_6$ ([M+H]$^+$) 565.2333. Found 565.2333.

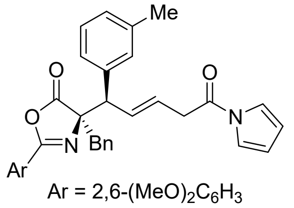

RR-**4aj:** HPLC AD3, H/IPA = 19:1, flow rate = 1.0 mL/min, λ = 210 nm, 29.1 min (minor isomer of major diastereomer), 31.8 min (minor diastereomer), 34.7 min (major isomer of major diastereomer), 55.0 min (minor diastereomer); ^{1}H NMR (400 MHz, $CDCl_3$) *δ* 7.30 (2H, brs), 7.27 (1H, t, *J* = 8.4 Hz), 7.24-7.17 (6H, m), 7.15 (1H, t, *J* = 7.5 Hz), 7.14 (1H, s), 7.02 (1H, d, *J* = 7.5 Hz), 6.43 (2H, d, *J* = 8.4 Hz), 6.36 (1H, ddt, *J* = 15.5, 9.8, 1.3 Hz), 6.27 (2H, t, *J* = 2.5 Hz), 6.00 (1H, dt, *J* = 15.5, 6.9 Hz), 3.93 (1H, d, *J* = 9.8 Hz), 3.71 (1H, ddd, *J* = 17.4, 6.9, 1.3 Hz), 3.65 (1H, ddd, *J* = 17.4, 6.9, 1.3 Hz), 3.59 (6H, s), 3.42 (1H, d, *J* = 13.7 Hz), 3.17 (1H, d, *J* = 13.7 Hz), 2.28 (3H, s); ^{13}C NMR (101 MHz, $CDCl_3$) *δ* 179.0, 168.5, 159.5, 158.0, 138.0, 137.9, 134.7, 133.0, 130.9, 130.3, 128.2_1, 128.1_5, 128.0, 126.9, 126.5, 125.6, 119.2, 113.4, 105.1, 103.5, 78.1, 56.1, 55.9, 42.9, 38.5, 21.6, one carbon atom was not found probably due to overlapping.; IR (film): 3030, 2926, 2839, 1805, 1717, 1661, 1595, 1474, 1333, 1300, 1254, 1113, 964, 910 cm^{-1}; HRMS (ESI) Calcd for $C_{34}H_{33}N_2O_5$ ([M+H]$^+$) 549.2384. Found 549.2384.

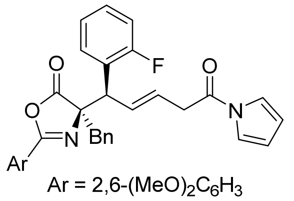

RR-**4ak:** HPLC OZ3, H/EtOH = 19:1, flow rate = 1.0 mL/min, λ = 210 nm, 20.1 min (minor diastereomer), 22.2 min (minor diastereomer), 34.0 min (minor isomer of major diastereomer), 46.2 min (major isomer of major diastereomer); ^{1}H NMR (400 MHz, $CDCl_3$) *δ* 7.62 (1H, td, *J* = 7.8 Hz, $J_{F\text{-}H}$ = 1.8 Hz), 7.29_3 (1H, t, *J* = 8.7 Hz), 7.28_6 (2H, brs), 7.24-7.16 (6H, m), 7.08-7.00 (2H, m), 6.45 (2H, d, *J* = 8.7 Hz), 6.30 (1H, ddq, $J_{H\text{-}H}$ = 15.6, 9.7, 1.3 Hz, $J_{F\text{-}H}$ = 1.3 Hz), 6.27 (2H, t, *J* = 2.3 Hz), 6.04 (1H, dt, *J* = 15.6, 6.5 Hz), 4.50 (1H, d, *J* = 9.7 Hz), 3.71 (1H, ddd, *J* = 16.8, 6.5, 1.3 Hz), 3.65 (1H, ddd, *J* = 16.8, 6.5, 1.3 Hz), 3.61 (6H, s), 3.46 (1H, d, *J* = 13.7 Hz), 3.20 (1H, d, *J* = 13.7 Hz); ^{13}C NMR (101 MHz, $CDCl_3$) *δ* 178.6, 168.3, 160.3 (d, $J_{F\text{-}C}$ = 251.6

Hz), 159.4, 158.3, 134.4, 133.0, 131.9, 131.0, 130.7 (d, $J_{F\text{-}C}$ = 3.9 Hz), 128.9 (d, $J_{F\text{-}C}$ = 8.7 Hz), 128.0, 127.0, 126.5, 125.4 (d, $J_{F\text{-}C}$ = 14.5 Hz), 123.9 (d, $J_{F\text{-}C}$ = 3.9 Hz), 119.2, 115.8 (d, $J_{F\text{-}C}$ = 24.2 Hz), 113.4, 105.0, 103.6, 77.8, 55.9, 46.9, 42.7, 38.4; ^{19}F NMR (376 MHz, $CDCl_3$) δ –115.5; IR (film): 3030, 2936, 2839, 1807, 1715, 1661, 1595, 1476, 1333, 1300, 1256, 1111, 964, 908 cm^{-1}; HRMS (ESI) Calcd for $C_{33}H_{30}N_2O_5F$ ([M+H]$^+$) 553.2133. Found 553.2135.

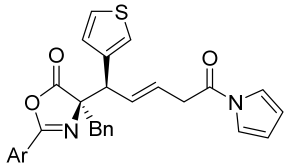

Ar = 2,6-$(MeO)_2C_6H_3$

RR-**4al:** HPLC OZ3, H/IPA = 4:1, flow rate = 1.0 mL/min, 40 °C, λ = 254 nm, 8.4 min (minor diastereomer), 9.0 min (minor isomer of major diastereomer), 11.1 min (minor diastereomer), 12.5 min (major isomer of major diastereomer); ^{1}H NMR [400 MHz, $(CD_3)_2CO$] δ 7.45 (2H, t, *J* = 2.3 Hz), 7.37 (1H, t, *J* = 8.5 Hz), 7.36 (1H, dd, *J* = 5.0, 3.0 Hz), 7.33 (1H, dd, *J* = 3.0, 1.4 Hz), 7.28-7.20 (3H, m), 7.20-7.15 (2H, m), 7.12 (1H, dd, *J* = 5.0, 1.4 Hz), 6.61 (2H, d, *J* = 8.5 Hz), 6.30 (1H, ddt, *J* = 15.4, 9.6, 1.4 Hz), 6.28 (2H, t, *J* = 2.3 Hz), 6.05 (1H, dt, *J* = 15.4, 6.4 Hz), 4.19 (1H, d, *J* = 9.6 Hz), 3.90 (1H, ddd, *J* = 16.7, 6.4, 1.4 Hz), 3.84 (1H, ddd, *J* = 16.7, 6.4, 1.4 Hz), 3.66 (6H, s), 3.41 (1H, d, *J* = 13.7 Hz), 3.13 (1H, d, *J* = 13.7 Hz; ^{13}C NMR [101 MHz, $(CD_3)_2CO$] δ 179.8, 169.6, 160.2, 158.0, 139.8, 135.8, 133.8, 133.3, 131.6, 129.4, 128.6, 127.7, 126.8, 125.4, 124.0, 120.1, 113.6, 106.2, 104.5, 78.6, 56.3, 52.1, 43.2, 38.6; IR (film): 3011, 2934, 2839, 1805, 1715, 1661, 1595, 1476, 1329, 1302, 1254, 1113, 964, 918 cm^{-1}; HRMS (ESI) Calcd for $C_{31}H_{29}N_2O_5S$ ([M+H]$^+$) 541.1792. Found 541.1792.

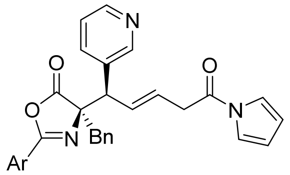

Ar = 2,6-$(MeO)_2C_6H_3$

RR-**4am:** HPLC OX3, H/IPA/EtOH = 8:1:1, flow rate = 1.0 mL/min, 40 °C, λ = 254 nm, 24.4 min (minor isomer of major diastereomer), 25.9 min (major isomer of major diastereomer), 46.0 min (minor diastereomer), 52.4 min (minor diastereomer); ^{1}H NMR (400 MHz, $CDCl_3$) δ 8.60 (1H, brs), 8.47 (1H, brd, *J* = 3.7 Hz), 7.67 (1H, d, *J* = 7.8 Hz), 7.29 (2H, brs), 7.26 (1H, t, *J* = 8.5 Hz), 7.24-7.14 (6H, m), 6.42 (2H, d, *J* = 8.5 Hz), 6.41 (1H, dd, *J* = 15.5, 9.8 Hz), 6.28 (2H, t, *J* = 2.3 Hz), 6.06 (1H, dt, *J* = 15.5, 6.7 Hz), 4.00 (1H, d, *J* = 9.8 Hz), 3.73 (1H, dd, *J* = 17.4, 6.7 Hz), 3.67 (1H, dd, *J* = 17.4, 6.7 Hz), 3.59 (6H, s), 3.45 (1H, d, *J* = 13.8 Hz), 3.19 (1H, d, *J* = 13.8 Hz); ^{13}C NMR (101 MHz, $CDCl_3$) δ 178.8, 168.1, 159.3, 158.5, 150.7, 148.6, 137.1, 134.2, 133.7, 133.0, 131.6, 130.8, 128.0, 127.1, 126.9, 123.2, 119.1, 113.5, 104.8, 103.5, 77.9, 55.9, 53.6, 42.7, 38.2; IR (film): 3030, 2930, 2841, 1805, 1713, 1661, 1593, 1470, 1327, 1302, 1256, 1113, 962, 908 cm^{-1}; HRMS (ESI) Calcd for $C_{32}H_{30}N_3O_5$ ([M+H]$^+$) 536.2180. Found 536.2175.

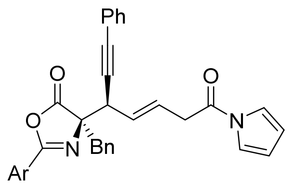

Ar = 2,6-$(MeO)_2C_6H_3$

RR-**4an:** HPLC OD3, H/EtOH = 10:1, flow rate = 1.0 mL/min, 40 °C, λ = 210 nm, 16.5 min (major isomer of major diastereomer), 18.3 min (minor diastereomer), 20.3 min (minor isomer of major diastereomer), 22.9 min (minor diastereomer); ^{1}H NMR (400 MHz, $CDCl_3$) δ 7.48-7.41 (2H, m), 7.33-7.18 (11H, m), 6.46 (2H, d, *J* = 8.4 Hz), 6.25_0 (2H, t, *J* = 2.4 Hz), 6.24_8 (1H, dt, *J* = 15.3, 7.3 Hz), 5.98 (1H, dd, *J* = 15.3, 7.1 Hz), 3.94 (1H, d, *J* = 7.1 Hz), 3.70 (1H, dd, *J* = 18.1, 7.3 Hz), 3.63 (1H, dd, *J* = 18.1, 7.3 Hz), 3.54 (6H, s), 3.41 (1H, d, *J* = 13.7 Hz), 3.37 (1H, d, *J* = 13.7 Hz); ^{13}C NMR (101 MHz, $CDCl_3$) δ 177.8, 168.1, 159.4, 158.5, 134.4, 133.0, 131.9, 130.8, 128.8, 128.3, 128.2, 128.1, 126.9_9, 126.9_5, 123.0, 119.1, 113.4, 105.4, 103.6, 86.7, 84.5, 76.7, 55.9, 42.5, 41.4, 38.3; IR (film): 3032, 2936, 2839, 2251, 1809, 1717, 1668, 1595, 1476, 1433, 1329, 1302, 1256, 1113, 964, 908 cm^{-1}; HRMS (ESI) Calcd for $C_{35}H_{31}N_2O_5$ ([M+H]$^+$) 559.2227. Found 559.2224.

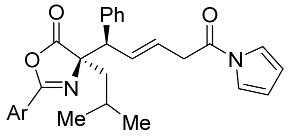

Ar = 2,6-$(MeO)_2C_6H_3$

RR-**4ba:** HPLC OD3, H/IPA = 10:1, flow rate = 1.0 mL/min, 40 °C, λ = 254 nm, 19.9 min (major isomer of major diastereomer), 26.1 min (minor diastereomer), 32.5 min (minor diastereomer), 36.6 min (minor isomer of major diastereomer); ^{1}H NMR (400 MHz, $CDCl_3$) δ 7.35 (1H, t, *J* = 8.5 Hz), 7.32-7.18 (7H, m), 6.53 (2H, d, *J* = 8.5 Hz), 6.26 (2H, t, *J* = 2.5 Hz), 6.25 (1H, dd, *J* = 15.5, 9.8 Hz), 5.90 (1H, dt, *J* = 15.5, 6.8 Hz), 3.74 (1H, d, *J* = 9.8 Hz), 3.73 (6H, s), 3.67 (1H, dd, *J* = 17.3, 6.8 Hz), 3.59 (1H, dd, *J* = 17.3, 6.8 Hz), 2.28-2.16 (1H, m), 1.83-1.62 (2H, m), 0.93 (3H, d, *J* = 6.4 Hz), 0.89 (3H, d, *J* = 6.4 Hz); ^{13}C NMR (101 MHz, $CDCl_3$) δ 180.5, 168.4, 159.4, 157.9, 138.0, 133.1, 133.0, 129.5, 128.3, 127.3, 125.6, 119.2, 113.3, 105.7, 103.8, 76.3, 56.3, 55.9, 45.6, 38.5, 24.8, 24.4, 23.0; IR (film): 2957, 2839, 1805, 1715, 1667, 1595, 1470, 1346, 1331, 1256, 1111, 957, 918 cm^{-1}; HRMS (ESI) Calcd for $C_{30}H_{33}N_2O_5$ ([M+H]$^+$) 501.2384. Found 501.2382.

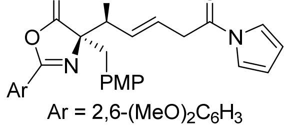

Ar = 2,6-$(MeO)_2C_6H_3$

RR-**4ca:** HPLC OZ3, H/IPA/EtOH = 18:1:1, flow rate = 1.0 mL/min, 40 °C, λ = 254 nm, 15.1 min (minor diastereomer), 17.1 min (major isomer of major diastereomer), 18.6 min (minor isomer of major diastereomer), 20.2 min (minor diastereomer); ^{1}H NMR (400 MHz, $CDCl_3$) δ 7.37 (2H, d, *J* = 7.4 Hz), 7.30 (2H, brs), 7.33-7.17 (4H, m), 7.13 (2H, d, *J* = 8.7 Hz), 6.74 (2H, d, *J* = 8.7 Hz), 6.43 (2H, d, *J* = 8.7 Hz), 6.38 (1H, dd, *J* = 15.6, 10.2 Hz), 6.27 (2H, t, *J* = 2.5 Hz), 6.01 (1H, dt, *J* = 15.6, 6.6 Hz), 3.96 (1H, d, *J* = 10.2 Hz), 3.74 (3H, s), 3.71 (1H, dd, *J* = 18.5, 6.6 Hz), 3.65 (1H, dd, *J* = 18.5, 6.6 Hz), 3.60 (6H, s), 3.38 (1H, d, *J* = 14.0 Hz), 3.12 (1H, d, *J* = 14.0 Hz); ^{13}C NMR (101 MHz, $CDCl_3$) δ 179.0, 168.4, 159.4, 158.6, 158.0, 138.1, 132.9, 132.8, 132.0, 129.5, 128.3, 127.4, 126.6, 125.7, 119.2, 113.4, 113.3, 105.1, 103.5, 78.3, 55.8, 55.2, 42.1, 38.4; IR (film): 3113, 2936, 2835, 1807, 1730, 1665, 1595, 1512, 1470, 1348, 1298, 1254, 1117, 962, 920 cm^{-1}; HRMS (ESI) Calcd for $C_{34}H_{33}N_2O_6$ ([M+H]$^+$) 565.2333. Found 565.2329.

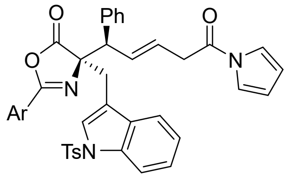

Ar = 2,6-$(MeO)_2C_6H_3$

RR-**4da:** HPLC IA3, H/IPA = 3:1, flow rate = 1.0 mL/min, 40 °C, λ = 254 nm, 9.9 min (minor isomer of major diastereomer), 10.9 min (major isomer of major diastereomer), 12.3 min (minor diastereomer), 13.9 min (minor diastereomer); ^{1}H NMR (400 MHz, $CDCl_3$) δ 7.92 (1H, d, J = 7.8 Hz), 7.57 (1H, d, J = 7.8 Hz), 7.57 (2H, d, J = 8.2 Hz), 7.51 (1H, s), 7.35 (2H, d, J = 8.0 Hz), 7.29 (2H, brs), 7.27-7.20 (5H, m), 7.18 (1H, t, J = 7.8 Hz), 6.91 (2H, d, J = 8.2 Hz), 6.39 (1H, ddt, J = 15.5, 10.2, 1.4 Hz), 6.32 (2H, d, J = 8.7 Hz), 6.28 (2H, t, J = 2.3 Hz), 6.03 (1H, dt, J = 15.5, 6.9 Hz), 4.02 (1H, d, J = 10.2 Hz), 3.69 (1H, ddd, J = 17.1, 6.9, 1.4 Hz), 3.64 (1H, ddd, J = 17.1, 6.9, 1.4 Hz), 3.55 (1H, d, J = 14.6 Hz), 3.29 (1H, d, J = 14.6 Hz), 3.21 (6H, s), 2.14 (3H, s); ^{13}C NMR (101 MHz, $CDCl_3$) δ 179.4, 168.4, 159.3, 158.5, 144.6, 137.8, 135.1, 134.8, 132.9, 132.7, 131.7, 129.7, 129.5, 128.3, 127.5, 126.7, 126.0, 125.8, 124.5, 123.3, 120.3, 119.2, 116.3, 113.5, 113.4, 104.7, 103.3, 77.8, 55.9, 55.6, 38.3, 31.7, 21.5.; IR (film): 3053, 2932, 2837, 1807, 1715, 1659, 1593, 1470, 1352, 1329, 1300, 1256, 1171, 1115, 966, 907 cm^{-1}; HRMS (ESI) Calcd for $C_{42}H_{38}N_3O_7S$ ($[M+H]^+$) 728.2425. Found 728.2418.

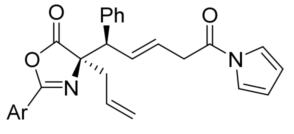

Ar = 2,6-$(MeO)_2C_6H_3$

RR-**4ea:** HPLC OD3, H/IPA = 4:1, flow rate = 1.0 mL/min, 40 °C, λ = 254 nm, 10.4 min (major isomer of major diastereomer), 13.5 min (minor diastereomer), 20.8 min (minor isomer of major diastereomer), 23.0 min (minor diastereomer); ^{1}H NMR (400 MHz, $CDCl_3$) δ 7.34 (1H, t, J = 8.6 Hz), 7.32-7.19 (7H, m), 6.52 (2H, d, J = 8.6 Hz), 6.29 (1H, ddt, J = 15.5, 10.4, 1.3 Hz), 6.27 (2H, d, J = 2.4 Hz), 5.95 (1H, dt, J = 15.5, 6.7 Hz), 5.80 (1H, dtd, J = 16.6, 9.7, 6.3 Hz), 5.20 (1H, d, J = 16.6 Hz), 5.14 (1H, d, J = 9.7 Hz), 3.86 (1H, d, J = 10.4 Hz), 3.71 (6H, s), 3.67 (1H, ddd, J = 16.8, 6.7, 1.3 Hz), 3.61 (1H, ddd, J = 16.8, 6.7, 1.3 Hz), 2.88 (1H, dd, J = 13.8, 6.3 Hz), 2.61 (1H, dd, J = 13.8, 9.7 Hz); ^{13}C NMR (101 MHz, $CDCl_3$) δ 179.0, 168.4, 159.3, 158.2, 138.1, 133.0, 132.7, 131.2, 129.4, 128.3, 127.3, 125.6, 120.1, 119.2, 113.4, 105.8, 103.8, 55.9, 54.8, 41.1, 38.4, one carbon atom was not found probably due to overlapping.; IR (film): 2940, 2907, 1807, 1715, 1668, 1593, 1476, 1346, 1327, 1256, 1113, 962, 918 cm^{-1}; HRMS (ESI) Calcd for $C_{29}H_{29}N_2O_5$ ($[M+H]^+$) 485.2071. Found 485.2063.

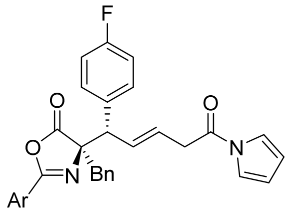

Ar = 2,6-$(MeO)_2C_6H_3$

RS-**4ab:** HPLC OZ3, H/IPA = 10:1, flow rate = 1.0 mL/min, λ = 254 nm, 12.6 min (minor isomer of major diastereomer), 14.1 min (major isomer of major diastereomer), 16.2 min (minor diastereomer), 54.2 min (minor diastereomer); ^{1}H NMR (400 MHz, $CDCl_3$) δ 7.45 (2H, dd, $J_{H\text{-}H}$ = 8.7 Hz, $J_{F\text{-}H}$ = 5.5 Hz), 7.32 (1H, t, J = 8.7 Hz), 7.23-7.16 (3H, m), 7.15-7.08 (2H, m), 7.04 (2H, t, $J_{H\text{-}H}$ = $J_{F\text{-}H}$ = 8.7 Hz), 6.48 (2H, d, J = 8.7 Hz), 6.20 (2H, t, J = 2.3 Hz), 6.19 (1H, dd, J = 15.5, 10.1 Hz), 5.90 (1H, dt, J = 15.5, 7.1 Hz), 3.88 (1H, d, J = 10.1 Hz), 3.62_9 (6H, s), 3.62_6 (1H, dd, J = 16.8, 7.1 Hz), 3.52 (1H, dd, J = 16.8, 7.1 Hz), 3.13 (1H, d, J = 13.5 Hz), 2.89 (1H, d, J = 13.5 Hz); ^{13}C NMR (101 MHz, $CDCl_3$) δ 179.8, 168.2, 162.3 (d, $J_{F\text{-}C}$ = 249.7 Hz), 159.3, 157.6, 134.4, 134.3 (d, $J_{F\text{-}C}$ = 2.9 Hz), 133.0, 132.4, 131.0 (d, $J_{F\text{-}C}$ = 7.7 Hz), 130.7, 128.1, 126.9, 125.7, 119.2, 115.6 (d, $J_{F\text{-}C}$ = 21.3 Hz), 113.4, 105.4, 103.7, 78.4, 56.0, 55.6, 41.7, 38.7; ^{19}F NMR (376 MHz, $CDCl_3$) δ –115.2; IR (film): 3148, 2938, 2841, 1805, 1717, 1670, 1595, 1508, 1476, 1337, 1302, 1258, 1223, 1113, 966 cm^{-1}; HRMS (ESI) Calcd for $C_{33}H_{30}N_2O_5F$ ($[M+H]^+$) 553.2133. Found 553.2131.

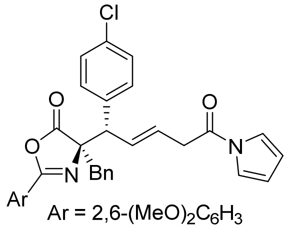

Ar = 2,6-$(MeO)_2C_6H_3$

RS-**4ac:** The absolute configuration of *RS*-**4ac** was determined by X-ray crystallographic analysis after derivertization (see page S111 and Supplymentary Figure 102). HPLC OD3, H/IPA = 10:1, flow rate = 1.0 mL/min, 40 °C, λ = 254 nm, 20.7 min (minor isomer of major diastereomer), 23.1 min (minor diastereomer), 36.7 min (minor diastereomer), 41.5 min (major isomer of major diastereomer); ^{1}H NMR (400 MHz, $CDCl_3$) δ 7.41 (2H, d, J = 8.5 Hz), 7.31_{51} (1H, t, J = 8.7 Hz), 7.31_{50} (1H, t, J = 8.5 Hz), 7.23 (2H, brs), 7.23-7.16 (3H, m), 7.15-7.08 (2H, m), 6.47 (2H, d, J = 8.7 Hz), 6.20 (2H, t, J = 2.3 Hz), 6.19 (1H, ddt, J = 15.6, 9.8, 1.4 Hz), 5.91 (1H, dt, J = 15.6, 6.8 Hz), 3.87 (1H, d, J = 9.8 Hz), 3.63 (1H, ddd, J = 16.9, 6.8, 1.4 Hz), 3.62 (6H, s), 3.52 (1H, ddd, J = 16.9, 6.8, 1.4 Hz), 3.14 (1H, d, J = 13.5 Hz), 2.91 (1H, d, J = 13.5 Hz); ^{13}C NMR (101 MHz, $CDCl_3$) δ 179.7, 168.1, 159.3, 157.6, 137.1, 134.3, 133.3, 133.0, 132.1, 130.8, 128.9, 128.1, 126.9, 125.9, 119.2, 113.4, 105.3, 103.7, 78.2, 56.0, 55.7, 41.7, 38.7; IR (film): 3030, 2936, 2839, 1805, 1717, 1668, 1595, 1476, 1335, 1300, 1256, 1113, 964, 908 cm^{-1}; HRMS (ESI) Calcd for $C_{33}H_{30}N_2O_5Cl$ ($[M+H]^+$) 569.1838. Found 569.1838.

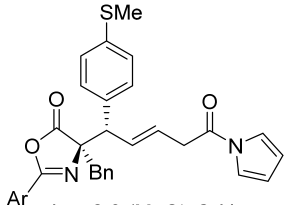

Ar = 2,6-$(MeO)_2C_6H_3$

RS-**4ad:** HPLC OX3, H/IPA/EtOH = 18:1:1, flow rate = 1.0 mL/min, λ = 254 nm, 30.4 min (minor isomer of major diastereomer), 33.6 min (minor diastereomer), 36.1 min (major isomer of major diastereomer), 45.8 min (minor diastereomer); ^{1}H NMR [400 MHz, $(CD_3)_2CO$] δ 7.56 (2H, d, J = 8.5 Hz), 7.42 (1H, t, J = 8.5 Hz), 7.36 (2H, t, J = 2.3 Hz), 7.28 (2H, d, J = 8.5 Hz), 7.25-7.16 (3H, m), 7.12-7.02 (2H, m), 6.68 (2H, d, J = 8.5 Hz), 6.20 (2H, t, J = 2.3 Hz), 6.17 (1H, ddt, J = 15.5, 10.1, 1.4 Hz), 5.88 (1H, dt, J = 15.5, 6.7 Hz), 3.91 (1H, d, J = 10.1 Hz), 3.77 (1H, ddd, J = 17.5, 6.7, 1.4 Hz), 3.73 (6H, s), 3.68 (1H, ddd, J = 17.5, 6.7, 1.4 Hz), 3.09 (1H, d, J = 13.7 Hz), 2.84 (1H, d, J = 13.7 Hz), 2.49 (3H, s); ^{13}C NMR [101 MHz, $(CD_3)_2CO$] δ 180.2, 169.2, 160.1, 157.7, 138.3, 136.7, 135.6, 133.7, 133.3, 131.3, 131.0, 128.6, 127.5, 126.9, 126.4, 119.9, 113.5, 106.7, 104.6, 79.0, 56.7, 56.4, 42.0, 38.7, 15.3; IR (film): 3030, 2922, 2839, 1805, 1715, 1670, 1595,

1476, 1337, 1300, 1256, 1113, 964, 908 cm^{-1}; HRMS (ESI) Calcd for $C_{34}H_{33}N_2O_5S$ ([M+H]$^+$) 581.2105. Found 581.2101.

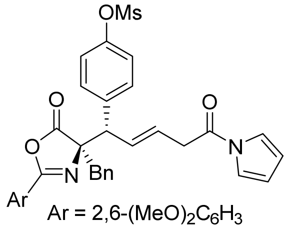

RS-**4ae:** HPLC OX3, H/EtOH = 4:1, flow rate = 1.0 mL/min, 40 °C, λ = 254 nm, 25.6 min (minor diastereomer), 27.9 min (minor diastereomer), 33.0 min (minor isomer of major diastereomer), 36.6 min (major isomer of major diastereomer); ^{1}H NMR (400 MHz, $CDCl_3$) δ 7.55 (2H, d, J = 8.7 Hz), 7.33 (1H, t, J = 8.5 Hz), 7.28 (2H, d, J = 8.7 Hz), 7.23-7.15 (3H, m), 7.22 (2H, brs), 7.14-7.07 (2H, m), 6.49 (2H, d, J = 8.5 Hz), 6.19 (2H, t, J = 2.5 Hz), 6.15 (1H, ddt, J = 15.5, 9.9, 1.2 Hz), 5.92 (1H, dt, J = 15.5, 6.7 Hz), 3.91 (1H, d, J = 9.9 Hz), 3.64 (6H, s), 3.62 (1H, ddd, J = 16.9, 6.7, 1.2 Hz), 3.51 (1H, ddd, J = 16.9, 6.7, 1.2 Hz), 3.13 (1H, d, J = 13.7 Hz), 3.12 (3H, s), 2.88 (1H, d, J = 13.7 Hz); ^{13}C NMR (101 MHz, $CDCl_3$) δ 179.5, 168.1, 159.3, 157.8, 148.6, 138.0, 134.2, 133.1, 132.0, 131.1, 130.7, 128.1, 127.0, 126.1, 122.2, 119.1, 113.4, 105.3, 103.7, 78.2, 56.0, 55.7, 41.6, 38.6, 37.4; IR (film): 3032, 2938, 2841, 1805, 1715, 1668, 1595, 1476, 1366, 1256, 1113, 964, 908 cm^{-1}; HRMS (ESI) Calcd for $C_{34}H_{33}N_2O_8S$ ([M+H]$^+$) 629.1952. Found 629.1947.

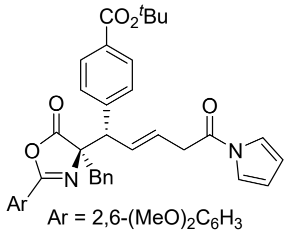

RS-**4af:** HPLC IC3, H/EtOH = 19:1, flow rate = 1.0 mL/min, λ = 250 nm, 27.2 min (minor diastereomer), 30.6 min (major isomer of major diastereomer), 36.6 min (minor diastereomer), 58.7 min (minor isomer of major diastereomer); ^{1}H NMR (400 MHz, $CDCl_3$) δ 7.97 (2H, d, J = 8.7 Hz), 7.54 (2H, d, J = 8.7 Hz), 7.31 (1H, t, J = 8.5 Hz), 7.23 (2H, brs), 7.22-7.15 (3H, m), 7.15-7.06 (2H, m), 6.47 (2H, d, J = 8.5 Hz), 6.22 (1H, ddt, J = 15.6, 9.6, 1.3 Hz), 6.19 (2H, d, J = 2.3 Hz), 5.92 (1H, dt, J = 15.6, 6.7 Hz), 3.95 (1H, d, J = 9.6 Hz), 3.62 (1H, ddd, J = 16.9, 6.7, 1.3 Hz), 3.61 (6H, s), 3.52 (1H, ddd, J = 16.9, 6.7, 1.3 Hz), 3.15 (1H, d, J = 13.5 Hz), 2.90 (1H, d, J = 13.5 Hz), 1.58 (9H, s); ^{13}C NMR (101 MHz, $CDCl_3$) δ 179.7, 168.0, 165.7, 159.3, 157.5, 143.3, 134.3, 133.0, 132.0, 131.2, 130.7, 129.9, 129.3, 128.1, 126.9, 126.1, 119.1, 113.4, 105.4, 103.7, 81.2, 78.2, 56.3, 55.9, 41.7, 38.6, 28.3; IR (film): 2974, 2839, 1807, 1709, 1670, 1595, 1476, 1294, 1256, 1111, 964, 908 cm^{-1}; HRMS (ESI) Calcd for $C_{38}H_{39}N_2O_7$ ([M+H]$^+$) 635.2752. Found 635.2749.

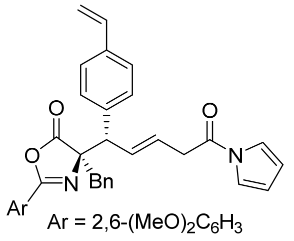

RS-**4ag:** HPLC OD3, H/EtOH = 10:1, flow rate = 1.0 mL/min, λ = 240 nm, 15.3 min (minor isomer of major diastereomer), 18.6 min (minor diastereomer), 28.3 min (minor diastereomer), 40.5 min (major isomer of major diastereomer); ^{1}H NMR (400 MHz, $CDCl_3$) δ 7.44 (2H, d, J = 8.5 Hz), 7.39 (2H, d, J = 8.5 Hz), 7.30 (1H, t, J = 8.5 Hz), 7.22 (2H, brs), 7.22-7.15 (3H, m), 7.15-7.07 (2H, m), 6.70 (1H, dd, J = 17.9, 11.1 Hz), 6.46 (2H, d, J = 8.5 Hz), 6.21 (1H, ddt, J = 15.5, 9.8, 1.2 Hz), 6.18 (2H, t, J = 2.5 Hz), 5.90 (1H, dt, J = 15.5, 6.8 Hz), 5.74 (1H, d, J = 17.9 Hz), 5.23 (1H, d, J = 11.1 Hz), 3.88 (1H, d, J = 9.8 Hz), 3.61 (1H, ddd, J = 16.7, 6.8, 1.2 Hz), 3.60 (6H, s), 3.50 (1H, ddd, J = 16.7, 6.8, 1.2 Hz), 3.14 (1H, d, J = 13.5 Hz), 2.92 (1H, d, J = 13.5 Hz); ^{13}C NMR (101 MHz, $CDCl_3$) δ 179.9, 168.1, 159.3, 157.4, 138.1, 136.7, 136.5, 134.5, 132.9, 132.5, 130.7, 129.5, 128.0, 126.8, 126.6, 125.5, 119.1, 113.9, 113.3, 105.5, 103.6, 78.4, 56.2, 55.9, 41.6, 38.7; IR (film): 3032, 2936, 2839, 1805, 1717, 1670, 1595, 1476, 1339, 1300, 1256, 1113, 964, 907cm^{-1}; HRMS (ESI) Calcd for $C_{35}H_{33}N_2O_5$ ([M+H]$^+$) 561.2384. Found 561.2383.

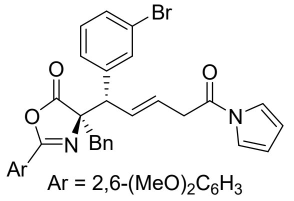

RS-**4ah:** HPLC OD3, H/IPA/EtOH = 15:1:4, flow rate = 0.5 mL/min, λ = 254 nm, 24.3 min (minor isomer of major diastereomer), 28.3 min (minor diastereomer), 32.1 min (minor diastereomer), 43.2 min (major isomer of major diastereomer); ^{1}H NMR (400 MHz, $CDCl_3$) δ 7.63 (1H, t, J = 1.8 Hz), 7.43 (1H, d, J = 8.0 Hz), 7.41 (1H, d, J = 8.0 Hz), 7.32 (1H, t, J = 8.7 Hz), 7.26-7.15 (6H, m), 7.15-7.07 (2H, m), 6.48 (2H, d, J = 8.7 Hz), 6.19 (2H, t, J = 2.5 Hz), 6.15 (1H, ddt, J = 15.5, 10.1, 1.1 Hz), 5.91 (1H, dt, J = 15.5, 6.6 Hz), 3.84 (1H, d, J = 10.1 Hz), 3.64 (6H, s), 3.61 (1H, ddd, J = 16.9, 6.6, 1.1 Hz), 3.51 (1H, ddd, J = 16.9, 6.6, 1.1 Hz), 3.13 (1H, d, J = 13.5 Hz), 2.91 (1H, t, J = 13.5 Hz); ^{13}C NMR (101 MHz, $CDCl_3$) δ 179.6, 168.1, 159.4, 157.6, 140.9, 134.3, 133.0, 132.5, 131.9, 130.7, 130.6, 130.3, 128.1, 128.0, 126.9, 126.1, 122.7, 119.1, 113.4, 105.4, 103.7, 78.1, 56.0, 41.6, 38.6, one carbon was not found probably due to overlapping.; IR (film): 3030, 2938, 2839, 1805, 1717, 1670, 1593, 1476, 1335, 1294, 1256, 1113, 964, 908 cm^{-1}; HRMS (ESI) Calcd for $C_{33}H_{30}N_2O_5{}^{79}Br$ ([M+H]$^+$) 613.1333. Found 613.1331.

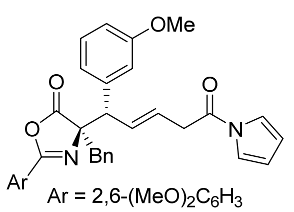

RS-**4ai:** HPLC AD3, H/IPA/EtOH = 85.9:9.1:5, flow rate = 1.0 mL/min, λ = 210 nm, 19.7 min (minor diastereomer), 21.8 min (major isomer of major diastereomer), 24.9 min (minor diastereomer), 29.0 min (minor isomer of major diastereomer); ^{1}H NMR (400 MHz, $CDCl_3$) δ 7.31 (1H, t, J = 8.4 Hz), 7.27 (1H, t, J = 7.9 Hz), 7.22 (2H, brs), 7.21-7.15 (3H, m), 7.15-7.10 (2H, m), 7.10 (1H, d, J = 7.9 Hz), 7.03 (1H, t, J = 2.8 Hz), 6.82 (1H, dd, J = 7.9, 2.8 Hz), 6.47 (2H, d, J = 8.4 Hz), 6.20 (1H, ddt, J = 15.6, 10.1, 1.4 Hz), 6.17 (2H, t, J = 2.5 Hz), 5.90 (1H, dt, J = 15.6, 6.7 Hz), 3.85 (1H, d, J = 10.1 Hz), 3.80 (3H, s), 3.62 (6H, s), 3.60 (1H, ddd, J = 16.8, 6.7, 1.4 Hz), 3.50 (1H, ddd, J = 16.8, 6.7, 1.4 Hz), 3.14 (1H, d, J = 13.5 Hz), 2.94 (1H, d, J = 13.5 Hz); ^{13}C NMR (101 MHz, $CDCl_3$) δ 180.0, 168.1, 159.7, 159.4, 157.3, 140.1, 134.6, 132.9, 132.6, 130.8, 129.7, 128.0, 126.8,

125.4, 121.6, 119.2, 115.4, 113.3, 112.8, 105.7, 103.7, 78.4, 56.6, 56.0, 55.3, 41.7, 38.8; IR (film): 2936, 2837, 1805, 1715, 1667, 1593, 1470, 1335, 1298, 1254, 1111, 966, 905 cm^{-1}; HRMS (ESI) Calcd for $C_{34}H_{33}N_2O_6$ ($[M+H]^+$) 565.2333. Found 565.2337.

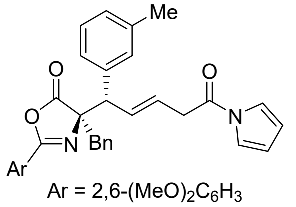

RS-**4aj:** HPLC AD3, H/IPA = 19:1, flow rate = 1.0 mL/min, λ = 210 nm, 29.0 min (minor diastereomer), 31.5 min (major isomer of major diastereomer), 34.7 min (minor diastereomer), 54.9 min (minor isomer of major diastereomer); ^{1}H NMR (400 MHz, $CDCl_3$) *δ* 7.32 (1H, d, *J* = 7.8 Hz), 7.31 (1H, t, *J* = 8.6 Hz), 7.28-7.21 (2H, m), 7.22 (2H, brs), 7.21-7.15 (3H, m), 7.15-7.09 (2H, m), 7.09 (1H, d, *J* = 7.8 Hz), 6.48 (2H, d, *J* = 8.6 Hz), 6.19 (1H, ddt, *J* = 15.5, 10.1, 1.4 Hz), 6.17 (2H, t, *J* = 2.3 Hz), 5.89 (1H, dt, *J* = 15.5, 6.7 Hz), 3.83 (1H, d, *J* = 10.1 Hz), 3.63 (6H, s), 3.59 (1H, ddd, *J* = 16.6, 6.7, 1.4 Hz), 3.49 (1H, ddd, *J* = 16.6, 6.7, 1.4 Hz), 3.12 (1H, d, *J* = 13.5 Hz), 2.91 (1H, d, *J* = 13.5 Hz), 2.36 (3H, s); ^{13}C NMR (101 MHz, $CDCl_3$) *δ* 180.0, 168.2, 159.4, 157.3, 138.4, 138.2, 134.6, 132.9, 130.8, 130.3, 128.7, 128.3, 128.0, 126.8, 126.3, 125.2, 119.2, 113.3, 105.7, 103.7, 78.5, 56.6, 56.0, 41.6, 38.8, 21.7, one carbon atom was not found probably due to overlapping.; IR (film): 3030, 2936, 2839, 1805, 1717, 1670, 1595, 1476, 1335, 1298, 1256, 1113, 964, 910 cm^{-1}; HRMS (ESI) Calcd for $C_{34}H_{33}N_2O_5$ ($[M+H]^+$) 549.2384. Found 549.2384.

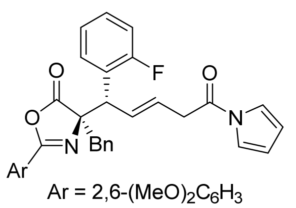

RS-**4ak:** HPLC OZ3, H/EtOH = 19:1, flow rate = 1.0 mL/min, λ = 210 nm, 20.7 min (minor isomer of major diastereomer), 22.8 min (major isomer of major diastereomer), 35.4 min (minor diastereomer), 49.0 min (minor diastereomer); ^{1}H NMR (400 MHz, $CDCl_3$) *δ* 7.69 (1H, td, *J* = 7.6, 1.7 Hz), 7.32 (1H, t, *J* = 8.5 Hz), 7.30-7.14 (7H, m), 7.14-7.06 (3H, m), 6.48 (2H, d, *J* = 8.5 Hz), 6.17 (2H, t, *J* = 2.3 Hz), 6.14 (1H, dd, *J* = 15.3, 9.8 Hz), 5.96 (1H, dt, *J* = 15.3, 6.7 Hz), 4.44 (1H, d, *J* = 9.8 Hz), 3.64 (6H, s), 3.60 (1H, ddd, *J* = 16.9, 6.7, 1.2 Hz), 3.49 (1H, ddd, *J* = 16.9, 6.7, 1.2 Hz), 3.23 (1H, d, *J* = 13.6 Hz), 2.89 (1H, d, *J* = 13.6 Hz); ^{13}C NMR (101 MHz, $CDCl_3$) *δ* 179.5, 168.1, 160.7 (d, $J_{F\text{-}C}$ = 249.7 Hz), 159.3, 157.7, 134.3, 133.0, 131.5, 130.8, 130.6 (d, $J_{F\text{-}C}$ = 2.9 Hz), 128.9 (d, $J_{F\text{-}C}$ = 8.7 Hz), 128.0, 126.8, 126.4, 125.5 (d, $J_{F\text{-}C}$ = 14.5 Hz), 124.7 (d, $J_{F\text{-}C}$ = 3.9 Hz), 119.2, 115.6 (d, $J_{F\text{-}C}$ = 23.2 Hz), 113.4, 105.5, 103.7, 78.6, 56.0, 47.0, 40.9, 38.7; ^{19}F NMR (376 MHz, $CDCl_3$) *δ* –117.0; IR (film): 3032, 2938, 2839, 1807, 1717, 1670, 1595, 1476, 1339, 1300, 1258, 1113, 966, 910 cm^{-1}; HRMS (ESI) Calcd for $C_{33}H_{30}N_2O_5F$ ($[M+H]^+$) 553.2133. Found 553.2135.

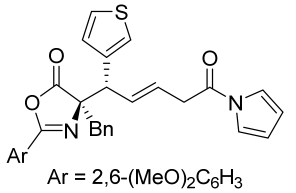

RS-**4al:** HPLC OZ3, H/IPA = 4:1, flow rate = 1.0 mL/min, 40 °C, λ = 254 nm, 8.4 min (minor isomer of major diastereomer), 9.0 min (minor diastereomer), 11.0 min (major isomer of major diastereomer), 12.5 min (minor diastereomer); ^{1}H NMR (400 MHz, $CDCl_3$) *δ* 7.31 (1H, t, *J* = 8.7 Hz), 7.32-7.27 (2H, m), 7.27-7.21 (3H, m), 7.21-7.16 (3H, m), 7.16-7.08 (2H, m), 6.48 (2H, d, *J* = 8.7 Hz), 6.19 (2H, t, *J* = 2.3 Hz), 6.14 (1H, ddt, *J* = 15.6, 9.8, 1.2 Hz), 5.89 (1H, dt, *J* = 15.6, 6.6 Hz), 4.06 (1H, d, *J* = 9.8 Hz), 3.63 (6H, s), 3.61 (1H, ddd, *J* = 16.8, 6.6, 1.2 Hz), 3.51 (1H, ddd, *J* = 16.8, 6.6, 1.2 Hz), 3.15 (1H, d, *J* = 13.8 Hz), 2.97 (1H, d, *J* = 13.8 Hz); ^{13}C NMR (101 MHz, $CDCl_3$) *δ* 179.8, 168.2, 159.4, 157.5, 138.5, 134.5, 132.9, 132.3, 130.8, 128.3, 128.0, 126.8, 125.7, 125.3, 123.3, 119.2, 113.4, 105.5, 103.7, 78.3, 56.0, 51.7, 41.6, 38.7; IR (film): 3030, 2936, 2839, 1805, 1717, 1670, 1595, 1476, 1331, 1304, 1258, 1113, 966, 910 cm^{-1}; HRMS (ESI) Calcd for $C_{31}H_{29}N_2O_5S$ ($[M+H]^+$) 541.1792. Found 541.1791.

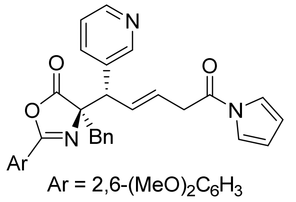

RS-**4am:** HPLC OX3, H/IPA/EtOH = 8:1:1, flow rate = 1.0 mL/min, 40 °C, λ = 254 nm, 24.4 min (minor diastereomer), 26.0 min (minor diastereomer), 45.7 min (major isomer of major diastereomer), 52.4 min (minor isomer of major diastereomer); ^{1}H NMR (400 MHz, $CDCl_3$) *δ* 8.67 (1H, brd, *J* = 1.8 Hz), 8.54 (1H, dd, *J* = 4.3, 1.8 Hz), 7.86 (1H, dt, *J* = 8.3, 1.8 Hz), 7.31 (1H, t, *J* = 8.7 Hz), 7.28 (1H, dd, *J* = 8.3, 4.3 Hz), 7.23 (2H, brs), 7.25-7.15 (3H, m), 7.15-7.05 (2H, m), 6.47 (2H, d, *J* = 8.7 Hz), 6.20_4 (1H, dd, *J* = 15.3, 10.1 Hz), 6.19_7 (2H, t, *J* = 1.8 Hz), 5.95 (1H, dt, *J* = 15.3, 6.8 Hz), 3.93 (1H, d, *J* = 10.1 Hz), 3.63 (1H, dd, *J* = 16.9, 6.8 Hz), 3.62 (6H, s), 3.53 (1H, dd, *J* = 16.9, 6.8 Hz), 3.17 (1H, d, *J* = 13.5 Hz), 2.90 (1H, d, *J* = 13.5 Hz); ^{13}C NMR (101 MHz, $CDCl_3$) *δ* 179.3, 167.9, 159.3, 157.8, 150.8, 148.9, 136.5, 134.3, 134.0, 133.0, 131.5, 130.7, 128.1, 126.9, 126.6, 123.7, 119.1, 113.4, 105.2, 103.6, 77.9, 56.0, 53.6, 41.7, 38.5; IR (film): 3028, 2839, 1805, 1713, 1667, 1591, 1470, 1329, 1302, 1256, 1109, 962, 905 cm^{-1}; HRMS (ESI) Calcd for $C_{32}H_{30}N_3O_5$ ($[M+H]^+$) 536.2180. Found 536.2174.

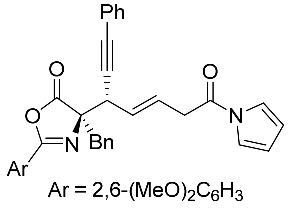

RS-**4an:** HPLC OD3, H/EtOH = 10:1, flow rate = 1.0 mL/min, 40 °C, λ = 210 nm, 16.5 min (minor diastereomer), 18.3 min (minor isomer of major diastereomer), 20.2 min (minor diastereomer), 22.9 min (major isomer of major diastereomer); ^{1}H NMR (400 MHz, $CDCl_3$) *δ* 7.48-7.41 (2H, m), 7.34-7.19 (11H, m), 6.46 (2H, d, *J* = 8.6 Hz), 6.25 (2H, t, *J* = 2.3 Hz), 6.10 (1H, dt, *J* = 15.4, 6.2 Hz), 5.96 (1H, dd, *J* = 15.4, 8.3 Hz), 3.92 (1H, d, *J* = 8.3 Hz), 3.69 (1H, dd, *J* = 16.8, 6.2 Hz), 3.63 (1H, dd, *J* = 16.8, 6.2 Hz), 3.56 (6H, s), 3.49 (1H, d, *J* = 13.5 Hz), 3.32 (1H, d, *J* = 13.5 Hz); ^{13}C NMR (101 MHz, $CDCl_3$) *δ* 178.5, 168.1, 159.4, 158.2,

134.3, 133.0, 132.0, 130.9, 129.0, 128.3, 128.2, 128.1, 127.0, 126.8, 123.1, 119.2, 113.4, 105.5, 103.7, 86.3, 85.0, 77.0, 55.9, 43.3, 41.3, 38.4; IR (film): 3032, 2938, 2839, 2247, 1807, 1717, 1668, 1595, 1476, 1456, 1433, 1331, 1301, 1256, 1113, 966, 910 cm^{-1}; HRMS (ESI) Calcd for $C_{35}H_{31}N_2O_5$ ([M+H]$^+$) 559.2233. Found 559.2227.

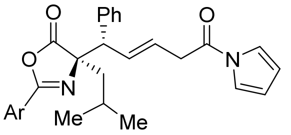

Ar = 2,6-(MeO)$_2$C$_6$H$_3$

RS-**4ba:** HPLC OD3, H/IPA = 10:1, flow rate = 1.0 mL/min, 40 °C, λ = 254 nm, 20.0 min (minor diastereomer), 25.5 min (major isomer of major diastereomer), 32.6 min (minor isomer of major diastereomer), 36.7 min (minor diastereomer); ^{1}H NMR (400 MHz, CDCl$_3$) δ 7.38_1 (1H, t, J = 8.5 Hz), 7.37_9 (2H, d, J = 7.4 Hz), 7.30 (2H, t, J = 7.4 Hz), 7.24 (1H, t, J = 7.4 Hz), 7.24 (2H, brs), 6.58 (2H, d, J = 8.5 Hz), 6.20 (2H, t, J = 2.3 Hz), 6.15 (1H, ddt, J = 15.6, 10.1, 1.3 Hz), 5.85 (1H, dt, J = 15.6, 6.6 Hz), 3.79 (6H, s), 3.67 (1H, d, J = 10.1 Hz), 3.61 (1H, ddd, J = 16.7, 6.6, 1.3 Hz), 3.50 (1H, ddd, J = 16.7, 6.6, 1.3 Hz), 1.79-1.58 (3H, m), 0.87 (3H, d, J = 6.2 Hz), 0.80 (3H, d, J = 6.8 Hz); ^{13}C NMR (101 MHz, CDCl$_3$) δ 181.3, 168.2, 159.4, 157.3, 138.7, 133.0, 132.5, 129.4, 128.6, 127.3, 125.2, 119.2, 113.3, 106.1, 103.9, 76.7, 57.2, 56.0, 44.5, 38.7, 24.6, 24.4, 22.8; IR (film): 3148, 2951, 2866, 1805, 1713, 1680, 1593, 1454, 1323, 1302, 1254, 1107, 955, 910 cm^{-1}; HRMS (ESI) Calcd for $C_{30}H_{33}N_2O_5$ ([M+H]$^+$) 501.2384. Found 501.2377.

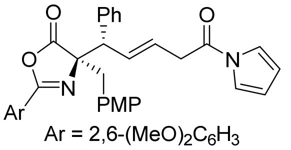

Ar = 2,6-(MeO)$_2$C$_6$H$_3$

RS-**4ca:** HPLC OZ3, H/IPA/EtOH = 18:1:1, flow rate = 1.0 mL/min, 40 °C, λ = 254 nm, 15.1 min (minor isomer of major diastereomer), 17.2 min (minor diastereomer), 18.6 min (minor diastereomer), 20.1 min (major isomer of major diastereomer); ^{1}H NMR (400 MHz, CDCl$_3$) δ 7.48 (2H, d, J = 7.7 Hz), 7.35 (2H, t, J = 7.7 Hz), 7.31 (1H, t, J = 8.5 Hz), 7.27 (1H, t, J = 7.7 Hz), 7.22 (2H, brs), 7.02 (2H, d, J = 8.7 Hz), 6.71 (2H, d, J = 8.7 Hz), 6.48 (2H, d, J = 8.5 Hz), 6.19 (1H, ddt, J = 15.6, 9.8, 1.2 Hz), 6.17 (2H, t, J = 2.3 Hz), 5.89 (1H, dt, J = 15.6, 6.7 Hz), 3.87 (1H, d, J = 9.8 Hz), 3.73 (3H, s), 3.63 (6H, s), 3.59 (1H, ddd, J = 16.6, 6.7, 1.2 Hz), 3.49 (1H, ddd, J = 16.6, 6.7, 1.2 Hz), 3.07 (1H, d, J = 13.9 Hz), 2.83 (1H, d, J = 13.9 Hz); ^{13}C NMR (101 MHz, CDCl$_3$) δ 180.0, 168.1, 159.3, 158.5, 157.3, 138.5, 132.9, 132.8, 131.7, 129.4, 128.7, 127.4, 126.5, 125.3, 119.1, 113.3_1, 113.2_6, 105.5, 103.6, 78.6, 56.4, 55.9, 55.1, 40.8, 38.7; IR (film): 3022, 2934, 2835, 1807, 1715, 1667, 1593, 1512, 1470, 1335, 1296, 1248, 1111, 964, 903 cm^{-1}; HRMS (ESI) Calcd for $C_{34}H_{33}N_2O_6$ ([M+H]$^+$) 565.2333. Found 565.2330.

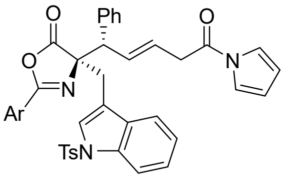

Ar = 2,6-(MeO)$_2$C$_6$H$_3$

RS-**4da:** HPLC IA3, H/IPA = 3:1, flow rate = 1.0 mL/min, 40 °C, λ = 254 nm, 9.9 min (minor diastereomer), 10.9 min (minor diastereomer), 12.3 min (major isomer of major diastereomer), 13.9 min (minor isomer of major diastereomer); ^{1}H NMR (400 MHz, CDCl$_3$) δ 7.90 (1H, d, J = 7.4 Hz), 7.60 (2H, d, J = 8.5 Hz), 7.50 (2H, d, J = 8.0 Hz), 7.43 (1H, s), 7.42 (1H, d, J = 7.4 Hz), 7.36 (2H, t, J = 8.0 Hz), 7.28_1 (1H, t, J = 7.4 Hz), 7.27_8 (1H, t, J = 8.7 Hz), 7.23 (2H, brs), 7.20 (1H, t, J = 8.0 Hz), 7.11 (1H, t, J = 7.4 Hz), 7.02 (2H, d, J = 8.5 Hz), 6.40 (2H, d, J = 8.7 Hz), 6.23 (1H, ddt, J = 15.3, 10.1, 1.0 Hz), 6.19 (2H, t, J = 2.3 Hz), 5.92 (1H, dt, J = 15.3, 6.8 Hz), 3.93 (1H, d, J = 10.1 Hz), 3.62 (1H, ddd, J = 16.9, 6.8, 1.0 Hz), 3.52 (1H, ddd, J = 16.9, 6.8, 1.0 Hz), 3.37 (6H, s), 3.24 (1H, d, J = 14.6 Hz), 2.97 (1H, d, J = 14.6 Hz), 2.20 (3H, s); ^{13}C NMR (101 MHz, CDCl$_3$) δ 180.0, 168.1, 159.2, 158.1, 144.7, 138.4, 135.1, 134.8, 132.9, 132.6, 131.5, 129.7, 129.3, 128.8, 127.5, 126.7, 126.0, 125.5, 124.4, 123.2, 120.4, 119.1, 116.0, 113.4, 113.3, 105.2, 103.4, 78.0, 56.5, 55.8, 38.7, 30.9, 21.5; IR (film): 3026, 2940, 2839, 1805, 1713, 1667, 1593, 1470, 1360, 1335, 1256, 1171, 1111, 968, 905 cm^{-1}; HRMS (ESI) Calcd for $C_{42}H_{38}N_3O_7S$ ([M+H]$^+$) 728.2425. Found 728.2422.

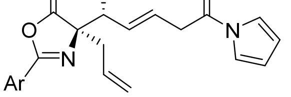

Ar = 2,6-(MeO)$_2$C$_6$H$_3$

RS-**4ea:** HPLC OD3, H/IPA = 4:1, flow rate = 1.0 mL/min, 40 °C, λ = 254 nm, 10.5 min (minor diastereomer), 13.4 min (major isomer of major diastereomer), 20.9 min (minor diastereomer), 23.0 min (minor isomer of major diastereomer); ^{1}H NMR (400 MHz, CDCl$_3$) δ 7.40 (2H, d, J = 7.5 Hz), 7.37 (1H, t, J = 8.3 Hz), 7.31 (2H, t, J = 7.5 Hz), 7.28-7.21 (3H, m), 6.57 (2H, d, J = 8.3 Hz), 6.22 (2H, t, J = 2.1 Hz), 6.16 (1H, ddt, J = 15.2, 9.2, 1.2 Hz), 5.88 (1H, dt, J = 15.2, 6.7 Hz), 5.69 (1H, dddd, J = 17.1, 10.5, 8.6, 6.2 Hz), 5.10 (1H, d, J = 17.1 Hz), 5.09 (1H, d, J = 10.5 Hz), 3.80 (1H, d, J = 9.2 Hz), 3.79 (6H, s), 3.63 (1H, ddd, J = 16.6, 6.7, 1.2 Hz), 3.53 (1H, ddd, J = 16.6, 6.7, 1.2 Hz), 2.52 (1H, dd, J = 13.9, 8.6 Hz), 2.41 (1H, dd, J = 13.9, 6.2 Hz); ^{13}C NMR (101 MHz, CDCl$_3$) δ 179.9, 168.2, 159.4, 157.7, 138.4, 133.0, 132.7, 131.1, 129.3, 128.7, 127.4, 125.3, 120.0, 119.2, 113.4, 106.2, 104.0, 77.4, 56.1, 55.7, 40.1, 38.7; IR (film): 2940, 2839, 1807, 1715, 1674, 1595, 1476, 1331, 1304, 1256, 1113, 964, 918 cm^{-1}; HRMS (ESI) Calcd for $C_{29}H_{29}N_2O_5$ ([M+H]$^+$) 485.2071. Found 485.2068.

Characterization of δ-Aryl Dienyl Phenylketone 5:

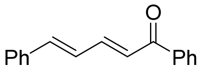

5a: ^{1}H NMR (400 MHz, CDCl$_3$) δ 7.97 (2H, d, J = 7.5 Hz), 7.60 (1H, ddd, J = 15.0, 7.1, 3.3 Hz), 7.56 (1H, tt, J = 7.5, 1.6 Hz), 7.49 (2H, d, J = 6.5 Hz), 7.48 (2H, tt, J = 7.5, 1.6 Hz), 7.37 (2H, tt, J = 6.5, 1.8 Hz), 7.31 (1H, tt, J = 6.5, 1.8 Hz), 7.09 (1H, d, J = 15.0 Hz), 7.04 (1H, dd, J = 15.6, 7.1 Hz), 6.99 (1H, dd, J = 15.6, 3.3 Hz); ^{13}C NMR (101 MHz, CDCl$_3$) δ 190.6, 144.9, 142.0, 138.4, 136.2, 132.8, 129.3,

129.0, 128.7, 128.5, 127.4, 127.1, 125.6; IR (film): 3057, 3028, 1655, 1599, 1572, 1447, 1350, 1287, 1250, 1150, 1016, 997 cm^{-1}; HRMS (ESI) Calcd for $C_{17}H_{14}ONa$ ([M+Na]$^+$) 257.0937. Found 257.0935.

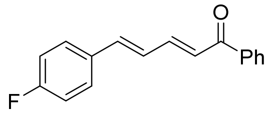

5b: ^{1}H NMR (400 MHz, $CDCl_3$) δ 7.97 (2H, d, J = 7.8 Hz), 7.58 (1H, ddd, J = 14.9, 8.7, 1.4 Hz), 7.56 (1H, t, J = 7.8 Hz), 7.48 (2H, t, J = 7.8 Hz), 7.47 (2H, dd, $J_{H\text{-}H}$ = 8.6 Hz, $J_{F\text{-}H}$ = 5.4 Hz), 7.08 (1H, d, J = 14.9 Hz), 7.06 (2H, t, $J_{H\text{-}H}$ = $J_{F\text{-}H}$ = 8.6 Hz), 6.97 (1H, dd, J = 15.6, 1.4 Hz), 6.92 (1H, dd, J = 15.6, 8.7 Hz); ^{13}C NMR (101 MHz, $CDCl_3$) δ 190.5, 163.4 (d, $J_{F\text{-}C}$ = 250.7 Hz), 144.7, 140.6, 138.3, 132.8, 132.5 (d, $J_{F\text{-}C}$ = 2.9 Hz), 129.1 (d, $J_{F\text{-}C}$ = 7.7 Hz), 128.7, 128.5, 126.9 (d, $J_{F\text{-}C}$ = 2.9 Hz), 125.6, 116.1 (d, $J_{F\text{-}C}$ = 22.3 Hz); ^{19}F NMR (376 MHz, $CDCl_3$) δ –111.1; IR (film): 3067, 1651, 1574, 1504, 1356, 1327, 1288, 1252, 1223, 1148, 1011 cm^{-1}; HRMS (ESI) Calcd for $C_{17}H_{13}OFNa$ ([M+Na]$^+$) 275.0843. Found 275.0844.

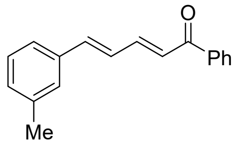

5c: ^{1}H NMR (400 MHz, $CDCl_3$) δ 7.97 (2H, dt, J = 7.3, 1.5 Hz), 7.60 (1H, ddd, J = 14.9, 8.6, 1.7 Hz), 7.57 (1H, tt, J = 7.3, 1.5 Hz), 7.49 (2H, tt, J = 7.3, 1.5 Hz), 7.32 (1H, s), 7.31 (1H, d, J = 7.3 Hz), 7.26 (1H, t, J = 7.3 Hz), 7.14 (1H, d, J = 7.3 Hz), 7.08 (1H, d, J = 14.9 Hz), 7.03 (1H, dd, J = 15.6, 8.6 Hz), 6.98 (1H, dd, J = 15.6, 1.7 Hz), 2.37 (3H, s); ^{13}C NMR (101 MHz, $CDCl_3$) δ 190.7, 145.1, 142.3, 138.6, 138.4, 136.2, 132.8, 130.2, 128.9, 128.7, 128.5, 128.2, 126.9, 125.4, 124.6, 21.5; IR (film): 3026, 2920, 1655, 1599, 1584, 1447, 1348, 1285, 1258, 1016 cm^{-1}; HRMS (ESI) Calcd for $C_{18}H_{16}ONa$ ([M+Na]$^+$) 271.1093. Found 271.1091.

Characterization of 1,6-Adduct 6 (see Methods in the main manuscript)**:**

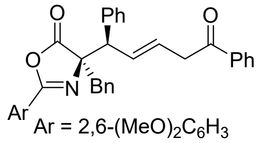

RR-**6aa:** HPLC OD3, H/IPA = 10:1, flow rate = 1.0 mL/min, 40 °C, λ = 230 nm, 23.1 min (minor diastereomer), 31.5 min (minor diastereomer), 34.9 min (major isomer of major diastereomer), 39.0 min (minor isomer of major diastereomer); ^{1}H NMR (400 MHz, $CDCl_3$) δ 7.93 (2H, d, J = 7.4 Hz), 7.51 (1H, tt, J = 7.4, 1.5 Hz), 7.41 (2H, t, J = 7.4 Hz), 7.38 (2H, d, J = 7.6 Hz), 7.28-7.14 (9H, m), 6.40 (2H, d, J = 8.4 Hz), 6.35 (1H, dd, J = 15.4, 9.9 Hz), 6.06 (1H, dt, J = 15.4, 6.8 Hz), 3.97 (1H, d, J = 9.9 Hz), 3.85 (1H, ddd, J = 17.1, 6.8, 1.2 Hz), 3.78 (1H, ddd, J = 17.1, 6.8, 1.2 Hz), 3.55 (6H, s), 3.45 (1H, d, J = 13.6 Hz), 3.16 (1H, d, J = 13.6 Hz); ^{13}C NMR (101 MHz, $CDCl_3$) δ 197.8, 179.0, 159.4, 157.9, 138.3, 136.6, 134.7, 133.2, 132.8, 131.7, 130.9, 129.5, 128.7, 128.3, 128.2, 127.9, 127.5, 127.2, 126.8, 105.1, 103.5, 78.2, 56.2, 55.8, 42.9, 42.4; IR (film): 3030, 2936, 2839, 1805, 1661, 1595, 1476, 1454, 1431, 1300, 1256, 1113, 962, 908 cm^{-1}; HRMS (ESI) Calcd for $C_{35}H_{32}NO_5$ ([M+H]$^+$) 546.2275. Found 545.2271.

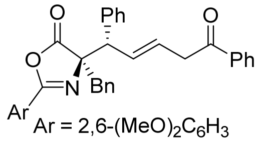

RS-**6aa:** HPLC OD3, H/IPA = 10:1, flow rate = 1.0 mL/min, 40 °C, λ = 230 nm, 23.1 min (minor isomer of major diastereomer), 31.5 min (major isomer of major diastereomer), 35.0 min (minor diastereomer), 38.8 min (minor diastereomer); ^{1}H NMR (400 MHz, $CDCl_3$) δ 7.85 (2H, d, J = 7.7 Hz), 7.49 (2H, d, J = 7.0 Hz), 7.42 (1H, tt, J = 7.7, 1.4 Hz), 7.38-7.23 (6H, m), 7.19-7.13 (3H, m), 7.13-7.06 (2H, m), 6.46 (2H, d, J = 8.6 Hz), 6.15 (1H, dd, J = 15.4, 9.8 Hz), 5.94 (1H, dt, J = 15.4, 6.5 Hz), 3.86 (1H, d, J = 9.8 Hz), 3.73 (1H, ddd, J = 16.4, 6.5, 1.4 Hz), 3.61 (6H, s), 3.60 (1H, ddd, J = 16.4, 6.5, 1.4 Hz), 3.10 (1H, d, J = 13.5 Hz), 2.87 (1H, d, J = 13.5 Hz); ^{13}C NMR (101 MHz, $CDCl_3$) δ 197.7, 179.9, 159.3, 157.3, 138.8, 136.4, 134.6, 133.0, 132.8, 131.8, 130.7, 129.4, 128.7, 128.6, 128.4, 127.9, 127.3, 127.1, 126.7, 105.7, 103.6, 78.5, 56.8, 55.9, 42.8, 41.6; IR (film): 3030, 2938, 2839, 1805, 1674, 1595, 1476, 1454, 1433, 1300, 1256, 1113, 964, 908 cm^{-1}; HRMS (ESI) Calcd for $C_{35}H_{32}NO_5$ ([M+H]$^+$) 546.2275. Found 546.2270.

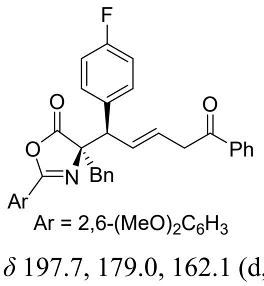

RR-**6ab:** HPLC OX3, H/EtOH = 19:1, flow rate = 1.0 mL/min, λ = 254 nm, 23.1 min (minor diastereomer), 25.2 min (minor isomer of major diastereomer), 29.2 min (minor diastereomer), 34.5 min (major isomer of major diastereomer); ^{1}H NMR (400 MHz, $CDCl_3$) δ 7.93 (2H, d, J = 7.4 Hz), 7.52 (1H, tt, J = 7.4, 1.5 Hz), 7.42 (2H, t, J = 7.4 Hz), 7.32 (2H, dd, $J_{H\text{-}H}$ = 8.5 Hz, $J_{F\text{-}H}$ = 5.6 Hz), 7.25 (1H, t, J = 8.5 Hz), 7.24-7.16 (5H, m), 6.92 (2H, t, $J_{H\text{-}H}$ = $J_{F\text{-}H}$ = 8.5 Hz), 6.41 (2H, d, J = 8.5 Hz), 6.31 (1H, dd, J = 15.4, 9.8 Hz), 6.06 (1H, dt, J = 15.4, 6.8 Hz), 3.97 (1H, d, J = 9.8 Hz), 3.86 (1H, ddd, J = 17.6, 6.8, 1.0 Hz), 3.80 (1H, ddd, J = 17.6, 6.8, 1.0 Hz), 3.57 (6H, s), 3.43 (1H, d, J = 13.6 Hz), 3.16 (1H, d, J = 13.6 Hz); ^{13}C NMR (101 MHz, $CDCl_3$) δ 197.7, 179.0, 162.1 (d, $J_{F\text{-}C}$ = 245.9 Hz), 159.3, 158.0, 136.6, 134.6, 134.1 (d, $J_{F\text{-}C}$ = 2.9 Hz), 133.2, 132.9, 131.5, 131.1 (d, $J_{F\text{-}C}$ = 7.7 Hz), 130.8, 128.7, 128.3, 127.9, 127.7, 126.9, 114.9 (d, $J_{F\text{-}C}$ = 21.3 Hz), 105.0, 103.5, 78.2, 55.8, 55.3, 42.7, 42.3; ^{19}F NMR (376 MHz, $CDCl_3$) δ –115.6; IR (film): 3030, 2938, 2839, 1805, 1661, 1595, 1508, 1476, 1433, 1300, 1256, 1113, 964, 908 cm^{-1}; HRMS (ESI) Calcd for $C_{35}H_{31}NO_5F$ ([M+H]$^+$) 564.2186. Found 564.2183.

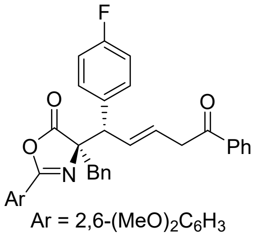

RS-**6ab:** HPLC OX3, H/EtOH = 19:1, flow rate = 1.0 mL/min, λ = 254 nm, 22.9 min (minor isomer of major diastereomer), 25.3 min (minor diastereomer), 28.8 min (major isomer of major diastereomer), 34.7 min (minor diastereomer); ^{1}H NMR (400 MHz, $CDCl_3$) *δ* 7.86 (2H, d, *J* = 7.8 Hz), 7.443_7 (2H, dd, $J_{H\text{-}H}$ = 8.9 Hz, $J_{F\text{-}H}$ = 5.5 Hz), 7.443_6 (1H, t, *J* = 7.8 Hz), 7.33 (2H, t, *J* = 7.8 Hz), 7.30 (1H, t, *J* = 8.5 Hz), 7.22-7.14 (3H, m), 7.14-7.07 (2H, m), 7.02 (2H, t, $J_{H\text{-}H} = J_{F\text{-}H}$ = 8.9 Hz), 6.46 (2H, d, *J* = 8.5 Hz), 6.13 (1H, dd, *J* = 15.4, 9.7 Hz), 5.94 (1H, dt, *J* = 15.4, 6.4 Hz), 3.86 (1H, d, *J* = 9.7 Hz), 3.75 (1H, ddd, *J* = 16.6, 6.4, 1.3 Hz), 3.63 (1H, ddd, *J* = 16.6, 6.4, 1.3 Hz), 3.61 (6H, s), 3.11 (1H, d, *J* = 13.5 Hz), 2.88 (1H, d, *J* = 13.5 Hz); ^{13}C NMR (101 MHz, $CDCl_3$) *δ* 197.6, 179.8, 162.2 (d, $J_{F\text{-}C}$ = 246.8 Hz), 159.3, 157.4, 136.4, 134.7 (d, $J_{F\text{-}C}$ = 2.9 Hz), 134.5, 133.1, 132.9, 131.5, 131.0 (d, $J_{F\text{-}C}$ = 7.7 Hz), 130.7, 128.6, 128.4, 128.0, 127.4, 126.8, 115.4 (d, $J_{F\text{-}C}$ = 21.3 Hz), 105.5, 103.6, 78.4, 55.9, 55.8, 42.7, 41.7; ^{19}F NMR (376 MHz, $CDCl_3$) *δ* –115.4; IR (film): 3032, 2938, 2839, 1805, 1674, 1595, 1508, 1476, 1433, 1300, 1256, 1223, 1111, 964, 908 cm^{-1}; HRMS (ESI) Calcd for $C_{35}H_{31}NO_5F$ ([M+H]$^+$) 564.2186. Found 565.2182.

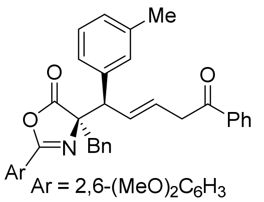

RR-**6ac:** The absolute configuration of *RR*-**6ac** was determined by X-ray crystallographic analysis (see Supplymentary Figure 105). HPLC OX3, H/EtOH = 10:1, flow rate = 1.0 mL/min, 40 °C, λ = 254 nm, 12.7 min (minor diastereomer), 15.8 min (minor isomer of major diastereomer), 17.7 min (minor diastereomer), 20.0 min (major isomer of major diastereomer); ^{1}H NMR (400 MHz, $CDCl_3$) *δ* 7.93 (2H, d, *J* = 7.6 Hz), 7.52 (1H, tt, *J* = 7.6, 1.6 Hz), 7.42 (2H, t, *J* = 7.6 Hz), 7.26 (1H, t, *J* = 8.4 Hz), 7.23-7.10 (8H, m), 7.01 (1H, d, *J* = 7.6 Hz), 6.41 (2H, d, *J* = 8.4 Hz), 6.32 (1H, dd, *J* = 15.4, 9.9 Hz), 6.04 (1H, dt, *J* = 15.4, 6.7 Hz), 3.92 (1H, d, *J* = 9.9 Hz), 3.85 (1H, ddd, *J* = 17.1, 6.7, 1.4 Hz), 3.78 (1H, ddd, *J* = 17.1, 6.7, 1.4 Hz), 3.57 (6H, s), 3.42 (1H, d, *J* = 13.6 Hz), 3.15 (1H, d, *J* = 13.6 Hz), 2.27 (3H, s); ^{13}C NMR (101 MHz, $CDCl_3$) *δ* 197.9, 179.1, 159.5, 157.8, 138.3, 137.8, 136.7, 134.8, 133.2, 132.8, 131.9, 130.9, 130.3, 128.7, 128.3, 128.1, 127.9, 127.3, 126.8, 126.5, 105.2, 103.5, 78.2, 56.3, 55.9, 42.9, 42.5, 21.5, one carbon atom was not found probably due to overlapping; IR (film): 3030, 2936, 2839, 1805, 1661, 1595, 1476, 1449, 1431, 1300, 1256, 1111, 964, 908 cm^{-1}; HRMS (ESI) Calcd for $C_{36}H_{34}NO_5$ ([M+H]$^+$) 560.2437. Found 560.2430.

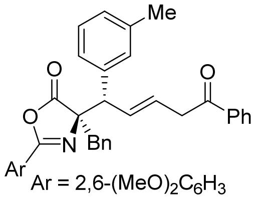

RS-**6ac:** HPLC OX3, H/EtOH = 10:1, flow rate = 1.0 mL/min, 40 °C, λ = 254 nm, 12.7 min (minor isomer of major diastereomer), 15.8 min (minor diastereomer), 17.6 min (major isomer of major diastereomer), 20.0 min (minor diastereomer); ^{1}H NMR (400 MHz, $CDCl_3$) *δ* 7.86 (2H, d, *J* = 7.6 Hz), 7.43 (1H, tt, *J* = 7.6, 1.1 Hz), 7.36-7.21 (6H, m), 7.20-7.14 (3H, m), 7.14-7.05 (3H, m), 6.47 (2H, d, *J* = 8.6 Hz), 6.15 (1H, dd, *J* = 15.3, 9.8 Hz), 5.94 (1H, dt, *J* = 15.3, 6.5 Hz), 3.83 (1H, d, *J* = 9.8 Hz), 3.73 (1H, ddd, *J* = 16.4, 6.5, 1.4 Hz), 3.62 (6H, s), 3.61 (1H, ddd, *J* = 16.4, 6.5, 1.4 Hz), 3.11 (1H, d, *J* = 13.6 Hz), 2.90 (1H, d, *J* = 13.6 Hz), 2.36 (3H, s); ^{13}C NMR (101 MHz, $CDCl_3$) *δ* 197.7, 180.0, 159.4, 157.2, 138.7, 138.1, 136.5, 134.7, 133.0, 132.8, 131.9, 130.7, 130.2, 128.6, 128.4, 128.1, 127.9, 126.9, 126.7, 126.3, 105.8, 103.6, 78.5, 56.8, 55.9, 42.8, 41.6, 21.7, one carbon atom was not found probably due to overlapping; IR (film): 3030, 2938, 2839, 1805, 1674, 1595, 1476, 1449, 1433, 1298, 1256, 1111, 964, 908 cm^{-1}; HRMS (ESI) Calcd for $C_{36}H_{34}NO_5$ ([M+H]$^+$) 560.2437. Found 560.2430.

Characterization of δ-Aryl Dienylidene Maronate 7:

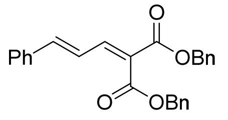

7: ^{1}H NMR (400 MHz, $CDCl_3$) *δ* 7.58 (1H, d, *J* = 11.6 Hz), 7.45-7.39 (2H, m), 7.39-7.30 (13H, m), 7.18 (1H, dd, *J* = 15.4, 11.6 Hz), 7.01 (1H, d, *J* = 15.4 Hz), 5.34 (2H, s), 5.26 (2H, s); ^{13}C NMR (101 MHz, $CDCl_3$) *δ* 165.1, 164.7, 146.5, 145.3, 135.9, 135.8, 135.7, 130.1, 129.0, 128.8, 128.7_4, 128.7_1, 128.5, 128.4, 128.3, 128.0, 124.2, 123.4, 67.3, 67.2; IR (film): 3032, 2953, 1717, 1616, 1589, 1450, 1283, 1231, 1150, 1155, 980 cm^{-1}; HRMS (ESI) Calcd for $C_{26}H_{22}O_4Na$ ([M+Na]$^+$) 421.1410. Found 421.1417.

Characterization of 1,6-Adduct 8 (see Methods in the main manuscript):

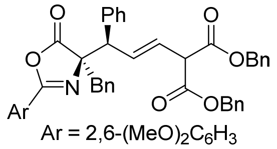

RR-**8**: HPLC OZ3, H/EtOH = 19:1, flow rate = 1.0 mL/min, 40 °C, λ = 254 nm, 22.9 min (minor diastereomer), 37.0 min (minor isomer of major diastereomer), 50.7 min (major isomer of major diastereomer), 54.6 min (minor diastereomer); ^{1}H NMR (400 MHz, $CDCl_3$) *δ* 7.33 (2H, d, *J* = 8.0 Hz), 7.27-7.15 (20H, m), 6.44 (1H, dd, *J* = 15.4, 10.0 Hz), 6.40 (2H, d, *J* = 8.4 Hz), 6.05 (1H, dd, *J* = 15.4, 9.2 Hz), 5.13 (1H, d, *J* = 12.2 Hz), 5.10 (1H, d, *J* = 12.3 Hz), 5.09 (1H, d, *J* = 12.2 Hz), 5.06 (1H, d, *J* = 12.3 Hz), 4.25 (1H, d, *J* = 9.2 Hz), 3.95 (1H, d, *J* = 10.0 Hz), 3.55 (6H, s), 3.35 (1H, d, *J* = 13.5 Hz), 3.08 (1H, d, *J* = 13.5 Hz); ^{13}C NMR (101 MHz, $CDCl_3$) *δ* 178.8, 167.6, 167.5, 159.4, 158.1, 137.5, 135.2, 135.1, 134.5, 134.3, 132.9, 131.0, 129.6, 128.6_0, 128.5_7, 128.4_3, 128.3_7, 128.3, 128.2, 128.1, 128.0, 127.9, 127.4, 126.9, 125.8, 105.0, 103.5, 78.1, 67.6, 67.4, 55.9, 55.8, 42.8; IR (film): 3032, 2938, 2839, 1807, 1732, 1661, 1595, 1476, 1454, 1433, 1300, 1256, 1146, 1113, 962, 908 cm^{-1}; HRMS (ESI) Calcd for $C_{44}H_{40}NO_8$ ([M+H]$^+$) 710.2748. Found

710.2749.

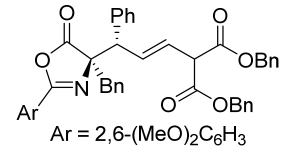

RS-**8:** HPLC OZ3, H/EtOH = 19:1, flow rate = 1.0 mL/min, 40 °C, λ = 254 nm, 22.9 min (minor isomer of major diastereomer), 36.9 min (minor diastereomer), 50.7 min (minor diastereomer), 54.5 min (major isomer of major diastereomer); ^{1}H NMR (400 MHz, $CDCl_3$) δ 7.49 (2H, d, *J* = 7.3 Hz), 7.35 (2H, t, *J* = 7.3 Hz), 7.27 (1H, t, *J* = 8.4 Hz), 7.27-7.13 (15H, m), 7.13-7.07 (2H, m), 6.43 (2H, d, *J* = 8.4 Hz), 6.27 (1H, dd, *J* = 15.3, 9.9 Hz), 6.00 (1H, dd, *J* = 15.3, 8.9 Hz), 5.08 (1H, d, *J* = 12.2 Hz), 5.05 (1H, d, *J* = 12.1 Hz), 5.02 (1H, d, *J* = 12.1 Hz), 4.97 (1H, d, *J* = 12.2 Hz), 4.07 (1H, dd, *J* = 8.9, 0.7 Hz), 3.89 (1H, d, *J* = 9.9 Hz), 3.54 (6H, s), 3.11 (1H, d, *J* = 13.5 Hz), 2.86 (1H, d, *J* = 13.5 Hz); ^{13}C NMR (101 MHz, $CDCl_3$) δ 179.6, 167.4, 167.3, 159.3, 157.6, 138.3, 135.3, 135.2, 134.5, 134.3, 132.8, 130.7, 129.4, 128.7, 128.5, 128.4, 128.3, 128.2, 128.0, 127.9, 127.5, 126.7, 125.3, 105.6, 103.6, 78.3, 67.4, 67.3, 55.9, 55.7, 41.6, two carbon atoms were not found probably due to overlapping; IR (film): 3032, 2940, 2839, 1807, 1732, 1670, 1595, 1476, 1454, 1298, 1256, 1113, 964, 907 cm^{-1}; HRMS (ESI) Calcd for $C_{44}H_{40}NO_8$ ([M+H]$^+$) 710.2748. Found 710.2747.

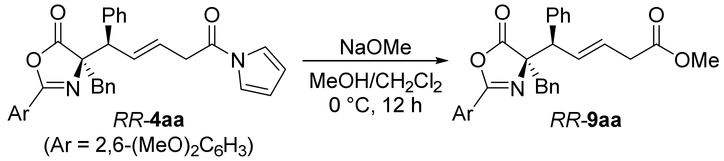

Methanolysis of *N*-Acylpyrrole Moiety of *RR*-4aa: A solution of *RR*-**4aa** (96.2 mg, 0.18 mmol) in CH_2Cl_2 (1.8 mL) was treated with a solution of NaOMe (10.8 mg, 0.20 mmol) in methanol (1.8 mL) at 0 °C for 12 h under Ar atmosphere. The whole mixture was poured into an ice-cooled, saturated aqueous solution of NH_4Cl. The aqueous phase was extracted with CH_2Cl_2 twice and the organic phases were washed with brine. The combined organic extracts were dried over Na_2SO_4 and filtered. All volatiles were removed by evaporation to give crude material, which was purified by column chromatography on silica gel (H/ethyl acetate (EA) = 1:1 as eluent) afforded *RR*-**9aa** in 91% yield (81.8 mg, 0.16 mmol). *RR*-**9aa:** ^{1}H NMR (400 MHz, $CDCl_3$) δ 7.37 (2H, d, *J* = 6.8 Hz), 7.29-7.14 (9H, m), 6.41 (2H, d, *J* = 8.4 Hz), 6.28 (1H, dd, *J* = 15.0, 9.6 Hz), 5.88 (1H, dt, *J* = 15.0, 7.2 Hz), 3.93 (1H, d, *J* = 9.6 Hz), 3.63 (3H, s), 3.57 (6H, s), 3.43 (1H, d, *J* = 13.2 Hz), 3.20 (1H, dd, *J* = 16.9, 7.2 Hz), 3.15 (1H, d, *J* = 13.2 Hz), 3.13 (1H, dd, *J* = 16.9, 7.2 Hz); ^{13}C NMR (101 MHz, $CDCl_3$) δ 178.9, 171.8, 159.4, 157.9, 138.1, 134.6, 132.8, 131.7, 130.9, 129.5, 128.2, 127.9, 127.3, 126.8, 126.6, 105.1, 103.5, 78.1, 56.0, 55.8, 51.8, 42.8, 37.8; IR (film): 3030, 2951, 2839, 1807, 1734, 1661, 1595, 1476, 1454, 1433, 1256, 1113, 964, 908 cm^{-1}; HRMS (ESI) Calcd for $C_{30}H_{30}NO_6$ ([M+H]$^+$) 500.2068. Found 500.2065.

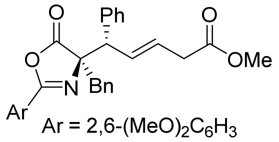

RS-**9aa** was obtained in 83% yield (194.7 mg, 0.39 mmol) by following the above procedure using *RS*-**4aa** as a starting material. *RS*-**9aa:** ^{1}H NMR (400 MHz, $CDCl_3$) δ 7.51 (2H, d, *J* = 7.4 Hz), 7.36 (2H, t, *J* = 7.4 Hz), 7.30 (1H, t, *J* = 8.3 Hz), 7.27 (1H, t, *J* = 7.4 Hz), 7.22-7.14 (3H, m), 7.14-7.07 (2H, m), 6.47 (2H, d, *J* = 8.3 Hz), 6.07 (1H, dd, *J* = 15.0, 9.6 Hz), 5.80 (1H, dt, *J* = 15.0, 7.3 Hz), 3.84 (1H, d, *J* = 9.6 Hz), 3.63 (6H, s), 3.57 (3H, s), 3.10 (1H, d, *J* = 13.6 Hz), 3.07 (1H, ddd, *J* = 16.5, 7.3, 1.2 Hz), 3.00 (1H, dd, *J* = 16.5, 7.3 Hz), 2.86 (1H, d, *J* = 13.6 Hz); ^{13}C NMR (101 MHz, $CDCl_3$) δ 180.0, 171.6, 159.4, 157.4, 138.9, 134.7, 132.8, 132.0, 130.7, 129.5, 128.7, 128.0, 127.4, 126.7, 126.0, 105.8, 103.7, 78.7, 56.6, 55.9, 51.8, 41.6, 38.0; IR (film): 3028, 2949, 2839, 1805, 1736, 1672, 1595, 1476, 1454, 1433, 1300, 1256, 1169, 1113, 964, 910 cm^{-1}; HRMS (ESI) Calcd for $C_{30}H_{30}NO_6$ ([M+H]$^+$) 500.2068. Found 500.2063.

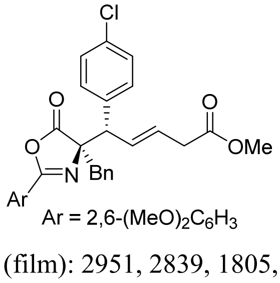

RS-**9ac** was obtained in 81% yield (104.4 mg, 0.20 mmol) by following the above procedure using *RS*-**4ac** as a starting material. *RS*-**9ac:** ^{1}H NMR (400 MHz, $CDCl_3$) δ 7.43 (2H, d, *J* = 8.8 Hz), 7.32 (2H, d, *J* = 8.8 Hz), 7.31 (1H, t, *J* = 8.5 Hz), 7.22-7.16 (3H, m), 7.16-7.08 (2H, m), 6.48 (2H, d, *J* = 8.5 Hz), 6.05 (1H, ddt, *J* = 15.2, 9.9, 1.2 Hz), 5.79 (1H, dt, *J* = 15.2, 7.2 Hz), 3.82 (1H, d, *J* = 9.9 Hz), 3.63 (6H, s), 3.60 (3H, s), 3.11 (1H, d, *J* = 13.6 Hz), 3.09 (1H, ddd, *J* = 16.4, 7.2, 1.2 Hz), 3.01 (1H, ddd, *J* = 16.4, 7.2, 1.2 Hz), 2.88 (1H, d, *J* = 13.6 Hz); ^{13}C NMR (101 MHz, $CDCl_3$) δ 179.7, 171.6, 159.4, 157.6, 137.5, 134.5, 133.3, 133.0, 131.4, 130.8_2, 130.7_6, 128.9, 128.1, 126.9, 126.6, 105.6, 103.7, 78.4, 56.0, 55.8, 51.9, 41.7, 38.0; IR (film): 2951, 2839, 1805, 1736, 1670, 1595, 1476, 1433, 1300, 1256, 1165, 1113, 964, 908 cm^{-1}; HRMS (ESI) Calcd for $C_{30}H_{29}NO_6Cl$ ([M+H]$^+$) 534.1678. Found 534.1678.

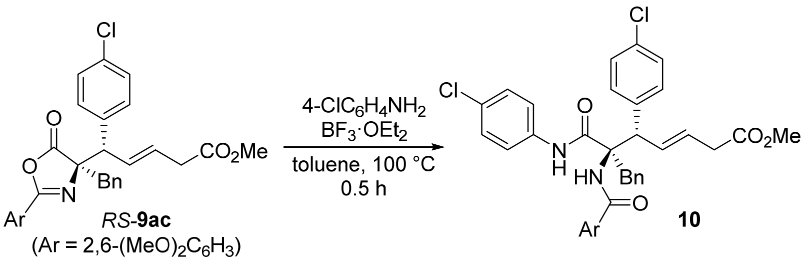

Azlactone Ring-Opening Reaction of *RS*-9ac: The monoester *RS*-**9ac** (25.7 mg, 0.048 mmol) and 4-chloroaniline (13.5 mg, 0.11 mmol) were placed in an oven-dried test tube and dissolved into toluene (0.48 mL). $BF_3 \cdot OEt_2$ (0.0067 mL, 0.053 mmol) was added to the solution and the reaction mixture was then stirred for 0.5 h at 100 °C under Ar atmosphere. After cooling to room temperature, the mixture was poured into water and the aqueous phase was extracted with EA twice. The combined organic extracts were dried over Na_2SO_4, filtrated, and concentrated. The subsequent purification of the crude material by column chromatography on silica gel (H/acetone = 2:1~1:2 as eluent) afforded **10** in 56% yield (16.9 mg, 0.027 mmol). The absolute configuration of **10** was determined by X-ray crystallographic analysis (see Supplymentary Figure 102). **10:** ^{1}H NMR (400 MHz, $CDCl_3$) δ 8.63 (1H, brs), 7.46 (2H, d, *J* = 7.8 Hz), 7.44 (2H, d, *J* = 8.9 Hz), 7.30 (2H, d, *J* = 8.9 Hz), 7.28-7.20 (4H, m), 7.19 (2H, d, *J* = 8.2 Hz), 7.08 (2H, d, *J* = 8.2 Hz), 6.51 (2H, d, *J* = 8.7 Hz), 6.35 (1H, dd, *J* = 15.4, 9.0 Hz), 6.25 (1H, brs), 5.76 (1H, dt, *J* = 15.4, 7.0 Hz), 4.14 (1H, brd, *J* = 5.0 Hz), 3.88 (1H, d, *J* = 14.4 Hz), 3.77 (1H, d, *J* = 14.4 Hz), 3.71 (3H, s), 3.61 (6H, s), 3.22 (1H, dd, *J* = 16.8, 7.0 Hz), 3.16 (1H, dd, *J* = 16.8, 7.0 Hz); ^{13}C NMR (101 MHz, $CDCl_3$) δ 172.6, 168.8, 164.8, 157.6, 138.0, 136.2, 136.0, 133.4, 132.9, 131.5, 131.4, 131.0, 129.7, 129.1, 128.5, 128.0, 127.4, 126.9, 122.0, 114.8, 104.1, 67.5, 55.8, 52.2_0, 52.1_7, 37.5; IR (film): 3366, 2949, 2839, 1732, 1680, 1593, 1489, 1472, 1435, 1398, 1250, 1107, 978, 907 cm^{-1}; HRMS (ESI) Calcd for $C_{36}H_{35}N_2O_6Cl_2$ ([M+H]$^+$) 661.1867. Found 661.1864.

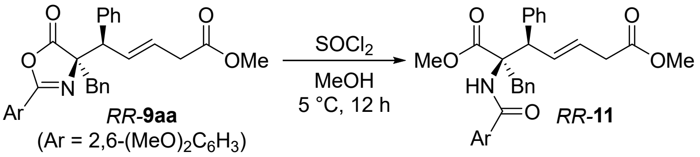

Methanolysis of Azlactone Moiety of *RR*-9aa: To a solution of *RR*-**9aa** (57.0 mg, 0.11 mmol) in methanol (1.1 mL) was introduced $SOCl_2$ (0.29 mL, 4.0 mmol) dropwise at 5 °C and the reaction mixture was stirred for 12 h at the same temperature. Then, a saturated aqueous solution of $NaHCO_3$ was added to quench the reaction. The aqueous phase was extracted with EA twice and the organic phases were washed with brine. The combined organic extracts were dried over Na_2SO_4 and filtered. After concentration, the residue was purified by column chromatography on silica gel (H/EA = 1:1 as eluent) to afford *RR*-**11** in 83% yield (48.4 mg, 0.091 mmol). *RR*-**11:** ^{1}H NMR (400 MHz, $CDCl_3$) δ 7.40 (2H, d, *J* = 6.8 Hz), 7.33-7.11 (9H, m), 6.74 (1H, brs), 6.53 (2H, d, *J* = 8.0 Hz), 6.31 (1H, dd, *J* = 14.7, 9.7 Hz), 5.81 (1H, dt, *J* = 14.7, 7.0 Hz), 4.43 (1H, d, *J* = 9.7 Hz), 4.38 (1H, d, *J* = 14.0 Hz), 3.76 (6H, s), 3.66 (3H, s), 3.60 (3H, s), 3.19 (1H, dd, *J* = 16.7, 7.0 Hz), 3.11 (1H, dd, *J* = 16.7, 7.0 Hz), 3.00 (1H, d, *J* = 14.0 Hz); ^{13}C NMR (101 MHz, $CDCl_3$) δ 172.5, 172.1, 165.2, 157.7, 139.6, 136.8, 132.7, 130.7, 130.6, 129.6, 128.1, 127.8, 127.1, 126.4, 125.2, 116.4, 104.0, 70.0, 56.8, 55.7, 52.2, 51.7, 38.6, 38.0; IR (film): 3397, 2951, 2839, 1734, 1668, 1595, 1495, 1472, 1435, 1252, 1206, 1161, 1111, 968, 910 cm^{-1}; HRMS (ESI) Calcd for $C_{31}H_{34}NO_7$ ([M+H]$^+$) 532.2330. Found 532.2324.

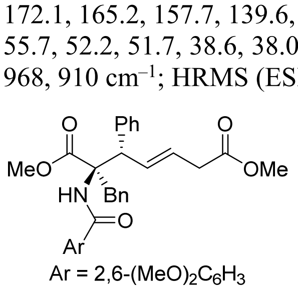

RS-**11** was obtained in 69% yield (50.6 mg, 0.095 mmol) by following the above procedure using *RS*-**9aa** as a starting material. *RS*-**11:** ^{1}H NMR (400 MHz, $CDCl_3$) δ 7.30-7.24 (4H, m), 7.24-7.12 (7H, m), 6.65 (1H, brs), 6.48 (2H, d, *J* = 8.8 Hz), 6.36 (1H, dd, *J* = 14.9, 10.4 Hz), 5.95 (1H, dt, *J* = 14.9, 7.1 Hz), 4.99 (1H, d, *J* = 10.4 Hz), 4.12 (1H, d, *J* = 13.4 Hz), 3.78 (3H, s), 3.68 (3H, s), 3.63 (6H, s), 3.40 (1H, d, *J* = 13.4 Hz), 3.20 (1H, dd, *J* = 16.5, 7.1 Hz), 3.15 (1H, dd, *J* = 16.5, 7.1 Hz); ^{13}C NMR (101 MHz, $CDCl_3$) δ 172.4, 172.0, 164.5, 158.2, 140.2, 137.0, 133.2, 130.9, 130.6, 129.0, 128.2, 127.9, 126.9, 126.5, 126.1, 115.6, 104.1, 70.5, 55.6, 52.7, 52.4, 51.9, 39.4, 38.3; IR (film): 3404, 3030, 2949, 2839, 1734, 1661, 1595, 1495, 1472, 1433, 1250, 1227, 1206, 1111, 972, 912 cm^{-1}; HRMS (ESI) Calcd for $C_{31}H_{34}NO_7$ ([M+H]$^+$) 532.2330. Found 532.2325.

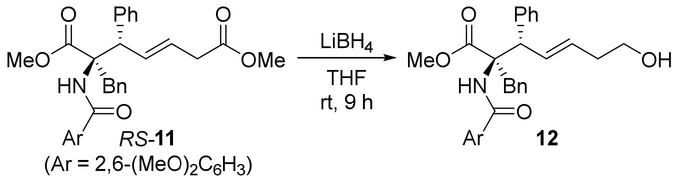

Site-Selective Reduction of *RS*-11: To a solution of *RS*-**11** (24.8 mg, 0.047 mmol) in THF (1.0 mL) was added $LiBH_4$ (20.3 mg, 0.93 mmol) at room temperature under Ar atmosphere. After 9 h of stirring, the reaction was quenched by adding water and the aqueous phase was extracted with EA twice. The organic phases were washed with brine. The combined organic extracts were dried over Na_2SO_4 and filtered. All volatiles were removed by evaporation and purification of the residue by column chromatography on silica gel (H/EA = 1:4 as eluent) afforded **12** in 95% yield (22.7 mg, 0.045 mmol). **12:** ^{1}H NMR (600 MHz, $CDCl_3$) δ 7.30-7.24 (3H, m), 7.24-7.13 (8H, m), 6.66 (1H, brs), 6.49 (2H, d, J = 8.4 Hz), 6.34 (1H, dd, J = 15.0, 10.2 Hz), 5.81 (1H, dt, J = 15.0, 6.9 Hz), 4.95 (1H, d, J = 10.2 Hz), 4.12 (1H, d, J = 14.1 Hz), 3.78 (3H, s), 3.69 (2H, t, J = 6.9 Hz), 3.64 (6H, s), 3.40 (1H, d, J = 14.1 Hz), 2.43 (1H, dq, J = 13.6, 6.9 Hz), 2.39 (1H, dq, J = 13.6, 6.9 Hz), 1.60 (1H, brs); ^{13}C NMR (151 MHz, $CDCl_3$) δ 172.5, 164.5, 158.2, 140.6, 136.9, 132.7, 131.0, 130.6, 130.5, 129.0, 128.2, 128.0, 126.9, 126.5, 115.6, 104.0, 70.4, 62.0, 55.7, 53.0, 52.5, 39.5, 36.3; IR (film): 3399, 3030, 2941, 2837, 1732, 1647, 1595, 1497, 1474, 1433, 1358, 1304, 1252, 1111, 1032, 972, 910 cm^{-1}; HRMS (ESI) Calcd for $C_{30}H_{34}NO_6$ ($[M+H]^+$) 504.2381. Found 504.2387.

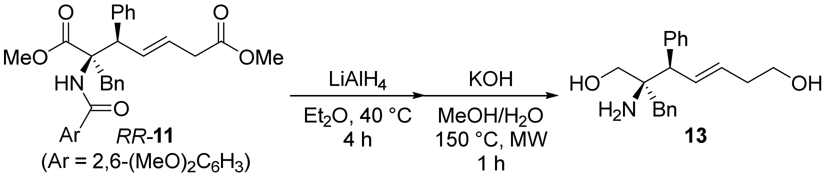

Reduction of Ester Moieties of *RR*-11 and Subsequent Deprotection on Nitrogen: $LiAlH_4$ (17.5 mg, 0.46 mmol) was placed in a test tube and suspended into dry Et_2O (0.50 mL) under Ar atmosphere. A solution of *RR*-**11** (12.2 mg, 0.023 mmol) in Et_2O (0.50 mL) was introduced slowly to the suspension at 0 °C and the reaction mixture was stirred for 4 h at 40 °C. The reaction was quenched by water and 1 N aqueous solution of NaOH at 0 °C. The aqueous phase was extracted with EA twice and the organic phases were washed with brine. The combined organic extracts were dried over Na_2SO_4 and filtered. All volatiles were removed under reduced pressure and the resulting crude residue was transferred to a test tube for a microwave apparatus with methanol (1.0 mL). Water (1.0 mL) and potassium hydroxide (0.17 g, 3.0 mmol) were added to the tube and the reaction mixture was stirred for 1 h at 150 °C under microwave irradiation. After addition of water, the aqueous phase was extracted with EA twice and the organic phases were washed with brine. The combined organic extracts were dried over Na_2SO_4, filtered, and concentrated. Purification of the residue by column chromatography on silica gel (EA/MeOH = 10:1 as eluent) afforded **13** in 78% yield (5.6 mg, 0.018 mmol). **13:** ^{1}H NMR (400 MHz, $CDCl_3$) δ 7.37-7.19 (8H, m), 7.17 (2H, d, J = 7.2 Hz), 6.21 (1H, dd, J = 14.6, 10.3 Hz), 5.61 (1H, dt, J = 14.6, 6.5 Hz), 3.68 (1H, dt, J = 12.2, 6.5 Hz), 3.66 (1H, dt, J = 12.2, 6.5 Hz), 3.60 (1H, d, J = 10.3 Hz), 3.27 (1H, d, J = 10.6 Hz), 3.14 (1H, d, J = 10.6 Hz), 2.89 (1H, d, J = 9.2 Hz), 2.74 (1H, d, J = 9.2 Hz), 2.39 (1H, dq, J = 13.2, 6.5 Hz), 2.34 (1H, dq, J = 13.2, 6.5 Hz), 1.68 (4H, brs); ^{13}C NMR (101 MHz, $CDCl_3$) δ 141.2, 137.2, 132.6, 130.9, 130.0, 129.5, 128.7, 128.4, 126.9, 126.7, 65.9, 62.0, 57.8, 55.6, 41.3, 36.3; IR (film): 3341, 3028, 2924, 1734, 1647, 1599, 1582, 1495, 1452, 1252, 1113, 1032, 974, 908 cm^{-1}; HRMS (ESI) Calcd for $C_{20}H_{26}NO_2$ ($[M+H]^+$) 312.1958. Found 312.1954.

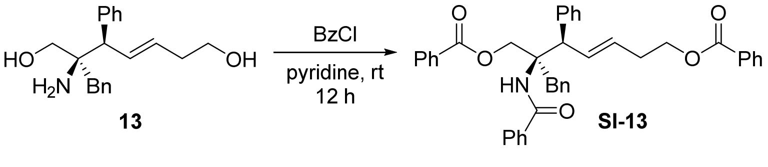

Benzoylation of Amino and Hydroxy Moieties of 13: To a solution of **13** (12.5 mg, 0.040 mmol) in pyridine (0.40 mL) was introduced benzoyl chloride (28.0 µL, 0.24 mmol) at room temperature under Ar atmosphere. After 12 h of stirring, the reaction mixture was poured into an ice-cooled, 1 N hydrochloric acid. The aqueous phase was extracted with EA twice and the organic phases were washed with brine. The combined organic extracts were dried over Na_2SO_4 and filtered. All volatiles were removed by evaporation and the resulting residue was purified by column chromatography on silica gel (H/EA = 10:1 as eluent) to give SI-**13** in 73% yield (18.1 mg, 0.029 mmol). The enantiomeric excess of SI-**13** was determined by HPLC analysis. SI-**13:** HPLC 99% ee, OZ3, H/IPA = 97:3, flow rate = 1.0 mL/min, 40 °C, λ

= 220 nm, 18.7 min (minor isomer of major diastereomer), 26.3 min (major isomer of major diastereomer), 38.6 min (minor diastereomer), 41.8 min (minor diastereomer); ^{1}H NMR (400 MHz, $CDCl_3$) δ 7.94 (2H, d, J = 7.4 Hz), 7.83 (2H, d, J = 7.4 Hz), 7.64 (2H, d, J = 7.4 Hz), 7.57 (1H, t, J = 7.4 Hz), 7.52-7.40 (6H, m), 7.40-7.27 (7H, m), 7.20-7.10 (3H, m), 7.10-7.02 (2H, m), 6.70 (1H, brs), 6.26 (1H, dd, J = 14.7, 9.6 Hz), 5.69 (1H, dt, J = 14.7, 6.9 Hz), 4.87 (1H, d, J = 9.6 Hz), 4.53 (1H, d, J = 12.0 Hz), 4.41 (1H, d, J = 12.0 Hz), 4.09 (1H, dt, J = 11.2, 6.9 Hz), 4.02_0 (1H, d, J = 13.8 Hz), 4.01_8 (1H, dt, J = 11.2, 6.9 Hz), 2.70 (1H, d, J = 13.8 Hz), 2.32 (1H, dq, J = 13.8, 6.9 Hz), 2.25 (1H, dq, J = 13.8, 6.9 Hz); ^{13}C NMR (101 MHz, $CDCl_3$) δ 168.1, 167.1, 166.4, 140.1, 136.7, 135.8, 133.8, 132.8, 132.3, 131.5, 130.6, 130.3, 130.1, 129.8, 129.6, 129.4, 128.9, 128.8, 128.4, 127.3, 126.8, 126.7, 67.7, 64.1, 62.8, 54.4, 39.0, 32.3; IR (film): 3370, 3061, 2957, 1717, 1663, 1531, 1489, 1450, 1314, 1269, 1111, 1070, 1026, 972, 908 cm^{-1}; HRMS (ESI) Calcd for $C_{41}H_{38}NO_5$ ([M+H]$^+$) 624.2744. Found 624.2733.

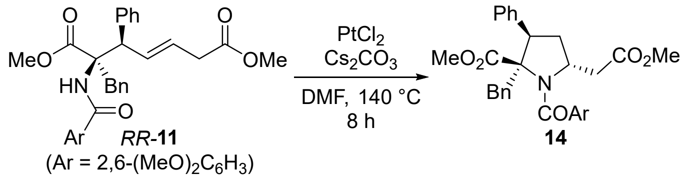

Cyclization Reaction of *RR*-11 Mediated by Platinum Chloride: *RR*-**11** (12.2 mg, 0.023 mmol), platinum chloride (6.12 mg, 0.023 mmol), and cesium carbonate (14.99 mg, 0.046 mmol) were suspended into DMF (0.23 mL) under Ar atmosphere. After 8 h of stirring at 140 °C, the reaction mixture was diluted with water. The aqueous phase was extracted with EA twice and the organic phases were washed with brine. The combined organic extracts were dried over Na_2SO_4, filtered, and concentrated. The resulting crude residue was analyzed by ^{1}H NMR (400 MHz) to determine diastereoselectivity (>20:1) and was purified by column chromatography on silica gel (H/EA = 1:4 as eluent) to afford **14** as a single diastereomer in 76% yield (9.3 mg, 0.017 mmol). The absolute configuration of **14** was determined by X-ray crystallographic analysis (see Supplymentary Figure 103). **14:** ^{1}H NMR (400 MHz, $CDCl_3$) δ 7.65 (2H, d, J = 6.8 Hz), 7.45-7.27 (9H, m), 6.59 (2H, d, J = 8.4 Hz), 4.20 (1H, ddd, J = 11.5, 7.7, 3.0 Hz), 4.06 (1H, d, J = 14.4 Hz), 3.95 (3H, s), 3.92 (3H, s), 3.65 (1H, dd, J = 13.1, 6.1 Hz), 3.51 (3H, s), 3.37 (3H, s), 3.25 (1H, d, J = 14.4 Hz), 2.81 (1H, td, J = 13.1, 7.7 Hz), 1.85 (1H, dd, J = 16.3, 3.0 Hz), 1.66 (1H, dd, J = 13.1, 6.1 Hz), 1.10 (1H, dd, J = 16.3, 11.5 Hz); ^{13}C NMR (101 MHz, $CDCl_3$) δ 171.5, 171.3, 167.2, 157.0_3, 156.9_9, 136.9, 136.3, 132.8, 130.8, 128.7, 128.6, 128.0, 127.9, 127.0, 115.8, 104.5, 104.3, 74.9, 56.2, 56.1, 55.9, 51.8, 51.4, 46.7, 38.5, 36.2, 33.6; IR (film): 2949, 2839, 1736, 1638, 1595, 1474, 1393, 1254, 1111, 910 cm^{-1}; HRMS (ESI) Calcd for $C_{31}H_{34}NO_7$ ([M+H]$^+$) 532.2330. Found 532.2324.

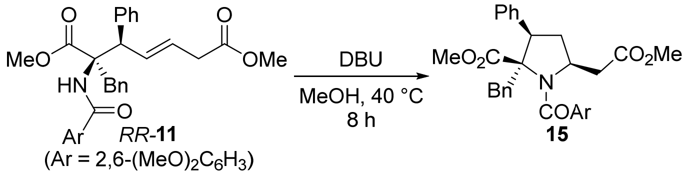

Cyclization Reaction of *RR*-11 under the Influence of DBU: *RR*-**11** (14.2 mg, 0.027 mmol) was placed in a test tube and dissolved into methanol (0.53 mL) under Ar atmosphere. DBU (80.0 µL, 0.53 mmol) was introduced to the solution and the reaction mixture was stirred for 8 h at 40 °C. After cooling to room temperature, the reaction was quenched by adding water. The aqueous phase was extracted with EA twice and the organic phases were washed with brine. The combined organic extracts were dried over Na_2SO_4 and filtered. All volatiles were removed by evaporation and the diastereomeric ratio of cyclization products was determined to be 4.4:1 by ^{1}H NMR (400 MHz) analysis of the crude residue. Purification of the residue was then performed by using preparative thin layer chromatography on silica gel (H/EA = 1:1 as eluent) to give **15** as a single diastereomer in 64% yield (9.2 mg, 0.017 mmol). **15:** ^{1}H NMR (400 MHz, $CDCl_3$) δ 7.62 (2H, d, J = 7.2 Hz), 7.42-7.22 (9H, m), 6.58 (1H, d, J = 8.0 Hz), 6.52 (1H, d, J = 8.0 Hz), 4.33 (1H, d, J = 15.0 Hz), 4.07 (1H, tdd, J = 9.0, 7.1, 4.9 Hz), 3.87 (3H, s), 3.72 (3H, s), 3.63 (1H, dd, J = 12.1, 7.1 Hz), 3.51 (3H, s), 3.46 (3H, s), 3.28 (1H, d, J = 15.0 Hz), 2.59 (1H, dd, J = 15.2, 9.0 Hz), 2.49 (1H, dd, J = 15.2, 4.9 Hz), 2.22 (1H, td, J = 12.1, 9.0 Hz), 2.15 (1H, dt, J = 12.1, 7.1 Hz); ^{13}C NMR (101 MHz, $CDCl_3$) δ 172.1, 171.9, 165.8, 158.7, 156.6, 137.4, 137.3, 132.2, 131.3, 128.7, 128.5, 128.2, 127.7, 126.5, 116.2, 104.8, 103.8, 77.2, 56.5, 55.6, 52.0, 51.5, 47.2, 39.8, 37.1, 36.9, one carbon atom was not found probably due to overlapping.; IR (film): 2947, 2837, 1734, 1628, 1593, 1474, 1433, 1398, 1254, 1111, 912 cm^{-1}; HRMS (ESI) Calcd for $C_{31}H_{34}NO_7$ ([M+H]$^+$) 532.2330. Found 532.2326.

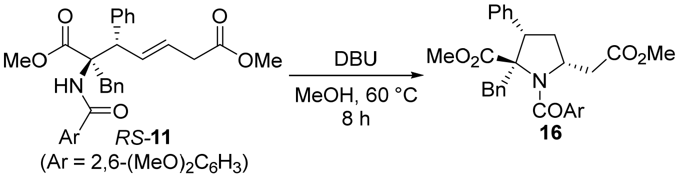

Cyclization Reaction of *RS*-11 under the Influence of DBU: A methanolic solution (1.0 mL) of *RS*-**11** (12.9 mg, 0.024 mmol) was treated with DBU (73.0 µL, 0.49 mmol) at 60 °C for 8 h under Ar atmosphere. The reaction mixture was diluted with water at room temperature. The aqueous phase was extracted with EA twice and the organic phases were washed with brine. The combined organic extracts were dried over Na_2SO_4, filtered, and concentrated. The diastereomeric ratio of cyclization products was determined to be 14:1 by ^{1}H NMR (400 MHz) analysis of the crude residue. The residue was purified by column chromatography on silica gel (H/EA = 1:4 as eluent) to afford **16** as a mixture of diastereomers in 88% yield (11.2 mg, 0.021 mmol). The absolute configuration of **16** was determined by X-ray crystallographic analysis (see Supplymentary Figure 104). **16:** ^{1}H NMR (400 MHz, $CDCl_3$) *major diastereomer δ* 7.40 (2H, t, *J* = 7.3 Hz), 7.33 (1H, t, *J* = 7.3 Hz), 7.28 (1H, t, *J* = 8.2 Hz), 7.22-7.11 (5H, m), 7.09-6.99 (2H, m), 6.58 (1H, d, *J* = 8.2 Hz), 6.56 (1H, d, *J* = 8.2 Hz), 4.32 (1H, tdd, *J* = 9.8, 6.3, 3.4 Hz), 4.00 (1H, dd, *J* = 13.4, 6.3 Hz), 3.95 (3H, s), 3.90 (3H, s), 3.89 (3H, s), 3.69 (1H, d, *J* = 14.4 Hz), 3.40 (3H, s), 2.93 (1H, d, *J* = 14.4 Hz), 2.36 (1H, dt, *J* = 12.2, 6.3 Hz), 2.19 (1H, dd, *J* = 16.6, 3.4 Hz), 1.43 (1H, ddd, *J* = 13.4, 12.2, 9.8 Hz) 1.40 (1H, dd, *J* = 16.6, 9.8 Hz); ^{13}C NMR (101 MHz, $CDCl_3$) *major diastereomer δ* 174.6, 171.6, 166.9, 157.7, 156.8, 137.8, 136.7, 132.3, 131.2, 128.5_7, 128.5_5, 127.5, 127.4, 126.6, 115.3, 104.6, 104.4, 73.9, 56.1_1, 56.0_5, 54.3, 52.6, 51.4, 49.3, 39.5, 36.2, 34.8; IR (film): 2947, 2839, 1734, 1632, 1595, 1474, 1400, 1362, 1302, 1254, 1171, 1111, 1032, 912 cm^{-1}; HRMS (ESI) Calcd for $C_{31}H_{34}NO_7$ ($[M+H]^+$) 532.2330. Found 532.2329.

Crystallographic Structure Determination of *RR*-4ad: The single crystal, which was obtained by the procedure described below, was mounted on MicroMesh. Data of X-ray diffraction were collected at 93 K on a Bruker D8 QUEST with CCD diffractometer with graphite-monochromated Mo/Kα radiation (λ = 0.71073 Å). An absorption correction was made using SADABS. The structure was solved by direct methods and Fourier syntheses, and refined by full-matrix least squares on F^2 by using SHELXL-2014.[11] All non-hydrogen atoms were refined with anisotropic displacement parameters. Hydrogen atoms bonded to nitrogen atoms were located from a difference synthesis and their coordinates and isotropic thermal parameters refined. The other hydrogen atoms were placed in calculated positions and isotropic thermal parameters refined.

Recrystallization of *RR*-4ad: Recrystallization was performed by using a H/$CHCl_3$ solvent system at room temperature to afford single crystals of *RR*-**4ad**. The crystallographic data and ORTEP diagram are shown in Supplymentary Figure 101.

Crystallographic Structure Determination of 10: The single crystal, which was obtained by the procedure described below, was mounted on MicroMesh. Data of X-ray diffraction were collected at 123 K on a Rigaku FR-X with Pilatus diffractometer with fine-focus sealed tube Mo/Kα radiation (λ = 0.71075 Å). An absorption correction was made using Crystal Clear. The structure was solved by direct methods and Fourier syntheses, and refined by full-matrix least squares on F^2 by using SHELXL-2014.[11] All non-hydrogen atoms were refined with anisotropic displacement parameters. Hydrogen atoms bonded to nitrogen atoms were located from a difference synthesis and their coordinates and isotropic thermal parameters refined. The other hydrogen atoms were placed in calculated positions and isotropic thermal parameters refined.

Recrystallization of 10: Recrystallization was performed by using a H/CH_2Cl_2 solvent system at room temperature to afford single crystals of **10**. The crystallographic data and ORTEP diagram are shown in Supplymentary Figure 102.

Crystallographic Structure Determination of 14: The single crystal, which was obtained by the procedure described below, was mounted on MicroMesh. Data of X-ray diffraction were collected at 123 K on a Rigaku FR-X with Pilatus diffractometer with fine-focus sealed tube Mo/Kα radiation (λ = 0.71075 Å). An absorption correction was made using Crystal Clear. The structure was solved by direct methods and Fourier syntheses, and refined by full-matrix least squares on F^2 by using SHELXL-2014.[11] All non-hydrogen atoms were refined with anisotropic displacement parameters. Hydrogen atoms bonded to nitrogen atoms were located from a difference synthesis and their coordinates and isotropic thermal parameters refined. The other hydrogen atoms were placed in calculated positions and isotropic thermal parameters refined.

Recrystallization of 14: Recrystallization was performed by using a H/EA solvent system at room temperature to afford single crystals of **14**. The crystallographic data and ORTEP diagram are shown in Supplymentary Figure 103.

Crystallographic Structure Determination of 16: The single crystal, which was obtained by the procedure described below, was mounted on MicroMesh. Data of X-ray diffraction were collected at 123 K on a Rigaku FR-X with Pilatus diffractometer with fine-focus sealed tube Mo/Kα radiation (λ = 0.71075 Å). An absorption correction was made using Crystal Clear. The structure was solved by direct methods and Fourier syntheses, and refined by full-matrix least squares on F^2 by using SHELXL-2014.[11] All non-hydrogen atoms were refined with anisotropic displacement parameters. Hydrogen atoms bonded to nitrogen atoms were located from a difference synthesis and their coordinates and isotropic thermal parameters refined. The other hydrogen atoms were placed in calculated positions and isotropic thermal parameters refined.

Recrystallization of 16: Recrystallization was performed by using a H/benzene solvent system at room temperature to afford single crystals of **16**. The crystallographic data and ORTEP diagram are shown in Supplymentary Figure 104.

Crystallographic Structure Determination of *RR*-6ac: The single crystal, which was obtained by the procedure described below, was mounted on MicroMesh. Data of X-ray diffraction were collected at 123 K on a Rigaku MicroMax-007HF with R-AXIS RAPID II with fine-focus sealed tube Cu/Kα radiation (λ = 1.54187 Å). An absorption correction was made using RAPID-AUTO. The structure was solved by direct methods and Fourier syntheses, and refined by full-matrix least squares on F^2 by using SHELXL-2014.[11] All non-hydrogen atoms were refined with anisotropic displacement parameters. Hydrogen atoms bonded to nitrogen atoms were located from a difference synthesis and their coordinates and isotropic thermal parameters refined. The other hydrogen atoms were placed in calculated positions and isotropic thermal parameters refined.

Recrystallization of *RR*-6ac: Recrystallization was performed by using a H/$CHCl_3$ solvent system at room temperature to afford single crystals of *RR*-**6ac**. The crystallographic data and ORTEP diagram are shown in Supplymentary Figure 105.

Crystallographic Structure Determination of 1aa·HCl: The single crystal, which was obtained by the procedure described below, was mounted on MicroMesh. Data of X-ray diffraction were collected at 123 K on a Rigaku FR-X with Pilatus diffractometer with fine-focus sealed tube Mo/Kα radiation (λ = 0.71075 Å). An absorption correction was made using Crystal Clear. The structure was solved by direct methods and Fourier syntheses, and refined by full-matrix least squares on F^2 by using SHELXL-2014.[11] All non-hydrogen atoms were refined with anisotropic displacement parameters. Hydrogen atoms bonded to nitrogen and oxygen atoms were located from a difference synthesis and their coordinates and isotropic thermal parameters refined. The other hydrogen atoms were placed in calculated positions and isotropic thermal parameters refined. SQUEEZE/PLATON was used in structural refinement.

Recrystallization of 1aa·HCl: Recrystallization was performed by using a H/EA solvent system at room temperature to afford single crystals of **1aa**·HCl. The crystallographic data and ORTEP diagram are shown in Supplymentary Figure 106.

Crystallographic Structure Determination of 1ba·HCl: The single crystal, which was obtained by the procedure described below, was mounted on MicroMesh. Data of X-ray diffraction were collected at 93 K on a Bruker D8 QUEST with CCD diffractometer with graphite-monochromated Mo/Kα radiation (λ = 0.71073 Å). An absorption correction was made using SADABS. The structure was solved by direct methods and Fourier syntheses, and refined by full-matrix least squares on F^2 by using SHELXL-2014.[11] All non-hydrogen atoms were refined with anisotropic displacement parameters. Hydrogen atoms bonded to nitrogen atoms were located from a difference synthesis and their coordinates and isotropic thermal parameters refined. The other hydrogen atoms were placed in calculated positions and isotropic thermal parameters refined.

Recrystallization of 1ba·HCl: Recrystallization was performed by using a H/acetone solvent system at room temperature to afford single crystals of **1ba**·HCl. The crystallographic data and ORTEP diagram are shown in Supplymentary Figure 107.

Crystallographic Structure Determination of 1da·HCl: The single crystal, which was obtained by the procedure described below, was mounted on MicroMesh. Data of X-ray diffraction were collected at 123 K on a Rigaku FR-X

with Pilatus diffractometer with fine-focus sealed tube Mo/Kα radiation (λ = 0.71075 Å). An absorption correction was made using Crystal Clear. The structure was solved by direct methods and Fourier syntheses, and refined by full-matrix least squares on F^2 by using SHELXL-2014.[11] All non-hydrogen atoms were refined with anisotropic displacement parameters. Hydrogen atoms bonded to nitrogen atoms were located from a difference synthesis and their coordinates and isotropic thermal parameters refined. The other hydrogen atoms were placed in calculated positions and isotropic thermal parameters refined. Hydrogen atoms bonded to O4 and O5 which are likely H_2O molecules could not be assigned.

Recrystallization of 1da·HCl: Recrystallization was performed by using a H/acetone solvent system at room temperature to afford single crystals of **1da**·HCl. The crystallographic data and ORTEP diagram are shown in Supplymentary Figure 108.

Crystallographic Structure Determination of *ent*-1ea·HCl: The single crystal, which was obtained by the procedure described below, was mounted on MicroMesh. Data of X-ray diffraction were collected at 123 K on a Rigaku FR-X with Pilatus diffractometer with fine-focus sealed tube Mo/Kα radiation (λ = 0.71075 Å). An absorption correction was made using Crystal Clear. The structure was solved by direct methods and Fourier syntheses, and refined by full-matrix least squares on F^2 by using SHELXL-2014.[11] All non-hydrogen atoms were refined with anisotropic displacement parameters. Hydrogen atoms bonded to nitrogen atoms were located from a difference synthesis and their coordinates and isotropic thermal parameters refined. The other hydrogen atoms were placed in calculated positions and isotropic thermal parameters refined.

Recrystallization of *ent*-1ea·HCl: Recrystallization was performed by using a H/acetone solvent system at room temperature to afford single crystals of *ent*-**1ea**·HCl. The crystallographic data and ORTEP diagram are shown in Supplymentary Figure 109.

Supplementary References:

(1) **Procedure for preparation of water-saturated solvent:** An organic solvent was shaken with distilled water and partitioning of two phases was performed to afford a water-saturated organic solvent.
(2) Uraguchi, D., Ueki, Y. & Ooi, T. *Science* **326**, 120–123 (2009).
(3) Uraguchi, D., Ueki, Y. & Ooi, T. *Angew. Chem. Int. Ed.* **50**, 3681–3683 (2011).
(4) Uraguchi, D., Yoshioka, K., Ueki, Y. & Ooi, T. *J. Am. Chem. Soc.* **134**, 19370–19373 (2012).
(5) Ruble, J. C. & Fu, G. C. *J. Am. Chem. Soc.* **120**, 11532–11533 (1998).
(6) Yamagiwa, N., Qin, H., Matsunaga, S. & Shibasaki, M. *J. Am. Chem. Soc.* **127**, 13419–13427 (2005).
(7) Yang, X.-Y., Tay, W. W., Li, Y., Pullarkat, S. A. & Leung, P.-H. *Organometallics* **34**, 5196–5201 (2015).
(8) Prevost, M. S., Delarce-Cochin, S., Marteaux, J., Colas, C., Renterghem, C. V., Blondel, A., Malliavin, T., Corringer, P.-J. & Joseph, D. *J. Med. Chem.* **56**, 4619–4630 (2013).
(9) Kim, T., Al-Muhanna, M. K., Al-Suwaidan, S. D., Al-Kaysi, R. O. & Bardeen, C. J. *Angew. Chem. Int. Ed.* **52**, 6889–6893 (2013).
(10) Vakulya, B., Varga, S. & Soós, T. *J. Org. Chem.* **73**, 3475–3480 (2008).
(11) Sheldrick, G. M. *Acta Cryst.* **C71**, 3–8 (2015).
