# Supplementary material for: Complete diastereodivergence in asymmetric 1,6-addition reactions enabled by minimal modification of a chiral catalyst
Source: Nat Commun. 2017 Mar 20;8:14793. doi: 10.1038/ncomms14793 (PMC5364390; doi:10.1038/ncomms14793)
Supplement: Supplementary Data 1 — Cartesian coordinates of DFT calculations for transition state models. [file ncomms14793-s2.docx]

**Supplementary Data 1.**

**Computational Details:**

All calculations were performed with the Gaussian 09 package.^1^ Typical local minima and TS structures were optimized at B3LYP/6-31G* level and frequency calculation was carried out to characterize the stationary points and to estimate Gibbs free energies. Single point calculations with the polarized continuum model (PCM, toluene and DCE) were performed at B3LYP-D3/6-311++G** level. To evaluate more reliable relative Gibbs energies including dispersion corrections, zero-point energy and thermal and entropic corrections calculated at B3LYP/6-31G* level at –30 °C and 1 atm were added to the electronic energy calculated at B3LYP-D3/6-311++G**. The molecular structures were depicted by using the CYLview v1.0.561 β.^2^

**Data for DFT Calculations:**

- **1aa**-*SS*


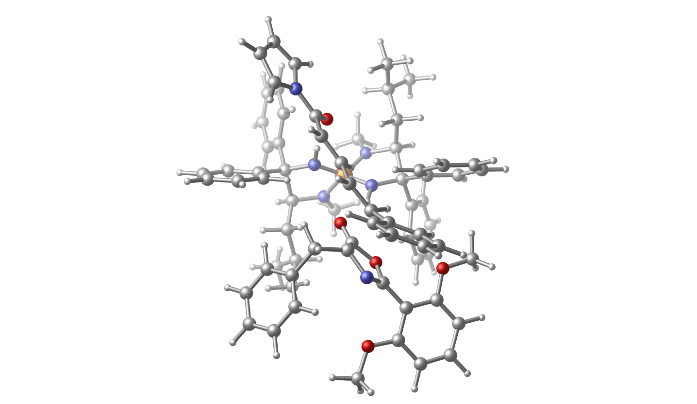

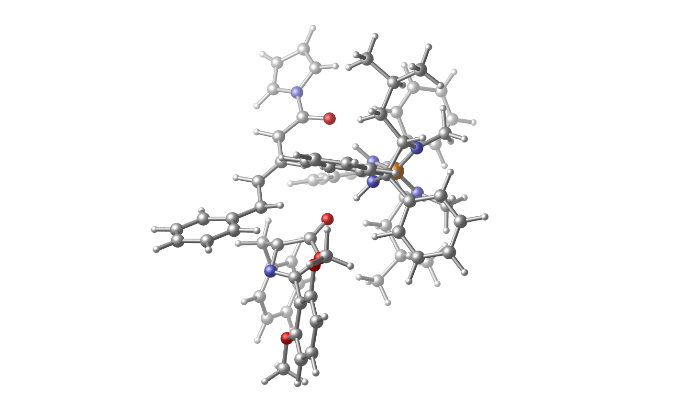


B3LYP/6-31G*

E = –3797.857997

Zero-point correction = 1.340415 (Hartree/Particle)

Thermal correction to Energy = 1.419315

Thermal correction to Enthalpy = 1.420259

Thermal correction to Gibbs Free Energy = 1.216443

Sum of electronic and zero-point Energies = –3796.517581

Sum of electronic and thermal Energies = –3796.438681

Sum of electronic and thermal Enthalpies = –3796.437737

Sum of electronic and thermal Free Energies = –3796.641554

PCM(toluene)-B3LYP-D3/6-311++G**//B3LYP/6-31G*@–30 °C

E = –3799.05151024

G = –3797.799957

PCM(DCE)-B3LYP-D3/6-311++G**//B3LYP/6-31G*@–30 °C

E = –3799.06559931

G = –3797.814046

0 1

O 0.79071100 -0.67978200 -0.23356600

C 2.00424500 -0.74667600 -0.05296900

C 2.84849800 -1.61214400 0.76919800

O 2.84423100 0.23031700 -0.57055900

N 4.18848800 -1.21062300 0.52780600

C 4.13431700 -0.15548100 -0.19830000

C 5.23914700 0.66310100 -0.72316900

C 5.29375200 2.04678300 -0.45941600

C 6.25219400 0.07936100 -1.51368100

C 6.33198700 2.83458500 -0.97180000

C 7.29830800 0.86413200 -2.01969400

C 7.32131000 2.22837800 -1.74298600

O 4.29473700 2.53365800 0.32954100

C 4.25770600 3.92974800 0.59612600

H 5.15675800 4.25887600 1.13313900

H 3.38014800 4.08830600 1.22253700

H 4.15473300 4.50868600 -0.33035700

O 6.12131400 -1.24845800 -1.76130000

C 7.06910100 -1.88528700 -2.60258900

H 8.07525700 -1.86566800 -2.16389900

H 7.09894100 -1.42473400 -3.59885800

H 6.72950800 -2.91817000 -2.69138400

H 6.37991200 3.89663900 -0.76707100

H 8.08013300 0.42286400 -2.62517100

H 8.13217300 2.83536600 -2.13713300

C 2.58381100 -3.09826700 0.89554100

H 1.53732200 -3.23113200 1.18595700

H 3.20201300 -3.47029400 1.72168800

C 2.88484800 -3.90713100 -0.35478600

C 4.20784300 -4.21359600 -0.70810500

C 1.84908800 -4.38558500 -1.16717700

C 4.48313500 -4.98654200 -1.83614700

C 2.12324700 -5.15669200 -2.29970700

C 3.44179200 -5.46201100 -2.63756800

H 5.01929100 -3.83329400 -0.09444100

H 0.81752700 -4.15890800 -0.90860000

H 5.51396900 -5.23081100 -2.08407300

H 1.30302000 -5.52671000 -2.91020700

H 3.65699900 -6.06994700 -3.51282200

C 2.30582800 -0.86020400 2.58477000

C 1.15070700 -1.54395100 3.10275500

H 2.10583000 0.14436900 2.21537900

H 1.32082100 -2.36340800 3.79855400

C -0.16402700 -1.23836600 2.79713700

H -0.35668800 -0.44227000 2.08318300

C -1.30026400 -1.86392600 3.33802000

H -1.15812000 -2.64907600 4.07276700

C -2.60712300 -1.45090200 3.02223200

O -2.93946200 -0.51162400 2.24158700

N -3.69247600 -2.14469300 3.66022700

C -3.67479500 -3.37374600 4.30380200

C -4.98191300 -1.64328000 3.71562200

C -4.94059200 -3.64301000 4.76179200

C -5.77092200 -2.53923700 4.39373400

H -2.77707500 -3.96958000 4.34121600

H -5.19680300 -0.67952100 3.28389400

H -5.24247500 -4.53679200 5.29228500

H -6.82477300 -2.41910300 4.60859400

H -0.09238700 1.03265100 -0.24344200

N -0.91277600 1.64814000 -0.26526300

P -2.25665200 1.12869400 -1.06399500

N -3.07768700 2.55065100 -0.82968000

C -2.39952400 3.42375300 0.16015700

C -0.86465900 3.11703300 -0.08041700

C -4.34071000 2.92867200 -1.45180200

H -4.63453300 2.16198500 -2.17054900

H -5.14739400 3.02563800 -0.72094400

H -4.23062100 3.88336600 -1.98310300

H -2.61836900 4.46070000 -0.10568500

C -2.84217900 3.15857900 1.61459800

H -2.53087400 2.14593900 1.89631000

H -2.29062700 3.84892700 2.26431400

C -4.34392200 3.30233000 1.93421900

C -4.58942700 2.84749700 3.38198600

C -4.86874200 4.73006200 1.71668900

H -4.90534100 2.61498400 1.28747900

C -0.01578900 3.52105100 1.13330300

C 0.35689800 2.60123000 2.11874400

C 0.34303300 4.86707700 1.30599400

C 1.07072700 3.00962000 3.24802300

C 1.04125600 5.28005000 2.44069700

C 1.41198300 4.35133700 3.41681900

H 0.06551100 1.56328500 2.02439400

H 0.07754600 5.59947400 0.54923200

H 1.34826900 2.27212500 3.99589900

H 1.29937500 6.32942900 2.55856100

H 1.95934400 4.67165100 4.29918200

C -0.28461900 3.78309000 -1.35433000

C 0.92933100 3.28359300 -1.85881700

C -0.87740500 4.86369100 -2.01852300

C 1.51916800 3.83961200 -2.99184300

C -0.28684200 5.42212800 -3.15818800

C 0.91173400 4.91224100 -3.65057700

H 1.42804500 2.45469600 -1.36348600

H -1.80831600 5.29267200 -1.66206100

H 2.45704800 3.43143200 -3.35887900

H -0.77149400 6.25847700 -3.65541400

H 1.37067000 5.34520500 -4.53528800

N -2.64509700 -0.35716300 -0.50660300

C -3.31916600 -1.20047700 -1.51843200

C -2.55301600 -0.76430000 -2.83386000

N -2.38013100 0.70393500 -2.66010000

H -2.73479600 -0.52256300 0.51518500

C -2.37659100 1.61612300 -3.79754600

H -1.53219400 1.43110000 -4.46651400

H -2.29898800 2.64439600 -3.43965800

H -3.30729500 1.51411600 -4.37144000

H -3.19536400 -0.91190000 -3.70464500

C -1.22443200 -1.51567800 -3.04644900

H -1.45951800 -2.58225300 -3.15058000

H -0.61868100 -1.41630300 -2.13928100

C -0.36880700 -1.09361200 -4.25934900

H -0.12695500 -0.02660900 -4.16064100

C 0.96646100 -1.85372600 -4.23135600

C -1.08309600 -1.31532000 -5.60199900

C -4.83192700 -0.85622900 -1.55413400

C -5.58246600 -0.72340000 -2.72947600

C -5.49801900 -0.70635900 -0.32528000

C -6.95099000 -0.43418500 -2.68301500

C -6.86169200 -0.42489200 -0.27818900

C -7.59643000 -0.28246600 -1.45825500

H -5.12002400 -0.84977800 -3.70338600

H -4.94418400 -0.81011300 0.60360500

H -7.50717400 -0.33281200 -3.61152200

H -7.35157400 -0.32299600 0.68653200

H -8.65976100 -0.06139800 -1.42130700

C -3.14089000 -2.69109700 -1.19010800

C -3.94235400 -3.64719800 -1.83172600

C -2.17728800 -3.13073700 -0.27359600

C -3.78112500 -5.00713000 -1.56985100

C -2.03058800 -4.49252100 0.00153100

C -2.82659400 -5.43635400 -0.64631800

H -4.71119800 -3.32993800 -2.52986600

H -1.52932800 -2.41865300 0.22389200

H -4.41412800 -5.72956500 -2.07855500

H -1.29067800 -4.80807900 0.73192100

H -2.71063500 -6.49475100 -0.42913800

H -0.43805300 -1.02727700 -6.44058400

H -1.34043200 -2.37462600 -5.73266200

H -2.01062400 -0.73708400 -5.69011900

H 1.60839900 -1.55032600 -5.06724400

H 1.51042800 -1.66645400 -3.29989900

H 0.80876000 -2.93683900 -4.30768000

H -5.93305200 4.79940800 1.97110600

H -4.33005100 5.44221100 2.35566500

H -4.75823000 5.06907400 0.67935900

H -5.65450500 2.90216600 3.63756700

H -4.25244500 1.81517500 3.52556800

H -4.04406200 3.48317400 4.09191900

C 3.57210400 -0.90313400 3.37289800

C 3.96357600 -2.02693000 4.12090500

C 4.41169500 0.22359200 3.39069400

C 5.14414200 -2.02059500 4.86163600

H 3.34276000 -2.91759400 4.12771700

C 5.58940800 0.23358800 4.13590700

H 4.13851300 1.09678900 2.80406300

C 5.96190500 -0.88903900 4.87659000

H 5.42435300 -2.90269800 5.43200400

H 6.21938900 1.11970400 4.13483700

H 6.88016800 -0.88382200 5.45815000

- **1aa**-*RR*


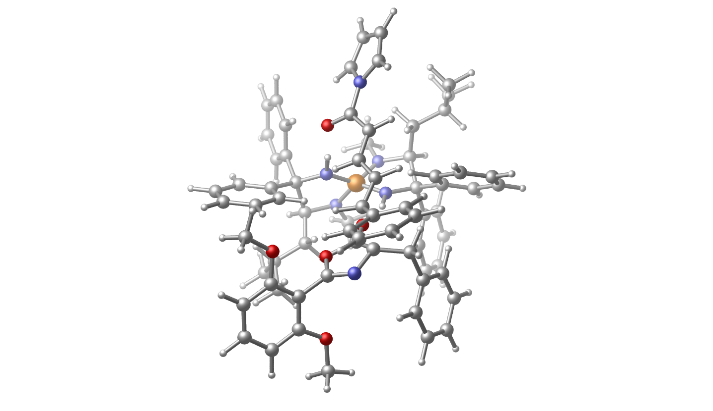

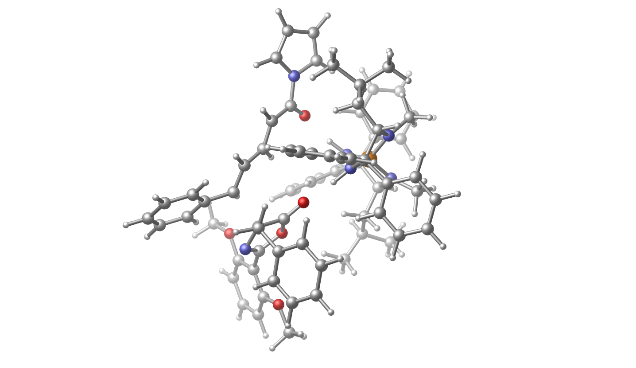


B3LYP/6-31G*

E = –3797.861773

Zero-point correction = 1.340230 (Hartree/Particle)

Thermal correction to Energy = 1.419195

Thermal correction to Enthalpy = 1.420139

Thermal correction to Gibbs Free Energy = 1.215217

Sum of electronic and zero-point Energies = –3796.521543

Sum of electronic and thermal Energies = –3796.442578

Sum of electronic and thermal Enthalpies = –3796.441634

Sum of electronic and thermal Free Energies = –3796.646556

PCM(toluene)-B3LYP-D3/6-311++G**//B3LYP/6-31G*@–30 °C

E = –3799.05592746

G = –3797.805396

PCM(DCE)-B3LYP-D3/6-311++G**//B3LYP/6-31G*@–30 °C

E = –3799.069885

G = –3797.819354

0 1

O 0.89790500 -0.46198400 0.24866200

C 2.10742900 -0.51048800 0.06295400

C 3.01107800 -1.56132200 -0.37787500

O 2.88815700 0.64417600 0.16199500

N 4.32747000 -1.03576700 -0.27874800

C 4.19632900 0.21420700 -0.02365300

C 5.25985000 1.22848000 0.12907900

C 5.62293700 2.06344900 -0.94107400

C 5.93050400 1.35980300 1.35922800

C 6.64605000 3.00866900 -0.79459800

C 6.95515800 2.30309100 1.51479800

C 7.29634400 3.11214400 0.43358300

O 4.91970300 1.87769900 -2.09931400

C 5.27420600 2.65121300 -3.23932900

H 6.31605500 2.47387900 -3.53358000

H 5.12398900 3.72291100 -3.05832500

H 4.60895500 2.32146900 -4.03849000

O 5.50663100 0.52275900 2.34333500

C 6.14035600 0.58054600 3.61145100

H 7.21059200 0.34790000 3.53749400

H 6.01444600 1.56491200 4.08095400

H 5.64498900 -0.17829700 4.21932300

H 6.93511300 3.65390000 -1.61478900

H 7.47810900 2.40973100 2.45724100

H 8.09059500 3.84464000 0.55061700

C 2.76980700 -2.99822200 0.02835600

H 1.77516300 -3.29125200 -0.32587200

H 3.50219900 -3.62257800 -0.49584600

C 2.86549700 -3.24929200 1.52613600

C 1.75366300 -3.70408800 2.24649400

C 4.07045800 -3.04331100 2.21657500

C 1.83765600 -3.94896600 3.62020000

C 4.15618500 -3.28820200 3.58703100

C 3.04031400 -3.74186300 4.29598200

H 0.81546700 -3.87873100 1.72555800

H 4.93618100 -2.67808900 1.67192500

H 0.96168300 -4.30493500 4.15695000

H 5.10135000 -3.13306300 4.10285300

H 3.11111900 -3.93789500 5.36316600

C 2.64015100 -1.46494300 -2.40305300

C 1.28946400 -1.78465500 -2.75535000

H 2.87810700 -0.40201700 -2.44088800

H 1.06601000 -2.80640800 -3.05678800

C 0.26229800 -0.85776700 -2.83141900

H 0.47720900 0.17052700 -2.54412500

C -1.03098800 -1.10516500 -3.32074200

H -1.26900600 -2.09487400 -3.69698600

C -2.01501200 -0.09767500 -3.37687100

O -1.96241800 1.03913800 -2.83983100

N -3.21759300 -0.39118000 -4.11151200

C -4.39112700 0.32784900 -3.96619000

C -3.42031000 -1.37211000 -5.07044100

C -5.33451300 -0.20113600 -4.81352300

C -4.71465000 -1.27886600 -5.52043100

H -4.43215300 1.14647300 -3.26619100

H -2.61422500 -2.01822200 -5.37992300

H -6.35289800 0.14802000 -4.92506400

H -5.16459100 -1.90212200 -6.28231200

H -0.84345700 -0.81442600 0.79418400

N -1.84643700 -0.70136400 0.95621500

P -2.54142200 0.78838200 0.99274700

N -4.07638600 0.17201900 1.11511900

C -4.13001600 -1.29594900 0.91053400

C -2.72334900 -1.78278100 1.45180700

C -5.30664200 0.95033000 1.12955300

H -5.07098300 2.00563200 1.28148300

H -5.85796800 0.85641500 0.18614500

H -5.95588200 0.61373500 1.94788400

H -4.92350600 -1.67526000 1.55849700

C -4.45642700 -1.67480700 -0.55100100

H -5.01105400 -0.84450400 -1.00641800

H -3.52710500 -1.75210200 -1.12375500

C -5.29561600 -2.96157100 -0.72351800

H -4.87162400 -3.74253800 -0.07861300

C -6.76017300 -2.74506100 -0.30857700

C -5.21491100 -3.45897600 -2.17387300

C -2.29149500 -3.14500700 0.88573500

C -1.51272900 -3.24309400 -0.27522900

C -2.69546200 -4.32951400 1.52237500

C -1.15902800 -4.49448400 -0.78818700

C -2.34542000 -5.57635800 1.00573700

C -1.57378700 -5.66477600 -0.15422600

H -1.17683200 -2.35118800 -0.79188400

H -3.28100300 -4.28056200 2.43483500

H -0.55259700 -4.54372900 -1.68828300

H -2.67049300 -6.47855300 1.51703900

H -1.29412500 -6.63557300 -0.55426400

C -2.65767100 -1.82382300 3.00309800

C -1.39510100 -1.71710000 3.60984900

C -3.77076900 -1.99977200 3.83495600

C -1.25224200 -1.77037600 4.99483000

C -3.63174400 -2.04593000 5.22646800

C -2.37364700 -1.92911000 5.81310200

H -0.50932300 -1.59654500 2.99372500

H -4.76604600 -2.10966300 3.41676000

H -0.25996500 -1.69219600 5.43069000

H -4.51466900 -2.17672600 5.84680600

H -2.26570800 -1.96673800 6.89369400

N -1.86295200 1.70865600 -0.17493200

C -1.75684100 3.14618300 0.17956400

C -1.51784700 3.06768200 1.74022400

N -2.41946900 1.96585900 2.15595600

H -1.80687000 1.39920400 -1.16652000

C -2.82244800 1.84132400 3.54883300

H -3.51634000 1.00577900 3.65531100

H -1.96951900 1.66009500 4.21436300

H -3.32768400 2.76065200 3.87011000

H -1.88449500 3.98105700 2.21253400

C -0.07049500 2.80396600 2.21468600

H -0.14200500 2.27613000 3.17629000

H 0.41300200 2.10106600 1.52865800

C 0.83781400 4.03370800 2.43840500

H 0.84966000 4.63877400 1.52478400

C 0.34450800 4.92242800 3.59188500

C 2.27597100 3.56216300 2.70317000

C -0.57956000 3.79103600 -0.56600100

C 0.51893800 3.02691500 -0.97963000

C -0.56281400 5.17402500 -0.79949100

C 1.61527400 3.63278000 -1.59556200

C 0.53077100 5.77873400 -1.41919500

C 1.62532200 5.00980700 -1.81806700

H 0.52804200 1.95718700 -0.80660100

H -1.40985300 5.78381700 -0.50150400

H 2.46311000 3.01886800 -1.88519400

H 0.52273600 6.85175700 -1.59274800

H 2.47710500 5.48252700 -2.30067500

C -3.08052000 3.86190800 -0.19889200

C -3.80610000 4.69730400 0.65902800

C -3.56916200 3.67148300 -1.50471800

C -4.98994300 5.31361400 0.23606300

C -4.74459200 4.28863500 -1.92689500

C -5.46497400 5.11199200 -1.05700900

H -3.46675800 4.88958300 1.67147500

H -3.02841300 3.02912800 -2.19454800

H -5.53349600 5.95451200 0.92570700

H -5.09619300 4.12680800 -2.94259300

H -6.38222300 5.59260200 -1.38667300

H 2.95067900 4.41653600 2.83565500

H 2.32738800 2.95309600 3.61611100

H 2.65313900 2.95392500 1.87470900

H 1.01802600 5.77504900 3.73783600

H -0.65748100 5.33159500 3.41349400

H 0.31265500 4.36114000 4.53566400

H -7.33080500 -3.67748600 -0.39131100

H -6.85868100 -2.39483800 0.72701600

H -7.24244700 -2.00244400 -0.95753900

H -5.82256300 -4.36148300 -2.31320000

H -5.57275300 -2.69723200 -2.87686600

H -4.18262200 -3.70131800 -2.44835600

C 3.74391900 -2.30186600 -2.95866900

C 4.97374300 -1.70027600 -3.27661200

C 3.60214000 -3.67813500 -3.21076000

C 6.01385300 -2.43846300 -3.83786800

H 5.10832000 -0.64261400 -3.07283400

C 4.64455900 -4.41954100 -3.76536300

H 2.66937500 -4.17935700 -2.97163200

C 5.85530000 -3.80299600 -4.08586800

H 6.95281300 -1.94649700 -4.07947800

H 4.50785800 -5.48205900 -3.95058800

H 6.66588200 -4.38026100 -4.52302200

- **1aa**-*RS*


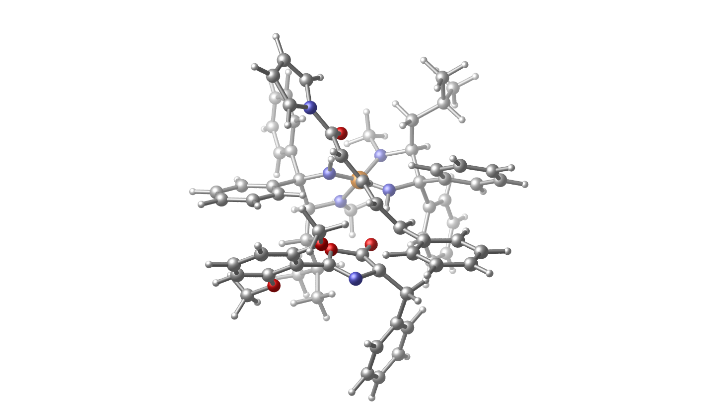

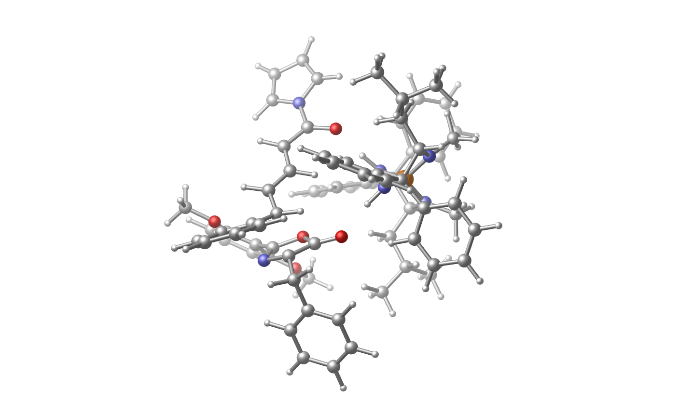


B3LYP/6-31G*

E = –3797.860355

Zero-point correction = 1.341186 (Hartree/Particle)

Thermal correction to Energy = 1.419764

Thermal correction to Enthalpy = 1.420708

Thermal correction to Gibbs Free Energy = 1.218293

Sum of electronic and zero-point Energies = –3796.519169

Sum of electronic and thermal Energies = –3796.440591

Sum of electronic and thermal Enthalpies = –3796.439647

Sum of electronic and thermal Free Energies = –3796.642062

PCM(toluene)-B3LYP-D3/6-311++G**//B3LYP/6-31G*@–30 °C

E = –3799.055861

G = –3797.802712

PCM(DCE)-B3LYP-D3/6-311++G**//B3LYP/6-31G*@–30 °C

E = –3799.069649

G = –3797.8165

0 1

O 0.89339200 -1.03301600 0.99063600

C 2.02835100 -0.64719700 0.69647400

C 3.22447100 -1.35276000 0.27195000

O 2.32713300 0.69362700 0.59429900

N 4.25094000 -0.39609800 0.13796300

C 3.68305000 0.75292000 0.25371800

C 4.29530300 2.08553600 0.13364900

C 5.05499800 2.44389200 -1.00391300

C 4.16052400 3.03243700 1.17536200

C 5.63803300 3.71506200 -1.10252100

C 4.73619700 4.30546700 1.07205500

C 5.46745400 4.62907500 -0.06722500

O 5.16480600 1.50684200 -1.98471800

C 5.87405400 1.83941700 -3.16858000

H 6.92793000 2.06216600 -2.95819200

H 5.41866700 2.69357700 -3.68598900

H 5.81251900 0.95589300 -3.80613300

O 3.47259800 2.61066800 2.26862400

C 3.26549100 3.52406000 3.33502000

H 4.21631600 3.85809600 3.77064700

H 2.68313000 4.39389600 3.00715300

H 2.69999600 2.97224100 4.08684000

H 6.21347200 3.99486500 -1.97556400

H 4.63017500 5.02940300 1.86984500

H 5.91911800 5.61423900 -0.14822500

C 3.55613800 -2.72002200 0.83055400

H 2.73377000 -3.40842900 0.60411900

H 4.44058900 -3.09442800 0.30553900

C 3.80856200 -2.72045000 2.33029400

C 2.92806000 -3.36668700 3.20661200

C 4.93190800 -2.07440500 2.86836000

C 3.16091800 -3.37382500 4.58408000

C 5.16614500 -2.07849700 4.24284500

C 4.28185900 -2.72923300 5.10742100

H 2.05432600 -3.87414800 2.80309700

H 5.61460300 -1.55897200 2.19849500

H 2.46795300 -3.88690800 5.24706300

H 6.04333700 -1.57404400 4.64085600

H 4.46736700 -2.73495700 6.17858000

C 2.59755500 -1.66928800 -1.69183100

C 2.36964900 -0.44338600 -2.39296000

H 1.69638800 -2.19279600 -1.37179400

H 3.17369200 -0.05184300 -3.00949400

C 1.15457900 0.22670700 -2.37741400

H 0.37112900 -0.17610300 -1.73789200

C 0.79746400 1.35798900 -3.11992700

H 1.52622000 1.80450300 -3.78755000

C -0.51856400 1.86802900 -3.09396100

O -1.49902700 1.39212500 -2.45845100

N -0.79341300 3.02584800 -3.89618600

C -2.07477000 3.40909100 -4.25423400

C 0.10313700 3.93742900 -4.43499800

C -1.99216000 4.54584000 -5.01910400

C -0.60730700 4.88577600 -5.12789600

H -2.91280300 2.80516000 -3.94648300

H 1.16021200 3.85893200 -4.23737600

H -2.82712500 5.07605500 -5.45824600

H -0.18497800 5.73682700 -5.64632900

H -0.81555500 -1.38420800 0.29315300

N -1.82178000 -1.38066800 0.09596600

P -2.75293800 -0.16573300 0.70431700

N -4.17360500 -0.87719000 0.22575700

C -3.96113500 -2.04186300 -0.66563200

C -2.57598400 -2.62787000 -0.16102500

C -5.50078700 -0.29072700 0.35085100

H -5.47575900 0.52521000 1.07512600

H -5.86389200 0.11778400 -0.59966900

H -6.21064300 -1.05034500 0.70105600

H -4.75698500 -2.75693400 -0.44692500

C -4.04869700 -1.63374600 -2.15289200

H -3.09919100 -1.18871300 -2.46613700

H -4.78476500 -0.82154800 -2.22372000

C -4.48483400 -2.73612800 -3.14426900

H -3.87040000 -3.62837100 -2.97551300

C -4.23798600 -2.26555200 -4.58533700

C -5.95898200 -3.13113700 -2.95601200

C -2.72790300 -3.42909400 1.16017600

C -3.86969300 -4.17342500 1.48817900

C -1.64949100 -3.45105700 2.06145800

C -3.94408800 -4.89661200 2.68265500

C -1.72150800 -4.17421900 3.25167300

C -2.87172400 -4.89779500 3.57217800

H -4.71969100 -4.21278000 0.81421900

H -0.74102300 -2.89871100 1.84021000

H -4.84542600 -5.45963400 2.91074400

H -0.87161500 -4.16999200 3.92906500

H -2.92858900 -5.45882200 4.50099700

C -1.85748000 -3.47615500 -1.22453400

C -1.05949900 -2.87058300 -2.20573000

C -2.01127900 -4.87014000 -1.26012000

C -0.44263200 -3.63341000 -3.19915300

C -1.40032000 -5.63226300 -2.25671400

C -0.61339900 -5.01795300 -3.23174700

H -0.92148100 -1.79614300 -2.20202000

H -2.60443600 -5.37106100 -0.50286600

H 0.17877500 -3.13702800 -3.93903900

H -1.53553100 -6.71066000 -2.26270800

H -0.13310800 -5.61191600 -4.00459800

N -2.09233200 1.25476100 0.23506100

C -2.35379500 2.36171600 1.18270100

C -2.27420300 1.59585600 2.56579000

N -2.95112400 0.30274300 2.28063800

H -1.80902100 1.40326100 -0.75185400

C -3.76861000 -0.37074800 3.28221900

H -4.55197000 0.30478100 3.65102700

H -3.17307200 -0.71259500 4.13288300

H -4.24494000 -1.24389800 2.83278800

H -2.87464200 2.11528700 3.31574400

C -0.83665900 1.42141100 3.09125500

H -0.42115800 2.42120400 3.26955200

H -0.22717900 0.96559800 2.30342000

C -0.67589400 0.58640300 4.37928000

H -1.10643100 -0.40814700 4.20055000

C -1.37672200 1.21779300 5.59300000

C 0.81633900 0.37273200 4.67558400

C -1.29640100 3.46789700 1.05034700

C -0.08673400 3.25752100 0.37859800

C -1.53188100 4.72299700 1.63366700

C 0.85678600 4.28246000 0.27905600

C -0.58270400 5.73980900 1.54820500

C 0.61508500 5.52557600 0.86238200

H 0.14074000 2.29419200 -0.06047800

H -2.47037200 4.91610700 2.14449200

H 1.78213900 4.09783000 -0.25724300

H -0.78855400 6.70507600 2.00383100

H 1.34863300 6.32305400 0.77631900

C -3.75641500 2.95975400 0.89445000

C -4.10320600 3.19424000 -0.44747600

C -4.68213500 3.31286100 1.88456600

C -5.33484800 3.75155800 -0.78372600

C -5.92266700 3.86626000 1.54750900

C -6.25511000 4.08676400 0.21310000

H -3.40160700 2.93602200 -1.23588700

H -4.45436400 3.17034200 2.93629600

H -5.57365900 3.92779000 -1.82919000

H -6.62378900 4.12627400 2.33649600

H -7.21691800 4.51922700 -0.04907000

H -4.54671700 -3.02991300 -5.30827600

H -4.80528100 -1.35126900 -4.80438200

H -3.17777900 -2.04903300 -4.75477700

H -6.24104900 -3.92612300 -3.65603600

H -6.17150100 -3.50051700 -1.94515300

H -6.62138700 -2.27528400 -3.14264100

H 0.95592300 -0.26563600 5.55607400

H 1.32539300 -0.10108200 3.83083800

H 1.31493300 1.32927500 4.87966000

H -1.22540800 0.60962500 6.49278200

H -0.96913800 2.21664500 5.79794000

H -2.45866200 1.32688200 5.45189000

C 3.65682600 -2.58017900 -2.20463500

C 3.36705600 -3.93820300 -2.41657500

C 4.95029600 -2.11822500 -2.50956600

C 4.32768600 -4.80482600 -2.93983300

H 2.37277700 -4.31208700 -2.18477100

C 5.90999600 -2.98693500 -3.02532900

H 5.20423400 -1.08515600 -2.29585300

C 5.60337100 -4.33237100 -3.24926000

H 4.07801900 -5.85040300 -3.10324300

H 6.90755500 -2.61460300 -3.24611700

H 6.35479600 -5.00602400 -3.65308100

- **1aa**-*SR*


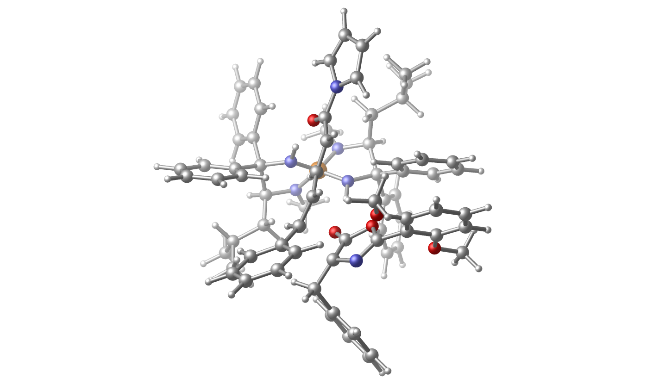

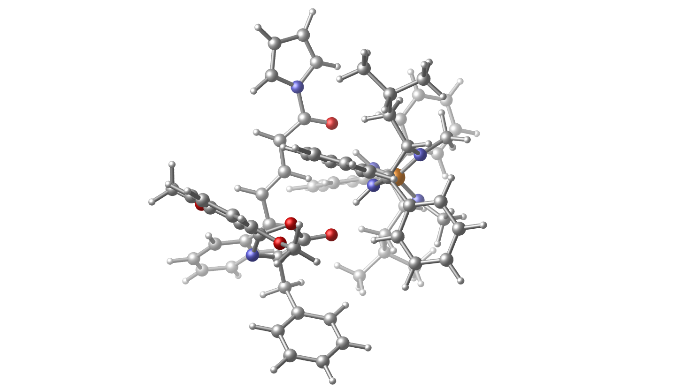


B3LYP/6-31G*

E = –3797.86084

Zero-point correction = 1.340082 (Hartree/Particle)

Thermal correction to Energy = 1.419075

Thermal correction to Enthalpy = 1.420019

Thermal correction to Gibbs Free Energy = 1.214593

Sum of electronic and zero-point Energies = –3796.520758

Sum of electronic and thermal Energies = –3796.441765

Sum of electronic and thermal Enthalpies = –3796.440821

Sum of electronic and thermal Free Energies = –3796.646248

PCM(toluene)-B3LYP-D3/6-311++G**//B3LYP/6-31G*@–30 °C

E = –3799.054043

G = –3797.804042

PCM(DCE)-B3LYP-D3/6-311++G**//B3LYP/6-31G*@–30 °C

E = –3799.067722

G = –3797.817721

0 1

O 0.98022800 0.75679900 1.17071700

C 2.12240100 0.62383100 0.71209800

C 3.14586900 1.58222900 0.35517600

O 2.59047700 -0.60069300 0.29982200

N 4.26924100 0.85542500 -0.08528100

C 3.88851400 -0.37153900 -0.15989500

C 4.66084700 -1.55144200 -0.57946900

C 4.84593900 -2.62107200 0.32489900

C 5.24924800 -1.63801100 -1.85865900

C 5.57568400 -3.75769400 -0.04523200

C 5.98671900 -2.77184000 -2.22819800

C 6.13595700 -3.81571500 -1.31886100

O 4.30030700 -2.44133400 1.55553300

C 4.42493600 -3.47452800 2.51947100

H 5.47717000 -3.67435700 2.76169500

H 3.94162000 -4.39804900 2.17910600

H 3.91181600 -3.10778500 3.40988000

O 5.03992600 -0.58273000 -2.69566000

C 5.59657300 -0.62997800 -4.00047100

H 5.20175100 -1.47571400 -4.57833500

H 5.30184100 0.30457600 -4.48083300

H 6.69190900 -0.69214000 -3.96944300

H 5.71988700 -4.57667500 0.64847500

H 6.43576000 -2.84712200 -3.21041300

H 6.70748000 -4.69353200 -1.60885000

C 3.34659700 2.85128500 1.15303800

H 2.40961000 3.42082300 1.16166000

H 4.09413700 3.46405200 0.64041400

C 3.78434500 2.58645400 2.58462200

C 2.85671900 2.60453300 3.63449000

C 5.12307800 2.29345200 2.88021200

C 3.25545200 2.34561700 4.94748200

C 5.52429700 2.03332600 4.19056200

C 4.59209200 2.06043100 5.23061500

H 1.81279100 2.81634100 3.41543200

H 5.84808200 2.26037500 2.07114600

H 2.52209200 2.37355500 5.75042900

H 6.56776900 1.81128300 4.40080100

H 4.90591200 1.86299700 6.25261500

C 2.20396600 2.20896200 -1.45518800

C 1.96534100 1.13058900 -2.35363700

H 1.32814600 2.55600700 -0.90619900

H 2.71160300 0.91643400 -3.11377400

C 0.77540000 0.41181100 -2.35876500

H 0.05598300 0.65300400 -1.57811100

C 0.37686600 -0.56311600 -3.27693800

H 1.06827400 -0.86451800 -4.05602000

C -0.94023500 -1.08211100 -3.28831700

O -1.90242700 -0.73006700 -2.55864000

N -1.23056000 -2.11166600 -4.24479400

C -2.51985900 -2.48671900 -4.58359100

C -0.34201000 -2.89644900 -4.96858100

C -2.45134600 -3.48962400 -5.51770100

C -1.06605500 -3.75421300 -5.75775500

H -3.35527800 -1.98335000 -4.12584900

H 0.72309600 -2.80261200 -4.83031900

H -3.29604000 -3.98012800 -5.98341800

H -0.65184500 -4.49605600 -6.42814400

H -0.36254900 -0.45928400 1.04688000

N -1.26111800 -0.96940600 1.05021400

P -2.67988800 -0.14861400 0.95968500

N -3.60516000 -1.49056300 1.26414100

C -2.82454400 -2.74686300 1.17205400

C -1.38802800 -2.30895300 1.67272200

C -5.05966500 -1.54340400 1.29580600

H -5.38737800 -2.14877400 2.15004500

H -5.47726500 -1.97618700 0.37873400

H -5.46160500 -0.53484000 1.40661800

H -3.26174500 -3.44689700 1.88748500

C -2.90528200 -3.36498000 -0.24265000

H -3.87155500 -3.07258300 -0.67521000

H -2.15178500 -2.90362400 -0.88708100

C -2.81215800 -4.90610500 -0.31199300

H -1.97170300 -5.23721700 0.30994100

C -2.52792600 -5.35257400 -1.75366500

C -4.08937600 -5.57823900 0.21855300

C -0.28192300 -3.25025600 1.17020400

C 0.04879100 -4.40120000 1.90256600

C 0.38305600 -3.01434000 -0.03905300

C 0.99941200 -5.30320000 1.42521000

C 1.34326100 -3.91132800 -0.51058400

C 1.65106800 -5.06227900 0.21348900

H -0.43623400 -4.59739900 2.85371700

H 0.16266300 -2.12667800 -0.61987300

H 1.23122200 -6.19333800 2.00494200

H 1.85253900 -3.69687300 -1.44525700

H 2.39671200 -5.76000300 -0.15825000

C -1.30159400 -2.16848700 3.21648900

C -0.30485600 -1.33003900 3.74669200

C -2.12897800 -2.85144100 4.11757900

C -0.15098700 -1.17470500 5.12287700

C -1.97892200 -2.69275200 5.49972700

C -0.99062600 -1.85367500 6.00914700

H 0.35633500 -0.78377500 3.08066000

H -2.90342100 -3.52386000 3.76313000

H 0.63079500 -0.51983500 5.49889100

H -2.63974200 -3.23233800 6.17342700

H -0.87309500 -1.73166500 7.08259800

N -2.62507700 0.81499600 -0.37447400

C -3.48252900 2.01506900 -0.25277800

C -3.25115000 2.38183600 1.26786900

N -3.27801600 1.05663400 1.93143000

H -2.33846600 0.40081500 -1.27875500

C -3.51804600 0.96190500 3.36363200

H -2.74862900 1.48604700 3.94419900

H -3.51468300 -0.08689400 3.66623100

H -4.49608200 1.39518700 3.60770500

H -4.10514800 2.94730300 1.64734800

C -1.94632100 3.14850000 1.58479700

H -1.17254200 2.84801800 0.87078400

H -1.58728400 2.80061400 2.56275500

C -2.05185000 4.68848200 1.64674300

H -2.54808000 5.04551300 0.73630100

C -2.87407800 5.16285600 2.85642100

C -0.64651500 5.30696600 1.68359000

C -3.04205500 3.13065000 -1.21488900

C -3.89849000 4.21385200 -1.47032800

C -1.79371400 3.11232200 -1.84603600

C -3.50859800 5.25140400 -2.31588700

C -1.40470100 4.14641600 -2.70151500

C -2.25785400 5.22294100 -2.93646400

H -4.88386900 4.24529700 -1.01651700

H -1.11869800 2.28304400 -1.68661700

H -4.19020200 6.07836900 -2.49710700

H -0.43216500 4.09688900 -3.18346700

H -1.95795500 6.02665000 -3.60341000

C -4.95612200 1.63492400 -0.56280600

C -6.05929000 2.14117000 0.13699100

C -5.20120600 0.76365300 -1.63765400

C -7.36425700 1.78008800 -0.21376600

C -6.50141800 0.40777900 -1.99210300

C -7.59176200 0.91178900 -1.27935000

H -5.92422000 2.82919700 0.96576300

H -4.36726700 0.35143900 -2.19833300

H -8.20041200 2.18414700 0.35111300

H -6.66126000 -0.26559200 -2.83007200

H -8.60533900 0.63243300 -1.55374000

H -0.69637400 6.40121600 1.73038700

H -0.08546100 4.96202500 2.56231200

H -0.07094400 5.03456900 0.79168500

H -2.95432300 6.25602500 2.86869800

H -3.89604600 4.76384800 2.85477200

H -2.39928900 4.85505000 3.79761500

H -3.99178300 -6.67010100 0.20392700

H -4.32210000 -5.28754700 1.25115100

H -4.95577900 -5.31494300 -0.40284600

H -2.48280000 -6.44615100 -1.82502900

H -3.30931300 -5.00382000 -2.44058900

H -1.57482400 -4.95034200 -2.11203300

C 3.11548100 3.30213200 -1.88027800

C 2.69628400 4.63878300 -1.77177700

C 4.38638000 3.04358400 -2.42543800

C 3.50700200 5.68564500 -2.21251000

H 1.71618200 4.85659900 -1.35266200

C 5.19739800 4.09065700 -2.85764900

H 4.74537400 2.02043400 -2.46310300

C 4.76146600 5.41543100 -2.75995800

H 3.15767700 6.71150200 -2.12547600

H 6.18109500 3.87311700 -3.26676500

H 5.39765500 6.22836000 -3.10026400

- **1da**-*RR* (HH)


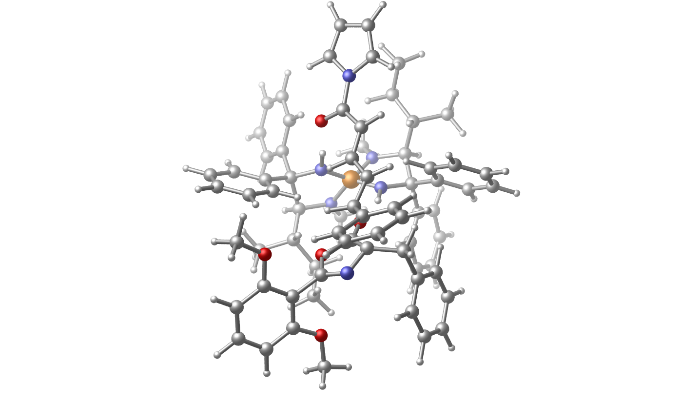

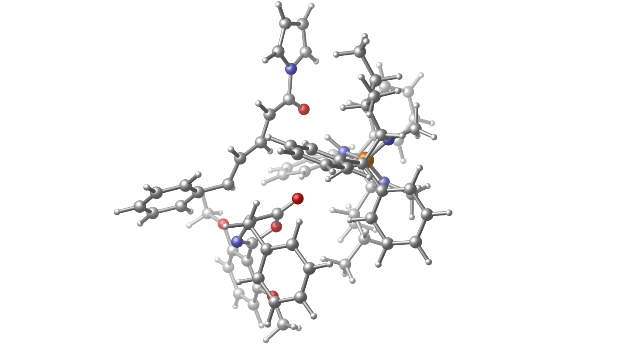


B3LYP/6-31G*

E = –3797.844244

Zero-point correction = 1.342046 (Hartree/Particle)

Thermal correction to Energy = 1.420406

Thermal correction to Enthalpy = 1.421350

Thermal correction to Gibbs Free Energy = 1.221185

Sum of electronic and zero-point Energies = –3796.502198

Sum of electronic and thermal Energies = –3796.423839

Sum of electronic and thermal Enthalpies = –3796.422894

Sum of electronic and thermal Free Energies = –3796.623059

PCM(toluene)-B3LYP-D3/6-311++G**//B3LYP/6-31G*@–30 °C

E = –3799.041782

G = –3797.786157

0 1

O -0.91309700 -0.59385400 -0.07886400

C -2.11204200 -0.41845800 0.09360400

C -3.12838800 -1.13453100 0.84528100

O -2.73865100 0.72905600 -0.39772900

N -4.36974400 -0.50610700 0.55314100

C -4.08662400 0.55910900 -0.10188900

C -5.00960200 1.58848200 -0.62265600

C -5.31476100 2.74042700 0.12101700

C -5.59554400 1.41490800 -1.88989700

C -6.20606700 3.69749800 -0.38137300

C -6.48873000 2.36603600 -2.39999100

C -6.78003000 3.49299800 -1.63459200

O -4.69277800 2.84449000 1.33421300

C -5.00204200 3.96056300 2.15990100

H -6.07069300 3.99165300 2.40605400

H -4.71040000 4.90457100 1.68261300

H -4.42284200 3.82137000 3.07389000

O -5.22369500 0.28348700 -2.54763600

C -5.82016700 -0.00799200 -3.80191900

H -6.91141900 -0.08957000 -3.71717700

H -5.56965200 0.75108400 -4.55429800

H -5.40621700 -0.97120500 -4.10415600

H -6.45085500 4.58721000 0.18506400

H -6.94700300 2.23787100 -3.37299200

H -7.47165600 4.23439900 -2.02583700

C -3.07892100 -2.64469400 0.93325800

H -2.10815600 -2.92907900 1.35516500

H -3.85402500 -2.97008100 1.63572400

C -3.27459900 -3.33799300 -0.40580000

C -2.21651800 -4.01567000 -1.02479300

C -4.51891800 -3.30619500 -1.05435600

C -2.39270600 -4.64775100 -2.25883200

C -4.69728600 -3.93716700 -2.28542400

C -3.63446900 -4.61105600 -2.89380900

H -1.24757100 -4.05473600 -0.53283400

H -5.34276700 -2.77148000 -0.59040200

H -1.55754500 -5.17034500 -2.71872300

H -5.67187600 -3.91011900 -2.76781800

H -3.77628500 -5.10816300 -3.85046100

C -2.69086500 -0.42397900 2.72121100

C -1.37648200 -0.75900200 3.17970500

H -2.80904500 0.61091500 2.40036900

H -1.26560900 -1.62452300 3.82992200

C -0.24885000 0.01329800 2.94863200

H -0.34333100 0.87718200 2.29322000

C 0.99871300 -0.15602900 3.57065800

H 1.08657600 -0.91486600 4.34042300

C 2.06503400 0.74220600 3.35615500

O 2.13894600 1.61847400 2.45657300

N 3.17527100 0.69590900 4.27469100

C 4.17396000 1.65499000 4.28460600

C 3.41952400 -0.19612800 5.31115600

C 5.04180300 1.37495300 5.31079800

C 4.56480700 0.19437100 5.96014500

H 4.13860200 2.46247200 3.57233200

H 2.76792100 -1.03417500 5.49763000

H 5.91707700 1.95355800 5.57573500

H 5.01232300 -0.31013100 6.80676900

H 0.77882200 -1.14940000 -0.41260300

N 1.77325700 -1.15828700 -0.65025500

P 2.52074900 0.19510700 -1.19396200

N 4.01505200 -0.52737500 -1.21524100

C 4.02247900 -1.91816200 -0.68653500

C 2.51742100 -2.40496700 -0.91918100

C 5.20957900 0.03244100 -1.84056000

H 4.98225400 1.01999600 -2.24511100

H 6.02200800 0.15035900 -1.12150700

H 5.55446500 -0.61346600 -2.65933400

H 4.69089500 -2.47850700 -1.34555300

C 4.56621700 -2.08776900 0.76755500

H 3.72523400 -1.98083600 1.46236300

C 5.60887000 -1.02491600 1.17578900

H 6.48947700 -1.10079500 0.52120400

C 6.07114400 -1.14061100 2.63364300

C 2.01292000 -3.50370900 0.03786100

C 1.44944000 -3.17288400 1.27960000

C 2.08858800 -4.86042100 -0.31626800

C 0.99540200 -4.17124300 2.14460400

C 1.63818700 -5.85579800 0.55096300

C 1.08888400 -5.51575900 1.78785500

H 1.35410900 -2.13592100 1.58363700

H 2.49588100 -5.14820400 -1.27960000

H 0.56218600 -3.88534900 3.09909000

H 1.70875800 -6.89801800 0.25117400

H 0.72982900 -6.28993800 2.46052500

C 2.27811800 -2.85211800 -2.38817300

C 0.99437800 -2.69164100 -2.93568000

C 3.25381600 -3.47083000 -3.18228200

C 0.70281700 -3.11518000 -4.23169800

C 2.96669900 -3.88873400 -4.48593800

C 1.69158000 -3.71033500 -5.01826700

H 0.20729800 -2.23755700 -2.34207200

H 4.25125000 -3.65391500 -2.79525600

H -0.30322400 -2.98301100 -4.62009400

H 3.74622700 -4.36006700 -5.07896500

H 1.46800700 -4.03695000 -6.03021600

N 2.00240400 1.46640000 -0.31037800

C 2.01471900 2.75156300 -1.05134600

C 1.61226400 2.27458600 -2.52346200

N 2.29976900 0.96393500 -2.64656300

H 1.95827700 1.45631500 0.72752900

C 2.83866000 0.53848600 -3.93386200

H 3.33181000 -0.42841700 -3.82559500

H 2.04870000 0.42523500 -4.67881600

H 3.56966400 1.26957200 -4.30509900

H 2.08785600 2.93117600 -3.25670300

C 0.08269700 2.25344400 -2.84686200

H -0.46101600 2.00809800 -1.92964400

C -0.32072600 1.18104400 -3.88328900

H 0.12987200 1.41653400 -4.85921400

C -1.83821700 1.03407100 -4.06115800

C 1.01673700 3.71545700 -0.38324600

C -0.14346800 3.22219600 0.23119100

C 1.23036400 5.10146800 -0.38380100

C -1.07667600 4.08887000 0.79961700

C 0.30125500 5.96859100 0.19313400

C -0.85842800 5.46653000 0.78357600

H -0.32415000 2.15433700 0.26197100

H 2.12905200 5.51310300 -0.83011100

H -1.97666600 3.67505500 1.24568500

H 0.49136100 7.03874400 0.18295400

H -1.58076400 6.14360300 1.23245700

C 3.44601400 3.35298200 -1.01948900

C 4.00758200 4.08254400 -2.07685200

C 4.20508100 3.20575800 0.15420600

C 5.29612800 4.61840600 -1.98087400

C 5.48815400 3.74262800 0.25163000

C 6.04506500 4.44582100 -0.81879100

H 3.44637900 4.26148900 -2.98875900

H 3.78280200 2.67422000 1.00183300

H 5.70707500 5.17436300 -2.81973900

H 6.05212600 3.61175400 1.17150700

H 7.04670900 4.86060400 -0.74397300

H -2.06044300 0.20421700 -4.74268800

H -2.33143900 0.82442000 -3.10650100

H -2.29416100 1.93504600 -4.48602900

H 6.70280000 -0.28767700 2.90172900

H 5.22345800 -1.14158200 3.32670500

H 6.65535800 -2.05059200 2.80877200

C -3.86584000 -0.89609400 3.51237800

C -5.01569500 -0.09148700 3.58948400

C -3.86939400 -2.11414000 4.21537200

C -6.11725800 -0.47874300 4.35007800

H -5.04054600 0.84258400 3.03714700

C -4.97366100 -2.50604400 4.97110100

H -3.00263700 -2.76651800 4.17374300

C -6.10277200 -1.68856100 5.04611600

H -6.99172300 0.16571300 4.39637800

H -4.94925200 -3.45255400 5.50544400

H -6.96146600 -1.99233000 5.63925900

C -0.35482300 3.64979300 -3.32589300

H 0.07997200 3.86840400 -4.31166700

H -1.44267400 3.70902000 -3.42214000

H -0.04805000 4.44086300 -2.63846800

H 0.08128100 0.21128500 -3.57122400

H 5.17988700 -0.02794300 1.02374600

C 5.15686600 -3.50196700 0.92096800

H 6.08551200 -3.59123400 0.33972200

H 5.39799200 -3.71627800 1.96572800

H 4.47054000 -4.28057300 0.58129500

- **1da**-*RR* (HH-2)


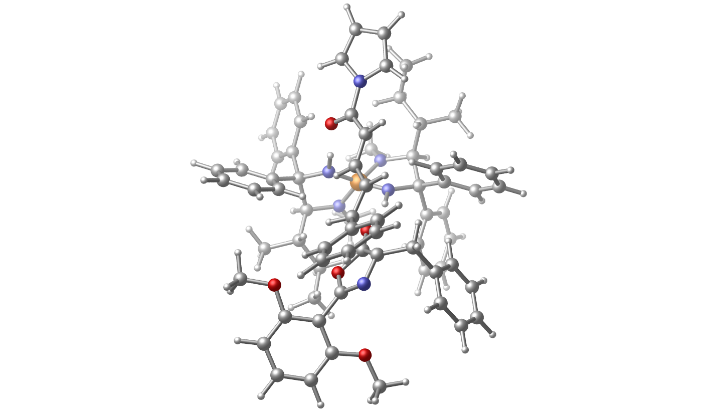

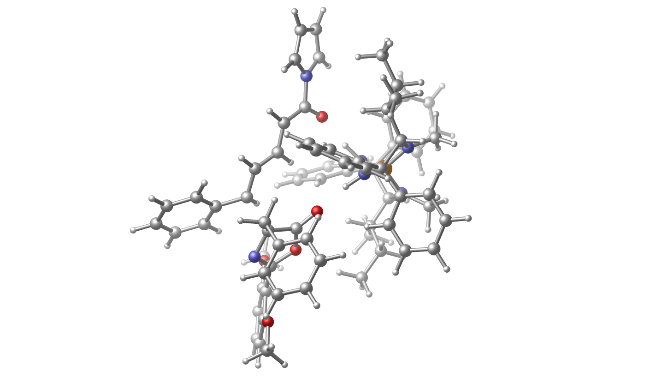


B3LYP/6-31G*

E = –3797.843126

Zero-point correction = 1.342173 (Hartree/Particle)

Thermal correction to Energy = 1.420456

Thermal correction to Enthalpy = 1.421400

Thermal correction to Gibbs Free Energy = 1.220799

Sum of electronic and zero-point Energies = –3796.500952

Sum of electronic and thermal Energies = –3796.422669

Sum of electronic and thermal Enthalpies = –3796.421725

Sum of electronic and thermal Free Energies = –3796.622327

PCM(toluene)-B3LYP-D3/6-311++G**//B3LYP/6-31G*@–30 °C

E = –3799.039651

G = –3797.78433

0 1

O 0.99085800 0.35727500 -0.17951500

C 2.19074800 0.23998100 0.05270200

C 3.10197800 0.87847700 0.99024800

O 2.93450000 -0.75251000 -0.58070100

N 4.39799800 0.38952500 0.69843400

C 4.24674000 -0.55354600 -0.15881000

C 5.28106600 -1.40900900 -0.76531800

C 5.24396000 -2.80768100 -0.60520500

C 6.32779900 -0.83634500 -1.51806100

C 6.22882700 -3.62206600 -1.17784300

C 7.31871000 -1.64742400 -2.08866800

C 7.25371800 -3.02689200 -1.91030100

O 4.21424400 -3.28501600 0.15259600

C 4.05027800 -4.69221500 0.25949100

H 4.88068800 -5.15431600 0.80900400

H 3.96363400 -5.16026000 -0.72895300

H 3.11978500 -4.84191900 0.80819400

O 6.28165100 0.51263700 -1.66201800

C 7.28143600 1.15486300 -2.43567700

H 8.27939100 1.01833900 -1.99893300

H 7.28709700 0.79142900 -3.47191800

H 7.02018600 2.21412800 -2.42400300

H 6.21065400 -4.69686100 -1.04762300

H 8.12505700 -1.21490700 -2.66792700

H 8.02256600 -3.65518300 -2.35228900

C 2.95502000 2.33699300 1.36353400

H 1.91473600 2.51039600 1.65815900

H 3.58492100 2.52515700 2.24093200

C 3.34572100 3.29398300 0.24894600

C 2.36945300 3.95364600 -0.50810200

C 4.69625300 3.53330100 -0.04670000

C 2.72846300 4.82944500 -1.53564100

C 5.05707300 4.41316200 -1.06724800

C 4.07433100 5.06447200 -1.81790300

H 1.31890600 3.78645000 -0.28672600

H 5.46168700 3.01455100 0.52321000

H 1.95234800 5.33092000 -2.10862900

H 6.10943600 4.59935700 -1.27137100

H 4.35666300 5.75248700 -2.61113000

C 2.52438400 -0.16285200 2.69859100

C 1.30097700 0.34173100 3.24137600

H 2.43018700 -1.11195300 2.17160900

H 1.36176800 1.11106000 4.00885700

C 0.04372800 -0.14618400 2.92730600

H -0.02612300 -0.92498600 2.17171800

C -1.15408300 0.21405900 3.56697000

H -1.09993300 0.90717900 4.39979400

C -2.37950800 -0.42876800 3.29670400

O -2.60956400 -1.28805300 2.40645600

N -3.49126600 -0.11179400 4.15755500

C -4.60316000 -0.92698100 4.27506200

C -3.64362500 0.96313500 5.02199300

C -5.44998600 -0.37999500 5.20781700

C -4.84365500 0.82622200 5.67694900

H -4.65776900 -1.83484400 3.69679800

H -2.90592700 1.74796800 5.07166100

H -6.39644000 -0.79743900 5.52560400

H -5.24656100 1.51720500 6.40614500

H -0.67805800 1.05210600 -0.43759200

N -1.65717200 1.15182200 -0.72026300

P -2.49662700 -0.13426200 -1.29442300

N -3.89339600 0.74079800 -1.48849600

C -3.80268400 2.14149400 -0.99795600

C -2.23752800 2.45963800 -1.09246600

C -5.08392600 0.27846000 -2.19646500

H -4.89167600 -0.70072100 -2.63754200

H -5.94180800 0.17590500 -1.52923100

H -5.34242000 0.98148300 -2.99931100

H -4.33677100 2.74458700 -1.73699200

C -4.46774500 2.42489900 0.38669700

H -3.71974000 2.25866200 1.16991800

C -5.65520800 1.49446100 0.71575700

H -6.44649600 1.62447400 -0.03673000

C -6.25625000 1.72978400 2.10754600

C -1.72029800 3.53047400 -0.11099900

C -1.34649300 3.18297700 1.19634700

C -1.60521600 4.87527500 -0.49809200

C -0.89369900 4.15487300 2.09121000

C -1.15478500 5.84481500 0.39848800

C -0.79800200 5.48952600 1.69965700

H -1.39906200 2.15170400 1.52894600

H -1.86203500 5.17344500 -1.50882800

H -0.60987600 3.85541100 3.09629900

H -1.07423900 6.87819300 0.07197600

H -0.43888200 6.24292400 2.39533200

C -1.81658200 2.83098000 -2.54099700

C -0.52550300 2.48859200 -2.97690000

C -2.63457300 3.55201000 -3.42270300

C -0.07902200 2.83565000 -4.25157000

C -2.19135800 3.89524200 -4.70385100

C -0.91300800 3.53556600 -5.12583600

H 0.14343400 1.94778500 -2.31494200

H -3.62648300 3.87415200 -3.12143600

H 0.92673300 2.56009000 -4.55667600

H -2.85106400 4.44952000 -5.36641200

H -0.56713800 3.80282700 -6.12064400

N -2.21564400 -1.40963100 -0.31479200

C -2.36036200 -2.71757100 -0.99544100

C -1.76410100 -2.38843300 -2.44173600

N -2.23254500 -0.99747900 -2.68605400

H -2.27462000 -1.33676600 0.72040000

C -2.56862000 -0.55403700 -4.03538400

H -3.01742100 0.43947400 -3.99294700

H -1.68522900 -0.48973700 -4.67343000

H -3.28638500 -1.24606900 -4.49584400

H -2.26824800 -3.00548900 -3.19048700

C -0.22990800 -2.61770200 -2.61945300

H 0.26792700 -2.37665300 -1.67437500

C 0.40580600 -1.70561800 -3.69196300

H -0.05041800 -1.91528600 -4.67104000

C 1.92843300 -1.85119600 -3.81334600

C -1.59429900 -3.77728600 -0.17888300

C -0.46886400 -3.41646200 0.57512400

C -2.01602400 -5.11485300 -0.14028800

C 0.20964300 -4.36069400 1.34622300

C -1.32851400 -6.06261000 0.61872900

C -0.21504100 -5.68903000 1.37149500

H -0.12624600 -2.38843000 0.56839100

H -2.89625500 -5.42327500 -0.69386500

H 1.05998300 -4.04456400 1.94468400

H -1.67872700 -7.09137300 0.63323300

H 0.30613500 -6.42217100 1.98149000

C -3.86452200 -3.09920900 -1.07203900

C -4.43245100 -3.80311600 -2.14310500

C -4.69495300 -2.77056900 0.01368200

C -5.79024600 -4.13870500 -2.14906100

C -6.04796200 -3.10832200 0.00964400

C -6.60601400 -3.78770600 -1.07546800

H -3.82504500 -4.11742600 -2.98614500

H -4.27905700 -2.25286400 0.87323900

H -6.20314800 -4.67922800 -2.99696500

H -6.66592000 -2.84085500 0.86277400

H -7.66128900 -4.04703500 -1.07937600

H 2.32228900 -1.11606200 -4.52509000

H 2.41737300 -1.67744700 -2.84982300

H 2.22294600 -2.84193900 -4.17581000

H -6.99488100 0.95508700 2.33870000

H -5.49101900 1.69013900 2.88977300

H -6.76449900 2.69765400 2.17769200

C 3.76183500 -0.11061900 3.52600300

C 4.70824000 -1.14392600 3.41417200

C 4.01996600 0.91930800 4.44773800

C 5.86165800 -1.15353500 4.19531400

H 4.53258000 -1.94499900 2.70145600

C 5.17727200 0.91480000 5.22480400

H 3.31066200 1.73264300 4.56613000

C 6.10347800 -0.12283700 5.10507300

H 6.57486900 -1.96739500 4.09105700

H 5.35267000 1.72327000 5.93018500

H 7.00300000 -0.12813700 5.71517700

C 0.02406900 -4.09725600 -2.96339300

H -0.33802000 -4.31979400 -3.97719000

H 1.09260300 -4.32857700 -2.93577900

H -0.47356600 -4.77945300 -2.27144600

H 0.18012100 -0.66186800 -3.44866900

H -5.31939200 0.45319600 0.65389100

C -4.91360500 3.89827100 0.43721200

H -5.76656700 4.06125300 -0.23665900

H -5.22964500 4.17843200 1.44565500

H -4.11692500 4.58589300 0.14543600

- **1da**-*RR* (HMe)


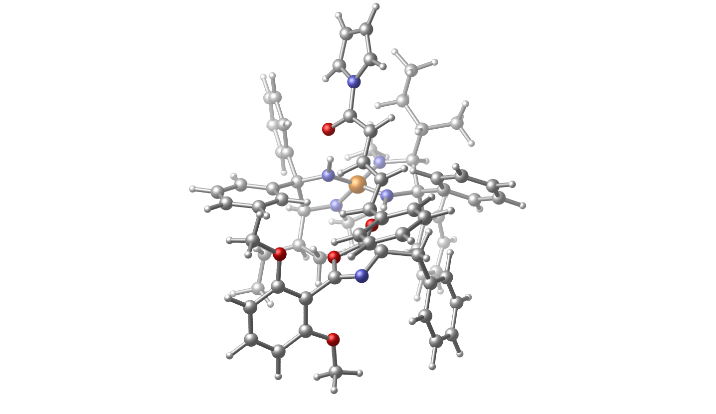

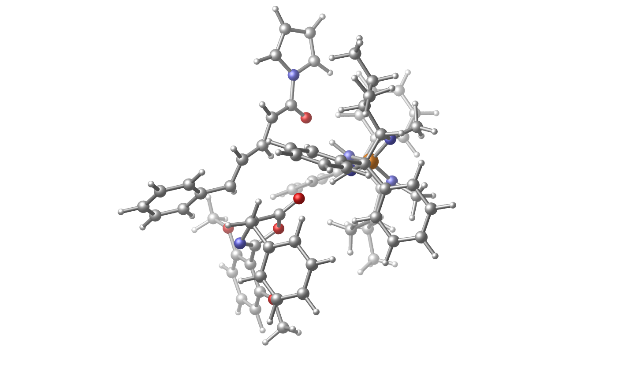


B3LYP/6-31G*

E = –3797.84022

Zero-point correction = 1.341429 (Hartree/Particle)

Thermal correction to Energy = 1.420011

Thermal correction to Enthalpy = 1.420955

Thermal correction to Gibbs Free Energy = 1.219630

Sum of electronic and zero-point Energies = –3796.498791

Sum of electronic and thermal Energies = –3796.420210

Sum of electronic and thermal Enthalpies = –3796.419266

Sum of electronic and thermal Free Energies = –3796.620590

0 1

O -0.95473000 -0.65405200 -0.05688000

C -2.14763900 -0.41829300 0.07637800

C -3.23898200 -1.15034600 0.69635000

O -2.68506500 0.81567400 -0.30077200

N -4.42739900 -0.41856000 0.42930700

C -4.05218900 0.69494500 -0.08450800

C -4.89605000 1.83233100 -0.50602600

C -5.11420400 2.92835700 0.34484600

C -5.50375000 1.81674700 -1.77485700

C -5.93577100 3.99022700 -0.05476300

C -6.32679900 2.87466000 -2.18357500

C -6.52956700 3.94464600 -1.31481400

O -4.48091400 2.86976100 1.55569800

C -4.71858700 3.90980200 2.49627200

H -5.78311700 3.98083100 2.75173100

H -4.36921500 4.87911400 2.11874500

H -4.14758000 3.64018600 3.38578100

O -5.22602300 0.72454600 -2.53506000

C -5.85073400 0.59160700 -3.80155300

H -6.94478600 0.58707600 -3.71188900

H -5.54719400 1.39244900 -4.48888400

H -5.51471400 -0.37044300 -4.19182400

H -6.11291900 4.83764700 0.59579300

H -6.80017700 2.86996000 -3.15777600

H -7.16718600 4.76710600 -1.62809400

C -3.28327100 -2.66042800 0.60992500

H -2.34769600 -3.05333300 1.02270100

H -4.10147000 -3.01419900 1.24640800

C -3.47247500 -3.17800400 -0.80738300

C -2.42363700 -3.80972000 -1.48806900

C -4.70151300 -3.02491100 -1.46742800

C -2.59475400 -4.27954500 -2.79306600

C -4.87520000 -3.49549900 -2.76900800

C -3.82205500 -4.12502800 -3.43850200

H -1.46683600 -3.94153000 -0.98975200

H -5.51728200 -2.52289000 -0.95514000

H -1.76714800 -4.77017000 -3.29976100

H -5.83905900 -3.37849900 -3.25961900

H -3.96049000 -4.49672400 -4.45100400

C -2.84584300 -0.66202200 2.65020400

C -1.54741000 -1.05866500 3.10818500

H -2.94234700 0.40289600 2.43925600

H -1.45926300 -1.98601300 3.67107500

C -0.41147700 -0.27655600 2.97151900

H -0.49567200 0.64839100 2.40311800

C 0.83423700 -0.50606300 3.57843500

H 0.94439300 -1.35110300 4.25039200

C 1.90404500 0.40033600 3.42520900

O 1.99891500 1.31035400 2.56127300

N 3.00062200 0.29816200 4.35180900

C 4.19376200 0.98182600 4.19351700

C 3.04675600 -0.38661600 5.55896500

C 4.99264100 0.72480600 5.28034900

C 4.26222800 -0.14411500 6.14964400

H 4.35358400 1.57620200 3.30930800

H 2.19523000 -0.94470000 5.91352800

H 5.98981400 1.11429000 5.43978500

H 4.58804500 -0.53419300 7.10524100

H 0.72835300 -1.06664800 -0.63389900

N 1.71936400 -1.15932500 -0.87178300

P 2.66711400 0.14552300 -1.16201500

N 4.06841500 -0.74361400 -1.11194700

C 3.87296100 -2.16858500 -0.73104800

C 2.35915800 -2.46188300 -1.15652100

C 5.37378800 -0.27424500 -1.56751600

H 5.27127600 0.72571500 -1.99202900

H 6.09482300 -0.22013500 -0.74898700

H 5.76957700 -0.94648300 -2.34044100

H 4.54618700 -2.73739100 -1.37763500

C 4.24641900 -2.53798800 0.73882900

H 3.36082700 -2.39366900 1.36793100

C 5.35875200 -1.65607500 1.34600500

H 6.27891400 -1.75917900 0.75249100

C 5.67622500 -1.99332500 2.80855400

C 1.65363100 -3.56766300 -0.34753300

C 1.03845200 -3.28630500 0.88120800

C 1.61462100 -4.88866700 -0.82295700

C 0.42776400 -4.30223900 1.62023400

C 1.00417600 -5.90142900 -0.08314300

C 0.40938700 -5.61315400 1.14585600

H 1.01798700 -2.27568800 1.27319100

H 2.06100800 -5.13367500 -1.78087500

H -0.04127700 -4.05376700 2.56790800

H 0.98878000 -6.91496200 -0.47496700

H -0.07172000 -6.39974800 1.72074300

C 2.22270900 -2.77078800 -2.67346900

C 0.98671200 -2.50596100 -3.28798500

C 3.23016800 -3.34896000 -3.45618500

C 0.77498300 -2.78431500 -4.63673900

C 3.02339500 -3.62305300 -4.81296900

C 1.79822000 -3.33790600 -5.41088600

H 0.17451100 -2.08770200 -2.70141300

H 4.19072800 -3.60976800 -3.02392000

H -0.19496000 -2.57281600 -5.07852000

H 3.82685900 -4.06584300 -5.39591500

H 1.63743100 -3.55234900 -6.46390300

N 2.15495300 1.36479700 -0.20593000

C 2.31482700 2.71503800 -0.80027500

C 2.17516000 2.44548300 -2.35873100

N 2.75439600 1.09056300 -2.52771400

H 2.04164600 1.26238500 0.82284700

C 3.07765300 0.60000900 -3.86212500

H 3.67383600 -0.31094700 -3.78617100

H 2.18484700 0.36605600 -4.45279900

H 3.66536900 1.35647600 -4.39650200

H 2.84581100 3.14468700 -2.86672200

C 0.83018200 2.58628000 -3.14317100

H 1.14373400 2.31774800 -4.16471600

C 0.32508500 4.04310800 -3.25951700

H -0.25042500 4.31559600 -2.37087800

C -0.53002900 4.28543300 -4.51049200

C 1.24082500 3.66154000 -0.24135600

C 0.04339200 3.16905400 0.29013300

C 1.44127500 5.04918900 -0.27309300

C -0.94379700 4.04230000 0.75084300

C 0.45746800 5.92230300 0.18873600

C -0.74191300 5.42152500 0.69918400

H -0.12755900 2.10052500 0.33877100

H 2.37222500 5.45225400 -0.65991400

H -1.87010000 3.63084200 1.14159300

H 0.63209600 6.99456000 0.15317000

H -1.50809500 6.10313600 1.06002700

C 3.72506800 3.25143000 -0.41296900

C 4.74045800 3.58762300 -1.31589100

C 3.99635300 3.38815900 0.96162300

C 5.98527900 4.04678200 -0.86694400

C 5.23300600 3.84625000 1.40813800

C 6.23718100 4.18051100 0.49476600

H 4.59218700 3.50266300 -2.38701700

H 3.23525200 3.11883500 1.68813700

H 6.75235200 4.30181400 -1.59385200

H 5.41009200 3.94558500 2.47592100

H 7.20124000 4.54103000 0.84343900

H -0.84698200 5.33307800 -4.56573900

H 0.02777500 4.05794800 -5.42822500

H -1.43646700 3.67013600 -4.51070700

H 6.36697400 -1.25649800 3.22894000

H 4.77319200 -1.97368000 3.42838400

H 6.13979300 -2.98037800 2.91097900

C -4.04993200 -1.19716200 3.35109900

C -5.18946600 -0.38501300 3.48270400

C -4.09250600 -2.48474700 3.91483800

C -6.31922600 -0.83299600 4.16451400

H -5.18123700 0.60495600 3.03738000

C -5.22477300 -2.93686500 4.59142900

H -3.23453400 -3.14382200 3.82361500

C -6.34319500 -2.11186700 4.72382100

H -7.18501300 -0.18185000 4.25676200

H -5.23113600 -3.93655800 5.01876200

H -7.22379600 -2.46306100 5.25547300

C -0.28122000 1.60023100 -2.76375000

H -0.74069100 1.82950200 -1.80250400

H -1.07455600 1.62178800 -3.51873400

H 0.08865500 0.57115900 -2.72097500

H 1.18505400 4.72708700 -3.28821400

H 5.05477900 -0.60448400 1.28865900

C 4.65693200 -4.02195000 0.79425100

H 5.63322300 -4.16429000 0.30959700

H 4.74700300 -4.36644400 1.82794000

H 3.93638200 -4.67486100 0.29787600

- **1da**-*RR* (MeH)


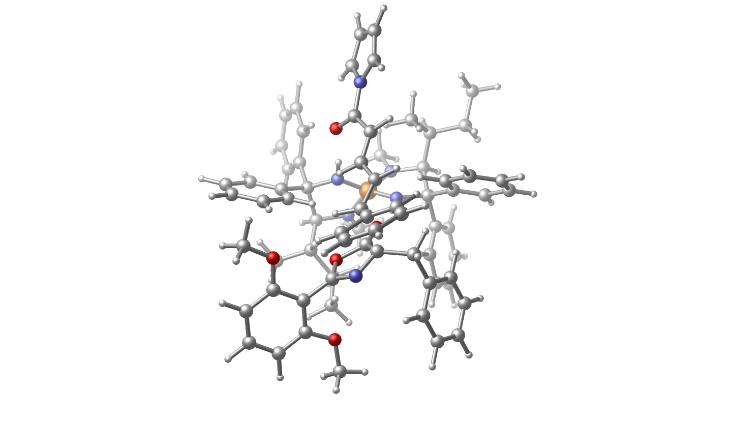

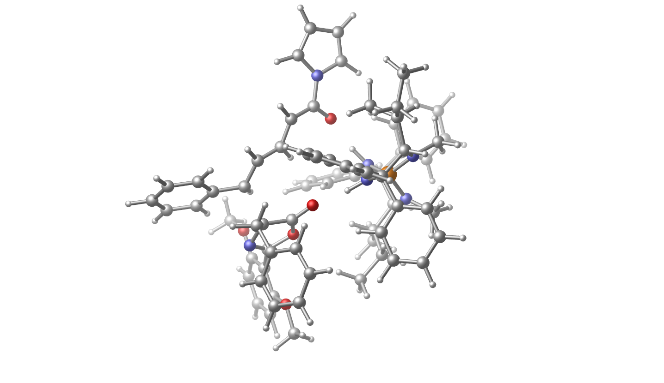


B3LYP/6-31G*

E = –3797.843528

Zero-point correction = 1.341814 (Hartree/Particle)

Thermal correction to Energy = 1.420404

Thermal correction to Enthalpy = 1.421348

Thermal correction to Gibbs Free Energy = 1.219660

Sum of electronic and zero-point Energies = –3796.501714

Sum of electronic and thermal Energies = –3796.423124

Sum of electronic and thermal Enthalpies = –3796.422179

Sum of electronic and thermal Free Energies = –3796.623868

0 1

O -0.86825200 -0.73415200 0.02234800

C -2.06931100 -0.52901400 0.13295300

C -3.12326500 -1.19979600 0.87632800

O -2.65919400 0.60679300 -0.42684800

N -4.34312900 -0.56223800 0.51774400

C -4.01990200 0.47045300 -0.16919200

C -4.91233800 1.49185600 -0.75490700

C -5.26811700 2.64792100 -0.04045400

C -5.43090900 1.29815100 -2.04825800

C -6.13846100 3.59204200 -0.60097700

C -6.30164300 2.23664200 -2.61687200

C -6.64190600 3.36967900 -1.88113800

O -4.71572900 2.76925400 1.20421900

C -5.08138000 3.88958700 2.00034100

H -6.16238200 3.91203300 2.18593600

H -4.77239900 4.83191300 1.53067200

H -4.55320400 3.76465500 2.94673700

O -5.02173400 0.15848400 -2.66876600

C -5.56843800 -0.16602700 -3.93775400

H -6.66201400 -0.24747300 -3.89282200

H -5.28963300 0.57410000 -4.69893000

H -5.14221600 -1.13586000 -4.19896700

H -6.42162800 4.48527200 -0.05837600

H -6.70652900 2.09319100 -3.61115600

H -7.31677500 4.10128900 -2.31746000

C -3.10795500 -2.70897100 1.00219200

H -2.15717000 -3.00269500 1.46114400

H -3.91103800 -3.00168500 1.68705000

C -3.27579100 -3.42893800 -0.32661000

C -2.20695000 -4.12403800 -0.90699300

C -4.50411100 -3.40363100 -1.00507000

C -2.35722700 -4.77869400 -2.13275300

C -4.65665800 -4.05684300 -2.22804400

C -3.58337200 -4.74751400 -2.79808200

H -1.25075400 -4.15907500 -0.39055200

H -5.33605800 -2.85678400 -0.57068600

H -1.51488700 -5.31592600 -2.56168700

H -5.61943500 -4.03424600 -2.73373500

H -3.70530900 -5.26283800 -3.74779600

C -2.74130300 -0.44401800 2.73151600

C -1.43157200 -0.74267700 3.22920800

H -2.86739600 0.58309700 2.38855900

H -1.31452300 -1.59967300 3.88957800

C -0.31937600 0.05905600 3.02147000

H -0.42403400 0.91804700 2.36047400

C 0.91737800 -0.07449100 3.67015500

H 1.02778700 -0.85146700 4.41936800

C 1.98760300 0.81869600 3.45236800

O 2.11353700 1.63458900 2.50187600

N 3.05953900 0.79976500 4.41190900

C 4.28620900 1.40615700 4.20190900

C 3.06701200 0.23069700 5.67825800

C 5.06817500 1.21046700 5.31297000

C 4.29020800 0.46579400 6.25407300

H 4.47787800 1.91391500 3.27140400

H 2.18847900 -0.25209200 6.07451700

H 6.08230000 1.56494100 5.44446100

H 4.59183800 0.15211600 7.24505500

H 0.84215600 -1.06416300 -0.50484600

N 1.84453500 -1.05621200 -0.70576400

P 2.57783800 0.32308500 -1.20177300

N 4.07719900 -0.38630000 -1.27609600

C 4.10896600 -1.79570100 -0.80507300

C 2.60868100 -2.28250300 -1.00287300

C 5.31836300 0.32392900 -1.55892200

H 5.09304600 1.27689500 -2.04192900

H 5.89068900 0.53948100 -0.64978300

H 5.94365300 -0.27210700 -2.23549700

H 4.73684700 -2.34077600 -1.51518700

C 4.85109600 -1.92310900 0.55968900

H 5.79892500 -1.40004900 0.35640700

C 5.26635900 -3.37174200 0.90574600

H 4.42653900 -3.90730700 1.35739400

C 6.47510700 -3.44170600 1.84863400

C 2.16893900 -3.43042300 -0.07697500

C 1.41303000 -3.19779400 1.07974500

C 2.50118100 -4.75448100 -0.40345900

C 1.02516500 -4.26202900 1.89913800

C 2.11942400 -5.81349000 0.41802500

C 1.37897800 -5.57150000 1.57698000

H 1.11583600 -2.19265500 1.35499400

H 3.05904900 -4.96378100 -1.31091600

H 0.44076800 -4.05509900 2.79141900

H 2.39335600 -6.82930700 0.14556500

H 1.07352900 -6.39627800 2.21510000

C 2.31722300 -2.71034200 -2.47490300

C 0.97396500 -2.71374300 -2.89088500

C 3.28362400 -3.13664300 -3.39427300

C 0.61422600 -3.11153600 -4.17629600

C 2.92611000 -3.53153700 -4.68883000

C 1.59232700 -3.51904500 -5.08739500

H 0.19257200 -2.41152500 -2.20019800

H 4.33367400 -3.17967600 -3.12460700

H -0.43517500 -3.10999100 -4.45774700

H 3.70098800 -3.85260900 -5.38027600

H 1.31542800 -3.82831700 -6.09158300

N 2.02463100 1.54910700 -0.27452600

C 1.97907600 2.85950400 -0.97397700

C 1.61041300 2.41378700 -2.46209600

N 2.37251900 1.15119500 -2.62387100

H 1.95602900 1.49628900 0.76106200

C 2.96536400 0.80469600 -3.91060600

H 3.59144000 -0.08146500 -3.79665500

H 2.20457300 0.58345800 -4.66308200

H 3.58877600 1.63275400 -4.27339900

H 2.04444600 3.12330600 -3.17104400

C 0.08668900 2.30929300 -2.79854300

H -0.44792500 1.99322400 -1.89721700

C -0.23142600 1.25424400 -3.88210700

H 0.22799400 1.55202000 -4.83663000

C -1.73279600 1.02549300 -4.10292000

C 0.93015200 3.75678100 -0.29554500

C -0.20943500 3.20171400 0.30265100

C 1.07653800 5.15174300 -0.28325500

C -1.18709000 4.01765100 0.87201300

C 0.10302200 5.96797800 0.29377900

C -1.03501500 5.40414400 0.87093600

H -0.34113300 2.12637900 0.32051800

H 1.95712800 5.60868900 -0.72210300

H -2.07025600 3.55782000 1.30586500

H 0.24064400 7.04614500 0.29407800

H -1.79217400 6.04102000 1.32130900

C 3.37901500 3.52568300 -0.88787000

C 4.00960200 4.18667300 -1.94980100

C 4.03685200 3.49810700 0.35525700

C 5.26965500 4.77464000 -1.78835000

C 5.28824800 4.08886000 0.51763500

C 5.91681400 4.72445600 -0.55641500

H 3.53118000 4.26821700 -2.92034200

H 3.55766400 3.01822100 1.20436000

H 5.73663600 5.27581100 -2.63243200

H 5.77141200 4.05690000 1.49079300

H 6.89414800 5.18247500 -0.42993400

H -1.88798400 0.21171800 -4.82143600

H -2.23437500 0.74814100 -3.16998500

H -2.23324900 1.91384300 -4.50362200

H 6.75084000 -4.48324500 2.04916000

H 7.35119000 -2.94334000 1.41402000

H 6.26654000 -2.96628200 2.81292700

C -3.92730000 -0.91347500 3.50978900

C -5.09420900 -0.13032400 3.53014000

C -3.92543800 -2.10643300 4.25432000

C -6.20748700 -0.51370600 4.27569100

H -5.12310900 0.78352500 2.94509600

C -5.04104900 -2.49463000 4.99528600

H -3.04533200 -2.74209900 4.25799600

C -6.18754000 -1.69840700 5.01350100

H -7.09514600 0.11418600 4.27751900

H -5.01173200 -3.42152200 5.56275900

H -7.05511900 -1.99911400 5.59515500

C -0.43960400 3.69027700 -3.23103700

H -0.01790300 3.96957100 -4.20709600

H -1.52866200 3.68150100 -3.32980700

H -0.18556100 4.47589900 -2.51686900

H 0.21800200 0.29677500 -3.59798400

H 5.51338000 -3.91330100 -0.01932400

C 4.19681300 -1.19919600 1.74024900

H 3.29808300 -1.71151900 2.08901500

H 4.89132700 -1.13648300 2.58334300

H 3.91642100 -0.17247200 1.49044500

- **1da**-*RR* (MeMe)


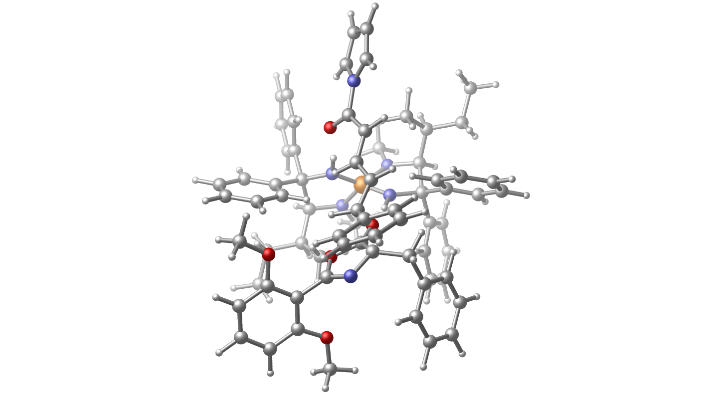

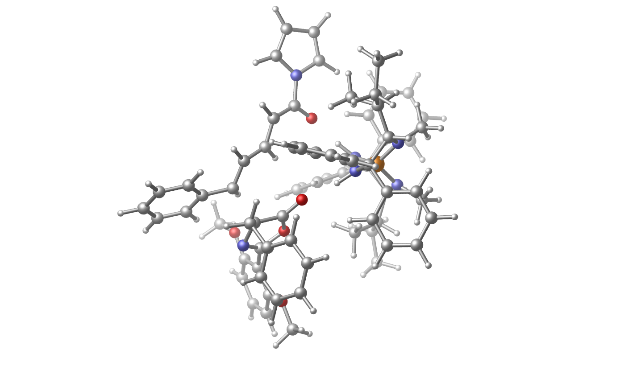


B3LYP/6-31G*

E = –3797.83938

Zero-point correction = 1.341142 (Hartree/Particle)

Thermal correction to Energy = 1.420091

Thermal correction to Enthalpy = 1.421035

Thermal correction to Gibbs Free Energy = 1.216828

Sum of electronic and zero-point Energies = –3796.498238

Sum of electronic and thermal Energies = –3796.419289

Sum of electronic and thermal Enthalpies = –3796.418345

Sum of electronic and thermal Free Energies = –3796.622552

0 1

O -0.93493700 -0.79738000 -0.06492500

C -2.12453300 -0.53951100 0.05798500

C -3.22939500 -1.25530100 0.67524000

O -2.64314900 0.69779500 -0.32983700

N -4.40768300 -0.51129100 0.38944900

C -4.01435700 0.59504800 -0.12534000

C -4.83718700 1.74376700 -0.55762800

C -5.10709600 2.81304300 0.31239400

C -5.37022300 1.76695500 -1.85936700

C -5.90613600 3.88625800 -0.10265900

C -6.16970500 2.83645100 -2.28368000

C -6.42481500 3.87952300 -1.39605000

O -4.54496300 2.72011000 1.55553700

C -4.84402300 3.72927600 2.51229200

H -5.92236200 3.78917400 2.70466900

H -4.47680300 4.71106000 2.18763200

H -4.32695900 3.43271700 3.42590700

O -5.04954000 0.69549100 -2.63288800

C -5.59237700 0.60577600 -3.94045500

H -6.68992200 0.60263800 -3.92130100

H -5.24380700 1.42732900 -4.58022100

H -5.23401100 -0.34433100 -4.34006400

H -6.12201600 4.71375100 0.56163500

H -6.58618400 2.86087600 -3.28321600

H -7.04437100 4.71122800 -1.72085800

C -3.28544100 -2.76562300 0.58968100

H -2.36398400 -3.16508400 1.02794900

H -4.12164500 -3.11043000 1.20755200

C -3.44181800 -3.29264000 -0.82857800

C -2.41265400 -4.01818100 -1.44219700

C -4.62152500 -3.06201400 -1.55373300

C -2.55446600 -4.50456800 -2.74507000

C -4.76521100 -3.54704800 -2.85347900

C -3.73202100 -4.27062800 -3.45594200

H -1.49468500 -4.21103900 -0.89259100

H -5.42138300 -2.48951100 -1.09315900

H -1.74350800 -5.06890400 -3.19888400

H -5.69114900 -3.36747000 -3.39546200

H -3.84845200 -4.65436800 -4.46670600

C -2.86775300 -0.76651600 2.61940700

C -1.57190000 -1.14745700 3.10058800

H -2.97246100 0.29840000 2.41147000

H -1.48236000 -2.07357900 3.66510400

C -0.44264700 -0.35104100 2.99263300

H -0.52131900 0.57329800 2.42175600

C 0.78513300 -0.57298000 3.63589200

H 0.87795000 -1.42202500 4.30527300

C 1.87427000 0.31227500 3.50207400

O 2.01526200 1.21362500 2.63423900

N 2.94771700 0.17476500 4.45065200

C 4.20280100 0.73181000 4.27534700

C 2.92864600 -0.48095800 5.67351100

C 4.97639500 0.41907100 5.36589200

C 4.16354400 -0.34817500 6.25793400

H 4.42086100 1.28568700 3.37726600

H 2.02582400 -0.94340600 6.03885600

H 6.00842300 0.70937200 5.51464000

H 4.45072400 -0.74361800 7.22364800

H 0.78688800 -0.97242400 -0.69413100

N 1.78773100 -1.03017400 -0.90064600

P 2.67465000 0.31740200 -1.19161700

N 4.09766900 -0.53160500 -1.31350600

C 3.97937400 -1.97598700 -0.99098300

C 2.45135600 -2.29447100 -1.28298000

C 5.40897100 0.06937000 -1.52057300

H 5.28761000 1.07565400 -1.92646100

H 5.98322600 0.14958900 -0.59052000

H 5.98412000 -0.53087900 -2.23671200

H 4.57990800 -2.50902800 -1.73356600

C 4.66098400 -2.30701500 0.37264900

H 5.66442700 -1.87334300 0.23628300

C 4.90960700 -3.81698100 0.59263900

H 4.00393000 -4.29760800 0.97280600

C 6.07352700 -4.10270900 1.55102300

C 1.88516100 -3.47984300 -0.48278200

C 1.14411300 -3.29807800 0.69138000

C 2.10012000 -4.78851000 -0.94368800

C 0.65777100 -4.40096100 1.39956400

C 1.61981700 -5.88689300 -0.23332600

C 0.89651100 -5.69770000 0.94634900

H 0.92868700 -2.30441500 1.06430400

H 2.64546300 -4.95300400 -1.86793800

H 0.08654600 -4.23237400 2.30816500

H 1.80417900 -6.89041100 -0.60766200

H 0.51510000 -6.55217600 1.49883300

C 2.17238900 -2.55260600 -2.79526800

C 0.83136500 -2.48138800 -3.21363200

C 3.13844900 -2.87338900 -3.75607000

C 0.47366600 -2.70563700 -4.54015600

C 2.78260700 -3.09467600 -5.09247600

C 1.45186500 -3.00930900 -5.49156700

H 0.05198600 -2.26035200 -2.49020900

H 4.18668900 -2.96286000 -3.49064400

H -0.57382900 -2.65258900 -4.82372300

H 3.55666800 -3.33772000 -5.81592700

H 1.17636600 -3.18436200 -6.52804500

N 2.16795200 1.45739300 -0.13754500

C 2.26530700 2.84927900 -0.64013400

C 2.08249200 2.68150100 -2.21053300

N 2.67813900 1.35414400 -2.49014100

H 2.04698900 1.27360800 0.87780900

C 2.99852900 0.97475800 -3.86046400

H 3.61220100 0.07235200 -3.85638100

H 2.10338300 0.76923000 -4.45885500

H 3.56468400 1.78198100 -4.34132200

H 2.72858700 3.42138500 -2.69004300

C 0.71588600 2.85108200 -2.95084300

H 1.01263100 2.67341500 -3.99715400

C 0.17497700 4.29915000 -2.93652500

H -0.38533000 4.48351900 -2.01611200

C -0.71592400 4.62125300 -4.14380100

C 1.18153400 3.71779300 0.01931000

C 0.00257000 3.15148300 0.51836900

C 1.35086600 5.10639300 0.11490300

C -0.99759800 3.95502600 1.06784700

C 0.35441500 5.91017300 0.66752000

C -0.82696600 5.33755600 1.14180900

H -0.14366700 2.07918800 0.47379100

H 2.26754500 5.56590000 -0.24137300

H -1.90999600 3.48850800 1.42826900

H 0.50604300 6.98461500 0.73074600

H -1.60267800 5.96495400 1.57359600

C 3.67051000 3.40544700 -0.25972500

C 4.58956400 3.97251900 -1.15047300

C 4.03216900 3.33271900 1.09917300

C 5.83316500 4.44057700 -0.70696500

C 5.26691300 3.80117200 1.54048700

C 6.17869000 4.35685600 0.63820000

H 4.36508300 4.07073500 -2.20732900

H 3.34218300 2.89955900 1.81830700

H 6.52495700 4.87425400 -1.42472800

H 5.51379300 3.73478400 2.59696300

H 7.14209200 4.72293300 0.98291100

H -1.06118600 5.66062200 -4.10431900

H -0.17477700 4.48617800 -5.08932000

H -1.60555300 3.98272600 -4.17516800

H 6.23289800 -5.18194200 1.65554200

H 7.01028600 -3.66201400 1.18637000

H 5.88475800 -3.70123500 2.55223000

C -4.07420500 -1.31253400 3.31109400

C -5.22437700 -0.51331900 3.42731400

C -4.10732800 -2.59758900 3.88109100

C -6.35540600 -0.97109600 4.10073600

H -5.22398500 0.47380700 2.97593200

C -5.24072000 -3.05959100 4.54910000

H -3.24090500 -3.24705500 3.80217700

C -6.36993000 -2.24715000 4.66658000

H -7.22960700 -0.32966100 4.18123400

H -5.23930800 -4.05705500 4.98162300

H -7.25140100 -2.60587300 5.19172200

C -0.36112900 1.80775600 -2.63068500

H -0.81420000 1.95272400 -1.65036400

H -1.16659000 1.86100400 -3.37103700

H 0.03726600 0.78870300 -2.66967400

H 1.01825900 5.00419500 -2.92743400

H 5.12736000 -4.29568100 -0.37336200

C 4.05373200 -1.62081600 1.59989700

H 3.09582300 -2.05928600 1.88617700

H 4.72678900 -1.70477100 2.45835200

H 3.89405600 -0.55191900 1.43345100

- **1da**-*RR* (EtEt)


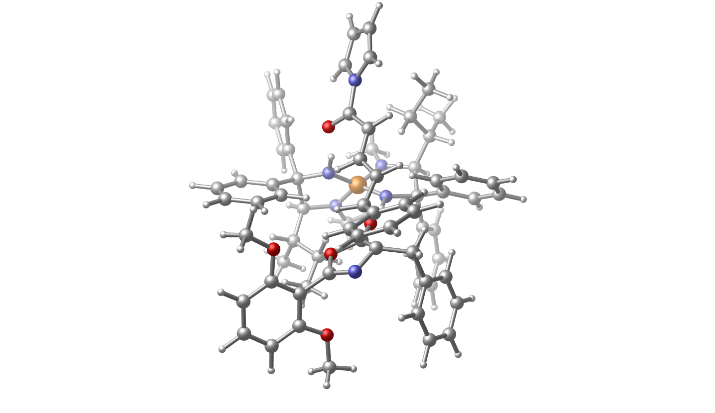

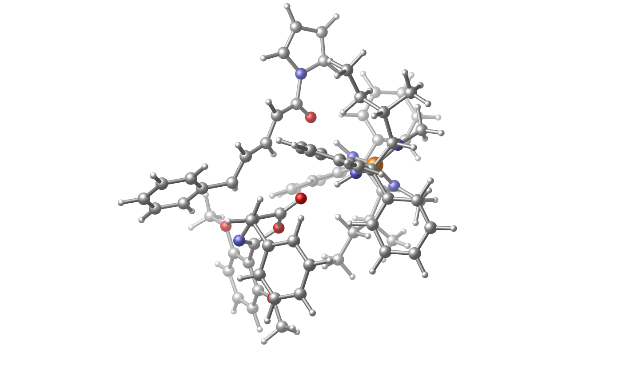


B3LYP/6-31G*

E = –3797.845811

Zero-point correction = 1.341656 (Hartree/Particle)

Thermal correction to Energy = 1.420221

Thermal correction to Enthalpy = 1.421165

Thermal correction to Gibbs Free Energy = 1.220963

Sum of electronic and zero-point Energies = –3796.504154

Sum of electronic and thermal Energies = –3796.425590

Sum of electronic and thermal Enthalpies = –3796.424646

Sum of electronic and thermal Free Energies = –3796.624848

PCM(toluene)-B3LYP-D3/6-311++G**//B3LYP/6-31G*@–30 °C

E = –3799.044961

G = –3797.789553

0 1

O 0.94722000 0.66533400 0.05815800

C 2.13269500 0.37262800 0.14876400

C 3.24472700 0.96148600 0.87721200

O 2.63066400 -0.79735000 -0.42629700

N 4.41008600 0.24485400 0.48872200

C 4.00104600 -0.75692400 -0.19897000

C 4.80849400 -1.83356000 -0.80842100

C 5.04928400 -3.03653300 -0.12454100

C 5.36014600 -1.65039400 -2.08932800

C 5.84316700 -4.03749900 -0.69887800

C 6.15403600 -2.64651700 -2.67289900

C 6.38416300 -3.82436000 -1.96537500

O 4.46274900 -3.14248900 1.10597200

C 4.71875900 -4.30645900 1.88247900

H 5.79013800 -4.42050400 2.08881000

H 4.34521900 -5.21041700 1.38515200

H 4.18063600 -4.16005800 2.81999100

O 5.05979200 -0.46389600 -2.68478700

C 5.63916700 -0.16460800 -3.94559300

H 6.73546600 -0.17968600 -3.89611500

H 5.30057800 -0.86354600 -4.72135300

H 5.30044300 0.84347400 -4.18974800

H 6.03834100 -4.96628100 -0.17749600

H 6.58402200 -2.51303500 -3.65797800

H 6.99974900 -4.59948500 -2.41400900

C 3.33969400 2.46490800 1.02736800

H 2.41852900 2.82006400 1.50238600

H 4.16894600 2.68593800 1.70826300

C 3.54812800 3.19967900 -0.28848400

C 2.55573100 4.04318300 -0.80383200

C 4.74058000 3.04900900 -1.01381700

C 2.74508600 4.72071600 -2.01180900

C 4.93174400 3.72508100 -2.21872800

C 3.93450200 4.56428600 -2.72401200

H 1.62860300 4.17563100 -0.25178400

H 5.51187300 2.38736200 -0.63059300

H 1.96134700 5.37207400 -2.39046700

H 5.86688600 3.60371900 -2.76107000

H 4.08775200 5.09642800 -3.65983600

C 2.84613600 0.19279700 2.72898600

C 1.55705500 0.54385700 3.24706900

H 2.91703500 -0.83117300 2.36215800

H 1.48573800 1.38612600 3.93240900

C 0.40967600 -0.19729500 3.01543600

H 0.47708900 -1.03797500 2.32609800

C -0.82804300 -0.03335600 3.65756300

H -0.92581400 0.72032500 4.43219900

C -1.92167500 -0.87751200 3.37841000

O -2.04180900 -1.65504100 2.39595300

N -3.02347700 -0.85635500 4.30313800

C -4.28261200 -1.34418400 4.00019200

C -3.03462300 -0.39602300 5.61177100

C -5.08939100 -1.18214500 5.09993600

C -4.29428100 -0.58412100 6.12675900

H -4.47783900 -1.74367200 3.01856100

H -2.13459600 -0.02256200 6.07368500

H -6.13325000 -1.46014300 5.16628900

H -4.60884600 -0.33517400 7.13200800

H -0.74494600 1.07178300 -0.54597600

N -1.74144400 1.22142600 -0.72178200

P -2.71731600 -0.01378900 -1.17906200

N -4.09309600 0.91103400 -1.08969500

C -3.87322300 2.32491500 -0.70132900

C -2.31080500 2.56532900 -0.93109900

C -5.43374700 0.41856400 -1.37975600

H -5.36644300 -0.53870900 -1.90105700

H -6.01917500 0.26364400 -0.46753500

H -5.96618200 1.13161800 -2.02128400

H -4.40828500 2.92478100 -1.44167400

C -4.46677500 2.74464000 0.67686700

H -3.95692900 3.68001900 0.93394100

C -4.20409100 1.74799500 1.82100200

H -4.79222700 0.83442800 1.66005400

C -1.70423600 3.57520700 0.05892600

C -1.02702300 3.16318100 1.21350400

C -1.84650800 4.95183800 -0.17947300

C -0.51552300 4.10675500 2.10932900

C -1.34228600 5.89057200 0.71915300

C -0.67236200 5.47125300 1.87049200

H -0.88222700 2.11092300 1.42604500

H -2.34841400 5.29561900 -1.07904600

H 0.00971700 3.76044500 2.99503800

H -1.46621800 6.95050900 0.51330100

H -0.27215000 6.20154900 2.56853100

C -1.97455900 3.02862800 -2.38061100

C -0.64610100 2.86721600 -2.81334100

C -2.87866000 3.62693800 -3.26680400

C -0.24485300 3.26662800 -4.08559000

C -2.48022500 4.02366500 -4.54909200

C -1.16482700 3.84202100 -4.96648100

H 0.09141300 2.43496300 -2.14410800

H -3.90989300 3.80680800 -2.98083500

H 0.79264100 3.13613100 -4.38021600

H -3.20811000 4.47983800 -5.21517000

H -0.85547800 4.15206600 -5.96094100

N -2.26264900 -1.33953200 -0.34328600

C -2.42725100 -2.61932000 -1.07566600

C -2.27828600 -2.19331400 -2.60253500

N -2.79819800 -0.81037900 -2.63606300

H -2.13296900 -1.34960200 0.68873900

C -3.19359300 -0.19562300 -3.89644500

H -3.77606100 0.70558500 -3.69595600

H -2.33349800 0.09081700 -4.51142500

H -3.81603800 -0.89511000 -4.46731800

H -2.97706100 -2.80693700 -3.17631700

C -0.89172600 -2.39348000 -3.29713900

H -0.46863700 -3.30559400 -2.86402200

C 0.11626400 -1.25479700 -3.04622900

H -0.24606600 -0.32931100 -3.51480200

C 1.53055600 -1.55866100 -3.55767400

C -1.34086800 -3.62031200 -0.65292700

C -0.13391400 -3.19554900 -0.08495800

C -1.52382800 -4.98915000 -0.89924700

C 0.87110100 -4.11570100 0.21926500

C -0.52097900 -5.90859800 -0.59350200

C 0.68155000 -5.47434600 -0.03300700

H 0.03184700 -2.14338900 0.11225200

H -2.45854000 -5.34103300 -1.32591800

H 1.80376100 -3.75677600 0.64516100

H -0.68376200 -6.96520300 -0.79004000

H 1.46256100 -6.19180600 0.20582400

C -3.83077700 -3.19898300 -0.73895900

C -4.84484000 -3.45621000 -1.66906700

C -4.09738300 -3.47046500 0.61644500

C -6.08551500 -3.96540900 -1.26457700

C -5.33032700 -3.97730000 1.01858100

C -6.33412300 -4.22932700 0.07869100

H -4.69900500 -3.26993100 -2.72782800

H -3.33659800 -3.26705700 1.36470600

H -6.85190500 -4.15599200 -2.01169500

H -5.50423200 -4.17801500 2.07251900

H -7.29532600 -4.62757500 0.39251000

H 2.22654600 -0.76912700 -3.25927600

H 1.90652700 -2.49664400 -3.13207800

H 1.56625800 -1.64420800 -4.64998700

C 4.06238500 0.58668700 3.50042800

C 5.18572500 -0.25801900 3.49610800

C 4.13144500 1.76642600 4.26266400

C 6.32556500 0.05408500 4.23464800

H 5.15788800 -1.16401200 2.89882900

C 5.27377500 2.08318700 4.99683100

H 3.28642700 2.44784800 4.28510300

C 6.37623200 1.22689000 4.99003800

H 7.17829800 -0.62020100 4.21766900

H 5.30006800 3.00136700 5.57841700

H 7.26466500 1.47196600 5.56627000

C -1.07707700 -2.68630800 -4.80082000

H -1.41516600 -1.80862100 -5.36331800

H -0.13514600 -3.01600900 -5.24922700

H -1.80807900 -3.48861300 -4.96164300

H 0.18245000 -1.04954800 -1.97377600

H -3.15384300 1.43946300 1.80589200

C -5.96233800 3.10417200 0.55198500

H -6.59902700 2.22375000 0.41165500

H -6.31075400 3.61289300 1.45565000

H -6.14097100 3.78549500 -0.28969900

C -4.52117000 2.32007200 3.20882700

H -4.26833400 1.60055600 3.99180000

H -3.94506600 3.23613400 3.39168900

H -5.58324400 2.56483200 3.32142100

- **1da**-*RR* (EtEt-2)


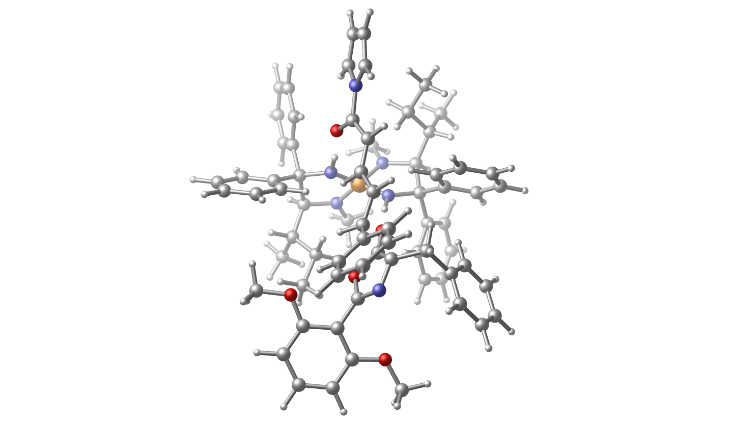

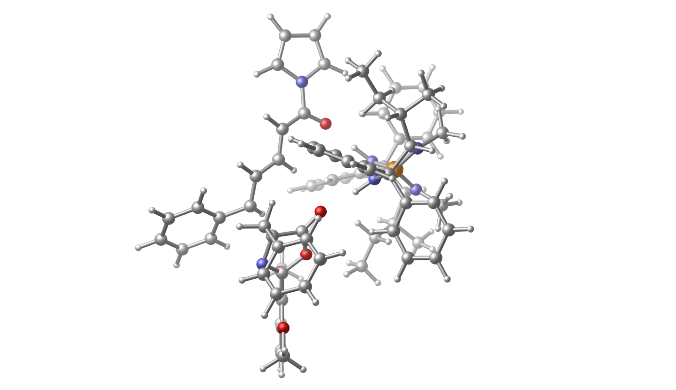


B3LYP/6-31G*

E = –3797.843424

Zero-point correction = 1.340920 (Hartree/Particle)

Thermal correction to Energy = 1.420005

Thermal correction to Enthalpy = 1.420949

Thermal correction to Gibbs Free Energy = 1.215465

Sum of electronic and zero-point Energies = –3796.502504

Sum of electronic and thermal Energies = –3796.423419

Sum of electronic and thermal Enthalpies = –3796.422475

Sum of electronic and thermal Free Energies = –3796.627959

PCM(toluene)-B3LYP-D3/6-311++G**//B3LYP/6-31G*@–30 °C

E = –3799.039127

G = –3797.788245

0 1

O 1.06260400 0.56539500 0.09875000

C 2.24127300 0.23209900 0.18389400

C 3.29399600 0.55371300 1.13238400

O 2.78601700 -0.71384500 -0.68101500

N 4.47957800 -0.05031200 0.65276600

C 4.13464600 -0.78631500 -0.34032300

C 4.98965500 -1.65576100 -1.16683100

C 4.76633500 -3.04475200 -1.21810300

C 6.05623200 -1.10721200 -1.90871700

C 5.58570000 -3.87506000 -1.99291700

C 6.88374000 -1.93494200 -2.68060600

C 6.63485700 -3.30489200 -2.71140300

O 3.73268200 -3.49894200 -0.45128700

C 3.39579700 -4.87716900 -0.51589900

H 4.20347800 -5.50895900 -0.12400800

H 3.16007200 -5.18339100 -1.54274200

H 2.50622300 -4.99432300 0.10472700

O 6.19094400 0.24096600 -1.83460000

C 7.24336700 0.86859100 -2.54769900

H 8.22601700 0.51318600 -2.21105200

H 7.15188300 0.70760600 -3.63032500

H 7.14589800 1.93305500 -2.32873000

H 5.42298400 -4.94505000 -2.02949200

H 7.70415600 -1.52113200 -3.25394600

H 7.27618700 -3.94635600 -3.31017000

C 3.39433700 1.92786500 1.75614500

H 2.40636200 2.20000300 2.14225600

H 4.08346400 1.86818200 2.60607400

C 3.88448200 2.98671200 0.78196600

C 2.97849600 3.78183600 0.06789600

C 5.25655700 3.17371600 0.56226300

C 3.43017200 4.74225100 -0.83969300

C 5.71066600 4.13669800 -0.33991000

C 4.79837000 4.92570900 -1.04531100

H 1.91225700 3.64682300 0.22501600

H 5.96785500 2.55180500 1.09894200

H 2.70873200 5.35008200 -1.38023400

H 6.77958300 4.27803500 -0.48518100

H 5.15174400 5.67964900 -1.74468800

C 2.66518800 -0.70563500 2.67821400

C 1.51153200 -0.19495200 3.34770600

H 2.46883400 -1.53556500 1.99984600

H 1.66232100 0.41790300 4.23438600

C 0.20555000 -0.50282500 3.00169600

H 0.04662900 -1.11790900 2.11819300

C -0.93646300 -0.15800200 3.74109500

H -0.80624800 0.40632900 4.65865000

C -2.23064800 -0.59184200 3.38916500

O -2.58886800 -1.11172700 2.30013400

N -3.26734500 -0.43077400 4.37299700

C -4.61608600 -0.54066300 4.07990400

C -3.13454800 -0.19212000 5.73347900

C -5.33131000 -0.35783200 5.23825000

C -4.38795100 -0.14070100 6.29056000

H -4.92671300 -0.72650600 3.06495600

H -2.16164000 -0.12258400 6.19267500

H -6.40973200 -0.37926500 5.32699600

H -4.60681300 0.02112100 7.33801900

H -0.58968000 0.95845300 -0.56637200

N -1.52341300 1.27197500 -0.85010600

P -2.66282400 0.23134400 -1.39434800

N -3.83080700 1.40228000 -1.50900200

C -3.40862900 2.75716900 -1.08996400

C -1.80740700 2.70045800 -1.08279100

C -5.18433000 1.17255900 -1.99806700

H -5.23920600 0.19056700 -2.47159200

H -5.92029900 1.19589700 -1.18795200

H -5.45188900 1.93724400 -2.73806800

H -3.70220700 3.42035500 -1.90696500

C -4.11922300 3.30893100 0.18412300

H -3.45545300 4.08463900 0.57989000

C -4.32726400 2.26780700 1.30196500

H -5.13237000 1.57673100 1.01575900

C -1.18742300 3.55128300 0.04320700

C -0.87837000 2.99224800 1.29090400

C -0.96712600 4.92475800 -0.14557800

C -0.37630500 3.78902700 2.32247400

C -0.47015800 5.71959100 0.88757800

C -0.17323200 5.15500800 2.12885300

H -1.01519800 1.93342400 1.47185600

H -1.17852900 5.38100000 -1.10713900

H -0.14363100 3.32608500 3.27720500

H -0.30758200 6.78035900 0.71592700

H 0.22080000 5.77191800 2.93175400

C -1.20322200 3.12543800 -2.45277200

C 0.03726000 2.58356300 -2.83106000

C -1.79110500 4.06540300 -3.31107700

C 0.64912500 2.94629900 -4.02981000

C -1.18108400 4.42793400 -4.51623800

C 0.03888600 3.86625600 -4.88494400

H 0.54086900 1.87718500 -2.17943700

H -2.72876300 4.54760200 -3.05246500

H 1.61015000 2.51080900 -4.28939700

H -1.66662100 5.15548900 -5.16154000

H 0.51326900 4.14712100 -5.82129700

N -2.57928400 -1.10747600 -0.46048600

C -3.01238700 -2.34992500 -1.13876500

C -2.60900600 -2.08866200 -2.65668200

N -2.74407900 -0.62421900 -2.81583400

H -2.54414400 -1.04770500 0.57393800

C -2.81155900 -0.01605700 -4.13852200

H -3.13902300 1.02199100 -4.04616900

H -1.84071300 -0.01738800 -4.64524200

H -3.53386100 -0.55705300 -4.76167100

H -3.38177000 -2.54146400 -3.28269000

C -1.25934600 -2.68539800 -3.16629700

H -1.15185500 -3.65158300 -2.66096900

C -0.01933200 -1.84211600 -2.81455200

H -0.08323900 -0.86400700 -3.31114800

C 1.30842400 -2.50905500 -3.19402900

C -2.30223100 -3.57495500 -0.54022900

C -1.18382300 -3.45118000 0.29018300

C -2.77784900 -4.86233000 -0.83682800

C -0.56068000 -4.58499200 0.81777400

C -2.14497100 -5.99374700 -0.32574000

C -1.03398800 -5.85945500 0.51015900

H -0.80350900 -2.46927600 0.54091100

H -3.65935100 -4.98155100 -1.46046000

H 0.28806100 -4.46115400 1.48531900

H -2.53112200 -6.98030300 -0.56818400

H -0.55247100 -6.74022500 0.92668100

C -4.54572700 -2.52017400 -0.92881900

C -5.48640800 -2.71802800 -1.94616000

C -5.00925200 -2.48515600 0.39985200

C -6.84824500 -2.86360600 -1.65210500

C -6.36232700 -2.63254400 0.69221500

C -7.29261700 -2.82080300 -0.33424400

H -5.18767300 -2.76717800 -2.98817300

H -4.30318900 -2.33622700 1.21248200

H -7.55506100 -3.01436200 -2.46414800

H -6.68895900 -2.60539600 1.72849800

H -8.34863600 -2.93707400 -0.10588100

H 2.14574800 -1.91857300 -2.81597700

H 1.38301400 -3.51060900 -2.75174100

H 1.42575800 -2.61200200 -4.27860600

C 3.92790900 -0.89938000 3.44282900

C 4.77666600 -1.96927700 3.10958400

C 4.30465000 -0.07246400 4.51610100

C 5.94880000 -2.21004600 3.82279800

H 4.50855000 -2.61501700 2.27819600

C 5.48113900 -0.30788300 5.22616000

H 3.67352200 0.76211700 4.80548900

C 6.30853200 -1.37944700 4.88552800

H 6.58441000 -3.04739700 3.54576500

H 5.74892300 0.34644600 6.05208500

H 7.22295300 -1.56493000 5.44307800

C -1.34096400 -3.00596800 -4.67375500

H -1.33936900 -2.10526500 -5.29761800

H -0.48931800 -3.61750500 -4.98560600

H -2.24921500 -3.57454600 -4.90979100

H -0.00596200 -1.63486000 -1.73932500

H -3.42854200 1.65204600 1.41067300

C -5.43277700 4.02773600 -0.18809400

H -6.20618100 3.33643500 -0.54077700

H -5.84200200 4.55730900 0.67753900

H -5.26860800 4.77518200 -0.97460600

C -4.65366300 2.89725600 2.66255700

H -4.79041200 2.12298300 3.42324400

H -3.83874100 3.55338000 2.99133400

H -5.57225000 3.49433100 2.63066200

- **1da**-*RR* (EtH)


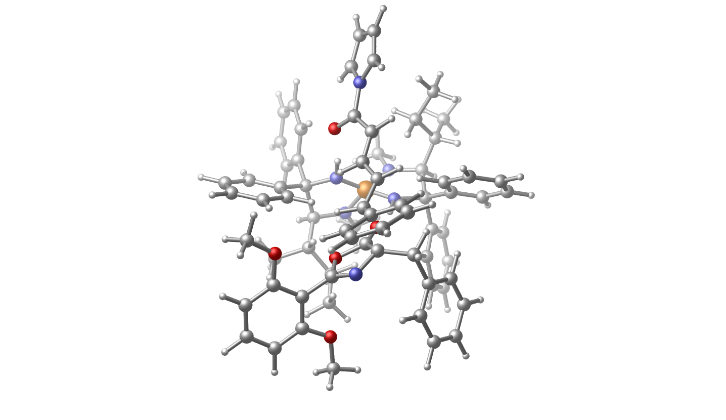

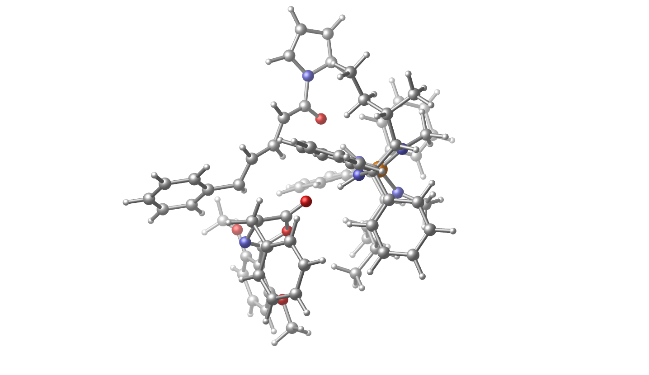


B3LYP/6-31G*

E = –3797.845098

Zero-point correction = 1.341503 (Hartree/Particle)

Thermal correction to Energy = 1.420145

Thermal correction to Enthalpy = 1.421089

Thermal correction to Gibbs Free Energy = 1.219545

Sum of electronic and zero-point Energies = –3796.503595

Sum of electronic and thermal Energies = –3796.424952

Sum of electronic and thermal Enthalpies = –3796.424008

Sum of electronic and thermal Free Energies = –3796.625553

PCM(toluene)-B3LYP-D3/6-311++G**//B3LYP/6-31G*@–30 °C

E = –3799.043533

G = –3797.789296

0 1

O 0.91128900 0.74917700 0.05292800

C 2.09730900 0.46490800 0.14603600

C 3.18844300 1.02686600 0.92470800

O 2.61831200 -0.66845900 -0.48257400

N 4.36788600 0.33768800 0.52816300

C 3.98374800 -0.63163200 -0.21784200

C 4.81289000 -1.67618300 -0.85356000

C 5.11862100 -2.87219900 -0.18280100

C 5.32138000 -1.46698300 -2.14836300

C 5.93228500 -3.83987500 -0.78639700

C 6.13424600 -2.42961400 -2.76064300

C 6.42761100 -3.60157400 -2.06680300

O 4.57356200 -3.00860400 1.06351200

C 4.89646900 -4.16830000 1.82112200

H 5.97718900 -4.24403800 1.99361100

H 4.54128700 -5.08134200 1.32695500

H 4.38327800 -4.04924300 2.77641100

O 4.96392700 -0.28714600 -2.72495600

C 5.50386900 0.04553600 -3.99487700

H 6.60084900 0.06899800 -3.96926600

H 5.17293200 -0.65674100 -4.77082800

H 5.12423000 1.04305400 -4.22124500

H 6.17669700 -4.76361500 -0.27704000

H 6.53146400 -2.27464100 -3.75626100

H 7.05793600 -4.35150300 -2.53725900

C 3.26159800 2.52377800 1.14123100

H 2.33418000 2.84252300 1.63049900

H 4.08617600 2.72641100 1.83326200

C 3.45726500 3.32152500 -0.13889100

C 2.45330200 4.17746700 -0.60940300

C 4.64859000 3.22046800 -0.87438200

C 2.63058700 4.91616600 -1.78283300

C 4.82762100 3.95672300 -2.04534600

C 3.81930000 4.80845700 -2.50551900

H 1.52663300 4.27151400 -0.04819600

H 5.42945200 2.55086600 -0.52558800

H 1.83895400 5.57757000 -2.12609200

H 5.76158400 3.87206800 -2.59643300

H 3.96328400 5.38797200 -3.41423900

C 2.76156400 0.18813500 2.73584900

C 1.45914700 0.50608200 3.24196800

H 2.85262500 -0.82271900 2.33810000

H 1.36497600 1.33271500 3.94335300

C 0.32635800 -0.25199200 2.99018900

H 0.41219300 -1.07900700 2.28646600

C -0.91204800 -0.12443000 3.63878200

H -1.01530600 0.61140200 4.42958700

C -1.99398200 -0.98675500 3.36391300

O -2.12524300 -1.74380000 2.36726800

N -3.07035900 -1.01331700 4.31799700

C -4.31882800 -1.54551100 4.04722800

C -3.06246500 -0.56357300 5.63079200

C -5.10030300 -1.42135100 5.16935100

C -4.29945200 -0.80110600 6.17834300

H -4.52668500 -1.94552000 3.06872100

H -2.16478200 -0.16108400 6.07202400

H -6.13034200 -1.73999700 5.26278000

H -4.59485800 -0.57090600 7.19378800

H -0.77168400 1.14894900 -0.49139300

N -1.77826400 1.21883500 -0.65799800

P -2.61937400 -0.08203900 -1.19063700

N -4.07530500 0.71113000 -1.14619600

C -3.99641400 2.13022200 -0.72003700

C -2.45318300 2.50931900 -0.88340500

C -5.35722800 0.08304200 -1.44574000

H -5.18992000 -0.85640800 -1.97736900

H -5.92415900 -0.14462200 -0.53733900

H -5.96067800 0.74227300 -2.08158000

H -4.55630100 2.70082300 -1.46539600

C -4.66834400 2.46841900 0.64339200

H -4.27237500 3.45435900 0.91312700

C -4.32136600 1.50578000 1.79405700

H -4.81482900 0.53813600 1.63015200

C -1.97246400 3.55839100 0.13446000

C -1.25199500 3.20196400 1.28142000

C -2.26676500 4.91480500 -0.07878900

C -0.84844600 4.18003600 2.19568400

C -1.87131700 5.88647500 0.83851000

C -1.15798800 5.52243000 1.98276900

H -0.99296400 2.16733400 1.47353600

H -2.80242800 5.21682700 -0.97414300

H -0.28866800 3.87926600 3.07701000

H -2.11347000 6.92967000 0.65326500

H -0.84235200 6.27917600 2.69584200

C -2.09887800 3.02123900 -2.31234100

C -0.73881300 3.02249300 -2.67197800

C -3.01753900 3.52004900 -3.24349500

C -0.31799800 3.48866500 -3.91466900

C -2.59838800 3.98413000 -4.49635000

C -1.24968700 3.96897800 -4.83927500

H 0.00786600 2.66590800 -1.96851200

H -4.07751200 3.56885900 -3.01675000

H 0.74249300 3.48514500 -4.15065900

H -3.33764800 4.36146300 -5.19831800

H -0.92492000 4.33256000 -5.81040900

N -2.10930200 -1.39640200 -0.36637300

C -2.15035100 -2.65601400 -1.15671600

C -1.79892800 -2.12481800 -2.61786300

N -2.51306600 -0.82665700 -2.67012200

H -2.02344700 -1.42836100 0.67024500

C -3.11506000 -0.36168400 -3.91316800

H -3.70207300 0.53740200 -3.71972600

H -2.36183400 -0.11473500 -4.66586200

H -3.78039700 -1.13427200 -4.32081300

H -2.27347200 -2.76734500 -3.36376300

C -0.27731000 -2.05375600 -2.97668000

H 0.28514900 -1.84961400 -2.05978800

C 0.07236200 -0.91325000 -3.95851700

H -0.40834400 -1.09767100 -4.93085900

C 1.58023400 -0.72761300 -4.17619500

C -1.12846600 -3.65043700 -0.58001400

C 0.04505000 -3.19899600 0.03901800

C -1.33438200 -5.03355800 -0.68997100

C 0.99712900 -4.10253800 0.51163200

C -0.38609200 -5.93787300 -0.21080700

C 0.78556800 -5.47580100 0.38932900

H 0.22267400 -2.13584800 0.14899100

H -2.24219900 -5.41205000 -1.14808400

H 1.90693900 -3.72064000 0.96580700

H -0.56940100 -7.00511900 -0.30470100

H 1.52315400 -6.18156000 0.76296300

C -3.57816300 -3.25923800 -1.07204000

C -4.29335700 -3.76717000 -2.16384700

C -4.17517800 -3.33353200 0.19957700

C -5.57539600 -4.30551700 -2.00014000

C -5.44784300 -3.87597100 0.36358500

C -6.15993000 -4.35935000 -0.73759800

H -3.86727500 -3.76453200 -3.16176400

H -3.63158600 -2.97000000 1.06748400

H -6.10839900 -4.68697200 -2.86740100

H -5.88236200 -3.92625200 1.35869500

H -7.15375700 -4.77986800 -0.60939000

H 1.76523600 0.15403700 -4.80131300

H 2.10469100 -0.58093300 -3.22601400

H 2.03399700 -1.58722300 -4.68175200

C 3.95819700 0.57151000 3.54425300

C 5.09943700 -0.24855600 3.52000300

C 3.99112300 1.71708000 4.35917700

C 6.22129200 0.05377000 4.28962600

H 5.10108600 -1.12537500 2.88041500

C 5.11535700 2.02414100 5.12473600

H 3.13183500 2.37940400 4.39923800

C 6.23579700 1.19201600 5.09751700

H 7.08863200 -0.60100900 4.25556100

H 5.11322000 2.91572700 5.74688100

H 7.11009600 1.42925600 5.69814000

C 0.18089800 -3.40895900 -3.54535600

H -0.26715400 -3.57887000 -4.53486200

H 1.26782900 -3.43766100 -3.66333000

H -0.09722900 -4.24579700 -2.90146600

H -0.33447700 0.02954000 -3.57724800

H -3.24605200 1.30229800 1.79239700

C -6.19120800 2.66202900 0.48696800

H -6.72475000 1.71645300 0.34572100

H -6.60951000 3.13763200 1.37899400

H -6.42726000 3.31214000 -0.36522600

C -4.71004500 2.05091600 3.17459800

H -4.40234100 1.36192700 3.96522300

H -4.22345400 3.01710100 3.35944400

H -5.79171200 2.19684400 3.27180900

- **1da**-*RR* (EtH-2)


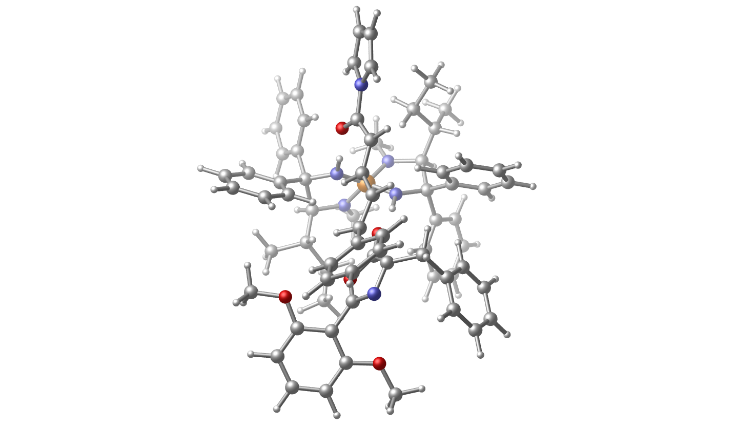

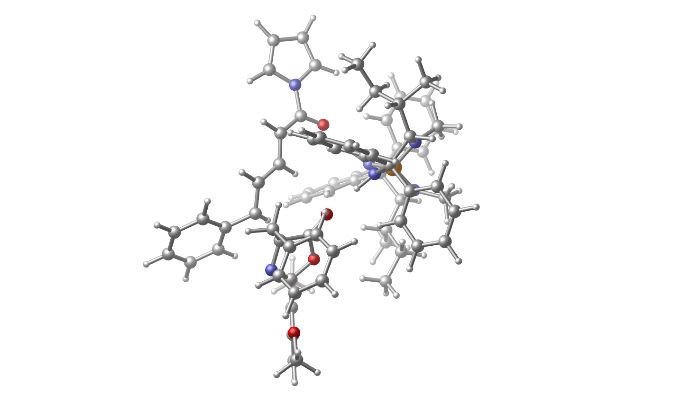


B3LYP/6-31G*

E = –3797.843499

Zero-point correction = 1.341846 (Hartree/Particle)

Thermal correction to Energy = 1.420409

Thermal correction to Enthalpy = 1.421354

Thermal correction to Gibbs Free Energy = 1.218632

Sum of electronic and zero-point Energies = –3796.501653

Sum of electronic and thermal Energies = –3796.423090

Sum of electronic and thermal Enthalpies = –3796.422145

Sum of electronic and thermal Free Energies = –3796.624867

PCM(toluene)-B3LYP-D3/6-311++G**//B3LYP/6-31G*@–30 °C

E = –3799.039126

G = –3797.785582

0 1

O 0.94909800 0.45434700 0.02304100

C 2.15080900 0.25635900 0.17300300

C 3.11168200 0.66864800 1.18528100

O 2.83641500 -0.61113700 -0.67217400

N 4.37985200 0.20566700 0.75541400

C 4.16801000 -0.54232000 -0.26546900

C 5.14882900 -1.29037500 -1.06953900

C 5.04366600 -2.68741800 -1.21594100

C 6.21027400 -0.61567200 -1.70923000

C 5.97468900 -3.40094900 -1.98093000

C 7.14825700 -1.32744700 -2.47017200

C 7.01568700 -2.70791300 -2.59500700

O 4.00540500 -3.27091400 -0.55023200

C 3.76865600 -4.65767100 -0.74952400

H 4.58756900 -5.26860300 -0.34777300

H 3.63192800 -4.88989100 -1.81321100

H 2.84610800 -4.88174700 -0.21257400

O 6.22990100 0.73271300 -1.55688900

C 7.23614900 1.48465400 -2.21448000

H 8.23872500 1.22250000 -1.85193100

H 7.19748500 1.34733400 -3.30334000

H 7.02227200 2.52685900 -1.97264500

H 5.90344000 -4.47611000 -2.08779000

H 7.96562400 -0.81644900 -2.96347900

H 7.74338700 -3.25907400 -3.18485000

C 3.03208600 2.03926900 1.82256300

H 2.01136400 2.18276800 2.19269100

H 3.70579000 2.04896700 2.68722000

C 3.40771100 3.16700000 0.87592200

C 2.42153400 3.93001600 0.23766000

C 4.75429500 3.45686700 0.61089200

C 2.76832700 4.95706200 -0.64333400

C 5.10306600 4.48758800 -0.26212300

C 4.11097900 5.24159000 -0.89480100

H 1.37377900 3.72098000 0.43573600

H 5.52684700 2.86000000 1.08737900

H 1.98535400 5.53602800 -1.12660700

H 6.15287500 4.70968800 -0.44218900

H 4.38340500 6.04683900 -1.57279300

C 2.54019100 -0.64251300 2.67401900

C 1.34765300 -0.21141600 3.33792300

H 2.39700600 -1.47783200 1.98849000

H 1.45252100 0.39973400 4.23233300

C 0.06768200 -0.59645400 2.97390900

H -0.04250900 -1.21455300 2.08661200

C -1.10470800 -0.34149800 3.70192900

H -1.02365900 0.20177200 4.63746200

C -2.36034300 -0.86369000 3.32434000

O -2.67647500 -1.36009600 2.21172300

N -3.40619500 -0.83516700 4.31133800

C -4.73340100 -1.10214800 4.02074000

C -3.29828700 -0.60021700 5.67531400

C -5.46103600 -1.02148500 5.18250900

C -4.54666200 -0.70474500 6.23527500

H -5.02201000 -1.32179700 3.00630500

H -2.34017000 -0.41962700 6.13477500

H -6.52816900 -1.17697300 5.27378200

H -4.77952000 -0.58172000 7.28499700

H -0.68121000 1.09871800 -0.41843400

N -1.64067000 1.28683600 -0.72098400

P -2.55094300 0.10469400 -1.39034600

N -3.89553000 1.06488500 -1.52242000

C -3.72223800 2.45770300 -1.04522300

C -2.13331500 2.65597000 -0.93091500

C -5.17735500 0.62239500 -2.05813900

H -5.05670200 -0.34495800 -2.54966100

H -5.92755200 0.50320500 -1.27015000

H -5.55014200 1.34624200 -2.79376400

H -4.06319600 3.09141800 -1.86780600

C -4.58521700 2.85291800 0.19089300

H -4.07499200 3.71222900 0.64010100

C -4.69011000 1.76357700 1.27669100

H -5.37529300 0.97362100 0.93887100

C -1.70652900 3.53578100 0.26381500

C -1.37772500 2.95817200 1.49945400

C -1.65980200 4.93420900 0.15160700

C -1.02726400 3.75720600 2.59010000

C -1.31421000 5.73068900 1.24369300

C -0.99629500 5.14604100 2.46999400

H -1.38676700 1.88195300 1.62616800

H -1.88472200 5.41254200 -0.79553900

H -0.77719600 3.27887500 3.53282600

H -1.28495300 6.81101600 1.12880700

H -0.71982800 5.76593800 3.31841900

C -1.54496900 3.23305700 -2.25046300

C -0.26303600 2.83143900 -2.65999900

C -2.20661200 4.19311900 -3.03108900

C 0.32377800 3.35396500 -3.81267000

C -1.62255600 4.71534600 -4.18827100

C -0.35543100 4.29504100 -4.58780200

H 0.29284800 2.10701500 -2.07431400

H -3.18597500 4.56365500 -2.74293200

H 1.31897800 3.02444200 -4.09808700

H -2.16431500 5.45450300 -4.77270700

H 0.09994700 4.69956900 -5.48759900

N -2.31240300 -1.25677100 -0.52068100

C -2.45837100 -2.50564000 -1.30797000

C -1.87112700 -2.05837200 -2.72355800

N -2.34891900 -0.65736300 -2.85049600

H -2.34105700 -1.26497000 0.51735400

C -2.73383000 -0.11628100 -4.14938300

H -3.17059400 0.87501900 -4.01459300

H -1.87663000 -0.01724100 -4.81900900

H -3.47978900 -0.76589200 -4.62644000

H -2.36931800 -2.62021100 -3.51789800

C -0.33345300 -2.24788800 -2.92521700

H 0.16644500 -2.07399200 -1.96598400

C 0.27363900 -1.23865600 -3.92543900

H -0.17935700 -1.38714300 -4.91723000

C 1.79960100 -1.32686800 -4.05609600

C -1.68351000 -3.63115000 -0.59693100

C -0.55112200 -3.34228000 0.17653600

C -2.09945400 -4.96789700 -0.69093100

C 0.13894900 -4.35639300 0.84227000

C -1.40075900 -5.98399300 -0.03853200

C -0.28095100 -5.68256800 0.73741200

H -0.20964000 -2.31788300 0.26762400

H -2.98233300 -5.22172600 -1.26768300

H 0.99568300 -4.09899700 1.45942600

H -1.74618700 -7.01081300 -0.12498200

H 0.24904600 -6.47079300 1.26557100

C -3.96257200 -2.88324200 -1.39417400

C -4.57448700 -3.41672600 -2.53644400

C -4.74704100 -2.72757000 -0.23759500

C -5.93257100 -3.75394400 -2.53714000

C -6.09821800 -3.06933500 -0.23643400

C -6.70173400 -3.57728300 -1.38943300

H -4.00512700 -3.59274900 -3.44368400

H -4.29192300 -2.34377800 0.67111400

H -6.38141000 -4.15988200 -3.44012000

H -6.68000600 -2.94269300 0.67287500

H -7.75611200 -3.84024300 -1.38887900

H 2.16886200 -0.53006900 -4.71247400

H 2.28429800 -1.20893100 -3.08180700

H 2.12560200 -2.27879800 -4.48876400

C 3.79650100 -0.79039300 3.46286300

C 4.69866900 -1.81663500 3.13394800

C 4.11453300 0.03929800 4.55232000

C 5.86703500 -2.01315400 3.86688600

H 4.47721800 -2.46323800 2.28914500

C 5.28691900 -0.15135800 5.28221300

H 3.44022400 0.84050000 4.83848800

C 6.16851500 -1.18017500 4.94560600

H 6.54510000 -2.81744700 3.59257500

H 5.50914100 0.50417300 6.12060500

H 7.07968600 -1.33096400 5.51869900

C -0.04864500 -3.68882000 -3.38690600

H -0.41758700 -3.84104800 -4.41116600

H 1.02586300 -3.89295000 -3.38947000

H -0.52014300 -4.43526900 -2.74502700

H 0.01700900 -0.22272900 -3.60631500

H -3.72008400 1.27600400 1.41521800

C -5.97136500 3.36968800 -0.24655500

H -6.59986400 2.58058800 -0.67358200

H -6.51340800 3.79060600 0.60575600

H -5.88071700 4.16615900 -0.99599600

C -5.16375400 2.30585700 2.63157300

H -5.21913200 1.50211200 3.37165700

H -4.46841800 3.06513700 3.00954300

H -6.15711700 2.76490100 2.56717200

- **1da**-*RR* (HEt)


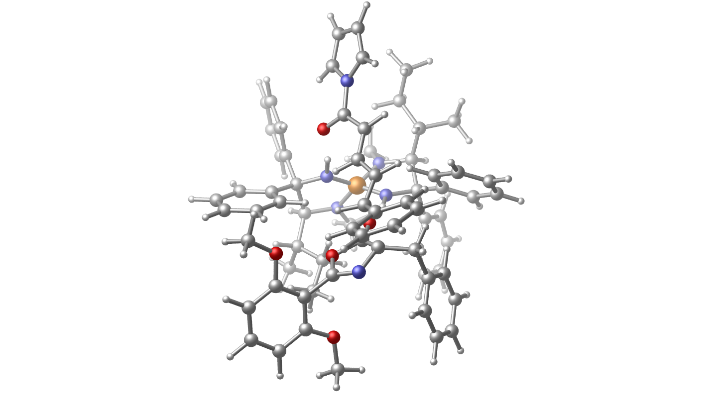

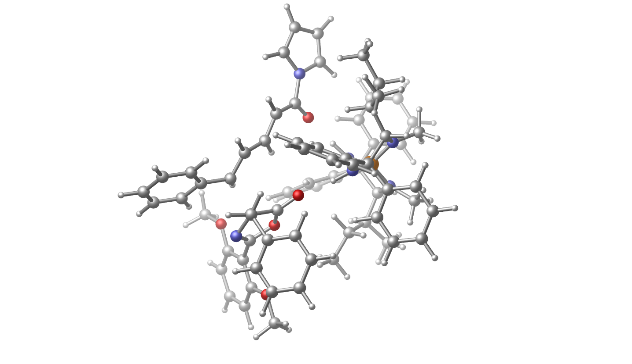


B3LYP/6-31G*

E = –3797.844594

Zero-point correction = 1.341977 (Hartree/Particle)

Thermal correction to Energy = 1.420299

Thermal correction to Enthalpy = 1.421244

Thermal correction to Gibbs Free Energy = 1.222278

Sum of electronic and zero-point Energies = –3796.502617

Sum of electronic and thermal Energies = –3796.424294

Sum of electronic and thermal Enthalpies = –3796.423350

Sum of electronic and thermal Free Energies = –3796.622316

PCM(toluene)-B3LYP-D3/6-311++G**//B3LYP/6-31G*@–30 °C

E = –3799.043217

G = –3797.786721

0 1

O 0.94247300 0.59916800 0.04108300

C 2.13728000 0.35190500 0.14112200

C 3.22645200 0.99160300 0.85931900

O 2.67577800 -0.81435000 -0.40582900

N 4.41595600 0.30488100 0.49377900

C 4.04264800 -0.72432300 -0.17359500

C 4.88725600 -1.78577200 -0.75875200

C 5.13613700 -2.98117700 -0.06483100

C 5.46539800 -1.59530100 -2.02693100

C 5.96502200 -3.96701900 -0.61530200

C 6.29510600 -2.57615600 -2.58642700

C 6.53289100 -3.74629500 -1.86878900

O 4.52117000 -3.09500300 1.15126900

C 4.78111200 -4.25086100 1.93836800

H 5.84908600 -4.34264300 2.17182900

H 4.43838000 -5.16395200 1.43557200

H 4.21681200 -4.11234500 2.86159000

O 5.15217600 -0.41933000 -2.63531000

C 5.75798400 -0.10918000 -3.88075700

H 6.85265000 -0.09866100 -3.80190400

H 5.45689000 -0.81750800 -4.66355300

H 5.40256400 0.89028800 -4.13619900

H 6.16690000 -4.88966000 -0.08559000

H 6.74609200 -2.43696100 -3.56130100

H 7.17605700 -4.50945100 -2.29892400

C 3.27500600 2.50011300 0.97665800

H 2.33632300 2.84014500 1.42716900

H 4.08782000 2.76326600 1.66198600

C 3.48312200 3.19479800 -0.36006100

C 2.43089700 3.86974000 -0.99265800

C 4.73252400 3.15743700 -0.99763400

C 2.61869100 4.49249600 -2.22946100

C 4.92290400 3.78040000 -2.23123500

C 3.86605900 4.45079300 -2.85343300

H 1.45827400 3.91368000 -0.50989700

H 5.55169500 2.62445600 -0.52342800

H 1.78798200 5.01361800 -2.69946800

H 5.90191200 3.74967500 -2.70439300

H 4.01680200 4.94152500 -3.81204400

C 2.82700400 0.24117500 2.72731700

C 1.52748200 0.57538900 3.22789600

H 2.92206100 -0.78533300 2.37356700

H 1.43748600 1.42043800 3.90779100

C 0.39209700 -0.18197800 2.98682500

H 0.47859000 -1.02588100 2.30403600

C -0.85493900 -0.03198000 3.61449100

H -0.96645700 0.71977900 4.38908400

C -1.92261000 -0.91399200 3.34542200

O -2.01513000 -1.70264900 2.36921100

N -3.01833800 -0.93689300 4.27773400

C -4.20703200 -1.60341500 4.03505700

C -3.06918300 -0.40632500 5.56015800

C -5.00783900 -1.48733100 5.14437500

C -4.28326900 -0.72746500 6.11492900

H -4.36245200 -2.08547700 3.08422400

H -2.22157900 0.10912200 5.98208200

H -6.00225100 -1.90044900 5.25359500

H -4.61224600 -0.45997700 7.11076600

H -0.73331900 1.11506200 -0.48485300

N -1.72503900 1.23600300 -0.70462900

P -2.67227500 -0.01927800 -1.16556900

N -4.07394800 0.85316000 -0.98816700

C -3.87720000 2.21632500 -0.42367100

C -2.36605400 2.56360000 -0.81619300

C -5.38063100 0.44868000 -1.49806900

H -5.29058100 -0.51554300 -2.00101000

H -6.11217800 0.34029600 -0.69449700

H -5.75632100 1.18856100 -2.21777400

H -4.55491600 2.86475200 -0.98519400

C -4.23699200 2.38785600 1.08535300

H -3.34378200 2.16500700 1.67992200

C -5.34073200 1.42988500 1.58143700

H -6.26950800 1.61368400 1.02176000

C -5.63627700 1.55920700 3.08111200

C -1.65180200 3.55363800 0.12538900

C -1.03141800 3.11018900 1.30270200

C -1.60646500 4.92513900 -0.17336400

C -0.40682600 4.01622800 2.16284400

C -0.98337900 5.82828800 0.68808400

C -0.38133700 5.37775000 1.86346700

H -1.01921200 2.05698700 1.55917800

H -2.05698600 5.29601100 -1.08766900

H 0.06669100 3.64258800 3.06610100

H -0.96284000 6.88423900 0.43205600

H 0.11043600 6.07912200 2.53197300

C -2.24393500 3.06809000 -2.28068100

C -1.02865600 2.85520400 -2.95343400

C -3.24594600 3.78058400 -2.95250500

C -0.83191000 3.31552500 -4.25414000

C -3.05470500 4.23690000 -4.26136900

C -1.84979800 4.00298600 -4.92001600

H -0.22231400 2.33051600 -2.45094700

H -4.18891600 4.00812700 -2.46598400

H 0.12231700 3.13950200 -4.74335800

H -3.85339500 4.78118700 -4.75861500

H -1.70054100 4.35882800 -5.93578300

N -2.17371800 -1.35986700 -0.37962900

C -2.33724500 -2.61665800 -1.15018900

C -2.20762800 -2.13999600 -2.66358500

N -2.74588300 -0.76147800 -2.64990100

H -2.06069100 -1.39927500 0.65354000

C -3.13059100 -0.10301300 -3.89211100

H -3.67976900 0.81344200 -3.66918300

H -2.26456600 0.17098600 -4.50319200

H -3.77877000 -0.76706600 -4.47701200

H -2.90264000 -2.74463900 -3.25158700

C -0.82270800 -2.30303800 -3.37093800

H -0.38897600 -3.22294500 -2.96605200

C 0.17618100 -1.16394900 -3.08824000

H -0.19105600 -0.23075500 -3.53788000

C 1.59502700 -1.44711600 -3.59924600

C -1.23955800 -3.62114300 -0.76670600

C -0.03753200 -3.20255100 -0.18399700

C -1.40794800 -4.98280400 -1.05945800

C 0.97712400 -4.12164300 0.09060600

C -0.39504100 -5.90086500 -0.78445500

C 0.80229800 -5.47282500 -0.20778100

H 0.11748600 -2.15516400 0.04518700

H -2.33890200 -5.33016900 -1.49803900

H 1.90499400 -3.76672300 0.53012900

H -0.54580100 -6.95189300 -1.01721900

H 1.59094600 -6.18948900 0.00712800

C -3.73280700 -3.21957500 -0.82122100

C -4.75824900 -3.43887800 -1.74859500

C -3.97918100 -3.55808500 0.52276200

C -5.98929000 -3.97726200 -1.35290200

C -5.20202900 -4.09532300 0.91610200

C -6.21686400 -4.30950700 -0.02119500

H -4.62820200 -3.20035700 -2.79881000

H -3.21016600 -3.38411600 1.26959700

H -6.76457400 -4.13719200 -2.09801500

H -5.35977800 -4.35129000 1.96061000

H -7.17013600 -4.73144300 0.28567600

H 2.28588200 -0.66262300 -3.27621200

H 1.97281700 -2.39409000 -3.19585300

H 1.63834000 -1.50422900 -4.69322600

H -6.32482400 0.77269400 3.40353700

H -4.72490700 1.44903200 3.67890300

H -6.09300300 2.52355000 3.32836400

C 4.02940100 0.67289000 3.49865800

C 5.16811800 -0.15078900 3.51963900

C 4.07152900 1.86937100 4.23633300

C 6.29658900 0.19736100 4.25942500

H 5.16119200 -1.06977900 2.94175200

C 5.20237200 2.22210000 4.97187300

H 3.21415500 2.53560800 4.23777700

C 6.32028700 1.38617700 4.99071900

H 7.16154800 -0.46137400 4.26240500

H 5.20773400 3.15252900 5.53429300

H 7.19977900 1.65939000 5.56802000

C -1.00826600 -2.55559700 -4.88198300

H -1.35513300 -1.66655200 -5.42078600

H -0.06357900 -2.86397700 -5.33982700

H -1.73161200 -3.36026200 -5.06415200

H 0.23459600 -0.98250900 -2.01137500

H -5.03864600 0.39701100 1.37378000

C -4.64798300 3.85071900 1.34003900

H -5.62761900 4.05503400 0.88527300

H -4.73100900 4.05494100 2.41083400

H -3.93082700 4.56478400 0.93012800

- **1da**-*RR* (HEt-2)


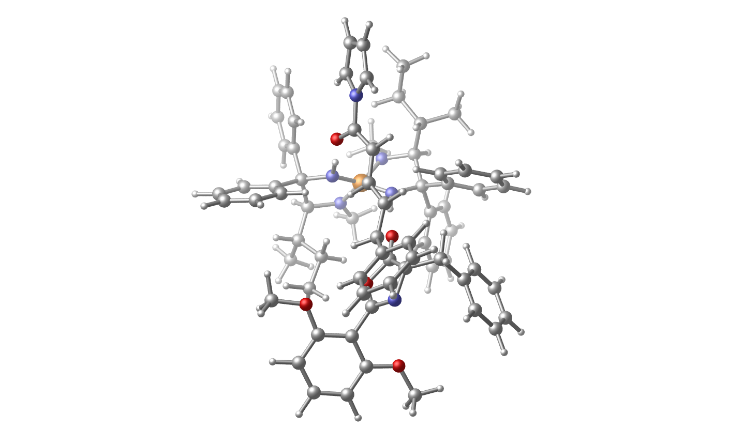

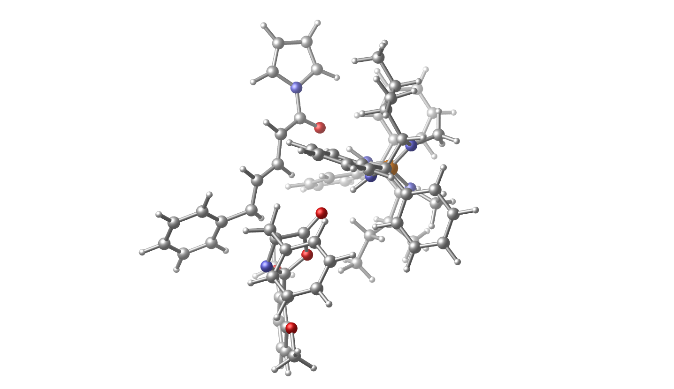


B3LYP/6-31G*

E = –3797.842054

Zero-point correction = 1.341586 (Hartree/Particle)

Thermal correction to Energy = 1.420183

Thermal correction to Enthalpy = 1.421128

Thermal correction to Gibbs Free Energy = 1.218103

Sum of electronic and zero-point Energies = –3796.500469

Sum of electronic and thermal Energies = –3796.421871

Sum of electronic and thermal Enthalpies = –3796.420927

Sum of electronic and thermal Free Energies = –3796.623951

PCM(toluene)-B3LYP-D3/6-311++G**//B3LYP/6-31G*@–30 °C

E = –3799.036733

G = –3797.783664

0 1

O 1.15781800 0.63130300 -0.01639400

C 2.32347400 0.26641400 0.10043500

C 3.40116500 0.67139600 0.98390000

O 2.82451000 -0.80398000 -0.63923400

N 4.56122400 -0.02215700 0.56952900

C 4.17534500 -0.87283700 -0.31063800

C 4.98593500 -1.88232200 -1.01441500

C 4.74292000 -3.25509400 -0.82200800

C 6.02662200 -1.48581900 -1.87820300

C 5.51764100 -4.21951800 -1.47832000

C 6.80957500 -2.44674700 -2.53344800

C 6.54159300 -3.79751300 -2.32435400

O 3.73616600 -3.55212300 0.05104700

C 3.37303800 -4.91321800 0.23040200

H 4.18648600 -5.49074600 0.68843200

H 3.08657400 -5.37877700 -0.72078500

H 2.51144300 -4.90433900 0.89987700

O 6.18177000 -0.14637700 -2.03208800

C 7.21973300 0.33576400 -2.86882400

H 8.20708100 0.01531400 -2.51149500

H 7.08737500 0.00782400 -3.90867600

H 7.14994200 1.42353500 -2.81848100

H 5.33974500 -5.27723600 -1.32858000

H 7.60990400 -2.15125400 -3.20068500

H 7.14816800 -4.54235500 -2.83286400

C 3.53938500 2.10570700 1.43962200

H 2.56136900 2.44437600 1.79795500

H 4.23749300 2.13471600 2.28360200

C 4.03970600 3.02291600 0.33583000

C 3.14141700 3.71665300 -0.48605300

C 5.41345200 3.16687600 0.09658300

C 3.60308200 4.53487300 -1.51896300

C 5.87765700 3.98816100 -0.93184000

C 4.97331200 4.67581100 -1.74498300

H 2.07381000 3.61322700 -0.31469100

H 6.11870800 2.62128700 0.71775000

H 2.88835200 5.06597800 -2.14315900

H 6.94789800 4.09702300 -1.09371700

H 5.33413600 5.31905500 -2.54403200

C 2.77234500 -0.40768200 2.67516400

C 1.60221000 0.14779100 3.27420600

H 2.60571700 -1.31299000 2.09239600

H 1.73301400 0.85729200 4.08917500

C 0.30408900 -0.22651600 2.96461000

H 0.15762600 -0.93578100 2.15131900

C -0.83797000 0.17722800 3.67458100

H -0.70370500 0.84634300 4.51819300

C -2.13140900 -0.31598800 3.40991500

O -2.50792300 -0.97779300 2.40718600

N -3.14427200 -0.04113100 4.39414400

C -4.49937700 -0.18008200 4.14982800

C -2.97705900 0.34591300 5.71653400

C -5.18546900 0.13172600 5.29804600

C -4.21616500 0.46119800 6.29580200

H -4.83592500 -0.47785200 3.17052900

H -1.99291100 0.46089200 6.14128300

H -6.26129800 0.12347800 5.41514500

H -4.40859000 0.73729800 7.32441200

H -0.55927000 0.93170700 -0.54594500

N -1.50522300 1.21391100 -0.82310200

P -2.63337100 0.14353800 -1.33744100

N -3.80872400 1.30586700 -1.49709200

C -3.41163000 2.63931400 -0.97751900

C -1.82122200 2.62980200 -1.12996900

C -5.05492900 1.14740200 -2.24047000

H -5.09942500 0.14337600 -2.66398700

H -5.92997500 1.27824500 -1.60071900

H -5.10470000 1.87903600 -3.05810400

H -3.83451600 3.36759700 -1.67397800

C -3.97221200 2.99464300 0.43799100

H -3.27008300 2.62140800 1.19247900

C -5.33770500 2.33476600 0.73035300

H -6.07699300 2.68543200 -0.00451300

C -5.88133700 2.61211700 2.13780100

C -1.07802700 3.54782100 -0.14187600

C -0.75442000 3.10954300 1.14996900

C -0.73031200 4.85864100 -0.50638800

C -0.12616200 3.96655700 2.05590900

C -0.10389100 5.71372600 0.40066300

C 0.19849400 5.27218400 1.68936800

H -0.97621500 2.09551200 1.46204200

H -0.94820600 5.22037700 -1.50578900

H 0.11173600 3.59766900 3.04951700

H 0.15453500 6.72346900 0.09312200

H 0.69267100 5.93494200 2.39437300

C -1.37036500 2.94755700 -2.58208300

C -0.13723500 2.43271000 -3.01891000

C -2.08905200 3.76195800 -3.46733400

C 0.34391800 2.70149500 -4.29901700

C -1.61076400 4.02819600 -4.75501100

C -0.39550100 3.49571800 -5.17887500

H 0.45842000 1.82135600 -2.34793300

H -3.02758800 4.21733200 -3.16839600

H 1.30298600 2.29225300 -4.60471900

H -2.19398000 4.65915400 -5.42080500

H -0.02315800 3.70347400 -6.17834000

N -2.56829400 -1.15452800 -0.34319200

C -3.07504700 -2.39614400 -0.96743300

C -2.62752200 -2.23248600 -2.48582600

N -2.68807700 -0.77136400 -2.72183900

H -2.53773700 -1.03574600 0.68647100

C -2.68418800 -0.22781500 -4.07444400

H -2.98346000 0.82176900 -4.04954400

H -1.69353500 -0.27965400 -4.53766400

H -3.39551300 -0.78029200 -4.70002900

H -3.40633900 -2.67978700 -3.10933200

C -1.29784800 -2.92046600 -2.92303100

H -1.28021600 -3.88156600 -2.39811900

C -0.02194200 -2.15859200 -2.52084300

H 0.01560900 -1.19399100 -3.04567700

C 1.26850200 -2.93654300 -2.80605000

C -2.47833200 -3.64663700 -0.30369900

C -1.45987300 -3.57661300 0.65155500

C -2.97616100 -4.91016100 -0.66311200

C -0.94802400 -4.74253300 1.22898300

C -2.45455100 -6.07193700 -0.09931800

C -1.43536100 -5.99251600 0.85326200

H -1.08225400 -2.61540600 0.97551200

H -3.78772000 -4.98529300 -1.38191500

H -0.17371000 -4.66170100 1.98747700

H -2.85421600 -7.03866900 -0.39413300

H -1.03794000 -6.89680300 1.30645000

C -4.62097000 -2.45114100 -0.78113100

C -5.55266300 -2.66562800 -1.80304700

C -5.10617300 -2.29602300 0.53118000

C -6.92582700 -2.71098000 -1.53062300

C -6.47082100 -2.34371300 0.80262300

C -7.39132200 -2.54922500 -0.22946000

H -5.23700500 -2.80536900 -2.83174600

H -4.40654900 -2.13671300 1.34753700

H -7.62531100 -2.87714600 -2.34599000

H -6.81530800 -2.22639100 1.82674200

H -8.45639200 -2.58726000 -0.01752300

H 2.12740100 -2.39719900 -2.40209900

H 1.23977800 -3.92674000 -2.33273600

H 1.43436800 -3.08506300 -3.87899500

H -6.80645500 2.04802100 2.30278800

H -5.17118600 2.30314200 2.91196300

H -6.11494100 3.67096700 2.29014100

C 4.03222300 -0.47442100 3.46490800

C 4.90635600 -1.55929200 3.27695000

C 4.38254100 0.49284200 4.42414300

C 6.07714100 -1.67970900 4.02231700

H 4.65781800 -2.31188500 2.53422200

C 5.55775100 0.37743200 5.16507000

H 3.73248000 1.34488900 4.59749900

C 6.41030800 -0.71080800 4.97063900

H 6.73268100 -2.53159200 3.85964400

H 5.80524700 1.13914100 5.90030800

H 7.32378700 -0.80250600 5.55251700

C -1.32366500 -3.26648200 -4.42703500

H -1.19688600 -2.38614800 -5.06673200

H -0.51766000 -3.96334100 -4.67490600

H -2.26645900 -3.75229800 -4.70859100

H -0.05763300 -1.92055200 -1.45247800

H -5.24748200 1.25036100 0.59915800

C -4.08363100 4.52431800 0.57840600

H -4.88552600 4.90812700 -0.06822000

H -4.32455100 4.80645100 1.60695800

H -3.15921800 5.03831400 0.31021000

- **1da**-*RS* (MeH)


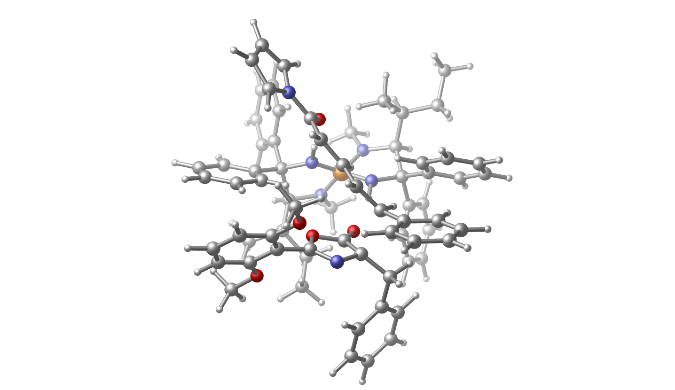

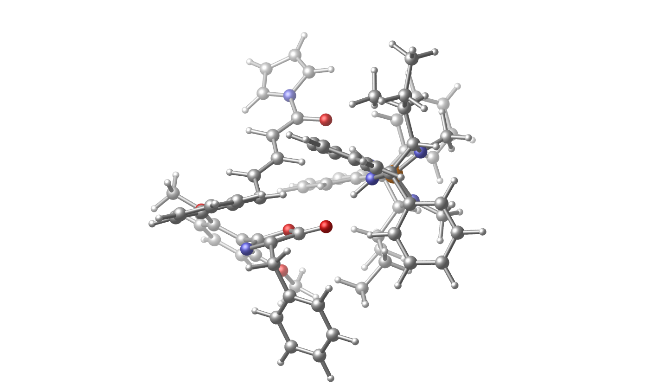


B3LYP/6-31G*

E = –3797.845138

Zero-point correction = 1.342135 (Hartree/Particle)

Thermal correction to Energy = 1.420477

Thermal correction to Enthalpy = 1.421421

Thermal correction to Gibbs Free Energy = 1.221153

Sum of electronic and zero-point Energies = –3796.503003

Sum of electronic and thermal Energies = –3796.424661

Sum of electronic and thermal Enthalpies = –3796.423717

Sum of electronic and thermal Free Energies = –3796.623985

0 1

O -0.84291800 -1.15342500 -0.83791700

C -1.98464900 -0.74402300 -0.62308200

C -3.18378000 -1.39764700 -0.12466300

O -2.29368300 0.59583600 -0.71109600

N -4.21919400 -0.43711100 -0.14153200

C -3.65615400 0.68920000 -0.40315000

C -4.27188200 2.02402100 -0.47366100

C -5.00952200 2.54925100 0.61208500

C -4.15610700 2.80386100 -1.64657700

C -5.58563100 3.82494500 0.53298000

C -4.72430700 4.08177400 -1.72238500

C -5.43065900 4.57507000 -0.62882400

O -5.10595400 1.76361700 1.71823900

C -5.79554300 2.26415700 2.85405100

H -6.85230000 2.45866500 2.63059700

H -5.33044100 3.18125200 3.23780500

H -5.72504400 1.47985700 3.60944500

O -3.49391500 2.21794900 -2.68036100

C -3.34996600 2.94461300 -3.89110000

H -4.32523800 3.20305400 -4.32363800

H -2.76187300 3.85866100 -3.74085600

H -2.81897800 2.27779600 -4.57107400

H -6.14414100 4.23305400 1.36562000

H -4.62970600 4.68046200 -2.61928600

H -5.87602000 5.56474700 -0.68551300

C -3.49963200 -2.82734000 -0.51459100

H -2.72585100 -3.49099400 -0.11211200

H -4.43965100 -3.10431300 -0.02594500

C -3.60864800 -3.05364600 -2.01494800

C -2.76134800 -3.96141000 -2.66262000

C -4.56365100 -2.36895700 -2.78187600

C -2.86088400 -4.18314600 -4.03860300

C -4.66264500 -2.58499500 -4.15608600

C -3.81145400 -3.49284900 -4.79164800

H -2.01982600 -4.50524800 -2.08112600

H -5.22354600 -1.65843900 -2.29174600

H -2.20062500 -4.90140800 -4.51975300

H -5.40996400 -2.04543900 -4.73287400

H -3.89310100 -3.66408000 -5.86204400

C -2.60318600 -1.47114900 1.85054600

C -2.36995100 -0.17280900 2.40775000

H -1.70444000 -2.04405400 1.61978500

H -3.18700800 0.29853900 2.94651100

C -1.15099700 0.48858600 2.35467200

H -0.33928800 0.01337000 1.80443300

C -0.83708600 1.70575300 2.97125200

H -1.60083900 2.20257400 3.55932100

C 0.46169000 2.25633400 2.92860800

O 1.47279600 1.76342100 2.36021500

N 0.67662900 3.48939600 3.63265900

C 1.93468500 3.95044600 3.98017100

C -0.26385100 4.40744000 4.07827600

C 1.79471000 5.14244600 4.64604000

C 0.39642900 5.43660600 4.70180600

H 2.80159200 3.35784600 3.73805000

H -1.31336000 4.27300400 3.87111800

H 2.60062800 5.73885600 5.05336700

H -0.06797200 6.31054500 5.14007600

H 0.84386200 -1.43148700 -0.14871100

N 1.84986500 -1.42391100 0.04846700

P 2.76942200 -0.25129400 -0.65349700

N 4.19834800 -0.94690600 -0.17460700

C 4.03644900 -2.14968400 0.67560700

C 2.59020500 -2.68316200 0.28470600

C 5.51851600 -0.36236900 -0.37446400

H 5.47871700 0.36443900 -1.18801000

H 5.88507300 0.15755800 0.51780000

H 6.23311200 -1.14993600 -0.64386400

H 4.76579300 -2.87968700 0.31455000

C 4.46940200 -1.86969000 2.15006600

H 5.48491000 -1.46536400 2.01143900

C 4.66834500 -3.15686100 2.98345400

H 3.70898900 -3.50482700 3.37662200

C 5.65508700 -2.97902800 4.14533600

C 2.62042800 -3.52055200 -1.03130300

C 3.72239800 -4.27265100 -1.46182400

C 1.45188600 -3.56995500 -1.81245600

C 3.67291500 -5.02223200 -2.64184800

C 1.40149400 -4.31583000 -2.98917900

C 2.51466800 -5.04337200 -3.41488100

H 4.64028400 -4.30100100 -0.88356400

H 0.56471500 -3.02174500 -1.51085400

H 4.54748400 -5.58967700 -2.94953200

H 0.48365100 -4.32257800 -3.57105700

H 2.47665500 -5.62276900 -4.33336400

C 1.90102200 -3.51007100 1.38555400

C 1.03343500 -2.91281600 2.30863600

C 2.13320700 -4.88988000 1.49009900

C 0.43916700 -3.66434400 3.32533800

C 1.54375600 -5.64102000 2.50628100

C 0.69578500 -5.03131100 3.43245300

H 0.82529900 -1.85178500 2.24431700

H 2.77615600 -5.38807700 0.77225700

H -0.22769000 -3.17274700 4.02804900

H 1.74406200 -6.70745100 2.56779700

H 0.23450400 -5.61637000 4.22344300

N 2.13212500 1.20014200 -0.25808400

C 2.40400900 2.25804700 -1.26275800

C 2.31245000 1.42717500 -2.61993300

N 2.93339700 0.12851500 -2.25744600

H 1.82096900 1.42450800 0.70709200

C 3.69252300 -0.63509800 -3.24080800

H 4.42737800 0.01505600 -3.73329600

H 3.04558900 -1.07618300 -4.00338700

H 4.22387100 -1.44619300 -2.73974400

H 2.97470700 1.87471300 -3.36570100

C 0.88771800 1.34064600 -3.26217200

C 0.58268600 -0.00987200 -3.94523600

H 1.25443300 -0.15853100 -4.80339600

C -0.87036700 -0.13826400 -4.42274900

C 1.36761200 3.38750800 -1.12918800

C 0.10048100 3.15209700 -0.58021400

C 1.67437300 4.68612600 -1.56604700

C -0.83573500 4.18306300 -0.47872700

C 0.73599900 5.71335100 -1.47270900

C -0.52454400 5.46602300 -0.92661800

H -0.17150600 2.16448500 -0.22851600

H 2.65643300 4.90354500 -1.97355600

H -1.80723800 3.97203600 -0.04300200

H 0.99870800 6.71070800 -1.81597400

H -1.25307100 6.26799900 -0.84150600

C 3.82310500 2.83644900 -1.01026700

C 4.18263900 3.11635700 0.31990400

C 4.74847700 3.13659800 -2.01775500

C 5.42967300 3.65505800 0.62879400

C 6.00403200 3.67241000 -1.70811800

C 6.35202700 3.93015200 -0.38447000

H 3.47295900 2.91732400 1.11817100

H 4.50864400 2.96941400 -3.06288900

H 5.67865500 3.86606700 1.66546300

H 6.70416500 3.89008600 -2.51067700

H 7.32583700 4.34800900 -0.14370100

H 5.79005500 -3.92180600 4.68745200

H 6.64141900 -2.65669500 3.78733800

H 5.30396200 -2.23421300 4.86760400

H -1.08198000 -1.16316800 -4.74639700

H -1.57197900 0.10300400 -3.61697500

H -1.08384900 0.52205100 -5.27150600

C -3.68467300 -2.29618400 2.46083800

C -3.41989700 -3.61767700 2.85570800

C -4.97563500 -1.78085700 2.67769700

C -4.40250600 -4.39436300 3.47174700

H -2.42948900 -4.03438300 2.68991200

C -5.95716800 -2.56024700 3.28653700

H -5.20966000 -0.78243000 2.32345300

C -5.67531800 -3.86790800 3.69247300

H -4.17222900 -5.41246400 3.77617900

H -6.95218200 -2.14853300 3.43786700

H -6.44363600 -4.47185800 4.16834700

H 0.77983600 -0.81892300 -3.23555100

C 0.72353700 2.50527400 -4.25557300

H 1.38448500 2.36500900 -5.12276100

H -0.30221600 2.56582300 -4.62938500

H 0.96133800 3.47098800 -3.80224900

H 0.14412600 1.45019900 -2.46644700

H 5.04045100 -3.95988500 2.33058700

C 3.67636700 -0.79197600 2.90058500

H 2.69137700 -1.14398800 3.21119600

H 4.21772800 -0.49285100 3.80461700

H 3.52715900 0.11207100 2.30583300

- **1da**-*RS* (MeMe)


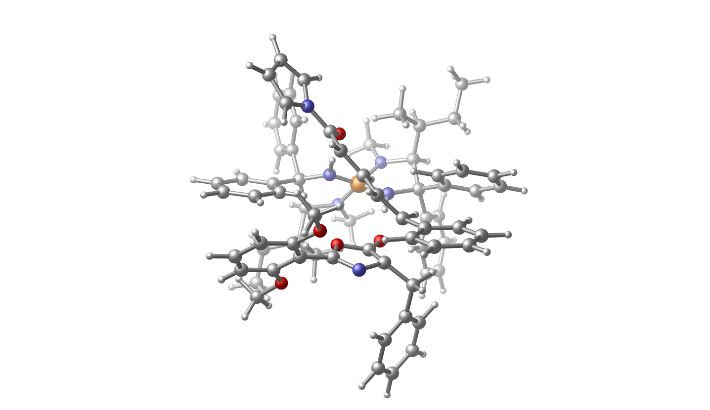

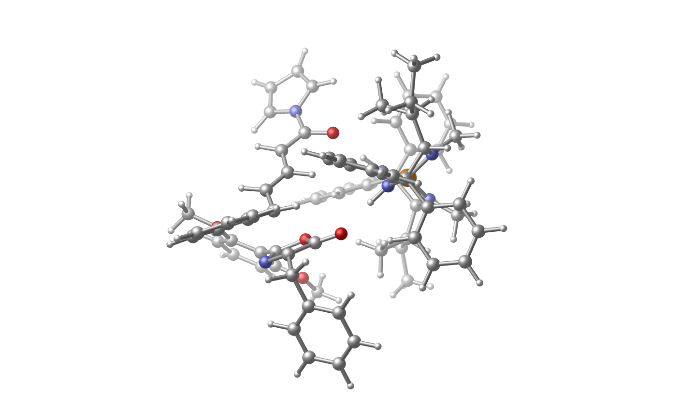


B3LYP/6-31G*

E = –3797.840828

Zero-point correction = 1.341320 (Hartree/Particle)

Thermal correction to Energy = 1.420063

Thermal correction to Enthalpy = 1.421007

Thermal correction to Gibbs Free Energy = 1.217883

Sum of electronic and zero-point Energies = –3796.499508

Sum of electronic and thermal Energie s= –3796.420765

Sum of electronic and thermal Enthalpies = –3796.419821

Sum of electronic and thermal Free Energies = –3796.622945

0 1

O -1.13039200 1.21130200 0.80261100

C -2.15635300 0.54677000 0.64988700

C -3.50780000 0.92427000 0.26909500

O -2.14469400 -0.83065700 0.67675500

N -4.29377900 -0.24606500 0.29780900

C -3.47081200 -1.22228800 0.45314900

C -3.76847500 -2.66342300 0.48406900

C -4.47545300 -3.28762900 -0.56986800

C -3.38459800 -3.45171400 1.59261700

C -4.76320000 -4.65912300 -0.52279000

C -3.66817400 -4.82270700 1.63718600

C -4.35341000 -5.40788100 0.57608400

O -4.83511800 -2.49602700 -1.61557500

C -5.49601900 -3.08721800 -2.72422400

H -6.46422700 -3.51583000 -2.43531800

H -4.88008500 -3.86452300 -3.19444700

H -5.65904500 -2.27610600 -3.43581100

O -2.75917200 -2.78525600 2.60016800

C -2.37208200 -3.51245500 3.75519300

H -3.23763900 -3.97143200 4.25051500

H -1.63502700 -4.28799400 3.51162800

H -1.91902400 -2.78035100 4.42463900

H -5.29694900 -5.14141300 -1.33159800

H -3.37213200 -5.42485100 2.48656700

H -4.57794300 -6.47070200 0.60866600

C -4.10595200 2.23123500 0.74487900

H -3.48357100 3.05875000 0.38592700

H -5.08909700 2.34277800 0.27661700

C -4.24019700 2.33143600 2.25614800

C -3.47401500 3.25076600 2.98357300

C -5.13858700 1.51073300 2.95449300

C -3.60076600 3.35379400 4.37084800

C -5.26498300 1.60931800 4.33979900

C -4.49700400 2.53219100 5.05475500

H -2.77598500 3.89677300 2.45534800

H -5.73121200 0.78657000 2.40204500

H -3.00334800 4.08144700 4.91581300

H -5.96853600 0.96614000 4.86304500

H -4.60005600 2.61163400 6.13409000

C -3.08425000 1.18551200 -1.73541100

C -2.63623800 -0.02290200 -2.36131200

H -2.30415500 1.91600200 -1.52189100

H -3.37028100 -0.61332300 -2.90194600

C -1.31729300 -0.45156300 -2.36072400

H -0.59503700 0.13572600 -1.79542900

C -0.79298500 -1.55851700 -3.03951900

H -1.45510400 -2.16549100 -3.64761000

C 0.58737900 -1.84610500 -3.03535000

O 1.49691700 -1.18575800 -2.46458700

N 1.01827800 -2.98849400 -3.79478300

C 2.31007000 -3.14261900 -4.26605500

C 0.27440600 -4.09078100 -4.18640300

C 2.38389800 -4.32710300 -4.95732800

C 1.09113900 -4.93416900 -4.89915400

H 3.03555500 -2.36501300 -4.08922100

H -0.75322800 -4.20134500 -3.87831900

H 3.26209100 -4.71598200 -5.45607500

H 0.80007000 -5.88720700 -5.32158500

H 0.62613700 1.51606700 0.32689700

N 1.60839800 1.73056800 0.11582500

P 2.80513700 0.71699200 0.60878200

N 4.01426400 1.72590700 0.08133800

C 3.53683000 2.91626600 -0.65824400

C 2.06240200 3.12409400 -0.10194300

C 5.43760200 1.41538000 0.12987800

H 5.62034800 0.65875900 0.89542000

H 5.81412100 1.02659700 -0.82318900

H 6.00209700 2.31944800 0.38919900

H 4.13942100 3.75929100 -0.30949800

C 3.87108200 2.80821500 -2.18067300

H 4.96036400 2.64273800 -2.15599900

C 3.69346000 4.13690700 -2.95121700

H 2.65066800 4.26232700 -3.25526700

C 4.59598700 4.24293900 -4.18796700

C 2.04797200 3.87253200 1.26618900

C 3.05565800 4.73541400 1.71637700

C 0.92041700 3.69695100 2.08934200

C 2.95711900 5.37943200 2.95511300

C 0.82123900 4.33790600 3.32253700

C 1.84320600 5.18064500 3.76646700

H 3.93601900 4.93242700 1.11321900

H 0.10527100 3.05561400 1.76754800

H 3.75893000 6.03874300 3.27760900

H -0.06199600 4.17546200 3.93474600

H 1.76753700 5.67984200 4.72868300

C 1.12926900 3.85784100 -1.07945600

C 0.32265300 3.15864700 -1.98453800

C 1.08541100 5.26003800 -1.09428400

C -0.47981200 3.83968800 -2.90283300

C 0.28568400 5.94188500 -2.01052600

C -0.49799800 5.23452500 -2.92387900

H 0.32609900 2.07595000 -1.98499200

H 1.67735900 5.82828900 -0.38432200

H -1.09056400 3.27076600 -3.59819800

H 0.27267600 7.02858700 -2.00496800

H -1.12121800 5.76450000 -3.63913100

N 2.38085900 -0.79176200 0.14470100

C 2.95908000 -1.86362300 0.98717800

C 3.00773900 -1.18904500 2.42508600

N 3.26974300 0.23946300 2.12839300

H 2.02083200 -0.98268500 -0.80892200

C 3.74501800 1.13167900 3.17810300

H 4.56863400 0.65321200 3.72220000

H 2.95619500 1.39400000 3.89240400

H 4.11298000 2.05723300 2.73111600

H 3.90006700 -1.56147800 2.93470700

C 1.87103800 -1.34914100 3.48103300

C 1.82183700 -2.75703900 4.11891000

H 1.22965600 -3.43170600 3.49339600

C 1.25835900 -2.75032700 5.54633300

C 2.09538000 -3.13407900 0.90330700

C 0.77595100 -3.09724800 0.43702000

C 2.63854800 -4.37328500 1.27670600

C 0.01590400 -4.26680000 0.35832000

C 1.87539700 -5.53764000 1.21329600

C 0.55833700 -5.48939800 0.75205100

H 0.32494900 -2.16160600 0.13022500

H 3.66923700 -4.43370300 1.61204900

H -1.00051300 -4.21127600 -0.01809800

H 2.31798100 -6.48505400 1.51003200

H -0.03413200 -6.39807700 0.68592100

C 4.38369600 -2.20028700 0.44955000

C 4.52155200 -2.36218200 -0.94097700

C 5.52019000 -2.39282400 1.24521900

C 5.75141700 -2.68350700 -1.50995600

C 6.75790400 -2.71108300 0.67382500

C 6.88104800 -2.85433600 -0.70535300

H 3.65530000 -2.24222200 -1.58551200

H 5.47106100 -2.31000800 2.32612000

H 5.82305600 -2.80878700 -2.58707300

H 7.62224400 -2.84943400 1.31840900

H 7.84109300 -3.10399800 -1.14903400

H 4.45653600 5.20817300 -4.68784100

H 5.65671600 4.15796700 -3.91870100

H 4.37597900 3.46044800 -4.92240500

H 1.23344300 -3.76404500 5.96236200

H 1.87171200 -2.13217800 6.21427800

H 0.23714100 -2.35486300 5.57995800

C -4.34101200 1.80017800 -2.24639500

C -4.37159900 3.16617200 -2.57169000

C -5.51243600 1.04389600 -2.43383000

C -5.52414200 3.75586500 -3.09290700

H -3.47763400 3.76752700 -2.42447500

C -6.66444900 1.63594600 -2.94753000

H -5.51997500 0.00150600 -2.13213000

C -6.67557000 2.99221000 -3.28601100

H -5.52168300 4.81350600 -3.34511000

H -7.56332700 1.03785300 -3.07707300

H -7.57597900 3.44956700 -3.68797600

H 2.83464000 -3.18380800 4.14960500

C 0.48673500 -0.83600600 3.06837200

H -0.15518000 -0.73745500 3.95096200

H 0.53085300 0.15307300 2.60511000

H -0.01930300 -1.50425800 2.36969800

H 2.22150000 -0.68564100 4.28767300

H 3.92005400 4.97946500 -2.28237800

C 3.27226900 1.60301500 -2.91640300

H 2.19495800 1.69074700 -3.06023100

H 3.72970600 1.50493500 -3.90688900

H 3.45243800 0.66288900 -2.38891600

- **1da**-*RS* (HMe)


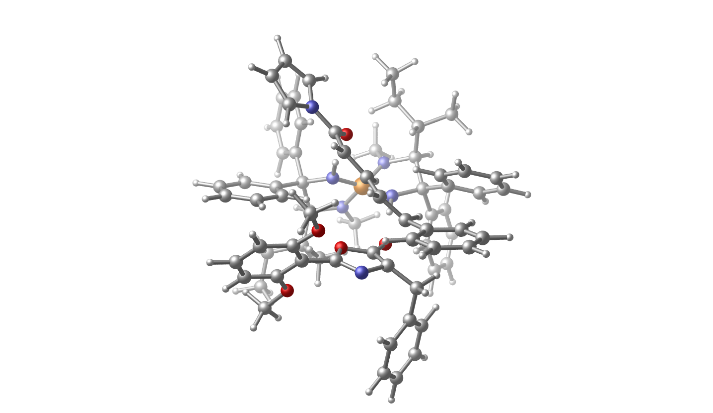

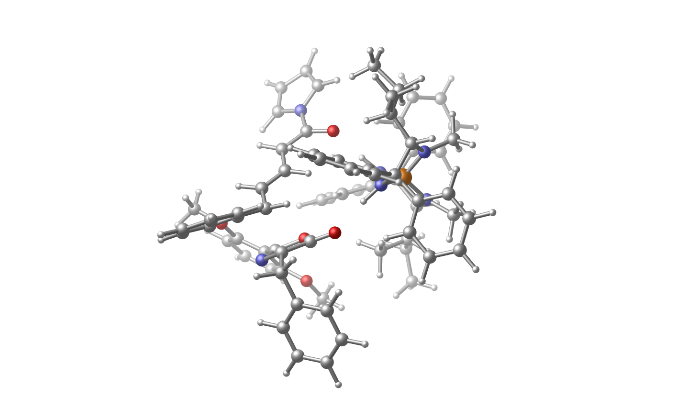


B3LYP/6-31G*

E = –3797.844091

Zero-point correction = 1.342174 (Hartree/Particle)

Thermal correction to Energy = 1.420524

Thermal correction to Enthalpy = 1.421468

Thermal correction to Gibbs Free Energy = 1.220961

Sum of electronic and zero-point Energies = –3796.501918

Sum of electronic and thermal Energies = –3796.423567

Sum of electronic and thermal Enthalpies = –3796.422623

Sum of electronic and thermal Free Energies = –3796.623131

0 1

O -1.28379200 1.27675700 0.74729900

C -2.24123200 0.51505900 0.59716600

C -3.61026400 0.74666200 0.17363500

O -2.10531000 -0.85666300 0.68524000

N -4.28586700 -0.48797700 0.22714600

C -3.38298000 -1.37587000 0.45100500

C -3.55460400 -2.83409500 0.56745800

C -4.10619600 -3.59797300 -0.48511600

C -3.20876900 -3.49398700 1.76744200

C -4.27812600 -4.98307400 -0.34728400

C -3.37744700 -4.87746900 1.90554500

C -3.90869200 -5.60337600 0.84267100

O -4.43899300 -2.92081300 -1.61696600

C -4.95549600 -3.64909400 -2.72046800

H -5.90512000 -4.13983900 -2.47139600

H -4.24078700 -4.40152000 -3.07819800

H -5.12653900 -2.91123200 -3.50603400

O -2.73849700 -2.69248900 2.76107800

C -2.44647600 -3.27452800 4.02115100

H -3.32995100 -3.76252600 4.45281500

H -1.62661400 -4.00052900 3.94928200

H -2.14112100 -2.44712600 4.66313900

H -4.69355800 -5.57314800 -1.15424300

H -3.10931200 -5.38224300 2.82498000

H -4.04354600 -6.67681100 0.94606400

C -4.34284300 2.01018600 0.56991200

H -3.77891100 2.87773200 0.20863300

H -5.30978800 2.01752200 0.05761800

C -4.55218500 2.13941800 2.07016500

C -3.81433200 3.06302200 2.82095700

C -5.48475800 1.33014400 2.73582100

C -4.00329900 3.18175000 4.19963400

C -5.67480500 1.44560900 4.11249800

C -4.93540700 2.37344700 4.85081800

H -3.08636300 3.69524200 2.31748000

H -6.05310600 0.60050600 2.16526700

H -3.42465900 3.90979000 4.76377200

H -6.40479500 0.81182800 4.61043700

H -5.08746800 2.46631900 5.92331200

C -3.14751600 0.96508800 -1.86114000

C -2.57478400 -0.22018000 -2.41753800

H -2.44558200 1.77087900 -1.64948900

H -3.23385700 -0.90107800 -2.94824600

C -1.21953500 -0.51342600 -2.37750600

H -0.57388100 0.16448400 -1.82265500

C -0.57330600 -1.58416100 -3.00613500

H -1.16114300 -2.28571400 -3.58804000

C 0.83019100 -1.71655700 -2.98777200

O 1.65769100 -0.92433800 -2.45530300

N 1.38779700 -2.84794500 -3.67547600

C 2.69610300 -2.89844400 -4.12537800

C 0.76305500 -4.03947200 -4.01242400

C 2.89648200 -4.10514200 -4.74946200

C 1.66936300 -4.83399200 -4.67008700

H 3.34152600 -2.04691700 -3.98336800

H -0.25238900 -4.23614500 -3.70740800

H 3.81638400 -4.43126600 -5.21698500

H 1.48048400 -5.83278400 -5.04172100

H 0.45816400 1.63937400 0.28316500

N 1.43891000 1.89082700 0.10525300

P 2.67604900 0.95307400 0.64392900

N 3.84082600 1.97067300 0.04684200

C 3.29765300 3.02015800 -0.84534900

C 1.84725100 3.27780800 -0.23252900

C 5.26130500 1.94369200 0.37356500

H 5.45905900 1.13052800 1.07362500

H 5.87616000 1.78018000 -0.51528300

H 5.56110400 2.89221300 0.83919000

H 3.92107400 3.90568300 -0.69740600

C 3.33583800 2.66528000 -2.36880300

H 2.40443500 2.14610500 -2.61449800

C 4.47118000 1.69412000 -2.76445000

H 5.44217600 2.20824000 -2.70373100

C 4.29142800 1.10067900 -4.16785300

C 1.89511000 4.12384300 1.06799100

C 2.89793500 5.05668900 1.36231600

C 0.84283500 3.97639200 1.98918800

C 2.86987800 5.79763400 2.54897100

C 0.81146200 4.71710200 3.16926600

C 1.82956200 5.62873700 3.45968900

H 3.71474400 5.23252200 0.66974900

H 0.03652100 3.27828900 1.78230100

H 3.66557200 6.50961100 2.75261200

H -0.01458100 4.58066100 3.86222800

H 1.80695100 6.20405000 4.38122300

C 0.84657600 3.90137600 -1.22051000

C 0.14992300 3.10854100 -2.14235300

C 0.63675500 5.28889000 -1.24782200

C -0.70957600 3.68460300 -3.07947600

C -0.22401700 5.86559400 -2.18222200

C -0.89922200 5.06654200 -3.10564200

H 0.28557600 2.03390300 -2.14115600

H 1.14592000 5.92878800 -0.53524700

H -1.23145900 3.04372900 -3.78446300

H -0.36770900 6.94286700 -2.18255100

H -1.56832700 5.51530400 -3.83488800

N 2.33148400 -0.59530200 0.24109000

C 2.98419900 -1.59071600 1.12007800

C 2.96364400 -0.86377400 2.53388700

N 3.13448900 0.57030300 2.18959800

H 2.05404700 -0.82271600 -0.73118500

C 3.52259300 1.53035000 3.21489300

H 4.40414400 1.16134000 3.75410400

H 2.71989100 1.71496500 3.93764800

H 3.77361700 2.48467200 2.74851800

H 3.87045800 -1.15508000 3.07013400

C 1.82117600 -1.06440800 3.57589000

C 1.85417900 -2.45354500 4.25476500

H 1.32973500 -3.18640600 3.63444100

C 1.25096800 -2.44846100 5.66578800

C 2.21924200 -2.92544300 1.06357200

C 0.90762400 -2.99521600 0.57777800

C 2.84190900 -4.11293500 1.47768000

C 0.23188400 -4.21662800 0.52238000

C 2.16292500 -5.32937900 1.43555300

C 0.85264800 -5.38684100 0.95680800

H 0.39809500 -2.10069700 0.24052900

H 3.86908100 -4.09396500 1.82762500

H -0.78141400 -4.24234300 0.13430100

H 2.66652500 -6.23498200 1.76397700

H 0.32560900 -6.33612100 0.91096100

C 4.44188300 -1.83488300 0.62061700

C 4.64345600 -1.95755600 -0.76556200

C 5.55402400 -1.99774400 1.45776400

C 5.90883100 -2.21374200 -1.28966400

C 6.82644400 -2.24830500 0.93199300

C 7.01137100 -2.35441300 -0.44387300

H 3.80305300 -1.85911100 -1.44600400

H 5.45581000 -1.94805900 2.53744600

H 6.02834000 -2.31174600 -2.36530200

H 7.66905100 -2.36472900 1.60886100

H 7.99845300 -2.55227300 -0.85294300

H 5.10594900 0.40247600 -4.39430600

H 3.34791100 0.54665500 -4.22460700

H 4.29167100 1.86438800 -4.95305700

H 1.29645700 -3.44761400 6.11413700

H 1.79266100 -1.76191400 6.32894500

H 0.20043000 -2.13804500 5.65861700

C -4.44454400 1.43609400 -2.41479900

C -4.60143100 2.78334000 -2.78034100

C -5.53036600 0.56217500 -2.60712200

C -5.79273000 3.24145400 -3.34449200

H -3.77432200 3.47328300 -2.63077000

C -6.72134900 1.02275300 -3.16440200

H -5.44306200 -0.46789600 -2.27673400

C -6.85766100 2.36194500 -3.54189600

H -5.88827100 4.28693700 -3.62733800

H -7.55289300 0.33489300 -3.29773400

H -7.78817600 2.71653700 -3.97763700

H 2.89380500 -2.80482800 4.32513500

C 0.41353000 -0.65770700 3.12488100

H -0.24855100 -0.57342200 3.99385200

H 0.39966500 0.31595800 2.62767600

H -0.03552600 -1.38088600 2.44238500

H 2.11252100 -0.35650500 4.36814100

H 4.50482100 0.86019000 -2.05651600

C 3.42487500 3.95825300 -3.19899600

H 4.39687200 4.44634700 -3.04079600

H 3.33250900 3.74652900 -4.26820300

H 2.64268700 4.67553900 -2.94144900

- **1da**-*RS* (HH)


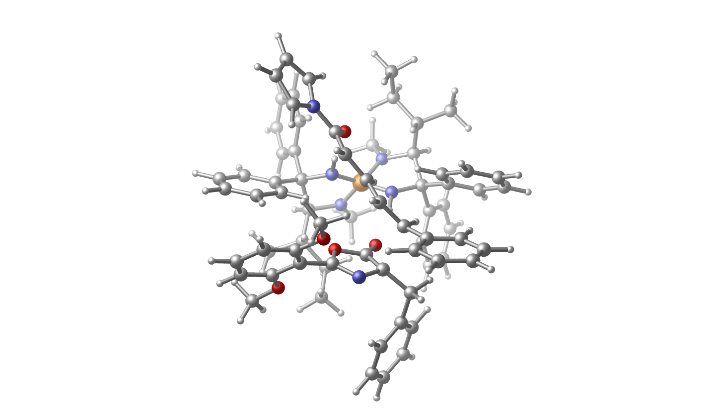

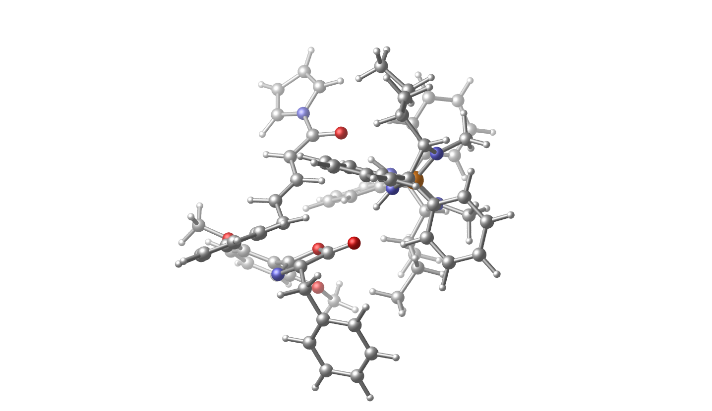


B3LYP/6-31G*

E = –3797.847583

Zero-point correction = 1.341927 (Hartree/Particle)

Thermal correction to Energy = 1.420269

Thermal correction to Enthalpy = 1.421213

Thermal correction to Gibbs Free Energy = 1.220496

Sum of electronic and zero-point Energies = –3796.505655

Sum of electronic and thermal Energies = –3796.427314

Sum of electronic and thermal Enthalpies = –3796.426369

Sum of electronic and thermal Free Energies = –3796.627087

PCM(toluene)-B3LYP-D3/6-311++G**//B3LYP/6-31G*@–30 °C

E = –3799.048862

G = –3797.793823

0 1

O 1.04005500 1.17514100 -0.83324700

C 2.09741700 0.57749000 -0.62379200

C 3.38405700 1.01121100 -0.10838700

O 2.18393200 -0.79414800 -0.74274200

N 4.24686900 -0.10421700 -0.13820800

C 3.50914200 -1.11673500 -0.42978500

C 3.89855700 -2.53292400 -0.52629600

C 4.53222700 -3.19395800 0.55097600

C 3.67009600 -3.25855900 -1.71758400

C 4.89569700 -4.54398900 0.44648400

C 4.02794900 -4.60891600 -1.81965100

C 4.63461400 -5.23363300 -0.73358400

O 4.74653800 -2.45862400 1.67533100

C 5.32807800 -3.09267400 2.80474100

H 6.34024500 -3.45646400 2.58643000

H 4.71082400 -3.92715300 3.16140900

H 5.38001800 -2.32488200 3.57840500

O 3.11895900 -2.55322300 -2.74174500

C 2.89581700 -3.21544000 -3.97722100

H 3.83100600 -3.60717400 -4.39787200

H 2.17238900 -4.03320200 -3.86787900

H 2.48889400 -2.45674400 -4.64621900

H 5.37281100 -5.05539300 1.27264400

H 3.84799200 -5.16501500 -2.73075100

H 4.91581300 -6.28053800 -0.81070000

C 3.93355400 2.37888500 -0.45536100

H 3.23883700 3.14694400 -0.09650700

H 4.87126500 2.51417700 0.09266100

C 4.17120900 2.57811700 -1.94388200

C 3.38702500 3.47282900 -2.68227700

C 5.18131800 1.86831900 -2.61032100

C 3.60381400 3.65919600 -4.04985000

C 5.39849200 2.04998600 -3.97572200

C 4.61108100 2.94723700 -4.70212600

H 2.60231800 4.03279400 -2.17811300

H 5.78872600 1.16325700 -2.04929200

H 2.98940000 4.36567900 -4.60350000

H 6.18736700 1.49192100 -4.47430200

H 4.78426800 3.09199800 -5.76552900

C 2.79380100 1.12129300 1.88618600

C 2.36333400 -0.14272700 2.39840500

H 1.99449900 1.82782300 1.66197800

H 3.09362400 -0.75052000 2.92462600

C 1.05658700 -0.60406400 2.32197900

H 0.33506000 0.00702000 1.78188200

C 0.54940200 -1.77455500 2.89849400

H 1.22261400 -2.41389900 3.45893300

C -0.82315500 -2.09837600 2.85085500

O -1.74481500 -1.40511700 2.33703500

N -1.23008200 -3.32410300 3.47741600

C -2.54229300 -3.60198300 3.82159600

C -0.44480300 -4.41059900 3.83708200

C -2.58752700 -4.84500800 4.40191300

C -1.25436000 -5.36213300 4.40509000

H -3.30780600 -2.86541200 3.64055200

H 0.60950600 -4.43145500 3.61275300

H -3.47396200 -5.33220600 4.78659100

H -0.93158900 -6.32784700 4.77204900

H -0.59275100 1.61162000 -0.09265300

N -1.59466800 1.71896100 0.09760500

P -2.66375500 0.71286100 -0.65034500

N -3.98554800 1.54437300 -0.09213500

C -3.64107100 2.56561400 0.92453000

C -2.18452900 3.02733000 0.46843700

C -5.36785100 1.37519000 -0.52435500

H -5.42367100 0.59725200 -1.28734700

H -6.01395100 1.07630600 0.30488700

H -5.74977200 2.31387100 -0.94769600

H -4.34778300 3.38797900 0.78698500

C -3.75186500 2.08044900 2.40830600

H -2.77939800 1.66932200 2.69659700

C -4.77012000 0.94025500 2.63559200

H -5.79534800 1.32959900 2.54516000

C -4.60120700 0.24636700 3.99359200

C -2.23160400 3.95452900 -0.77589500

C -3.27330700 4.85733600 -1.03151500

C -1.15001900 3.92955800 -1.67350800

C -3.25100800 5.68835700 -2.15624900

C -1.12505300 4.76048800 -2.79312200

C -2.17868600 5.64146800 -3.04475400

H -4.11609000 4.93914400 -0.35239300

H -0.31464100 3.25645700 -1.50357600

H -4.07656400 6.37371700 -2.33002800

H -0.27576900 4.71572300 -3.46976500

H -2.16084600 6.28614300 -3.91924800

C -1.34578500 3.68731200 1.57771500

C -0.60595600 2.91293300 2.48240400

C -1.32240100 5.08271300 1.72823900

C 0.11657900 3.51089600 3.51685800

C -0.60173700 5.68116400 2.76218200

C 0.11972400 4.89802500 3.66411300

H -0.59561700 1.83383100 2.38818200

H -1.86544200 5.71280000 1.03242700

H 0.68086700 2.88329500 4.20071900

H -0.60183400 6.76392800 2.85605000

H 0.68239800 5.36358800 4.46874200

N -2.22634700 -0.83058900 -0.33303500

C -2.67247100 -1.78572000 -1.37650800

C -2.45374600 -0.91687800 -2.69549700

N -2.86895900 0.44546500 -2.27063500

H -2.00717000 -1.12737900 0.63691800

C -3.47318200 1.37128000 -3.22182600

H -4.32642200 0.89490200 -3.72263300

H -2.75752400 1.69749700 -3.97986400

H -3.82767100 2.25896800 -2.69533700

H -3.17555800 -1.22208900 -3.45754600

C -1.03031900 -1.02562300 -3.33510200

C -0.50254400 0.28938800 -3.94795700

H -1.12488100 0.58444500 -4.80561700

C 0.96045200 0.20370500 -4.40412300

C -1.83093700 -3.07209700 -1.29887300

C -0.54920800 -3.06851700 -0.73270700

C -2.33798400 -4.28449400 -1.79321500

C 0.20507600 -4.24207000 -0.66709100

C -1.58066700 -5.45400200 -1.73599900

C -0.30485400 -5.43807900 -1.16992700

H -0.12529100 -2.15228700 -0.33954100

H -3.33691700 -4.32280300 -2.21569200

H 1.19160400 -4.21111300 -0.21532500

H -1.99729300 -6.38047600 -2.12278600

H 0.28128000 -6.35142300 -1.11181100

C -4.16583400 -2.14150800 -1.13996000

C -4.56783800 -2.42777800 0.17690300

C -5.12537400 -2.24411700 -2.15526300

C -5.88360800 -2.78339500 0.46573900

C -6.44885100 -2.59577200 -1.86550300

C -6.83489900 -2.86363900 -0.55459200

H -3.84080300 -2.37550000 0.98267600

H -4.86127000 -2.06447800 -3.19246300

H -6.16381400 -3.00405900 1.49229400

H -7.17238400 -2.66316900 -2.67398300

H -7.86158500 -3.13931300 -0.32931300

H -5.34126600 -0.55461500 4.10783300

H -3.60565300 -0.20519500 4.06475600

H -4.73299200 0.93220100 4.83760600

H 1.34430600 1.19640500 -4.66251300

H 1.59681200 -0.19726800 -3.60814100

H 1.07815100 -0.43176900 -5.28980200

C 3.98461000 1.75678600 2.51477800

C 3.92514500 3.09771400 2.92882600

C 5.17900600 1.04496300 2.72959200

C 5.01201500 3.70410700 3.56050100

H 3.01165400 3.66456800 2.76657800

C 6.26532500 1.65445300 3.35389000

H 5.25908700 0.02733400 2.36187600

C 6.18683300 2.98407200 3.77842000

H 4.93981400 4.74114700 3.87918800

H 7.18368600 1.09150800 3.50279900

H 7.03644300 3.45492400 4.26621600

H -0.57922900 1.08497800 -3.20083000

C -1.04521400 -2.15039000 -4.38592100

H -1.67625300 -1.86924200 -5.24114700

H -0.04003800 -2.34682400 -4.76901700

H -1.42723300 -3.09008000 -3.97877600

H -0.32162100 -1.29407000 -2.54532000

H -4.64653900 0.17874300 1.85964600

C -4.07765500 3.27911200 3.31753400

H -5.08705600 3.65684200 3.10253300

H -4.05072300 2.99237900 4.37294000

H -3.37454600 4.10496800 3.18748300

- **1da**-*RS* (EtH)


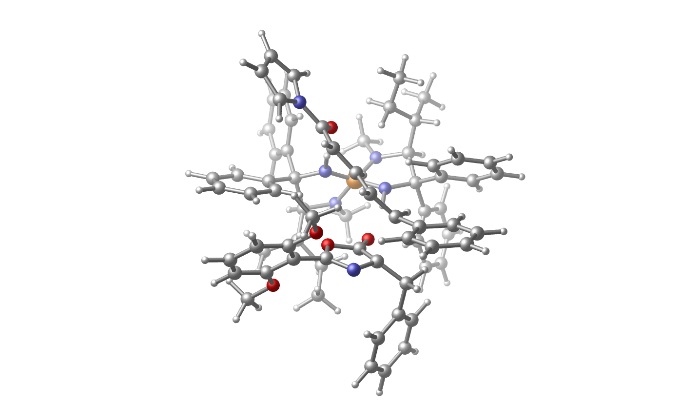

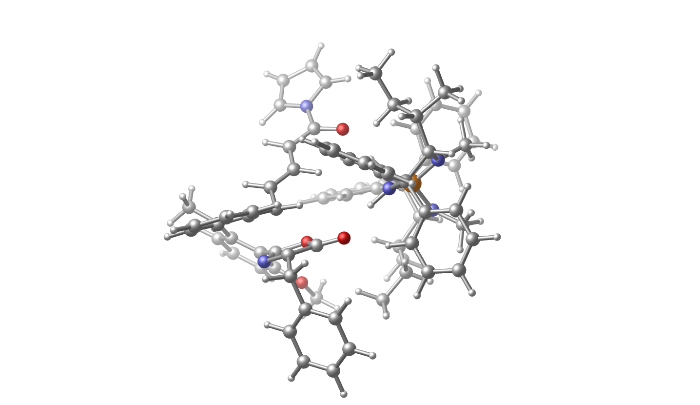


B3LYP/6-31G*

E = –3797.848941

Zero-point correction = 1.342010 (Hartree/Particle)

Thermal correction to Energy = 1.420365

Thermal correction to Enthalpy = 1.421309

Thermal correction to Gibbs Free Energy = 1.220626

Sum of electronic and zero-point Energies = –3796.506931

Sum of electronic and thermal Energies = –3796.428576

Sum of electronic and thermal Enthalpies = –3796.427632

Sum of electronic and thermal Free Energies = –3796.628314

PCM(toluene)-B3LYP-D3/6-311++G**//B3LYP/6-31G*@–30 °C

E = –3799.049946

G = –3797.794783

0 1

O -0.95215200 -1.13366700 -0.82100400

C -2.04677900 -0.60211800 -0.62476900

C -3.30323700 -1.10402100 -0.09404600

O -2.21891200 0.75588100 -0.77937700

N -4.23478900 -0.04544600 -0.15544900

C -3.56210600 1.00341000 -0.47440900

C -4.03743700 2.38922900 -0.61047900

C -4.73202400 3.03274800 0.43967500

C -3.82723200 3.10265600 -1.81287100

C -5.17670300 4.35457900 0.29689300

C -4.26400000 4.42612800 -1.95161000

C -4.93250000 5.03413800 -0.89261400

O -4.92002500 2.31125200 1.57764100

C -5.56033000 2.93279700 2.68206800

H -6.59041400 3.22387800 2.43979900

H -5.00507700 3.81446900 3.02690600

H -5.57460900 2.18188800 3.47369400

O -3.21283000 2.41054700 -2.80956800

C -2.97731300 3.06676800 -4.04575600

H -3.91458600 3.40262900 -4.50809600

H -2.30240300 3.92293000 -3.92143100

H -2.50565500 2.32160700 -4.68716300

H -5.70179800 4.85301000 1.10156200

H -4.09867200 4.97337800 -2.87089000

H -5.27585600 6.05973600 -0.99882600

C -3.76718300 -2.51203000 -0.40433400

H -3.03272200 -3.22634600 -0.01487500

H -4.69979800 -2.68462100 0.14234800

C -3.98084300 -2.77894000 -1.88619100

C -3.19184300 -3.71515200 -2.56587400

C -4.97772500 -2.10019200 -2.60343400

C -3.38829300 -3.96961700 -3.92563100

C -5.17390200 -2.34946600 -3.96135300

C -4.37981600 -3.28523400 -4.62946600

H -2.42044800 -4.25558300 -2.02129000

H -5.59258600 -1.36725700 -2.08826700

H -2.77130300 -4.70831600 -4.43254600

H -5.95222900 -1.81396400 -4.49966000

H -4.53676500 -3.48207300 -5.68698600

C -2.71647000 -1.12583900 1.89129500

C -2.36291000 0.17551200 2.37012300

H -1.87357600 -1.78900100 1.69448500

H -3.13190200 0.75645500 2.87112000

C -1.08328300 0.70638300 2.29044300

H -0.32251500 0.11781700 1.77881600

C -0.64821800 1.92029400 2.83512700

H -1.36092100 2.53252500 3.37641300

C 0.70311100 2.32268100 2.78232300

O 1.66265000 1.68180100 2.27260000

N 1.04289300 3.57171500 3.40253100

C 2.34027100 3.92302500 3.73481800

C 0.20056900 4.61067800 3.77313600

C 2.32080600 5.16532200 4.31784800

C 0.96041900 5.60561600 4.33552800

H 3.14409000 3.23196200 3.54093800

H -0.85579900 4.57202700 3.56141300

H 3.18209200 5.70173300 4.69404600

H 0.58680100 6.55038800 4.70874000

H 0.67653300 -1.57114400 -0.08387900

N 1.67609600 -1.66986400 0.12184800

P 2.73531700 -0.65193100 -0.62197900

N 4.07048200 -1.47534200 -0.08011900

C 3.76294200 -2.58858200 0.84518400

C 2.26146200 -2.98060500 0.47506100

C 5.45078300 -1.08010600 -0.33513300

H 5.49216100 -0.43207300 -1.21264800

H 5.88758200 -0.53081700 0.50538200

H 6.05825300 -1.97208500 -0.52905800

H 4.40380200 -3.41991600 0.54268000

C 4.09061300 -2.31015400 2.34697200

H 3.47230700 -3.00777500 2.92248600

C 3.73898300 -0.88262000 2.80963400

H 4.43840500 -0.16708700 2.35590700

C 3.75001800 -0.69598800 4.33170400

C 2.19703800 -3.93392100 -0.75403500

C 3.18033900 -4.88945400 -1.04902400

C 1.06839500 -3.88013000 -1.59079600

C 3.05790300 -5.74116300 -2.15117200

C 0.94496000 -4.72967100 -2.69000800

C 1.94168000 -5.66281300 -2.98080800

H 4.05762800 -4.99907200 -0.41924500

H 0.26982700 -3.17162000 -1.39338700

H 3.84154300 -6.46658000 -2.35403300

H 0.06205900 -4.65512100 -3.31936800

H 1.84656000 -6.32212300 -3.83925900

C 1.49933700 -3.60515800 1.65821400

C 0.76497000 -2.80738100 2.54644400

C 1.56205000 -4.98512500 1.90267600

C 0.11679900 -3.36981500 3.64807200

C 0.91938700 -5.54718000 3.00628700

C 0.19244400 -4.74282000 3.88455300

H 0.70626000 -1.73750400 2.38870600

H 2.10613400 -5.63300500 1.22367800

H -0.45022500 -2.72669200 4.31516500

H 0.98279600 -6.61940400 3.17228000

H -0.31175700 -5.18094700 4.74160600

N 2.26441600 0.88352700 -0.31795400

C 2.66845600 1.84436700 -1.37482200

C 2.49094800 0.95401800 -2.68580800

N 2.95234800 -0.38574200 -2.24221600

H 1.98937200 1.20080100 0.63068600

C 3.61133200 -1.29762300 -3.16987800

H 4.40853700 -0.77061600 -3.71007400

H 2.91300400 -1.71915200 -3.89736200

H 4.05506600 -2.12368500 -2.61109200

H 3.20656000 1.27839200 -3.44583000

C 1.07052600 0.99748000 -3.33878600

C 0.61760100 -0.34070300 -3.96138500

H 1.26939700 -0.60454000 -4.80739400

C -0.84005100 -0.32525200 -4.44218700

C 1.76904800 3.09146000 -1.31375300

C 0.47614900 3.02806300 -0.77774900

C 2.22925300 4.32520900 -1.80055600

C -0.33541700 4.16338400 -0.73528000

C 1.41573800 5.45738300 -1.76486800

C 0.12878800 5.38146700 -1.22985100

H 0.08837300 2.09461400 -0.38872800

H 3.23447800 4.40960500 -2.20067700

H -1.32970900 4.08459500 -0.30689100

H 1.79674400 6.40193000 -2.14460300

H -0.50167500 6.26578400 -1.18900200

C 4.14392500 2.26548700 -1.13490900

C 4.52444200 2.57989000 0.18192400

C 5.10602700 2.39309900 -2.14456200

C 5.82461200 2.98489600 0.47638600

C 6.41396000 2.79461400 -1.84898100

C 6.78045400 3.08800600 -0.53789300

H 3.79127200 2.51241600 0.98105600

H 4.85631300 2.19289700 -3.18156300

H 6.08940700 3.22675300 1.50236200

H 7.14079000 2.87976100 -2.65280800

H 7.79516000 3.40168400 -0.30797300

H 3.40769900 0.31273900 4.58432100

H 3.07305300 -1.40812100 4.81977700

H 4.74763000 -0.83112600 4.76505700

H -1.16891300 -1.33421600 -4.71370700

H -1.50915900 0.03726800 -3.65449300

H -0.97512100 0.31017500 -5.32528500

C -3.86568300 -1.81448200 2.54344700

C -3.71707800 -3.13029600 3.01142700

C -5.10524700 -1.17591700 2.73027800

C -4.76243100 -3.78229600 3.66737400

H -2.76676300 -3.64013300 2.87325700

C -6.14985300 -1.83142900 3.37886000

H -5.25217600 -0.18088900 2.32322500

C -5.98349300 -3.13463200 3.85639000

H -4.62140000 -4.79835900 4.02786400

H -7.10406100 -1.32556200 3.50567300

H -6.80075100 -3.64148000 4.36303300

H 0.72045400 -1.13325300 -3.21404200

C 1.03956500 2.12461000 -4.38658100

H 1.68783900 1.87483500 -5.23868700

H 0.02870700 2.27559700 -4.77528300

H 1.37537900 3.07996200 -3.97587200

H 0.34127900 1.22525700 -2.55508400

H 2.74838600 -0.59878500 2.44676300

C 5.55321000 -2.68123900 2.66633800

H 6.27206700 -1.99732500 2.20143400

H 5.73079100 -2.65344700 3.74586200

H 5.78954900 -3.69756100 2.32660300

- **1da**-*RS* (HEt)


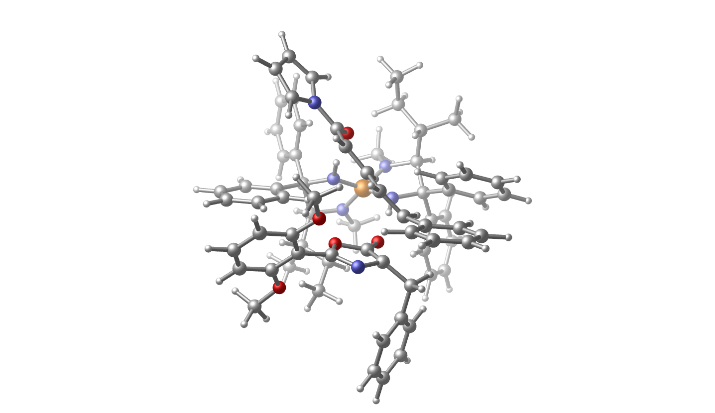

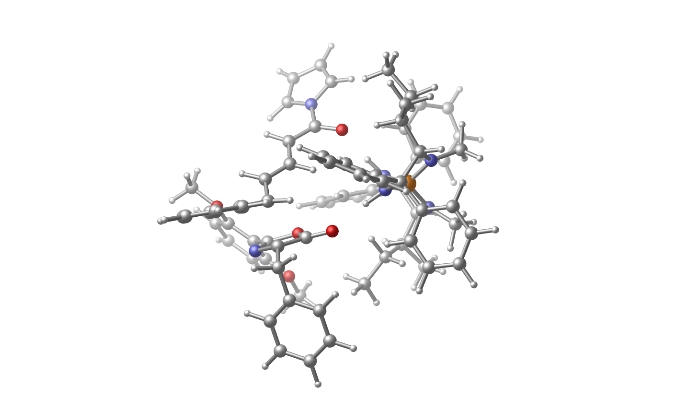


B3LYP/6-31G*

E = –3797.845308

Zero-point correction = 1.341405 (Hartree/Particle)

Thermal correction to Energy = 1.420093

Thermal correction to Enthalpy = 1.421038

Thermal correction to Gibbs Free Energy = 1.217914

Sum of electronic and zero-point Energies = –3796.503904

Sum of electronic and thermal Energies = –3796.425215

Sum of electronic and thermal Enthalpies = –3796.424271

Sum of electronic and thermal Free Energies = –3796.627394

PCM(toluene)-B3LYP-D3/6-311++G**//B3LYP/6-31G*@–30 °C

E = –3799.04514

G = –3797.792242

0 1

O -1.06921300 1.18321700 0.66353800

C -2.14952500 0.60163800 0.54522300

C -3.45460600 1.05045400 0.09695600

O -2.25953600 -0.76830100 0.70481900

N -4.33459600 -0.04661700 0.18244900

C -3.60421900 -1.06649400 0.46425100

C -4.02971200 -2.47072800 0.61434400

C -4.62585600 -3.17235400 -0.45728000

C -3.88936600 -3.13325600 1.85413700

C -5.03931800 -4.50289300 -0.29986700

C -4.29721700 -4.46442900 2.01101500

C -4.86523500 -5.13150500 0.92903700

O -4.75875700 -2.49277400 -1.62860100

C -5.30397800 -3.16963200 -2.75107800

H -6.33669300 -3.49150200 -2.56553000

H -4.69673000 -4.03986000 -3.03157800

H -5.29377900 -2.44403100 -3.56606800

O -3.36678000 -2.38854400 2.86615900

C -3.28636300 -2.97007400 4.15951500

H -4.27393100 -3.28802400 4.51722700

H -2.59919900 -3.82574800 4.17177900

H -2.89876300 -2.18699300 4.81179900

H -5.48887600 -5.04530900 -1.12173400

H -4.18636800 -4.97253200 2.96050800

H -5.18489400 -6.16331500 1.04843100

C -3.96240700 2.43312200 0.44343100

H -3.23682300 3.17618600 0.09316900

H -4.89263600 2.60446400 -0.10687100

C -4.20305600 2.62637500 1.93186700

C -3.32216000 3.38564700 2.71258000

C -5.31129000 2.03623400 2.55738700

C -3.54112600 3.55562500 4.08190800

C -5.53265300 2.20443800 3.92385800

C -4.64864300 2.96600200 4.69245900

H -2.45997500 3.85073000 2.23979000

H -5.99225900 1.43256100 1.96360100

H -2.84919900 4.15435500 4.66987800

H -6.39918000 1.74180400 4.39024300

H -4.82361200 3.10030100 5.75698800

C -2.93977900 1.13497700 -1.94496100

C -2.50559400 -0.12801000 -2.44969100

H -2.14673500 1.85274100 -1.73853700

H -3.23228800 -0.74685800 -2.96768400

C -1.19654900 -0.57994300 -2.35976200

H -0.48658300 0.04343500 -1.81972600

C -0.67236800 -1.75410800 -2.91061500

H -1.32751300 -2.41498800 -3.46778900

C 0.70274000 -2.05720700 -2.83442400

O 1.60834800 -1.33934500 -2.32563800

N 1.13178900 -3.29375000 -3.42727200

C 2.43475300 -3.53611200 -3.82812300

C 0.37239600 -4.42099200 -3.70267800

C 2.49875100 -4.80015300 -4.36129200

C 1.18891500 -5.36578100 -4.27358400

H 3.17721200 -2.76157700 -3.72388100

H -0.66669900 -4.46934900 -3.41828000

H 3.38205400 -5.26897500 -4.77503700

H 0.88660600 -6.35812200 -4.58211000

H 0.73014300 1.56701400 0.16390300

N 1.73166400 1.70197700 -0.00595400

P 2.85354500 0.66928500 0.61301300

N 4.12352800 1.47963800 -0.07800600

C 3.69870400 2.52161200 -1.04167200

C 2.29971600 3.00021500 -0.44224100

C 5.53635100 1.28970300 0.22908100

H 5.64834800 0.47839100 0.95047100

H 6.10630400 1.02559300 -0.66491500

H 5.96166400 2.20697200 0.65837200

H 4.42786700 3.33244500 -0.96394200

C 3.66286900 2.05298700 -2.53421000

H 2.66576200 1.64668900 -2.73017200

C 4.64959400 0.91128200 -2.86755800

H 5.68220200 1.29105500 -2.85296800

C 4.36677400 0.24891600 -4.22215900

C 2.47051400 3.92136600 0.79614600

C 3.57033200 4.76449500 1.00205100

C 1.43861000 3.94171700 1.75036200

C 3.65206200 5.58080500 2.13553100

C 1.51608700 4.75777900 2.87748200

C 2.62819400 5.57853100 3.08010800

H 4.37923400 4.80874900 0.27992700

H 0.56445800 3.31221400 1.61213000

H 4.52067800 6.21989200 2.27151300

H 0.70265300 4.75195300 3.59811000

H 2.69136500 6.21230300 3.96042000

C 1.37018000 3.67443900 -1.46544800

C 0.55319800 2.91493600 -2.31397500

C 1.34609600 5.07158100 -1.59818000

C -0.24326300 3.52940100 -3.28217100

C 0.54933000 5.68665400 -2.56402700

C -0.24724000 4.91815800 -3.41385500

H 0.54364200 1.83477900 -2.23203600

H 1.95128400 5.68911700 -0.94339200

H -0.86186500 2.91328900 -3.92845700

H 0.55051900 6.77030800 -2.64658300

H -0.86703300 5.39627500 -4.16754900

N 2.31089400 -0.85007500 0.34249300

C 2.80817600 -1.83837200 1.32446200

C 2.89221400 -0.97857100 2.66043700

N 3.28360600 0.36901400 2.18565000

H 2.00309200 -1.12702200 -0.60735500

C 3.87446700 1.33292300 3.10468400

H 4.71637400 0.87111800 3.63535600

H 3.15055000 1.69580800 3.84154300

H 4.24379800 2.19613700 2.54827100

H 3.73856000 -1.35241500 3.24035300

C 1.65995300 -0.99955300 3.62562300

C 0.62289400 0.10261400 3.33461100

H 1.03572900 1.08094500 3.61677600

C -0.72375400 -0.09633500 4.03861300

C 1.83599600 -3.02318500 1.43385600

C 0.51925900 -2.93397400 0.96559100

C 2.25419000 -4.21742700 2.04101400

C -0.35390800 -4.01656400 1.09337100

C 1.37718800 -5.29192600 2.18079600

C 0.06913400 -5.19759500 1.70159700

H 0.15667800 -2.02001800 0.51056200

H 3.27587000 -4.31702600 2.39474400

H -1.36582800 -3.92530600 0.71205500

H 1.72372100 -6.20838000 2.65166900

H -0.61216100 -6.03916700 1.79575300

C 4.20011700 -2.36191800 0.86504900

C 4.35393600 -2.69412900 -0.49269000

C 5.29242100 -2.57503200 1.71654200

C 5.55534000 -3.20596800 -0.97767000

C 6.50233200 -3.08192500 1.22886300

C 6.64091400 -3.39840400 -0.11988800

H 3.52613700 -2.55528600 -1.18162300

H 5.22542000 -2.36386900 2.77905000

H 5.63594700 -3.46325400 -2.03035100

H 7.33227300 -3.23156800 1.91482500

H 7.57879300 -3.79613900 -0.49812400

H 5.07866700 -0.56472100 -4.40438500

H 3.35865300 -0.17953200 -4.22899800

H 4.45218400 0.94843700 -5.06073000

H -1.39367800 0.74033700 3.81541400

H -1.21219500 -1.00633800 3.67647300

H -0.62486800 -0.15982500 5.12906600

C -4.16112300 1.74537400 -2.53227900

C -4.14775100 3.09192400 -2.93328100

C -5.34305200 1.00566700 -2.72104600

C -5.26634900 3.67641600 -3.52870600

H -3.24562700 3.68099000 -2.78631400

C -6.46104500 1.59291900 -3.30934200

H -5.38761600 -0.01744000 -2.36257000

C -6.42777400 2.92807400 -3.72236500

H -5.22979200 4.71807500 -3.83822300

H -7.36857200 1.00814300 -3.43905500

H -7.30204600 3.38152100 -4.18227200

H 0.41923400 0.14879900 2.26213900

C 2.13722600 -0.97910400 5.09154900

H 2.64528800 -0.04199100 5.35061100

H 1.29428500 -1.09324600 5.77989000

H 2.83350600 -1.80267300 5.29368200

H 1.16260300 -1.96300000 3.47558500

H 4.58048600 0.13433400 -2.10023000

C 3.90565800 3.25815800 -3.46078400

H 4.93627000 3.62302800 -3.34851500

H 3.76646400 2.98200800 -4.51005700

H 3.22976900 4.09047500 -3.25227600

- **1da**-*RS* (EtEt)


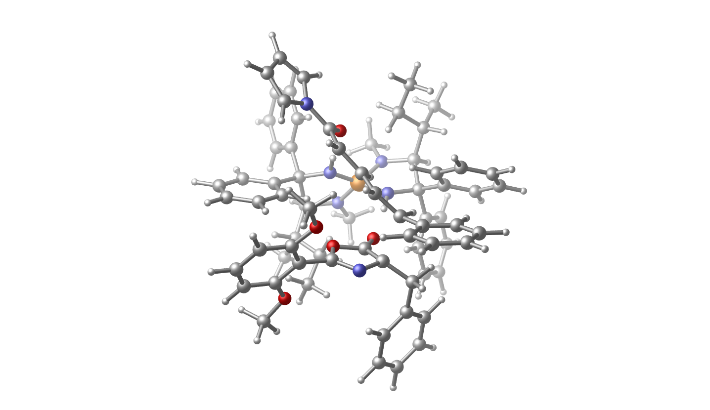

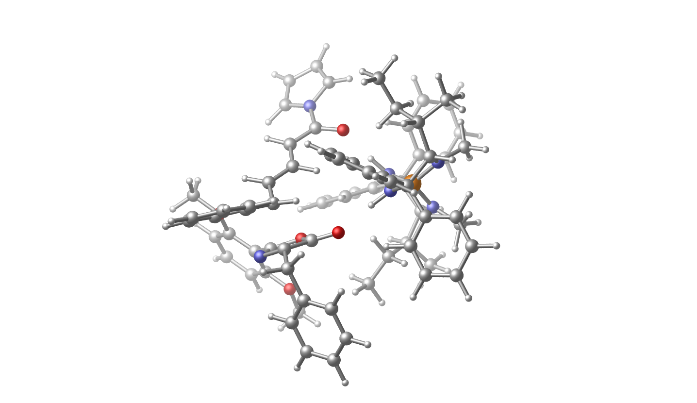


B3LYP/6-31G*

E = –3797.846449

Zero-point correction = 1.341103 (Hartree/Particle)

Thermal correction to Energy = 1.420027

Thermal correction to Enthalpy = 1.420971

Thermal correction to Gibbs Free Energy = 1.216481

Sum of electronic and zero-point Energies = –3796.505346

Sum of electronic and thermal Energies = –3796.426422

Sum of electronic and thermal Enthalpies = –3796.425478

Sum of electronic and thermal Free Energies = –3796.629969

PCM(toluene)-B3LYP-D3/6-311++G**//B3LYP/6-31G*@–30 °C

E = –3799.045815

G = –3797.794099

0 1

O 1.02777100 1.07650800 -0.60420800

C 2.13304400 0.54370000 -0.49896300

C 3.40409200 1.02173200 0.01404900

O 2.31228300 -0.80107900 -0.76121600

N 4.34117200 -0.02293000 -0.14887100

C 3.66629300 -1.05043400 -0.52361300

C 4.16318800 -2.41050200 -0.80609600

C 4.71057600 -3.22114700 0.21102800

C 4.14188300 -2.90728100 -2.12708700

C 5.19226500 -4.50499500 -0.08248300

C 4.61957300 -4.19022900 -2.42253100

C 5.13534600 -4.97241400 -1.39226400

O 4.73170800 -2.68795800 1.46296500

C 5.24673100 -3.47195200 2.52901600

H 6.30286200 -3.72424600 2.36954200

H 4.66924300 -4.39503700 2.66661400

H 5.15467600 -2.85171500 3.42203400

O 3.65983400 -2.04853900 -3.06712100

C 3.74717700 -2.42289600 -4.43394500

H 4.78486800 -2.63041500 -4.72530300

H 3.12567200 -3.30060600 -4.65312400

H 3.37295700 -1.56782100 -4.99798800

H 5.60567300 -5.13438100 0.69528800

H 4.60049000 -4.57282500 -3.43525100

H 5.50794100 -5.96836800 -1.61668100

C 3.84454900 2.45176500 -0.21330500

H 3.15133100 3.12980500 0.29772800

H 4.82117100 2.57822600 0.26649400

C 3.93265700 2.85967100 -1.67691100

C 3.21663000 3.96674700 -2.14996500

C 4.74253300 2.15449200 -2.58032800

C 3.30717400 4.36631000 -3.48574600

C 4.83074500 2.54827500 -3.91561700

C 4.11452900 3.65663400 -4.37502900

H 2.58513400 4.52557400 -1.46252100

H 5.29874200 1.29028700 -2.22808200

H 2.75123300 5.23647800 -3.82787400

H 5.46626200 1.99060100 -4.59949000

H 4.18870400 3.96498500 -5.41485200

C 2.88125000 0.93233700 2.02959900

C 2.45868100 -0.37006800 2.43926800

H 2.07671600 1.65319000 1.88470000

H 3.19342100 -1.02338700 2.90045000

C 1.14885900 -0.81505800 2.33471900

H 0.42928600 -0.15099800 1.85882500

C 0.63296100 -2.02654800 2.80790600

H 1.29680300 -2.72207900 3.30968800

C -0.74382500 -2.32259000 2.73806700

O -1.65473700 -1.57823100 2.28111100

N -1.17009100 -3.58271800 3.27903400

C -2.46814200 -3.83564500 3.68794900

C -0.41291200 -4.72438000 3.49475200

C -2.53191500 -5.12077300 4.16729900

C -1.22655100 -5.68868700 4.03627800

H -3.20758100 -3.05375800 3.62502400

H 0.62180800 -4.76578200 3.19389300

H -3.41225300 -5.60290100 4.57189100

H -0.92546800 -6.69484400 4.29776100

H -0.71801700 1.49509700 -0.12541600

N -1.70986000 1.67245500 0.06084000

P -2.86879300 0.70596400 -0.59345600

N -4.11632000 1.60739600 0.02738200

C -3.69115900 2.71751100 0.90763100

C -2.19307300 3.01539600 0.44480500

C -5.52870900 1.29367800 -0.15113800

H -5.64655000 0.60997500 -0.99459100

H -5.96187400 0.81419900 0.73309900

H -6.08935900 2.21213000 -0.36334600

H -4.29921000 3.58092500 0.62720400

C -3.95104800 2.48164800 2.43034100

H -3.25533700 3.14001700 2.96115000

C -3.67408800 1.03797700 2.89514400

H -4.44810600 0.36814000 2.49565400

C -3.60573400 0.87341300 4.41831700

C -2.12674700 3.95287600 -0.79548000

C -3.09635000 4.91401900 -1.11157700

C -0.99752600 3.86391600 -1.62957600

C -2.96106000 5.73717300 -2.23477900

C -0.85954000 4.68564300 -2.74692300

C -1.84530600 5.62430600 -3.06055100

H -3.97207900 5.05131700 -0.48527200

H -0.20959300 3.14936100 -1.41079800

H -3.73436400 6.46840600 -2.45579000

H 0.02648000 4.58733500 -3.36852600

H -1.74061200 6.26277300 -3.93359400

C -1.33265300 3.59708200 1.57967300

C -0.61895600 2.76157600 2.44923900

C -1.29266300 4.98101800 1.80410500

C 0.10682900 3.29260500 3.51733700

C -0.57063300 5.51222800 2.87343000

C 0.13293700 4.67067800 3.73581700

H -0.64115700 1.68812700 2.30614100

H -1.82062900 5.65380400 1.13633600

H 0.65204800 2.62092700 4.17451500

H -0.55513500 6.58818100 3.02597800

H 0.69636600 5.08373500 4.56811800

N -2.42243500 -0.83816400 -0.30552300

C -2.90527100 -1.81290600 -1.30741800

C -2.96757600 -0.94738700 -2.64801100

N -3.26945900 0.42286600 -2.17669000

H -2.08688800 -1.14398300 0.62587200

C -3.80142200 1.42172900 -3.09447700

H -4.63657400 0.99522300 -3.66351300

H -3.04301600 1.78035800 -3.79833000

H -4.16596000 2.28066300 -2.52748800

H -3.84956800 -1.27091100 -3.20453200

C -1.76790600 -1.04808500 -3.64250900

C -0.60954500 -0.08783300 -3.31420800

H -0.91499300 0.94665700 -3.52382100

C 0.68628800 -0.39320500 -4.07264400

C -1.92405000 -2.99526700 -1.40204900

C -0.59141900 -2.86660200 -0.98797200

C -2.33978200 -4.21845900 -1.94905200

C 0.30068800 -3.93273100 -1.11683900

C -1.44587300 -5.28040200 -2.08426800

C -0.12144400 -5.14291400 -1.66604100

H -0.23297600 -1.93502700 -0.56781400

H -3.37071000 -4.35146300 -2.26132400

H 1.32620100 -3.80349800 -0.78430600

H -1.79200100 -6.22019400 -2.50693900

H 0.57335400 -5.97303000 -1.76276700

C -4.30899300 -2.33034800 -0.87913100

C -4.52015400 -2.60127800 0.48427200

C -5.35991300 -2.59596500 -1.76769500

C -5.74150600 -3.09431900 0.93910000

C -6.58839000 -3.08540300 -1.31109500

C -6.78745200 -3.33259300 0.04475300

H -3.71889400 -2.43472800 1.19787900

H -5.24313100 -2.44251200 -2.83587600

H -5.87025000 -3.30129400 1.99819900

H -7.38518300 -3.27568300 -2.02565800

H -7.74073300 -3.71448700 0.40019700

H -3.31630300 -0.15228700 4.66900400

H -2.85407000 1.54454000 4.85202600

H -4.56369900 1.08150400 4.90848900

H 1.47645500 0.30005300 -3.76733600

H 1.03570900 -1.40535500 -3.84302500

H 0.56518600 -0.30551600 -5.15918300

C 4.08749100 1.51207700 2.68093700

C 4.04243500 2.81649200 3.20047100

C 5.28019600 0.77922700 2.82127100

C 5.14267500 3.36674100 3.85949900

H 3.12950500 3.39827700 3.09771000

C 6.37953100 1.33245400 3.47423400

H 5.34745200 -0.20960900 2.37978700

C 6.31601600 2.62565900 4.00151700

H 5.08183600 4.37570500 4.26011100

H 7.29586200 0.75429800 3.56594300

H 7.17595100 3.05245000 4.51129400

H -0.38362900 -0.12739800 -2.24576800

C -2.25553700 -0.90106100 -5.09833700

H -2.61999400 0.10893500 -5.31965700

H -1.44489600 -1.11452500 -5.80178300

H -3.06765800 -1.60538200 -5.31878300

H -1.38427300 -2.06982300 -3.55251900

H -2.72900800 0.67858200 2.48087200

C -5.36479800 2.95808600 2.82057000

H -6.15333000 2.33455400 2.38479400

H -5.49419200 2.93379900 3.90696600

H -5.53938300 3.99217800 2.49759700

- **1da**-*SR* (MeMe)


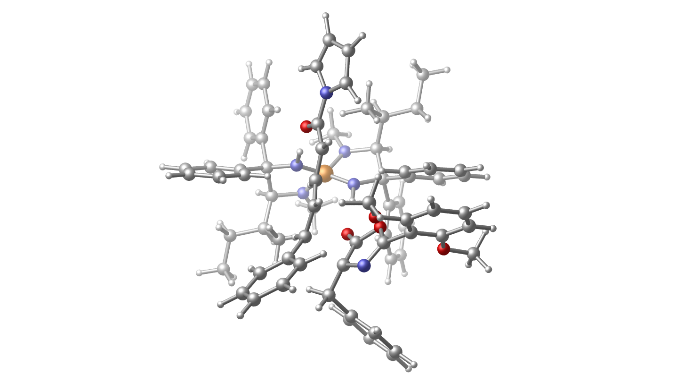

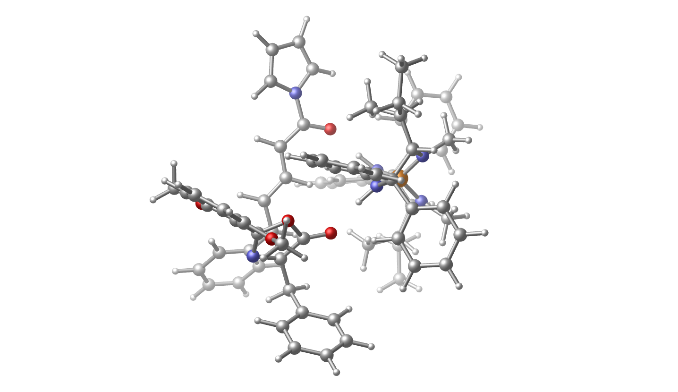


B3LYP/6-31G*

E = –3797.8409

Zero-point correction = 1.341047 (Hartree/Particle)

Thermal correction to Energy = 1.419926

Thermal correction to Enthalpy = 1.420870

Thermal correction to Gibbs Free Energy = 1.216860

Sum of electronic and zero-point Energies = –3796.499853

Sum of electronic and thermal Energies = –3796.420974

Sum of electronic and thermal Enthalpies = –3796.420030

Sum of electronic and thermal Free Energies = –3796.624040

0 1

O -1.09354300 0.31315400 -1.20223900

C -2.23254200 0.42216200 -0.73480000

C -3.17393900 1.52238200 -0.71770000

O -2.78563700 -0.56271400 0.05014700

N -4.34428700 1.06238700 -0.07698100

C -4.05614000 -0.09927000 0.39226400

C -4.91417800 -1.02816200 1.14779900

C -5.27253100 -2.26281300 0.56426100

C -5.41025200 -0.71323900 2.42834800

C -6.08321100 -3.17452400 1.25187100

C -6.22905800 -1.62024200 3.11648300

C -6.54918200 -2.83749800 2.52081500

O -4.79800300 -2.46537500 -0.69259700

C -5.13677800 -3.66427200 -1.37053100

H -6.22363800 -3.76628500 -1.48786700

H -4.73798900 -4.54463300 -0.85197600

H -4.67069700 -3.58669500 -2.35393400

O -5.03469100 0.49201200 2.94192800

C -5.47561300 0.84339800 4.24502200

H -5.11648700 0.13010700 4.99796400

H -5.04789900 1.82705300 4.44561500

H -6.57008800 0.90622400 4.29711900

H -6.35955800 -4.12186500 0.80555200

H -6.60980300 -1.38798800 4.10301800

H -7.18214900 -3.53922300 3.05764300

C -3.29793700 2.46850400 -1.89184700

H -2.32692700 2.94387700 -2.07325700

H -3.99881300 3.26230500 -1.61477800

C -3.76503700 1.78844500 -3.16895900

C -2.87973800 1.56881800 -4.23180600

C -5.09305700 1.35847800 -3.30595900

C -3.30765600 0.94290200 -5.40525900

C -5.52261700 0.73100100 -4.47502600

C -4.63202500 0.52160000 -5.53112900

H -1.84612800 1.89407200 -4.13795500

H -5.78458100 1.51100600 -2.48167600

H -2.60771000 0.79449900 -6.22477700

H -6.55702400 0.40772100 -4.56380100

H -4.96916600 0.03798500 -6.44454300

C -2.17566300 2.64727900 0.77836100

C -1.93440700 1.89449000 1.96301400

H -1.30270700 2.78222100 0.13907100

H -2.67616800 1.92399300 2.75637200

C -0.75065100 1.19862500 2.17440000

H -0.02865500 1.20697600 1.35875600

C -0.36822300 0.52053400 3.33553400

H -1.07753700 0.44396400 4.15218300

C 0.94512900 0.03297800 3.52714300

O 1.93413300 0.19253200 2.76619400

N 1.19879900 -0.69836100 4.73570200

C 2.47087100 -0.94479000 5.22399100

C 0.27936900 -1.28807300 5.59300300

C 2.36027400 -1.67233900 6.38236200

C 0.96645200 -1.89579100 6.61350900

H 3.32688600 -0.55453700 4.69878400

H -0.77653600 -1.26278400 5.37660400

H 3.18282500 -2.00588300 7.00145100

H 0.52277800 -2.44906200 7.43109100

H 0.37830400 -0.72719700 -0.91746200

N 1.25137700 -1.27280900 -0.85904100

P 2.72885200 -0.55563200 -0.93548100

N 3.54277800 -2.00301000 -0.96273200

C 2.68001600 -3.19084000 -0.76584100

C 1.26657000 -2.69625500 -1.28403700

C 4.99149900 -2.15660400 -0.99915700

H 5.25424100 -2.98211700 -1.67221100

H 5.41504900 -2.36012200 -0.00929900

H 5.44937400 -1.24019600 -1.37474100

H 3.04304200 -3.95209400 -1.46174100

C 2.89201100 -3.82476600 0.64613900

H 3.98861600 -3.92199000 0.68746900

C 2.36013100 -5.27271000 0.75688900

H 2.47471300 -5.78108500 -0.21171200

C 3.08306100 -6.10161100 1.82707800

C 0.08204100 -3.47002100 -0.68140300

C -0.26786500 -4.72060600 -1.21531200

C -0.66170100 -2.97215900 0.39333400

C -1.29930400 -5.47283200 -0.65772900

C -1.70578700 -3.71923400 0.94367800

C -2.01944400 -4.97711500 0.43206700

H 0.27100500 -5.11339300 -2.07182900

H -0.43991800 -1.99659400 0.80770400

H -1.54229400 -6.44494600 -1.07930900

H -2.27682300 -3.30271400 1.76771100

H -2.82371700 -5.56205900 0.87100400

C 1.15185200 -2.75499500 -2.83895200

C 0.16758800 -1.95348100 -3.44531300

C 1.92622800 -3.57604900 -3.66922700

C -0.02165400 -1.96294400 -4.82542100

C 1.74120900 -3.58238000 -5.05670100

C 0.76906900 -2.77544600 -5.64196300

H -0.46306300 -1.31306400 -2.83602600

H 2.68493900 -4.23400200 -3.25834700

H -0.79676800 -1.33498600 -5.25703400

H 2.36192700 -4.22727400 -5.67363200

H 0.62375200 -2.78297600 -6.71893000

N 2.77270800 0.66381900 0.16570300

C 3.78234400 1.69945200 -0.16484400

C 3.66454200 1.77890600 -1.74371400

N 3.41152600 0.37037200 -2.13291000

H 2.42589200 0.52301800 1.13163300

C 3.67924000 -0.07491700 -3.49358700

H 2.95153600 0.32133500 -4.21115600

H 3.63054900 -1.16448800 -3.53678000

H 4.68384000 0.24393400 -3.79953700

H 4.64873200 2.03235300 -2.14750000

C 2.67831900 2.75750400 -2.45393700

H 2.72184500 2.41482300 -3.49981200

C 3.18680000 4.21814100 -2.48858300

H 4.28295200 4.22037300 -2.57851300

C 2.60596600 5.03682900 -3.64927200

C 3.51070300 3.02499600 0.56916700

C 4.49008200 4.03137600 0.55730700

C 2.33488400 3.25629800 1.29026700

C 4.28354600 5.24224600 1.21458400

C 2.12859800 4.46762300 1.95583500

C 3.09713900 5.46825600 1.91621100

H 5.42830100 3.86878700 0.03612300

H 1.57531900 2.49054900 1.35762800

H 5.05644500 6.00584900 1.18563200

H 1.20593400 4.61478100 2.51017500

H 2.93780300 6.40893200 2.43634600

C 5.18688200 1.19975100 0.29634700

C 6.36788000 1.34700900 -0.44267400

C 5.27801400 0.60810000 1.56865200

C 7.59489700 0.89713100 0.05740600

C 6.50001000 0.16334200 2.06912100

C 7.66708400 0.29984500 1.31322200

H 6.36179100 1.81810900 -1.42052400

H 4.38478400 0.49730300 2.17678100

H 8.49267600 1.02036000 -0.54294200

H 6.53928300 -0.28846000 3.05681300

H 8.61969400 -0.04865800 1.70279900

H 3.01829100 6.05216700 -3.65151800

H 2.84171900 4.57993900 -4.61894300

H 1.51653400 5.12764400 -3.58143100

H 2.69549100 -7.12649000 1.85082500

H 4.16126400 -6.15825100 1.62886700

H 2.95074700 -5.67868400 2.82854100

C -3.04696100 3.84849200 0.85740800

C -2.60269800 5.06336200 0.30933400

C -4.29849600 3.82456800 1.49976100

C -3.36936100 6.22444000 0.41484200

H -1.63719700 5.09893700 -0.19031600

C -5.06559000 4.98326200 1.59808000

H -4.67904200 2.88315700 1.88170200

C -4.60469500 6.18941400 1.06220300

H -3.00036800 7.15478800 -0.00975400

H -6.03455700 4.94453900 2.08998900

H -5.20671300 7.09076600 1.14356700

H 1.28779600 -5.26372600 0.97086000

C 2.48496800 -2.95917400 1.84331000

H 2.87270900 -3.38915200 2.77266700

H 2.87674600 -1.94154200 1.78349000

H 1.40015200 -2.88909400 1.94395600

C 1.21068700 2.66037600 -2.02266000

H 0.58327700 3.24887500 -2.70170800

H 0.82692400 1.63852700 -2.04633600

H 1.06148100 3.05136500 -1.01290900

H 2.95983900 4.71894300 -1.54290500

- **1da**-*SR* (MeH)


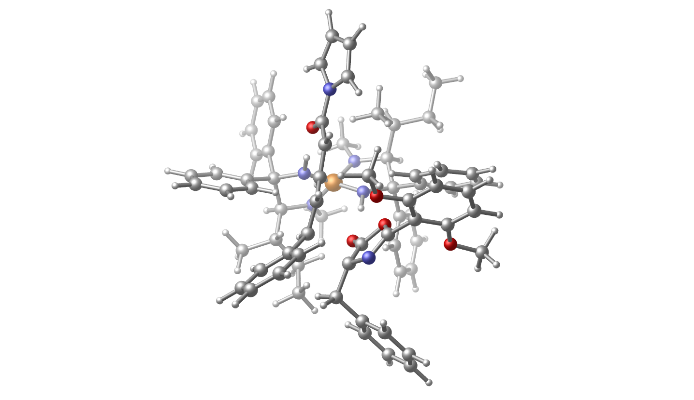

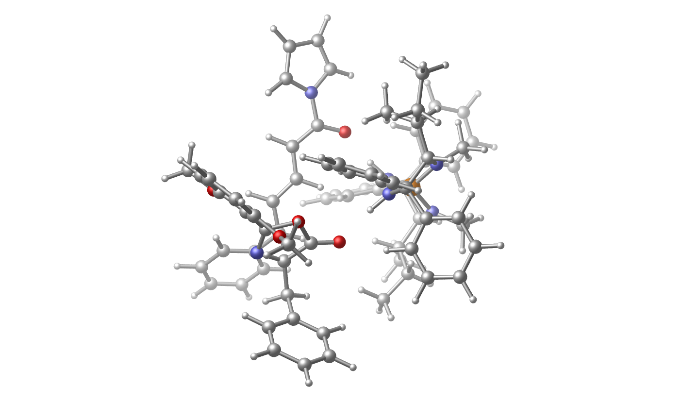


B3LYP/6-31G*

E = –3797.841961

Zero-point correction = 1.342118 (Hartree/Particle)

Thermal correction to Energy = 1.420544

Thermal correction to Enthalpy = 1.421488

Thermal correction to Gibbs Free Energy = 1.219985

Sum of electronic and zero-point Energies = –3796.499844

Sum of electronic and thermal Energies = –3796.421418

Sum of electronic and thermal Enthalpies = –3796.420473

Sum of electronic and thermal Free Energies = –3796.621976

0 1

O -1.08544200 0.30184900 -1.33242800

C -2.18889800 0.34187200 -0.77332500

C -3.23068800 1.35106400 -0.72897500

O -2.56973300 -0.62637600 0.13038000

N -4.27632000 0.84463600 0.06990300

C -3.83488600 -0.24864300 0.57995400

C -4.53992900 -1.19769100 1.46010100

C -4.88487100 -2.47017900 0.95605000

C -4.92248500 -0.86078800 2.77328800

C -5.56150000 -3.40053600 1.75499800

C -5.60458900 -1.78806200 3.57480000

C -5.90907300 -3.04386200 3.05628900

O -4.53538200 -2.69121200 -0.33814000

C -4.91274700 -3.91267600 -0.95303900

H -6.00123800 -4.05325600 -0.93054000

H -4.42001200 -4.76963900 -0.47792300

H -4.57776000 -3.83528500 -1.98846600

O -4.58341700 0.38797400 3.19861500

C -4.91897300 0.76858600 4.52434900

H -4.43800200 0.11705100 5.26533800

H -4.54544400 1.78705100 4.64277300

H -6.00481300 0.75962100 4.68422600

H -5.82558800 -4.37794600 1.37032600

H -5.89472800 -1.54025500 4.58801900

H -6.43640800 -3.76063600 3.68032000

C -3.59110800 2.18436500 -1.93962200

H -2.68360200 2.65091500 -2.33938700

H -4.25005800 2.99458500 -1.61188700

C -4.27803200 1.38541600 -3.03818400

C -3.61640300 1.08298000 -4.23451500

C -5.59540400 0.93230400 -2.87147900

C -4.25030900 0.35109700 -5.24144900

C -6.23039700 0.20074100 -3.87442200

C -5.56087200 -0.09277100 -5.06529600

H -2.59572000 1.42739000 -4.37961300

H -6.11340100 1.14775200 -1.94129900

H -3.71999700 0.13259100 -6.16565700

H -7.25289800 -0.13898400 -3.72733300

H -6.05820200 -0.65936500 -5.84866600

C -2.18969000 2.64386500 0.59491100

C -1.89182900 2.01466200 1.83791500

H -1.34838400 2.73551700 -0.09325800

H -2.59948200 2.12936500 2.65350300

C -0.69545100 1.35358500 2.08454400

H -0.00469000 1.26096600 1.24833400

C -0.26272000 0.82732200 3.30446500

H -0.94254400 0.83833500 4.14925200

C 1.06732600 0.39311700 3.50662400

O 2.03048100 0.49387800 2.70338600

N 1.37342100 -0.20098200 4.77601400

C 2.66379400 -0.36692300 5.24982700

C 0.49226500 -0.72091600 5.71457500

C 2.60233900 -0.97446400 6.47881500

C 1.22121500 -1.20475500 6.77163700

H 3.49536300 -0.01475000 4.66215200

H -0.56889900 -0.74265400 5.52534900

H 3.44916500 -1.22425500 7.10459900

H 0.81369400 -1.68151700 7.65369400

H 0.43913500 -0.75217300 -0.94317400

N 1.33249100 -1.25054600 -0.81407400

P 2.76930000 -0.45396000 -0.92659200

N 3.67015900 -1.84117900 -0.80252000

C 2.87265800 -3.06777300 -0.56748000

C 1.44459300 -2.69257000 -1.15008500

C 5.12599800 -1.90485400 -0.75840200

H 5.47722400 -2.72949000 -1.39144500

H 5.50387600 -2.05652700 0.25858900

H 5.55020000 -0.97213000 -1.13219400

H 3.30235200 -3.83847900 -1.21319100

C 3.08398400 -3.61634400 0.88067500

H 4.18295900 -3.65471700 0.94598800

C 2.62855400 -5.08320000 1.05781900

H 2.79527200 -5.63451100 0.12088400

C 3.36863400 -5.81131300 2.18798800

C 0.29962000 -3.50512300 -0.52360900

C 0.02487200 -4.79756400 -0.99854900

C -0.47050700 -3.00472200 0.53081900

C -0.95432900 -5.58645600 -0.39870100

C -1.46006800 -3.79046800 1.12564900

C -1.69531300 -5.08871600 0.67578000

H 0.58294000 -5.19655000 -1.83940100

H -0.30962600 -1.99792500 0.89451400

H -1.13834900 -6.58998100 -0.77395000

H -2.04972200 -3.37513100 1.93691600

H -2.45595700 -5.70331000 1.15051600

C 1.37898900 -2.85664500 -2.70033400

C 0.39760400 -2.12494700 -3.39394400

C 2.19362000 -3.72010800 -3.44514100

C 0.25458500 -2.23940100 -4.77537200

C 2.05293800 -3.83336400 -4.83289200

C 1.08579600 -3.09174600 -5.50587700

H -0.26917900 -1.45789100 -2.85515500

H 2.94887500 -4.33260700 -2.96350100

H -0.51679100 -1.66216800 -5.27814100

H 2.70447300 -4.50956300 -5.38055800

H 0.97514100 -3.18030000 -6.58324300

N 2.70989100 0.86328600 0.05528800

C 3.62452400 1.95004400 -0.38708800

C 3.43718800 1.86326900 -1.96339000

N 3.40257400 0.40027300 -2.20116300

H 2.38564300 0.79024800 1.03911100

C 3.99740300 -0.16608500 -3.40583100

H 3.46532900 0.15172400 -4.30657300

H 3.95415600 -1.25519800 -3.35673700

H 5.04815000 0.14206500 -3.49055200

H 4.33210100 2.23806600 -2.46731200

C 2.20955200 2.64433400 -2.53727000

H 1.43450900 2.66096700 -1.76194100

C 1.57795900 1.97244100 -3.77678100

H 1.45070700 0.90199500 -3.58946800

C 0.20371400 2.54235700 -4.14636800

C 3.21620500 3.29702300 0.23451500

C 4.15246700 4.33771500 0.34804100

C 1.91347600 3.53121900 0.68784400

C 3.78738500 5.57519800 0.87700100

C 1.54725800 4.76622100 1.22620500

C 2.48155400 5.79628100 1.31873300

H 5.17714000 4.18361000 0.02653300

H 1.17566100 2.74350400 0.63583900

H 4.53105100 6.36427300 0.95217300

H 0.52930300 4.90903400 1.57796000

H 2.20013600 6.75757700 1.74000300

C 5.07384000 1.61396400 0.05713100

C 6.20792200 1.81631400 -0.74028300

C 5.26329100 1.12082100 1.36005500

C 7.48842000 1.51238100 -0.26492500

C 6.53896200 0.82268300 1.83585600

C 7.65983600 1.01088000 1.02336200

H 6.11870300 2.22285600 -1.74262000

H 4.40506900 0.96938300 2.00891300

H 8.34931200 1.67466400 -0.90842700

H 6.65620100 0.44425100 2.84791000

H 8.65399600 0.77608000 1.39381100

H -0.19144200 2.03740300 -5.03555400

H -0.50344700 2.37419900 -3.32694100

H 0.23323500 3.61464400 -4.36719200

H 3.03692200 -6.85335400 2.26041400

H 4.45296200 -5.81848900 2.01710800

H 3.18850100 -5.34212200 3.16107800

C -3.08764000 3.82733100 0.60379300

C -2.70882000 4.99526400 -0.07889500

C -4.30645200 3.83099800 1.30679000

C -3.50696900 6.13947200 -0.04578800

H -1.77072600 5.00795800 -0.62944300

C -5.10550300 4.97171900 1.33262400

H -4.63592800 2.92089500 1.79731500

C -4.70911100 6.13283700 0.66217800

H -3.18887400 7.03428000 -0.57501500

H -6.04823000 4.95391300 1.87408500

H -5.33559100 7.02068600 0.68698700

H 1.55245500 -5.12359500 1.24728600

C 2.61370800 -2.71101900 2.02436700

H 3.03460600 -3.05303600 2.97576900

H 2.92634200 -1.67205800 1.89900100

H 1.52693300 -2.71670300 2.12863100

C 2.61572500 4.09176500 -2.86891900

H 3.34272100 4.10286900 -3.69300100

H 1.74960400 4.67942200 -3.18737800

H 3.06307300 4.60793700 -2.01764600

H 2.25821600 2.07006400 -4.63603100

- **1da**-*SR* (HH)


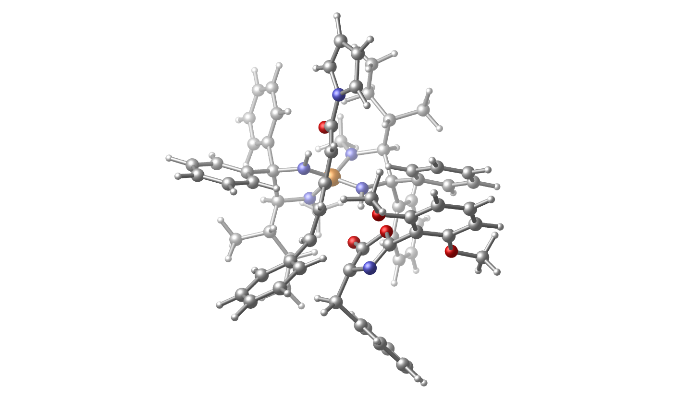

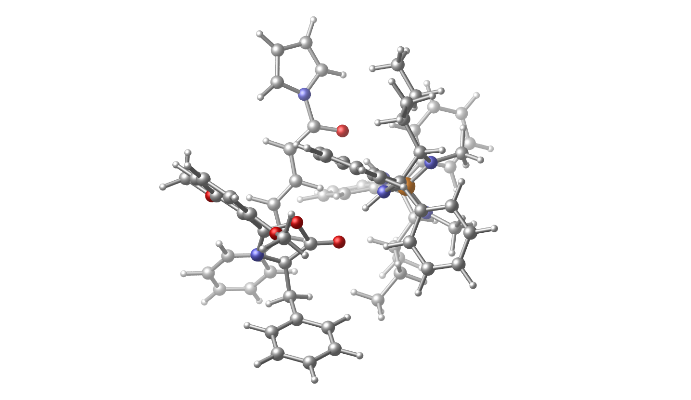


B3LYP/6-31G*

E = –3797.844291

Zero-point correction = 1.342160 (Hartree/Particle)

Thermal correction to Energy = 1.420517

Thermal correction to Enthalpy = 1.421461

Thermal correction to Gibbs Free Energy = 1.220179

Sum of electronic and zero-point Energies = –3796.502130

Sum of electronic and thermal Energies = –3796.423774

Sum of electronic and thermal Enthalpies = –3796.422830

Sum of electronic and thermal Free Energies = –3796.624112

PCM(toluene)-B3LYP-D3/6-311++G**//B3LYP/6-31G*@–30 °C

E = –3799.041975

G = –3797.787147

0 1

O -1.16028000 0.35359200 -1.38721600

C -2.24198300 0.36681600 -0.78398700

C -3.24448800 1.39668600 -0.60511500

O -2.62155600 -0.67177300 0.03400900

N -4.27759900 0.85222300 0.18175900

C -3.85492600 -0.29841200 0.56975900

C -4.54810400 -1.29710100 1.40127800

C -4.91395900 -2.53464700 0.82915000

C -4.89713100 -1.03966100 2.74212300

C -5.57923100 -3.50764300 1.58569300

C -5.56667700 -2.01043800 3.50131500

C -5.89326100 -3.23002100 2.91418800

O -4.60072900 -2.67896600 -0.48482900

C -4.99892200 -3.86127000 -1.15917100

H -6.08810400 -3.99509700 -1.12507500

H -4.50556500 -4.74700800 -0.74088900

H -4.68137700 -3.72941800 -2.19468100

O -4.53797100 0.17776600 3.23696800

C -4.84424200 0.47861300 4.58963800

H -4.35325700 -0.21918300 5.28038900

H -4.46140800 1.48592100 4.76213900

H -5.92672700 0.46621900 4.77096100

H -5.86070400 -4.45743500 1.14793300

H -5.83076000 -1.82370900 4.53455300

H -6.41187700 -3.98025800 3.50537500

C -3.60254400 2.36353100 -1.71080100

H -2.68978600 2.85361600 -2.06710200

H -4.24611900 3.14251000 -1.29159500

C -4.30611500 1.69246400 -2.87982400

C -3.59328000 1.29543900 -4.01865400

C -5.68436300 1.44084500 -2.83261700

C -4.23979300 0.67119000 -5.08707700

C -6.33308600 0.81653900 -3.89799600

C -5.61358700 0.43125800 -5.03157900

H -2.52151900 1.47343300 -4.06102200

H -6.24448500 1.73026700 -1.94739600

H -3.67063000 0.37655700 -5.96594200

H -7.40350700 0.63298500 -3.84429100

H -6.11996900 -0.05026200 -5.86443300

C -2.08007600 2.51216500 0.79265400

C -1.78380400 1.75994000 1.96449800

H -1.26082500 2.60815400 0.07899500

H -2.44698800 1.86289700 2.81848900

C -0.64340600 0.97773400 2.10353500

H 0.00447500 0.88772200 1.23260400

C -0.21941700 0.33066400 3.26723900

H -0.85600800 0.37149400 4.14441600

C 1.05656600 -0.26502200 3.39039400

O 1.99167300 -0.26628500 2.54733600

N 1.33522600 -0.92040500 4.63763000

C 2.61529700 -1.14962100 5.11126700

C 0.42952900 -1.42509700 5.55967100

C 2.52348200 -1.78431600 6.32565100

C 1.13351600 -1.96626700 6.60673000

H 3.46300400 -0.80923800 4.53969300

H -0.63048500 -1.39928200 5.36289400

H 3.35663200 -2.08196700 6.94870900

H 0.70309100 -2.45067500 7.47355700

H 0.30013600 -0.70661700 -1.03320900

N 1.21112300 -1.19100400 -0.99243200

P 2.62618100 -0.36610100 -1.08839000

N 3.55254800 -1.73874100 -1.15927200

C 2.78595600 -2.97908900 -0.88087900

C 1.32362100 -2.61313600 -1.40639300

C 4.95672100 -1.80764500 -1.55079900

H 5.07261500 -2.47029600 -2.41870500

H 5.58742600 -2.17645500 -0.73959800

H 5.31275700 -0.81238400 -1.81848500

H 3.21926700 -3.75088600 -1.52180700

C 2.88140900 -3.49977800 0.58999900

H 2.10974900 -2.99648200 1.18295500

C 4.23162000 -3.17471900 1.26612000

H 4.41481800 -2.09778500 1.19940700

C 4.30112700 -3.58290100 2.74297800

C 0.19275200 -3.42493200 -0.74925700

C -0.28406700 -4.60334000 -1.34405600

C -0.35956700 -3.02637200 0.47577400

C -1.25230300 -5.38270000 -0.71112400

C -1.33494900 -3.80122400 1.10388700

C -1.77680100 -4.98799300 0.52100900

H 0.10532000 -4.92175800 -2.30519800

H -0.03501600 -2.10420500 0.94369300

H -1.59636400 -6.29804000 -1.18622000

H -1.75357100 -3.46443000 2.04739700

H -2.53062800 -5.59327800 1.01779400

C 1.22261800 -2.71622000 -2.95333500

C 0.29463900 -1.89737000 -3.62037000

C 1.96680300 -3.62127500 -3.72334200

C 0.13802400 -1.96711000 -5.00405300

C 1.81440500 -3.68778400 -5.11214200

C 0.90254000 -2.85799200 -5.76039400

H -0.31977900 -1.20138400 -3.05695400

H 2.66955700 -4.30335100 -3.25557300

H -0.59107800 -1.32350800 -5.48922000

H 2.41070200 -4.39669500 -5.68092600

H 0.78237600 -2.90953400 -6.83919600

N 2.64869100 0.80163600 0.06819100

C 3.56879200 1.91539500 -0.26913400

C 3.28037100 2.07157100 -1.82974800

N 3.13589900 0.66443600 -2.28471800

H 2.39594800 0.55389200 1.04354800

C 3.53167300 0.26711200 -3.63170100

H 2.85831000 0.67156500 -4.39110800

H 3.50874200 -0.82108800 -3.71342500

H 4.55183500 0.61312200 -3.84413700

H 4.17138700 2.45863800 -2.33137600

C 2.09421800 3.02447300 -2.18930000

H 1.39173200 3.01873500 -1.34796700

C 1.28839400 2.58435200 -3.42968300

H 0.96099100 1.54821900 -3.29500700

C 0.05015600 3.44966700 -3.69251000

C 3.24064600 3.15509500 0.58448200

C 4.21032800 4.14463000 0.81500700

C 1.97488800 3.32906200 1.15731500

C 3.91345400 5.27634300 1.57399600

C 1.67731900 4.45769100 1.92340200

C 2.64427900 5.43937800 2.13210600

H 5.21038900 4.03088800 0.41008100

H 1.21225000 2.57461600 1.02209200

H 4.68288900 6.02651000 1.73697100

H 0.68596000 4.55473700 2.35707900

H 2.41691300 6.31689900 2.73130300

C 5.03564400 1.49014000 0.01586000

C 6.12402900 1.84531400 -0.79259100

C 5.29768300 0.75652100 1.18550800

C 7.42731200 1.46060400 -0.46007100

C 6.59643300 0.37643200 1.52047300

C 7.66947200 0.72133600 0.69544500

H 5.97982200 2.43709000 -1.69113600

H 4.47761300 0.47822900 1.84136800

H 8.25056600 1.74643900 -1.10973200

H 6.76849500 -0.19006900 2.43176500

H 8.68141300 0.42259600 0.95498000

H -0.54101900 3.03457500 -4.51628400

H -0.59634600 3.48397600 -2.80823400

H 0.30811900 4.47904900 -3.96251100

H 5.24899200 -3.25030900 3.18296900

H 3.49112200 -3.12389000 3.31908900

H 4.24517700 -4.66790800 2.87897300

C -2.90364200 3.73962300 0.93554300

C -2.46394700 4.94571700 0.36527100

C -4.11103800 3.74331100 1.65829200

C -3.19087700 6.12559600 0.52839600

H -1.53328700 4.95942400 -0.19769200

C -4.83933500 4.92071200 1.81333200

H -4.48911400 2.80750200 2.05756600

C -4.38162600 6.11830700 1.25548900

H -2.82629900 7.04914000 0.08555400

H -5.77474200 4.90379200 2.36736800

H -4.95260600 7.03458900 1.38110500

H 5.04608500 -3.67609400 0.72215600

C 2.63613700 -5.02004500 0.61819900

H 2.53675800 -5.38122400 1.64555200

H 1.73021000 -5.31215300 0.08537400

H 3.48404000 -5.54969800 0.16065900

C 2.63214800 4.45566400 -2.37287200

H 3.23594300 4.52162100 -3.28891600

H 1.81577200 5.17808300 -2.46176900

H 3.25527200 4.77504600 -1.53467600

H 1.93152500 2.61139400 -4.32097000

- **1da**-*SR* (HMe)


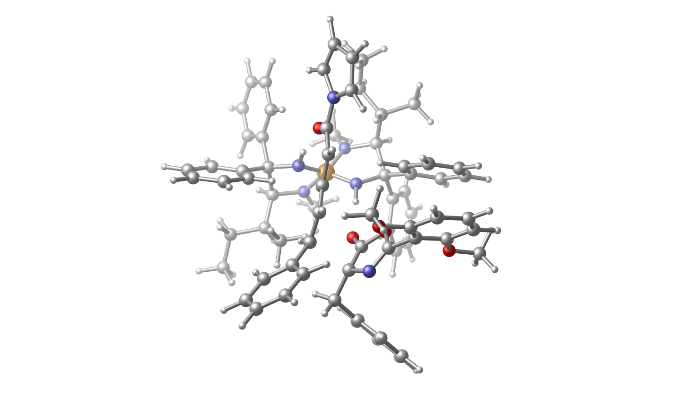

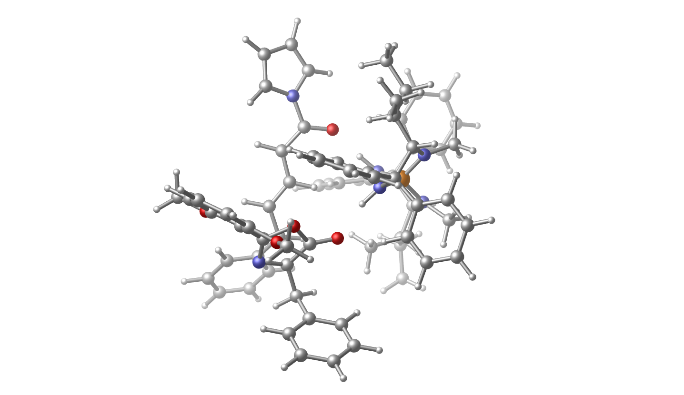


B3LYP/6-31G*

E = –3797.843512

Zero-point correction = 1.341498 (Hartree/Particle)

Thermal correction to Energy = 1.420139

Thermal correction to Enthalpy = 1.421083

Thermal correction to Gibbs Free Energy = 1.218081

Sum of electronic and zero-point Energies = –3796.502015

Sum of electronic and thermal Energies = –3796.423373

Sum of electronic and thermal Enthalpies = –3796.422429

Sum of electronic and thermal Free Energies = –3796.625431

0 1

O 1.17575100 0.35667700 1.31638000

C 2.28439400 0.43003400 0.77171600

C 3.20894300 1.53015200 0.60154500

O 2.79842900 -0.61422600 0.04197300

N 4.33909400 1.02942800 -0.07524100

C 4.03606500 -0.17085300 -0.42526800

C 4.85423500 -1.14844500 -1.16181200

C 5.23722200 -2.34925300 -0.52523800

C 5.29315800 -0.91034900 -2.47991900

C 6.01335100 -3.30287400 -1.19572500

C 6.07543000 -1.86067700 -3.15179100

C 6.41979900 -3.04331700 -2.50253800

O 4.82461900 -2.47784200 0.76277800

C 5.20026100 -3.63480900 1.49113200

H 6.29193300 -3.72678300 1.56493200

H 4.78429800 -4.54447300 1.04109700

H 4.77839200 -3.50344400 2.48873100

O 4.90145300 0.26652100 -3.04489600

C 5.28467500 0.53822900 -4.38435200

H 4.88873400 -0.21454100 -5.07825200

H 4.85320600 1.51180400 -4.62289100

H 6.37601100 0.58977800 -4.48941600

H 6.30905100 -4.22359800 -0.70807700

H 6.41042800 -1.68777400 -4.16668300

H 7.02508500 -3.77832100 -3.02662500

C 3.39086400 2.58204000 1.67302700

H 2.42542600 3.06094100 1.87391100

H 4.05956900 3.35492100 1.28180400

C 3.95038600 2.02128600 2.97076900

C 3.12474700 1.82799700 4.08554200

C 5.30432200 1.67027000 3.07262700

C 3.63640500 1.30480900 5.27513200

C 5.81773900 1.14573000 4.25847600

C 4.98605600 0.96230100 5.36611800

H 2.07139600 2.08976100 4.01713400

H 5.94976600 1.80081600 2.20806700

H 2.98120600 1.17182800 6.13330500

H 6.87087600 0.88196600 4.31948400

H 5.38795000 0.55839100 6.29200000

C 2.08237600 2.49703000 -0.93065500

C 1.84231500 1.64971800 -2.04886200

H 1.23352600 2.62763600 -0.25974700

H 2.53719900 1.68877900 -2.88307300

C 0.71279500 0.84753900 -2.15851900

H 0.03663500 0.82912400 -1.30442900

C 0.33101700 0.09436000 -3.27247800

H 0.98934300 0.07183600 -4.13406000

C -0.92865700 -0.53950400 -3.37061900

O -1.87657800 -0.50064700 -2.54274300

N -1.17242900 -1.29522700 -4.56745600

C -2.44014000 -1.60371200 -5.02939900

C -0.24287400 -1.83686700 -5.44392000

C -2.31727600 -2.32482400 -6.19166500

C -0.91979500 -2.47956100 -6.45058300

H -3.30364500 -1.24965100 -4.49078500

H 0.81440400 -1.75951200 -5.24602800

H -3.13487500 -2.69708100 -6.79488600

H -0.46612800 -3.01246900 -7.27609700

H -0.28601700 -0.67473400 1.03046400

N -1.17827700 -1.19226800 1.05110600

P -2.63626800 -0.44718500 1.08267300

N -3.48183700 -1.86544800 1.23475700

C -2.64672900 -3.07422100 1.02132300

C -1.20791200 -2.60088400 1.52271700

C -4.86455600 -1.99044900 1.68324100

H -4.91113100 -2.61659800 2.58411400

H -5.50447400 -2.43111700 0.91522900

H -5.26124700 -1.00276100 1.92221500

H -3.03590100 -3.83529800 1.70207200

C -2.72241000 -3.67476600 -0.41975300

H -1.99795700 -3.14973600 -1.05233900

C -4.10555500 -3.47594500 -1.07758900

H -4.36368200 -2.41267900 -1.05396200

C -4.17923200 -3.95445300 -2.53281700

C -0.03131400 -3.36751100 0.89305300

C 0.52660800 -4.48153900 1.53904000

C 0.48785200 -2.99038300 -0.35282200

C 1.54734100 -5.21853000 0.93870300

C 1.51586000 -3.72162100 -0.94805400

C 2.04359400 -4.84382300 -0.31135800

H 0.16024800 -4.78194600 2.51513400

H 0.09675900 -2.11807600 -0.86355100

H 1.95511100 -6.08488100 1.45366400

H 1.90615000 -3.39970800 -1.90878500

H 2.84030000 -5.41485900 -0.78102800

C -1.08926200 -2.62654000 3.07154000

C -0.16538100 -1.75793700 3.67985400

C -1.80979400 -3.49851600 3.89930600

C 0.01189500 -1.74956800 5.06224200

C -1.63747600 -3.48594000 5.28788900

C -0.72916100 -2.60928900 5.87654800

H 0.42830200 -1.08327800 3.07029000

H -2.50917200 -4.21447900 3.48028000

H 0.73950900 -1.07059700 5.49928600

H -2.21549100 -4.17068300 5.90328000

H -0.59338000 -2.60085400 6.95475400

N -2.69499900 0.65111300 -0.13971800

C -3.69368800 1.71576400 0.10358400

C -3.53225900 1.96717400 1.66564300

N -3.24778200 0.61236000 2.20254800

H -2.40545400 0.35712200 -1.08955500

C -3.39494700 0.33332500 3.62495800

H -2.61981300 0.82255100 4.22556400

H -3.31669700 -0.74208700 3.79714300

H -4.37828500 0.67274500 3.97472100

H -4.50984500 2.25131900 2.06509400

C -2.54468800 3.02973500 2.23855800

H -2.56758000 2.81398300 3.31819200

C -3.07052400 4.47934200 2.11035400

H -4.16563700 4.48012300 2.21269900

C -2.48595500 5.43196800 3.16172200

C -3.42664400 2.94454200 -0.78587200

C -4.40331500 3.94517500 -0.91248400

C -2.24108400 3.08439400 -1.51582500

C -4.18407700 5.06491100 -1.71251800

C -2.02252800 4.20187200 -2.32535800

C -2.98914200 5.20078400 -2.42255400

H -5.34879100 3.85057300 -0.38826800

H -1.47670400 2.32071900 -1.46718100

H -4.95508200 5.82724200 -1.78812300

H -1.09130000 4.27811900 -2.87959800

H -2.82102000 6.06962700 -3.05307600

C -5.11667000 1.18080900 -0.24977200

C -6.27965900 1.49668000 0.46611000

C -5.25236700 0.38369000 -1.39947300

C -7.53026700 1.01554500 0.06496000

C -6.49864100 -0.09300800 -1.80283700

C -7.64620400 0.21562300 -1.06913500

H -6.23982000 2.13251900 1.34489600

H -4.37579000 0.13114100 -1.98869400

H -8.41223100 1.27407800 0.64533600

H -6.57062200 -0.70681600 -2.69674400

H -8.61727200 -0.15818000 -1.38202500

H -2.90891000 6.43699900 3.05253000

H -2.70613100 5.08720900 4.18019300

H -1.39827900 5.52415000 3.07059900

H -5.15491900 -3.70123000 -2.96487800

H -3.41003900 -3.47444700 -3.14731300

H -4.05835100 -5.03901600 -2.62246600

C 2.88430600 3.73103700 -1.13218100

C 2.39187200 4.96274000 -0.66969400

C 4.11982500 3.71590200 -1.80541300

C 3.09588700 6.14731800 -0.88918600

H 1.43839700 4.99164300 -0.14708400

C 4.82474300 4.89878600 -2.01669900

H 4.53868800 2.76508300 -2.11857000

C 4.31572600 6.12038300 -1.56589500

H 2.69071400 7.08993700 -0.52956500

H 5.78287100 4.86760500 -2.52987800

H 4.86919700 7.04047600 -1.73487700

H -4.87129300 -4.00535200 -0.49062500

C -2.37183600 -5.17411400 -0.38008000

H -2.26537200 -5.57790600 -1.39085600

H -1.43839300 -5.37625300 0.14694700

H -3.17236900 -5.73772000 0.12019600

C -1.08305400 2.89831100 1.79631200

H -0.44990400 3.55545200 2.40336100

H -0.68995200 1.88565000 1.91170800

H -0.95645500 3.19071100 0.75086400

H -2.85898500 4.86944500 1.11061500

- **1da**-*SR* (EtEt)


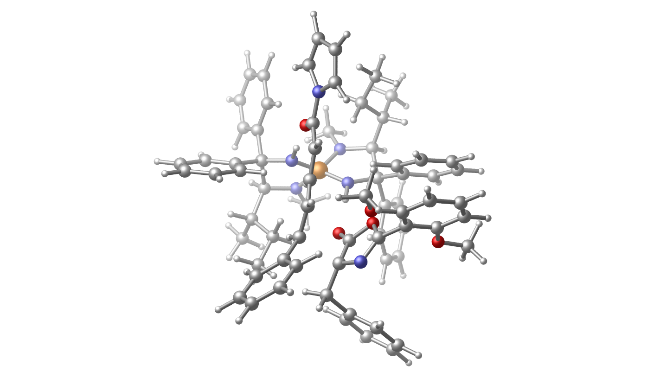

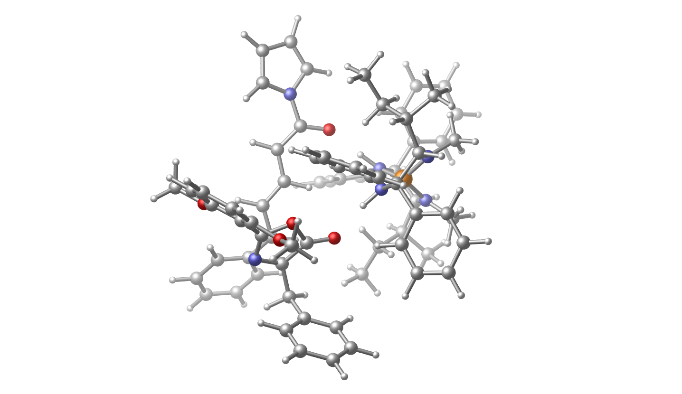


B3LYP/6-31G*

E = –3797.846402

Zero-point correction = 1.341305 (Hartree/Particle)

Thermal correction to Energy = 1.420096

Thermal correction to Enthalpy = 1.421040

Thermal correction to Gibbs Free Energy = 1.217907

Sum of electronic and zero-point Energies = –3796.505098

Sum of electronic and thermal Energies = –3796.426306

Sum of electronic and thermal Enthalpies = –3796.425362

Sum of electronic and thermal Free Energies = –3796.628495

PCM(toluene)-B3LYP-D3/6-311++G**//B3LYP/6-31G*@–30 °C

E = –3799.043497

G = –3797.790604

0 1

O -1.10809100 0.07735600 -1.32757700

C -2.22601700 0.21451900 -0.81604200

C -3.28325200 1.18285300 -1.03202400

O -2.61809900 -0.53554200 0.26818500

N -4.35000700 0.83880400 -0.17569300

C -3.90590700 -0.09980800 0.58180100

C -4.61777300 -0.83577100 1.64088400

C -4.90227300 -2.20520400 1.45127900

C -5.06002000 -0.20688000 2.82138300

C -5.58404100 -2.93957300 2.42966600

C -5.74657900 -0.93838200 3.80160200

C -5.99418600 -2.29275800 3.59337000

O -4.49458300 -2.71919000 0.26168400

C -4.79681300 -4.07169900 -0.04181300

H -5.88000200 -4.25141400 -0.03644600

H -4.30537100 -4.75903700 0.65703300

H -4.40504000 -4.24113300 -1.04591500

O -4.77109300 1.11903900 2.94446800

C -5.16974600 1.79326100 4.12817800

H -4.69891400 1.35725500 5.01879600

H -4.83214300 2.82414200 4.00906900

H -6.26027700 1.78093400 4.25171900

H -5.80430200 -3.99011000 2.28557600

H -6.08279500 -0.46461300 4.71523300

H -6.52606500 -2.85680600 4.35512200

C -3.60639300 1.71320600 -2.41166800

H -2.73105900 2.23026800 -2.81989800

H -4.39795900 2.46177400 -2.30524200

C -4.04481400 0.63118400 -3.38670100

C -3.21542300 0.23910700 -4.44496200

C -5.28841600 -0.00197800 -3.24288300

C -3.61717800 -0.75290900 -5.34325200

C -5.69034600 -0.99538700 -4.13533600

C -4.85732300 -1.37448500 -5.19118100

H -2.24770100 0.71972400 -4.56828200

H -5.93409900 0.28413800 -2.41708300

H -2.96454100 -1.03107500 -6.16799000

H -6.65941700 -1.47224300 -4.00922600

H -5.17439800 -2.14399600 -5.89058300

C -2.30724600 2.76476100 -0.02323400

C -1.99267000 2.43578200 1.32719000

H -1.46618300 2.72890800 -0.71622600

H -2.69320100 2.73111100 2.10317000

C -0.80165100 1.82799500 1.70291900

H -0.12470300 1.53079800 0.90262500

C -0.36432900 1.58889100 3.00916700

H -1.01777700 1.85692600 3.83206800

C 0.93309300 1.11330900 3.30188400

O 1.86282400 0.87289300 2.48869100

N 1.24753700 0.90570400 4.68861100

C 2.54228300 0.83473400 5.17283900

C 0.37262600 0.73665200 5.75225200

C 2.48991100 0.63309200 6.53035700

C 1.10977000 0.56341600 6.89717800

H 3.37138000 0.96419500 4.49673600

H -0.69335500 0.70556800 5.59303300

H 3.34263100 0.55037800 7.19145600

H 0.70743800 0.39399800 7.88748500

H 0.38782900 -0.89347500 -0.80971100

N 1.26014700 -1.41787700 -0.64258400

P 2.73450300 -0.75072300 -0.90974900

N 3.55752400 -2.15614600 -0.59437000

C 2.71314700 -3.27286900 -0.12452200

C 1.25240000 -2.90110600 -0.64774000

C 5.00431100 -2.30982000 -0.68828400

H 5.24013200 -3.28461900 -1.13128900

H 5.49537500 -2.23747200 0.28790400

H 5.41528700 -1.52649200 -1.32785900

H 3.04313900 -4.15380200 -0.67921800

C 2.88064400 -3.63892100 1.38367100

H 1.97961400 -4.19853700 1.65533700

C 2.97279100 -2.42336100 2.32517600

H 2.20502700 -1.68832000 2.06826200

C 2.81156700 -2.78231300 3.80782100

C 0.15973000 -3.44224900 0.28986800

C -0.23055400 -4.78771800 0.19819400

C -0.40756900 -2.64865100 1.29300700

C -1.13692800 -5.33188600 1.10690800

C -1.32860300 -3.18960600 2.19227800

C -1.68789100 -4.53402200 2.11250400

H 0.17802100 -5.41797100 -0.58574900

H -0.13782000 -1.60354500 1.38174600

H -1.41373400 -6.38008000 1.02499800

H -1.76641700 -2.54827000 2.95114400

H -2.39652500 -4.95383400 2.82166200

C 0.98780000 -3.40599900 -2.09714300

C -0.01698400 -2.76253200 -2.84249700

C 1.64368200 -4.49200300 -2.69322700

C -0.33766900 -3.17811900 -4.13313400

C 1.32736000 -4.90643900 -3.99189100

C 0.33759200 -4.25090500 -4.71947800

H -0.56383700 -1.92692000 -2.41694900

H 2.40605700 -5.04995300 -2.15936700

H -1.12698700 -2.66119800 -4.67261200

H 1.85958200 -5.74917700 -4.42580300

H 0.08999700 -4.57474600 -5.72689700

N 2.79399300 0.69465500 -0.13302800

C 3.80405700 1.61406400 -0.69969800

C 3.71932700 1.29586000 -2.25850600

N 3.39661000 -0.14757200 -2.30768000

H 2.43352600 0.79729600 0.83072200

C 3.61448100 -0.93284400 -3.51580100

H 2.84965500 -0.74133000 -4.27595700

H 3.58080800 -1.99635600 -3.26852000

H 4.59877000 -0.70205900 -3.94069400

H 4.72572700 1.39724200 -2.67340600

C 2.80267500 2.20952800 -3.12626300

H 2.94664600 3.22382600 -2.73760900

C 1.30363400 1.88093900 -3.01161800

H 1.02701400 1.79654700 -1.95670500

C 0.39626800 2.92630100 -3.67037900

C 3.48812400 3.08243700 -0.37019800

C 4.42640100 4.07871400 -0.68703700

C 2.28691900 3.47073800 0.23084600

C 4.15936800 5.42185800 -0.43076700

C 2.02255200 4.81684600 0.49800300

C 2.95284300 5.79847700 0.16409200

H 5.37936000 3.80546300 -1.13012500

H 1.55158500 2.73044600 0.51325400

H 4.90076600 6.17379500 -0.68818200

H 1.08288700 5.08545600 0.97247600

H 2.74694800 6.84498900 0.37182500

C 5.20050600 1.27342300 -0.09667000

C 6.40363700 1.32023100 -0.81364100

C 5.26378400 0.95111000 1.27036900

C 7.62536500 1.03127900 -0.19669500

C 6.48105200 0.66650300 1.88686700

C 7.67045700 0.69955300 1.15512600

H 6.41801200 1.58786500 -1.86563900

H 4.35270200 0.92282800 1.86062700

H 8.54062100 1.06982300 -0.78181000

H 6.49814100 0.42190700 2.94567400

H 8.61908100 0.47542100 1.63526700

H -0.65500600 2.67611800 -3.49724800

H 0.57625300 3.92496600 -3.25229100

H 0.54427000 2.98703600 -4.75435200

H 2.83115400 -1.87907700 4.42558400

H 1.85187700 -3.28397100 3.98354100

H 3.60691600 -3.44711400 4.16453000

C -3.24560200 3.88809400 -0.27793000

C -2.90662200 4.88356000 -1.20924000

C -4.46739300 4.00635000 0.40998100

C -3.74690000 5.97451700 -1.43450300

H -1.96650600 4.80659500 -1.75099500

C -5.30838400 5.09246600 0.17860200

H -4.76673700 3.21902400 1.09426900

C -4.95167100 6.08461200 -0.73957700

H -3.45927600 6.73690700 -2.15425700

H -6.25284600 5.16266600 0.71274800

H -5.61053600 6.93099300 -0.91551500

H 3.93574000 -1.91520500 2.17542700

C 4.05828400 -4.61575600 1.58192100

H 5.03216200 -4.13967700 1.42282800

H 4.05727100 -5.01782100 2.59955100

H 3.98704600 -5.47090500 0.89782800

C 3.28598700 2.24568600 -4.59080600

H 3.08819900 1.30919900 -5.12373600

H 2.78417300 3.04523500 -5.14411300

H 4.36386800 2.44268300 -4.64963700

H 1.10144300 0.89562400 -3.45240700

- **1da**-*SR* (HEt)


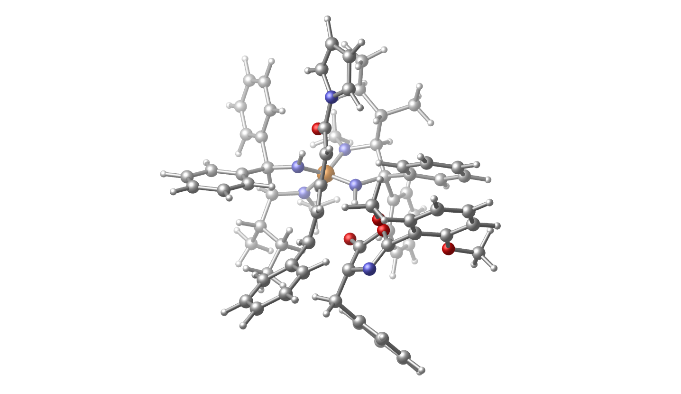

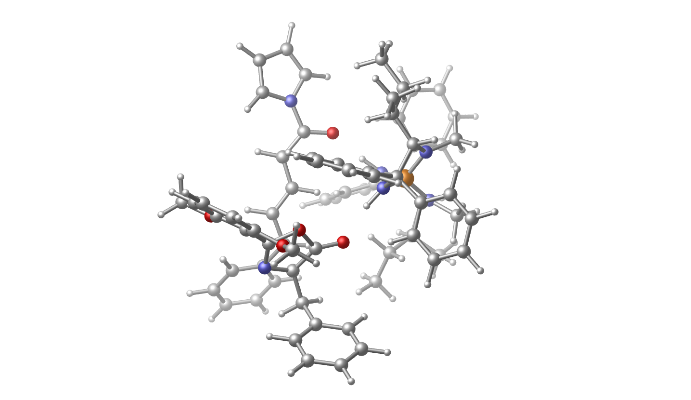


B3LYP/6-31G*

E = –3797.844646

Zero-point correction = 1.341398 (Hartree/Particle)

Thermal correction to Energy = 1.420057

Thermal correction to Enthalpy = 1.421001

Thermal correction to Gibbs Free Energy = 1.218045

Sum of electronic and zero-point Energies = –3796.503248

Sum of electronic and thermal Energies = –3796.424589

Sum of electronic and thermal Enthalpies = –-3796.423645

Sum of electronic and thermal Free Energies = –3796.626601

PCM(toluene)-B3LYP-D3/6-311++G**//B3LYP/6-31G*@–30 °C

E = –3799.042251

G = –3797.789251

0 1

O -1.15336300 0.45976300 -1.30659900

C -2.25802200 0.44132500 -0.74810800

C -3.26627800 1.46232400 -0.55255400

O -2.67527700 -0.64404700 -0.01244600

N -4.33257200 0.87573800 0.15597900

C -3.92889200 -0.29854100 0.49057800

C -4.65727400 -1.33828800 1.23736100

C -5.00679200 -2.53975200 0.58409600

C -5.05250100 -1.15491600 2.57745800

C -5.70493400 -3.54871600 1.25957800

C -5.75529000 -2.16155900 3.25561500

C -6.06683700 -3.34367400 2.58905400

O -4.64367000 -2.61318700 -0.72298300

C -5.01492900 -3.75682300 -1.47445400

H -6.10501700 -3.88653300 -1.49630900

H -4.54416500 -4.66571300 -1.08053900

H -4.65059800 -3.57400100 -2.48655700

O -4.70211600 0.02886800 3.15461100

C -5.05594400 0.25398300 4.51051700

H -4.59482500 -0.48643500 5.17706400

H -4.67381700 1.24661400 4.75465600

H -6.14440300 0.23965200 4.65122000

H -5.97570700 -4.47024800 0.75915800

H -6.05564100 -2.03162100 4.28763600

H -6.61125900 -4.12189400 3.11753700

C -3.57547700 2.49493700 -1.61233000

H -2.66341400 3.05314000 -1.85049100

H -4.28872900 3.21005900 -1.19141800

C -4.14253700 1.88966800 -2.88668000

C -3.33487000 1.69955400 -4.01535700

C -5.48592000 1.49317900 -2.95158900

C -3.85478300 1.13540600 -5.18219600

C -6.00764000 0.92792100 -4.11499500

C -5.19437400 0.74826800 -5.23664000

H -2.28903800 1.99506800 -3.97609500

H -6.11736400 1.62177500 -2.07645700

H -3.21412000 1.00501800 -6.05160900

H -7.05286300 0.62982300 -4.14746800

H -5.60269000 0.31290000 -6.14530600

C -2.16256900 2.51061800 0.95097700

C -1.85987800 1.68823300 2.07208900

H -1.33745500 2.67819900 0.25848800

H -2.53543300 1.71398200 2.92256400

C -0.70446700 0.92212300 2.16724400

H -0.04622600 0.90765200 1.29938400

C -0.28104500 0.19643400 3.28406900

H -0.92117900 0.17185600 4.15912200

C 0.99346700 -0.40822200 3.36498500

O 1.92185000 -0.36445400 2.51506300

N 1.27811500 -1.13870600 4.56871900

C 2.56065100 -1.41009100 5.01239900

C 0.37752800 -1.68765600 5.47051900

C 2.47571600 -2.11466900 6.18816800

C 1.08720500 -2.29783700 6.47485700

H 3.40637000 -1.04506100 4.45332400

H -0.68457000 -1.63874400 5.29030000

H 3.31254700 -2.45768300 6.78227400

H 0.66110200 -2.82787000 7.31674000

H 0.33080000 -0.60093700 -1.05412000

N 1.22973600 -1.10799200 -1.06970200

P 2.67563400 -0.33965100 -1.11450200

N 3.54668800 -1.74494900 -1.23834700

C 2.73663900 -2.96544700 -1.00655900

C 1.28328300 -2.52752600 -1.50623500

C 4.93258300 -1.84999700 -1.68244200

H 4.99308400 -2.47901800 -2.58055300

H 5.57672400 -2.27715400 -0.91070200

H 5.31506400 -0.85725000 -1.92430900

H 3.13581200 -3.72566500 -1.68255200

C 2.83482200 -3.54966200 0.44022600

H 2.10105900 -3.03651100 1.07150300

C 4.21470600 -3.31223900 1.09284100

H 4.44837300 -2.24342100 1.05890300

C 4.30078900 -3.77511200 2.55254500

C 0.13176800 -3.30513400 -0.84465100

C -0.41606200 -4.44039000 -1.46146300

C -0.36585100 -2.92212100 0.40821300

C -1.40302900 -5.19308600 -0.82493900

C -1.35881700 -3.67031300 1.04031900

C -1.87493900 -4.81398000 0.43301400

H -0.06751000 -4.74678700 -2.44207000

H 0.01569400 -2.03264700 0.89611700

H -1.80244300 -6.07612300 -1.31775300

H -1.73246200 -3.34442000 2.00623900

H -2.64627000 -5.39588400 0.93056200

C 1.15184200 -2.59408500 -3.05241300

C 0.22936800 -1.73761500 -3.67942500

C 1.85846600 -3.49683800 -3.85911200

C 0.04087200 -1.77019600 -5.06013000

C 1.67522800 -3.52523400 -5.24580300

C 0.76894700 -2.65963600 -5.85373100

H -0.35494100 -1.04062900 -3.08609200

H 2.55434200 -4.20627400 -3.42309100

H -0.68514200 -1.09975700 -5.51260800

H 2.24284300 -4.23302300 -5.84453800

H 0.62458000 -2.68259600 -6.93058300

N 2.71218300 0.77517700 0.09151400

C 3.69877500 1.84847400 -0.14801800

C 3.61239700 2.05188300 -1.73029200

N 3.27626000 0.70685100 -2.25221000

H 2.41872400 0.48919300 1.04253000

C 3.45203400 0.37896800 -3.66154900

H 2.68644800 0.84651700 -4.28938300

H 3.37766700 -0.70199800 -3.79832600

H 4.44004600 0.70842600 -4.00629300

H 4.62164800 2.27006900 -2.08823200

C 2.71378700 3.21169600 -2.25443500

H 2.84883200 4.03112100 -1.53971200

C 1.21189500 2.87767700 -2.29022900

H 0.91577100 2.41512500 -1.34425600

C 0.32388500 4.10290700 -2.53694800

C 3.33656300 3.11734200 0.64524700

C 4.26151300 4.16866000 0.75298500

C 2.09010900 3.26765600 1.26262200

C 3.93915700 5.33848500 1.43869400

C 1.76950500 4.43525900 1.95967700

C 2.68977100 5.47793900 2.04700800

H 5.24701600 4.07347200 0.30782200

H 1.35904300 2.47229900 1.21260000

H 4.67220300 6.13818300 1.50570800

H 0.79503400 4.51684400 2.43326700

H 2.44194000 6.38639700 2.58921700

C 5.11213500 1.37304800 0.30511400

C 6.30360800 1.72167100 -0.34657000

C 5.20904700 0.60074600 1.47562100

C 7.54540600 1.29729300 0.13672100

C 6.44676000 0.18002900 1.96015900

C 7.62360300 0.52147100 1.29061600

H 6.29241600 2.34097000 -1.23815700

H 4.31058200 0.32195900 2.01791500

H 8.44988800 1.58068900 -0.39533000

H 6.48847600 -0.41632600 2.86773400

H 8.58767800 0.19091300 1.66733500

H -0.73343800 3.82444000 -2.49163800

H 0.49935900 4.87534500 -1.77729600

H 0.49842800 4.55619900 -3.51910300

H 5.27092700 -3.49542600 2.98068200

H 3.52166900 -3.30694100 3.16355600

H 4.20475900 -4.86123100 2.65248400

C -3.02840900 3.69889500 1.15820800

C -2.62100900 4.95205100 0.67118900

C -4.24491900 3.61880800 1.86102500

C -3.38867100 6.09532900 0.89635700

H -1.68470300 5.03105900 0.12327300

C -5.01354300 4.76018800 2.07802500

H -4.59868100 2.64849200 2.19463200

C -4.58818100 6.00451600 1.60315500

H -3.04896900 7.05598100 0.51717700

H -5.95540700 4.67826400 2.61508600

H -5.19072100 6.89237100 1.77655100

H 4.99153300 -3.83060900 0.51071300

C 2.52046400 -5.05724000 0.41540500

H 2.42950500 -5.45533200 1.42990200

H 1.58924200 -5.28667000 -0.10433100

H 3.33172700 -5.60529200 -0.08492600

C 3.22881100 3.74534900 -3.60743900

H 3.04906300 3.04809900 -4.43297900

H 2.73333600 4.68634300 -3.86470900

H 4.30623400 3.94968500 -3.57045500

H 1.01431500 2.12361600 -3.06351600

- **1da**-*SR* (EtH)


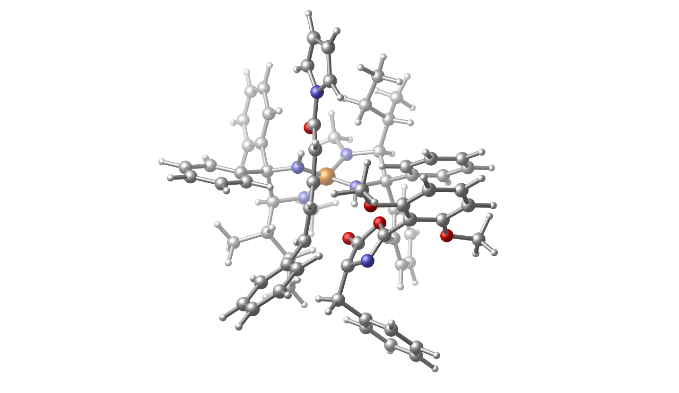

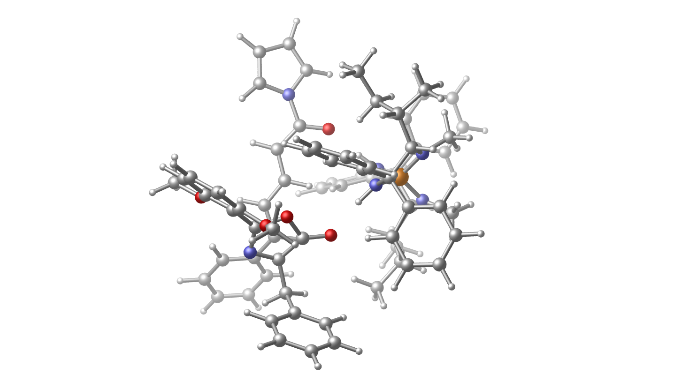


B3LYP/6-31G*

E = –3797.845507

Zero-point correction = 1.341377 (Hartree/Particle)

Thermal correction to Energy = 1.420045

Thermal correction to Enthalpy = 1.420989

Thermal correction to Gibbs Free Energy = 1.217709

Sum of electronic and zero-point Energies = –3796.504130

Sum of electronic and thermal Energies = –3796.425462

Sum of electronic and thermal Enthalpies = –3796.424518

Sum of electronic and thermal Free Energies = –3796.627798

PCM(toluene)-B3LYP-D3/6-311++G**//B3LYP/6-31G*@–30 °C

E = –3799.041914

G = –3797.78919

0 1

O -1.12412900 -0.15791400 -1.36336900

C -2.21700600 0.06452300 -0.82772400

C -3.27482500 1.01054400 -1.12782600

O -2.56459800 -0.52108100 0.36798100

N -4.30089000 0.80937100 -0.18209100

C -3.83179000 -0.02324800 0.67723200

C -4.50308700 -0.59649900 1.85599400

C -4.79277700 -1.97792300 1.87897500

C -4.91305500 0.20167300 2.94238100

C -5.44169600 -2.55767100 2.97636600

C -5.56720400 -0.37511400 4.04073600

C -5.81672600 -1.74495600 4.04389200

O -4.42570200 -2.66412500 0.76504600

C -4.78081300 -4.03327900 0.65481800

H -5.86728200 -4.17188400 0.72997500

H -4.27769400 -4.64153500 1.41594300

H -4.44126600 -4.34533500 -0.33398900

O -4.62870200 1.53023300 2.85461800

C -5.00746600 2.37848100 3.92748000

H -4.51389500 2.09064000 4.86486900

H -4.68172200 3.37996800 3.64140400

H -6.09497200 2.37954700 4.07633700

H -5.66422600 -3.61741600 2.99599300

H -5.87779100 0.22938100 4.88347400

H -6.32279400 -2.18901100 4.89711100

C -3.66338000 1.33859500 -2.55325500

H -2.78672000 1.71249000 -3.09329800

H -4.39383800 2.15337600 -2.52539000

C -4.24725700 0.15008700 -3.30347000

C -3.53486800 -0.47066900 -4.33724500

C -5.51369500 -0.35301000 -2.96992600

C -4.07115800 -1.56215800 -5.02513100

C -6.05032200 -1.44393300 -3.65300200

C -5.33193900 -2.05351900 -4.68488100

H -2.55240000 -0.09187900 -4.60745800

H -6.06894300 0.11179200 -2.16005500

H -3.50617000 -2.02167000 -5.83328300

H -7.03475200 -1.81759800 -3.38145600

H -5.75342600 -2.90046400 -5.22055700

C -2.22796200 2.69022700 -0.39708400

C -1.91830300 2.57728000 0.99044900

H -1.39455100 2.50838500 -1.07724200

H -2.59052900 3.04688400 1.70310900

C -0.76080100 1.97680100 1.46989700

H -0.11395500 1.49419000 0.73829400

C -0.31993700 1.95873900 2.79562700

H -0.94847600 2.40609500 3.55766600

C 0.95788100 1.48156900 3.16419400

O 1.87018900 1.06752400 2.40340200

N 1.27167100 1.49282900 4.56625700

C 2.56431600 1.43009200 5.05740300

C 0.39730400 1.55214500 5.64217200

C 2.51175500 1.45970400 6.42930800

C 1.13288500 1.53065800 6.80086200

H 3.39331900 1.39540000 4.36985300

H -0.66998700 1.55451600 5.48908300

H 3.36383500 1.43862900 7.09605100

H 0.72974700 1.55130700 7.80504400

H 0.39509500 -1.04188000 -0.72006800

N 1.27839200 -1.51184600 -0.46830600

P 2.73163000 -0.83399600 -0.82224500

N 3.60093900 -2.17174900 -0.36701000

C 2.78757800 -3.25604900 0.22297500

C 1.32301800 -2.98922600 -0.34137800

C 5.05426300 -2.28176600 -0.41091800

H 5.33535400 -3.27473400 -0.78196900

H 5.51040100 -2.13066000 0.57237700

H 5.46306000 -1.52762100 -1.08495100

H 3.14291000 -4.18604300 -0.22642000

C 2.95496600 -3.44854900 1.76379100

H 2.06262200 -3.99049100 2.09267300

C 3.01794500 -2.13495400 2.56527800

H 2.22551100 -1.45509100 2.24025200

C 2.88333300 -2.33040600 4.08078300

C 0.24557500 -3.49243500 0.63042300

C -0.06726200 -4.86088100 0.66164700

C -0.37996600 -2.63962200 1.54579600

C -0.95439200 -5.36799600 1.60930000

C -1.28183100 -3.14578500 2.48457500

C -1.56161900 -4.51078600 2.53045400

H 0.39001900 -5.53594300 -0.05590900

H -0.16896700 -1.57729900 1.53434700

H -1.17200800 -6.43299000 1.62506700

H -1.76548400 -2.46340900 3.17685700

H -2.25080900 -4.90324100 3.27384400

C 1.08476700 -3.62242200 -1.74408400

C -0.03811300 -3.17952300 -2.46738200

C 1.88602300 -4.61201000 -2.32806100

C -0.33936100 -3.69861100 -3.72387400

C 1.58628400 -5.13295400 -3.59269400

C 0.47444200 -4.67964200 -4.29666200

H -0.68566800 -2.41446000 -2.05046300

H 2.75704000 -5.00650000 -1.81502100

H -1.21798300 -3.33404000 -4.24959200

H 2.22950500 -5.89893000 -4.01854300

H 0.24094800 -5.08646300 -5.27695800

N 2.74736300 0.68927200 -0.20760900

C 3.68418400 1.59031800 -0.92554500

C 3.44477800 1.11964200 -2.42609000

N 3.34071700 -0.35377600 -2.28971300

H 2.39101600 0.89846400 0.74303300

C 3.84134700 -1.22990100 -3.34185100

H 3.28778400 -1.09382800 -4.27463700

H 3.73015100 -2.27113100 -3.03404900

H 4.90437000 -1.03019500 -3.53284900

H 4.33931500 1.31135600 -3.02498200

C 2.23386600 1.79732000 -3.14554300

H 1.50040800 2.06409500 -2.37553500

C 1.50211000 0.87281500 -4.14486500

H 1.31876700 -0.10042500 -3.67851700

C 0.14903900 1.42526300 -4.60669800

C 3.34745500 3.06603300 -0.64699200

C 4.32427200 4.06137800 -0.81491700

C 2.07170400 3.46040600 -0.22787000

C 4.02494400 5.40455500 -0.58988900

C 1.77207300 4.80362300 0.00687200

C 2.74606100 5.78310300 -0.17705800

H 5.33068600 3.79038400 -1.11626000

H 1.30087500 2.72028800 -0.06659500

H 4.79948300 6.15448100 -0.72789600

H 0.77316500 5.07010700 0.34059400

H 2.51639600 6.82888900 0.00832500

C 5.13276500 1.31223200 -0.44010700

C 6.25255100 1.28570400 -1.28142800

C 5.33882300 1.12931600 0.93806500

C 7.53443600 1.05695000 -0.76917500

C 6.61580500 0.90788200 1.45046900

C 7.72164900 0.86354300 0.59762400

H 6.14994700 1.45161600 -2.34924300

H 4.48909700 1.16104200 1.61400700

H 8.38440600 1.03541100 -1.44637700

H 6.74650600 0.77278300 2.52091400

H 8.71704000 0.68784200 0.99638000

H -0.31485100 0.74260100 -5.32788800

H -0.53025800 1.51557900 -3.75235000

H 0.23225900 2.40375500 -5.09150400

H 2.86858500 -1.36164900 4.58983500

H 1.94739800 -2.84771700 4.32578000

H 3.70916500 -2.91571500 4.50170900

C -3.11906500 3.80004000 -0.82474400

C -2.74097800 4.62911500 -1.89344100

C -4.33511200 4.06793100 -0.16971700

C -3.53735400 5.70577300 -2.28537500

H -1.80430100 4.43491500 -2.41108500

C -5.13250700 5.13916100 -0.56663400

H -4.66441500 3.40440100 0.62368600

C -4.73666900 5.96649500 -1.62184300

H -3.21994600 6.33925900 -3.10996000

H -6.07347500 5.32608500 -0.05492000

H -5.36151900 6.80177100 -1.92725200

H 3.96287400 -1.61635200 2.34953800

C 4.14580800 -4.37946300 2.07437000

H 5.11508800 -3.91107300 1.86999300

H 4.14217200 -4.66707800 3.13002700

H 4.09136800 -5.30597200 1.48863500

C 2.70284400 3.08189200 -3.85261400

H 3.38170700 2.83398100 -4.68052600

H 1.85665600 3.63205300 -4.27451800

H 3.22715700 3.76187000 -3.17833300

H 2.14228200 0.69635400 -5.02191900

- **1da**-*SS* (MeMe)


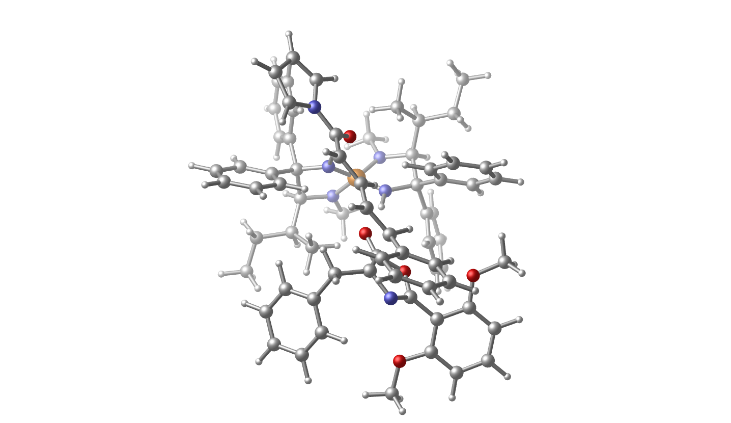

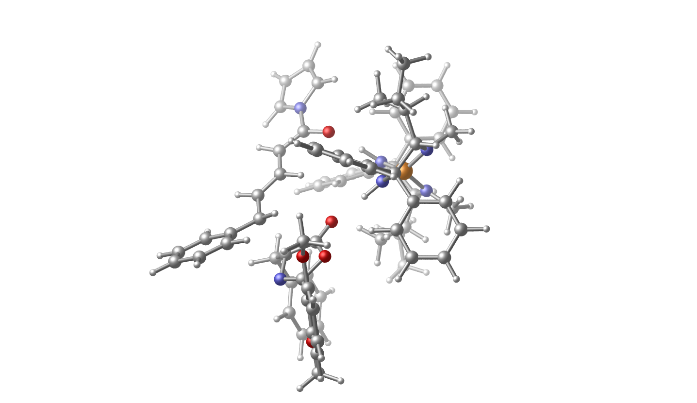


B3LYP/6-31G*

E = –3797.837525

Zero-point correction = 1.341470 (Hartree/Particle)

Thermal correction to Energy = 1.420033

Thermal correction to Enthalpy = 1.420978

Thermal correction to Gibbs Free Energy = 1.219512

Sum of electronic and zero-point Energies = –3796.496055

Sum of electronic and thermal Energies = –3796.417492

Sum of electronic and thermal Enthalpies = –3796.416548

Sum of electronic and thermal Free Energies = –3796.618013

0 1

O 0.87752400 -0.52699700 -0.05926000

C 2.09383300 -0.66883200 0.00190900

C 2.96003700 -1.67248700 0.62495300

O 2.94688200 0.32037100 -0.48116100

N 4.29194600 -1.32301800 0.27642800

C 4.23485500 -0.18221900 -0.30506400

C 5.34209600 0.63735300 -0.83016800

C 5.59895700 1.91401600 -0.29313300

C 6.16183700 0.15862700 -1.87231800

C 6.65156300 2.69921200 -0.77977100

C 7.22105600 0.93831800 -2.35833400

C 7.44907600 2.19571500 -1.80515800

O 4.77474600 2.29636400 0.72263500

C 4.95078100 3.58669700 1.29115300

H 5.93648900 3.68495700 1.76409800

H 4.16819800 3.68517900 2.04350000

H 4.82785100 4.37424200 0.53709000

O 5.83842300 -1.06510800 -2.36659700

C 6.59237400 -1.58518800 -3.45007100

H 7.64162000 -1.74653200 -3.17084800

H 6.54762000 -0.92587500 -4.32692000

H 6.12932500 -2.54240800 -3.69403400

H 6.85737500 3.67769300 -0.36424200

H 7.85693400 0.57637900 -3.15644100

H 8.27058100 2.79917900 -2.18223500

C 2.59713300 -3.14233000 0.63256200

H 1.59018700 -3.23527300 1.05140600

H 3.28436300 -3.64086100 1.32806500

C 2.66136800 -3.85007500 -0.71202200

C 1.51770700 -4.44714500 -1.25666100

C 3.87332600 -3.97069400 -1.41079800

C 1.57490200 -5.14649200 -2.46535800

C 3.93226600 -4.66894700 -2.61671600

C 2.78428400 -5.26016400 -3.15094800

H 0.57113000 -4.37087600 -0.72655500

H 4.76505900 -3.50339100 -1.00451900

H 0.67498300 -5.60902700 -2.86356700

H 4.88290000 -4.76497600 -3.13721800

H 2.83466200 -5.80934500 -4.08791000

C 2.72174700 -1.09949200 2.56756400

C 1.52643300 -1.67195700 3.12756300

H 2.64651900 -0.03837600 2.33267200

H 1.62568900 -2.56795700 3.73700900

C 0.25794800 -1.16308700 2.92606600

H 0.16520600 -0.29207800 2.28549400

C -0.94296200 -1.65295600 3.46952700

H -0.90867000 -2.47443400 4.17761700

C -2.17729200 -1.05820700 3.15905200

O -2.36979300 -0.08365500 2.37720000

N -3.35429900 -1.59323400 3.79523700

C -3.53019800 -2.82937700 4.39517500

C -4.53568300 -0.88102000 3.90756700

C -4.81317600 -2.89850800 4.88201400

C -5.45096200 -1.65667800 4.57864300

H -2.74540200 -3.56881800 4.38222900

H -4.58315700 0.12790400 3.53047100

H -5.25088600 -3.74885300 5.38869400

H -6.46125000 -1.36561400 4.83567900

H -0.27534700 0.99888800 -0.36253200

N -1.03829100 1.66986300 -0.51899100

P -2.46397900 1.20376900 -1.17281600

N -3.08715100 2.74645000 -1.18214100

C -2.28895600 3.72861000 -0.41308900

C -0.81381100 3.13196800 -0.45749500

C -4.43848400 3.08428600 -1.61377500

H -4.79315100 2.32986600 -2.31813400

H -5.14955700 3.12833700 -0.78065600

H -4.42848100 4.05826200 -2.11827000

H -2.27146600 4.65428500 -0.99591900

C -3.02678800 4.13894400 0.90578600

C -2.38831400 5.33973100 1.64104800

C -3.40426300 6.16092000 2.44674300

H -1.90715800 6.00356900 0.90933000

C 0.01573800 3.48363900 0.78721500

C 0.03029300 2.64696500 1.91043000

C 0.75241800 4.67621300 0.82742200

C 0.74734700 3.00597500 3.05449100

C 1.45547900 5.04194300 1.97478500

C 1.45480700 4.20815900 3.09590700

H -0.53665900 1.72190900 1.90933500

H 0.77171400 5.32901700 -0.03993900

H 0.74323800 2.33979300 3.91274200

H 2.00453400 5.98015700 1.99066900

H 2.00078100 4.49260400 3.99187700

C -0.03750400 3.54560300 -1.74178700

C 1.23256300 2.96950100 -1.92851000

C -0.51888700 4.40061300 -2.74016600

C 1.98761500 3.23861700 -3.06628700

C 0.23987200 4.67386600 -3.88511400

C 1.49463300 4.09586900 -4.05401900

H 1.64435100 2.30100700 -1.17877800

H -1.49244900 4.87129900 -2.65664000

H 2.96387500 2.77434800 -3.17592800

H -0.16100200 5.34386000 -4.64156000

H 2.08324900 4.30904400 -4.94233000

N -2.94372600 -0.13463900 -0.37197700

C -3.76132700 -1.07149700 -1.17095900

C -3.20872800 -0.85548000 -2.64739800

N -2.83043400 0.58026700 -2.66803100

H -2.83862300 -0.20512900 0.65823300

C -2.55852000 1.24828900 -3.93438100

H -1.62170800 0.91067600 -4.39271300

H -2.48310000 2.32467700 -3.76763600

H -3.38022900 1.05870000 -4.63585900

H -4.05447000 -0.96466900 -3.33182300

C -2.08926800 -1.74114800 -3.27892800

H -1.98511500 -1.29479100 -4.28116200

C -2.52240200 -3.20138100 -3.54425300

H -3.59666900 -3.22846300 -3.77564700

C -1.75752500 -3.85363600 -4.70390900

C -5.25556100 -0.64932700 -1.02932400

C -6.14189400 -0.41933600 -2.08772500

C -5.74736200 -0.50171500 0.28033300

C -7.46924000 -0.04056300 -1.84974400

C -7.06677800 -0.12982600 0.51954300

C -7.93779100 0.10744100 -0.54776500

H -5.82810100 -0.53153500 -3.11994000

H -5.09360100 -0.68951000 1.12700300

H -8.13229900 0.13313400 -2.69349800

H -7.41429000 -0.03551700 1.54476500

H -8.96880000 0.39720700 -0.36355200

C -3.60113200 -2.50388700 -0.63437000

C -4.56907100 -3.47457600 -0.93272000

C -2.51236300 -2.86955500 0.16694900

C -4.43570300 -4.78299800 -0.47197500

C -2.38730900 -4.17793400 0.64101200

C -3.34109800 -5.14185700 0.31707200

H -5.43822100 -3.20679100 -1.52579200

H -1.75952700 -2.13919800 0.43941000

H -5.19626300 -5.51880800 -0.71961500

H -1.54372500 -4.42957000 1.27773200

H -3.24293100 -6.15840500 0.68834600

H -2.11365000 -4.87597200 -4.87560700

H -1.89297100 -3.29387600 -5.63841300

H -0.68240700 -3.91053100 -4.50214000

H -2.91357200 7.00696200 2.94119500

H -4.19467000 6.56569300 1.80159600

H -3.88666900 5.56247900 3.22739400

C 4.03320900 -1.42770400 3.19839400

C 4.30307600 -2.67890500 3.77983200

C 5.04437300 -0.45289700 3.23951100

C 5.53225000 -2.94089700 4.38320500

H 3.54753700 -3.45836700 3.76454300

C 6.27151500 -0.71122200 3.84702500

H 4.86301000 0.51480000 2.78016900

C 6.52229400 -1.95756300 4.42327600

H 5.71527600 -3.91675400 4.82615400

H 7.03518500 0.06243300 3.86777500

H 7.47891200 -2.16149200 4.89742200

C -3.40639100 2.99117300 1.85001600

H -3.96153100 2.20434000 1.32998000

H -2.54551500 2.51856800 2.32272900

H -4.05864600 3.36713500 2.64601300

H -3.97602300 4.53192000 0.50756200

H -1.59342400 4.99535400 2.30828200

C -0.71445000 -1.63602700 -2.61255500

H -0.36857600 -0.59960100 -2.55457800

H -0.70810200 -2.03953800 -1.59994700

H 0.03323500 -2.18841300 -3.19015800

H -2.39162300 -3.80323400 -2.64086800

- **1da**-*SS* (HMe)


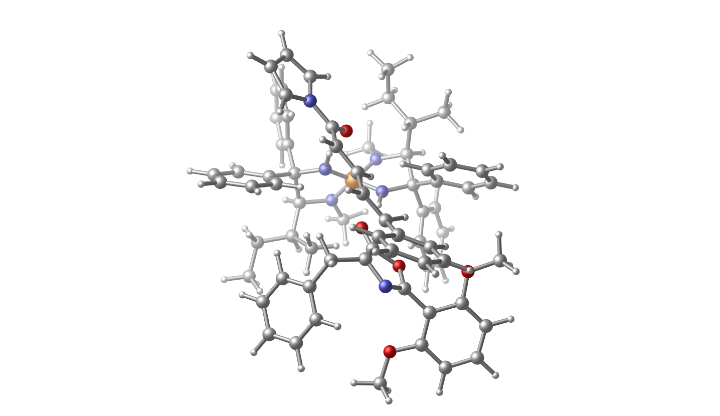

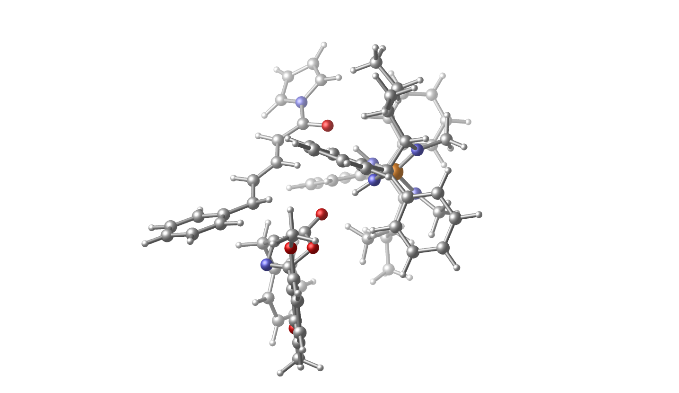


B3LYP/6-31G*

E = –3797.840213

Zero-point correction = 1.341502 (Hartree/Particle)

Thermal correction to Energy = 1.419984

Thermal correction to Enthalpy = 1.420929

Thermal correction to Gibbs Free Energy = 1.219235

Sum of electronic and zero-point Energies = –3796.498711

Sum of electronic and thermal Energies = –3796.420229

Sum of electronic and thermal Enthalpies = –3796.419285

Sum of electronic and thermal Free Energies = –3796.620978

0 1

O 0.89364000 -0.45749900 -0.06493900

C 2.10822400 -0.62243200 -0.00460500

C 2.94979800 -1.65484600 0.60085300

O 2.97927100 0.35855100 -0.46929400

N 4.29027000 -1.32027600 0.27317900

C 4.25668900 -0.17176300 -0.29471800

C 5.38403300 0.61889600 -0.82453600

C 5.73285000 1.85163100 -0.24231500

C 6.12605500 0.15087100 -1.92725200

C 6.80449900 2.60295300 -0.74145400

C 7.20285400 0.89600700 -2.42828000

C 7.52490900 2.11022400 -1.82732100

O 4.97357700 2.22920100 0.82435200

C 5.24532200 3.47966100 1.44073000

H 6.25391800 3.50319900 1.87355900

H 4.50155200 3.58470400 2.23046100

H 5.13507400 4.30747900 0.72889500

O 5.71100900 -1.02844000 -2.45992600

C 6.37484800 -1.53261900 -3.60766100

H 7.42788900 -1.76088900 -3.39738400

H 6.31787800 -0.82885700 -4.44859900

H 5.84725800 -2.45152000 -3.86712600

H 7.08229400 3.54777400 -0.29137700

H 7.77912400 0.54079300 -3.27344400

H 8.36011000 2.68790200 -2.21462200

C 2.56636400 -3.11950700 0.56518600

H 1.54862700 -3.20925500 0.95773900

H 3.23057800 -3.64499000 1.26315000

C 2.65329200 -3.79212600 -0.79620300

C 1.50609700 -4.31182700 -1.40841500

C 3.88714600 -3.95483900 -1.44617900

C 1.58080400 -4.97639600 -2.63558400

C 3.96450600 -4.62192600 -2.66878000

C 2.81239500 -5.13571100 -3.27052800

H 0.54200500 -4.20242400 -0.91748300

H 4.78230700 -3.54514100 -0.98859900

H 0.67642200 -5.37753200 -3.08676800

H 4.93203300 -4.75571100 -3.14812000

H 2.87669000 -5.66105000 -4.22025200

C 2.67746200 -1.13520700 2.56630900

C 1.47233700 -1.72367400 3.08419600

H 2.60888100 -0.06830000 2.35824700

H 1.56066600 -2.64297000 3.65970400

C 0.20396700 -1.21220700 2.88219100

H 0.11489700 -0.31671000 2.27694300

C -1.00004300 -1.73981400 3.37941400

H -0.96349300 -2.60550100 4.03222200

C -2.24100400 -1.14745600 3.08819600

O -2.44371200 -0.11973800 2.37961600

N -3.41702700 -1.76473900 3.65122400

C -3.55673900 -3.03407200 4.19080400

C -4.63630600 -1.11586000 3.74549900

C -4.85225800 -3.18548500 4.62107200

C -5.53804900 -1.96325000 4.34406600

H -2.73781500 -3.73529800 4.18307200

H -4.72512500 -0.09848500 3.40194300

H -5.26637500 -4.07669500 5.07454400

H -6.57072000 -1.73177200 4.57121300

H -0.20260600 1.07768800 -0.25442500

N -0.96298000 1.75248400 -0.40632500

P -2.39499400 1.30310100 -1.06244500

N -3.08070300 2.79699800 -0.84923800

C -2.30316800 3.66751000 0.06207500

C -0.79674100 3.21384800 -0.21447900

C -4.29458700 3.29247800 -1.48662800

H -4.70527700 2.51880900 -2.13677300

H -5.05871900 3.55474700 -0.75053000

H -4.06961600 4.18135600 -2.09148900

H -2.44607700 4.69171800 -0.29056200

C -2.76191500 3.62329900 1.55828900

C -4.25140300 3.26334400 1.75536900

C -4.60536300 2.96193400 3.21755600

H -4.49319100 2.37497500 1.16297600

C 0.15647300 3.47901900 0.96245900

C 0.24878700 2.57816000 2.03291600

C 0.91879000 4.65550100 1.01322600

C 1.06712300 2.85296600 3.13000700

C 1.72664000 4.93680800 2.11560200

C 1.80362300 4.03695800 3.18047800

H -0.34094300 1.66807100 2.02830400

H 0.87912800 5.36291500 0.19125700

H 1.11711800 2.13725600 3.94590900

H 2.29599500 5.86261600 2.13864800

H 2.42800300 4.25785200 4.04254400

C -0.21272300 3.82488400 -1.51612900

C 0.90991400 3.19576100 -2.08443700

C -0.70982900 4.97127700 -2.14789500

C 1.49717500 3.68561600 -3.24848900

C -0.12392700 5.46205800 -3.32077200

C 0.97867200 4.82054600 -3.87825900

H 1.33938600 2.31868400 -1.60832200

H -1.55712200 5.51143900 -1.73891100

H 2.36573200 3.17922900 -3.66081400

H -0.53588100 6.35137600 -3.79090300

H 1.43481100 5.20168400 -4.78795200

N -2.82216200 -0.12873400 -0.40359200

C -3.64167500 -0.97865500 -1.29456600

C -3.09506900 -0.60634400 -2.73999900

N -2.74849100 0.83447300 -2.61634100

H -2.80264600 -0.24541600 0.62749900

C -2.42743800 1.62138600 -3.80130400

H -1.45950200 1.34834400 -4.23722300

H -2.38825500 2.68032100 -3.53716000

H -3.20745400 1.48095500 -4.55919100

H -3.93864500 -0.67118900 -3.43346100

C -1.95408200 -1.40236600 -3.44820300

H -1.84045300 -0.84648900 -4.39296300

C -2.36349200 -2.82871900 -3.88211000

H -3.42945500 -2.83699900 -4.15073700

C -1.55506000 -3.34934700 -5.07831800

C -5.13803000 -0.58262100 -1.10782300

C -5.99211300 -0.12461200 -2.11789100

C -5.66132400 -0.68914900 0.19318700

C -7.32071900 0.22045500 -1.83885300

C -6.98095300 -0.34811700 0.47307000

C -7.82148100 0.11133400 -0.54500000

H -5.64946300 -0.02358100 -3.14205100

H -5.03232900 -1.04925100 1.00171900

H -7.95942800 0.57190900 -2.64531600

H -7.35021800 -0.45241200 1.48960500

H -8.85348000 0.37563900 -0.33052800

C -3.47652000 -2.45812700 -0.91282500

C -4.42480300 -3.39764300 -1.34495300

C -2.41410500 -2.89668500 -0.11330300

C -4.29736900 -4.74544500 -1.01533700

C -2.29670000 -4.24621700 0.23094000

C -3.22991800 -5.17653100 -0.22471100

H -5.27372000 -3.07259800 -1.93910200

H -1.67732100 -2.19114200 0.25372200

H -5.04131400 -5.45642100 -1.36493900

H -1.47562900 -4.55972300 0.86973300

H -3.13723000 -6.22476400 0.04630000

H -1.90265200 -4.34542600 -5.37570000

H -1.65650800 -2.68888800 -5.94925800

H -0.48813700 -3.43142500 -4.84423200

H -5.64944700 2.63823300 3.29951800

H -3.97071900 2.15658900 3.60386900

H -4.48348800 3.83462400 3.86798500

C 3.97847000 -1.48855000 3.20353800

C 4.23046700 -2.75300100 3.76440300

C 4.99908500 -0.52503800 3.27231400

C 5.45037800 -3.03792000 4.37600600

H 3.46825300 -3.52510300 3.72724500

C 6.21704100 -0.80664400 3.88784300

H 4.83364100 0.45173500 2.82675300

C 6.44938600 -2.06531200 4.44466600

H 5.61898400 -4.02337900 4.80314000

H 6.98824500 -0.04136500 3.92993900

H 7.39866900 -2.28711900 4.92547000

C -2.44232700 4.96965300 2.23324600

H -1.40310500 5.27293200 2.08933000

H -3.08551600 5.76234800 1.82565800

H -2.61905100 4.92220700 3.31154400

H -2.19988300 2.83034700 2.06017000

H -4.89009900 4.08037600 1.38805900

C -0.59259200 -1.35890100 -2.74903300

H -0.26047100 -0.33278500 -2.56699200

H -0.60079800 -1.87265400 -1.78760100

H 0.17173700 -1.83660400 -3.37005400

H -2.25615000 -3.52399500 -3.04512200

- **1da**-*SS* (HH)


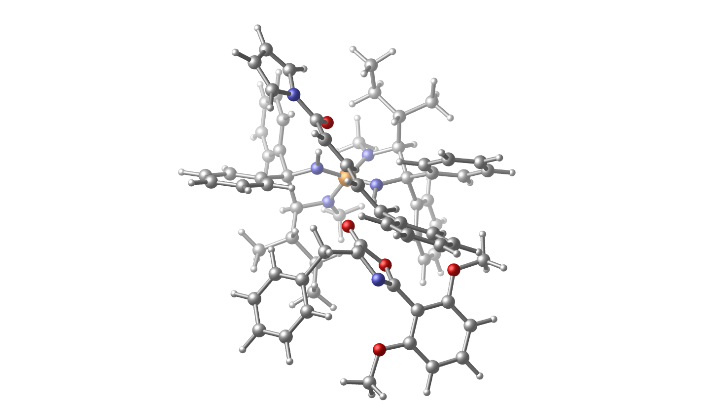

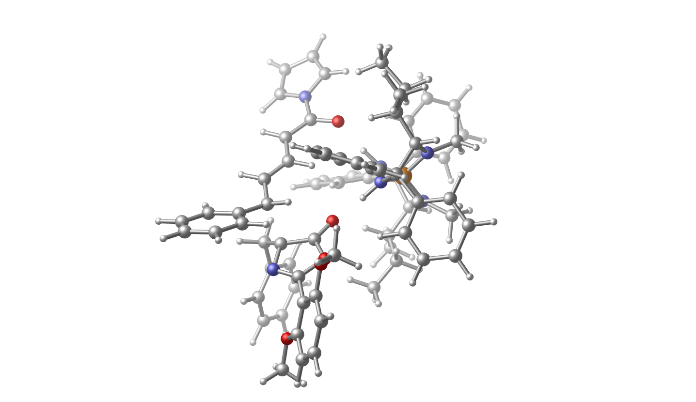


B3LYP/6-31G*

E = –3797.844356

Zero-point correction = 1.341466 (Hartree/Particle)

Thermal correction to Energy = 1.419924

Thermal correction to Enthalpy = 1.420868

Thermal correction to Gibbs Free Energy = 1.220206

Sum of electronic and zero-point Energies = –3796.502890

Sum of electronic and thermal Energies = –3796.424433

Sum of electronic and thermal Enthalpies = –3796.423488

Sum of electronic and thermal Free Energies = –3796.624151

PCM(toluene)-B3LYP-D3/6-311++G**//B3LYP/6-31G*@–30 °C

E = –3799.043499

G = –3797.788763

0 1

O 0.73152800 -0.64610800 -0.27385600

C 1.94083700 -0.78096800 -0.10768300

C 2.74563500 -1.69667500 0.70327800

O 2.82849200 0.14927200 -0.63240600

N 4.10408300 -1.36961900 0.44317500

C 4.09951000 -0.31268500 -0.28136900

C 5.24367100 0.43489800 -0.82942100

C 5.40391400 1.80724400 -0.55194600

C 6.18975300 -0.20762900 -1.65638800

C 6.48290200 2.52570200 -1.08195300

C 7.27604200 0.50703500 -2.18117100

C 7.40563600 1.86149400 -1.88701600

O 4.46152500 2.35481300 0.26641900

C 4.54032000 3.74423400 0.55582300

H 5.47512800 3.99279900 1.07482900

H 3.69245500 3.95962100 1.20588000

H 4.46077200 4.34607500 -0.35845700

O 5.95649900 -1.51860800 -1.91870300

C 6.82365000 -2.20413200 -2.80764200

H 7.84336200 -2.27000300 -2.40632600

H 6.85264300 -1.72347200 -3.79434500

H 6.40427100 -3.20628000 -2.90569200

H 6.61279300 3.57847000 -0.86544500

H 8.00741800 0.01948200 -2.81337800

H 8.24783500 2.41435800 -2.29480400

C 2.40417100 -3.16915300 0.81647400

H 1.35698400 -3.24887200 1.12224200

H 3.01295000 -3.58090200 1.63088200

C 2.64095100 -3.98571900 -0.44256700

C 1.56407300 -4.43851500 -1.21587500

C 3.94108900 -4.33451500 -0.83904900

C 1.77497700 -5.22114600 -2.35422900

C 4.15312300 -5.12155100 -1.97100400

C 3.07128700 -5.56782000 -2.73487900

H 0.54889500 -4.18483800 -0.92043500

H 4.78423700 -3.97663400 -0.25565400

H 0.92323500 -5.56840000 -2.93424100

H 5.16669300 -5.40051500 -2.25133700

H 3.23822200 -6.18556900 -3.61376200

C 2.26303800 -0.94220200 2.51918100

C 1.09661500 -1.59865400 3.05171300

H 2.08006900 0.07227500 2.16744900

H 1.25368400 -2.42967600 3.73683200

C -0.21146500 -1.26698600 2.74727100

H -0.38461600 -0.45846500 2.04220500

C -1.36174000 -1.88807800 3.26290800

H -1.23883100 -2.69817200 3.97367500

C -2.65929000 -1.45278600 2.93799700

O -2.96767700 -0.47432300 2.19746900

N -3.76081300 -2.17369100 3.51673300

C -3.75663600 -3.43024400 4.10555600

C -5.05292600 -1.67864300 3.55869700

C -5.03341100 -3.72246900 4.51648000

C -5.85714100 -2.60548800 4.17457500

H -2.85825900 -4.02505400 4.14137600

H -5.26003500 -0.69715200 3.16545500

H -5.34634700 -4.63930300 4.99915000

H -6.91700200 -2.49736700 4.36495300

H -0.10850200 1.09363400 -0.14444100

N -0.90571600 1.73803800 -0.16143300

P -2.23622000 1.30537300 -1.03110700

N -2.98903400 2.76288200 -0.78918800

C -2.31577300 3.58846700 0.24219600

C -0.78421600 3.20161200 0.04142200

C -4.20590300 3.22923300 -1.44294100

H -4.52138500 2.50120000 -2.19213700

H -5.02691500 3.35519500 -0.73296700

H -4.02091200 4.18950700 -1.94249700

H -2.47451400 4.63309700 -0.03862600

C -2.85913500 3.39913100 1.70067500

C -4.33266800 2.93918500 1.77566700

C -4.74888300 2.46844000 3.17517600

H -4.49015300 2.10331100 1.08772200

C 0.10195800 3.51082300 1.25849200

C 0.29901100 2.57287800 2.27964100

C 0.71219500 4.76884800 1.38489700

C 1.07898400 2.88039900 3.39715000

C 1.47763000 5.08374300 2.50824100

C 1.66792000 4.13847600 3.51929700

H -0.16795500 1.59782700 2.21507800

H 0.58856900 5.51097100 0.60249000

H 1.21870900 2.12913500 4.16921800

H 1.92880900 6.06953600 2.58922900

H 2.26953300 4.38053800 4.39119100

C -0.15786800 3.84948100 -1.22281600

C 1.01416400 3.27326900 -1.74500000

C -0.66561600 4.98981000 -1.85775500

C 1.63965900 3.80806200 -2.86961000

C -0.03980500 5.52706200 -2.98852900

C 1.11278100 4.93763800 -3.50153800

H 1.45503200 2.40090200 -1.27064700

H -1.55573700 5.48483100 -1.48386200

H 2.54271100 3.33793200 -3.24951500

H -0.46084400 6.41012500 -3.46235600

H 1.59875200 5.35357400 -4.37995000

N -2.71680000 -0.17327900 -0.53697500

C -3.39827600 -0.95366500 -1.59532400

C -2.56025700 -0.51974200 -2.87994800

N -2.32354500 0.93013100 -2.64031500

H -2.80600500 -0.39507700 0.47457200

C -2.24452500 1.87156700 -3.75143400

H -1.36836800 1.68790100 -4.37756400

H -2.17050300 2.88959000 -3.36493900

H -3.14562500 1.79640500 -4.37446500

H -3.19084000 -0.57868600 -3.77125500

C -1.27660900 -1.36967200 -3.15560800

H -0.90670800 -1.74260400 -2.19511200

C -0.11531100 -0.56503300 -3.77973200

H 0.09600200 0.30528100 -3.14989700

C 1.18203800 -1.36958100 -3.93229800

C -4.88423000 -0.50941000 -1.67890100

C -5.58411900 -0.30736300 -2.87504200

C -5.58400600 -0.34484200 -0.47053900

C -6.93123200 0.07263500 -2.86794300

C -6.92651800 0.02722200 -0.46212800

C -7.60751600 0.24505400 -1.66277100

H -5.09975500 -0.45161200 -3.83535500

H -5.07233900 -0.51649300 0.47229900

H -7.44706500 0.22723500 -3.81225400

H -7.44264800 0.14140500 0.48736600

H -8.65400100 0.53773100 -1.65673700

C -3.33801800 -2.45099400 -1.24427200

C -4.29014600 -3.34229500 -1.76262200

C -2.35048000 -2.95547700 -0.38679800

C -4.24733600 -4.70003400 -1.44748400

C -2.31957500 -4.31297400 -0.05875700

C -3.26228600 -5.19194500 -0.59025900

H -5.08103100 -2.97499000 -2.40880500

H -1.60414400 -2.29225200 0.03608400

H -4.99684100 -5.36935800 -1.86156800

H -1.55831200 -4.67446100 0.62692600

H -3.23862800 -6.24647500 -0.32928300

H 1.99980400 -0.71421800 -4.25296300

H 1.47662900 -1.82907400 -2.98339300

H 1.09187000 -2.16817500 -4.67601600

H -5.79374300 2.13596900 3.16854200

H -4.13026200 1.62045100 3.48675000

H -4.66596200 3.25842000 3.92934600

C 3.53880000 -1.02251700 3.29043000

C 3.91143300 -2.16115700 4.02519200

C 4.40752200 0.08167600 3.30351200

C 5.10236700 -2.19095000 4.74864100

H 3.26762000 -3.03533100 4.03523700

C 5.59606700 0.05561000 4.03108400

H 4.14856000 0.96591200 2.72718000

C 5.94982300 -1.08142700 4.75881900

H 5.36752700 -3.08403700 5.30903800

H 6.24903300 0.92497000 4.02669300

H 6.87637500 -1.10430800 5.32666800

C -2.67246600 4.70318900 2.49731500

H -1.64251200 5.06494800 2.47779000

H -3.31677100 5.49434900 2.08869700

H -2.94729100 4.56411800 3.54680800

H -2.27359300 2.60319600 2.17291500

H -4.99536300 3.75554800 1.45136900

C -1.64262100 -2.56806700 -4.04993000

H -2.49895700 -3.12561000 -3.66458500

H -1.88842100 -2.22794000 -5.06605300

H -0.80641000 -3.26887000 -4.12725800

H -0.41320700 -0.18527200 -4.76827000

- **1da**-*SS* (MeH)


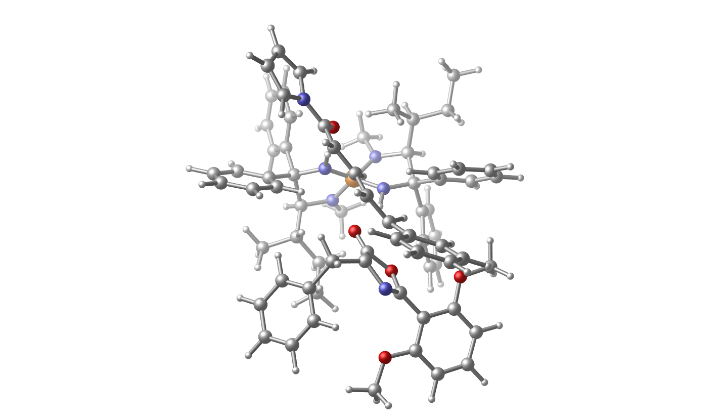

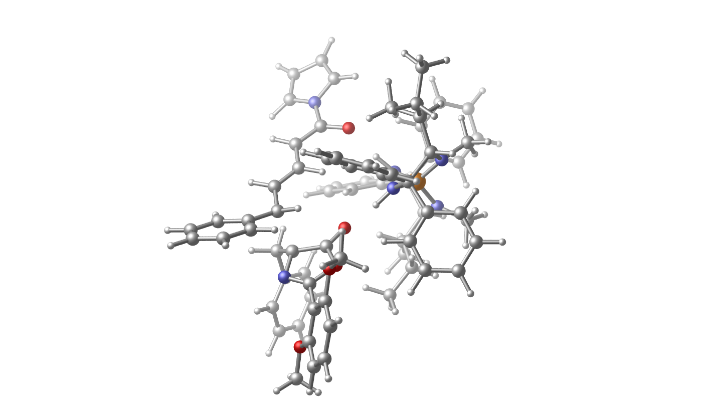


B3LYP/6-31G*

E = –3797.841891

Zero-point correction = 1.340835 (Hartree/Particle)

Thermal correction to Energy = 1.419647

Thermal correction to Enthalpy = 1.420591

Thermal correction to Gibbs Free Energy = 1.218302

Sum of electronic and zero-point Energies = –3796.501056

Sum of electronic and thermal Energies = –3796.422244

Sum of electronic and thermal Enthalpies = –3796.421300

Sum of electronic and thermal Free Energies = –3796.623589

0 1

O 0.67612400 -0.70839400 -0.26191000

C 1.88415800 -0.83862000 -0.08474300

C 2.68634900 -1.73540800 0.75236300

O 2.77377000 0.08255200 -0.62200000

N 4.04672800 -1.41041000 0.49164100

C 4.04361900 -0.36840200 -0.25310100

C 5.18741000 0.37562900 -0.80776800

C 5.34773600 1.75017200 -0.54090500

C 6.13258000 -0.27324700 -1.63053400

C 6.42616300 2.46449300 -1.07796500

C 7.21849300 0.43713300 -2.16193800

C 7.34820300 1.79382900 -1.87867100

O 4.40525800 2.30547600 0.27343000

C 4.50627900 3.69046800 0.58072800

H 5.44924000 3.91707600 1.09465000

H 3.66720100 3.90956100 1.24080000

H 4.42673400 4.30566200 -0.32436800

O 5.89902000 -1.58635800 -1.88095100

C 6.76563100 -2.28022200 -2.76412500

H 7.78567800 -2.34187000 -2.36306000

H 6.79338000 -1.80904700 -3.75540000

H 6.34642500 -3.28335400 -2.85206900

H 6.55613800 3.51904400 -0.87047500

H 7.94934400 -0.05546100 -2.79084100

H 8.18977800 2.34365300 -2.29177000

C 2.35037000 -3.20802200 0.88429200

H 1.30518300 -3.28799800 1.19693600

H 2.96487900 -3.60714100 1.70059200

C 2.58437500 -4.03785000 -0.36643300

C 1.50580200 -4.50126500 -1.13112600

C 3.88393800 -4.38768000 -0.76396300

C 1.71479500 -5.29516500 -2.26199600

C 4.09398800 -5.18570300 -1.88858900

C 3.01057800 -5.64246200 -2.64391300

H 0.49101600 -4.24735600 -0.83468400

H 4.72834500 -4.02202200 -0.18721100

H 0.86189300 -5.65086500 -2.83516600

H 5.10725300 -5.46513900 -2.16957700

H 3.17587300 -6.26881500 -3.51699600

C 2.20225700 -0.95244200 2.54104500

C 1.03869600 -1.60063900 3.09203500

H 2.01630800 0.05574900 2.17220800

H 1.20309400 -2.40998500 3.80093100

C -0.27329500 -1.28875400 2.78265500

H -0.45690500 -0.50336800 2.05311800

C -1.41340900 -1.89845800 3.33324500

H -1.27630100 -2.66300100 4.09051200

C -2.71936900 -1.49774200 2.99515100

O -3.04810000 -0.58830200 2.18421700

N -3.80738300 -2.16688700 3.65662500

C -3.79821100 -3.38117700 4.32662500

C -5.08934900 -1.64902600 3.71046000

C -5.06373500 -3.62554300 4.80018700

C -5.88380500 -2.52007200 4.41451000

H -2.90659200 -3.98594400 4.36959800

H -5.29277400 -0.69021300 3.26223200

H -5.37200300 -4.50397800 5.35233300

H -6.93437200 -2.38237500 4.63502400

H -0.14870100 1.06076800 -0.25715300

N -0.93812600 1.71611900 -0.26489000

P -2.28134800 1.27442700 -1.11005600

N -2.97973500 2.77834700 -0.98693000

C -2.24859700 3.70720200 -0.08990800

C -0.75209800 3.18140000 -0.16400500

C -4.33832700 3.09069000 -1.41770000

H -4.63328200 2.41079300 -2.21926000

H -5.07165100 2.99068300 -0.60968500

H -4.37307700 4.11768800 -1.80091700

H -2.27460200 4.69374300 -0.56271100

C -3.00909900 3.90443700 1.26107100

C -2.51699000 5.11550600 2.08862100

C -3.61130100 5.71711000 2.98062500

H -2.15112000 5.90102400 1.41201100

C 0.08483600 3.55006200 1.07043500

C 0.30842700 2.64429700 2.11316100

C 0.64234300 4.83399300 1.17000800

C 1.05106300 3.01553500 3.23767800

C 1.36853400 5.21275200 2.29830200

C 1.57644800 4.30356300 3.33946700

H -0.10733900 1.64541400 2.05983000

H 0.50436000 5.54573800 0.36151400

H 1.21193600 2.29083800 4.03082300

H 1.77640500 6.21845900 2.36108300

H 2.14647600 4.59547100 4.21736300

C 0.00098400 3.67959800 -1.43385400

C 1.25389400 3.09787000 -1.70209600

C -0.47082000 4.65181700 -2.32343500

C 1.99734600 3.46797300 -2.81995800

C 0.27682900 5.02802700 -3.44614600

C 1.51158400 4.43843100 -3.70083400

H 1.66734400 2.34902100 -1.03317500

H -1.42858800 5.13605300 -2.16805000

H 2.95860000 2.99376800 -2.99840900

H -0.11723500 5.78612200 -4.11821400

H 2.09072500 4.72987600 -4.57290400

N -2.78781800 -0.16196900 -0.52704100

C -3.46645300 -1.01227700 -1.53497900

C -2.64425400 -0.65121800 -2.84988900

N -2.40553000 0.80867400 -2.69454000

H -2.82630300 -0.37217600 0.49071400

C -2.39079600 1.68676400 -3.85837000

H -1.53031900 1.49230200 -4.50332200

H -2.33851700 2.72586800 -3.53001900

H -3.30893700 1.54790000 -4.44428700

H -3.28544000 -0.76151800 -3.72853300

C -1.35971900 -1.50738100 -3.09997000

H -0.96015700 -1.81227400 -2.12727800

C -0.22729500 -0.72979400 -3.80679900

H -0.01017500 0.18206300 -3.24064500

C 1.07705900 -1.52539800 -3.94603400

C -4.95629200 -0.58428600 -1.62369600

C -5.66558800 -0.41316200 -2.81860000

C -5.64598100 -0.39276900 -0.41313900

C -7.01354900 -0.03509700 -2.80934400

C -6.98869000 -0.02273600 -0.40269600

C -7.67973100 0.16574500 -1.60308900

H -5.18848700 -0.57864400 -3.77916800

H -5.12407500 -0.54475100 0.52780300

H -7.53764100 0.09572300 -3.75270500

H -7.49817800 0.11210800 0.54777100

H -8.72670900 0.45675400 -1.59546300

C -3.38817100 -2.48649300 -1.10087300

C -4.35108700 -3.40679600 -1.54336300

C -2.37149900 -2.94191000 -0.25048100

C -4.29195000 -4.74625500 -1.15991500

C -2.32454900 -4.28011100 0.14693200

C -3.27876200 -5.18890100 -0.30884200

H -5.16190200 -3.07593400 -2.18435100

H -1.61473000 -2.25662800 0.11396700

H -5.05015900 -5.43895300 -1.51557400

H -1.54204100 -4.60205800 0.82817100

H -3.24191000 -6.22816600 0.00629800

H 1.87313600 -0.88294100 -4.33920000

H 1.40896700 -1.91246700 -2.97725300

H 0.97573500 -2.37610000 -4.62794900

H -3.22568900 6.57555700 3.54216600

H -4.46554000 6.06521300 2.38589300

H -3.98786000 4.99194400 3.71006300

C 3.47571700 -1.01060400 3.32069000

C 3.85587100 -2.13774600 4.06896800

C 4.33242600 0.10263000 3.32819200

C 5.04212200 -2.14739200 4.80059300

H 3.22156400 -3.01880400 4.08289600

C 5.51628400 0.09703800 4.06402200

H 4.06830500 0.97764200 2.74026200

C 5.87740800 -1.02862100 4.80554400

H 5.31326600 -3.03205400 5.37140200

H 6.15981400 0.97340900 4.05514000

H 6.80028300 -1.03574900 5.37976300

C -3.19727600 2.64419700 2.11242500

H -3.63343400 1.81739300 1.54810500

H -2.26040300 2.28403300 2.53941500

H -3.87914000 2.85173500 2.94417500

H -4.01205800 4.19602800 0.91133400

H -1.66457200 4.82651500 2.71016900

C -1.73077200 -2.76572700 -3.90580700

H -2.57067500 -3.30788800 -3.46645300

H -2.00299400 -2.49535200 -4.93609600

H -0.88658200 -3.45931700 -3.95693000

H -0.55949000 -0.41673500 -4.80787600

- **1da**-*SS* (HEt)


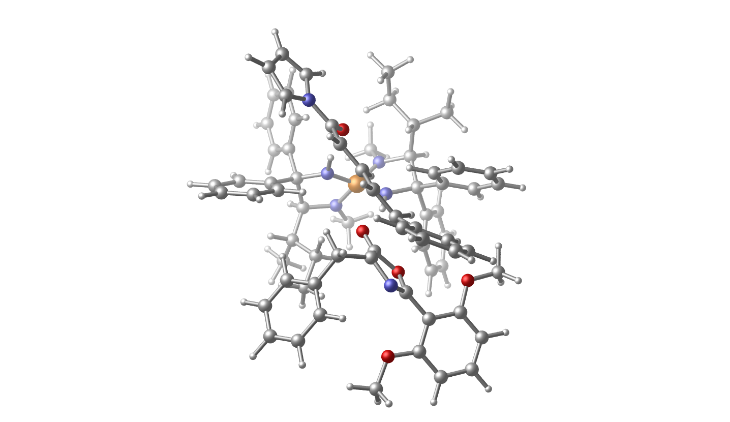

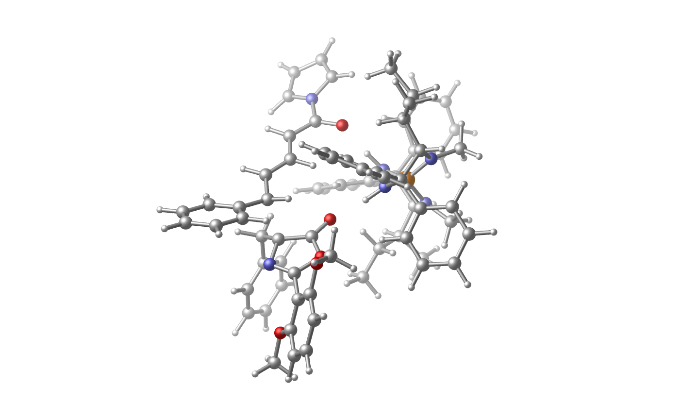


B3LYP/6-31G*

E = –3797.842438

Zero-point correction = 1.341957 (Hartree/Particle)

Thermal correction to Energy = 1.420464

Thermal correction to Enthalpy = 1.421408

Thermal correction to Gibbs Free Energy = 1.219077

Sum of electronic and zero-point Energies = –3796.500481

Sum of electronic and thermal Energies = –3796.421975

Sum of electronic and thermal Enthalpies = –3796.421031

Sum of electronic and thermal Free Energies = –3796.623361

PCM(toluene)-B3LYP-D3/6-311++G**//B3LYP/6-31G*@–30 °C

E = –3799.038823

G = –3797.784906

0 1

O 0.82802700 -0.53449600 -0.19224300

C 2.04378200 -0.67681500 -0.08932800

C 2.88273100 -1.62255800 0.65108300

O 2.90883200 0.27046500 -0.62073100

N 4.22814400 -1.27926000 0.35305000

C 4.19282900 -0.19964100 -0.33643100

C 5.31577100 0.56101700 -0.91136200

C 5.51042600 1.91640100 -0.58098100

C 6.20564100 -0.05402000 -1.81749600

C 6.57067200 2.64533000 -1.13418900

C 7.27261100 0.67060900 -2.36769400

C 7.43857700 2.00792900 -2.01807500

O 4.61918200 2.43589500 0.30898700

C 4.72271100 3.81195400 0.64672700

H 5.67903000 4.03386900 1.13821900

H 3.90136200 4.00821900 1.33590500

H 4.61409000 4.44929100 -0.24019500

O 5.93742900 -1.34800100 -2.12583700

C 6.74513800 -2.00600700 -3.08852800

H 7.78544600 -2.09524700 -2.74922000

H 6.72295800 -1.48741900 -4.05612300

H 6.31020400 -3.00017800 -3.19880000

H 6.72794300 3.68504500 -0.87641000

H 7.96056300 0.20422900 -3.06169500

H 8.26598200 2.56868800 -2.44499700

C 2.55353400 -3.10090200 0.73272000

H 1.49433600 -3.19428500 0.98808500

H 3.12462900 -3.51565300 1.57268100

C 2.86232500 -3.91477200 -0.51294800

C 1.83154100 -4.40651500 -1.32310900

C 4.18623000 -4.23621600 -0.85013500

C 2.10863800 -5.20211700 -2.43794000

C 4.46517100 -5.03534000 -1.95899100

C 3.42753300 -5.52215200 -2.75886600

H 0.79920500 -4.16989600 -1.07704200

H 4.99504500 -3.84835900 -0.23842900

H 1.29089500 -5.57832100 -3.04792400

H 5.49648000 -5.29236000 -2.19142500

H 3.64598400 -6.15029400 -3.61893300

C 2.45745200 -0.94835500 2.52227300

C 1.31482600 -1.64393900 3.05635100

H 2.25199200 0.07604800 2.21613200

H 1.49658800 -2.49593700 3.70859600

C 0.00007800 -1.32799200 2.76931300

H -0.18516400 -0.50170600 2.09062600

C -1.14334900 -1.98329900 3.25688700

H -1.02096600 -2.81747100 3.93977800

C -2.44136600 -1.54960400 2.93595500

O -2.75712900 -0.54051900 2.23925000

N -3.53579700 -2.31566900 3.47172800

C -3.52555300 -3.62963700 3.91388600

C -4.81525900 -1.81254000 3.63009100

C -4.78789800 -3.95245400 4.34827200

C -5.60660400 -2.79423300 4.17541900

H -2.63567100 -4.23321500 3.83232900

H -5.02059700 -0.78710300 3.36959100

H -5.09524900 -4.91558600 4.73465500

H -6.65394200 -2.69525300 4.42955100

H -0.21870300 1.07616400 -0.21123000

N -1.01083100 1.72976800 -0.23726000

P -2.40548500 1.32156400 -1.00524400

N -3.13995200 2.76585600 -0.65157200

C -2.39949700 3.56395300 0.35063500

C -0.88161600 3.18073500 0.04626400

C -4.38461700 3.26070600 -1.22677300

H -4.74258300 2.55701900 -1.98051300

H -5.16639600 3.37085300 -0.47107800

H -4.21835800 4.23459800 -1.70618800

H -2.56776100 4.61500800 0.10110500

C -2.86156200 3.35105800 1.83357500

C -4.32583000 2.88058000 1.98384500

C -4.64903200 2.36724700 3.39296600

H -4.52066900 2.06335900 1.28269000

C 0.06107100 3.42142200 1.23586700

C 0.24280400 2.45057700 2.22893100

C 0.72484200 4.64967000 1.37690800

C 1.05834000 2.69797700 3.33508100

C 1.52757800 4.90482700 2.48982400

C 1.70059800 3.92838800 3.47365400

H -0.27086200 1.50020500 2.15302100

H 0.61219300 5.41634200 0.61705300

H 1.18179900 1.92304600 4.08625200

H 2.01842900 5.87026100 2.58515900

H 2.32890500 4.12489900 4.33818000

C -0.31567300 3.88961300 -1.21268800

C 0.82436600 3.32937600 -1.81739600

C -0.84219600 5.06196800 -1.76862300

C 1.40058800 3.91081700 -2.94448200

C -0.26681600 5.64521400 -2.90380800

C 0.85386300 5.07155000 -3.49847300

H 1.27587400 2.43145100 -1.40446900

H -1.70664100 5.54845500 -1.32888000

H 2.28121000 3.45407900 -3.38803200

H -0.70176900 6.55216400 -3.31590300

H 1.30087700 5.52410600 -4.37948100

N -2.81395300 -0.18440300 -0.52780400

C -3.60729500 -0.93683300 -1.51893500

C -3.06856400 -0.37284100 -2.91280500

N -2.68112300 1.02644000 -2.61132700

H -2.80684800 -0.42774400 0.48153200

C -2.48655800 1.99315900 -3.68461400

H -1.57170800 1.79744300 -4.25333400

H -2.40953700 2.99836800 -3.26549100

H -3.34020900 1.96356500 -4.37291600

H -3.92255100 -0.30357100 -3.58962100

C -1.98844300 -1.20705000 -3.67027700

H -2.23541500 -2.25726400 -3.47661200

C -0.55622600 -0.96786400 -3.16103100

H -0.52368200 -1.13144200 -2.08067900

C 0.50441500 -1.85895000 -3.81784900

C -5.12048200 -0.63071600 -1.31127200

C -6.04986600 -0.49019000 -2.35101200

C -5.59819700 -0.55093300 0.00835800

C -7.40258300 -0.25164100 -2.08592100

C -6.94663200 -0.32081400 0.27359700

C -7.85758400 -0.16207500 -0.77293500

H -5.74673000 -0.57693500 -3.38976900

H -4.91279900 -0.67846000 0.84045700

H -8.09681400 -0.14187900 -2.91517500

H -7.28448700 -0.27613200 1.30541200

H -8.90875400 0.01983900 -0.56613400

C -3.37631700 -2.44819200 -1.33803600

C -4.24069200 -3.36872100 -1.95087400

C -2.31598800 -2.93980400 -0.56544500

C -4.04569000 -4.74115600 -1.80518500

C -2.13525400 -4.31608600 -0.40370900

C -2.99312500 -5.22229100 -1.02485500

H -5.08438400 -3.01410200 -2.53498600

H -1.62520500 -2.25781300 -0.08292000

H -4.72884800 -5.43372500 -2.28988600

H -1.32117500 -4.67291300 0.22106900

H -2.85019800 -6.29162600 -0.89527000

H 1.47249400 -1.72158000 -3.32593400

H 0.24425900 -2.92040400 -3.73071000

H 0.63873800 -1.63233900 -4.88177000

H -5.69017800 2.02738800 3.44518000

H -4.00463600 1.51689400 3.64035700

H -4.52063100 3.13599600 4.16259900

C 3.76046800 -1.04936800 3.24078800

C 4.15962100 -2.20624000 3.93223600

C 4.63216100 0.05297600 3.24616800

C 5.37940300 -2.25613300 4.60502600

H 3.51382800 -3.07872400 3.94963500

C 5.84938900 0.00663300 3.92307300

H 4.35195300 0.95176600 2.70342400

C 6.22975300 -1.14896000 4.60720600

H 5.66446600 -3.16305000 5.13246100

H 6.50401900 0.87469300 3.91357300

H 7.17888000 -1.18749500 5.13557500

C -2.63190000 4.64382000 2.63689200

H -1.60307800 5.00364100 2.56617800

H -3.29509000 5.44130000 2.27328800

H -2.85026900 4.49169100 3.69788100

H -2.25033600 2.55204500 2.26444400

H -5.01268100 3.70045600 1.72583900

C -2.11747000 -1.01809500 -5.19600000

H -1.50455100 -1.75184800 -5.72789000

H -3.15328500 -1.16248000 -5.52839000

H -1.79613500 -0.02478400 -5.52894800

H -0.27943200 0.08409500 -3.31438200

- **1da**-*SS* (EtEt)


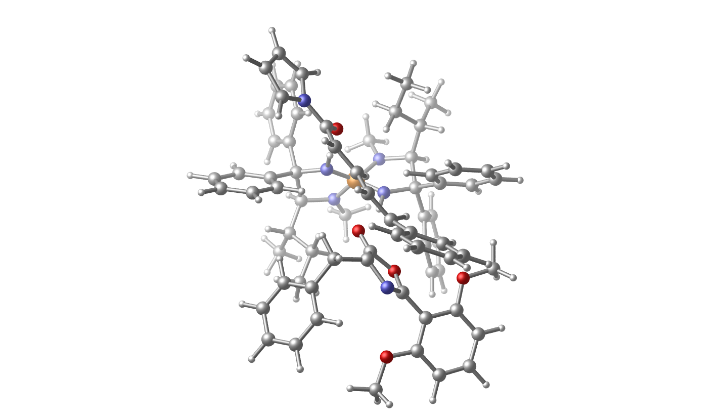

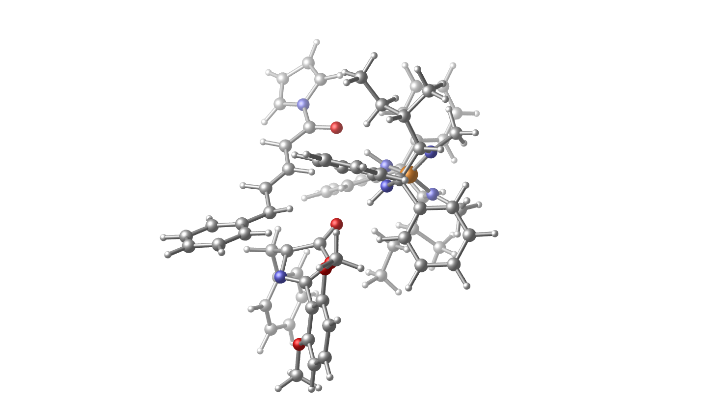


B3LYP/6-31G*

E = –3797.843973

Zero-point correction = 1.341576 (Hartree/Particle)

Thermal correction to Energy = 1.420239

Thermal correction to Enthalpy = 1.421184

Thermal correction to Gibbs Free Energy = 1.218851

Sum of electronic and zero-point Energies = –3796.502397

Sum of electronic and thermal Energies = –3796.423733

Sum of electronic and thermal Enthalpies = –3796.422789

Sum of electronic and thermal Free Energies = –3796.625121

PCM(toluene)-B3LYP-D3/6-311++G**//B3LYP/6-31G*@–30 °C

E = –3799.041826

G = –3797.788136

0 1

O 0.77880200 -0.59460200 -0.25254100

C 1.99354500 -0.73681200 -0.13904800

C 2.82897000 -1.71835500 0.55849900

O 2.85967100 0.24694100 -0.59748700

N 4.17665800 -1.34755700 0.30259400

C 4.14344600 -0.22797300 -0.31951500

C 5.26473300 0.58168900 -0.82566600

C 5.41749200 1.92585700 -0.43035300

C 6.19574000 0.02800400 -1.73060400

C 6.47314400 2.70365900 -0.92256500

C 7.25918400 0.80160700 -2.21753600

C 7.38099500 2.12626600 -1.80764800

O 4.49200000 2.38562100 0.45860200

C 4.58539900 3.73461000 0.89766800

H 5.53754100 3.92279400 1.41031200

H 3.75947900 3.87585200 1.59452500

H 4.47777900 4.43478900 0.05961400

O 5.97111700 -1.25738200 -2.10142900

C 6.82804600 -1.85452200 -3.06154800

H 7.85754600 -1.93435700 -2.68878300

H 6.82711900 -1.29584200 -4.00677500

H 6.42162700 -2.85263200 -3.22955700

H 6.59662200 3.73490600 -0.61720300

H 7.97851900 0.38235900 -2.90975200

H 8.20502800 2.72491900 -2.18693600

C 2.51082400 -3.20192300 0.53809100

H 1.45121400 -3.32064000 0.78125000

H 3.08115100 -3.66770000 1.35127000

C 2.83098400 -3.92646700 -0.75832100

C 1.80763400 -4.35784400 -1.61127900

C 4.15791100 -4.22536300 -1.10409100

C 2.09563000 -5.07202000 -2.77725200

C 4.44751100 -4.94346400 -2.26435900

C 3.41748000 -5.37002800 -3.10717600

H 0.77300700 -4.13793800 -1.35857900

H 4.96075900 -3.88406100 -0.45757300

H 1.28418900 -5.40269900 -3.42104000

H 5.48078900 -5.18525700 -2.50430100

H 3.64390900 -5.93528900 -4.00782400

C 2.37389900 -1.16706100 2.45172700

C 1.23230100 -1.90463900 2.92874200

H 2.16150700 -0.12665100 2.21060300

H 1.41653300 -2.79740600 3.52327400

C -0.08421000 -1.57695500 2.66203400

H -0.27709900 -0.70517000 2.04345400

C -1.22102100 -2.26752000 3.11383200

H -1.08828600 -3.13334300 3.75390100

C -2.52422900 -1.82343800 2.82863400

O -2.85023000 -0.79461400 2.17013200

N -3.61198200 -2.59668400 3.36557200

C -3.60070200 -3.91623100 3.79093300

C -4.88660100 -2.09045000 3.54875500

C -4.85810500 -4.23916400 4.23909300

C -5.67437400 -3.07529500 4.09279500

H -2.71461900 -4.52259800 3.69098600

H -5.09088100 -1.06184500 3.29986000

H -5.16391800 -5.20569800 4.61819100

H -6.71796700 -2.97567500 4.36148700

H -0.18931800 1.07655300 -0.27602400

N -0.95908800 1.75556100 -0.21625900

P -2.38773900 1.43913000 -0.96493000

N -3.04320500 2.91673100 -0.57880500

C -2.21639400 3.72877700 0.33854700

C -0.73764600 3.18512600 0.10057500

C -4.39751400 3.32471800 -0.93553200

H -4.73415000 2.74957600 -1.80106200

H -5.11316200 3.15846100 -0.12347800

H -4.40322500 4.38911300 -1.19804900

H -2.24506100 4.75728400 -0.03136100

C -2.73766000 3.79624400 1.81090300

C -3.23869200 2.44745400 2.36065100

C -3.45696900 2.43148400 3.87834800

H -2.52949900 1.65176500 2.12179700

C 0.12777700 3.35428600 1.35758600

C 0.31883700 2.31294500 2.27084000

C 0.68799200 4.60882100 1.64529000

C 1.05056400 2.51416000 3.44430800

C 1.40216500 4.81711800 2.82512600

C 1.59031200 3.76809800 3.72962600

H -0.12035700 1.34235100 2.08007400

H 0.56271400 5.42933000 0.94446100

H 1.18732600 1.68489300 4.13258200

H 1.81488200 5.80067000 3.03546500

H 2.15056400 3.92845900 4.64674600

C -0.01736400 3.85260800 -1.10551400

C 1.22995900 3.31862700 -1.47833000

C -0.51707800 4.92796900 -1.84926200

C 1.94235900 3.83621300 -2.55680500

C 0.19910200 5.45102100 -2.93321600

C 1.42938100 4.90837600 -3.29274100

H 1.66008800 2.49112100 -0.92153800

H -1.47111600 5.38379800 -1.60571000

H 2.90140200 3.39753900 -2.81887600

H -0.21508400 6.28687900 -3.49128400

H 1.98463000 5.31445300 -4.13396000

N -2.85431100 -0.06229800 -0.52744100

C -3.68102100 -0.76296900 -1.53024100

C -3.15001400 -0.16998500 -2.91423500

N -2.71366200 1.20385100 -2.57290200

H -2.81825800 -0.37900500 0.45964500

C -2.53889000 2.20540200 -3.61628200

H -1.64098500 2.02285200 -4.21608500

H -2.44377700 3.19359800 -3.16222900

H -3.41073100 2.20517500 -4.28197000

H -4.01438500 -0.05068000 -3.57073800

C -2.11073800 -1.00830400 -3.72275600

H -2.37663500 -2.05779000 -3.55233400

C -0.65962300 -0.81605700 -3.24768800

H -0.60257100 -0.99668700 -2.17102600

C 0.35607400 -1.72932700 -3.94385400

C -5.18013800 -0.41839400 -1.28776900

C -6.12379700 -0.23599300 -2.30790900

C -5.62855600 -0.33675100 0.04201900

C -7.46163500 0.04805500 -2.01455600

C -6.96251500 -0.05901700 0.33502500

C -7.88705900 0.14341400 -0.69198100

H -5.84333000 -0.32384600 -3.35301000

H -4.93115300 -0.50506700 0.85697900

H -8.16756100 0.18982100 -2.82893400

H -7.28013100 -0.01084300 1.37325700

H -8.92648100 0.36248000 -0.46288700

C -3.48277200 -2.28441900 -1.40229200

C -4.39398600 -3.16470800 -2.00575400

C -2.39917800 -2.82511600 -0.69719600

C -4.22213500 -4.54539000 -1.91736900

C -2.24034600 -4.20927000 -0.59326700

C -3.14506700 -5.07535800 -1.20542600

H -5.25585200 -2.77300400 -2.53709500

H -1.67328200 -2.17698800 -0.22008400

H -4.94222600 -5.20609600 -2.39301300

H -1.40682700 -4.60353800 -0.01860200

H -3.01933800 -6.15133600 -1.12066300

H 1.34041100 -1.62954600 -3.47594600

H 0.06397200 -2.78313200 -3.86417800

H 0.46981800 -1.49199500 -5.00774000

H -3.72130900 1.42029100 4.20348900

H -2.54154700 2.72126200 4.40921100

H -4.25822900 3.10845400 4.19658300

C 3.66877900 -1.29737600 3.18196300

C 4.07770100 -2.49255100 3.79805100

C 4.52179000 -0.18468500 3.27530000

C 5.28949100 -2.56946100 4.48276100

H 3.44610800 -3.37405700 3.74586200

C 5.73085800 -0.25815600 3.96444600

H 4.23382800 0.74473500 2.79118500

C 6.12145300 -1.45198800 4.57280000

H 5.58278500 -3.50601500 4.95050500

H 6.37091300 0.61875300 4.02384000

H 7.06417800 -1.51205800 5.11051500

C -3.78468900 4.91714600 1.97326700

H -3.42084600 5.86769600 1.56282100

H -4.73455400 4.68121300 1.48136100

H -4.00237000 5.08621700 3.03226900

H -1.87914200 4.10212400 2.41762000

H -4.17680800 2.17155200 1.86002200

C -2.27430900 -0.77079200 -5.23829200

H -1.68889100 -1.49884200 -5.80779300

H -3.32067800 -0.88564100 -5.54848600

H -1.94208600 0.22645400 -5.54863100

H -0.35649700 0.22954100 -3.39551800

- **1da**-*SS* (EtH)


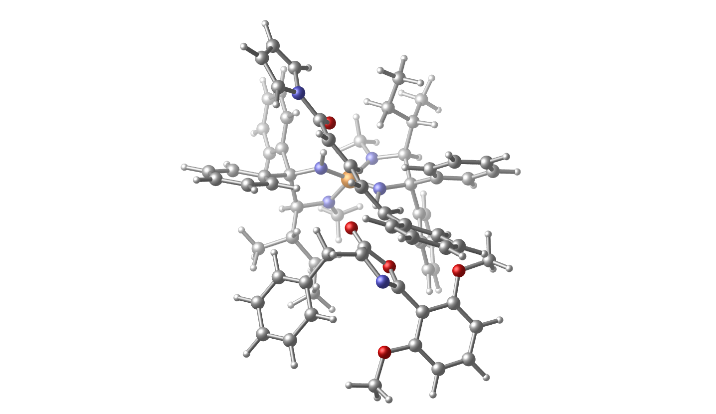

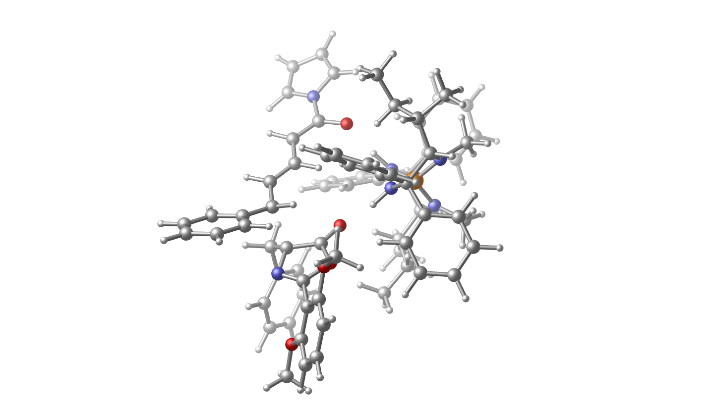


B3LYP/6-31G*

E = –3797.846039

Zero-point correction = 1.340972 (Hartree/Particle)

Thermal correction to Energy = 1.419679

Thermal correction to Enthalpy = 1.420623

Thermal correction to Gibbs Free Energy = 1.218482

Sum of electronic and zero-point Energies = –3796.505067

Sum of electronic and thermal Energies = –3796.426360

Sum of electronic and thermal Enthalpies = –3796.425416

Sum of electronic and thermal Free Energies = –3796.627557

PCM(toluene)-B3LYP-D3/6-311++G**//B3LYP/6-31G*@–30 °C

E = –3799.045637

G = –3797.792354

0 1

O 0.70274000 -0.65420300 -0.31882800

C 1.90968600 -0.79958000 -0.14396400

C 2.70673100 -1.77666700 0.60066300

O 2.80252800 0.17191100 -0.57800300

N 4.06799800 -1.42899000 0.38315700

C 4.06984700 -0.31515600 -0.24982000

C 5.21703400 0.48005800 -0.71929900

C 5.37305200 1.82224700 -0.31862600

C 6.17025800 -0.08528900 -1.59293800

C 6.45418600 2.58672500 -0.77495500

C 7.25916400 0.67468100 -2.04362400

C 7.38380700 1.99776400 -1.62939800

O 4.42302800 2.29423600 0.53828000

C 4.52527400 3.63844400 0.99159600

H 5.46369100 3.80511500 1.53581500

H 3.68040300 3.78914000 1.66333100

H 4.45652700 4.34687800 0.15658200

O 5.94169700 -1.36814100 -1.97118000

C 6.82162800 -1.97479100 -2.90409500

H 7.83664500 -2.07157600 -2.49728400

H 6.86070200 -1.41330500 -3.84677700

H 6.40657500 -2.96613000 -3.09041500

H 6.58017300 3.61648300 -0.46554100

H 7.99631500 0.24639700 -2.71116900

H 8.22753600 2.58595200 -1.98065900

C 2.36296700 -3.25343000 0.58428100

H 1.31047000 -3.35615000 0.86462300

H 2.95689300 -3.73448800 1.37112300

C 2.62014200 -3.96078900 -0.73527400

C 1.55654900 -4.34698300 -1.56136400

C 3.92713700 -4.27488800 -1.13836900

C 1.78741600 -5.03073000 -2.75802100

C 4.15893800 -4.96340300 -2.32905300

C 3.09028400 -5.34374200 -3.14542400

H 0.53626400 -4.11980600 -1.26134300

H 4.76009500 -3.96792800 -0.51276800

H 0.94597700 -5.32813300 -3.37932500

H 5.17736800 -5.21765900 -2.61520300

H 3.27236000 -5.88519300 -4.07040600

C 2.21411100 -1.17722400 2.46783100

C 1.04480300 -1.87313000 2.93842300

H 2.03452500 -0.13624700 2.20160800

H 1.19735200 -2.75904800 3.55190200

C -0.26154900 -1.50716100 2.66751600

H -0.43000400 -0.63982300 2.03348000

C -1.41237200 -2.15395400 3.14818900

H -1.28825800 -3.00171400 3.81332600

C -2.71025100 -1.69319000 2.86165200

O -3.02191500 -0.69386800 2.15428800

N -3.81108900 -2.40728700 3.44978600

C -3.81901500 -3.67239600 4.01927800

C -5.08985100 -1.88441600 3.53416900

C -5.09109800 -3.94335600 4.45876200

C -5.89840500 -2.80311500 4.15652600

H -2.93322400 -4.28688200 4.02393800

H -5.28308300 -0.89223100 3.16060000

H -5.41167800 -4.86079800 4.93523600

H -6.95032300 -2.67511900 4.37628200

H -0.11157000 1.09931100 -0.21592300

N -0.88973000 1.76703800 -0.15832700

P -2.26103100 1.41313000 -0.99749600

N -2.94709800 2.90124700 -0.72164100

C -2.17265600 3.75740900 0.20501100

C -0.68242100 3.21404500 0.06661500

C -4.29467300 3.27259200 -1.14007800

H -4.59726200 2.65792800 -1.99033000

H -5.03359100 3.12911000 -0.34520300

H -4.30615300 4.32495200 -1.44704900

H -2.18920600 4.77029800 -0.20729900

C -2.76415600 3.87890400 1.64670200

C -3.27838700 2.54896500 2.22962400

C -3.57952000 2.59701900 3.73227500

H -2.54860300 1.75327400 2.06367600

C 0.13429200 3.46987600 1.34105900

C 0.32916200 2.47901800 2.30910100

C 0.65703100 4.75094200 1.57841200

C 1.02738200 2.75803200 3.48736600

C 1.33776500 5.03600300 2.76159100

C 1.52916200 4.03808900 3.72165200

H -0.08068200 1.48810200 2.15699600

H 0.52984600 5.53159300 0.83353900

H 1.16871000 1.96882400 4.22043500

H 1.72298400 6.03835700 2.93086700

H 2.06327000 4.25791000 4.64207800

C 0.08123800 3.80771900 -1.15126700

C 1.34278200 3.25781500 -1.44417000

C -0.39373300 4.83138300 -1.97943500

C 2.09227200 3.70868200 -2.52765000

C 0.36040700 5.28902400 -3.06694800

C 1.60401000 4.73030800 -3.34725900

H 1.75577700 2.46940800 -0.82206900

H -1.35828400 5.29435100 -1.80125300

H 3.06035900 3.25753800 -2.72762400

H -0.03529800 6.08608600 -3.69126500

H 2.18818800 5.08497300 -4.19212100

N -2.76895200 -0.06657300 -0.53296700

C -3.47587500 -0.81706500 -1.59866200

C -2.67023400 -0.35041700 -2.89056400

N -2.41894800 1.08811500 -2.61407300

H -2.81445600 -0.35342500 0.46432800

C -2.41199400 2.06421900 -3.69665500

H -1.56031900 1.92449600 -4.36740900

H -2.34886800 3.07026700 -3.27908800

H -3.33798300 1.98284400 -4.28115800

H -3.32535100 -0.38111700 -3.76522300

C -1.39475100 -1.18958400 -3.23037300

H -0.99261400 -1.59000000 -2.29398700

C -0.25951600 -0.35779800 -3.86775900

H -0.04003400 0.50164700 -3.22572700

C 1.04260400 -1.14185000 -4.07384800

C -4.96097200 -0.36542100 -1.62658600

C -5.69026200 -0.10308700 -2.79286400

C -5.62606800 -0.25202900 -0.39281000

C -7.03351500 0.28614000 -2.73116100

C -6.96453600 0.12896600 -0.33069400

C -7.67549600 0.40733000 -1.50132600

H -5.23311300 -0.20491700 -3.77188600

H -5.08861700 -0.47226100 0.52552900

H -7.57344300 0.48845700 -3.65272400

H -7.45482500 0.20167000 0.63650300

H -8.71904300 0.70670000 -1.45352400

C -3.40771400 -2.32460000 -1.29913300

C -4.38023600 -3.19455300 -1.81585700

C -2.38707500 -2.86257900 -0.50313300

C -4.32533100 -4.56427200 -1.55953400

C -2.34330200 -4.23254100 -0.23402900

C -3.30653400 -5.08998600 -0.76411700

H -5.19539900 -2.80144100 -2.41503300

H -1.62491700 -2.21783700 -0.08013400

H -5.09094700 -5.21683100 -1.97089200

H -1.55575500 -4.62082500 0.40567800

H -3.27229000 -6.15457000 -0.54911600

H 1.83877000 -0.47112700 -4.41646100

H 1.37632100 -1.60496900 -3.13971400

H 0.93875000 -1.93505900 -4.82150200

H -3.85438300 1.59805100 4.08512300

H -2.69764800 2.91927100 4.30008900

H -4.40249900 3.27892700 3.97562400

C 3.48590400 -1.32390100 3.23633600

C 3.85575000 -2.52147500 3.87197300

C 4.35351900 -0.22484900 3.34970200

C 5.04355400 -2.61316900 4.59553500

H 3.21255700 -3.39349000 3.80353700

C 5.53881700 -0.31294200 4.07766800

H 4.09647400 0.70562500 2.85050700

C 5.89025800 -1.50852200 4.70581000

H 5.30675900 -3.55135600 5.07770200

H 6.19073900 0.55405300 4.15182500

H 6.81411400 -1.58022900 5.27395500

C -3.82618300 4.99580500 1.71454600

H -3.45339700 5.92960600 1.27463700

H -4.75363100 4.72842700 1.19648100

H -4.08813900 5.21226700 2.75481200

H -1.93613100 4.21815600 2.27808800

H -4.18508400 2.23749200 1.69352500

C -1.78380500 -2.36213800 -4.14851900

H -2.61938500 -2.94090900 -3.74934000

H -2.07158700 -1.99127300 -5.14258900

H -0.94467500 -3.05106100 -4.28173800

H -0.59076500 0.04148600 -4.83793400

- **1ba**-*SS* (HH)


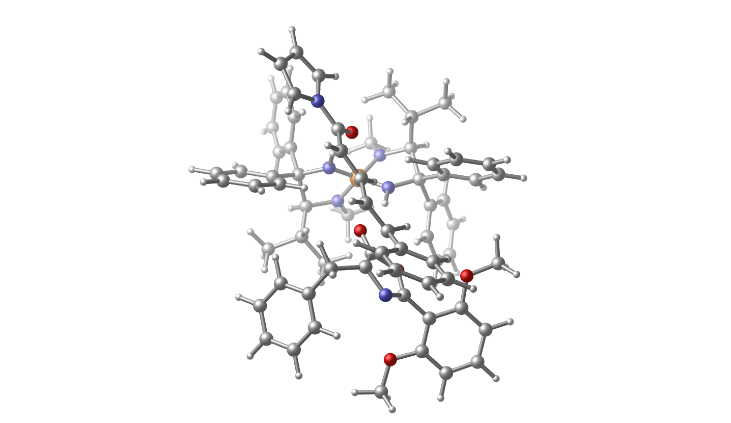

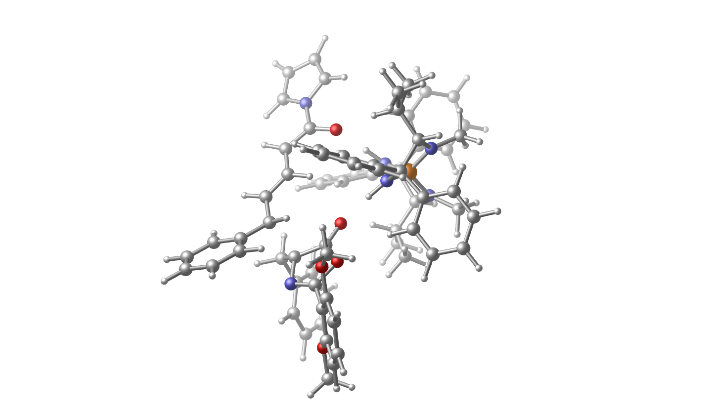


B3LYP/6-31G*

E = –3719.221269

Zero-point correction = 1.284239 (Hartree/Particle)

Thermal correction to Energy = 1.359952

Thermal correction to Enthalpy = 1.360896

Thermal correction to Gibbs Free Energy = 1.164995

Sum of electronic and zero-point Energies = –3717.937030

Sum of electronic and thermal Energies = –3717.861317

Sum of electronic and thermal Enthalpies = –3717.860373

Sum of electronic and thermal Free Energies = –3718.056274

PCM(toluene)-B3LYP-D3/6-311++G**//B3LYP/6-31G*@–30 °C

E = –3720.386288

G = –3719.187561

0 1

O 0.68837200 -0.59212600 -0.18805900

C 1.90837500 -0.71726500 -0.13773000

C 2.79378300 -1.73932600 0.42231900

O 2.73833100 0.31646200 -0.56032600

N 4.11831200 -1.34250200 0.09554800

C 4.03760800 -0.16998800 -0.41585400

C 5.12529900 0.70235800 -0.89218200

C 5.33617600 1.96322300 -0.30041200

C 5.96714800 0.29408500 -1.94688900

C 6.36399200 2.80294600 -0.74744400

C 7.00296900 1.12793200 -2.39153700

C 7.18432600 2.36882000 -1.78637300

O 4.49510900 2.27477400 0.72538200

C 4.60814600 3.55538100 1.33156500

H 5.58654800 3.68635600 1.81174600

H 3.82051600 3.59650800 2.08409600

H 4.45136600 4.35695900 0.59883800

O 5.68681500 -0.91687500 -2.49559700

C 6.46099800 -1.36281200 -3.59750500

H 7.51344000 -1.50528700 -3.31999300

H 6.39997600 -0.66489800 -4.44288600

H 6.02839300 -2.32117100 -3.88832800

H 6.53386700 3.77026600 -0.29150200

H 7.65648800 0.82126300 -3.19860100

H 7.98727100 3.01439400 -2.13247000

C 2.46047200 -3.21380800 0.32924800

H 1.45145600 -3.35582100 0.72904600

H 3.15166600 -3.74537800 0.99550900

C 2.55279200 -3.82282900 -1.06118100

C 1.41511300 -4.34958100 -1.68580600

C 3.78480900 -3.91848900 -1.72753900

C 1.50071900 -4.96158500 -2.93969000

C 3.87121000 -4.52851200 -2.97901100

C 2.73051900 -5.05406800 -3.59151600

H 0.45254900 -4.29112500 -1.18291300

H 4.67114400 -3.50117500 -1.25949300

H 0.60613700 -5.37602400 -3.39820000

H 4.83707900 -4.60806000 -3.47351200

H 2.80168900 -5.53647500 -4.56319700

C 2.54326200 -1.30016300 2.39276600

C 1.35627400 -1.92398500 2.91496500

H 2.45147900 -0.22652700 2.23081700

H 1.46571400 -2.86375300 3.45240200

C 0.08452700 -1.40337500 2.76957200

H -0.01426700 -0.48323800 2.20325500

C -1.11358800 -1.93354800 3.27951300

H -1.07749600 -2.82207200 3.90087600

C -2.34649600 -1.30507100 3.03077400

O -2.52975400 -0.25748300 2.34555500

N -3.53092000 -1.89344600 3.59730200

C -3.69892500 -3.15152800 4.15537000

C -4.74189300 -1.22469300 3.65779100

C -5.00389600 -3.27528300 4.56451600

C -5.66642600 -2.04766600 4.25329900

H -2.89000900 -3.86392700 4.17845000

H -4.80404500 -0.21078600 3.29725900

H -5.43941800 -4.15137800 5.02739500

H -6.70063000 -1.79899500 4.45305100

H -0.25054400 1.10976500 0.00194800

N -1.03979900 1.75660500 -0.09057300

P -2.36398600 1.29880200 -0.93968500

N -3.13845600 2.74675800 -0.71680700

C -2.50260200 3.57471200 0.33841800

C -0.95665100 3.20817400 0.18604600

C -4.32753800 3.21047900 -1.42167300

H -4.61587100 2.47485200 -2.17439200

H -5.17647300 3.34490300 -0.74751500

H -4.12107100 4.16517200 -1.92362300

H -2.66562600 4.61808500 0.05655300

C -3.10626700 3.37166700 1.76554000

C -4.60290300 3.01747600 1.76917000

H -4.82081300 2.11884100 1.18610500

C -0.12355100 3.43887400 1.45869200

C -0.07491900 2.46636000 2.46830900

C 0.58230600 4.63603600 1.64824200

C 0.65556300 2.68809800 3.63721500

C 1.29865900 4.86369100 2.82447200

C 1.34137700 3.88901000 3.82375300

H -0.62978200 1.54084400 2.35548000

H 0.57328200 5.39869900 0.87641300

H 0.67980300 1.91596300 4.40102600

H 1.82679200 5.80498200 2.95546700

H 1.90243500 4.06446000 4.73794300

C -0.29402700 3.92532800 -1.02041800

C 0.85560400 3.34318900 -1.58346600

C -0.74382900 5.13718300 -1.56051200

C 1.51389100 3.94009100 -2.65674000

C -0.08740100 5.73529300 -2.64209800

C 1.04155700 5.13856600 -3.19761800

H 1.25293800 2.41652500 -1.18032700

H -1.60869000 5.64533700 -1.14672700

H 2.40064100 3.46448600 -3.06695100

H -0.46530500 6.67227300 -3.04323300

H 1.55176700 5.60246500 -4.03748800

N -2.83486300 -0.17151000 -0.41325300

C -3.49497800 -0.99233300 -1.45126200

C -2.66134700 -0.57075900 -2.74269000

N -2.45684800 0.88943100 -2.53975900

H -2.83453100 -0.37929200 0.60227000

C -2.35228900 1.80228700 -3.67162800

H -1.47858600 1.58406700 -4.29010800

H -2.25547700 2.82697900 -3.30680300

H -3.25226300 1.73655900 -4.29757900

H -3.28101100 -0.67292900 -3.63787300

C -1.35222400 -1.38991900 -2.97382700

H -0.98923700 -1.72595900 -1.99813400

C -0.19781900 -0.59347000 -3.60285900

H 0.06631100 0.29147100 -3.01802900

C -4.99024800 -0.58283400 -1.54461000

C -5.68258100 -0.36852000 -2.74250700

C -5.70557100 -0.45720000 -0.34092400

C -7.03742300 -0.01586300 -2.74045000

C -7.05484000 -0.11205700 -0.33628300

C -7.72860800 0.11750800 -1.53907700

H -5.18622500 -0.48018200 -3.70083300

H -5.20280100 -0.64170500 0.60421800

H -7.54712400 0.14871400 -3.68639300

H -7.58125300 -0.02890600 0.61070600

H -8.78075700 0.38908100 -1.53724400

C -3.40077100 -2.48091300 -1.07574900

C -4.32182900 -3.40252200 -1.59742500

C -2.40919800 -2.94984600 -0.20315300

C -4.24465900 -4.75577300 -1.27037400

C -2.34281400 -4.30367100 0.13535000

C -3.25456700 -5.21296800 -0.39923100

H -5.11361600 -3.06191500 -2.25711400

H -1.68353700 -2.26575500 0.22235400

H -4.96985100 -5.44962000 -1.68740400

H -1.57789600 -4.63635900 0.83150800

H -3.20307900 -6.26479000 -0.13121200

C 3.86023600 -1.64745000 3.00238000

C 4.14969900 -2.92785300 3.50520600

C 4.85585000 -0.66112700 3.10479800

C 5.38369200 -3.20794100 4.09047200

H 3.40553600 -3.71595500 3.44365100

C 6.08757600 -0.93778000 3.69461500

H 4.65867400 0.33125000 2.70866800

C 6.35860900 -2.21390500 4.19102300

H 5.58204400 -4.20637300 4.47226800

H 6.83889700 -0.15495200 3.76382900

H 7.31893700 -2.43221500 4.65103500

C -2.89852200 4.62898700 2.62850900

H -1.85134900 4.92745100 2.70548900

H -3.46331300 5.47583100 2.21380400

H -3.26927500 4.45426000 3.64465400

H -2.59698300 2.52476800 2.23471600

H -5.22388800 3.84138800 1.39452400

C -1.64150300 -2.62136900 -3.84963300

H -2.42508300 -3.25996400 -3.43511500

H -1.95229900 -2.31262200 -4.85787000

H -0.73558800 -3.22740600 -3.95396600

H -0.41906200 -0.28781600 -4.63371700

H -4.91644900 2.82313100 2.80095400

H 0.68996900 -1.23358700 -3.63934900

- **1ba**-*SS* (MeH)


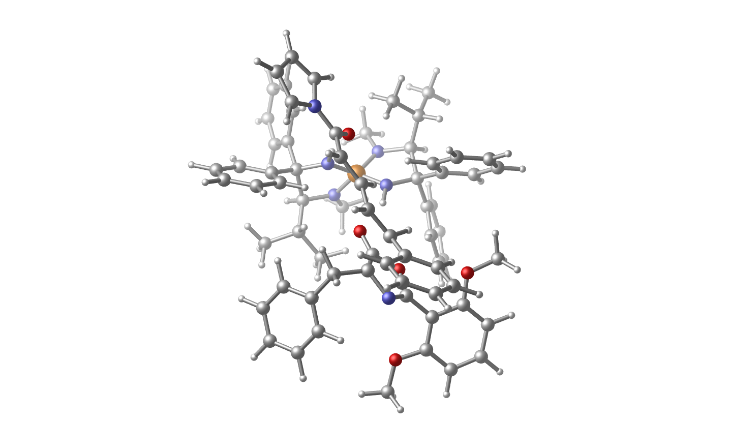

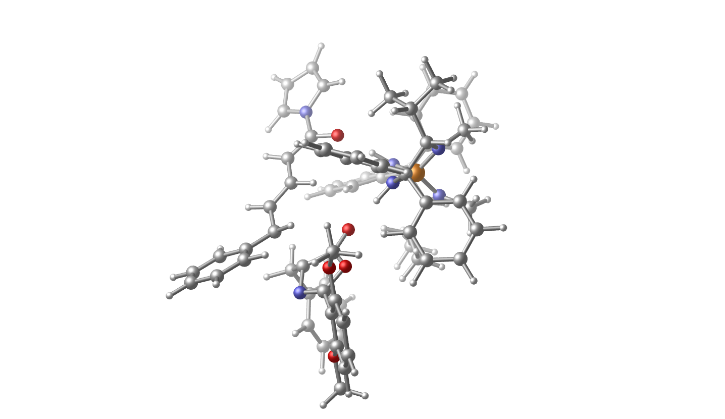


B3LYP/6-31G*

E = –3719.222543

Zero-point correction = 1.284083 (Hartree/Particle)

Thermal correction to Energy = 1.359878

Thermal correction to Enthalpy = 1.360822

Thermal correction to Gibbs Free Energy = 1.164880

Sum of electronic and zero-point Energies = –3717.938460

Sum of electronic and thermal Energies = –3717.862665

Sum of electronic and thermal Enthalpies = –3717.861721

Sum of electronic and thermal Free Energies = –3718.057663

PCM(toluene)-B3LYP-D3/6-311++G**//B3LYP/6-31G*@–30 °C

E = –3720.3882

G = –3719.189581

0 1

O 0.68817800 -0.62159000 -0.22449300

C 1.90815500 -0.74520200 -0.17569400

C 2.79768000 -1.80177100 0.31141400

O 2.73685900 0.31918300 -0.51514900

N 4.12127400 -1.37995000 0.01337000

C 4.03728600 -0.17097200 -0.40404700

C 5.11797200 0.74698700 -0.80235000

C 5.28039400 1.98329100 -0.14474100

C 5.99934500 0.41186900 -1.85129300

C 6.29512000 2.87069500 -0.52513200

C 7.02305600 1.29310300 -2.22756500

C 7.15369700 2.50858800 -1.56109400

O 4.40587300 2.22416200 0.87358400

C 4.48214600 3.46898400 1.55778200

H 5.45176500 3.59196800 2.05711100

H 3.68577800 3.44663700 2.30197000

H 4.31371000 4.30926100 0.87292500

O 5.76676800 -0.77754400 -2.46415400

C 6.57457000 -1.14591200 -3.57041600

H 7.62492100 -1.27407700 -3.27850800

H 6.51009200 -0.40807200 -4.38085200

H 6.17492500 -2.09964700 -3.91818800

H 6.42574500 3.81926100 -0.01978800

H 7.70509500 1.04240400 -3.03046600

H 7.94650500 3.19144500 -1.85490000

C 2.46311500 -3.26516900 0.11382000

H 1.46231300 -3.43823900 0.52179200

H 3.16824900 -3.84506900 0.72230600

C 2.52494500 -3.75971500 -1.32253900

C 1.36956700 -4.21456400 -1.97082900

C 3.74462100 -3.81244100 -2.01556100

C 1.42659600 -4.71441400 -3.27501400

C 3.80225000 -4.30938600 -3.31763400

C 2.64421400 -4.76339500 -3.95408900

H 0.41654800 -4.18784200 -1.44712600

H 4.64447700 -3.45071400 -1.52719000

H 0.51946300 -5.07631800 -3.75300200

H 4.75825300 -4.35498100 -3.83507400

H 2.69241500 -5.15746100 -4.96616700

C 2.56387700 -1.49516900 2.30460400

C 1.38595200 -2.16038400 2.79464900

H 2.46541800 -0.41345800 2.21603300

H 1.50854900 -3.12963100 3.27399300

C 0.10838400 -1.64638700 2.68373600

H -0.00563000 -0.69456200 2.17387000

C -1.07871500 -2.22607500 3.16388900

H -1.02542700 -3.14731500 3.73426100

C -2.32185000 -1.60496200 2.95510300

O -2.51976500 -0.51986300 2.33664300

N -3.49325400 -2.24585400 3.48892700

C -3.64087600 -3.54858000 3.94048300

C -4.70729100 -1.59586700 3.62930200

C -4.93652800 -3.71845200 4.36245500

C -5.61366100 -2.47449700 4.17048800

H -2.82646500 -4.25293900 3.88482600

H -4.78564800 -0.55539100 3.35880500

H -5.35679100 -4.63502100 4.75589600

H -6.64544600 -2.25286000 4.41047100

H -0.24747600 1.09685400 -0.02189100

N -1.02751200 1.76434300 -0.00302000

P -2.38402000 1.41407400 -0.85422500

N -3.06448900 2.91039800 -0.60958900

C -2.32435700 3.75748900 0.35178200

C -0.83201300 3.20149400 0.28519100

C -4.38465600 3.30275000 -1.08946800

H -4.67593000 2.66464600 -1.92615700

H -5.15475500 3.21109000 -0.31684200

H -4.35623500 4.34299700 -1.43572400

H -2.30737300 4.76971100 -0.06110100

C -2.99267600 3.89665200 1.75823900

C -3.64872800 2.61787500 2.29469100

H -2.96254200 1.77083700 2.35986900

C -0.09169200 3.37985700 1.61895600

C -0.07412100 2.36290900 2.58242700

C 0.53481000 4.59973500 1.91492000

C 0.56743000 2.55768200 3.80797300

C 1.15889300 4.79969200 3.14658900

C 1.18388200 3.77558300 4.09692400

H -0.58716500 1.42635100 2.39164900

H 0.53860000 5.39729100 1.17749000

H 0.57462300 1.75164600 4.53635500

H 1.62968000 5.75599900 3.36066100

H 1.67490100 3.92842400 5.05439800

C -0.00035000 3.83023300 -0.86812000

C 1.29523200 3.32213900 -1.07192000

C -0.45247200 4.83121000 -1.73633600

C 2.10282400 3.79326400 -2.10368700

C 0.35944900 5.31033500 -2.77162400

C 1.63860300 4.79509100 -2.96053200

H 1.68664100 2.54614700 -0.42204900

H -1.44324200 5.25974500 -1.63193800

H 3.09559500 3.37182600 -2.23507400

H -0.01942900 6.09017200 -3.42743400

H 2.26768800 5.16684000 -3.76489800

N -2.92458600 -0.04848900 -0.37215900

C -3.60756900 -0.82026100 -1.43580700

C -2.77825300 -0.37623300 -2.72149000

N -2.52638600 1.06550000 -2.46627700

H -2.87022300 -0.34444400 0.62104800

C -2.45324800 2.01629700 -3.56820000

H -1.60887400 1.80586800 -4.22917300

H -2.32498200 3.02444100 -3.17027600

H -3.37905600 1.98497600 -4.15834200

H -3.41496600 -0.42359600 -3.60917500

C -1.49677600 -1.22033400 -3.01047500

H -1.12351500 -1.60589100 -2.05726000

C -0.33365400 -0.42868500 -3.63030200

H -0.02976700 0.42146700 -3.01386400

C -5.09219600 -0.37047300 -1.49733100

C -5.79744900 -0.11308600 -2.67916500

C -5.78349300 -0.24810200 -0.27941500

C -7.14076600 0.27969300 -2.64696200

C -7.12178200 0.13577000 -0.24494100

C -7.80773500 0.40962100 -1.43152400

H -5.32059100 -0.22074700 -3.64788900

H -5.26739500 -0.46520500 0.65127900

H -7.66101400 0.47829400 -3.58054100

H -7.63088000 0.21507600 0.71188300

H -8.85110500 0.71220700 -1.40672400

C -3.54022700 -2.32357800 -1.11909700

C -4.49987700 -3.20187900 -1.64563000

C -2.52612200 -2.85220500 -0.30852800

C -4.43824700 -4.57038900 -1.38437800

C -2.47448000 -4.22112000 -0.03603800

C -3.42466300 -5.08688700 -0.57602500

H -5.30870300 -2.81663900 -2.25831400

H -1.77233400 -2.20385800 0.12348000

H -5.19349700 -5.22981600 -1.80390500

H -1.69065300 -4.59914300 0.61421300

H -3.38457200 -6.15099200 -0.35953400

C 3.88636500 -1.87460300 2.88269800

C 4.19487400 -3.19066100 3.26827300

C 4.86681900 -0.88681400 3.07480300

C 5.43291400 -3.50400100 3.82718900

H 3.46246900 -3.98070700 3.13306300

C 6.10247400 -1.19685100 3.63960700

H 4.65453200 0.13422600 2.76935900

C 6.39259300 -2.50839100 4.01896900

H 5.64667000 -4.52983500 4.11693200

H 6.84180500 -0.41224200 3.78063200

H 7.35608500 -2.75291800 4.45871100

C -3.98534000 5.07665700 1.78173200

H -3.51061100 6.00969800 1.45259700

H -4.86185600 4.90538600 1.14719800

H -4.35129000 5.23636500 2.80224600

H -2.18843800 4.17016300 2.44763500

H -4.50046300 2.30889700 1.67672700

C -1.83673000 -2.40712600 -3.92852200

H -2.62434200 -3.04515400 -3.52082500

H -2.16537600 -2.04849000 -4.91427600

H -0.94783700 -3.02746000 -4.08267500

H -0.56845100 -0.07075700 -4.64108100

H 0.53392400 -1.09149800 -3.71548700

H -4.03446600 2.80351400 3.30413300

- **1ba**-*SS* (MeMe)


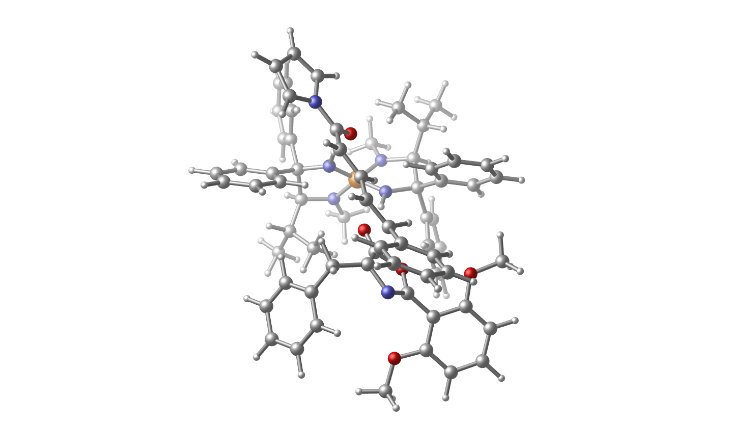

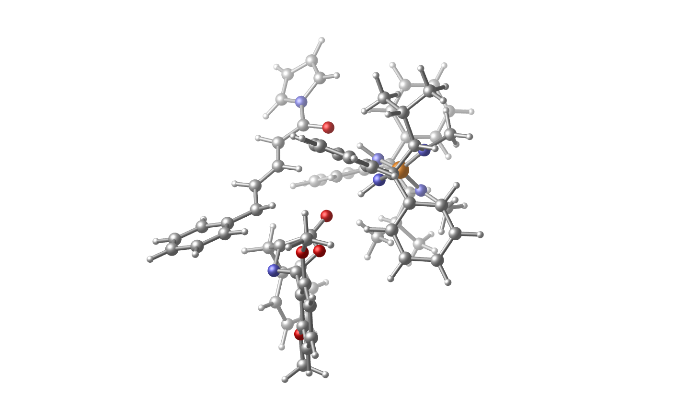


B3LYP/6-31G*

E = –3719.221138

Zero-point correction = 1.283904 (Hartree/Particle)

Thermal correction to Energy = 1.359739

Thermal correction to Enthalpy = 1.360683

Thermal correction to Gibbs Free Energy = 1.164759

Sum of electronic and zero-point Energies = –3717.937234

Sum of electronic and thermal Energies = –3717.861399

Sum of electronic and thermal Enthalpies = –3717.860455

Sum of electronic and thermal Free Energies = –3718.056378

PCM(toluene)-B3LYP-D3/6-311++G**//B3LYP/6-31G*@–30 °C

E = –3720.385301

G = –3719.186807

0 1

O 0.80040000 -0.49400500 -0.15767000

C 2.01798800 -0.64791600 -0.14668000

C 2.88434400 -1.77417300 0.20371400

O 2.86865800 0.42571500 -0.38537700

N 4.21359300 -1.35532800 -0.06368600

C 4.15721500 -0.10747400 -0.35117500

C 5.25946200 0.81139300 -0.68387200

C 5.49952000 1.95351200 0.10517400

C 6.08776400 0.56527200 -1.79840400

C 6.54267000 2.83471600 -0.20630500

C 7.13754900 1.44106000 -2.10974400

C 7.34812500 2.56146300 -1.30992300

O 4.66921700 2.10803300 1.17454700

C 4.81237500 3.26553000 1.98663600

H 5.79243300 3.28985800 2.48066300

H 4.02275700 3.19875800 2.73534300

H 4.67715000 4.18282500 1.40003200

O 5.77887800 -0.53369700 -2.53367800

C 6.53148500 -0.80873700 -3.70397700

H 7.58425000 -1.01213300 -3.46855300

H 6.47426800 0.01762300 -4.42473700

H 6.07645400 -1.69989600 -4.13858000

H 6.73480800 3.70929700 0.40241700

H 7.77908900 1.25823500 -2.96280500

H 8.16240700 3.23941800 -1.55195200

C 2.51167700 -3.19907300 -0.15039300

H 1.49873200 -3.38254700 0.22084100

H 3.18751700 -3.86125900 0.40476400

C 2.59107700 -3.54011600 -1.62959300

C 1.43188500 -3.81827400 -2.36466700

C 3.83095500 -3.61944700 -2.28269000

C 1.50335800 -4.16639900 -3.71637000

C 3.90438100 -3.97049300 -3.63060300

C 2.74112700 -4.24483600 -4.35473300

H 0.46274200 -3.76761000 -1.87394500

H 4.73544900 -3.39271700 -1.72634900

H 0.59048200 -4.38599900 -4.26507700

H 4.87537300 -4.04402300 -4.11596200

H 2.80070900 -4.52387400 -5.40384900

C 2.66523400 -1.69717700 2.23676400

C 1.47805600 -2.39801400 2.64633700

H 2.58331400 -0.61137600 2.26833400

H 1.58579100 -3.41453900 3.01957300

C 0.20668800 -1.86304400 2.57076600

H 0.11096100 -0.86402600 2.15894500

C -0.99462800 -2.47542700 2.96892800

H -0.96368100 -3.44738900 3.45029400

C -2.22780600 -1.82475000 2.80062800

O -2.41381200 -0.68332200 2.28962600

N -3.41098800 -2.50978400 3.25539000

C -3.59199800 -3.86381000 3.48489000

C -4.59523700 -1.85457700 3.54492300

C -4.88110500 -4.06362500 3.91614200

C -5.51746300 -2.78521300 3.96190800

H -2.80536700 -4.57082000 3.27424300

H -4.64197600 -0.78076100 3.45945400

H -5.32391000 -5.02059600 4.15994500

H -6.53096300 -2.57528300 4.27865100

H -0.32172800 1.06651100 -0.08414300

N -1.08278500 1.75695900 -0.04468400

P -2.51795000 1.48264700 -0.78272100

N -3.14238100 2.96712200 -0.36916700

C -2.31412100 3.72948600 0.58814900

C -0.84301600 3.15637900 0.37054600

C -4.47226600 3.43582600 -0.73906200

H -4.82893200 2.87295100 -1.60371100

H -5.20112100 3.30918700 0.06844600

H -4.42687800 4.49875900 -1.00575500

H -2.30500200 4.76735100 0.24535500

C -2.87916000 3.77933100 2.04546000

C -3.53304200 2.47903500 2.53264300

H -2.86784700 1.61361400 2.49242800

C -0.02192300 3.19717800 1.66788400

C 0.01481700 2.10201500 2.54034800

C 0.64682400 4.37465800 2.03409000

C 0.71068600 2.18173900 3.74890700

C 1.32651000 4.45897300 3.24942500

C 1.36521000 3.35964600 4.11095100

H -0.52637200 1.19415400 2.29650000

H 0.63538000 5.23182500 1.36709600

H 0.72823400 1.31825000 4.40806400

H 1.82756600 5.38488300 3.52075300

H 1.89500000 3.42360800 5.05792900

C -0.06722300 3.87339300 -0.76957600

C 1.19967200 3.35791600 -1.09837300

C -0.54357900 4.95973800 -1.51305300

C 1.95576700 3.90512800 -2.13094100

C 0.21662800 5.51331400 -2.55081100

C 1.46750300 4.98982300 -2.86498800

H 1.60768700 2.51861300 -0.54380900

H -1.51304100 5.40102300 -1.30671000

H 2.92943600 3.47934700 -2.35764100

H -0.17958900 6.35809700 -3.10860700

H 2.05718900 5.42023600 -3.66997600

N -3.00896800 -0.01516200 -0.36232900

C -3.82457900 -0.70812000 -1.38034400

C -3.28466800 -0.09894600 -2.75043100

N -2.87316800 1.27836300 -2.39253500

H -2.91861100 -0.35001500 0.61587800

C -2.65270400 2.28522100 -3.42268200

H -1.73511000 2.10360600 -3.99218500

H -2.56888300 3.26936400 -2.95694400

H -3.50012800 2.29600400 -4.11887400

H -4.13748900 -0.00575200 -3.42785000

C -2.21171400 -0.91789300 -3.53406800

H -2.46929400 -1.97056000 -3.38137300

C -0.77553900 -0.71250100 -3.04162100

H -0.65721500 -0.94297500 -1.98147300

C -5.32132800 -0.38110100 -1.11067800

C -6.19909400 0.21974500 -2.01986400

C -5.82087400 -0.71417600 0.16119200

C -7.52940900 0.48592400 -1.67103700

C -7.14233700 -0.45338600 0.50943400

C -8.00715600 0.15154300 -0.40750100

H -5.87419300 0.49420300 -3.01772000

H -5.17094600 -1.18946200 0.89013100

H -8.18736500 0.95335900 -2.39931300

H -7.49469800 -0.73279500 1.49860100

H -9.04052000 0.35403500 -0.13898400

C -3.62064100 -2.22798500 -1.29314700

C -4.52598100 -3.08304800 -1.94091000

C -2.54068700 -2.79219200 -0.60361900

C -4.34883500 -4.46514000 -1.91224200

C -2.37551800 -4.17947200 -0.56065500

C -3.27201500 -5.02059700 -1.21786800

H -5.38310600 -2.66589900 -2.46224500

H -1.82368800 -2.16216700 -0.09004700

H -5.06168400 -5.10822600 -2.42171300

H -1.54264800 -4.59389300 0.00048800

H -3.14052600 -6.09865800 -1.18248900

C 3.98549900 -2.16286100 2.74897600

C 4.26797800 -3.51815200 2.99351000

C 4.99423100 -1.22252300 3.01910500

C 5.50752900 -3.91517300 3.49252700

H 3.51370300 -4.27315800 2.79430800

C 6.23170000 -1.61701700 3.52368700

H 4.80304400 -0.17134300 2.82110100

C 6.49524600 -2.96661900 3.76364700

H 5.70054800 -4.96974500 3.67307000

H 6.99341300 -0.86836900 3.72714400

H 7.46013000 -3.27667400 4.15654900

C -3.83653200 4.97472800 2.22629600

H -3.36468200 5.91894100 1.92618500

H -4.76343600 4.86591800 1.65254600

H -4.11917200 5.06954200 3.28082100

H -2.02386100 3.98370600 2.69574100

H -4.43248300 2.23408200 1.95489000

C -2.31859600 -0.67605100 -5.05292500

H -1.64456000 -1.35623500 -5.58569200

H -3.33499100 -0.86431700 -5.42155500

H -2.04097700 0.34351500 -5.34180300

H -0.43394100 0.31764900 -3.19810800

H -3.84530200 2.59985500 3.57673400

H -0.09245000 -1.36871700 -3.59177900

- **1ba**-*SS* (HMe)


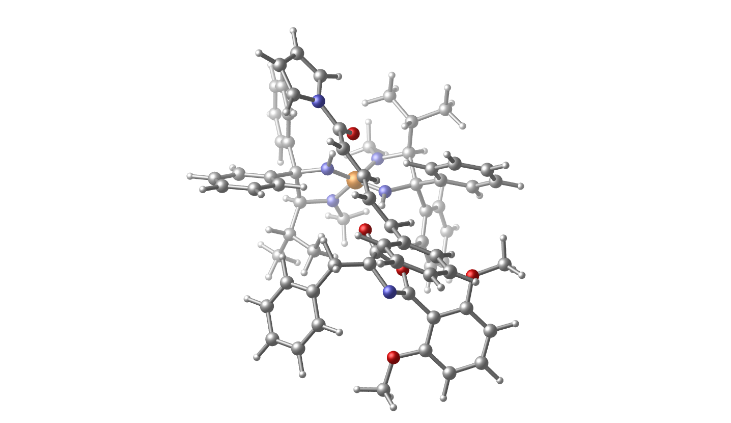

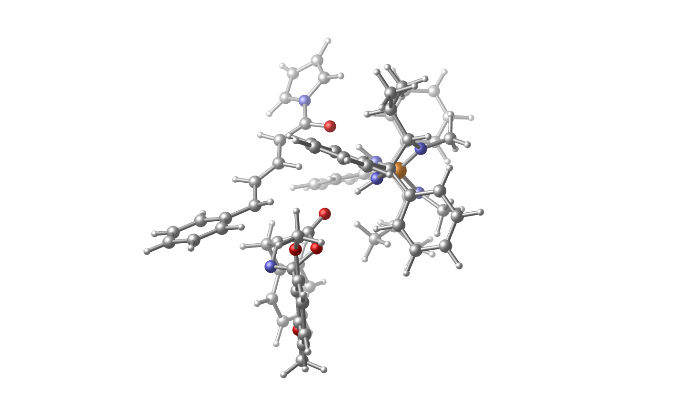


B3LYP/6-31G*

E = –3719.220135

Zero-point correction = 1.284550 (Hartree/Particle)

Thermal correction to Energy = 1.360151

Thermal correction to Enthalpy = 1.361095

Thermal correction to Gibbs Free Energy = 1.166366

Sum of electronic and zero-point Energies = –3717.935585

Sum of electronic and thermal Energies = –3717.859984

Sum of electronic and thermal Enthalpies = –3717.859040

Sum of electronic and thermal Free Energies = –3718.053768

PCM(toluene)-B3LYP-D3/6-311++G**//B3LYP/6-31G*@–30 °C

E = –3720.384062

G = –3719.184179

0 1

O 0.80449700 -0.46969800 -0.12950400

C 2.02266800 -0.62448800 -0.12175500

C 2.88750500 -1.73062400 0.28744000

O 2.87188800 0.42957500 -0.43866700

N 4.21543300 -1.33281200 -0.01515200

C 4.15840300 -0.10643000 -0.38381600

C 5.26472500 0.77762800 -0.79310700

C 5.57529900 1.92898900 -0.04518100

C 6.02703700 0.48285600 -1.94155600

C 6.62712300 2.77091500 -0.42833200

C 7.08413900 1.31957100 -2.32646800

C 7.36720900 2.45005800 -1.56417900

O 4.80259300 2.13451600 1.05816600

C 5.01990200 3.30699200 1.83032700

H 6.02003900 3.30977600 2.28305600

H 4.26020800 3.28831000 2.61191500

H 4.89231600 4.21301600 1.22464900

O 5.64999600 -0.62393400 -2.63298800

C 6.34266900 -0.95511900 -3.82547800

H 7.39970100 -1.17775900 -3.62979900

H 6.27392500 -0.15054100 -4.56958800

H 5.84736100 -1.84789600 -4.20981300

H 6.87575100 3.65301800 0.14824700

H 7.67611500 1.10007700 -3.20632100

H 8.18721300 3.09814400 -1.86234600

C 2.50990300 -3.17356700 0.02408000

H 1.49415900 -3.32849500 0.40075400

H 3.18004000 -3.80116200 0.62447900

C 2.59550300 -3.61064000 -1.42960400

C 1.43901100 -3.93041600 -2.15171200

C 3.83825400 -3.73941100 -2.06925300

C 1.51559400 -4.36753800 -3.47701500

C 3.91712700 -4.18004400 -3.39042000

C 2.75620000 -4.49527400 -4.10168600

H 0.46732900 -3.84276100 -1.67167100

H 4.74099100 -3.48113100 -1.52407600

H 0.60416800 -4.61783600 -4.01488800

H 4.89061400 -4.29117500 -3.86338400

H 2.81976500 -4.84388500 -5.12956100

C 2.67587900 -1.53610700 2.32077800

C 1.47922300 -2.19323600 2.77298300

H 2.61305500 -0.44924200 2.28734900

H 1.57303700 -3.18632100 3.20789300

C 0.21467100 -1.64604900 2.66700100

H 0.13417600 -0.67483500 2.19214600

C -0.99478700 -2.21517500 3.10343600

H -0.97530000 -3.15846800 3.63924100

C -2.22099100 -1.56041800 2.90010900

O -2.40015700 -0.44733200 2.32647600

N -3.41192500 -2.20944800 3.39011300

C -3.60436600 -3.54410200 3.70812900

C -4.59493800 -1.52951200 3.62201100

C -4.89886100 -3.70675600 4.13864800

C -5.52761200 -2.42439800 4.09091000

H -2.82015200 -4.26860100 3.55629000

H -4.63393200 -0.46451700 3.45891200

H -5.34994400 -4.64228300 4.44302700

H -6.54255200 -2.18731000 4.38307200

H -0.31203900 1.07028900 -0.05307000

N -1.08312100 1.74729400 -0.10681100

P -2.49553100 1.38194300 -0.84927200

N -3.20830700 2.82093100 -0.43515100

C -2.46958800 3.53864700 0.63032100

C -0.95096700 3.16148000 0.31746900

C -4.41841000 3.38931200 -1.01592300

H -4.76156800 2.75525100 -1.83476400

H -5.22897100 3.46131500 -0.28660400

H -4.20934400 4.39235000 -1.41086600

H -2.62619000 4.60633800 0.45628800

C -2.97127500 3.21986000 2.07606400

C -4.47209400 2.89190800 2.15650800

H -4.74975500 2.04677700 1.52085300

C -0.02539200 3.25154700 1.54248700

C 0.05388900 2.19340300 2.45944100

C 0.72206600 4.41160700 1.79257700

C 0.84803800 2.29904200 3.60258800

C 1.50570600 4.52128700 2.94236400

C 1.57184200 3.46539400 3.85366000

H -0.52956300 1.29256100 2.30058000

H 0.69079700 5.23906100 1.09136400

H 0.89023600 1.46438500 4.29683000

H 2.06480400 5.43607900 3.12284000

H 2.17918700 3.55174100 4.75109500

C -0.36038500 3.98107600 -0.86016900

C 0.74445100 3.43938100 -1.54104500

C -0.82949500 5.23596100 -1.26791300

C 1.34110000 4.11871600 -2.60059400

C -0.23521100 5.91730300 -2.33664800

C 0.84923500 5.36101900 -3.00994100

H 1.15262900 2.48034900 -1.23581400

H -1.66008600 5.71311400 -0.75843200

H 2.19587700 3.67511300 -3.10400300

H -0.62607700 6.88698100 -2.63423900

H 1.31151900 5.88996900 -3.83904100

N -2.92025900 -0.12903300 -0.40223800

C -3.73463800 -0.84775200 -1.40119900

C -3.19726400 -0.26300700 -2.78405300

N -2.80892800 1.13142500 -2.45901300

H -2.88006400 -0.38831800 0.60132200

C -2.56028900 2.11185600 -3.50897000

H -1.63701200 1.90473000 -4.05965700

H -2.46781800 3.10608000 -3.06602300

H -3.39693800 2.12055300 -4.21822900

H -4.04777000 -0.20103100 -3.46809800

C -2.10807800 -1.08752000 -3.53764900

H -2.35641300 -2.13865700 -3.35984600

C -0.67889300 -0.85617300 -3.03721700

H -0.57086400 -1.05210900 -1.96914100

C -5.23490100 -0.52541800 -1.14213400

C -6.11506100 0.04737400 -2.06718700

C -5.73586000 -0.83310100 0.13561500

C -7.44903700 0.30826100 -1.72859200

C -7.06047700 -0.57564900 0.47464500

C -7.92793700 -0.00119400 -0.45909800

H -5.78879000 0.30327600 -3.06955800

H -5.08614800 -1.28589200 0.87873400

H -8.10885600 0.75298700 -2.46930400

H -7.41068200 -0.83402000 1.47018600

H -8.96394100 0.19813100 -0.19857400

C -3.52468300 -2.36386800 -1.27465200

C -4.41177900 -3.24002900 -1.91948600

C -2.46421700 -2.90415600 -0.53729100

C -4.23551000 -4.62021200 -1.84159500

C -2.30044600 -4.28945300 -0.44528500

C -3.17853800 -5.15198500 -1.09959000

H -5.25511000 -2.84026600 -2.47601400

H -1.76106600 -2.25498900 -0.02757200

H -4.93366300 -5.28017500 -2.34982200

H -1.48295800 -4.68627300 0.15031000

H -3.04796400 -6.22820500 -1.02587000

C 3.99132900 -1.99565700 2.84960000

C 4.24985100 -3.33882000 3.17513900

C 5.02153700 -1.06118400 3.05105400

C 5.48644300 -3.72934300 3.68665900

H 3.47935100 -4.08970700 3.02989700

C 6.25599400 -1.44895900 3.56793200

H 4.85006100 -0.02109000 2.78801900

C 6.49541400 -2.78614200 3.88920800

H 5.66035100 -4.77442600 3.93065500

H 7.03469900 -0.70493500 3.71676700

H 7.45808400 -3.09075500 4.29168500

C -2.67064700 4.38963500 3.02956300

H -1.61294400 4.65824900 3.05628800

H -3.24027100 5.28184000 2.73367000

H -2.97358500 4.13149700 4.05037600

H -2.45134700 2.32526400 2.43061100

H -5.09999300 3.75331400 1.89476300

C -2.20495700 -0.88733700 -5.06325200

H -1.51860100 -1.57340000 -5.57222100

H -3.21599300 -1.09761700 -5.43471400

H -1.93757600 0.12744200 -5.37744400

H -0.34415200 0.17174400 -3.22084300

H -4.71782400 2.61866100 3.18877200

H 0.01430000 -1.52324600 -3.56095800

- **1ba**-*RR* (HH)


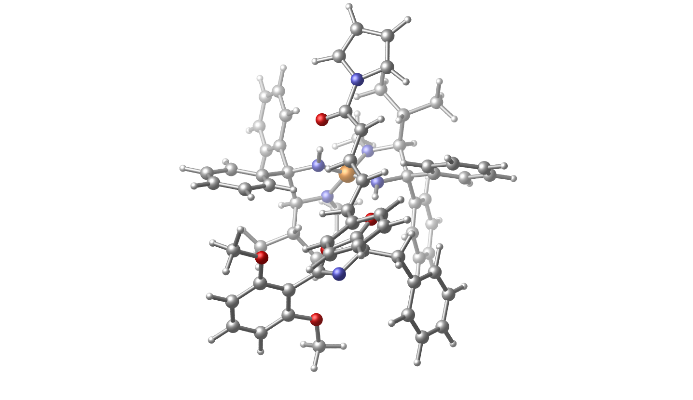

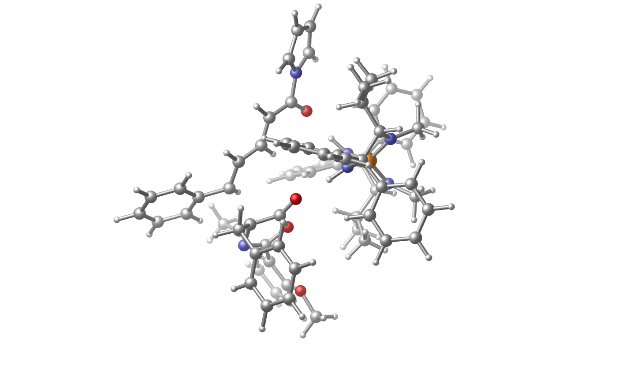


B3LYP/6-31G*

E = –3719.223806

Zero-point correction = 1.284255 (Hartree/Particle)

Thermal correction to Energy = 1.359867

Thermal correction to Enthalpy = 1.360811

Thermal correction to Gibbs Free Energy = 1.165651

Sum of electronic and zero-point Energies = –3717.939550

Sum of electronic and thermal Energies = –3717.863938

Sum of electronic and thermal Enthalpies = –3717.862994

Sum of electronic and thermal Free Energies = –3718.058154

PCM(toluene)-B3LYP-D3/6-311++G**//B3LYP/6-31G*@–30 °C

E = –3720.393004

G = –3719.193756

0 1

O -0.92670200 -1.20608200 -0.08544100

C -2.06259700 -0.75369300 -0.04075700

C -3.28141000 -1.25442300 0.56643100

O -2.37130700 0.51129200 -0.54050900

N -4.32161900 -0.36375300 0.18868900

C -3.74984900 0.63220800 -0.38417300

C -4.37002500 1.85894800 -0.92352600

C -4.80766800 2.89376800 -0.07740800

C -4.54779800 1.99362700 -2.31393700

C -5.42495900 4.03448700 -0.60834800

C -5.16017300 3.13227200 -2.85216700

C -5.59161900 4.13590400 -1.98728900

O -4.58221600 2.71128000 1.25709200

C -5.06561700 3.69246700 2.16598300

H -6.15279400 3.81302600 2.08270500

H -4.57766000 4.66221900 2.00573500

H -4.81740900 3.32049500 3.16119800

O -4.09529900 0.94729900 -3.06001100

C -4.39708800 0.91821900 -4.44753700

H -5.47854200 0.98227600 -4.62144000

H -3.89239400 1.72944600 -4.98799500

H -4.02669100 -0.04268200 -4.80790400

H -5.76500200 4.83596700 0.03509300

H -5.30407000 3.23764700 -3.92045000

H -6.06846700 5.02143800 -2.39881500

C -3.55110000 -2.74529200 0.55955600

H -2.74251600 -3.24031200 1.10975600

H -4.48311400 -2.92799300 1.10334300

C -3.63708100 -3.32432000 -0.84242300

C -2.55132600 -4.01302400 -1.40028000

C -4.79340400 -3.15676400 -1.61784900

C -2.61849100 -4.52436300 -2.69846900

C -4.86347300 -3.66758900 -2.91417400

C -3.77590900 -4.35427000 -3.46008300

H -1.64704600 -4.14590300 -0.81167200

H -5.63696500 -2.61370900 -1.19975100

H -1.76635200 -5.06008900 -3.11012000

H -5.77154500 -3.53416300 -3.49764300

H -3.83333100 -4.75819300 -4.46801100

C -2.90958100 -0.73341800 2.52646900

C -1.65643200 -1.21472500 3.02249500

H -2.91840000 0.32871900 2.28160400

H -1.63586700 -2.16218800 3.55698400

C -0.47006600 -0.50698500 2.92717500

H -0.48560300 0.44288200 2.39462700

C 0.75254800 -0.84861600 3.52818400

H 0.79183900 -1.72536800 4.16598200

C 1.88470300 -0.01440200 3.44439600

O 1.99066600 1.06486400 2.80486500

N 3.04709600 -0.41292600 4.20211000

C 4.03500900 0.47434900 4.58644000

C 3.38000900 -1.67318500 4.67472800

C 4.98465700 -0.21259700 5.30413700

C 4.57298800 -1.58069200 5.35211400

H 3.94175300 1.51646800 4.32670400

H 2.76373300 -2.53026400 4.45365700

H 5.87208900 0.21502800 5.75219700

H 5.09406000 -2.40169200 5.82758800

H 0.79871300 -1.09946600 -0.52696800

N 1.80966600 -1.04293000 -0.69400000

P 2.54085400 0.39207400 -0.99650600

N 4.05240200 -0.28322900 -1.08198700

C 4.07616000 -1.71127400 -0.66419500

C 2.62585000 -2.21838100 -1.07641700

C 5.26532600 0.37896100 -1.54829900

H 5.02728800 1.39381200 -1.87104800

H 6.01801900 0.44824300 -0.75985900

H 5.69355400 -0.16888600 -2.39839800

H 4.83640200 -2.20074500 -1.27966700

C 4.45280800 -1.94191400 0.83278100

H 3.52778400 -1.90965600 1.41827000

C 5.38356400 -0.86875900 1.41903300

H 6.37755800 -0.89543700 0.95329200

C 2.14153800 -3.47199700 -0.32713500

C 1.39833100 -3.37949700 0.85680400

C 2.44024400 -4.74878200 -0.83025300

C 0.97619600 -4.53391000 1.52182200

C 2.02553600 -5.89890600 -0.16065700

C 1.28839200 -5.79658700 1.02077400

H 1.13290300 -2.41370400 1.27020800

H 2.99735500 -4.84889900 -1.75647500

H 0.39487700 -4.43386400 2.43443100

H 2.27130700 -6.87532300 -0.56989400

H 0.95551400 -6.69204800 1.53877400

C 2.47926800 -2.46368900 -2.60310600

C 1.17862800 -2.46965800 -3.13771500

C 3.54596400 -2.72137200 -3.47238100

C 0.95634300 -2.70910600 -4.49164700

C 3.32554300 -2.95700900 -4.83484200

C 2.03221400 -2.94980700 -5.35087400

H 0.32809500 -2.29397100 -2.48496200

H 4.56718100 -2.75324900 -3.10688800

H -0.06179500 -2.71328300 -4.87149300

H 4.17364900 -3.15068600 -5.48665300

H 1.86140300 -3.13620900 -6.40770600

N 1.94201200 1.49306400 0.06057600

C 1.97011200 2.87822900 -0.46912200

C 1.58645800 2.61806200 -1.99210000

N 2.35561600 1.38774100 -2.30798300

H 1.89588100 1.30891900 1.08174600

C 2.87737600 1.16494900 -3.65072600

H 3.41423800 0.21562200 -3.68502600

H 2.07466000 1.11907300 -4.39058900

H 3.56814400 1.97194600 -3.92993700

H 1.99829200 3.41095000 -2.62254400

C 0.05573100 2.51698300 -2.28170500

H -0.43344400 2.12870200 -1.38450500

C -0.31890800 1.54712500 -3.41468000

H 0.00215600 1.91575100 -4.39787500

C 0.96911400 3.75216000 0.30580000

C -0.15970800 3.18949800 0.91627300

C 1.15388600 5.13975900 0.40267800

C -1.08508100 3.98966200 1.58687200

C 0.23039400 5.94028400 1.07496700

C -0.89471800 5.36841700 1.66998900

H -0.32416700 2.12032800 0.86463700

H 2.02855100 5.60291800 -0.04135100

H -1.95609100 3.52352300 2.03804800

H 0.39804800 7.01232600 1.13818200

H -1.61032700 5.99216500 2.19958700

C 3.40024700 3.46140700 -0.31779200

C 4.01161400 4.28671000 -1.27121700

C 4.10678900 3.18517500 0.86577800

C 5.29648500 4.79996200 -1.06452500

C 5.38593400 3.69928800 1.07354000

C 5.99117900 4.50547700 0.10663500

H 3.49500600 4.55500400 -2.18733900

H 3.65126100 2.56447700 1.63251400

H 5.74755300 5.43303300 -1.82442000

H 5.90945300 3.46814500 1.99743400

H 6.98949300 4.90321800 0.26807300

C -4.17506700 -1.16245700 3.19166700

C -5.29026800 -0.30632400 3.16921600

C -4.30350100 -2.38896200 3.86762300

C -6.47846800 -0.65328400 3.80931100

H -5.21907000 0.63063700 2.62687300

C -5.49404000 -2.73946100 4.50324700

H -3.46782900 -3.08061100 3.90243900

C -6.58738500 -1.87192800 4.48165900

H -7.32406700 0.02960700 3.77960200

H -5.56478500 -3.69315800 5.02035400

H -7.51346000 -2.14376300 4.98158000

C -0.53011500 3.90462400 -2.59509800

H -0.11222200 4.29703800 -3.53318200

H -1.61641700 3.83582500 -2.71981400

H -0.33284900 4.63417300 -1.80649200

H 0.09834700 0.54766100 -3.26333300

H 4.97396700 0.13935100 1.31681800

C 5.10932700 -3.31971400 1.03068600

H 6.07385500 -3.36317200 0.50572800

H 5.30524300 -3.48573100 2.09543500

H 4.49090000 -4.14463600 0.67162700

H 5.51686100 -1.05610200 2.48862400

H -1.40810900 1.43990500 -3.43759100

- **1ba**-*RR* (HH-2)


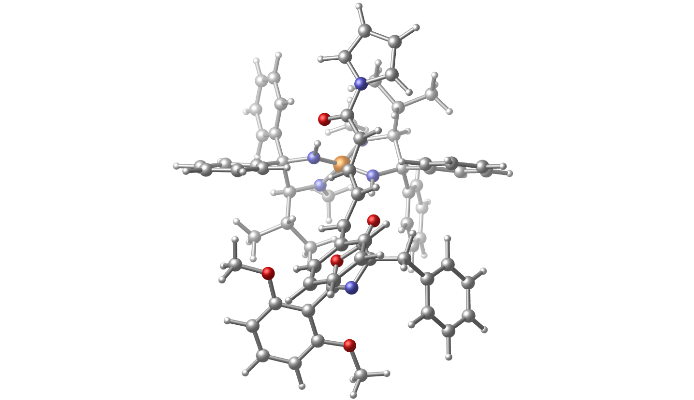

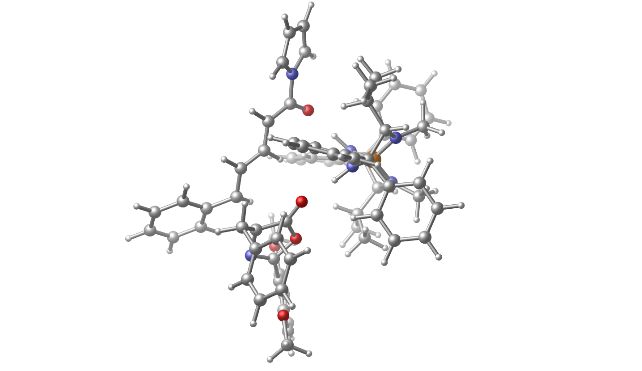


B3LYP/6-31G*

E = –3719.223197

Zero-point correction = 1.283914 (Hartree/Particle)

Thermal correction to Energy = 1.359692

Thermal correction to Enthalpy = 1.360637

Thermal correction to Gibbs Free Energy = 1.164711

Sum of electronic and zero-point Energies = –3717.939284

Sum of electronic and thermal Energies = –3717.863505

Sum of electronic and thermal Enthalpies = –3717.862561

Sum of electronic and thermal Free Energies = –3718.058486

PCM(toluene)-B3LYP-D3/6-311++G**//B3LYP/6-31G*@–30 °C

E = –3720.390173

G = –3719.191725

0 1

O -1.05852600 -1.08949200 0.05733700

C -2.17634200 -0.58591000 0.10157500

C -3.34123100 -0.87552600 0.91217500

O -2.50412200 0.54036600 -0.65205800

N -4.39116500 -0.05691500 0.43355400

C -3.85910400 0.75016100 -0.41126600

C -4.51550900 1.82983900 -1.17229000

C -4.24415100 3.17983000 -0.88386200

C -5.43760800 1.51767300 -2.19045400

C -4.88379700 4.20511900 -1.59286000

C -6.08304600 2.53899600 -2.90194500

C -5.79537100 3.86589300 -2.59118000

O -3.34886600 3.39362800 0.12348900

C -3.04585800 4.73435700 0.48898100

H -3.93521700 5.25615600 0.86480800

H -2.62286700 5.29380300 -0.35419600

H -2.29661100 4.66445300 1.27786100

O -5.62482100 0.19293600 -2.42318700

C -6.54915100 -0.20605500 -3.42192900

H -7.56438500 0.14384700 -3.19409900

H -6.25451800 0.15881400 -4.41490300

H -6.53014400 -1.29705600 -3.41500100

H -4.68835700 5.24647200 -1.36951800

H -6.79334800 2.30850200 -3.68648200

H -6.29560100 4.65859400 -3.14134000

C -3.64991900 -2.29147000 1.34063700

H -2.76499100 -2.69291100 1.84694200

H -4.46692600 -2.25739100 2.06967200

C -4.03103600 -3.19718800 0.18116300

C -3.10150500 -4.09218700 -0.36467600

C -5.31643000 -3.14163900 -0.37754200

C -3.44710600 -4.91607000 -1.43841200

C -5.66441600 -3.96624800 -1.44774800

C -4.73073400 -4.85793400 -1.98268600

H -2.09963700 -4.14177800 0.05452200

H -6.03953000 -2.43889300 0.02743600

H -2.71162200 -5.60650500 -1.84427100

H -6.67048700 -3.92004100 -1.85918600

H -5.00377000 -5.50426500 -2.81335400

C -2.69791400 0.10112700 2.65521700

C -1.56895100 -0.54514900 3.24377900

H -2.46818900 1.01786500 2.11255000

H -1.74364900 -1.30077200 4.00716400

C -0.25482500 -0.21817900 2.95283400

H -0.08593800 0.54850700 2.19893700

C 0.88582600 -0.74623900 3.57983900

H 0.75280300 -1.50565500 4.34346700

C 2.18156400 -0.24300800 3.35074500

O 2.50919800 0.73229500 2.62396300

N 3.26068100 -0.88710000 4.05595100

C 4.47199600 -0.27276100 4.31544600

C 3.30365100 -2.17681100 4.56412100

C 5.27516900 -1.15999000 4.99107200

C 4.53512400 -2.37426700 5.14099200

H 4.62612700 0.74901100 4.00828900

H 2.47397900 -2.85240700 4.42843000

H 6.27756000 -0.96182000 5.34769800

H 4.87154400 -3.28782300 5.61388500

H 0.63693900 -1.05576300 -0.50823100

N 1.63082800 -1.13612800 -0.75781500

P 2.52602700 0.17261300 -1.17567600

N 3.89235400 -0.72232100 -1.45624900

C 3.76271100 -2.12910700 -0.98937600

C 2.21783400 -2.42176500 -1.21307100

C 5.11928800 -0.25630000 -2.09122400

H 4.99103500 0.77457600 -2.42428600

H 5.96760600 -0.28179000 -1.40340000

H 5.35295900 -0.88101600 -2.96366500

H 4.36487300 -2.74031800 -1.66778800

C 4.27768300 -2.37953200 0.46368100

H 3.44366800 -2.19866700 1.14990100

C 5.41564700 -1.44161400 0.89778800

H 6.33330800 -1.62244100 0.32265000

C 1.66101400 -3.59076700 -0.38368300

C 1.10673900 -3.39375200 0.88737200

C 1.70771400 -4.89700300 -0.89712700

C 0.62622600 -4.47674300 1.62791900

C 1.23269800 -5.97716200 -0.15452100

C 0.68850900 -5.77155700 1.11487300

H 1.03120400 -2.39925300 1.31007100

H 2.11286600 -5.07485900 -1.88838400

H 0.19609700 -4.29533700 2.60921400

H 1.28173500 -6.97876500 -0.57364800

H 0.31019700 -6.61099100 1.69205500

C 1.85422000 -2.64501300 -2.70581800

C 0.50511700 -2.49769800 -3.07440600

C 2.76977200 -3.01071800 -3.69976200

C 0.09328000 -2.69245000 -4.39054900

C 2.35826400 -3.20480300 -5.02380800

C 1.02061500 -3.04383000 -5.37557100

H -0.23172900 -2.23521200 -2.32022300

H 3.81852400 -3.15733400 -3.46373100

H -0.95669200 -2.57449600 -4.64433200

H 3.09205700 -3.48568500 -5.77501400

H 0.70042300 -3.19600200 -6.40277500

N 2.27940400 1.34446200 -0.05234200

C 2.47623700 2.70717500 -0.60139800

C 1.80054300 2.53543900 -2.02590600

N 2.30807900 1.20561800 -2.45349000

H 2.35331700 1.12894100 0.95953200

C 2.55754200 0.91565200 -3.85979000

H 2.98330700 -0.08412000 -3.95783900

H 1.63797400 0.94656500 -4.44948800

H 3.26840000 1.64227400 -4.27510400

H 2.20622200 3.26537900 -2.73240300

C 0.24130700 2.66523900 -2.03259200

H -0.12511400 2.38363100 -1.03866000

C -0.46055400 1.73826300 -3.03916900

H -0.23854400 2.02227700 -4.07597000

C 1.83589300 3.77583800 0.30021700

C 1.02282700 3.44762400 1.38982900

C 2.10956800 5.13346200 0.06020100

C 0.49207000 4.44705600 2.21174400

C 1.56737900 6.13017900 0.86793700

C 0.75430100 5.79066900 1.95245200

H 0.83233300 2.41238200 1.63787300

H 2.76366200 5.41631500 -0.75919100

H -0.11432100 4.16076600 3.06703900

H 1.79458800 7.17232900 0.65913800

H 0.34721400 6.56566100 2.59651100

C 3.99704200 3.01585300 -0.68979000

C 4.60924500 3.65124500 -1.77758500

C 4.79890400 2.67741300 0.41396500

C 5.98181900 3.92336200 -1.77451200

C 6.16522600 2.95137600 0.41955300

C 6.76613800 3.57333200 -0.67788400

H 4.03019200 3.94920500 -2.64594600

H 4.34860000 2.19318600 1.27627400

H 6.43124600 4.41242400 -2.63502900

H 6.76097000 2.67862900 1.28651500

H 7.83181800 3.78544300 -0.67447700

C -3.97213700 0.20581300 3.41693200

C -4.79644400 1.32860500 3.22599900

C -4.38476600 -0.76140000 4.35103800

C -5.97940100 1.48540900 3.94483400

H -4.49952600 2.08060500 2.50089900

C -5.57186800 -0.60873600 5.06593800

H -3.77471500 -1.64241800 4.52453200

C -6.37444500 0.51654700 4.86911200

H -6.59579500 2.36573600 3.78059400

H -5.86793500 -1.37070800 5.78263200

H -7.29718000 0.63668900 5.43081200

C -0.19124700 4.11357600 -2.32183600

H 0.13506300 4.42170100 -3.32518900

H -1.28422600 4.18661700 -2.29787800

H 0.20982100 4.82922400 -1.60138900

H -0.19421600 0.68741400 -2.89920700

H 5.14549000 -0.38644400 0.80549100

C 4.74759400 -3.83444200 0.63990200

H 5.63162400 -4.02998700 0.01684300

H 5.03308500 -4.00686200 1.68314600

H 3.98100200 -4.56649000 0.37850600

H 5.64543800 -1.62372000 1.95217700

H -1.54225200 1.81502400 -2.89876100

- **1ba**-*RR* (MeMe)


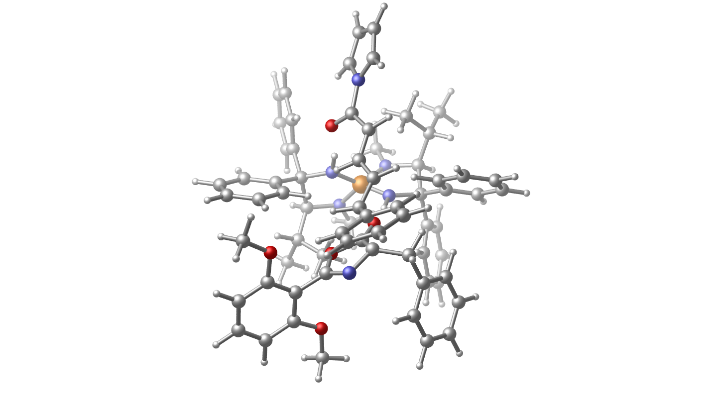

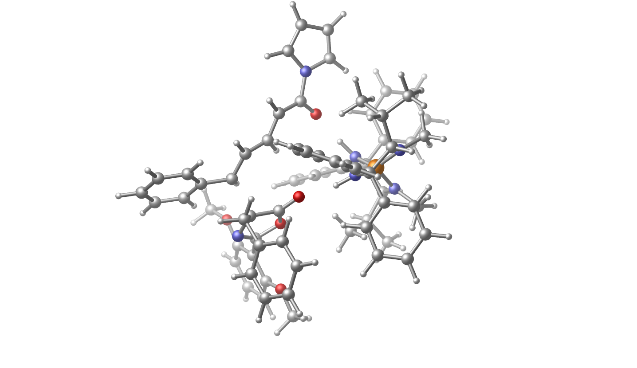


B3LYP/6-31G*

E = –3719.221152

Zero-point correction = 1.283776 (Hartree/Particle)

Thermal correction to Energy = 1.359805

Thermal correction to Enthalpy = 1.360750

Thermal correction to Gibbs Free Energy = 1.164227

Sum of electronic and zero-point Energies = –3717.937376

Sum of electronic and thermal Energies = –3717.861347

Sum of electronic and thermal Enthalpies = –3717.860402

Sum of electronic and thermal Free Energies = –3718.056925

PCM(toluene)-B3LYP-D3/6-311++G**//B3LYP/6-31G*@–30 °C

E = –3720.3874

G = –3719.189329

0 1

O 0.93886000 0.81634000 0.14331200

C 2.10148200 0.43605800 0.09998700

C 3.29983300 0.85315300 0.81202900

O 2.47100000 -0.67546900 -0.65420000

N 4.38093400 0.11688300 0.24918100

C 3.85217900 -0.75794300 -0.52553600

C 4.52324200 -1.80756900 -1.31900400

C 4.91119600 -3.02669000 -0.73776800

C 4.78176200 -1.58682500 -2.68435800

C 5.57439100 -3.99919200 -1.49780200

C 5.44251000 -2.55394600 -3.45228800

C 5.83148800 -3.74524300 -2.84339000

O 4.58486800 -3.18633200 0.57983500

C 4.98804000 -4.38011300 1.23965400

H 6.07875700 -4.49627500 1.22375000

H 4.52066500 -5.26460000 0.78869800

H 4.64833300 -4.27617900 2.27105100

O 4.35017600 -0.38934500 -3.16634200

C 4.65132900 -0.04444800 -4.50947900

H 5.73391000 -0.04108000 -4.69032500

H 4.16733200 -0.72725800 -5.22004600

H 4.25759200 0.96360700 -4.64877100

H 5.88091700 -4.93995900 -1.05791400

H 5.65216000 -2.38778100 -4.50182100

H 6.34548000 -4.49862900 -3.43437400

C 3.50728100 2.32073800 1.12557700

H 2.66626600 2.65771600 1.74246000

H 4.41593700 2.41309500 1.72981300

C 3.61556100 3.20371900 -0.10733400

C 2.61873900 4.14111500 -0.40715200

C 4.71775400 3.10111900 -0.97047700

C 2.71713600 4.95794600 -1.53714400

C 4.81724700 3.91381300 -2.09953600

C 3.81739000 4.84692300 -2.38825100

H 1.76060500 4.23714500 0.25351800

H 5.49246000 2.37101300 -0.75364500

H 1.93394200 5.68254100 -1.74617500

H 5.68262700 3.82420400 -2.75224300

H 3.90077200 5.48650800 -3.26370300

C 2.98213800 -0.09067500 2.57750100

C 1.73372300 0.23303400 3.20577500

H 3.00114200 -1.07361000 2.10624300

H 1.72379500 1.00333200 3.97412500

C 0.55230800 -0.45031600 2.96834500

H 0.55383700 -1.21922900 2.19673200

C -0.64057200 -0.31885700 3.69686300

H -0.66319100 0.35566800 4.54653700

C -1.77991400 -1.09749300 3.40826200

O -1.98956200 -1.76863700 2.36343100

N -2.82076000 -1.13154900 4.40047000

C -4.10254900 -1.58908600 4.14730600

C -2.74418600 -0.76453000 5.73653900

C -4.83568300 -1.49822600 5.30461800

C -3.96977700 -0.97794600 6.31688400

H -4.36289500 -1.92379300 3.15682800

H -1.81440000 -0.42687400 6.16533700

H -5.87531400 -1.77652300 5.41946100

H -4.21676100 -0.79613800 7.35489100

H -0.78127400 1.19268000 -0.40863000

N -1.78733400 1.33669000 -0.53058700

P -2.75757300 0.13437000 -1.07744000

N -4.14228700 1.03831100 -0.90710600

C -3.93189100 2.36918900 -0.28944600

C -2.39560800 2.68158000 -0.56310100

C -5.48537700 0.54093200 -1.18140400

H -5.42519800 -0.32020500 -1.85033400

H -6.00486100 0.22381500 -0.27089000

H -6.07905600 1.32383700 -1.66831200

H -4.52648300 3.08054700 -0.86919400

C -4.43097800 2.49967900 1.18152100

H -3.92416200 3.38074400 1.58788400

C -4.08036800 1.30550800 2.07362100

H -4.53917100 0.37866500 1.71199300

C -1.77943400 3.59988800 0.50408100

C -0.99790400 3.10516300 1.55486000

C -2.02575400 4.98127300 0.44120600

C -0.48134300 3.97423600 2.52076500

C -1.51742400 5.84378900 1.41041800

C -0.73935600 5.34244700 2.45663800

H -0.77297100 2.04866100 1.63165700

H -2.61240500 5.38793400 -0.37775600

H 0.12579200 3.56669100 3.32433500

H -1.72257700 6.90891900 1.34229400

H -0.33574200 6.01392200 3.20967800

C -2.13099000 3.30648800 -1.96400200

C -0.79302800 3.35130300 -2.39650900

C -3.10965900 3.83350000 -2.81389400

C -0.45033100 3.89219800 -3.63234900

C -2.76846700 4.37513500 -4.05962300

C -1.44061800 4.40549900 -4.47538000

H -0.00516500 2.96670600 -1.75502500

H -4.15663500 3.83823500 -2.52940600

H 0.59485900 3.91962800 -3.92786500

H -3.55152100 4.77514700 -4.69875900

H -1.17643200 4.82897700 -5.44065000

N -2.27214700 -1.24726800 -0.35325800

C -2.44846400 -2.46756600 -1.17779600

C -2.30464400 -1.91933900 -2.66393700

N -2.86056900 -0.55136300 -2.58881800

H -2.11999100 -1.33110700 0.67203900

C -3.27816100 0.14769200 -3.79682800

H -3.88155500 1.01601200 -3.52647800

H -2.42941700 0.50099600 -4.39270600

H -3.88643500 -0.52269200 -4.41578200

H -2.97971000 -2.50306200 -3.29460200

C -0.90252100 -2.01096800 -3.34818700

H -0.43437200 -2.92259800 -2.96673800

C 0.03455000 -0.83831100 -3.03213200

H -0.33671200 0.10424500 -3.45228800

C -1.36631100 -3.50448700 -0.84172000

C -0.14978400 -3.12908600 -0.25948400

C -1.56079700 -4.85043800 -1.18505600

C 0.85287400 -4.07383900 -0.03508700

C -0.55999900 -5.79501000 -0.95904500

C 0.65156000 -5.40948900 -0.38286800

H 0.02378300 -2.09460600 0.00943800

H -2.50289000 -5.16569600 -1.62374700

H 1.79422100 -3.75382900 0.40295900

H -0.73168000 -6.83361100 -1.22986400

H 1.43079800 -6.14649800 -0.20617800

C -3.85251800 -3.06381600 -0.87842700

C -4.85325900 -3.29322400 -1.82967000

C -4.12949500 -3.39268500 0.46216100

C -6.09401900 -3.82735200 -1.45914500

C -5.36234100 -3.92517500 0.83008900

C -6.35458600 -4.14575000 -0.12988000

H -4.69572800 -3.06538800 -2.87861600

H -3.37540200 -3.21934300 1.22512500

H -6.85103100 -3.99433300 -2.22132300

H -5.54519500 -4.17262500 1.87256100

H -7.31585300 -4.56335400 0.15747900

C 4.25137900 0.18789700 3.31761800

C 5.36260900 -0.65062900 3.12265700

C 4.38362700 1.25040900 4.22912500

C 6.55109700 -0.44678800 3.82153500

H 5.28781800 -1.46059000 2.40440200

C 5.57397100 1.45853900 4.92496100

H 3.55068400 1.92504900 4.40050100

C 6.66361600 0.60841800 4.72833000

H 7.39358000 -1.11367000 3.65496200

H 5.64789200 2.28680700 5.62532600

H 7.58975100 0.76830000 5.27440100

C -1.03870000 -2.20899300 -4.87090900

H -1.46730000 -1.33517900 -5.37486700

H -0.05180600 -2.38554500 -5.31378700

H -1.66641700 -3.07645300 -5.11078900

H 0.18251500 -0.69617600 -1.96073900

H -3.00193300 1.14428700 2.13959800

C -5.93803000 2.81741700 1.24473300

H -6.56362900 1.97388800 0.93530700

H -6.22058400 3.06612100 2.27371000

H -6.19722600 3.67814100 0.61492400

H -4.44935900 1.46988500 3.09116900

H 1.02520500 -1.02160300 -3.46226500

- **1ba**-*RR* (MeMe-2)


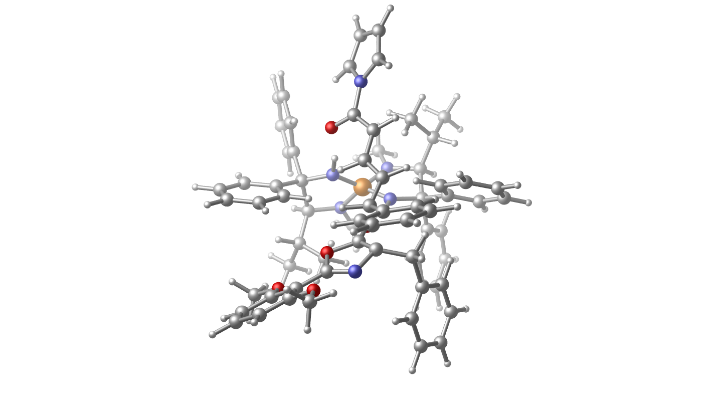

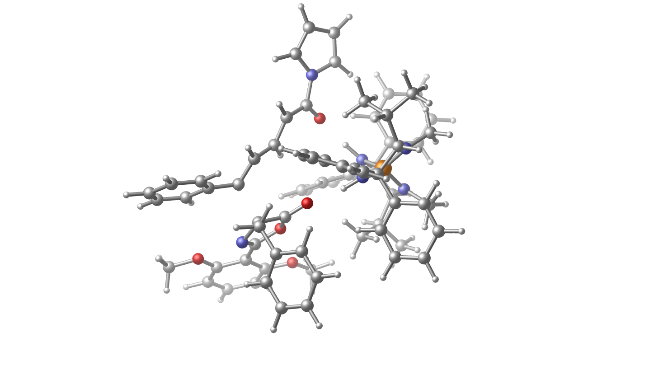


B3LYP/6-31G*

E = –3719.215704

Zero-point correction = 1.284403 (Hartree/Particle)

Thermal correction to Energy = 1.359861

Thermal correction to Enthalpy = 1.360805

Thermal correction to Gibbs Free Energy = 1.168187

Sum of electronic and zero-point Energies = –3717.931301

Sum of electronic and thermal Energies = –3717.855843

Sum of electronic and thermal Enthalpies = –3717.854899

Sum of electronic and thermal Free Energies = –3718.047516

PCM(toluene)-B3LYP-D3/6-311++G**//B3LYP/6-31G*@–30 °C

E = –3720.383522

G = –3719.182208

0 1

O -0.93197000 -0.39629800 -0.24562500

C -2.13274000 -0.16438000 -0.18420600

C -3.27535000 -0.95214700 0.26723300

O -2.63383600 1.09806000 -0.46665300

N -4.43069600 -0.19614400 -0.04493100

C -4.02461600 0.97710600 -0.40472300

C -4.83387800 2.15761000 -0.73758000

C -4.34864000 3.21585700 -1.55629800

C -6.16744000 2.27404900 -0.24821500

C -5.14328100 4.33445600 -1.84013400

C -6.95567200 3.39241100 -0.54692300

C -6.43548000 4.41200400 -1.33476300

O -3.09582700 3.07967300 -2.06519400

C -2.55608200 4.14000600 -2.83813800

H -2.49527200 5.06972900 -2.25940600

H -3.13998800 4.31312900 -3.75176700

H -1.54889600 3.82221400 -3.10739900

O -6.62131900 1.26349600 0.53103100

C -7.96618100 1.28199300 0.98112300

H -8.16260100 2.13427300 1.64553000

H -8.67072900 1.30946000 0.14010600

H -8.09508800 0.35141200 1.53515800

H -4.76300000 5.13457000 -2.46114000

H -7.96411400 3.47079700 -0.16207800

H -7.04849900 5.27992000 -1.56234900

C -3.31154300 -2.44736000 0.02521600

H -2.42278600 -2.88932300 0.48854500

H -4.18746300 -2.84747800 0.54744100

C -3.37053200 -2.85380900 -1.43913100

C -2.34577800 -3.61994100 -2.00901000

C -4.46449300 -2.49735400 -2.24361100

C -2.40755200 -4.02144800 -3.34650800

C -4.52552900 -2.89264900 -3.57949700

C -3.49760100 -3.65757400 -4.13747500

H -1.49606900 -3.91392100 -1.39756400

H -5.26241400 -1.89950000 -1.81281100

H -1.60245900 -4.62021600 -3.76512300

H -5.38146000 -2.60676900 -4.18631000

H -3.55043500 -3.97118900 -5.17723700

C -3.05929200 -0.67518200 2.24606400

C -1.79224900 -1.09323200 2.78491500

H -3.16594200 0.40691000 2.15966500

H -1.74451300 -2.06305700 3.27686500

C -0.66838300 -0.28718600 2.84308100

H -0.70641100 0.68265300 2.34888500

C 0.50539900 -0.56149900 3.56483600

H 0.54777300 -1.45740600 4.17565000

C 1.59726000 0.32941500 3.57675400

O 1.79633900 1.28460200 2.77967600

N 2.59899600 0.12700700 4.58832400

C 3.86329200 0.68818300 4.53611100

C 2.49809700 -0.61526300 5.75629900

C 4.56225200 0.29458400 5.65043300

C 3.69193600 -0.53095000 6.42907200

H 4.14000200 1.30320400 3.69583800

H 1.57246000 -1.09765500 6.02662500

H 5.58222500 0.56816500 5.88765900

H 3.91392000 -0.99483600 7.38147100

H 0.83226300 -1.02859000 -0.64916700

N 1.83986400 -1.18470300 -0.71379500

P 2.88882400 0.06869700 -0.86946900

N 4.22643700 -0.90397200 -0.72430900

C 3.92745900 -2.33711500 -0.49465800

C 2.42804100 -2.50627700 -1.00821400

C 5.60149800 -0.41882000 -0.74204900

H 5.63207700 0.57692200 -1.18955800

H 6.02730300 -0.34890200 0.26446100

H 6.22621800 -1.09241300 -1.34085100

H 4.57531200 -2.89505800 -1.17517900

C 4.25787000 -2.86410900 0.93357900

H 3.69243700 -3.79578300 1.04169900

C 3.83789700 -1.92295700 2.06518300

H 4.36256800 -0.96310000 2.01097300

C 1.66862600 -3.62643700 -0.27943600

C 0.79144000 -3.36081500 0.77952900

C 1.87578700 -4.96273500 -0.65929700

C 0.14829900 -4.40878800 1.44498500

C 1.23849900 -6.00537400 0.01074900

C 0.36907100 -5.73238900 1.06879200

H 0.59689700 -2.34413400 1.10010900

H 2.53477700 -5.19303000 -1.49090100

H -0.52724200 -4.17560600 2.26308900

H 1.41612000 -7.03112100 -0.30138100

H -0.13336200 -6.54334300 1.58896200

C 2.34787200 -2.76448600 -2.54292500

C 1.13791900 -2.45930100 -3.19023600

C 3.37631500 -3.31922100 -3.31561600

C 0.97080400 -2.67856900 -4.55589800

C 3.21380200 -3.53650400 -4.68867300

C 2.01377500 -3.21348200 -5.31653700

H 0.30718600 -2.05368900 -2.62140100

H 4.32347700 -3.60230700 -2.86781200

H 0.01755600 -2.43919100 -5.01872700

H 4.03359400 -3.96327300 -5.26077000

H 1.88721300 -3.38355800 -6.38224400

N 2.34875000 1.29577400 0.06101900

C 2.61557000 2.64888300 -0.48890900

C 2.64503500 2.40381800 -2.05966800

N 3.18421900 1.03527900 -2.19038500

H 2.08759700 1.18872000 1.06365800

C 3.73571800 0.57065700 -3.45705600

H 4.30991000 -0.34321000 -3.29374500

H 2.96066300 0.35083100 -4.19940200

H 4.40611400 1.33638200 -3.86501900

H 3.39000300 3.08320300 -2.48107300

C 1.33552600 2.66377500 -2.87215700

H 0.83589400 3.50288200 -2.37884100

C 0.35795600 1.48189700 -2.89628100

H 0.78644300 0.61321000 -3.40956600

C 1.49906100 3.62280900 -0.08246000

C 0.23134200 3.16626400 0.29688900

C 1.72492900 5.00595200 -0.15260900

C -0.78842000 4.07239600 0.59474400

C 0.70598900 5.91008500 0.14559500

C -0.55620400 5.44587200 0.52269300

H 0.02927800 2.10339500 0.34916000

H 2.70579200 5.37978100 -0.43157100

H -1.76558800 3.69187400 0.87571000

H 0.90303700 6.97770800 0.09092600

H -1.34850400 6.14963200 0.76469000

C 3.97556200 3.14821500 0.07434200

C 5.09473300 3.48200000 -0.69741200

C 4.08420900 3.25759500 1.47347700

C 6.28547500 3.91052200 -0.09714100

C 5.26759000 3.68513200 2.07019600

C 6.37763200 4.01563600 1.28723600

H 5.07101500 3.41912600 -1.78015500

H 3.23860600 2.98992300 2.10089900

H 7.13665300 4.16419700 -0.72403600

H 5.31946200 3.76284000 3.15306100

H 7.29988400 4.35200500 1.75332800

C -4.28386400 -1.29840100 2.83722600

C -5.40495500 -0.49969700 3.11412100

C -4.34703100 -2.65949500 3.18643800

C -6.53569300 -1.03293000 3.73244600

H -5.38413700 0.54991800 2.84079100

C -5.48244200 -3.19773600 3.79045300

H -3.49981400 -3.30819200 2.98546800

C -6.58257300 -2.38589100 4.07200100

H -7.38021600 -0.38611000 3.95854200

H -5.50378400 -4.25370300 4.04806600

H -7.46327700 -2.80243700 4.55384800

C 1.65965900 3.13911100 -4.30251000

H 2.13895600 2.36097600 -4.90666900

H 0.73799900 3.42631200 -4.82214200

H 2.32056300 4.01496500 -4.29692800

H 0.04685000 1.17000400 -1.89726100

H 2.76438600 -1.72063600 2.05853800

C 5.74259700 -3.25799700 1.06315000

H 6.41365900 -2.39301800 1.04760600

H 5.90644400 -3.77383700 2.01575700

H 6.05338300 -3.94035500 0.26147900

H 4.08070800 -2.36504700 3.03723100

H -0.55284400 1.75756500 -3.43925700

- **1ba**-*RR* (HMe)


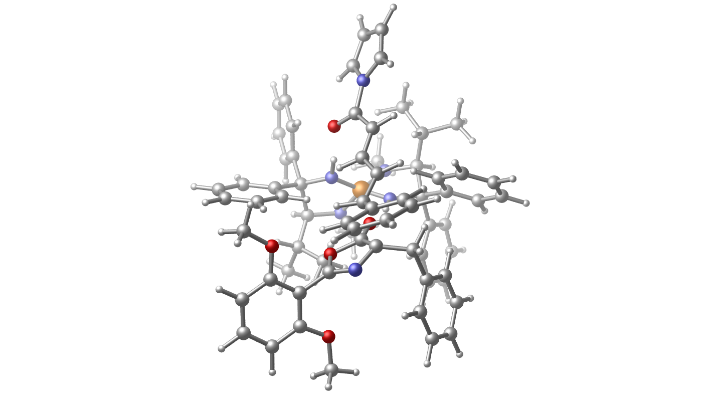

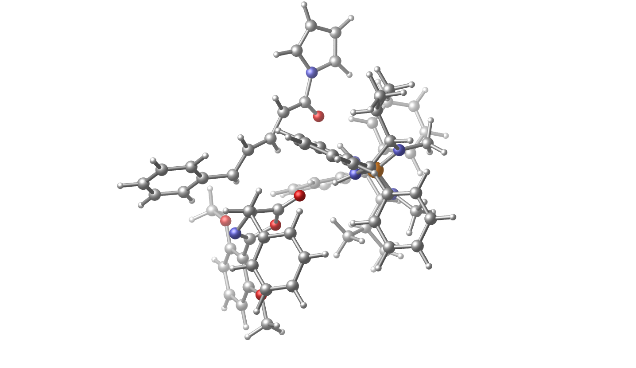


B3LYP/6-31G*

E = –3719.221859

Zero-point correction = 1.284445 (Hartree/Particle)

Thermal correction to Energy = 1.360165

Thermal correction to Enthalpy = 1.361109

Thermal correction to Gibbs Free Energy = 1.165363

Sum of electronic and zero-point Energies = –3717.937414

Sum of electronic and thermal Energies = –3717.861694

Sum of electronic and thermal Enthalpies = –3717.860749

Sum of electronic and thermal Free Energies = –3718.056496

PCM(toluene)-B3LYP-D3/6-311++G**//B3LYP/6-31G*@–30 °C

E = –3720.388279

G = –3719.189212

0 1

O 0.90457300 0.55463100 -0.00805200

C 2.10534400 0.31735800 0.03435300

C 3.21959400 0.96081600 0.70969200

O 2.62857800 -0.83839700 -0.54744200

N 4.39729800 0.28769800 0.28624700

C 4.00327500 -0.73926500 -0.37320300

C 4.83063900 -1.79222100 -0.99753900

C 5.07751400 -3.00740800 -0.33766300

C 5.39202900 -1.57618300 -2.26943900

C 5.88318500 -3.98984600 -0.92733000

C 6.19863500 -2.55367900 -2.86761400

C 6.43158600 -3.74539000 -2.18493500

O 4.48841900 -3.14262500 0.88923900

C 4.76276200 -4.31406300 1.64775300

H 5.83599000 -4.41502100 1.85144800

H 4.40264700 -5.21622300 1.13735600

H 4.22355700 -4.19016600 2.58794900

O 5.08851500 -0.38033000 -2.84112000

C 5.65147600 -0.06110700 -4.10337500

H 6.74853000 -0.05902200 -4.06561400

H 5.31734300 -0.75850800 -4.88296000

H 5.29416700 0.94344000 -4.33571400

H 6.08272800 -4.92777900 -0.42430200

H 6.63666300 -2.39474100 -3.84536600

H 7.05676200 -4.50574500 -2.64549200

C 3.25769100 2.46727200 0.84803000

H 2.34459000 2.78697500 1.36210100

H 4.10776000 2.72791200 1.48804200

C 3.37330600 3.19521900 -0.48242200

C 2.29917100 3.93772700 -0.98974500

C 4.55716400 3.13276300 -1.23357100

C 2.40167300 4.60336300 -2.21436100

C 4.66217400 3.79820600 -2.45501600

C 3.58447600 4.53703100 -2.95143700

H 1.37657000 4.00014500 -0.41828900

H 5.39088300 2.54692000 -0.85730100

H 1.55568400 5.17632400 -2.58635700

H 5.59206500 3.74740500 -3.01727600

H 3.66962600 5.06069100 -3.90064000

C 2.90336300 0.19462600 2.58490000

C 1.63882600 0.56125300 3.14744300

H 2.95577200 -0.83155400 2.22108700

H 1.60456400 1.41197900 3.82546400

C 0.46664800 -0.15354800 2.95893200

H 0.48947800 -1.00755600 2.28390100

C -0.74597100 0.06955600 3.63044400

H -0.78691400 0.84645200 4.38682500

C -1.88056500 -0.73994900 3.41647500

O -2.06269200 -1.55013200 2.47090600

N -2.94974500 -0.63883100 4.37548900

C -4.20768100 -1.17862800 4.17111600

C -2.92569800 -0.05324100 5.63391700

C -4.97860300 -0.92416000 5.27878600

C -4.16099800 -0.21122700 6.21080700

H -4.42482000 -1.68605100 3.24596900

H -2.02263200 0.38669800 6.02499700

H -6.01070900 -1.22063600 5.41392700

H -4.44536600 0.13227400 7.19701700

H -0.79831200 1.10697200 -0.44238600

N -1.79523400 1.21452700 -0.64352600

P -2.72888100 -0.05002300 -1.10955900

N -4.13850600 0.80956400 -0.94655300

C -3.95490000 2.13032900 -0.28938200

C -2.46984100 2.52994100 -0.70855800

C -5.44104700 0.42312600 -1.47778800

H -5.36622300 -0.56050000 -1.94467700

H -6.19355700 0.36288100 -0.68869300

H -5.77923600 1.14794900 -2.23083400

H -4.66967900 2.80909500 -0.76255700

C -4.25054500 2.13112500 1.24189900

H -3.33678700 1.82692100 1.76491000

C -5.35064200 1.14054900 1.65624900

H -6.32861600 1.42714000 1.24757600

C -1.77061200 3.52087400 0.23954800

C -1.08045700 3.07411800 1.37543700

C -1.82058900 4.90208900 -0.00935200

C -0.47892700 3.98607700 2.24592000

C -1.22002700 5.81057200 0.86211500

C -0.54704600 5.35622100 1.99732600

H -0.99911900 2.01608700 1.59631800

H -2.32927300 5.27618800 -0.89146800

H 0.04863300 3.61068100 3.11802400

H -1.27361000 6.87437700 0.64638200

H -0.07379200 6.06254300 2.67398600

C -2.39560500 3.06632600 -2.16435100

C -1.18542300 2.91291900 -2.86199200

C -3.43962400 3.74396200 -2.80743400

C -1.03163700 3.40179800 -4.15786200

C -3.29124900 4.22820000 -4.11197800

C -2.08904500 4.05677800 -4.79433300

H -0.34975300 2.41173900 -2.38369900

H -4.38398400 3.91908600 -2.30223000

H -0.07970600 3.27394200 -4.66611600

H -4.12140700 4.74453400 -4.58704200

H -1.97288800 4.43492300 -5.80637700

N -2.21070300 -1.37554600 -0.30897200

C -2.40192400 -2.64277500 -1.05131800

C -2.26158800 -2.18930700 -2.57150500

N -2.80356900 -0.81201500 -2.58236400

H -2.08398900 -1.38328900 0.72215600

C -3.19036300 -0.17480300 -3.83509700

H -3.70831600 0.76303900 -3.62693700

H -2.32671100 0.05742400 -4.46648300

H -3.86693000 -0.83366900 -4.39344300

H -2.94695500 -2.80279500 -3.16125900

C -0.86416800 -2.34451500 -3.25261900

H -0.40535100 -3.23072000 -2.80528600

C 0.08940500 -1.16440200 -3.02638600

H -0.27388000 -0.25083800 -3.51215800

C -1.32454100 -3.66089400 -0.64808600

C -0.10368400 -3.24735500 -0.10052700

C -1.52753200 -5.02804000 -0.88578300

C 0.89479500 -4.17745300 0.19271300

C -0.53085200 -5.95763800 -0.58962100

C 0.68486400 -5.53514400 -0.04915400

H 0.07839600 -2.19497700 0.08197800

H -2.47241600 -5.37125200 -1.29657200

H 1.83970400 -3.82589100 0.59708700

H -0.70889300 -7.01324200 -0.77817100

H 1.46078100 -6.26084100 0.18130800

C -3.80972400 -3.21043500 -0.71163300

C -4.79257300 -3.54796700 -1.64965200

C -4.10711800 -3.40301000 0.65103100

C -6.03464300 -4.05419000 -1.24606100

C -5.34101600 -3.90882000 1.05189700

C -6.31484000 -4.23752300 0.10435500

H -4.61877500 -3.43133100 -2.71414100

H -3.37011300 -3.14451900 1.40621300

H -6.77694700 -4.30663600 -1.99910100

H -5.53946900 -4.05024500 2.11115900

H -7.27705100 -4.63343600 0.41795000

C 4.15227100 0.59077900 3.30003500

C 5.26863200 -0.26220200 3.26289500

C 4.26092400 1.78199400 4.03948200

C 6.44024900 0.05290700 3.94841000

H 5.20969700 -1.17744900 2.68211500

C 5.43509500 2.10207300 4.71994500

H 3.42215000 2.46981500 4.08544000

C 6.53041000 1.23752100 4.68140600

H 7.28700500 -0.62781800 3.90692700

H 5.49205000 3.02936800 5.28465100

H 7.44386800 1.48531700 5.21584600

C -1.00764500 -2.65743400 -4.75538100

H -1.43263800 -1.82251600 -5.32385700

H -0.02397800 -2.87516700 -5.18672300

H -1.64269400 -3.53598800 -4.92527800

H 0.24580500 -0.94352900 -1.96915900

H -5.12598200 0.11659300 1.34648500

C -4.65082100 3.53702200 1.72423000

H -5.59271900 3.84897800 1.25175200

H -4.81529800 3.52724100 2.80709600

H -3.89648700 4.29568200 1.50849200

H 1.07165800 -1.39360700 -3.45407900

H -5.44035300 1.13288500 2.74676900

- **1ba**-*RR* (HMe-2)


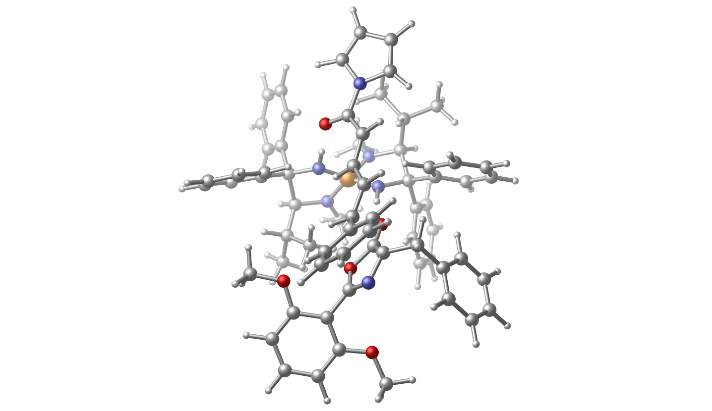

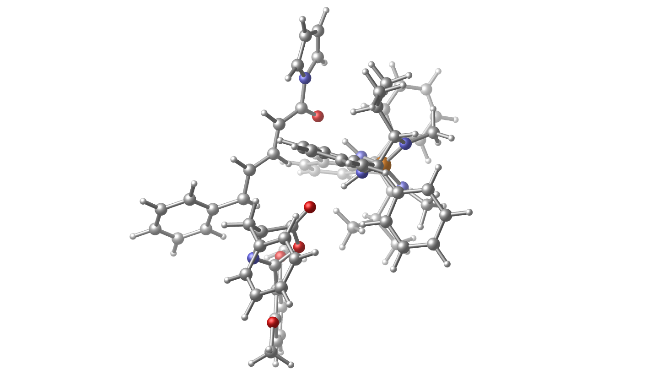


B3LYP/6-31G*

E = –3719.223318

Zero-point correction = 1.283707 (Hartree/Particle)

Thermal correction to Energy = 1.359706

Thermal correction to Enthalpy = 1.360650

Thermal correction to Gibbs Free Energy = 1.164139

Sum of electronic and zero-point Energies = –3717.939610

Sum of electronic and thermal Energies = –3717.863611

Sum of electronic and thermal Enthalpies = –3717.862667

Sum of electronic and thermal Free Energies = –3718.059178

PCM(toluene)-B3LYP-D3/6-311++G**//B3LYP/6-31G*@–30 °C

E = –3720.389364

G = –3719.191382

0 1

O -1.17384400 -0.91007900 -0.05758700

C -2.29699100 -0.42049800 0.02931100

C -3.42917500 -0.71301500 0.88525700

O -2.66607100 0.69341300 -0.72033100

N -4.49772900 0.10402000 0.45698800

C -4.00469700 0.90964800 -0.41460000

C -4.68191900 2.01060800 -1.12124200

C -4.25905400 3.34154000 -0.94119500

C -5.77198000 1.74929800 -1.97565700

C -4.90649400 4.39493200 -1.59869900

C -6.42769600 2.80071600 -2.63188300

C -5.98422500 4.10599600 -2.43390400

O -3.21422300 3.51071300 -0.07873300

C -2.70102600 4.82063300 0.12141300

H -3.44789600 5.47781000 0.58545100

H -2.36276000 5.26648500 -0.82246000

H -1.84627100 4.70709800 0.78981100

O -6.10330600 0.44096800 -2.12134300

C -7.19752300 0.09699300 -2.95458200

H -8.13412300 0.54195400 -2.59404400

H -7.02757100 0.40466400 -3.99511200

H -7.26881700 -0.99073700 -2.90504200

H -4.59133400 5.42118200 -1.45651200

H -7.26572200 2.60832600 -3.29043500

H -6.49290700 4.92092100 -2.94230000

C -3.71816100 -2.12133400 1.34855800

H -2.79173900 -2.53912900 1.75744100

H -4.45299400 -2.07329500 2.16012300

C -4.24328700 -3.01676300 0.23822800

C -3.38495000 -3.87750500 -0.45831300

C -5.59738400 -2.98243300 -0.12454600

C -3.86727100 -4.68835000 -1.48815300

C -6.08156900 -3.79373000 -1.15113900

C -5.21794300 -4.65142800 -1.83730100

H -2.33192300 -3.91074400 -0.19159900

H -6.26812200 -2.30641900 0.39878500

H -3.18499400 -5.35308500 -2.01258700

H -7.13769800 -3.76295400 -1.41041200

H -5.59619200 -5.28752300 -2.63392000

C -2.70015900 0.28461100 2.61967500

C -1.60402600 -0.41888800 3.19898300

H -2.42563600 1.17261800 2.05132800

H -1.81410900 -1.17129500 3.95686200

C -0.27547400 -0.15496000 2.91129800

H -0.06203100 0.61907000 2.17556100

C 0.83072200 -0.76153800 3.53126200

H 0.65074100 -1.53373400 4.27231900

C 2.15235200 -0.32684800 3.32311400

O 2.53452800 0.65525300 2.62980100

N 3.19147200 -1.05290600 4.00640400

C 4.42747400 -0.51025100 4.30663200

C 3.16488300 -2.36410000 4.45687700

C 5.17748400 -1.46429000 4.95150900

C 4.37787200 -2.64648200 5.03849400

H 4.63341700 0.51679500 4.05145600

H 2.30427800 -2.99062900 4.28273300

H 6.18340000 -1.33146100 5.32787900

H 4.66326200 -3.59497200 5.47474000

H 0.57243500 -0.92872100 -0.59197500

N 1.55393200 -1.12524300 -0.82253700

P 2.63576600 0.05368500 -1.18990200

N 3.87652800 -1.01805600 -1.43290400

C 3.54455600 -2.39822500 -0.99822400

C 1.98164600 -2.47658300 -1.27373500

C 5.17011300 -0.71204100 -2.03271200

H 5.16584800 0.31411300 -2.40489400

H 5.98727700 -0.80544300 -1.31300800

H 5.36129400 -1.39042500 -2.87451500

H 4.07682900 -3.07532400 -1.67213600

C 3.98424900 -2.73892500 0.46140000

H 3.16935400 -2.45205900 1.13421700

C 5.23205800 -1.97376900 0.93230600

H 6.12706100 -2.27073200 0.36986200

C 1.25894800 -3.57401300 -0.47595700

C 0.73842000 -3.33556100 0.80223800

C 1.13802500 -4.86466100 -1.01587600

C 0.13517300 -4.36614700 1.52690400

C 0.53793700 -5.89308100 -0.29002100

C 0.03519800 -5.64885400 0.98919900

H 0.78644700 -2.34767900 1.24306300

H 1.51366300 -5.07225700 -2.01263700

H -0.26368800 -4.15292100 2.51500000

H 0.45920200 -6.88393800 -0.72961100

H -0.43883300 -6.44688800 1.55419000

C 1.64249300 -2.63436900 -2.78024800

C 0.35363100 -2.26013900 -3.19947300

C 2.51809800 -3.15753200 -3.73948300

C -0.03578700 -2.38745400 -4.53105800

C 2.13048900 -3.28194000 -5.07861700

C 0.85465500 -2.89479800 -5.48127500

H -0.35571100 -1.87336600 -2.47308500

H 3.51533700 -3.48465900 -3.46377000

H -1.04033300 -2.09414400 -4.82391700

H 2.83323400 -3.68735200 -5.80209900

H 0.55280300 -2.99365400 -6.52045800

N 2.51076600 1.24252200 -0.06634300

C 2.95628400 2.55887300 -0.57183500

C 2.44492900 2.52326800 -2.07474700

N 2.63986500 1.10881000 -2.47186900

H 2.52048000 1.00769800 0.94306600

C 2.68287400 0.71510100 -3.87565000

H 3.15461200 -0.26492600 -3.96932100

H 1.68544800 0.64846900 -4.32239500

H 3.27501400 1.44351600 -4.44118500

H 3.13178800 3.11242500 -2.68871600

C 1.02172200 3.09463200 -2.34032200

H 0.97830900 4.03081200 -1.77382200

C -0.12267400 2.20010800 -1.85620300

H -0.13067600 1.23411800 -2.37368600

C 2.36294900 3.71915200 0.24447600

C 1.55043300 3.51658200 1.36496500

C 2.67598100 5.03865800 -0.12436000

C 1.05120700 4.60733100 2.08551500

C 2.16738800 6.12362700 0.58491000

C 1.34744600 5.91203500 1.69652500

H 1.33408700 2.51578300 1.71763600

H 3.33110600 5.22218100 -0.97165500

H 0.43825800 4.42360600 2.96400500

H 2.42121600 7.13419100 0.27568200

H 0.95973800 6.75621500 2.26052400

C 4.50787700 2.66123500 -0.46701000

C 5.32505200 3.26852300 -1.42943600

C 5.12070100 2.16507800 0.69690400

C 6.70910600 3.35962400 -1.24916600

C 6.49948500 2.25827600 0.87842700

C 7.30385700 2.85314600 -0.09622500

H 4.90382700 3.68850700 -2.33732600

H 4.51598800 1.70164300 1.47124200

H 7.31695600 3.83099000 -2.01726100

H 6.94439000 1.86435000 1.78829200

H 8.37894300 2.92388600 0.04471600

C -3.95881100 0.47129300 3.38654700

C -4.72315200 1.63293300 3.17723500

C -4.41451800 -0.45132400 4.34571300

C -5.88981200 1.86878500 3.90052900

H -4.39087100 2.35379900 2.43591600

C -5.58592100 -0.21952000 5.06505400

H -3.84968800 -1.35838500 4.53744900

C -6.32881400 0.94251200 4.84840400

H -6.45904900 2.77752200 3.72168900

H -5.91603700 -0.94809800 5.80132800

H -7.23903800 1.12442800 5.41390600

C 0.82575600 3.48617900 -3.81824600

H 0.73248600 2.61639900 -4.47682000

H -0.09664700 4.06796200 -3.92656400

H 1.65141000 4.10591800 -4.19136200

H -0.06988200 2.00265300 -0.78378600

H 5.11295900 -0.88998100 0.85321700

C 4.24334200 -4.24689100 0.62636500

H 5.09750600 -4.55959100 0.00945100

H 4.49226800 -4.46584900 1.67023100

H 3.38426400 -4.86166200 0.35162200

H -1.08917800 2.67455900 -2.05028100

H 5.41429800 -2.20219500 1.98701000

- **1ba**-*RR* (MeH)


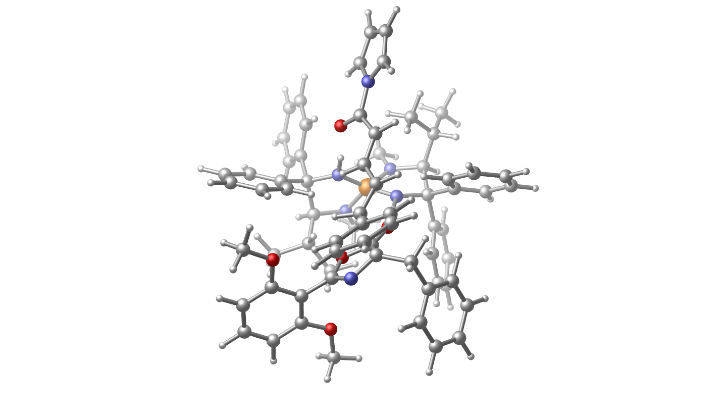

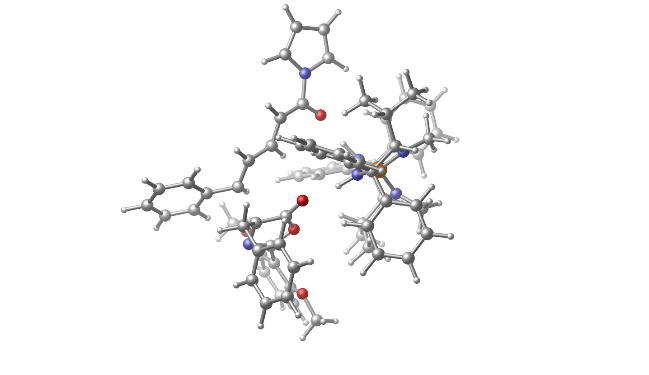


B3LYP/6-31G*

E = –3719.222454

Zero-point correction = 1.283531 (Hartree/Particle)

Thermal correction to Energy = 1.359585

Thermal correction to Enthalpy = 1.360529

Thermal correction to Gibbs Free Energy = 1.163853

Sum of electronic and zero-point Energies = –3717.938923

Sum of electronic and thermal Energies = –3717.862869

Sum of electronic and thermal Enthalpies = –3717.861925

Sum of electronic and thermal Free Energies = –3718.058601

PCM(toluene)-B3LYP-D3/6-311++G**//B3LYP/6-31G*@–30 °C

E = –3720.389858

G = –3719.192132

0 1

O 0.95537300 1.20301800 0.19663600

C 2.06460400 0.69137100 0.12354500

C 3.30614600 0.96909900 0.82642400

O 2.30446500 -0.43499000 -0.65897800

N 4.29807200 0.13471100 0.23771600

C 3.67421700 -0.66706700 -0.54498900

C 4.22122800 -1.77724400 -1.34980700

C 4.58703600 -2.99920500 -0.75644600

C 4.39331600 -1.61391100 -2.73756200

C 5.12795000 -4.03325300 -1.53274400

C 4.93015900 -2.64362200 -3.52030000

C 5.29123500 -3.83917100 -2.90218000

O 4.37074700 -3.09963400 0.58761300

C 4.74865700 -4.30182100 1.24633400

H 5.82198500 -4.49923900 1.13482700

H 4.17792600 -5.15970400 0.86916300

H 4.51758300 -4.14528600 2.30111600

O 4.01043300 -0.40246800 -3.22909400

C 4.29287100 -0.09628400 -4.58683900

H 5.36561200 -0.18332700 -4.80070600

H 3.73084900 -0.74370400 -5.27223700

H 3.97688400 0.93869500 -4.72575300

H 5.41123800 -4.97760700 -1.08535400

H 5.06949800 -2.52043300 -4.58724300

H 5.70923500 -4.64171400 -3.50394100

C 3.66062900 2.40815200 1.14667900

H 2.88169500 2.81018000 1.80474300

H 4.60063200 2.41137200 1.70710500

C 3.78335200 3.28542700 -0.08710900

C 2.76823500 4.18929200 -0.42758500

C 4.90679600 3.19526400 -0.92238400

C 2.87220000 4.98693800 -1.57009300

C 5.01299700 3.98985200 -2.06388400

C 3.99601500 4.89020700 -2.39260600

H 1.89012900 4.26820600 0.20872700

H 5.69483700 2.48929900 -0.67404200

H 2.07607000 5.68698900 -1.81164500

H 5.89510500 3.91082400 -2.69507600

H 4.08343200 5.51617800 -3.27740800

C 2.91376500 0.05801700 2.59206300

C 1.68596300 0.47067000 3.20806200

H 2.86646000 -0.92823500 2.12917700

H 1.71240400 1.27826200 3.93636500

C 0.47417300 -0.16958700 3.00483800

H 0.44680800 -0.97801700 2.27554600

C -0.71119400 0.04571000 3.72443400

H -0.70366300 0.77523100 4.52719400

C -1.87481000 -0.72458400 3.51218200

O -2.11934600 -1.47841100 2.53384700

N -2.90118600 -0.64889800 4.51813300

C -4.18191000 -1.14254600 4.33832000

C -2.81257900 -0.12741100 5.80177600

C -4.90203600 -0.92304300 5.48604600

C -4.02891900 -0.28021500 6.41858300

H -4.44828400 -1.59985900 3.40027800

H -1.88353100 0.26871700 6.17835200

H -5.93673700 -1.19666500 5.64695800

H -4.26550200 0.02239600 7.43048400

H -0.74323500 1.24579900 -0.37742300

N -1.75450700 1.29887100 -0.54964400

P -2.57439000 -0.00291900 -1.11156600

N -4.02744600 0.80101700 -1.16894600

C -3.97325000 2.18561800 -0.64124200

C -2.44078300 2.58646600 -0.78025300

C -5.29622300 0.18359200 -1.53852300

H -5.10990600 -0.70523500 -2.14436300

H -5.87946800 -0.12654700 -0.66537500

H -5.89249600 0.89046600 -2.12732100

H -4.54782400 2.80791300 -1.33337000

C -4.63037200 2.38679200 0.75693500

H -4.24942400 3.34611800 1.12255700

C -4.26287000 1.31426900 1.78573900

H -4.59055600 0.31809100 1.46875800

C -2.00904500 3.64442500 0.24770300

C -1.27574800 3.31958800 1.39485400

C -2.37251500 4.98479700 0.03765000

C -0.92015400 4.31514600 2.31011600

C -2.02688700 5.97260300 0.95722400

C -1.29508900 5.64082100 2.09995400

H -0.96546500 2.29924300 1.58422900

H -2.92254700 5.26053500 -0.85789500

H -0.34694100 4.04083200 3.19140300

H -2.32142300 7.00288700 0.77536600

H -1.01681500 6.41050800 2.81484700

C -2.06282300 3.10370200 -2.19908600

C -0.69080500 3.21768000 -2.48944300

C -2.97408800 3.47751500 -3.19326000

C -0.24999700 3.68200200 -3.72543700

C -2.53321000 3.94185200 -4.43892900

C -1.17233800 4.04642200 -4.71091500

H 0.04467400 2.94704500 -1.73695500

H -4.04419300 3.42046900 -3.02326000

H 0.81748300 3.76664500 -3.90989800

H -3.26518000 4.22400300 -5.19150400

H -0.83135100 4.41104300 -5.67617000

N -2.10383500 -1.30426500 -0.23449200

C -2.17475200 -2.58236300 -0.99021000

C -1.73598400 -2.10160100 -2.44192000

N -2.41117700 -0.78744500 -2.56446100

H -2.04267900 -1.29669000 0.80384300

C -2.91402200 -0.33561900 -3.85507200

H -3.42216700 0.62184400 -3.73337700

H -2.10557300 -0.19710900 -4.57742400

H -3.62708900 -1.06544000 -4.26199300

H -2.18158100 -2.75284800 -3.19889000

C -0.19547300 -2.05777600 -2.69798400

H 0.30215200 -1.85895600 -1.74519400

C 0.25493500 -0.93838500 -3.65148100

H -0.07403000 -1.11912900 -4.68338500

C -1.23505000 -3.62200400 -0.35752900

C -0.09359200 -3.23051300 0.35296500

C -1.49034000 -4.99431700 -0.50121300

C 0.77633000 -4.17939200 0.89056300

C -0.62213900 -5.94413800 0.03718500

C 0.51653700 -5.54050500 0.73559300

H 0.12105800 -2.17789500 0.48727700

H -2.37735000 -5.32873600 -1.02911700

H 1.65666000 -3.84285100 1.43020500

H -0.84372200 -7.00121600 -0.08451200

H 1.18758000 -6.28130600 1.16282600

C -3.63311200 -3.11057900 -0.94862400

C -4.31951200 -3.63213800 -2.05208700

C -4.29219600 -3.09642300 0.29383100

C -5.63360000 -4.09890700 -1.92943700

C -5.59814100 -3.56606400 0.41736800

C -6.28000100 -4.06408600 -0.69644000

H -3.84479900 -3.69239000 -3.02616300

H -3.77168800 -2.72070400 1.17092200

H -6.14415400 -4.49290200 -2.80452700

H -6.08291100 -3.54765500 1.39001900

H -7.29945900 -4.42779600 -0.60000300

C 4.19837100 0.25462900 3.33321900

C 5.26632700 -0.63197100 3.10931000

C 4.38725400 1.28374700 4.27244000

C 6.46695400 -0.50550100 3.80577500

H 5.14974800 -1.41572900 2.36801800

C 5.59020000 1.41455400 4.96558600

H 3.58928000 1.99209100 4.47116000

C 6.63606800 0.51814200 4.73964300

H 7.27471900 -1.20816100 3.61575100

H 5.70754200 2.21864200 5.68787200

H 7.57155500 0.61772000 5.28409100

C 0.30536200 -3.40874200 -3.23736400

H -0.12440600 -3.60961700 -4.22901800

H 1.39536100 -3.39091200 -3.34649200

H 0.05043800 -4.24542700 -2.58306900

H -0.10357300 0.04615000 -3.33844200

H -3.18672400 1.26996000 1.96553800

C -6.15915700 2.55573700 0.65288200

H -6.67109900 1.62817500 0.37712000

H -6.56217200 2.86891200 1.62244100

H -6.43425700 3.32383600 -0.08132200

H -4.74915700 1.52370700 2.74413100

H 1.34855900 -0.89485000 -3.65242500

- **1ba**-*RR* (MeH-2)


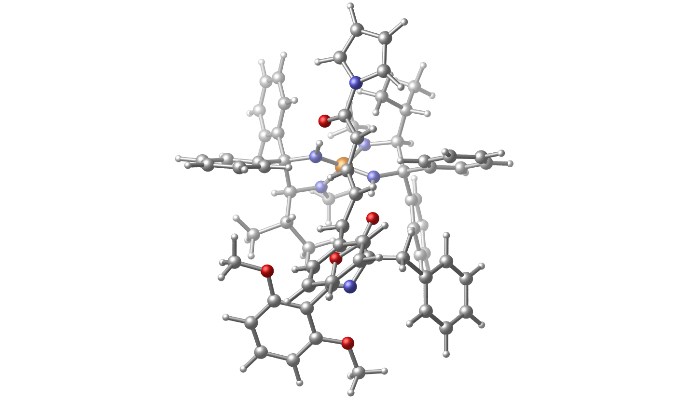

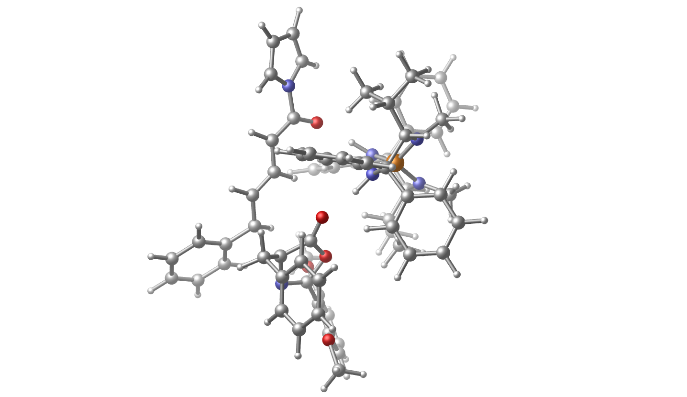


B3LYP/6-31G*

E = –3719.222716

Zero-point correction = 1.283823 (Hartree/Particle)

Thermal correction to Energy = 1.359725

Thermal correction to Enthalpy = 1.360669

Thermal correction to Gibbs Free Energy = 1.164092

Sum of electronic and zero-point Energies = –3717.938893

Sum of electronic and thermal Energies = –3717.862991

Sum of electronic and thermal Enthalpies = –3717.862047

Sum of electronic and thermal Free Energies = –3718.058624

PCM(toluene)-B3LYP-D3/6-311++G**//B3LYP/6-31G*@–30 °C

E = –3720.389102

G = –3719.191155

0 1

O 1.03821900 1.05885200 0.23998300

C 2.14946400 0.54028500 0.20666500

C 3.35113700 0.76836500 0.98657500

O 2.42844300 -0.54318900 -0.62238400

N 4.37076500 -0.02814200 0.40800100

C 3.79111600 -0.78076500 -0.45453000

C 4.39564200 -1.83183900 -1.29469200

C 4.11966600 -3.18982300 -1.05041100

C 5.27346900 -1.48740200 -2.34085700

C 4.71120300 -4.19138200 -1.83132800

C 5.87076200 -2.48506300 -3.12435800

C 5.57973000 -3.82053600 -2.85643900

O 3.26947700 -3.43552000 -0.01182500

C 2.96645200 -4.78667600 0.31297600

H 3.86509200 -5.33199000 0.62828600

H 2.50009000 -5.30746300 -0.53197100

H 2.25345300 -4.74027200 1.13646700

O 5.46905100 -0.15605300 -2.52547100

C 6.34809900 0.27298700 -3.55233200

H 7.36897900 -0.09607800 -3.38917400

H 6.00057300 -0.04851700 -4.54315000

H 6.34313700 1.36300600 -3.50178100

H 4.51251400 -5.23898500 -1.64260200

H 6.54712300 -2.23002700 -3.93087800

H 6.04292500 -4.59483400 -3.46236700

C 3.69491800 2.16172900 1.46433200

H 2.85025400 2.53699000 2.05306700

H 4.56174800 2.08921600 2.13006000

C 3.99361000 3.12160100 0.32490400

C 3.03447600 4.04973100 -0.10197500

C 5.22761400 3.07927600 -0.34086600

C 3.29885500 4.91481700 -1.16703700

C 5.49560100 3.94558200 -1.40097400

C 4.53156600 4.86663500 -1.82004600

H 2.07329700 4.09400900 0.40443300

H 5.97232200 2.35254100 -0.02810800

H 2.54141700 5.62945900 -1.47952600

H 6.46349700 3.90808500 -1.89629200

H 4.74294400 5.54511800 -2.64297400

C 2.79851600 -0.29302400 2.67129100

C 1.68947100 0.30044500 3.35218900

H 2.55078500 -1.18131900 2.09008700

H 1.89060200 0.98114200 4.17696400

C 0.36738700 0.00412200 3.06573600

H 0.17710200 -0.67634700 2.23797900

C -0.75819900 0.45052000 3.77580500

H -0.60852400 1.09367000 4.63666600

C -2.05917400 -0.00580200 3.48527500

O -2.39663400 -0.82924300 2.59372900

N -3.12870700 0.49666900 4.30854800

C -4.36357200 -0.11972000 4.40222800

C -3.14808800 1.63842200 5.09548400

C -5.15671100 0.61726700 5.24751200

C -4.38562100 1.74006800 5.68241200

H -4.53815700 -1.04096300 3.87085400

H -2.29873600 2.30169600 5.13280000

H -6.17306400 0.37774900 5.53200700

H -4.70679400 2.53396100 6.34431100

H -0.62273300 1.08717500 -0.45485400

N -1.60612400 1.22867700 -0.72246500

P -2.53247200 -0.01509000 -1.24793600

N -3.83286700 0.95426400 -1.60981100

C -3.66666800 2.36298400 -1.17949900

C -2.09107700 2.56307300 -1.14158900

C -5.11813500 0.47354700 -2.10414400

H -4.98890900 -0.49725500 -2.58587500

H -5.85369700 0.35217200 -1.30253200

H -5.51435200 1.18105700 -2.84216400

H -4.05967700 2.98950800 -1.98530000

C -4.47036700 2.75399400 0.09971200

H -3.97106800 3.63713800 0.50962500

C -4.47937300 1.67785500 1.19033600

H -5.01081700 0.77532200 0.86645900

C -1.66283500 3.64670700 -0.14118200

C -1.16986600 3.33492700 1.13069400

C -1.79232300 4.99773700 -0.50166400

C -0.81745400 4.35358600 2.02072100

C -1.45062100 6.01145400 0.39131200

C -0.95830600 5.69247400 1.65917800

H -1.04410200 2.30396500 1.43799000

H -2.15353200 5.25933000 -1.49250800

H -0.43133300 4.08608000 3.00045300

H -1.56020200 7.05059900 0.09213200

H -0.68323200 6.48134700 2.35413400

C -1.47790000 2.89692500 -2.53182700

C -0.07392300 2.92695700 -2.61710500

C -2.21008600 3.15856900 -3.69539000

C 0.57027400 3.20771300 -3.81857600

C -1.56420500 3.43952400 -4.90588700

C -0.17423500 3.46614500 -4.97336500

H 0.52348100 2.73410600 -1.73063700

H -3.29489000 3.15079900 -3.68847000

H 1.65618900 3.23304800 -3.84525600

H -2.15939700 3.64061500 -5.79311100

H 0.32598800 3.68865500 -5.91210700

N -2.38514800 -1.23557500 -0.15958900

C -2.59319400 -2.57558500 -0.76077500

C -1.86808800 -2.37811600 -2.15824300

N -2.30799000 -1.01561200 -2.55137000

H -2.40336900 -1.06485000 0.86153300

C -2.52737200 -0.67805700 -3.95192300

H -2.92538200 0.33522400 -4.02357800

H -1.59976700 -0.71668400 -4.52879300

H -3.25016400 -1.37302400 -4.39971600

H -2.28124900 -3.06603700 -2.90162000

C -0.31603300 -2.56846800 -2.13284500

H 0.04016300 -2.31918900 -1.12668300

C 0.43878400 -1.64919900 -3.10779100

H 0.21983400 -1.90053000 -4.15368300

C -2.00053300 -3.69112700 0.11667900

C -1.13329000 -3.42609300 1.18164000

C -2.35471300 -5.02765100 -0.13586700

C -0.63082300 -4.46672500 1.96880700

C -1.84065700 -6.06609100 0.63737000

C -0.97444300 -5.78964800 1.69804800

H -0.87894500 -2.40726200 1.43905200

H -3.04816400 -5.26138600 -0.93788200

H 0.01744900 -4.22802100 2.80782300

H -2.13082900 -7.09069500 0.41991100

H -0.58864600 -6.59640400 2.31570000

C -4.11877700 -2.83381500 -0.90205300

C -4.72138800 -3.41171500 -2.02625300

C -4.93902800 -2.50474800 0.19094000

C -6.10210700 -3.63580000 -2.06874800

C -6.31313200 -2.73228100 0.15236800

C -6.90409200 -3.29558700 -0.98187400

H -4.12842000 -3.70252800 -2.88753000

H -4.49384200 -2.06522000 1.07924300

H -6.54401200 -4.08048600 -2.95677100

H -6.92294500 -2.47074900 1.01307400

H -7.97590600 -3.47128600 -1.01371900

C 4.09951300 -0.44218000 3.38303100

C 4.90876300 -1.55816100 3.10886400

C 4.55103100 0.47647800 4.34708800

C 6.11477900 -1.75630300 3.77768300

H 4.58147300 -2.27206800 2.35873900

C 5.76096100 0.28285500 5.01246500

H 3.95378100 1.35204100 4.58249400

C 6.54812000 -0.83600100 4.73376000

H 6.71896200 -2.63094500 3.54976700

H 6.08707300 1.00769600 5.75421600

H 7.48881800 -0.98837500 5.25654300

C 0.06624700 -4.02675300 -2.44175400

H -0.25480700 -4.30335000 -3.45590000

H 1.15513600 -4.14128900 -2.40256400

H -0.37411600 -4.73950100 -1.74177900

H 0.21497600 -0.59130800 -2.94683900

H -3.47247500 1.37944200 1.48571600

C -5.90262600 3.20388700 -0.24908800

H -6.52183000 2.38665900 -0.63454400

H -6.39934200 3.58966400 0.64818000

H -5.90303300 4.00708000 -0.99689100

H -4.98509900 2.04829200 2.08801200

H 1.51437700 -1.77544000 -2.95583600

- **1ba**-*SR* (HH)


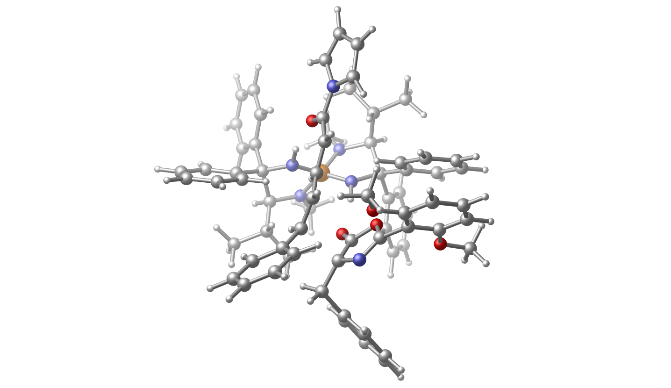

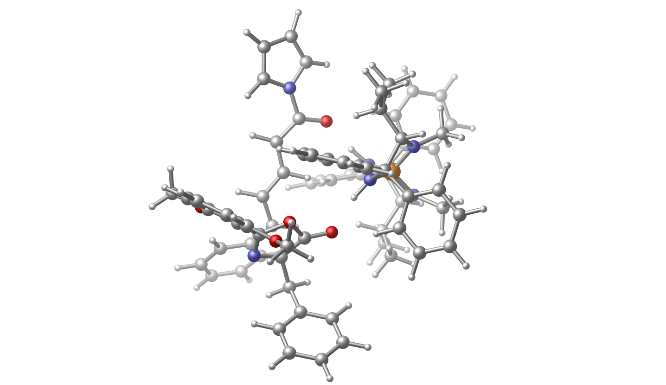


B3LYP/6-31G*

E = –3719.223401

Zero-point correction = 1.284334 (Hartree/Particle)

Thermal correction to Energy = 1.359988

Thermal correction to Enthalpy = 1.360933

Thermal correction to Gibbs Free Energy = 1.165995

Sum of electronic and zero-point Energies = –3717.939067

Sum of electronic and thermal Energies = –3717.863413

Sum of electronic and thermal Enthalpies = –3717.862468

Sum of electronic and thermal Free Energies = –3718.057406

PCM(toluene)-B3LYP-D3/6-311++G**//B3LYP/6-31G*@–30 °C

E = –3720.390595

G = –3719.191043

0 1

O -0.95792400 0.32705600 -1.38554800

C -2.08418800 0.40644100 -0.87822300

C -3.03942600 1.49089700 -0.79427000

O -2.59454200 -0.60736800 -0.10328200

N -4.17578600 1.00095800 -0.12097300

C -3.85520500 -0.16971400 0.30569800

C -4.66871800 -1.12104700 1.07942000

C -4.96447000 -2.38915700 0.53142400

C -5.19119500 -0.79319600 2.34770400

C -5.73518400 -3.31763600 1.24224400

C -5.97026300 -1.71817300 3.05741100

C -6.22663600 -2.96695000 2.49738600

O -4.47900200 -2.60748600 -0.71804500

C -4.74946000 -3.84636400 -1.35353500

H -5.82851900 -4.00881900 -1.47548200

H -4.31023000 -4.68491000 -0.79981300

H -4.27965500 -3.78025900 -2.33609400

O -4.87909500 0.44231200 2.83104600

C -5.36588000 0.81462900 4.11138600

H -4.98728800 0.14836300 4.89733000

H -4.99424000 1.82586200 4.28528200

H -6.46303600 0.82160400 4.13919000

H -5.96297200 -4.28953900 0.82233600

H -6.36917300 -1.47524900 4.03409900

H -6.82938300 -3.68217100 3.05094500

C -3.21646400 2.47481300 -1.92884700

H -2.24155400 2.91319100 -2.17306300

H -3.86007700 3.28908100 -1.58244900

C -3.81204600 1.84346400 -3.17740500

C -3.00359300 1.50580200 -4.27010000

C -5.18559900 1.57107100 -3.25271000

C -3.55075700 0.91883100 -5.41301000

C -5.73490900 0.98383800 -4.39220700

C -4.91980400 0.65713800 -5.47888400

H -1.93502700 1.70150500 -4.22048400

H -5.81940800 1.81319000 -2.40386500

H -2.90704400 0.67094600 -6.25403500

H -6.80275800 0.78288300 -4.43330100

H -5.34917400 0.20410400 -6.36902500

C -1.98213700 2.55949300 0.71809800

C -1.77375300 1.78116200 1.89247900

H -1.10833300 2.66679800 0.07444900

H -2.50805300 1.84501000 2.69060900

C -0.62459100 1.02613900 2.09587400

H 0.08879500 0.99297400 1.27395700

C -0.26053800 0.33501500 3.25469000

H -0.95791400 0.29832900 4.08442200

C 1.03104700 -0.21861800 3.42387200

O 2.01396000 -0.11439300 2.64548400

N 1.26620400 -0.96585000 4.62610500

C 2.53339100 -1.26690900 5.09674300

C 0.33389100 -1.52000300 5.49270800

C 2.40692700 -1.99476800 6.25359600

C 1.00801800 -2.16080000 6.50172900

H 3.39694100 -0.90958200 4.56058400

H -0.72275700 -1.44797600 5.29042200

H 3.22242200 -2.36456000 6.86127300

H 0.55208200 -2.69839000 7.32297600

H 0.38798600 -0.79508100 -0.89258000

N 1.28266200 -1.29098200 -0.75213700

P 2.70883900 -0.49158800 -0.89631000

N 3.62103900 -1.87039500 -0.79022000

C 2.82402200 -3.05505400 -0.38060700

C 1.39906600 -2.74757500 -1.01715500

C 5.04439700 -1.99438100 -1.08090500

H 5.20441100 -2.78034500 -1.83070800

H 5.62548200 -2.23659500 -0.18786700

H 5.42226300 -1.05134600 -1.47826200

H 3.27499100 -3.91743900 -0.87888700

C 2.82822800 -3.33892700 1.15566600

H 2.01477900 -2.76318000 1.60709700

C 4.11436900 -2.89422800 1.86989500

H 4.32404700 -1.83279700 1.72082500

C 0.23774900 -3.49975300 -0.34579300

C -0.14515600 -4.76904900 -0.80777300

C -0.43912500 -2.95859500 0.75445400

C -1.15281700 -5.48922900 -0.16697700

C -1.45427900 -3.67569000 1.38955100

C -1.80984200 -4.94609900 0.93935500

H 0.34816200 -5.20310300 -1.67141500

H -0.18366900 -1.97162900 1.12131300

H -1.42387400 -6.47513400 -0.53644600

H -1.96885000 -3.22856800 2.23467700

H -2.59633700 -5.50427600 1.44062300

C 1.36553200 -3.00076100 -2.54946800

C 0.41578900 -2.30093600 -3.31518000

C 2.19087600 -3.91900600 -3.21232600

C 0.31114100 -2.50039900 -4.69055600

C 2.08979200 -4.11690500 -4.59396400

C 1.15206400 -3.40701100 -5.34024500

H -0.25182700 -1.59068900 -2.83598200

H 2.92161000 -4.50638400 -2.66588800

H -0.43537100 -1.94542100 -5.25260800

H 2.74758400 -4.83321900 -5.07967600

H 1.07229700 -3.56146300 -6.41294700

N 2.70205900 0.79811500 0.12515800

C 3.60341100 1.88416900 -0.32982500

C 3.30828500 1.85667100 -1.89243500

N 3.25301900 0.40028900 -2.18383600

H 2.44220800 0.64555400 1.11644600

C 3.68722000 -0.12766900 -3.47194300

H 3.03804700 0.20104800 -4.28714800

H 3.66255800 -1.21870000 -3.44712600

H 4.71445500 0.19552800 -3.68653800

H 4.16400000 2.25272100 -2.44703000

C 2.03968100 2.65589100 -2.33075100

H 1.33243200 2.63761600 -1.49419200

C 1.30269900 2.05194900 -3.53705700

H 0.98238200 1.02317200 -3.35877800

C 3.25933500 3.21212200 0.36664700

C 4.19630200 4.25821600 0.39442100

C 2.02116000 3.41990500 0.98533400

C 3.89271500 5.47685600 0.99947100

C 1.71776000 4.63760800 1.59910200

C 2.64979500 5.67350300 1.60490900

H 5.17506000 4.12168000 -0.05388500

H 1.28511900 2.62810400 1.00540100

H 4.63540300 6.27038800 1.00513600

H 0.74857600 4.76343200 2.07362500

H 2.41649100 6.62048600 2.08399000

C 5.07558500 1.51913000 0.00533100

C 6.15703600 1.75516900 -0.85334700

C 5.34656900 0.96751700 1.26913600

C 7.46441100 1.43295800 -0.47326800

C 6.64889800 0.65027800 1.65120500

C 7.71641200 0.87741900 0.77912000

H 6.00389800 2.20320200 -1.82988600

H 4.53052600 0.78310000 1.96197000

H 8.28320200 1.62303000 -1.16245300

H 6.82852500 0.22607100 2.63542700

H 8.73163000 0.62858000 1.07583600

C -2.80528300 3.79202700 0.81868400

C -2.30822900 5.00267700 0.30757500

C -4.06575600 3.79810900 1.44388900

C -3.03254200 6.18873400 0.43489900

H -1.33552400 5.01456400 -0.17927500

C -4.79044300 4.98200200 1.56288600

H -4.48615400 2.86023500 1.79190400

C -4.27705400 6.18355900 1.06550800

H -2.62385800 7.11514100 0.03898800

H -5.76747700 4.96704100 2.03990200

H -4.84617800 7.10448200 1.16248500

H 4.98612600 -3.47951200 1.54901300

C 2.60316800 -4.83480000 1.43873000

H 2.55798100 -5.00768400 2.51950800

H 1.67996700 -5.21962000 1.00220200

H 3.43946500 -5.42969600 1.04487700

C 2.39633500 4.11837700 -2.65231000

H 3.06797200 4.16676100 -3.52081200

H 1.49061100 4.67902300 -2.90954300

H 2.88091400 4.63393600 -1.82109300

H 1.91292300 2.08764000 -4.44847500

H 0.40034100 2.64203500 -3.73257300

H 3.99050000 -3.05221700 2.94638500

- **1ba**-*SR* (MeMe)


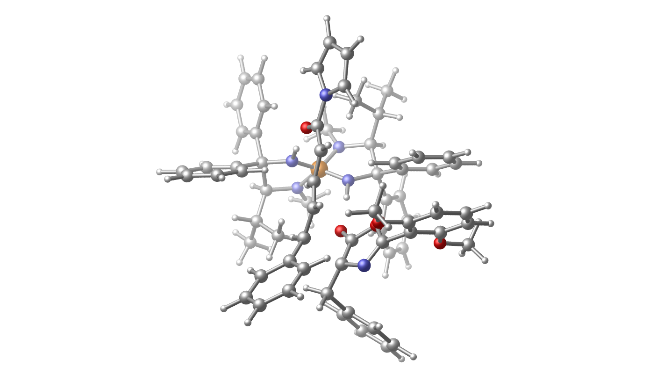

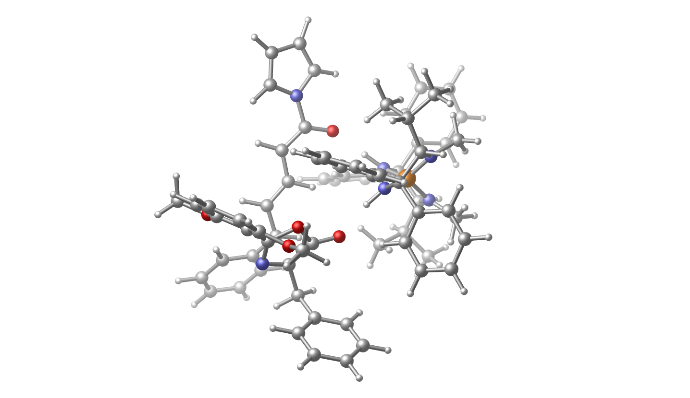


B3LYP/6-31G*

E = –3719.223026

Zero-point correction = 1.283438 (Hartree/Particle)

Thermal correction to Energy = 1.359566

Thermal correction to Enthalpy = 1.360510

Thermal correction to Gibbs Free Energy = 1.163052

Sum of electronic and zero-point Energies = –3717.939588

Sum of electronic and thermal Energies = –3717.863460

Sum of electronic and thermal Enthalpies = –3717.862516

Sum of electronic and thermal Free Energies = –3718.059974

PCM(toluene)-B3LYP-D3/6-311++G**//B3LYP/6-31G*@–30 °C

E = –3720.387469

G = –3719.190401

0 1

O -0.97715000 0.02719800 -1.25931700

C -2.13383200 0.19671300 -0.85751800

C -3.14456600 1.17734500 -1.19664800

O -2.64120400 -0.52417700 0.19607400

N -4.29787800 0.86515300 -0.44579000

C -3.94639300 -0.06403600 0.37124400

C -4.75786400 -0.75457600 1.38714500

C -4.98448800 -2.14309200 1.26458000

C -5.33830900 -0.06619800 2.47152300

C -5.75128300 -2.83252600 2.21220500

C -6.11173400 -0.75289200 3.41843700

C -6.30351600 -2.12479400 3.27717700

O -4.43626500 -2.72349600 0.16569100

C -4.63225400 -4.11235900 -0.04723200

H -5.69785300 -4.35652800 -0.15043300

H -4.19429900 -4.70748600 0.76307300

H -4.11499600 -4.34152400 -0.98015100

O -5.08663600 1.27155200 2.54424100

C -5.62299100 2.00523100 3.63480700

H -5.24584100 1.63300400 4.59611000

H -5.29019900 3.03437300 3.48990100

H -6.72013800 1.97600600 3.63997400

H -5.92767500 -3.89685700 2.11797700

H -6.55575400 -0.23166600 4.25702400

H -6.90257000 -2.65443800 4.01331700

C -3.30019200 1.68671600 -2.61296200

H -2.36558200 2.16501200 -2.92842500

H -4.07333900 2.46155800 -2.60985600

C -3.66056000 0.59768200 -3.61118600

C -2.73095700 0.15992000 -4.56287700

C -4.93048600 0.00169400 -3.59488700

C -3.05924700 -0.84150100 -5.48050800

C -5.25981800 -1.00073800 -4.50667800

C -4.32607000 -1.42646100 -5.45503500

H -1.74196500 0.61197600 -4.58711200

H -5.65509800 0.32382800 -2.85188100

H -2.32742800 -1.15586800 -6.22151200

H -6.25021800 -1.44876100 -4.47969400

H -4.58605200 -2.20312400 -6.16992400

C -2.25326500 2.78228900 -0.14033000

C -2.00690000 2.46422700 1.22640400

H -1.37097000 2.75360600 -0.78067400

H -2.76472600 2.71722100 1.96294600

C -0.81111400 1.90233700 1.65378400

H -0.07845100 1.66703600 0.88279900

C -0.42672400 1.62943900 2.96989100

H -1.12991900 1.82358800 3.77194600

C 0.87708400 1.18712400 3.28884100

O 1.84062100 1.03520000 2.49316300

N 1.15294200 0.89170400 4.66627200

C 2.43478800 0.73412000 5.16589600

C 0.25235100 0.69765000 5.70559000

C 2.34987300 0.45299100 6.50634500

C 0.96126800 0.42459500 6.84816300

H 3.27958700 0.85663300 4.50874900

H -0.81147600 0.73055500 5.53436700

H 3.18629900 0.28940300 7.17320000

H 0.53438300 0.21796000 7.82104000

H 0.46327700 -0.91174800 -0.63747100

N 1.33310200 -1.42653000 -0.43006400

P 2.80825100 -0.79154600 -0.75149200

N 3.62424900 -2.17244000 -0.32598000

C 2.77240000 -3.22683200 0.26260800

C 1.31506300 -2.90137300 -0.29620500

C 5.07014600 -2.34427500 -0.39804600

H 5.29989200 -3.35697100 -0.74988300

H 5.55882300 -2.18802800 0.56955700

H 5.49051900 -1.62547700 -1.10378700

H 3.09621600 -4.16491700 -0.19318000

C 2.93533400 -3.41545100 1.80326300

H 2.01426400 -3.89457600 2.14802100

C 3.10582300 -2.10929100 2.58704800

H 2.30140500 -1.39322500 2.40825900

C 0.20793300 -3.33680300 0.67997400

C -0.19609200 -4.68049200 0.72600800

C -0.37496800 -2.43316400 1.57583600

C -1.13769000 -5.11131600 1.65958400

C -1.33039800 -2.86177800 2.49893300

C -1.70983800 -4.20170300 2.55233700

H 0.22399200 -5.39774100 0.02774400

H -0.09198300 -1.38790100 1.56044300

H -1.42631100 -6.15930300 1.68435800

H -1.77600400 -2.13554300 3.17189900

H -2.44812400 -4.53337900 3.27765800

C 1.05662000 -3.54517900 -1.69068200

C 0.10436100 -2.94330200 -2.53249900

C 1.67078100 -4.72241500 -2.14106600

C -0.20493800 -3.48651900 -3.77836600

C 1.36520300 -5.26635400 -3.39314400

C 0.42866900 -4.65026100 -4.21953900

H -0.40976800 -2.04068800 -2.21799200

H 2.39135200 -5.25025600 -1.52425300

H -0.95180400 -2.99565400 -4.39701400

H 1.86390100 -6.17753100 -3.71382600

H 0.19003200 -5.07389200 -5.19149000

N 2.87478900 0.70779700 -0.08698300

C 3.86451300 1.59076600 -0.73938800

C 3.74750300 1.15522500 -2.26501900

N 3.46640300 -0.29731600 -2.19352700

H 2.50471700 0.87940300 0.86116400

C 3.70028500 -1.17777600 -3.33197400

H 2.90273900 -1.11457700 -4.07994400

H 3.75259800 -2.21287700 -2.98618000

H 4.65258800 -0.92043400 -3.81018900

H 4.73495300 1.25435700 -2.72423500

C 2.77204300 1.97583700 -3.15869600

H 2.95296400 3.02413500 -2.89803500

C 1.28998700 1.67855900 -2.91359400

H 0.99578800 1.86145400 -1.87882900

C 3.55096000 3.07821000 -0.50812800

C 4.44450500 4.04842000 -0.99272800

C 2.41238900 3.50842900 0.17999000

C 4.19208700 5.40665000 -0.81630600

C 2.16406400 4.87169400 0.36587200

C 3.04636100 5.82569700 -0.13502800

H 5.35141800 3.74144000 -1.50589200

H 1.71915100 2.79197400 0.59949100

H 4.89655400 6.13788600 -1.20402400

H 1.27392700 5.17622700 0.90917500

H 2.85147700 6.88483900 0.00977100

C 5.27521700 1.30860800 -0.14095900

C 6.46123600 1.28244600 -0.88584200

C 5.36916200 1.12504200 1.24971500

C 7.69692300 1.05730400 -0.26990400

C 6.59984000 0.90549300 1.86537700

C 7.77257200 0.86441400 1.10723600

H 6.45117900 1.44262400 -1.95948400

H 4.47014500 1.15690800 1.85821600

H 8.59903800 1.03624700 -0.87594200

H 6.64114800 0.77028800 2.94292900

H 8.73219100 0.69090400 1.58645900

C -3.18602700 3.89326000 -0.46207100

C -2.78999600 4.89112200 -1.36813700

C -4.45372100 4.00253600 0.13812500

C -3.62000200 5.97593400 -1.65303300

H -1.81269700 4.82219100 -1.84069000

C -5.28414200 5.08251600 -0.15313500

H -4.79468300 3.21283800 0.79920700

C -4.87140200 6.07716500 -1.04460500

H -3.28787000 6.74029500 -2.35121700

H -6.26465600 5.14571800 0.31268000

H -5.52265500 6.91855100 -1.26688900

H 4.05058200 -1.61098600 2.33807800

C 4.07363300 -4.40277300 2.12773400

H 5.06190600 -4.00757400 1.86752400

H 4.08517400 -4.61880300 3.20202500

H 3.94419800 -5.35746800 1.60201500

C 3.11880400 1.84367800 -4.65444700

H 2.87186200 0.85606200 -5.05765200

H 2.54798700 2.57842100 -5.23346700

H 4.18334500 2.03060900 -4.84507100

H 1.03624400 0.63853300 -3.14406700

H 3.12117300 -2.31700500 3.66271600

H 0.67016100 2.31646100 -3.55499700

- **1ba**-*SR* (HMe)

B3LYP/6-31G*

E = –3719.229538

Zero-point correction = 1.283668 (Hartree/Particle)

Thermal correction to Energy = 1.359715

Thermal correction to Enthalpy = 1.360659

Thermal correction to Gibbs Free Energy = 1.163838

Sum of electronic and zero-point Energies = –3717.939698

Sum of electronic and thermal Energies = –3717.863651

Sum of electronic and thermal Enthalpies = –3717.862707

Sum of electronic and thermal Free Energies = –3718.059528

PCM(toluene)-B3LYP-D3/6-311++G**//B3LYP/6-31G*@–30 °C

E = –3720.38958

G = –3719.191841

0 1

O -1.02958000 0.21975000 -1.34562900

C -2.15836000 0.35259000 -0.85824000

C -3.08445000 1.46472800 -0.84216100

O -2.71097300 -0.61040000 -0.04601900

N -4.24395100 1.03930700 -0.16510500

C -3.96394700 -0.12008200 0.31843600

C -4.81699300 -1.01239600 1.12029700

C -5.15619400 -2.28778300 0.61669100

C -5.33319900 -0.62027600 2.37240100

C -5.96600900 -3.16020800 1.35453400

C -6.15225700 -1.48801200 3.10881500

C -6.45276000 -2.74548300 2.59197200

O -4.66923100 -2.57024500 -0.61953300

C -4.98105400 -3.82024800 -1.21208100

H -6.06447000 -3.94739200 -1.33713100

H -4.57803300 -4.65433000 -0.62465900

H -4.50158800 -3.80777400 -2.19207400

O -4.97423000 0.61833500 2.81396000

C -5.45430000 1.05578800 4.07616600

H -5.10901100 0.40318000 4.88843500

H -5.04271300 2.05661800 4.21727200

H -6.55040400 1.10852200 4.09430100

H -6.22730200 -4.13766000 0.96835200

H -6.54808900 -1.19504700 4.07300400

H -7.08617000 -3.41675000 3.16594200

C -3.21260800 2.40400400 -2.01993700

H -2.22726600 2.82766200 -2.24681500

H -3.86249200 3.23448200 -1.72710800

C -3.76564900 1.73075900 -3.26607400

C -2.92466900 1.39077600 -4.33319300

C -5.13048200 1.42429100 -3.36663600

C -3.43151300 0.76768900 -5.47572200

C -5.63938200 0.80106400 -4.50573000

C -4.79207600 0.47176700 -5.56680700

H -1.86289100 1.61614400 -4.26478200

H -5.78902500 1.66880200 -2.53749300

H -2.76309800 0.51857900 -6.29694900

H -6.70106800 0.57409800 -4.56684400

H -5.19013800 -0.00956200 -6.45666200

C -2.01451300 2.59429500 0.63958000

C -1.80349900 1.85656300 1.83738500

H -1.14455100 2.67349900 -0.01260700

H -2.53783500 1.94047500 2.63385400

C -0.65540100 1.10493600 2.05878700

H 0.06052200 1.05386800 1.23973900

C -0.29852600 0.43458400 3.23185900

H -0.99841200 0.41877400 4.06004900

C 0.98421700 -0.13423300 3.41064600

O 1.96577700 -0.06177900 2.62586200

N 1.21076500 -0.86122100 4.62651800

C 2.47357700 -1.17524700 5.10068800

C 0.27100100 -1.38316300 5.50530400

C 2.33714200 -1.87864100 6.27141500

C 0.93615800 -2.01599400 6.52508700

H 3.34279300 -0.84429300 4.55692300

H -0.78468100 -1.29774700 5.30342300

H 3.14748300 -2.25070900 6.88456000

H 0.47277100 -2.53024200 7.35701400

H 0.43547000 -0.77413600 -0.91195300

N 1.32849300 -1.27691900 -0.79311300

P 2.78429000 -0.52792700 -0.89070000

N 3.64177300 -1.93657600 -0.72298000

C 2.79576500 -3.08663200 -0.31777700

C 1.40131000 -2.74700800 -1.00399100

C 5.06259000 -2.11621600 -0.99494700

H 5.20436400 -2.89254800 -1.75867300

H 5.61623800 -2.40216100 -0.09737100

H 5.48417500 -1.18033000 -1.36511100

H 3.23154400 -3.97380100 -0.78536600

C 2.75194400 -3.33965300 1.22302000

H 1.96038300 -2.71388100 1.64611600

C 4.04604700 -2.94552100 1.95311600

H 4.31668400 -1.90100700 1.78413800

C 0.19835400 -3.43715700 -0.34155900

C -0.22653700 -4.69783500 -0.79070500

C -0.47145500 -2.85451700 0.74125700

C -1.26907600 -5.36966200 -0.15349400

C -1.52133700 -3.52293500 1.37244400

C -1.91884400 -4.78542200 0.93603500

H 0.26223400 -5.16337000 -1.64055700

H -0.18498900 -1.87250800 1.09844200

H -1.57293300 -6.34976500 -0.51266500

H -2.03029600 -3.04245600 2.20237900

H -2.73221800 -5.30576800 1.43522300

C 1.39251900 -3.03899100 -2.52906300

C 0.44109200 -2.36675800 -3.31769100

C 2.24255300 -3.95278600 -3.16496100

C 0.35826900 -2.58930500 -4.69049000

C 2.16355300 -4.17345200 -4.54504900

C 1.22410800 -3.49141800 -5.31438000

H -0.24415000 -1.66181300 -2.85519800

H 2.97702300 -4.51707400 -2.59983100

H -0.39037100 -2.05698800 -5.27138400

H 2.83986300 -4.88563500 -5.01099800

H 1.16132800 -3.66409100 -6.38545000

N 2.76509400 0.78120600 0.10400900

C 3.74560400 1.81523600 -0.29062400

C 3.68281200 1.74413200 -1.87973300

N 3.45728500 0.30693600 -2.15850200

H 2.46150700 0.64236800 1.08400600

C 3.69826600 -0.25201400 -3.48331800

H 2.92241000 0.03513800 -4.20126500

H 3.70910900 -1.34248600 -3.42514200

H 4.67044500 0.08895200 -3.85915200

H 4.67724400 1.98017200 -2.26704700

C 2.69739900 2.71833400 -2.59486200

H 2.73544000 3.65167600 -2.02396700

C 1.24198000 2.24097700 -2.61372000

H 0.85430400 2.03650900 -1.61465000

C 3.35589900 3.19573500 0.26512000

C 4.26184800 4.26530700 0.17813100

C 2.10770500 3.43137500 0.85115700

C 3.92024800 5.53247000 0.64714600

C 1.76767300 4.69872800 1.33142700

C 2.66974900 5.75567400 1.22790300

H 5.24799000 4.10851800 -0.24807800

H 1.39112400 2.62692900 0.94577500

H 4.63860200 6.34421100 0.56724900

H 0.79256200 4.84775200 1.78694600

H 2.40684300 6.74150700 1.60169200

C 5.15285300 1.44477300 0.26474100

C 6.35322000 1.65276100 -0.42845500

C 5.23258800 0.92737100 1.56961600

C 7.58760200 1.33614400 0.14834800

C 6.46240900 0.61518100 2.14683300

C 7.64888000 0.81331500 1.43752900

H 6.35543400 2.07343600 -1.42917100

H 4.32653500 0.76021900 2.14459800

H 8.49966200 1.50441700 -0.41853200

H 6.49053100 0.21716500 3.15765700

H 8.60713400 0.56772100 1.88700100

C -2.83688300 3.82922900 0.69077300

C -2.34213800 5.01518500 0.12211500

C -4.09224800 3.86572400 1.32502800

C -3.06315000 6.20723400 0.20295000

H -1.37401700 5.00289300 -0.37379200

C -4.81353800 5.05533500 1.39795900

H -4.51191000 2.94521900 1.71681100

C -4.30205500 6.23271800 0.84397300

H -2.65635400 7.11432500 -0.23718300

H -5.78664600 5.06355500 1.88308400

H -4.86853800 7.15834200 0.90512600

H 4.89112300 -3.58399700 1.66354500

C 2.44784100 -4.81592400 1.53393700

H 2.37793700 -4.96164500 2.61741200

H 1.51381900 -5.16460300 1.09096100

H 3.25952400 -5.46021000 1.16715600

C 3.18354000 3.07109100 -4.01426100

H 3.14251900 2.21789700 -4.69999400

H 2.55163100 3.85851500 -4.44049000

H 4.21480800 3.44613500 -4.00627200

H 1.11875500 1.32444600 -3.20109300

H 0.60434000 3.00808900 -3.06895100

H 3.89351200 -3.07189000 3.03013200

- **1ba**-*SR* (MeH)

B3LYP/6-31G*

E = –3719.222645

Zero-point correction = 1.283761 (Hartree/Particle)

Thermal correction to Energy = 1.358758

Thermal correction to Enthalpy = 1.359702

Thermal correction to Gibbs Free Energy = 1.166085

Sum of electronic and zero-point Energies = –3717.938884

Sum of electronic and thermal Energies = –3717.863887

Sum of electronic and thermal Enthalpies = –3717.862943

Sum of electronic and thermal Free Energies = –3718.056560

PCM(toluene)-B3LYP-D3/6-311++G**//B3LYP/6-31G*@–30 °C

E = –3720.387746

G = –3719.188334

0 1

O -0.89976300 0.12382200 -1.31773700

C -2.04938700 0.24154600 -0.87464200

C -3.08592100 1.22696800 -1.11052100

O -2.51208300 -0.56272600 0.13644700

N -4.21124900 0.83859800 -0.35315600

C -3.82054500 -0.13939500 0.38445700

C -4.59099000 -0.91585300 1.36872700

C -4.77411600 -2.30200700 1.16859500

C -5.18099600 -0.30972900 2.49689000

C -5.50284600 -3.07006800 2.08524400

C -5.91595300 -1.07596100 3.41277400

C -6.06231500 -2.44365600 3.19626100

O -4.22710500 -2.80011500 0.02950600

C -4.39859300 -4.17664500 -0.26949000

H -5.46103300 -4.43674400 -0.36559600

H -3.92923200 -4.81282100 0.49017100

H -3.89832800 -4.33285200 -1.22649500

O -4.97820500 1.02996000 2.63974200

C -5.52630800 1.68302700 3.77450100

H -5.12351700 1.27397200 4.71016000

H -5.23296000 2.73009700 3.68228800

H -6.62157400 1.61304600 3.79030400

H -5.64435000 -4.13277600 1.93252900

H -6.36642100 -0.61888500 4.28460500

H -6.63181400 -3.03468800 3.90876600

C -3.29604600 1.82464300 -2.48530000

H -2.36605300 2.30391400 -2.81303100

H -4.05244500 2.61137800 -2.40301200

C -3.72498400 0.80075600 -3.52455600

C -2.83538100 0.36219200 -4.51339400

C -5.02104700 0.26446100 -3.50720800

C -3.22794400 -0.58187100 -5.46571800

C -5.41513400 -0.68018000 -4.45443600

C -4.52064600 -1.10706100 -5.43928200

H -1.82608200 0.76618800 -4.53762500

H -5.71490000 0.58616800 -2.73532400

H -2.52552000 -0.89800200 -6.23388200

H -6.42503500 -1.08216600 -4.42594800

H -4.83066300 -1.83860100 -6.18130300

C -2.19500400 2.74213000 0.03594300

C -1.97671500 2.34899300 1.39011900

H -1.30191600 2.74137000 -0.59083800

H -2.72869800 2.60791800 2.13025600

C -0.80786100 1.72427900 1.80536300

H -0.08908100 1.46974400 1.02795700

C -0.42721900 1.40621100 3.11112600

H -1.11652000 1.60378600 3.92435900

C 0.87086600 0.92807000 3.40682300

O 1.82664200 0.78403300 2.60253600

N 1.14869100 0.58419100 4.77252200

C 2.43079300 0.39094700 5.25836800

C 0.25096400 0.37145300 5.81036600

C 2.34916500 0.06901300 6.58982300

C 0.96217800 0.05147100 6.93937500

H 3.27231600 0.51959500 4.59805800

H -0.81310800 0.42621200 5.64597900

H 3.18668200 -0.12922300 7.24584800

H 0.53784700 -0.17905500 7.90802400

H 0.43575400 -0.93744300 -0.64934900

N 1.31754500 -1.43454800 -0.44692600

P 2.75046800 -0.71160900 -0.78232200

N 3.64488000 -2.07754200 -0.49110500

C 2.85778100 -3.21181700 0.03999400

C 1.37174700 -2.91326400 -0.45358100

C 5.09484700 -2.17802800 -0.60392200

H 5.35972100 -3.13231000 -1.07505900

H 5.59263000 -2.11572900 0.36921900

H 5.47613700 -1.36503000 -1.22382900

H 3.20956700 -4.09765600 -0.49310300

C 3.07355300 -3.50854300 1.55748200

H 2.17921800 -4.04449600 1.88896200

C 3.22256400 -2.26232700 2.43714900

H 2.39074600 -1.56207100 2.33869500

C 0.31137500 -3.48890800 0.50194100

C -0.02144000 -4.85127200 0.44343700

C -0.29923800 -2.69293200 1.47847300

C -0.92049500 -5.40626000 1.35321100

C -1.21247800 -3.24567500 2.37832500

C -1.52091100 -4.60420200 2.32683700

H 0.42181400 -5.48613200 -0.31757600

H -0.06538800 -1.63746800 1.54573700

H -1.15383700 -6.46666700 1.29649200

H -1.68007900 -2.60303100 3.11824100

H -2.22495700 -5.03359500 3.03477800

C 1.11236600 -3.44321300 -1.89533200

C 0.12134700 -2.81182200 -2.66786900

C 1.76260500 -4.55247100 -2.45511500

C -0.19180700 -3.25994800 -3.95012900

C 1.45321200 -5.00082900 -3.74365500

C 0.47684400 -4.35595300 -4.49905400

H -0.41973100 -1.95844000 -2.27261700

H 2.51537200 -5.10127400 -1.89821900

H -0.96793300 -2.74772300 -4.51292100

H 1.98070400 -5.86063700 -4.14890300

H 0.23569500 -4.70524800 -5.49949300

N 2.79107300 0.74025000 -0.01643800

C 3.67320600 1.72512700 -0.69052700

C 3.35386800 1.40279800 -2.21477400

N 3.28445500 -0.08038300 -2.22070400

H 2.43588800 0.85416800 0.94801700

C 3.71486800 -0.85168400 -3.38031600

H 3.07190800 -0.67691300 -4.24671700

H 3.67307300 -1.91608000 -3.14096100

H 4.74754400 -0.59193700 -3.64894900

H 4.20444600 1.67741100 -2.84541400

C 2.08440300 2.11147000 -2.78652600

H 1.37939400 2.24990800 -1.95932700

C 1.34313400 1.29465400 -3.85728000

H 1.01774200 0.31949600 -3.48803000

C 3.33979500 3.16195300 -0.25538700

C 4.28607900 4.18643400 -0.42378400

C 2.10199700 3.49371700 0.30621400

C 3.99105700 5.50069400 -0.06447200

C 1.80663600 4.80859000 0.67274400

C 2.74732100 5.81927500 0.48478500

H 5.26503300 3.95944300 -0.83313200

H 1.36025900 2.72697300 0.47862000

H 4.74081200 6.27467600 -0.20636400

H 0.83775200 5.02890600 1.11199000

H 2.52051600 6.84218700 0.77283900

C 5.14884800 1.42492600 -0.31307700

C 6.21998800 1.47434000 -1.21397200

C 5.43169600 1.13362500 1.03230300

C 7.52932800 1.21751300 -0.79199300

C 6.73559300 0.88462200 1.45595600

C 7.79287200 0.91913900 0.54287300

H 6.05674200 1.71985700 -2.25858000

H 4.61924600 1.10067900 1.75259300

H 8.34064600 1.25656100 -1.51431100

H 6.92562000 0.66545700 2.50331600

H 8.80970400 0.72197100 0.87123600

C -3.10249300 3.89027600 -0.22702400

C -2.67110000 4.94479400 -1.04835200

C -4.38295200 3.97397800 0.34956700

C -3.48015600 6.05921300 -1.27327700

H -1.68311400 4.89596900 -1.50087800

C -5.19235000 5.08414200 0.11806100

H -4.74875000 3.14318100 0.94412900

C -4.74492700 6.13430900 -0.68902900

H -3.12131200 6.86728500 -1.90595200

H -6.18295700 5.12799200 0.56428800

H -5.37963900 6.99889200 -0.86528200

H 4.14333600 -1.71217400 2.20705700

C 4.25136200 -4.47947400 1.77337900

H 5.21860100 -4.03565500 1.51226400

H 4.30472600 -4.77302700 2.82784100

H 4.13257300 -5.39673500 1.18253400

C 2.44167500 3.48820200 -3.37449700

H 3.10764700 3.37335400 -4.24107200

H 1.53555500 3.99514800 -3.72498600

H 2.93264700 4.14565300 -2.65487000

H 1.95273200 1.15379400 -4.75897900

H 0.44447300 1.84329100 -4.16042000

H 3.27565600 -2.55536600 3.49174400

- **1ba**-*RS* (HH)

B3LYP/6-31G*

E = –3719.222719

Zero-point correction = 1.284169 (Hartree/Particle)

Thermal correction to Energy = 1.359892

Thermal correction to Enthalpy = 1.360837

Thermal correction to Gibbs Free Energy = 1.164424

Sum of electronic and zero-point Energies = –3717.938550

Sum of electronic and thermal Energies = –3717.862826

Sum of electronic and thermal Enthalpies = –3717.861882

Sum of electronic and thermal Free Energies = –3718.058294

PCM(toluene)-B3LYP-D3/6-311++G**//B3LYP/6-31G*@–30 °C

E = –3720.391378

G = –3719.193125

0 1

O 1.05037600 1.27055900 -0.80846500

C 2.06447600 0.58891900 -0.64674500

C 3.38578800 0.90592900 -0.13788500

O 2.05100300 -0.77882600 -0.83253700

N 4.16680900 -0.26457800 -0.23489400

C 3.35541200 -1.20524800 -0.56568700

C 3.65024700 -2.63270000 -0.77662100

C 4.17265400 -3.43959800 0.25760100

C 3.45454800 -3.21086200 -2.04975700

C 4.45487100 -4.79453900 0.03032800

C 3.73617400 -4.56329200 -2.27847900

C 4.22880800 -5.33771400 -1.23142300

O 4.37025400 -2.83205200 1.45879900

C 4.85764900 -3.60755400 2.54320100

H 5.85443000 -4.01546100 2.33178900

H 4.17398400 -4.42975800 2.79126200

H 4.92079800 -2.92256800 3.39031500

O 3.01117000 -2.36195900 -3.01733900

C 2.96694300 -2.82916700 -4.35642600

H 3.94887600 -3.19141700 -4.68733000

H 2.22421300 -3.62718800 -4.48287400

H 2.67711800 -1.96756800 -4.96030800

H 4.84729400 -5.41975900 0.82217600

H 3.58083200 -5.00707400 -3.25371000

H 4.44880300 -6.38786500 -1.40427600

C 4.02425100 2.24669600 -0.43414500

H 3.37771000 3.04319700 -0.04836000

H 4.96764500 2.30303400 0.11750500

C 4.27681800 2.48307400 -1.91488400

C 3.53726700 3.43576100 -2.62641100

C 5.25628700 1.74960700 -2.60105600

C 3.76888600 3.65637100 -3.98605700

C 5.48864800 1.96634300 -3.95881400

C 4.74635900 2.92197400 -4.65780000

H 2.77526500 4.01305800 -2.10729300

H 5.82775100 0.99909000 -2.06172100

H 3.18761100 4.40565600 -4.51872500

H 6.25468800 1.39029500 -4.47253400

H 4.93134000 3.09381700 -5.71524400

C 2.83617900 0.97136600 1.88209700

C 2.34175300 -0.27945300 2.36449900

H 2.07794200 1.73192900 1.69580200

H 3.04472100 -0.94364400 2.85863000

C 1.00503600 -0.65022000 2.32441800

H 0.31146700 0.01502100 1.81254600

C 0.43340700 -1.78208900 2.91765400

H 1.07323800 -2.46635200 3.46418100

C -0.96208100 -1.99317600 2.92367600

O -1.84029400 -1.23962100 2.42083600

N -1.44959000 -3.16372900 3.59608500

C -2.76776900 -3.31078600 3.99366800

C -0.75243600 -4.30678300 3.96024300

C -2.90371100 -4.52918900 4.61104600

C -1.62335600 -5.16565500 4.58296500

H -3.46593500 -2.50850200 3.81845800

H 0.28690300 -4.42907300 3.70087200

H -3.81536700 -4.92339400 5.04068000

H -1.37593600 -6.14719900 4.96612500

H -0.58574300 1.63959500 -0.08550400

N -1.58284300 1.77088200 0.11931800

P -2.68517200 0.78104300 -0.59743900

N -3.98008800 1.65457800 -0.04176000

C -3.59731600 2.67976100 0.96106500

C -2.13418400 3.09664000 0.48557300

C -5.36845000 1.50362200 -0.46206500

H -5.43044000 0.76692300 -1.26437600

H -6.00747500 1.15947100 0.35459200

H -5.75434800 2.46209600 -0.83362000

H -4.28542700 3.51837600 0.82451400

C -3.70973700 2.20780200 2.44501700

H -2.77307900 1.70915800 2.71301700

C -4.82376900 1.17805100 2.69451500

H -5.82195600 1.59962400 2.51950200

C -2.16885200 4.01678500 -0.76429700

C -3.19680300 4.93317100 -1.02610000

C -1.08721900 3.96997600 -1.66130100

C -3.16220900 5.75616000 -2.15652900

C -1.05006300 4.79279400 -2.78646100

C -2.09071800 5.68745100 -3.04444900

H -4.03835800 5.03282800 -0.34796200

H -0.26052000 3.28794800 -1.48481000

H -3.97746600 6.45263300 -2.33482900

H -0.20091000 4.73140400 -3.46197600

H -2.06316900 6.32605200 -3.92316700

C -1.26123200 3.73706700 1.58004500

C -0.53953800 2.94356000 2.48291300

C -1.18358600 5.13177300 1.71537700

C 0.21965500 3.52385900 3.50089400

C -0.42673900 5.71257300 2.73353000

C 0.27767100 4.91143900 3.63315900

H -0.57416100 1.86402900 2.40029500

H -1.71326300 5.77451800 1.02071300

H 0.76751300 2.88241800 4.18543900

H -0.38471700 6.79541500 2.81657400

H 0.86869400 5.36343000 4.42514200

N -2.28901600 -0.76882900 -0.25554700

C -2.75691900 -1.72521700 -1.28764900

C -2.49060100 -0.88211300 -2.61054600

N -2.90292200 0.49047100 -2.21268000

H -2.08706200 -1.05138400 0.72303900

C -3.47209900 1.41442000 -3.18653100

H -4.33479400 0.95296200 -3.68518800

H -2.74184800 1.70576900 -3.94506700

H -3.80616700 2.32176100 -2.67985200

H -3.18450600 -1.19179900 -3.39699900

C -1.04302300 -0.99514200 -3.18343500

C -0.52673700 0.28105500 -3.86657700

H -1.08298500 0.51448700 -4.78389600

C -1.96375400 -3.03991300 -1.18664600

C -0.68878100 -3.08079000 -0.60694200

C -2.51001500 -4.23523900 -1.68130600

C 0.02268300 -4.28040000 -0.53255200

C -1.79604000 -5.43089400 -1.61307500

C -0.52507500 -5.45869100 -1.03656200

H -0.23624700 -2.18023700 -0.20985600

H -3.50463800 -4.23858400 -2.11536300

H 1.00816000 -4.28337800 -0.07677200

H -2.24190900 -6.34311000 -2.00122800

H 0.02902500 -6.39147000 -0.97438900

C -4.26426700 -2.02401800 -1.06506700

C -4.69173900 -2.28239100 0.24940400

C -5.21479900 -2.10022400 -2.09090800

C -6.02290800 -2.58717700 0.52581700

C -6.55351700 -2.40032300 -1.81394500

C -6.96436000 -2.64216300 -0.50537600

H -3.97293300 -2.24794400 1.06352100

H -4.93147700 -1.93806800 -3.12591600

H -6.32275100 -2.78805100 1.55086800

H -7.26983700 -2.44812500 -2.63022600

H -8.00297600 -2.87837800 -0.29015000

C 4.07663400 1.50858000 2.50374600

C 4.11339400 2.83796500 2.95586800

C 5.22330900 0.71213700 2.67844800

C 5.24857100 3.35223500 3.58402200

H 3.23674800 3.46801600 2.82520900

C 6.35785700 1.22945900 3.29984300

H 5.22821400 -0.29953900 2.28546600

C 6.37555500 2.54914200 3.76106700

H 5.25158200 4.38207600 3.93271200

H 7.23801500 0.60207800 3.41839100

H 7.26248200 2.94812900 4.24648300

H -0.55359600 1.14669000 -3.20087000

C -0.96084900 -2.16514700 -4.17917000

H -1.60667200 -1.97757800 -5.04873100

H 0.06402300 -2.27220700 -4.54897700

H -1.25644100 -3.11908300 -3.73689700

H -0.36013500 -1.19219100 -2.35170100

H -4.70508300 0.28047600 2.08254000

C -3.92853300 3.40900400 3.38224600

H -4.90048100 3.88041700 3.17977000

H -3.93778800 3.07613500 4.42605900

H -3.15597100 4.17435800 3.28567200

H -4.78581700 0.86357800 3.74329300

H 0.52033500 0.13007400 -4.15044700

- **1ba**-*RS* (MeH)

B3LYP/6-31G*

E = –3719.223894

Zero-point correction = 1.283967 (Hartree/Particle)

Thermal correction to Energy = 1.359819

Thermal correction to Enthalpy = 1.360763

Thermal correction to Gibbs Free Energy = 1.164371

Sum of electronic and zero-point Energies = –3717.939928

Sum of electronic and thermal Energies = –3717.864076

Sum of electronic and thermal Enthalpies = –3717.863131

Sum of electronic and thermal Free Energies = –3718.059523

PCM(toluene)-B3LYP-D3/6-311++G**//B3LYP/6-31G*@–30 °C

E = –3720.391948

G = –3719.193754

0 1

O 0.92027800 1.17013700 -0.81019400

C 2.00085200 0.59434400 -0.66807500

C 3.29000600 1.03250500 -0.16030900

O 2.11894100 -0.76222200 -0.87598600

N 4.18018500 -0.05595900 -0.28703000

C 3.46038900 -1.06635200 -0.62488000

C 3.88137000 -2.45900500 -0.84825900

C 4.53057000 -3.20495600 0.16035000

C 3.66521900 -3.06919900 -2.10415600

C 4.92303200 -4.53045500 -0.07491400

C 4.05219100 -4.39436400 -2.33828600

C 4.67453100 -5.10750500 -1.31691700

O 4.73098400 -2.57365900 1.34891100

C 5.33528700 -3.29590700 2.41135400

H 6.35417000 -3.61324100 2.15500600

H 4.74059400 -4.17497800 2.69134000

H 5.37445900 -2.60282100 3.25327800

O 3.09584500 -2.27442800 -3.05006500

C 2.91738900 -2.79597500 -4.35701000

H 3.87241200 -3.10676900 -4.80035900

H 2.22050700 -3.64376300 -4.36174600

H 2.49646700 -1.97871800 -4.94511500

H 5.41384400 -5.10854900 0.69761800

H 3.88234100 -4.86225000 -3.29965100

H 4.97873600 -6.13552900 -1.49491100

C 3.79521200 2.43359600 -0.43432300

H 3.08767500 3.15748200 -0.01391600

H 4.73893500 2.56098900 0.10555600

C 4.00237400 2.74204900 -1.90915600

C 3.24650800 3.73360500 -2.54674000

C 4.96542500 2.05086800 -2.66003800

C 3.44308500 4.03067000 -3.89758400

C 5.16141500 2.34300300 -4.00937200

C 4.40107000 3.33451000 -4.63490800

H 2.50166000 4.28418400 -1.97586000

H 5.55428000 1.27566700 -2.17751600

H 2.85109300 4.81046800 -4.37161000

H 5.91404000 1.79782200 -4.57401600

H 4.55799100 3.56452700 -5.68577600

C 2.76722400 1.00853800 1.84875900

C 2.38162200 -0.29260400 2.30012400

H 1.94420600 1.70799400 1.69903700

H 3.14516300 -0.91815400 2.75313500

C 1.07991300 -0.77098200 2.25997800

H 0.32230000 -0.13970500 1.79653900

C 0.62205900 -1.98113600 2.79446400

H 1.33249100 -2.63197300 3.29220300

C -0.74461600 -2.33104900 2.79211500

O -1.69939000 -1.64288000 2.33920900

N -1.10516500 -3.58053500 3.39884200

C -2.39540000 -3.88199000 3.80013000

C -0.29228600 -4.66792600 3.68586100

C -2.39979900 -5.14193900 4.34460600

C -1.06341200 -5.64464500 4.26482800

H -3.17355600 -3.14586900 3.68081600

H 0.74976500 -4.67009000 3.40905500

H -3.26097500 -5.64968300 4.75893900

H -0.71367600 -6.61729400 4.58595200

H -0.68423700 1.59375500 -0.02582600

N -1.67273800 1.71935600 0.21782200

P -2.78288900 0.73948100 -0.50330900

N -4.07736100 1.60515800 0.07234500

C -3.70569100 2.70320900 0.99221800

C -2.20715400 3.05032700 0.57680800

C -5.47704000 1.25655600 -0.14232100

H -5.56563400 0.60350300 -1.01256300

H -5.91132200 0.73024000 0.71422700

H -6.05820700 2.16773400 -0.32834100

H -4.33099500 3.55622500 0.71882600

C -3.99209000 2.42161200 2.50125700

H -3.32061400 3.07781200 3.06377900

C -3.72178600 0.97849600 2.94826100

H -4.41443400 0.27215600 2.47602700

C -2.14881000 3.99316100 -0.66069900

C -3.12552400 4.95528400 -0.95502600

C -1.02975200 3.91897900 -1.50914500

C -3.00663500 5.79379800 -2.06788800

C -0.90987500 4.75529300 -2.61872800

C -1.90064000 5.69528000 -2.90876400

H -3.99505500 5.08104800 -0.31772300

H -0.23584600 3.20490100 -1.31258900

H -3.78514800 6.52498400 -2.26989400

H -0.03436100 4.66514100 -3.25631100

H -1.80839400 6.34452100 -3.77518500

C -1.39311800 3.65912200 1.73248700

C -0.65307700 2.84618400 2.60205800

C -1.41208300 5.04157700 1.96970100

C 0.04456800 3.39745000 3.67865300

C -0.71986500 5.59235800 3.04863600

C 0.01345300 4.77335400 3.90806500

H -0.63007900 1.77391900 2.44931300

H -1.96043600 5.69908400 1.30326500

H 0.61314200 2.74318300 4.33354400

H -0.74936800 6.66674100 3.21008600

H 0.55619800 5.20259900 4.74587700

N -2.35226000 -0.80983100 -0.21421000

C -2.79607200 -1.75200400 -1.27223500

C -2.60649300 -0.85763600 -2.57601300

N -3.04657100 0.48570400 -2.11901700

H -2.06178100 -1.14030100 0.72619700

C -3.70661000 1.41321700 -3.03008600

H -4.52860400 0.90504000 -3.55084200

H -3.01744200 1.82055000 -3.77416000

H -4.11770700 2.24857500 -2.46019000

H -3.32028500 -1.16726200 -3.34462300

C -1.18386600 -0.89529900 -3.21594000

C -0.77829100 0.41203500 -3.91513100

H -1.40808900 0.62463300 -4.78891300

C -1.93261800 -3.02419600 -1.23644400

C -0.62317400 -3.00024500 -0.73894600

C -2.44074100 -4.24046800 -1.71955600

C 0.15995000 -4.15603300 -0.73553500

C -1.65625100 -5.39342400 -1.72074000

C -0.35082000 -5.35596700 -1.22823900

H -0.19950000 -2.08267800 -0.34963500

H -3.45858200 -4.29385100 -2.09197800

H 1.17036700 -4.10702200 -0.34160100

H -2.07339800 -6.32378500 -2.09756600

H 0.25840900 -6.25586300 -1.21974800

C -4.27982500 -2.12987900 -1.01604800

C -4.64588400 -2.46220300 0.30053600

C -5.26458800 -2.19771400 -2.00913900

C -5.95339700 -2.82930700 0.61107500

C -6.58009400 -2.56050600 -1.69710800

C -6.93127200 -2.87424800 -0.38654100

H -3.89518600 -2.43773800 1.08595400

H -5.02746400 -1.97892800 -3.04529200

H -6.20707100 -3.08699500 1.63598900

H -7.32499200 -2.59985600 -2.48785000

H -7.95172400 -3.15863900 -0.14424300

C 3.96291400 1.63215100 2.48114400

C 3.88490200 2.94220400 2.98153300

C 5.17770400 0.93609800 2.62031200

C 4.97486000 3.53345500 3.62204600

H 2.95449400 3.49572900 2.88015200

C 6.26675000 1.53071300 3.25415600

H 5.26971300 -0.05611100 2.19056100

C 6.17063700 2.82907000 3.76357700

H 4.88830400 4.54620700 4.00810600

H 7.20045300 0.98081300 3.34482100

H 7.02256400 3.28814200 4.25850400

H -0.80638700 1.26930900 -3.23845800

C -1.08561900 -2.05271300 -4.22510100

H -1.77619000 -1.89082200 -5.06488300

H -0.07323600 -2.10533800 -4.63972500

H -1.31413400 -3.02365500 -3.78069600

H -0.45151100 -1.05683700 -2.41958000

H -2.70900900 0.63479700 2.72865100

C -5.42214000 2.85282300 2.88228600

H -6.18966100 2.23822400 2.39907400

H -5.56585700 2.75867600 3.96457200

H -5.61252000 3.89984400 2.61456800

H -3.86619200 0.89575000 4.03164200

H 0.25304500 0.31906000 -4.27242500

- **1ba**-*RS* (HMe)

B3LYP/6-31G*

E = –3719.222486

Zero-point correction = 1.284479 (Hartree/Particle)

Thermal correction to Energy = 1.360212

Thermal correction to Enthalpy = 1.361156

Thermal correction to Gibbs Free Energy = 1.164447

Sum of electronic and zero-point Energies = –3717.938007

Sum of electronic and thermal Energies = –3717.862274

Sum of electronic and thermal Enthalpies = –3717.861330

Sum of electronic and thermal Free Energies = –3718.058038

PCM(toluene)-B3LYP-D3/6-311++G**//B3LYP/6-31G*@–30 °C

E = –3720.390774

G = –3719.192443

0 1

O -1.20082400 1.29523500 0.60827900

C -2.16265700 0.52527000 0.60155800

C -3.55523100 0.71199900 0.23819500

O -2.01436100 -0.82766500 0.83384100

N -4.22035200 -0.50697100 0.47321700

C -3.30115100 -1.36798500 0.73740600

C -3.46015000 -2.80619900 1.00762200

C -4.12979800 -3.65209500 0.09344800

C -2.97876500 -3.36810300 2.21198100

C -4.29027300 -5.01748600 0.36899100

C -3.13607800 -4.73307900 2.48535500

C -3.78989100 -5.53956100 1.55798000

O -4.58255800 -3.07422500 -1.05199300

C -5.22541800 -3.88962100 -2.01967900

H -6.14183200 -4.34278600 -1.62035800

H -4.55976300 -4.68068000 -2.38845600

H -5.48267200 -3.22115100 -2.84293700

O -2.39276900 -2.49603700 3.07606600

C -1.88293700 -2.99781300 4.30123400

H -2.67293100 -3.45666100 4.90998300

H -1.08050300 -3.72695300 4.13168200

H -1.48078800 -2.13242400 4.83019200

H -4.79651500 -5.66869200 -0.33203300

H -2.76618000 -5.16199500 3.40784400

H -3.91677600 -6.59816700 1.76865500

C -4.26672200 2.01642800 0.52548500

H -3.74913300 2.82881300 0.00275200

H -5.27362900 1.95173200 0.10119000

C -4.34980200 2.35166900 2.00623200

C -3.61763900 3.41879600 2.54128500

C -5.16077000 1.59872100 2.86839400

C -3.69430500 3.73232100 3.90022200

C -5.23743600 1.90790700 4.22604900

C -4.50519700 2.97750200 4.74815000

H -2.98442000 4.01063300 1.88405600

H -5.72487200 0.76163100 2.46585200

H -3.12379400 4.57032300 4.29466300

H -5.87377000 1.31463000 4.87841100

H -4.56941800 3.22103600 5.80577000

C -3.21455000 0.70162800 -1.82916400

C -2.65487300 -0.53195500 -2.28685900

H -2.51070400 1.52980000 -1.75022100

H -3.32806700 -1.27402000 -2.70642100

C -1.29151000 -0.78752500 -2.28604200

H -0.64736900 -0.03843500 -1.83057400

C -0.62739500 -1.88846400 -2.83907300

H -1.20120400 -2.66236600 -3.33752100

C 0.78138000 -1.95381400 -2.85939900

O 1.58466900 -1.08798300 -2.41468500

N 1.37708000 -3.10511800 -3.48050000

C 2.67128000 -3.11317300 -3.97215600

C 0.81408800 -4.35138700 -3.70608900

C 2.92400800 -4.35085400 -4.51195900

C 1.74572000 -5.14061500 -4.33503700

H 3.26297500 -2.21339100 -3.91877400

H -0.17860300 -4.58102700 -3.35252600

H 3.84550400 -4.65796100 -4.98921900

H 1.60540700 -6.17383200 -4.62507200

H 0.54406600 1.58395700 0.10935600

N 1.51883300 1.82635300 -0.10914500

P 2.77505700 0.94369000 0.46890400

N 3.91792800 1.91511300 -0.23766700

C 3.34428900 2.89035200 -1.19554600

C 1.90132800 3.17999200 -0.57965600

C 5.34589100 1.91114700 0.05724600

H 5.55298300 1.17338300 0.83399700

H 5.94198900 1.64906800 -0.82009200

H 5.66130900 2.90027800 0.41541400

H 3.96042800 3.79096100 -1.12760300

C 3.36566500 2.41649500 -2.68360500

H 2.45600600 1.83859200 -2.86958900

C 4.53687800 1.48076100 -3.02654800

H 5.50698800 1.98871000 -2.95266400

C 1.95774800 4.14202700 0.63653400

C 2.94288300 5.12040100 0.82226800

C 0.92844600 4.05573700 1.59112300

C 2.92008700 5.96637600 1.93692400

C 0.90210100 4.90069200 2.69903600

C 1.90289800 5.85812900 2.88229700

H 3.74100700 5.25246800 0.09879300

H 0.13402900 3.32536100 1.46683900

H 3.70178600 6.71213800 2.05663400

H 0.09276500 4.80927400 3.41867800

H 1.88476800 6.51517500 3.74758900

C 0.86933500 3.68678000 -1.60192400

C 0.17995700 2.79157100 -2.43151300

C 0.61744500 5.05905400 -1.75065400

C -0.71697300 3.25439500 -3.39578400

C -0.27995200 5.52236900 -2.71366700

C -0.94964900 4.62228900 -3.54335100

H 0.35172300 1.72590000 -2.33731500

H 1.12108100 5.77585400 -1.11100500

H -1.23387700 2.53685100 -4.02684200

H -0.45655600 6.59045100 -2.80955600

H -1.64745100 4.98260700 -4.29451000

N 2.44172700 -0.63322500 0.19009900

C 3.10527800 -1.55107000 1.13934300

C 3.09812800 -0.71004100 2.48935300

N 3.26868500 0.68897600 2.03286400

H 2.13837900 -0.93166200 -0.75584100

C 3.67311800 1.73829300 2.96047200

H 4.55978800 1.41781100 3.52105100

H 2.88017600 1.99076000 3.67216400

H 3.91976200 2.64472000 2.40396500

H 4.00101400 -0.96681100 3.04886900

C 1.90555200 -0.93664900 3.46995600

C 0.64531700 -0.13575500 3.12529500

H 0.81594200 0.94448900 3.19489200

C 2.32876400 -2.87197200 1.25316200

C 1.01632400 -2.99965400 0.78286600

C 2.93575100 -3.98090600 1.86397800

C 0.33068800 -4.20984900 0.91556800

C 2.24508700 -5.18246300 2.00997800

C 0.93833500 -5.30279400 1.53185000

H 0.51299000 -2.16101700 0.31684200

H 3.96057100 -3.91044200 2.21659800

H -0.68260000 -4.28758900 0.53470300

H 2.73513100 -6.02864200 2.48479500

H 0.40154100 -6.24241900 1.63194900

C 4.54322800 -1.85930300 0.62859700

C 4.68160500 -2.21943900 -0.72379900

C 5.69725800 -1.84664500 1.42182300

C 5.92494500 -2.54341900 -1.26067000

C 6.94901400 -2.16622600 0.88238600

C 7.06981600 -2.51458100 -0.45995600

H 3.80789900 -2.25100400 -1.36786300

H 5.65076100 -1.59733500 2.47693300

H 5.99306700 -2.82791200 -2.30713900

H 7.82662000 -2.14405700 1.52353100

H 8.04074900 -2.76702200 -0.87751700

C -4.54558800 1.10163500 -2.35845700

C -4.72677300 2.38685100 -2.89519500

C -5.63921900 0.21676200 -2.36049500

C -5.95206000 2.77141000 -3.44132700

H -3.89259800 3.08472100 -2.89496100

C -6.86409800 0.60519300 -2.89936500

H -5.52768400 -0.75745300 -1.89555900

C -7.02592700 1.88067600 -3.44830300

H -6.06710100 3.76874400 -3.85878300

H -7.70173900 -0.08806800 -2.88364000

H -7.98277100 2.17886400 -3.86905100

H 0.27064500 -0.35290600 2.12456500

C 2.33242700 -0.70228300 4.93260600

H 2.56974700 0.34665200 5.14093400

H 1.52014500 -0.98900300 5.61038900

H 3.21075000 -1.30388800 5.19913600

H 1.65578100 -1.99980900 3.39541800

H 4.55551700 0.58855800 -2.39513000

C 3.39873200 3.62404000 -3.63680300

H 4.34031500 4.17831400 -3.51880100

H 3.34569400 3.28599900 -4.67757300

H 2.57422900 4.32064500 -3.47164100

H 4.42852700 1.14460300 -4.06371500

H -0.15969300 -0.38011200 3.82667500

- **1ba**-*RS* (MeMe)

B3LYP/6-31G*

E = –3719.223609

Zero-point correction = 1.284095 (Hartree/Particle)

Thermal correction to Energy = 1.360001

Thermal correction to Enthalpy = 1.360945

Thermal correction to Gibbs Free Energy = 1.164096

Sum of electronic and zero-point Energies = –-3717.939514

Sum of electronic and thermal Energies = –3717.863608

Sum of electronic and thermal Enthalpies = –3717.862664

Sum of electronic and thermal Free Energies = –3718.059513

PCM(toluene)-B3LYP-D3/6-311++G**//B3LYP/6-31G*@–30 °C

E = –3720.391858

G = –3719.193855

0 1

O -1.15720700 1.28400800 0.58871800

C -2.13710400 0.53793200 0.60213300

C -3.52170400 0.74595700 0.21540700

O -2.02264000 -0.80518500 0.88533900

N -4.21836800 -0.44703500 0.49643200

C -3.32155000 -1.31988600 0.79694400

C -3.51086900 -2.74468400 1.11031200

C -4.23311400 -3.59434200 0.23966700

C -2.99983900 -3.29259800 2.30970300

C -4.41672600 -4.94855900 0.55259400

C -3.17790200 -4.64749800 2.61771300

C -3.88470000 -5.45741900 1.73337000

O -4.71010100 -3.03323000 -0.90397600

C -5.40503500 -3.85537000 -1.82932700

H -6.32094100 -4.27096500 -1.39004600

H -4.77374500 -4.67485500 -2.19628200

H -5.66854700 -3.20174900 -2.66244800

O -2.36461200 -2.41752500 3.13466200

C -1.79836800 -2.91026400 4.33879300

H -2.56271100 -3.34219100 4.99800400

H -1.02119000 -3.65827700 4.13785100

H -1.35025200 -2.04471200 4.82909300

H -4.96364200 -5.60214800 -0.11480700

H -2.78508700 -5.06499700 3.53588400

H -4.02848800 -6.50787800 1.97197000

C -4.20102700 2.07817200 0.45181700

H -3.66801800 2.85531700 -0.10753500

H -5.21036400 2.01858300 0.03209300

C -4.27287000 2.48256000 1.91602800

C -3.57940400 3.60616200 2.38299100

C -5.04100600 1.74480200 2.82923700

C -3.65140300 3.98877500 3.72461200

C -5.11202000 2.12254000 4.16981600

C -4.41792000 3.24710200 4.62389800

H -2.98164500 4.18985800 1.68627300

H -5.57652700 0.86615300 2.47996300

H -3.11422300 4.87163900 4.06434700

H -5.71462500 1.53937500 4.86207000

H -4.47809800 3.54359500 5.66810400

C -3.16891100 0.64622600 -1.82543100

C -2.65275700 -0.62737600 -2.22709100

H -2.43505400 1.45141200 -1.78616400

H -3.35570200 -1.36412900 -2.60543200

C -1.30265800 -0.94356200 -2.21511500

H -0.61571100 -0.20044300 -1.81281600

C -0.70644700 -2.11438700 -2.69944000

H -1.33237200 -2.88032600 -3.14437400

C 0.69366500 -2.27619700 -2.72050500

O 1.55879800 -1.44332100 -2.33394900

N 1.20267900 -3.49966600 -3.27671000

C 2.48505800 -3.62598900 -3.78187700

C 0.55270000 -4.71557800 -3.42113800

C 2.64483300 -4.90728900 -4.24986300

C 1.41860300 -5.60247100 -4.01259600

H 3.13873600 -2.76863300 -3.78906400

H -0.44787900 -4.85595700 -3.04379000

H 3.53645900 -5.30455700 -4.71716800

H 1.20379800 -6.63881100 -4.23904600

H 0.57487000 1.58954100 0.08126500

N 1.53394000 1.83846600 -0.19077500

P 2.81684900 0.99298800 0.38851000

N 3.93452000 1.99092900 -0.32747500

C 3.34781600 3.02201400 -1.20965200

C 1.87021400 3.20703600 -0.64275700

C 5.37855500 1.81043300 -0.24541900

H 5.61796400 1.17964300 0.61296900

H 5.78963900 1.32974400 -1.13971600

H 5.86522900 2.78430800 -0.11417000

H 3.89574800 3.94632700 -1.01204800

C 3.51298600 2.74430600 -2.73800600

H 2.71732200 3.30562400 -3.23712100

C 3.37212900 1.27000100 -3.14060000

H 4.17342500 0.65524900 -2.71413700

C 1.81214400 4.17194000 0.57662800

C 2.74809800 5.18168600 0.83503300

C 0.72346700 4.04658100 1.45941800

C 2.62131600 6.01929900 1.94894600

C 0.59521200 4.88140100 2.56790200

C 1.54833100 5.87039900 2.82368400

H 3.59101900 5.34715500 0.17195100

H -0.04021100 3.29520100 1.28166200

H 3.36796800 6.78968800 2.12391500

H -0.25751400 4.75466400 3.22983100

H 1.45023900 6.51981600 3.68940300

C 0.88272700 3.68040200 -1.72195900

C 0.17611900 2.76383800 -2.51128800

C 0.71367600 5.04930000 -1.97667800

C -0.67118100 3.20211300 -3.53072000

C -0.13034600 5.48796900 -2.99731800

C -0.82781900 4.56628300 -3.77925500

H 0.29811300 1.70077400 -2.34302200

H 1.23560300 5.78191200 -1.36948000

H -1.20607000 2.46908100 -4.12816300

H -0.24565500 6.55397900 -3.17491100

H -1.48621700 4.90731700 -4.57366300

N 2.50004300 -0.59331400 0.15086200

C 3.16528800 -1.48751500 1.12434600

C 3.18077600 -0.60810200 2.44894500

N 3.34428100 0.77483400 1.94805900

H 2.14348200 -0.94610000 -0.75660800

C 3.79230000 1.84571200 2.82934200

H 4.66291700 1.51052900 3.40568400

H 3.01074800 2.16530500 3.52707400

H 4.08127700 2.71204700 2.23079600

H 4.09352700 -0.84912300 2.99918300

C 2.00883200 -0.80509800 3.45984000

C 0.73907400 -0.01899800 3.11627500

H 0.90720200 1.06331600 3.15353300

C 2.37881300 -2.79721400 1.28855500

C 1.05148200 -2.92182900 0.86164900

C 2.99188900 -3.89776000 1.90834700

C 0.35788000 -4.12093900 1.04241500

C 2.29375400 -5.08819300 2.10296700

C 0.97256700 -5.20600700 1.66583100

H 0.54278000 -2.09131700 0.38808000

H 4.02725600 -3.83016700 2.22918000

H -0.66598900 -4.19629400 0.69047200

H 2.78936100 -5.92827800 2.58276500

H 0.43059200 -6.13818300 1.80151200

C 4.59380400 -1.81970200 0.60342900

C 4.70717500 -2.22688200 -0.73810100

C 5.76179600 -1.77571200 1.37455800

C 5.94234200 -2.56304400 -1.28629200

C 7.00483200 -2.10866400 0.82313000

C 7.10203400 -2.50132200 -0.50879200

H 3.81853600 -2.28347600 -1.36022700

H 5.73408300 -1.48954300 2.42092000

H 5.99503800 -2.88202100 -2.32378200

H 7.89415400 -2.06115900 1.44650500

H 8.06628600 -2.76315900 -0.93601700

C -4.47961600 1.07085300 -2.38992700

C -4.60562400 2.33396700 -2.99087300

C -5.60688700 0.22995500 -2.36112100

C -5.80999200 2.73803900 -3.56882000

H -3.74548500 2.99909400 -3.01520000

C -6.81071100 0.63816500 -2.93202400

H -5.53805000 -0.72400700 -1.84841200

C -6.91779000 1.89031800 -3.54432100

H -5.88199800 3.71736400 -4.03566600

H -7.67472600 -0.02091500 -2.89184200

H -7.85831300 2.20405600 -3.98981100

H 0.34529400 -0.26412500 2.12932800

C 2.46331300 -0.51748200 4.90455200

H 2.69393800 0.54037300 5.07221200

H 1.66755100 -0.78797000 5.60811600

H 3.35244200 -1.10185200 5.17357100

H 1.76256000 -1.87118500 3.42664400

H 2.42338500 0.82254500 -2.83779600

C 4.83786100 3.33012000 -3.26642400

H 5.71754300 2.81716400 -2.86184900

H 4.88062500 3.23309200 -4.35710500

H 4.93154100 4.39697300 -3.02710500

H -0.05131700 -0.24612900 3.84007900

H 3.44084000 1.17893000 -4.23079300

**Supplymentary References:**

(1) Gaussian 09, Revision D.01, Frisch, M. J., Trucks, G. W., Schlegel, H. B., Scuseria, G. E., Robb, M. A., Cheeseman, J. R., Scalmani, G., Barone, V., Mennucci, B., Petersson, G. A., Nakatsuji, H., Caricato, M., Li, X., Hratchian, H. P., Izmaylov, A. F., Bloino, J., Zheng, G., Sonnenberg, J. L., Hada, M., Ehara, M., Toyota, K., Fukuda, R., Hasegawa, J., Ishida, M., Nakajima, T., Honda, Y., Kitao, O., Nakai, H., Vreven, T., Montgomery, Jr., J. A., Peralta, J. E., Ogliaro, F., Bearpark, M., Heyd, J. J., Brothers, E., Kudin, K. N., Staroverov, V. N., Keith, T., Kobayashi, R., Normand, J., Raghavachari, K., Rendell, A., Burant, J. C., Iyengar, S. S., Tomasi, J., Cossi, M., Rega, N., Millam, J. M., Klene, M., Knox, J. E., Cross, J. B., Bakken, V., Adamo, C., Jaramillo, J., Gomperts, R., Stratmann, R. E., Yazyev, O., Austin, A. J., Cammi, R., Pomelli, C., Ochterski, J. W., Martin, R. L., Morokuma, K., Zakrzewski, V. G., Voth, G. A., Salvador, P., Dannenberg, J. J., Dapprich, S., Daniels, A. D., Farkas, O., Foresman, J. B., Ortiz, J. V., Cioslowski, J. & Fox, D. J. Gaussian, Inc., Wallingford CT, 2013.

(2) CYLview, 1.0b, Legault, C. Y. Université de Sherbrooke, 2009.
